# Vascular plants of Victoria Island (Northwest Territories and Nunavut, Canada): a specimen-based study of an Arctic flora

**DOI:** 10.3897/phytokeys.141.48810

**Published:** 2020-03-06

**Authors:** Jeffery M. Saarela, Paul C. Sokoloff, Lynn J. Gillespie, Roger D. Bull, Bruce A. Bennett, Serguei Ponomarenko

**Affiliations:** 1 Centre for Arctic Knowledge and Exploration and Botany Section, Research & Collections, Canadian Museum of Nature, Ottawa, Ontario, Canada Canadian Museum of Nature Ottawa Canada; 2 33 Chinook Lane, Whitehorse, Yukon Unaffiliated Whitehorse Canada

**Keywords:** biodiversity, Canadian Arctic Archipelago, checklist, floristics, herbarium, natural history collections, range extension, taxonomy

## Abstract

Victoria Island in Canada’s western Arctic is the eighth largest island in the world and the second largest in Canada. Here, we report the results of a floristic study of vascular plant diversity of Victoria Island. The study is based on a specimen-based dataset comprising 7031 unique collections from the island, including some 2870 new collections gathered between 2008 and 2019 by the authors and nearly 1000 specimens variously gathered by N. Polunin (in 1947), M. Oldenburg (1940s–1950s) and S. Edlund (1980s) that, until recently, were part of the unprocessed backlog of the National Herbarium of Canada and unavailable to researchers. Results are presented in an annotated checklist, including keys and distribution maps for all taxa, citation of specimens, comments on taxonomy, distribution and the history of documentation of taxa across the island, and photographs for a subset of taxa. The vascular plant flora of Victoria Island comprises 38 families, 108 genera, 272 species, and 17 additional taxa. Of the 289 taxa known on the island, 237 are recorded from the Northwest Territories portion of the island and 277 from the Nunavut part. Thirty-nine taxa are known on the island from a single collection, seven from two collections and three from three collections. Twenty-one taxa in eight families are newly recorded for the flora of Victoria Island: *Artemisia tilesii*, *Senecio lugens*, *Taraxacum scopulorum* (Asteraceae); *Crucihimalaya bursifolia*, *Draba fladnizensis*, *D. juvenilis*, *D. pilosa*, *D. simmonsii* (Brassicaceae); Carex bigelowii subsp. bigelowii, Eriophorum russeolum subsp. albidum (Cyperaceae); Anthoxanthum monticola subsp. monticola, *Bromus pumpellianus*, Deschampsia cespitosa subsp. cespitosa, *D. sukatschewii*, Festuca rubra subsp. rubra, *Lolium perenne*, Poa pratensis subsp. pratensis (Poaceae); *Stuckenia filiformis* (Potamogetonaceae); Potentilla × prostrata (Rosaceae); *Galium aparine* (Rubiaceae); and Salix ovalifolia var. ovalifolia (Salicaceae). Eight of these are new to the flora of the Canadian Arctic Archipelago: *Senecio lugens*, *Draba juvenilis*, *D. pilosa*, Anthoxanthum monticola subsp. monticola, *Bromus pumpellianus*, Deschampsia cespitosa subsp. cespitosa, Poa pratensis subsp. pratensis and Salix ovalifolia var. ovalifolia. One of these, *Galium aparine*, is newly recorded for the flora of Nunavut. Four first records for Victoria Island are introduced plants discovered in Cambridge Bay in 2017: three grasses (Festuca rubra subsp. rubra, *Lolium perenne*, and Poa pratensis subsp. pratensis) and *Galium aparine*. One taxon, Juncus arcticus subsp. arcticus, is newly recorded from the Northwest Territories. Of the general areas on Victoria Island that have been botanically explored the most, the greatest diversity of vascular plants is recorded in Ulukhaktok (194 taxa) and the next most diverse area is Cambridge Bay (183 taxa). The floristic data presented here represent a new baseline on which continued exploration of the vascular flora of Victoria Island – particularly the numerous areas of the island that remain unexplored or poorly explored botanically – will build.

## Introduction

The Canadian Arctic Archipelago is a large group of islands occupying the northern third of Canada and comprising 94 islands greater than 130 km^2^ and 36,469 smaller islands (Adams and Dunbar 2016). The Archipelago covers approximately 1.42 million km^2^. The Archipelago is fully within Inuit Nunangat, the Inuit homeland, a broader Arctic region encompassing approximately 35 percent of Canada’s landmass and 50 percent of its coastline.

The flora of an area (whether local, regional, national, continental or global) refers to all plant species and taxa at other ranks occurring in the area; it is a principal measure of biodiversity. Exploration and documentation of the vascular plant flora of the Canadian Arctic Archipelago has been ongoing since the earliest expeditions in search of the Northwest Passage, nearly 200 years ago, during which crew members obtained new scientific information on the natural history of the lands being explored, including collections of plants. Vascular plant specimens have accumulated from across the Archipelago through the decades, variously collected opportunistically or as part of botanical studies by both botanists and non-botanists alike. Many floristic studies of areas of the Canadian Arctic Archipelago have been published, ranging from simple lists of plants to more detailed accounts of plant biodiversity, including information about taxonomy, nomenclature, distribution and ecology (Polunin 1934, 1938b, a, 1939, 1940b, 1947, Cody 1951b, a, Brassard and Beschel 1968, Brassard and Longton 1970, Kuc 1970, Mason et al. 1972, Kuc 1974, Cody et al. 1976, Gillett 1976, Bridgland and Gillett 1983, Cody et al. 1984b, Soper and Powell 1985, Schwarzenbach 2010). Among the most important taxonomic treatments of (or relevant to) the vascular flora of the Canadian Arctic Archipelago published prior to the middle of the twentieth century are works by Brown (1823), Richardson and Brown (1823), Simmons (1906), Simmons (1913) and Polunin (1940a). Critical works published after 1950 are those by Porsild (Porsild 1955, 1957, 1964, Porsild and Cody 1980), most of which are regularly consulted today, and Aiken et al. (2007), the most recent comprehensive survey of the area. Gillespie et al. (2015) reported additions to the flora of the Canadian Arctic Archipelago, increasing the number of species and infraspecific taxa currently recorded from the region to 375. A recent synthesis of the taxonomy of the global Arctic flora has advanced our understanding of the Canadian Arctic flora in an international context (Elven et al. 2011).

In contrast to the flora of an area, which is based on presence or absence of species regardless of abundance, vegetation refers to assemblage(s) of plant species, often focused on or characterized by the subset of species that are dominant in ecological communities. Arctic vegetation is responding rapidly to the changing Arctic climate, which is warming at twice the rate of the rest of the planet (Anisimov et al. 2007). Elmendorf et al. (2012) identified several biome-wide trends in a global plot-based study of tundra vegetation response to warming, including increased canopy height of vascular plants and increased litter abundance. Shrubification, an increase in shrub biomass, cover and abundance of woody species in response to climate change, has been documented widely across the Arctic (Myers-Smith et al. 2011, Tremblay et al. 2012, Gerald et al. 2013) and decreases in species richness of vascular plants and lichens are associated with it in field studies (Pajunen et al. 2011, Fraser et al. 2014). Species distribution models also predict a decrease in species diversity with shrubification (Mod and Luoto 2016). Shrubification is thought to be responsible, at least in part, for Arctic greening, an increase in biomass and productivity in Arctic ecosystems that has been measured by satellite-derived Normalised Difference Vegetation Index (NDVI) (Pouliot et al. 2009, Bhatt et al. 2010, Gerald et al. 2013). Arctic browning (a decrease in greenness) (Epstein et al. 2015, Phoenix and Bjerke 2016, Lara et al. 2018) has also been documented in some Arctic areas, attributed to such phenomena as thermokarst development, tundra fire, anomalous weather such as extreme winter warming, and insect and fungal pathogen outbreaks (Phoenix and Bjerke 2016). Flowering times and seed dispersal of vascular plants in the Canadian Arctic are correlated with summer temperature, with considerable variation among taxa and geographical areas (Panchen and Gorelick 2017); a changing climate is likely to affect these critical stages of vascular plant life history. As Arctic vegetation changes over the next century as temperatures increase, the composition of the Arctic flora, including the subset of species that is a minor or insignificant component of Arctic vegetation, is likely to also change.

Many regions of the Canadian Arctic Archipelago remain underexplored or unexplored botanically, given the massive size of the region, the short window of opportunity for making field collections during Arctic summer, the small number of taxonomically trained and oriented Arctic botanists conducting field research, and the great logistical challenges and costs associated with accessing remote Arctic areas (Mallory et al. 2018). Given the rapidly changing Arctic climate, there is now increased urgency in advancing taxonomy-focused, specimen-based documentation of the current diversity and distribution of the Canadian Arctic vascular flora, in order to establish a baseline to which future changes in species- and infraspecies-level diversity and distribution can be compared. Such floristic data is foundational to many fields of study, including biogeography, taxonomy, terrestrial ecology, conservation, invasive species biology, and it contributes to basic understanding of Canada’s natural heritage. Specimen-based documentation–meaning that every report of a species is supported by a voucher specimen and associated collection data archived in a publically accessible and permanent collection (i.e., herbarium)–ensures that preserved material, which is part of the permanent scientific record, can be consulted, used and cited indefinitely, as necessary, by future researchers. Studying previously-collected material is a standard part of taxonomic practice, allowing workers to confirm or revise earlier identifications in light of misidentifications, which are common in herbaria (Goodwin et al. 2015), and changing taxonomic concepts through time, which are also common. The latter is exemplified by the “consensus” taxonomy proposed (except in a few cases in which a consensus was not achieved among authors from different geographical regions) for the circumpolar Arctic flora by Elven et al. (2011), which includes many taxonomic concepts that conflict with one or more regionally-accepted approaches to classification.

Here, we report the results of a collections-based floristic study of Victoria Island in the western Canadian Arctic Archipelago. Our study synthesizes existing published and unpublished information on the flora of the island, including new results from five field seasons (2008, 2010, 2013, 2014 and 2016) of botanical collecting at sites across the island. A small subset of collections from our 2008 and 2010 trips, representing first records of the following species for Victoria Island (or first records with confirmed vouchers), were reported in Gillespie et al. (2015): *Carex bicolor*, *Eriophorum brachyantherum*, *Luzula wahlenbergii*, *Corallorhiza trifida*, *Puccinellia banksiensis*, *Stuckenia vaginata*, *Suaeda calceoliformis*, *Arenaria humifusa*, *Arenaria longipedunculata*, *Sabulina stricta*, *Andromeda polifolia*, Oxytropis deflexa var. foliolosa, *Pinguicula vulgaris* and *Salix arctophila*. We present an annotated checklist of the vascular flora of Victoria Island in which we summarize the history of documentation of the flora and cite all specimens including new reports of first records for the island and many new records of species at sites across the island. We also provide taxonomic keys to identify all taxa currently recorded on the island, distribution maps for all taxa on the island, and photographs of many taxa. This work will serve as a new baseline on which continued floristic exploration of Victoria Island can build.

## Victoria Island

**Figure 1.**
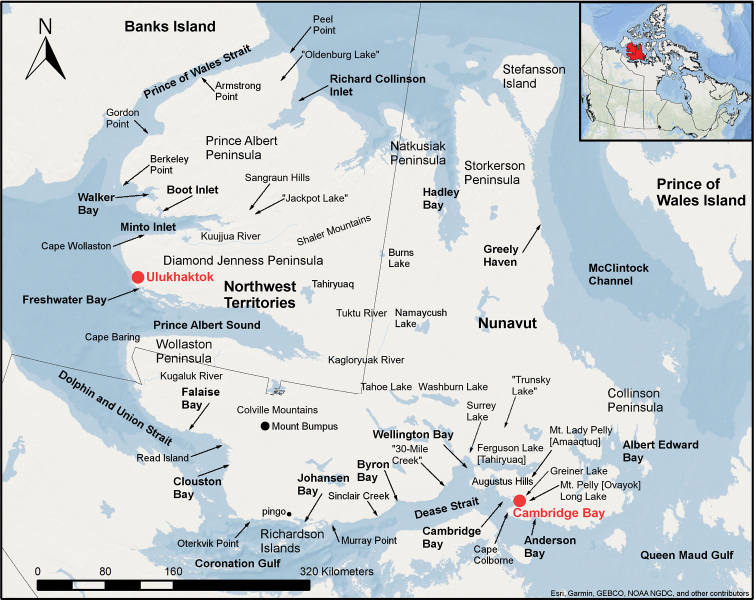
Map of Victoria Island showing locations of named places referred to in the current study. Inset: map of Canada showing the location of Victoria Island in the western Canadian Arctic Archipelago.

Victoria Island (217, 291 km^2^), about 3.8% larger than Great Britain, is the eighth largest island in the world and the second largest (after Baffin Island, 507,451 km^2^) in Canada. It is located in the western Canadian Arctic Archipelago (Fig. 1), bordered by Banks Island to the northwest, from which it is divided by Prince of Wales Straight; the mainland of Nunavut to the south, divided by Dolphin and Union Strait, Coronation Gulf, Dease Strait and the western portion of Queen Maud Gulf; King William Island to the southeast, separated by Victoria Straight; Boothia Peninsula and Prince of Wales Island to the east and northeast, respectively, separated by McClintock Channel; and Melville Island to the north, separated by Parry Channel. Wollaston Peninsula on the southwest part of the island, the southeast part of the island, and Prince Albert Peninsula on the northwest part of the island, were initially given, by Europeans, the English names Wollaston Land (Franklin and Richardson 1828), Victoria Land (Simpson 1843) and Prince Albert Land (Belcher 1855), named for English chemist and physicist William Hyde Wollaston, Queen Victoria and Prince Albert, respectively. John Rae, in 1851, confirmed that Wollaston and Victoria lands were connected (Rae 1852a, b).

Victoria Island was part of British Arctic territory from the 16^th^ century to 1880, the year the British Arctic Territories were claimed by Canada and became part of the Northwest Territories. It remained wholly part of that territory until creation of the territory of Nunavut in 1999. Victoria Island now spans the two territories. The territorial border corresponds to the boundary of the Inuvialuit Settlement Region, which includes much of the western third of the island. The Northwest Territories portion of the island comprises the northern half of the western tip of Wollaston Peninsula, land north of the 70^th^ parallel north except for a southern protrusion around most of Quunnqug Lake, and land east of the 110^th^ meridian west. The Northwest Territories portion of the island is part of the Inuvik Region, and the Nunavut portion is part of the Kitikmeot Region. Victoria Island has been inhabited since approximately 4500 BP (Savelle and Dyke 2002). Today, people live in the communities of Ulukhaktok (formerly Holman), located at the end of the Diamond Jenness Peninsula on the north side of the entrance to Prince Albert Sound on the west coast of the island, and Cambridge Bay (Iqaluktuutiaq), located on the southeast coast of the island along a bay of the same name.

## Ecology

Victoria Island is fully within the Canadian Arctic ecozone, and of the three major Arctic regions recognized in Canada, it is part of the Northern Arctic, as is most of the Canadian Arctic Archipelago (Ecological Stratification Working Group 1995). In the global Köppen–Geiger climate classification, Victoria Island is part of the ET (polar tundra) group (Kottek et al. 2006). For the Arctic area including Victoria Island, climate model projections predict that, by 2071–2100, mean air temperatures could increase by up to 8 °C and the K-G classification of different parts of the island could shift to Semi-arid and subarctic climates (Beck et al. 2018). Such climate changes would likely be accompanied by a major shift in vascular plant species composition, reflecting the milder conditions. Vegetation studies have been conducted at a broad scale across the western half of the island (Edlund 1983) and at a fine a scale in the Wellington Bay area of southeastern Victoria Island (Schaefer and Messier 1994, Schaefer and Messier 1995). Peros and Gajewski (2008) reconstructed vegetation change during the Holocene using sediment-derived pollen data from a lake in the Kuujjua River area of the island, and found that herbaceous plants increased and woody plants decreased through the Holocene in response to long-term cooling; their pollen dataset also records a 0.5 °C increase in temperature over the last century.

**Figure 2.**
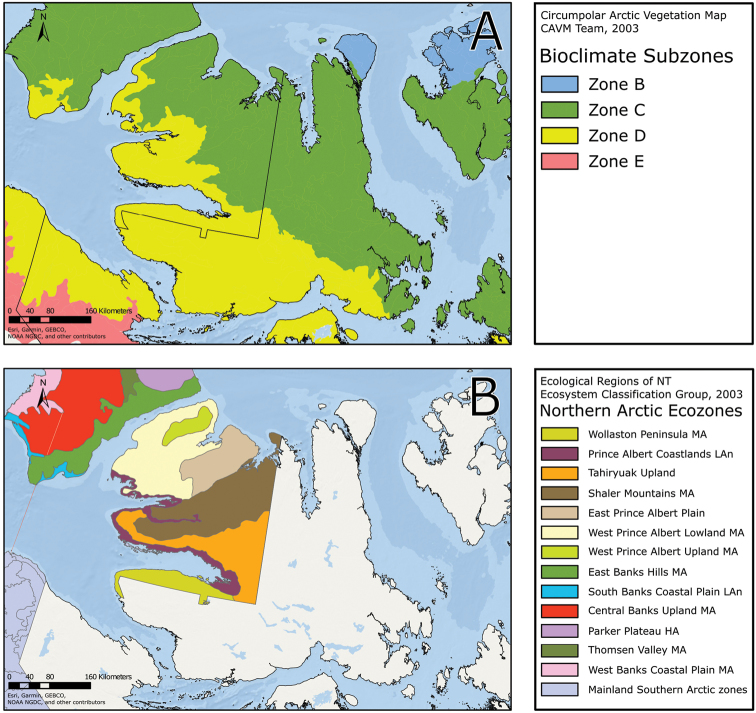
Ecological zones recognized on Victoria Island. **A** Locations of bioclimate subzones C and D according to the Circumpolar Arctic Vegetation Map (CAVM Team 2003) **B** ecological regions recognized in the Northwest Territories part of Victoria Island by the Ecosystem Classification Group (2013). MA = Mid-Arctic. LAn = Low Arctic-north.

The Circumpolar Arctic Vegetation Map–an international effort to develop a unified terminology for describing global Arctic vegetation–divides the circumpolar Arctic into five bioclimate zones (CAVM Team 2003, Walker et al. 2005). The zones are named A to E from north to south, with A, restricted in Canada to the northwestern Queen Elizabeth Islands, being the coldest and harshest, with a mean July temperature of 0–3 °C, <5% cover of vascular plants, vascular plant growth low to the ground (barely exceeding the height of mosses, woody plants absent), and less than 50 species in local floras, and E, restricted in Canada to the mainland, being the warmest and least harsh, with a mean July temperature of 9–12 °C, 80–100% cover of vascular plants, a herbaceous/dwarf-shrub layer 20–50(–80 cm) tall, and 200 to 500 species in local floras. Victoria Island spans bioclimate subzones D and C, reflecting the climatic gradient across the island (Fig. 2). Subzone C encompasses the southern and western portion of the island, with the boundary running from the southeast to northwest from approximately Macready Pt. to northwest of Hay Pt. Subzone C has a mean July temperature of 5–7 °C, a summer warmth index (i.e., sum of mean monthly temperatures greater than 0 °C) of 9–12, 5–50% cover of vascular plants, herbaceous layer 5–10 cm tall, prostrate and hemiprostrate dwarf shrubs less than 15 cm tall, and 75–150 species in local floras. Subzone D has a mean temperature of 7–9 °C, a summer warmth index of 12–20, 50–80% cover of vascular plants, herbaceous and dwarf shrub layers 10–40 cm tall, and 125–250 species in local floras. The Canadian High Arctic Research Station (CHARS) is currently leading a bioclimatic mapping of Victoria Island project. The goal of the project is to refine and better characterize previously determined boundaries between subzones C and D on the island based on results of fieldwork and aerial surveys.

The two communities on Victoria Island are located within bioclimate subzone D. Cambridge Bay has a mean annual air temperature of -13.9 °C for the climate normal period of 1981–2010 and the mean annual temperature in July is 8.9 °C and in February is -32.5 °C for the same period (Environment Canada 2019a). Mean annual air temperatures in Cambridge Bay have increased by an average of 0.6 °C per decade over the 30 year period 1986–2015 with most warming occurring in fall and winter (BGC Engineering Inc. 2016). Average rainfall in Cambridge Bay is 72.1 mm, with the greatest average rainfall in July and August (23.9 mm in each). Average snowfall in Cambridge Bay is 80.2 cm, with the greatest average snowfall in October (15.9 cm). Ulukhaktok has a mean annual air temperature of -11.6 °C for the climate normal period of 1981–2010 and the mean annual temperature in July is 9.0 °C and in February is -28.8 °C (Environment Canada 2019b). Average rainfall in Ulukhaktok is 74.2 mm, with greatest average rainfall in July (22.2 mm) and August (30.2 mm). Average snowfall is 83.3 cm, with greatest average snowfall in October (16.9 cm).

The Northwest Territories Ecosystem Classification Group (2013) produced a detailed, multilevel ecosystem classification for the territory, including the Northwest Territories part of Victoria Island. The Level 1 (Tundra) and Level 2 (Northern Arctic) ecoregions of which Victoria Island is part are broad regional landscapes.They recognized two Level III ecoregions, which are interpretations of regional climates, on Victoria Island: Northern Arctic Mid-Arctic Ecoregion and Northern Arctic Low Arctic-north Ecoregion. Within the Northern Arctic Mid-Arctic Ecoregion they identified six Level IV ecoregions (Fig. 2), which are characterized by bedrock geology, landform, hydrology and vegetation: (1) the West Prince Albert Lowland Mid-Arctic Ecoregion, encompassing most of the Prince Albert Peninsula, composed of dry, calcareous, undulating to hummocky till and glaciofluvial deposits with sparse tundra cover on uplands and sedge tundra on seepage slopes; (2) the West Prince Albert Upland Mid-Arctic Ecoregion, in the middle of the northwestern arm of the Prince Albert Peninsula, comprising a low, discontinuous ridge with gravelly to clayey tills and variable tundra cover; (3) the East Prince Albert Plain Mid-Arctic Ecoregion, on the east side of Richard Collinson Inlet, a primarily frost-shattered calcareous bedrock plain with bouldery till and sparse to absent tundra cover; (4) the Shaler Mountains Mid-Arctic Ecoregion, extending northeast from Diamond Jenness Peninsula across the centre of Victoria Island to the tip of Natkusiak Peninsula, composed of deeply eroded bedrock ridges, plateaus and undulating stony till plains, with upland areas mostly sparsely vegetated and continuous tundra communities common on lower slopes and lowlands; (5) the Tahiryuak Upland Mid-Arctic Ecoregion, east of Prince Albert Sound and along the south side and the west end of Diamond Jenness Peninsula, with a high lake density between approximately 100–300 m above sea level, composed of dry, undulating to hummocky stony tills, marine silts and clays, and bedrock outcrops, with tundra best developed in moist depressions and on seepage slopes; (6) the Wollaston Peninsula Mid-Arctic Ecoregion, including that part of the Wollaston Peninsula that is part of the Northwest Territories, composed of a central level to hummocky till landscape with variable tundra cover surrounded by a thin belt of ancient and mostly unvegetated gravel beaches. Within the Northern Arctic Low Arctic-north Ecoregion they recognized the Prince Albert Coastlands Low Arctic-north Ecoregion, restricted to a narrow zone of coastal areas and protected valleys extending from the head of Prince Albert Sound, along the south side of Diamond Jenness Peninsula, around Minto Inlet to the north side of Walker Bay, and inland along the Kuujjua River. It is composed of fluvial and marine coastal plains, steep rock-walled inlets, plateau remnants and hummocky to undulating till, with pockets of vigorous shrub tundra in deep sheltered valleys. There is no comparable ecosystem classification available for the Nunavut part of Victoria Island.

With the establishment of the Canadian High Arctic Research Station (CHARS; Polar Knowledge Canada) in Cambridge Bay in 2015, new ecological research has been initiated on Victoria Island and the adjacent mainland, including long-term, experiment-based monitoring of the terrestrial ecosystem (McLennan 2017). Victoria Island is part of the CHARS Experimental and Reference Area (ERA), a large study area centred around the CHARS campus in Cambridge Bay, including island and mainland areas and the communities of Ulukhaktok, Kugluktuk, Cambridge Bay, Gjoa Haven, Taloyoak and Kugaaruk (POLAR Knowledge Canada 2019). The Canadian Arctic-Subarctic Biogeoclimatic Ecosystem Classification (CASBEC) initiative aims to develop a standardized approach to classifying, naming, and interpreting Arctic and Subarctic terrestrial ecological communities at a range of scales, based on plant associations (McLennan et al. 2018, Ponomarenko et al. 2019). Floristic and taxonomic data, such as that presented here for Victoria Island, underpins this type of research, and we expect that new data and specimens obtained during future ecosystem classification and related research on Victoria Island will contribute to advancing floristic knowledge of the island.

## Geology

Detailed accounts of the glaciation, geomorphology and surficial geology of Victoria Island are given by Washburn (1947), Thorsteinsson and Tozer (1962) and Fyles (1963), from which the following description is summarized. Most of Victoria Island is underlain by dolomite, with some limestone, sandstone and shale, originating in the early Paleozoic. Substrates across the island are primarily calcareous. Precambrian rocks form a belt of rugged terrain–the Shaler Mountains–comprising sandstone, shale, siltstone limestone, dolomite and gypsum overlain by basaltic lava and agglomerate, located on north-central Victoria Island, trending north-easterly from Diamond Jenness Peninsula to Hadley Bay, encompassing much of Diamond Jenness Peninsula, and reaching an altitude of 655 m, the highest point on the island. Isolated outcrops of Precambrian sandstone and gabbro are also exposed on the Richardson Islands on the south side of the island and in the adjacent Johansen Bay area and from Wellington Bay north to Washburn Lake; for a map, see de Kemp et al. (2006). Granitic outcrops occupying a small area are recorded on the west side of Hadley Bay.

Victoria Island was overlain by the Laurentide ice sheet during the Wisconsin glaciation, and the post-glacial landscape of the lowlands is characterized by numerous glacial landforms, including drumlins, drumlinoid ridges, till plain, moraines, esker complexes, kame hills, abandoned river valleys and meltwater channels, and glacio-lacustrine deposits (clay and silts). Marine strand lines marking former post-glacial marine limits are conspicuous on the island; nearly half the island was, at one point, submerged. Other marine features in inland areas include marine shells, raised deltas, raised beach ridges, abandoned strand lines and marine sediments. Coastal parts of western Victoria Island are characterized by having large morainal belts that often reach heights of 30 m or higher. Morainal topography is particularly varied on Wollaston Peninsula, attaining maximum height at the summit of Mt. Bumpus. Morainal belts of eastern Victoria Island are much smaller. The island is further characterized by its abundant lakes and rivers, floodplain deposits, alluvial fans and deltas. Pingos are present on southeastern Victoria Island, particularly on Wollaston Peninsula.

## Ovayok Territorial Park

**Figure 3.**
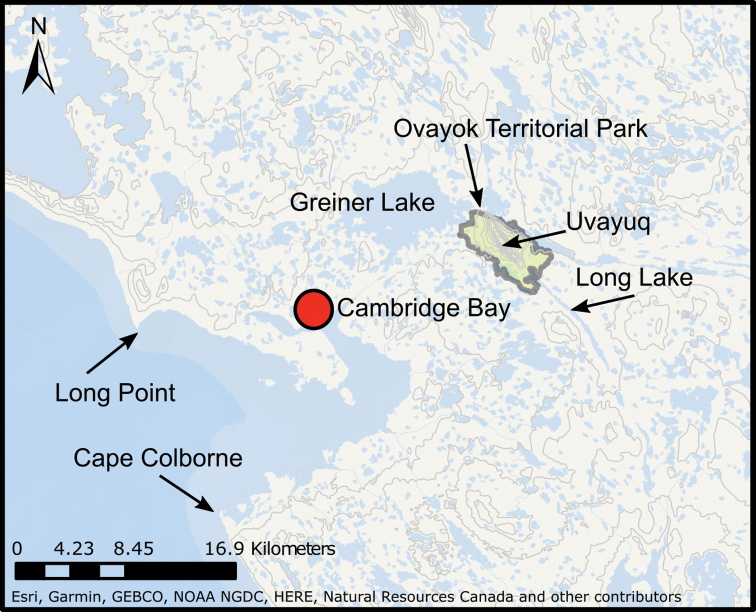
Location of Ovayok Territorial Park in the vicinity of Cambridge Bay on Victoria Island, Nunavut.

Ovayok Territorial Park, the only protected area on Victoria Island and one of three territorial parks in the Kitikmeot Region of Nunavut, is located 15 km east of the community of Cambridge Bay (Fig. 3). The park is centered on Uvayuq (formerly Ovayuk/Mount Pelly), an esker reaching an altitude of more than 200 m above sea level. Uvayuq is the largest of three eskers in the area; the others are Amaaqtuq (“Baby Pelly”) and Uvayuruhiq (formerly Mount Lady Pelly), located northwest of the park between Greiner (Iqaluktuuttiaq) and Ferguson (Tahiryuaq) lakes. The park covers approximately 16 km^2^. Uvayuq was given its English name, Mount Pelly, by Thomas Simpson and Peter Warren Dease in 1839, honouring then-governor of the Hudson’s Bay Company, John Henry Pelly (Simpson 1843). Up to date botanical information for vascular plant diversity is available for few Arctic territorial (Saarela et al. 2017b) and national parks (Saarela et al. 2013). Botanical diversity has not been reported for Ovayok Territorial Park, although the area has been a popular place for collecting, given its prominence on the landscape in the Cambridge Bay area.

## Previous botanical collecting on Victoria Island

### 19^th^ century collections

The earliest botanical collections on Victoria Island were gathered during a four-year (1850–1854), two-ship (*Enterprise* and *Investigator*), British expedition in search of Sir John Franklin’s missing ships *Erebus* and *Terror*, and by John Rae in 1851. These collections are housed at the Royal Botanic Gardens Kew (K; herbarium acronyms follow Thiers (continuously updated)) and are summarized in Simmons’ (1913) account of the early history of botanical exploration of the Canadian Arctic islands. Travelling aboard *Investigator*, commanded by Robert McClure, Moravian missionary and interpreter Johan A. Miertsching made five collections along Prince Albert Sound (*Draba hirta* L. [=*D. glabella*], *Saxifraga oppositifolia*, *Potentilla rubricaulis*, *Astragalus aboriginorum* Richardson [=*A. richardsonii*], *Arctostaphylos alpina* [=*Arctous alpina*]), and a collection of *Salix arctica*, taken at “Mount Adventure” [Adventure Mountain] on Prince Albert Peninsula, is attributed to McClure (Simmons 1913). *Enterprise*, commanded by Richard Collinson, was separated from *Investigator* in the Pacific, but eventually reached the western Arctic. The surgeon, Charles Anderson, who was among the crew on *Enterprise*, made botanical collections at Minto Inlet and Cambridge Bay, representing 94 species according to Simmons (1913) (Suppl. material 1). A collection of *Oxyria digyna* from Walker Bay, taken by Collinson, is also recorded by Simmons (1913).

John Rae made collections along the “south shore” of Victoria Island in 1851 while exploring and mapping the area (Rae and McGoogan 2012). He travelled to the island from the mainland twice that year. On 5 May, Rae crossed, by sledge, the frozen Dolphin and Union Straight and reached Douglas Island (68°28'N, 113°29'W), just west of Wollaston Land, the next day. Rae then travelled eastwards along the southern shore of Victoria Island as far as Wilbank Bay (68°38'N, 110°10'W). Upon his return to Douglas Island, on 15 May, he travelled northwards along the west coast of Wollaston Land to ca. 14 miles beyond Cape Baring (70°02'17"N, 117°21'33"W), before turning around. He returned to the mainland on 2 June. On the second leg of the 1851 expedition, Rae travelled by boat. He left the mouth of the Coppermine River in early July and travelled eastwards along the south shore of Coronation Gulf to the east end of Kent Peninsula. On 27 July he crossed the strait to Victoria Island, reaching Cambridge Bay. Leaving the bay, Rae followed the coast eastwards to Albert Edward Bay, and then travelled north overland on foot to a point ca. seven miles south of Pelly Point (70°12'N, 101°01'W) on the Collinson Peninsula, before turning around. On the return trip Rae apparently followed the southern coast of Victoria Island, as he describes stopping north-east of Cape Peel (69°02'30"N, 107°16'15"W) along Dease Strait and Point Ross [Ross Point; 68°31'N, 111°10'0"W) at Johansen Bay, which was reached on 28 August 1851. We are not aware of information as to precise localities of Rae’s botanical collections; there is no mention of them in his account of his travels on Victoria Island (Rae and McGoogan 2012). Given that his travels on the southwestern portion of the island occurred in May, when there would have been snow on the ground and conditions would not have been suitable for collecting plants, we assume that his collections were gathered on the “south shore” of the southeastern part of the island during July and August. Hooker (1856) listed Rae’s 1851 collections from Victoria Island and from the adjacent mainland, but he did not indicate in the list which specimens were from the island. Simmons (1913), having reviewed the collections at K, reported that Rae made 34 collections on Victoria Island (Suppl. material 1). These collections are also summarized in Porsild (1955).

### 20^th^ century collections

A substantial amount of information on the flora of Victoria Island accumulated in the 20^th^ century. The herbaria in which the collections described below are housed are listed in the Annotated Checklist, unless otherwise indicated. Diamond Jenness, anthropologist, made collections on “Wollaston Land” (Wollaston Peninsula) in 1915, during the Canadian Arctic Expedition, 1913–1918. These were reported in Macoun and Holm (1921). A list of 41 plant collections made by Jenness on the island was also included in Jenness (1991).

Considerable plant exploration of Victoria Island occurred in the 1940s, when many plant collections were made at fur trading posts on the eastern shore of Walker Bay (Fort Collinson), at Holman and Cambridge Bay, and on Read Island just off the south coast of the Wollaston Peninsula. Father Arthème Dutilly, associated with the Catholic University of America, collected in 1940 at Holman, at a site referred to on his collection labels as “Willows Patch” [Boot Inlet; see comments under *Salix alaxensis* about the location of this site], at Kookyoak River [=Kuujjua R.] and at Cambridge Bay (Louis-Marie 1961). Dutilly’s collections are distributed widely in herbaria in Canada, the United States and Europe (Boivin 1983). Although collected over 70 years ago, most of Dutilly’s Victoria Island collections were not included in the distribution maps in Arctic floristic treatments that include the island (Porsild 1955, 1957, 1964, Porsild and Cody 1980, Aiken et al. 2007). The geologist Albert L. Washburn made collections at Cambridge Bay in 1940. In 1941 and 1942, Lillian Ross, spouse of trading post manager Ray Ross, collected at Holman Island post, Read Island post and sites on southern Victoria Island adjacent to Read Island. In 1947, the geologist Y.O. Fortier made collections at Greely Haven (1 August) and Cambridge Bay (3 August). Also in 1947, Nicholas Polunin, Oxford University, made collections at Cambridge Bay (24 August); these collections have not previously been accessible for research, being part of the unprocessed backlog of the National Herbarium of Canada (CAN). In 1948, John L. (Pete) Jenness, son of Diamond Jenness, made a few collections in the vicinity of Richard Collinson Inlet and on the Storkerson Peninsula.

Botanist A. Erling Porsild, with the National Museum of Canada, collected on Victoria Island in 1949 at several sites, spending no more than two days at each: Read Island (27 July), the interior of the Wollaston Peninsula (27 July), the head of Prince Albert Sound (4 August), Holman Island (Ulukhaktok; 8 August), “Jackpot Lake” (an unnamed lake east of the head of Minto Inlet; 16 August), Walker Bay (at Fort Collinson; 25 August), the head of Minto Inlet (2–3 August), Cambridge Bay (5 August), an unnamed lake ca. 60 miles north of Cambridge Bay (6 August), Tahoe Lake and Washburn Lake (4 August), and a few other places inland. Those collections reportedly doubled the number of species known from the island, bringing the then-known flora to 201 species (Porsild 1950a). The collections were reported in Porsild (1955), a monograph on vascular plants of the Western Canadian Arctic Archipelago, including Axel Heiberg, Amund Ringnes, Ellef Ringnes, Borden, Mackenzie King, Cornwallis, Bathurst, Prince Patrick, Melville, Banks, Victoria, Prince of Wales, and King William islands, and numerous smaller islands. Porsild returned to Cambridge Bay in 1959 and again collected there (12 August).

Margaret Oldenburg, an amateur botanist from Grand Marais, Minnesota, travelled and botanized extensively across Victoria Island in the 1940s and 1950s. She travelled primarily by chartered aircraft, typically collecting during brief stops. In 1943, she made collections at Holman Island (=Ulukhaktok; 1 August) and Read Island (23–24, 31 August, 2–3 September). In 1944, she collected at Cambridge Bay (14–15 August) and again at Read Island (20 August). In 1945, she collected at Ulukhaktok (12 August), Walker Bay (12 August) and “Oldenburg Lake” (11 August), an unnamed lake on northwestern Victoria Island west of Peel Point. We determined the location of “Oldenburg Lake” (73.11750, -114.58) based on information in Whyard (1984). In 1946, she collected on the north side of Prince Albert Sound (19 August). In 1954, she collected at “Char Lake” (15 August), which is a small lake southwest of the head of Richard Collinson Inlet on the Prince Albert Peninsula, “Yellow Lake” (8–9 August), which is an inland site east of Deans Dundas Bay, and on a pingo on northwestern Wollaston Peninsula (16 August). We determined the locations of “Char Lake”, “Yellow Lake” and the pingo based on maps on Oldenburg’s specimen labels, on which the sites are mapped as “Sta. 20” [station 20], “Sta. 10” and “Sta. 21”, respectively. Presence of pingos in the area indicated by Oldenburg is confirmed by Geological Survey of Canada (2017). Despite the importance of Oldenburg’s collections, which are extensive and, in many cases, represent the first (and in some cases only) collections from sites on the island, they were not considered in earlier Arctic floristic works. They were, however, known to Porsild (1955) as being unnamed and inaccessible in the Herbarium of the University of Minnesota (MIN). A subset of Oldenburg’s collections from Victoria Island are housed at GH, MIN and UBC, but the majority were recently “discovered”, along with her collections from other Arctic and non-Arctic sites, in the unprocessed backlog cabinets of the National Herbarium of Canada.

Botanical documentation of the Victoria Island flora continued through the 1950s. Entomologists E. Smith and D.K. Sweatman, Department of Agriculture, Ottawa, collected in 1950 at Holman Island (Ulukhaktok). In 1952, entomologists D.P. Gray and B. Gibbard, also with the Dept. of Agriculture, made collections at Holman Island (Ulukhaktok). In 1959 and 1960, W.D. Stretton, Army Survey Establishment, made collections at Cambridge Bay and at numerous sites in interior Victoria Island, during defence mapping operations. Areas where Stretton collected include the north side and head of Prince Albert Sound, Berkeley Point, Burns Lake, Freshwater Bay, Gordon Point, Kuujjua River, Murray Point, Richard Collinson Inlet, Storkerson Peninsula, Ulukhaktok and the west end of the Diamond Jenness Peninsula. Botanists J.A. Calder, D.B.O. Savile and I. Kukkonen collected at Cambridge Bay on 12 August 1959.

Collections in the 1960s were focused on southeastern Victoria Island and in the 1970s in the Ulukhaktok area. Homer A. “Steve” Stephens (and his associate George M. Sutton) made extensive collections in 1962 in the vicinities of Cambridge Bay and Mount Pelly, in association with research on the White-rumped Sandpiper at Cambridge Bay. Stephens also made collections on Jenny Lind Island immediately southeast of Victoria Island in Queen Maud Gulf (Sutton 1967, Parmelee et al. 1968), which are not considered here. In the same year, Robert Hainault made collections at Mt. Pelly, Mt. Lady Pelly and Ferguson Lake north of Cambridge Bay. Ecologist Josef Svoboda, University of Toronto, made collections at Holman (Ulukhaktok) in 1974. Ecologist Lawrence C. Bliss, University of Alberta, collected there that same year.

In the 1980s, Sylvia A. Edlund, Geological Survey of Canada, made substantial contributions to knowledge of the flora of Victoria Island, associated with research characterizing the relationships between surficial geology and plant communities. She made more than 1800 collections at numerous sites across the island, including many from sites where no other collections have been made. Sites visited and collected by Edlund in 1982 include Armstrong Point, Boot Inlet, Cape Baring, Cape Wollaston, Kugaluk River, Kuujjua River, the head of Minto Inlet, Mt. Bumpus, Murray Point, Natkusiak Peninsula, Peel Point, the north side and head of Prince Albert Sound, Richard Collinson Inlet, Sangraun Hills, Shaler Mountains near Richard Collinson Inlet, Tahiryuaq (a large lake north of the head of Prince Albert Sound), Ulukhaktok and southwestern Wollaston Peninsula (Edlund 1983). In 1983, Edlund collected at Cambridge Bay and the head of Minto Inlet, and in 1986 at Hadley Bay and Storkerson Peninsula on northeastern Victoria Island. Edlund collected in 1987 with salicologist George Argus (National Museum of Canada) at Albert Edward Bay, Anderson Bay, Byron Bay, Cambridge Bay and vicinity (including “Starvation Cove”), Cape Colborne, Collinson Peninsula, Diamond Jenness Peninsula, Ferguson Lake, Greiner Lake, Jonnessee Lake, Namaycush Lake, the head of Prince Albert Sound, Surrey Lake and Washburn Lake. Other sites visited in 1987 (without Argus listed as a collector) include the vicinities of Burns Lake, Cape Peel and Namaycush Lake. Edlund’s vouchers are deposited in the National Herbarium of Canada; most are unicates. None of Edlund’s collections have been published, but many were mapped in Aiken et al. (2007). Some 322 of Edlund’s Victoria Island collections, however, were not included in Aiken et al. (2007), as the material was, at the time, part of the unmounted backlog in the National Herbarium of Canada and inaccessible for research.

Botanical exploration of Victoria Island continued in the 1990s. William T. Dushenko made collections at Byron Bay in 1992, in association with research on environmental contaminants (Dushenko et al. 1996). In 1993, Anne H. Weerstra made collections in the Kagloryuak River valley east of the head of Prince Albert Sound, during field research on the breeding biology of the King Eider. Botanist Lynn J. Gillespie, Canadian Museum of Nature, collected in the vicinity of Cambridge Bay in August 1994, and again with Laurie L. Consaul, Canadian Museum of Nature, in July 1997. In 1999, William A. Gould, International Institute of Tropical Forestry, Puerto Rico, made collections at Cambridge Bay, Mt. Pelly, Hadley Bay and along the Tuktu River on central Victoria Island during fieldwork associated with the Circumpolar Arctic Vegetation Map project (Gould et al. 2003). In the same year, Carolyn L. Parker made collections in the vicinity of Cambridge Bay, and Bente Eriksen, Esther Levesque and C.L. Parker made collections in the vicinity of Falaise Bay on Wollaston Peninsula, as part of the Tundra Northwest Expedition 1999 (Eriksen et al. 2006). In 2003 and 2004, collections were made at Ulukhaktok by Robert Bandringa in association with ethnobotanical work (Bandringa et al. 2010).

Over three decades, ecologist Dietbert Thannheiser, University of Hamburg, Germany, conducted phytosociological research at sites on Victoria Island, including Cambridge Bay (1983, 1984, 1986, 1987, 1998), Holman (Ulukhaktok; 1986, 1973), the head of Minto Inlet (1986), Mt. Pelly (1984), Wellington Bay (1983), Hadley Bay (1986), Surrey Lake, Richardson Islands and Johansen Bay (Thannheiser 1986, Thannheiser et al. 2001). Thannheiser et al. (2001) published a list of species occurrences for each of these regions, including numerous first records for Victoria Island. No vouchers are cited, but the paper indicates that vouchers are in personal herbaria of the authors; most or all are now housed in the herbarium of The Arctic University of Norway​ (TROM) but were not examined here. It is unclear, however, if all, or only a subset of material, is vouchered. Some records reported in that paper were considered in Gillespie et al. (2015), but none were considered in Aiken et al. (2007) nor, to our knowledge, in other taxonomic works.

Despite over a century of exploration and documentation of the vascular flora of Victoria Island, only a small subset of the collection data has been published in detail. Material gathered on the island during the Canadian Arctic Expedition was published by Macoun and Holm (1921). Porsild (1955) included voucher information for Victoria Island collections known to him at the time, including details of the collections he made on the island in 1949. It is relatively straightforward to align records reported by Porsild (1955) to maps published in his later treatments (Porsild 1957, 1964, Porsild and Cody 1980), but for collections that were not cited in Porsild (1955), substantial effort is needed to match dots on distribution maps in floristic or taxonomic treatments with vouchers. None of the critical Arctic treatments that cover Victoria Island (Porsild 1957, 1964, Porsild and Cody 1980, Aiken et al. 2007) include details of the specimens on which the works are based. In many instances, Porsild (1955) noted unvouchered observations of species occurrences made by him in 1949, and he included these site records on his subsequent maps without any indication that they are based on unvouchered reports (Porsild 1957, 1964, Porsild and Cody 1980). Aiken et al. (2007) mapped many thousands of records using digital tools but did not publish the dataset underlying the distribution maps. The large dots on the maps in that treatment each cover ca. 40 km, making it difficult or impossible, in some cases, to align a dot to a particular voucher specimen or area, especially in areas where numerous collections have been made, such as in the Cambridge Bay region.

## Materials and methods

### Field work

2008, 2010 and 2017

In July 2008, our team, comprising L.J. Gillespie, J.M. Saarela, L.L. Consaul and R.D. Bull (Canadian Museum of Nature), explored and collected plants along southern Victoria Island, Nunavut. Research was carried out under Nunavut Research Institute Scientific Research Licence 0401308N-A, Nunavut Wildlife Research Permit No. WL 2008-1039, Nunavut Water Board Permit No. 3BC-AFP0813 and Polar Continental Shelf Program (PCSP) Project Number 515-08. We established three base camps: (1) at a site ca. 13 km north of Oterkvik Point and 10 km north of the coast at Coronation Gulf (68°36'50"N, 112°34'21"W; 3–11 July); (2) 8 km east-northeast of Johansen Bay airstrip along the Nakoyoktok River at its outflow from a large unnamed lake (68°39'25"N, 110°42'30"W; 12–21 July); and (3) Sinclair Creek North Warning System site (abandoned DEW-line site) (68°45'5"N, 109°06'20"W; 22–24 July). In each area, we explored as many habitats as possible, by foot, at each camp, and made collections as we encountered taxa. Our aim was to document all of the vascular plant species in the vicinity of each of our three camps with at least one voucher specimen. We also explored several remote sites accessed by helicopter. Staging from Oterkvik Point, we visited five sites via helicopter on 7 July: two sites along a bay on Coronation Gulf east of Oterkvik Point (68°30'46"N, 112°33'60"W; 68°29'17"N, 112°40'13"W); an esker ca. 21 km north of the coast at Coronation Gulf and 24 km north-northeast of Oterkvik Point (68°41'59"N, 112°26'23"W); a low rocky ridge between two lakes near the previous locality (68°42'48"N, 112°30'08"W); low rocky hills at the coast in the vicinity of Oterkvik Point (68°31'32"N, 111°59'58"W; longitude coordinates recorded on specimen labels for this site as 112°59'58"W are erroneous). Collections made while exploring the Oterkvik Point area on foot and during the five helicopter stops noted above are recorded in the annotated checklist as being from Oterkvik Pt. Staging from Oterkvik Point, we also visited two more distant sites via helicopter on 8 July: the eastern-most slopes of the Colville Mountains (69°32'45"N, 112°41'27"W) and the vicinity of a river flowing into Clouston Bay, 3–4 km from the river mouth (69°02'39"N, 113°25'15"W). These are treated as separate sites in the annotated checklist. Staging from Johansen Bay we visited five sites via helicopter on 20 July: a flat-topped steep-sided hill, 11 km northeast of the Johansen Bay airstrip (68°39'12"N, 110°54'47"W); a pingo 23 km west of the Johansen Bay airstrip (68°26'23"N, 111°40'22"W); the Johansen Bay airstrip (68°35'50"N, 111°6'59"W); the west end of Johansen Bay at the mouth of Mackenzie Creek (68°36'4"N, 111°21'7"W); and Mackenzie Creek, about 1 km from its mouth at the west end of Johansen Bay, along a river canyon above a waterfall and rapids (68°36'28"N, 111°22'10"W). Collections made while exploring the Johansen Bay area on foot and during the five helicopter stops noted above are recorded in the annotated checklist as being from Johansen Bay. Staging from Sinclair Cr., we visited one site via helicopter on 21 July: Murray Point on the west side of Wilbank Bay (68°35'33.5"N, 110°18'24"W). Murray Point is treated as a separate site in the annotated checklist. In total we made 1091 collections (numbers) of vascular plants, one of lichens and 27 of bryophytes.

In July 2010, we conducted fieldwork on northwestern Victoria Island, Northwest Territories, again aiming to document all species present at each site visited. Our team comprised L.J. Gillespie, J.M. Saarela, Jennifer Doubt, R.D. Bull and P.C. Sokoloff (Canadian Museum of Nature). Research was carried out under Aurora Research Institute Licence No. 14733, Inuvialuit Land Administration Licence No. ILA10HN004, and PCSP Project Number 509-10. We established three base camps: (1) at a site ca. 8 km inland from the head of Minto Inlet adjacent to a Geo-Mapping for Energy and Minerals (GEM) program, Natural Resources Canada, camp (71°37'10"N, 115°26'22"W; 7–8, 19–26 July); (2) at a site on the northeast side of a small round unnamed lake (ca. 1 km diameter) ca. 4 km north of Boot Inlet on the north side of Minto Inlet (71°30'35"N, 117°20'35"W; 9–12 July); and (3) at a site on the southeast side of “Fish Lake” on the lower Kuujjua River between two small lakes, on the south side of Minto Inlet (71°12'28"N, 116°22'46"W; 12–18 July). Collections were also made in Ulukhaktok by J.M. Saarela and R.D. Bull (5–6 July; Saarela nos. 1410–1508). Staging from the Kuujjua River camp we visited three sites by helicopter on 17 July: the base of north-facing cliffs 68 km east-northeast of Ulukhaktok (71°2'60"N, 116°9'48"W); the sandy banks of the Kuujjua River south of “Fish Lake”, ca. 17 km southeast of the head of Minto Inlet (71°6'43"N, 116°6'21"W); the shore of the Kuujjua River delta at Minto Inlet (71°15'23"N, 116°49'35"W). Staging from the Minto Inlet camp we visited three sites via helicopter on 25 July: a deep canyon on an escarpment south of the head of Minto Inlet in the vicinity of large waterfall (71°25'30"N, 115°12'2"W), a coastal saline flat along the end of the eastern most inlet (north arm) at the head of Minto Inlet (71°31'7"N, 115°6'30"W), and an esker on a plain 3 km south of the head of Minto Inlet (71°27'10"N, 115°17'3"W). We made 1048 collections (numbers) of vascular plants, one of lichens, five of bryophytes and 14 of fungi. Jennifer Doubt made extensive collections of bryophytes during this expedition, which are not considered here.

In 2017, 20 collections were made in Cambridge Bay by J.M. Saarela (nos. 5296–5301) during the Canada C3 expedition (https://canadac3.ca/en/homepage/).

All 2008, 2010 and 2017 collections were dried in the field in standard plant presses. For each collection we preserved a small sample of leaf tissue in silica gel for future molecular analyses. In most cases, we tagged the plant from which we obtained the sample. These tissue samples are preserved in the National Biodiversity Cryobank of Canada at the Canadian Museum of Nature. These collections were variously determined by L.J. Gillespie, J.M. Saarela and P.C. Sokoloff unless otherwise indicated (Suppl. material 2). Willows were identified by George Argus (CAN). A subset of our *Draba*, *Papaver* and *Potentilla* collections were determined by R. Elven and confirmed by us. The first set of our collections is deposited in the National Herbarium of Canada (CAN), Canadian Museum of Nature. Duplicate specimens have been distributed to the following herbaria, as noted in the specimen citations and Suppl. material 2: University of Alaska Museum of the North (ALA); Aurora Research Institute, Inuvik (indicated in the annotated checklist as “ari”, as the herbarium does not have an official acronym); the University of Alberta Vascular Plant Herbarium (ALTA); Icelandic Institute of Natural History, Akureyri Division (AMNH); the B.A. Bennett Herbarium (BABY), Yukon; the Botanical Museum in Oslo (O); the Missouri Botanical Garden (MO); the Marie-Victorin Herbarium (MT), University of Montreal; the Ayre Herbarium (NFLD), Memorial University of Newfoundland; the herbarium in the Beaty Biodiversity Museum (UBC) Beaty Biodiversity Museum; the United States National Herbarium (US), National Museum of Natural History, Smithsonian Institution; the Intermountain Herbarium (UTC), Utah State University; the Royal British Columbia Museum (V); the University of Manitoba Herbarium (WIN); and the herbarium of the Institut für Systematische Botanik (Z), Universität Zürich.

2013, 2014

Fieldwork was conducted by B.A. Bennett in the vicinity of Cambridge Bay in 2013 and the Cambridge Bay and broader vicinity in 2014. The aim of this work was to document all species of vascular plants in the area to contribute to understanding of the ecology of the greater Canadian High Arctic Research Station (CHARS) (Polar Knowledge Canada) research area. In 2013, collections were made at numerous sites within the community, along the road to Mt. Pelly, within Ovayok Territorial Park, at the east end of Greiner Lake, and along the road west of the community, as far west as the hills above Long Point. In 2014, collections were made within the hamlet, across the bay from the hamlet, north of the DEW line site, at Long Point and at three sites more distant from the hamlet: “30-Mile Creek”, near the Ekalluk River and the west end of Ferguson Lake, and “Trunsky Lake”. Vouchers of material collected in 2014 has been distributed to ALA, BABY, CAN, MO, UBC, V as well as the CHARS herbarium (indicated in the annotated checklist as “chars”, as the herbarium does not have an official acronym) in Cambridge Bay; Stanley L. Welsh Herbarium (BRY), Brigham Young University; National Collection of Vascular Plants (DAO), Agriculture and Agri-Food Canada, Ottawa; Herbarium (MICH), University of Michigan; Michael J. Oldham herbarium, Peterborough, Ontario (indicated in the annotated checklist as “od”, as the herbarium does not have an official acronym); Herbarium (SRP), Boise State University; Herbarium (UAAH), University of Alaska Anchorage; and Herbarium (WTU), University of Washington. We here consider 319 collections made by B. Bennett and associates in 2013 and 2014.

2016, 2018 and 2019

Botanical fieldwork was conducted by CHARS ecologist S. Ponomarenko during three field seasons. From 3–18 August 2016 a vegetation survey was carried out for the CHARS Intensive Monitoring Area (IMA), an area restricted to two small watersheds within the Greiner Lake watershed that drains to the northern shore of Greiner Lake approximately 20–30 km to the northeast of Cambridge Bay, an area measuring about 50 km^2^. This work was conducted as part of the high resolution vegetation mapping of the CHARSIMA project (Ponomarenko et al. 2019).

In the 2018 and 2019 field seasons, floristic collections were obtained during helicopters surveys of a broader area. In 2018, five days of helicopter surveys were undertaken within the Greiner Lake watershed, an area measuring approximately 1,500 km^2^. In 2019, eight days of helicopter surveys were undertaken north and east of Greiner Lake watershed along the border of bioclimatic subzones C and D. This survey covered an area of about 2,000 km^2^ north and east from the Greiner Lake watershed. This work constituted a part of the vegetation inventory and mapping project aiming to assemble baseline data for the CHARSERA.

Vouchers collected during these three years of fieldwork are housed at CAN and CHARS. In total more than 700 specimens were collected, of which 394 collections were reviewed for this work. The remaining 300 collections, mainly collected in 2019, are not yet processed.

Herbarium research and data curation

In addition to accounting for our new collections, we attempted to locate and confirm or revise determinations of all unique collections of vascular plants from Victoria Island. To find specimens collected previously from the study area, we manually searched the collections at CAN, DAO, MT, the University of Calgary (UAC), UBC and Herbier Louis-Marie (QFA), Université Laval, and we queried the Global Biodiversity Information Facility (GBIF), Canadensys (http://data.canadensys.net/explorer/en/search), the Consortium of Pacific Northwest Herbaria (http://www.pnwherbaria.org/about.php), Integrated Digitized Biocollections (iDigBio) portal (https://www.idigbio.org/portal/search), and various institutional online databases. In addition to the herbaria noted above, specimens from Victoria Island cited here are deposited in the R.L. McGregor Herbarium (KANU), University of Kansas; H.A. Stephens Herbarium (KSTC), Emporia State University; TRTE Herbarium (TRTE), University of Toronto Mississauga; Herbier du Québec (QUE), Sainte-Foy, Québec; and Harvard University Herbaria (GH). We also reviewed relevant taxonomic and floristic literature (Porsild 1955, 1957, 1964, Porsild and Cody 1980, Aiken et al. 2007), conducted online searches, and consulted an unpublished database of specimen records that was developed and used in production of the "Flora of the Canadian Arctic Archipelago" (Aiken et al. 2007). We have seen and confirmed at least one duplicate for most of the specimens cited. A subset of material was confirmed based on review of specimen images available online. Determinations for some specimens we have not seen are accepted based on the authority of previous determiners, especially for taxa that are well known and not taxonomically problematic. We have also accepted some non-confirmed records from well-collected sites for which we have reviewed and confirmed numerous other records, such that a possible mis-identification of one of many records of a species from a site would not affect understanding of diversity at the site. With a few exceptions, we did not study the 19^th^ century collections, for which site information is vague.

As part of the current study, three batches of unprocessed material gathered on Victoria Island in the 1940s, 1940s–50s and 1980s that was stored in the backlog collection of the National Herbarium of Canada were organized, identified, mounted and inserted into the permanent collection. One batch comprised 134 sheets collected by Nicholas Polunin at Cambridge Bay in 1947. The second batch comprised 498 sheets collected by Margaret Oldenburg at various sites across the island (see Introduction). The third batch comprised 359 collections gathered by Sylvia Edlund at various sites across the island in 1982, 1986 and 1987 (see Introduction). In the course of processing this material, we discovered that 38 of these Edlund backlog collections from Victoria Island had previously been assigned CAN accession numbers and were recorded in the museum database (and thus mobilized online), but the material remained unmounted, until now. These 991 collections–of which some were gathered 70+ years ago – are now available to the scientific community and are published here. The backlog material from Victoria Island dealt with here is a subset of larger backlog batches of Arctic specimens at CAN gathered by Edlund, Oldenburg and Polunin.

During the course of this study, imaging of all vascular plant material housed at CAN from Yukon, Northwest Territories and Nunavut was completed and all images were linked to the institutional database and mobilized via GBIF. GBIF tools were used to identify and fix putative data errors in Victoria Island records. The images also facilitated completion of data entry for CAN specimens from Victoria Island for which only “skeletal” records existed (i.e., taxon name and higher-level geographic provenance – country and province/territory) or for which data entry was otherwise incomplete.

We amalgamated all collection data obtained from different resources into a spreadsheet. Substantial manual cleaning of the complete dataset was undertaken to make the dataset useable, including standardizing names of collectors, date format and locality descriptions among specimens gathered by the same collector at the same site. We combined records of duplicate specimens housed in different collections into single records, maintaining information on the disposition(s) of the duplicate specimens.

We spent considerable time improving the georeferencing of records in our dataset. Most collections from Victoria Island were made long before the existence of the Global Positioning System and many before the existence of detailed topographical maps, or of maps, period. As such, many collections either lacked coordinates or included the following: (1) inaccurate or erroneous primary coordinates, (2) accurate or inaccurate secondary coordinates but no attribution or source information for the georeferenced coordinates, or (3) accurate but imprecise coordinates determined to the nearest minute, being within ca. one nautical mile of the site, assuming the coordinates are correct. Prior to the advent of digital mapping, this level of precision was generally sufficient for dot-based distribution maps presented at a fixed scale. For example, each dot in the maps in Porsild and Cody (1980) covers about 110 km across (ca. 9,503 km^2^) and in Aiken et al. (2007) about 40 km across (ca. 1,256 km^2^). In digital environments, however, inaccurate coordinates may be problematic and misleading. For many Victoria Island collections, imprecise label coordinates often placed terrestrial collecting sites in freshwater or marine environments or far from descriptions of the places where collections were made, well beyond distances likely to have been travelled by earlier collectors by foot in the limited time that was typically available for collecting at a site.

Therefore, to improve accuracy of geographical coordinates, we secondarily georeferenced many sites following standard point-radius protocols, including determining estimates of coordinate uncertainty in metres, in cases where we were confident that existing location data could be improved upon. Georeferencing was done by J.M. Saarela and Paul Wise, Canadian Museum of Nature. Georeferencing data is included in Suppl. material 2. Where possible, we refined georeferencing data through consultation of published information about the itineraries of past collectors. We also attempted to standardize georeferencing data for collections made in the same area by the same collectors at the same time and by different collectors at different times. At times, this required superseding previous georeferencing for a site. For example, many collections over several decades have been made at “Cambridge Bay”, and that area has been georeferenced differently many times by different workers at different herbaria, including georeferencing the hamlet and the bay, which both have the same name even though it is safe to assume that collections were made on land in the vicinity of the hamlet, not in the bay (a marine environment). The name “Holman Island” on labels has also been the cause of confusion. The hamlet of Ulukhaktok, the Kangiryuarmiutun name for the area, was until 2006 known as Holman and sometimes referred to as Holman Island, as it is on many early herbarium collections. Holman Island is also the name of a small island in the Amundsen Gulf to the east-southeast of Ulukhaktok, after which the community was originally named. Holman Island was named for John R. Holman, a member of the Inglefield Arctic Expedition (1853–54). Although contemporary workers have regularly confused the two places when georeferencing, we are not aware of any botanical collections from the true Holman Island (70°39'6"N, 117°43'33"W), and we have corrected those errors. Collections made at Holman or “vicinity of Holman Post [trading post]” up to 1966 were gathered on the east side of Kings Bay, the site of the community until it moved across the bay to its current location on Queen’s Bay in that year. All collections from the community now known as Ulukhaktok are included under that name.

Distribution maps were generated in ArcMap 10.5.1. Additionally, using ArcMap 10.5.1, we generated maps showing the locations of all collecting sites on the island, maps showing the locations of collections made by S. Edlund, including for specimens previously accessioned into the CAN herbarium and for specimens that had been housed in backlog and were newly accessioned as part of this study, and collections made by A.E. Porsild, Gillespie et al., B. Bennett, S. Ponomarenko, and M. Oldenburg. A heat map showing the density of collections at sites on the island was generated using QGIS 3.4.

Annotated checklist

The vascular flora of Victoria Island is summarised in an annotated checklist. Classification of lycophytes and ferns follows The Pteridophyte Phylogeny Group (2016). Angiosperms are organized according to the linear classification of flowering plants proposed by The Angiosperm Phylogeny Group (2016). Genera are listed alphabetically within families and species are listed alphabetically within genera. Taxonomy at genus, species and infraspecific levels is based on consideration of the relevant global taxonomic literature, including Elven et al. (2011), treatments in the Flora of North America series (Flora of North America Editorial Committee 1993+), and taxonomic monographs and revisions, such as Mosyakin (2016) and Wiegleb et al. (2017). For each species we provide important synonyms, focusing on names used in critical Canadian (Porsild and Cody 1980, Aiken et al. 2007) or international (Elven et al. 2011) Arctic taxonomic treatments, more recent national or continental treatments, particularly the Flora of North America, and other taxonomic works. Common name(s) in English are mostly from the Flora of North America series and Brouillet et al. (2010+). Global distribution summaries are those described by Elven et al. (2011). For each taxon recorded from Victoria Island we include a summary of the broader distribution in the Canadian Arctic, referencing primary and secondary literature, and, in a few case, unpublished specimens. For species that are rare on Victoria Island and known from only one or few collections, we provide information on habitat, when available. Photographs are included for a subset of taxa to facilitate identification and illustrate habitats. We also provide keys to the families, genera, species and infraspecific taxa, to facilitate identification of plants on the island. Keys were adapted from diverse published sources. Measurements in the keys refer to lengths unless otherwise indicated.

All species reported for the study area are documented by one or more voucher specimens, and only vouchered records are included and mapped. Observations of species noted by Porsild (1955), which were mapped in subsequent work (Porsild 1957, 1964, Porsild and Cody 1980, Aiken et al. 2007), are mentioned in the text unless we cite a voucher obtained by a different collector from the same area, confirming the occurrence of the taxon in that area. These comments thus identify occurrences mapped in earlier treatments for which no voucher exists. For each collection, we list the collector(s), collection number or *s.n.* [*sin nombre* = without number] if no collection number exists, and the code(s) identifying the herbaria where the collection is housed. Nineteenth century collections are summarized in Suppl. material 1 and not included in the annotated checklist, except in a few cases. In a few cases, unvouchered observations of rare taxa in the Cambridge Bay are noted, but not mapped; collections should be obtained for proper documentation of these occurrences.

To simplify publication of voucher information, we assigned each collection to a general area of the island. Where possible, we used general areas as described on specimen labels. In cases where no or vague site information is given on labels, however, we assigned specimens to a nearby named place. For example, collections by Edlund reported from south of Burns Lake were gathered in 1982 and 1987 at sites ca. 40–45 km south or south-southwest of the lake, but on the specimen labels the location is described only as “Geological Survey of Canada peat study location” along with the coordinates. In most cases, the toponyms we use are recognized by the Geographical Names Board of Canada. A few sites names, however, are not officially recognized. Examples include “Oldenburg Lake”, “Trunsky Lake” and “30-Mile Creek”, also known locally as “30 Mile River” and “Halovik River”. Locations of all general areas are shown in Fig. 1. The “Cambridge Bay” area includes all collections from the immediate vicinity of the hamlet, along the road to Ovayok Territorial Park (but not including collections made within the park limit), and along and in the vicinity of the road west of the hamlet, including Long Point and the Augustus Hills. Ulukhaktok includes the immediate area of the hamlet. Collections made by S. Ponomarenko recorded from Greiner L. include specimens taken from throughout the Greiner L. watershed, except those that were gathered close to the west side of Albert Edward Bay, which are recorded as being from that general area. The following abbrevations are used in the Annotated Checklist: **B.**, Bay; **C.**, Cape; **Cr.**, Creek; **I.**, Island; **Inl.**, Inlet; **Mts.**, Mountains; **P.**, Peninsula; **Pt.**, Point; **R.**, River; **S.**, Sound; **TP**, Territorial Park.

Some toponyms on Victoria Island have changed in recent years. Ferguson Lake, a large lake north of Cambridge Bay that flows into Wellington Bay via the Ekalluk River, is officially known as Tahiryuaq, the Inuinnaqtun name. We use the English name for the lake to avoid confusion with two other lakes on Victoria Island also officially named Tahiryuaq. Both of these are in Northwest Territories, one north of Prince Albert Sound (70°56'2"N, 112°15'7"W) where collections were made by Edlund and the other southeast of the head of Minto Inlet (71°27'2"N, 114°45'8"W). The esker formerly known as Ovayuk/Mount Pelly, where many collections have been made, was renamed Uvayuq, effective 21 September 2012. The esker northwest of Mount Pelly and north of Cambridge Bay formerly known as Mount Lady Pelly (69°15'28"N, 104°48'37"W), where collections were made in 1962, was renamed Amaaqtuq, effective 21 September 2012.

For each taxon recorded from Victoria Island we summarize previous reports of the species occurrence on the island by site and indicate sites for which a taxon is newly recorded here. “Previously recorded” at a site means a species occurrence was stated or mapped for a site in one or more earlier published works, which are cited in the text here, regardless of whether or not voucher information was published, and the earlier report is supported by one or more vouchers cited in the text here, unless otherwise indicated. In most cases, the vouchers cited here are the collections on which the earlier records were based. In many cases, new material has been gathered from sites at which a species was previously reported, particularly those areas that are best collected, like Cambridge Bay and Ulukhaktok. Although we do not, in most cases, comment on the accumulation of material of a species at a particular site, we do cite all the relevant material known to us from each site.

“Newly recorded” at a site means the current study is the first to publish an occurrence of a species at a particular site. Such new records reported here include collections made during our fieldwork from 2008 on, earlier collections that previous workers did not consider in their floristic treatments, earlier collections that were not processed and available for study until recently, and new identifications of material known to previous authors, either because previous determinations were incorrect or in light of revised taxon circumscriptions. In all cases we include explicit citation of one or more voucher specimens supporting new records.

Thannheiser et al. (2001) reported species from numerous sites across Victoria Island, including many listed as new records for sites. Because we have not reviewed any voucher specimens supporting those reports, nor are we aware of the existence of vouchers for all reports, we do not include these records in our maps. Nevertheless, we recognize the importance of the work done by Thannheiser and colleagues over many years on Victoria Island and note all instances where Thannheiser et al. (2001) report a species from a site for which we have not seen a voucher. In cases where Thannheiser et al. (2001) newly reported a species from an area and we have confirmed and cite a collection made by a different collector in the same area, we state that occurrence of the species in the area as reported by Thannheiser et al. (2001) is confirmed (“conf.”); this does not mean we have confirmed a voucher taken by Thannheiser and colleagues supporting their report of the species. This is the case, for example, for many records newly reported from Johansen Bay by Thannheiser et al. (2001) confirmed to be present in that area by collections we made in that area in 2008.

Patterns of floristic diversity

To characterize patterns of floristic diversity on Victoria Island we scored, based on our dataset, the presence of species/taxa in the following regions: Nunavut and Northwest Territories, the six areas that we aimed to document comprehensively in 2008 and 2010, two additional well-collected areas (Cambridge Bay, Ulukhaktok), and Ovayok Territorial Park, the only protected area on the island (Table 2). The geographical limits of all these areas correspond to the way the sites are listed in the annotated checklist (see above), except we included Murray Point in the Johansen Bay area rather than treating it separately. We determined the area (km^2^) represented by each of these general study areas by drawing a bounding box around all sites in Google Earth (Google 2019) and using the polygon measuring tool: Oterkvik Point: 355 km^2^; Johansen Bay: 212 km^2^; Sinclair Creek, 1 km^2^; Boot Inlet: 8.77 km^2^; the head of Minto Inlet: 128 km^2^; Kuujjua River: 198 km^2^; Cambridge Bay: 200 km^2^; Ulukhaktok: 16.5 km^2^. We constructed species discovery curves for the six 2008 and 2010 areas to characterize the cumulative number of species recorded at each as a function of the time (days) we spent searching for species diversity. We also scored the presence of taxa in the Circumpolar Vegetation Map bioclimate subzones C and D across Victoria Island (CAVM Team 2003), in the Nunavut portion of the island, and the ecological regions in the Northwest Territories portion of the island defined by Ecosystem Classification Group (2013). For the Nunavut portion of the island we additionally scored presence/absence of taxa east and west of Wellington Bay and in two subzones of bioclimate subzone C, one comprising Storkerson Peninsula, the head and west side of Hadley Bay and Natkusiak Peninsula and the other comprising the rest of the subzone across the middle of the island.

## Results

**Figure 4.**
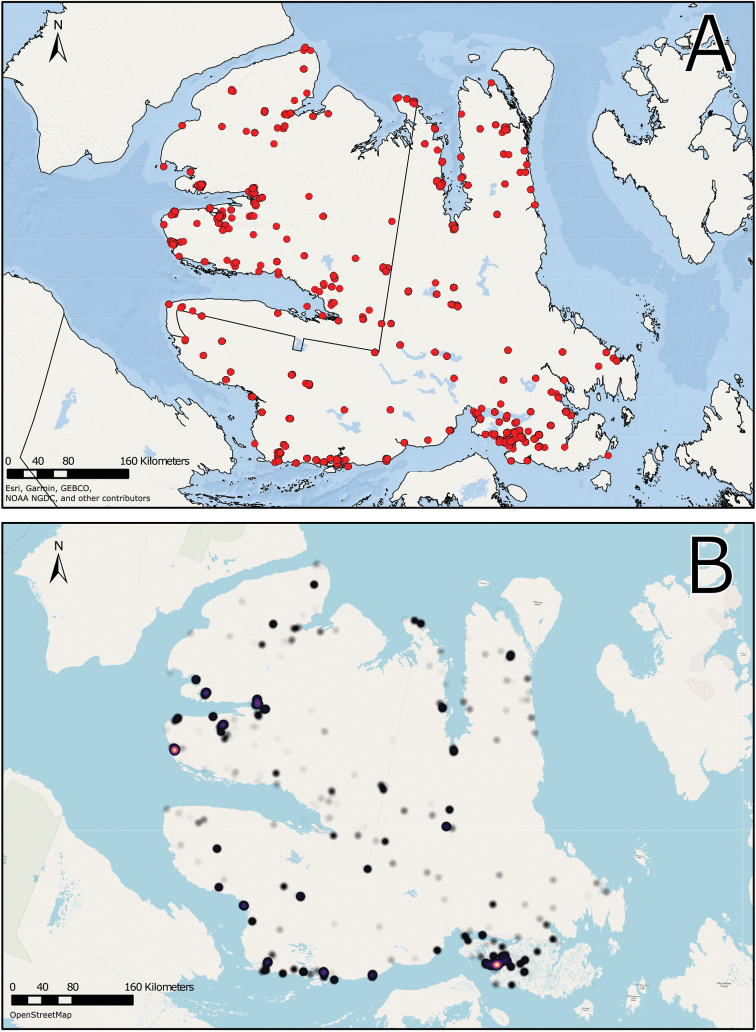
**A** Locations of all collecting sites on Victoria Island **B** heat map showing density of collections at sites on Victoria Island. Lighter colour indicates lesser density of collections and darker colour indicates greater density of collections at a site.

**Figure 5.**
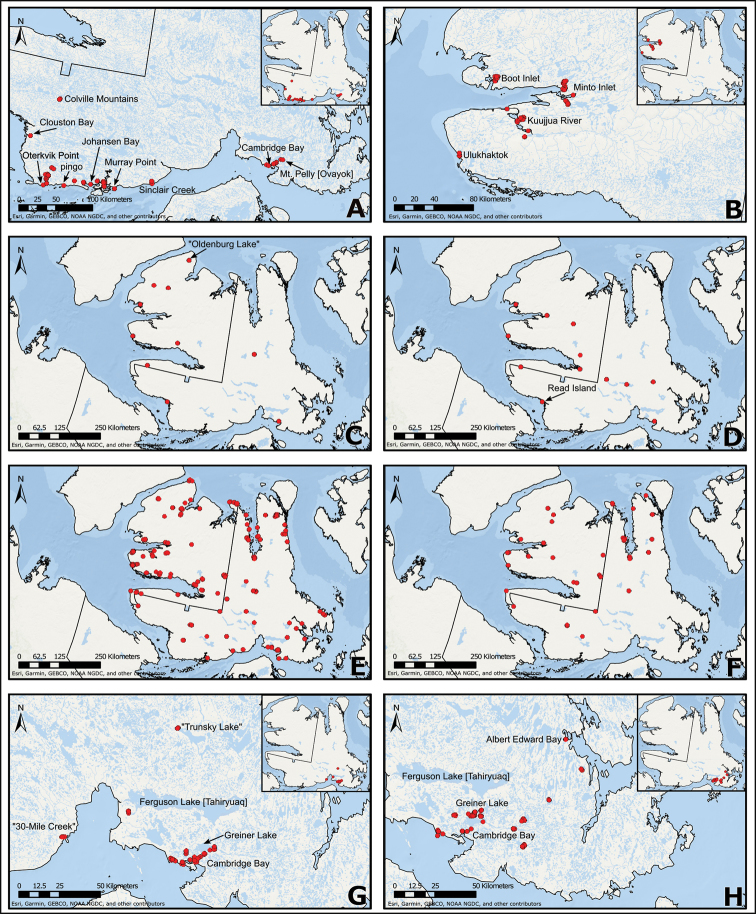
Locations of collecting sites of major collectors on Victoria Island. **A** L.J. Gillespie et al. 2008 **B** L.J. Gillespie et al. 2010 **C** M. Oldenburg **D** A.E. Porsild **E** S.A. Edlund (existing) **F** S.A. Edlund (backlog) **G** B.A. Bennett **H** S. Ponomarenko.

**Figure 6.**
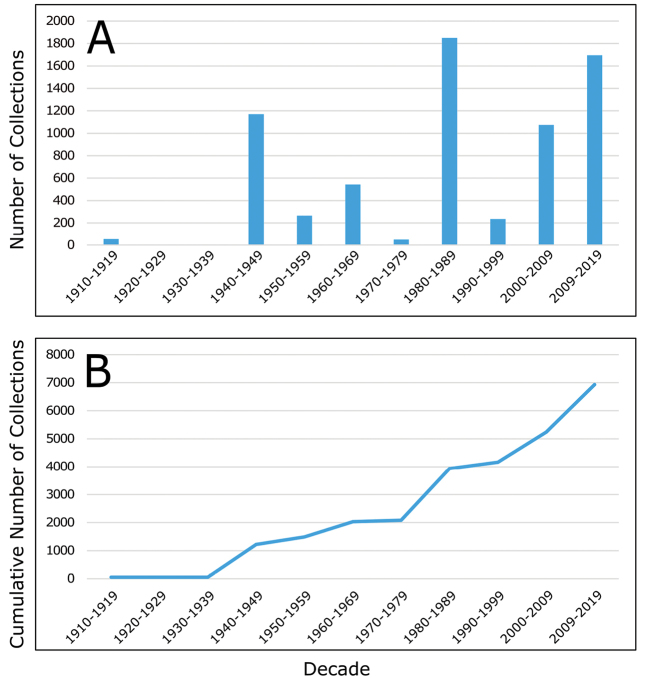
**A** Number of collections on Victoria Island by decade included in the current study, from 1910 to the present **B** cumulative number of collections made on Victoria Island by decade, from 1910 to the present.

We compiled a dataset of some 7031 unique collections of vascular plants from Victoria Island (Suppl. material 2). Although many sites on the island have been visited by vascular plant collectors, there is considerable range in density of collections at particular sites and areas across the island, with few collections at many sites and many collections from only a few sites (Fig. 4). The most densely collected areas, in terms of numbers of unique collections, are Boot Inlet, Cambridge Bay, Johansen Bay, Kuujjua River, the head of Minto Inlet, Oterkvik Point and Ulukhaktok. Locations of collecting sites of major collectors on Victoria Island are shown in Fig. 5. The greatest number of specimens were collected in the 1940s, 1980s, 2000s, and 2010s, ranging in each of those decades from 1074 to 1850 collections (Fig. 6A). There are no collections in our dataset gathered on the island in the 1920s and 1930s, and few collections included here were gathered in the 1910s (54) and 1970s (49) (Fig. 5). The most rapid accumulation of collections on Victoria Island has occurred since 2000 (Fig. 6B).

The vascular flora of Victoria Island comprises 38 families, 108 genera, 272 species, and 17 additional taxa (Table 1). Of the 289 taxa known on the island, 237 are recorded from the Northwest Territories portion of the island and 277 from the Nunavut part. Lycophytes comprise one order, family, genus and species. Monilophytes comprise two orders, four families, four genera and six families. Angiosperms comprise 18 orders, 103 genera, 33 families, 265 species and 17 additional taxa. Monocots comprise three orders, six families, 24 genera, 83 species, and nine infraspecific taxa. Eudicots are represented by fifteen orders, 27 families, 79 genera, 182 species, and eight additional taxa. A complete list of taxa is presented in Table 2 and the Annotated checklist.

**Table 1.** Number of genera and species in each family of vascular plants recorded from Victoria Island. The higher level classification of angiosperms follows The Angiosperm Phylogeny Group (2016).

			Order	Family	Genera	Species/Taxa
**Lycophytes**			Lycopodiales	Lycopodiaceae	1	1
**Monilophytes**			Equisetales	Equisetaceae	1	3
			Polypodiales	Cystopteridaceae	1	1
				Dryopteridaceae	1	1
				Woodsiaceae	1	1
**Monocots**			Alismatales	Potamogetonaceae	1	2
				Tofieldiaceae	1	2
			Asparagales	Orchidaceae	1	1
			Poales	Juncaceae	2	7/8
				Cyperaceae	2	33/35
				Poaceae	17	38/44
**Eudicots**			Ranunculales	Ranunculaceae	6	13
				Papaveraceae	1	4
	Superasterids		Caryophyllales	Plumbaginaceae	1	1
				Polygonaceae	2	2
				Caryophyllaceae	7	19/21
				Amaranthaceae	1	1
				Montiaceae	1	1
	Superasterids	Asterids	Ericales	Primulaceae	2	3
				Ericaceae	8	11
			Gentianales	Rubiaceae	1	1
				Gentianaceae	2	2
			Boraginales	Boraginaceae	1	2
			Lamiales	Plantaginaceae	2	2
				Lentibulariaceae	1	1
				Orobanchaceae	2	8
			Asterales	Asteraceae	13	25/26
	Superrosids		Saxifragales	Saxifragaceae	3	15
				Haloragaceae	1	1
		Rosids	Fabales	Fabaceae	4	10
			Rosales	Rosaceae	3	13/15
				Campanulaceae	1	1
			Fagales	Betulaceae	1	1
			Celastrales	Celastraceae	1	1
			Malpighiales	Salicaceae	1	10/11
				Linaceae	1	1
			Myrtales	Onagraceae	2	2
			Brassicales	Brassicaceae	10	31/33
		**Total**	**21**	**38**	**108**	**272/289**

Three families are represented by more than ten genera: Asteraceae (13), Brassicaceae (10), and Poaceae (17). Of the remaining 35 families, 21 are represented by a single genus, eight by two genera, two by three, one by four, one by five, one by seven and one by eight (Table 1). The only hybrid recorded with a hybrid formula on the island is within the genus *Salix*. Two infraspecific taxa are recorded on the island in 13 species: *Artemisia borealis* (Asteraceae), *Braya glabella*, *B. thorild-wulffii* (Brassicaceae), *Silene involucrata*, *S. uralensis* (Caryophyllaceae), *Carex bigelowii*, *Eriophorum scheuchzeri* (Cyperaceae), *Juncus arcticus* (Juncaceae), *Anthoxanthum monticola*, *Elymus alaskanus*, *Festuca rubra*, *Poa arctica* (Poaceae), *Potentilla arenosa* (Rosaceae). Three infraspecific taxa of *Poa pratensis* are recorded on Victoria Island.

The number of collections per taxon from Victoria Island ranges from 1 to 162 (mean 24 ± 23). Thirty-nine taxa are known on the island from a single collection: *Eurybia sibirica*, *Senecio lugens* (Asteraceae), *Mertensia drummondii* (Boraginaceae), Braya thorild-wulffii subsp. glabrata, *Cardamine bellidifolia*, *Crucihimalaya bursifolia*, *Draba norvegica*, *D. pauciflora*, *Erysimum coarctatum*, *Parrya nudicaulis* (Brassicaceae), *Sabulina elegans*, *S. stricta*, *Sagina caespitosa* (Caryophyllaceae), Eriophorum russeolum subsp. albidum (Cyperaceae), *Andromeda polifolia* (Ericaceae), Oxytropis deflexa var. foliolosa (Fabaceae), *Luzula wahlenbergii* (Juncaceae), *Montia fontana* (Montiaceae), *Corallorhiza trifida* (Orchidaceae), Castilleja pallida var. caudata, *Pedicularis hirsuta* (Orobanchaceae), Anthoxanthum nitens subsp. nitens, *Bromus pumpellianus*, Deschampsia cespitosa subsp. cespitosa, *D. sukatschewii*, Festuca rubra subsp. rubra, *Lolium perenne*, Poa pratensis subsp. pratensis (Poaceae), *Stuckenia filiformis*, *S. vaginata* (Potamogetonaceae), *Pulsatilla nuttalliana* (Ranunculaceae), Potentilla hyparctica subsp. hyparctica, P. × prostrata, *P. vulcanicola*, *Rubus chamaemorus* L. (Rosaceae), *Galium aparine* (Rubiaceae), *Salix arctophila*, S. ovalifolia var. ovalifolia, *S. planifolia* (Salicaceae). Seven taxa are known from two collections: Braya thorild-wulffii subsp. thorild-wulffii, *Draba fladnizensis*, *D. oligosperma* (Brassicaceae), *Arenaria longipedunculata* (Caryophyllaceae), *Equisetum scirpoides* (Equisetaceae), Anthoxanthum monticola subsp. monticola (Poaceae) and *Ranunculus sulphureus* (Ranunculaceae). Three taxa are known from three collections: Antennaria monocephala subsp. angustata, *Artemisia tilesii* (Asteraceae) and *Carex bicolor* (Cyperaceae). Of the remaining taxa, 26 are known from 4–5 collections, 31 from 6–10, 51 from 11–19, 41 from 21–30, 28 from 31–40, 27 from 41–50 and 16 from 51–59. Fifteen species are known from 61–91 collections: *Parrya arctica* (Brassicaceae), *Sabulina rubella*, Silene uralensis subsp. uralensis (Caryophyllaceae), Carex aquatilis subsp. stans, C. fuliginosa subsp. misandra, *C. membranacea*, C. scirpoidea subsp. scirpoidea (Cyperaceae), *Pedicularis lanata* (Orobanchaceae), Arctagrostis latifolia subsp. latifolia, *Dupontia fisheri*, *Festuca baffinensis*, Poa glauca subsp. glauca (Poaceae), *Bistorta vivipara* (Polygonaceae), Dryas integrifolia subsp. integrifolia (Rosaceae) and *Salix richardsonii* (Salicaceae). Three species are known from over 100 collections: *Draba corymbosa* (102), *Salix arctica* (162), *Draba cinerea* (114) and *Stellaria longipes* (113).

Twenty-one taxa in eight families are newly recorded for the flora of Victoria Island, namely *Artemisia tilesii*, *Senecio lugens*, *Taraxacum scopulorum* (Asteraceae), *Crucihimalaya bursifolia*, *Draba fladnizensis*, *D. juvenilis*, *D. pilosa*, *D. simmonsii* (Brassicaceae), Carex bigelowii subsp. bigelowii, Eriophorum russeolum subsp. albidum (Cyperaceae), Anthoxanthum monticola subsp. monticola, *Bromus pumpellianus*, Deschampsia cespitosa subsp. cespitosa, *D. sukatschewii*, Festuca rubra subsp. rubra, *Lolium perenne*, Poa pratensis subsp. pratensis (Poaceae), *Stuckenia filiformis* (Potamogetonaceae), Potentilla × prostrata (Rosaceae), *Galium aparine* (Rubiaceae) and Salix ovalifolia var. ovalifolia (Salicaceae). Eight of these are new to the flora of the Canadian Arctic Archipelago: *Senecio lugens*, *Draba juvenilis*, *D. pilosa*, Anthoxanthum monticola subsp. monticola, *Bromus pumpellianus*, Deschampsia cespitosa subsp. cespitosa, Poa pratensis subsp. pratensis, Salix ovalifolia var. ovalifolia. One of these, *Galium aparine*, is newly recorded for the flora of Nunavut. Four of these first records for Victoria Island are introduced plants discovered in Cambridge Bay in 2017: three grasses (Festuca rubra subsp. rubra, *Lolium perenne*, and Poa pratensis subsp. pratensis) and *Galium aparine*. One taxon, Juncus arcticus subsp. arcticus, is newly recorded from the Northwest Territories.

Considering diversity in the CAVM subzones present on the island, 157 taxa are recorded in subzone C, 283 in subzone D, and 149 taxa are recorded in both subzones (Suppl. material 3). Five taxa recorded in both subzones are known from single occurrences in subzone C (*Carex vaginata*, *Cystopteris fragilis*, Equisetum arvense subsp. alpestre, *Huperzia arctica*, *Woodsia glabella*). Eight taxa are recorded in subzone C that are not recorded in subzone D: Braya thorild-wulffii subsp. glabrata, *Cardamine bellidifolia*, *Draba pauciflora*, Deschampsia cespitosa subsp. cespitosa (borderline subzone C/D), *Puccinellia bruggemannii*, *Ranunculus sabinei*, *Ranunculus sulphureus* and Saxifraga flagellaris subsp. platysepala. A total of 134 taxa are recorded in subzone D that are not recorded in subzone C.

Within the Northwest Territories, 125 taxa are recorded in the West Prince Albert Lowland Mid Arctic (MA) ecoregion, 37 in the West Prince Albert Upland MA ecoregion, six in the East Prince Albert Plain MA ecoregion, 51 in the Shaler Mountains MA ecoregion, 76 in the Tahiryuak Upland MA ecoregion, 29 in Wollaston Peninsula MA ecoregion and 231 in Prince Albert Coastlands Low Arctic-north ecoregion (Suppl. material 3).

Of the eight general areas on Victoria Island that have been botanically explored the most, the greatest diversity of vascular plants is recorded in Ulukhaktok, where 194 taxa are known (Table 2). The next most-diverse sites, in descending order of diversity, are Cambridge Bay (183 taxa), Johansen Bay (181), Kuujjua R. (176), the head of Minto Inlet (173), Boot Inlet (139), Oterkvik Point (127), Sinclair Cr. (85) and Ovayok Territorial Park (57). If the flora of Ovayok is considered as part of the Cambridge Bay area, 187 taxa are recorded in the area; only four species are recorded from Ovayok that are not otherwise known from the Cambridge Bay area (*Cystopteris fragilis*, Poa abbreviata subsp. abbreviata, *Potentilla uschakovii*, *Tofieldia pusilla*), whereas there are many species recorded from Cambridge Bay not known to occur in the park.

**Figure 7.**
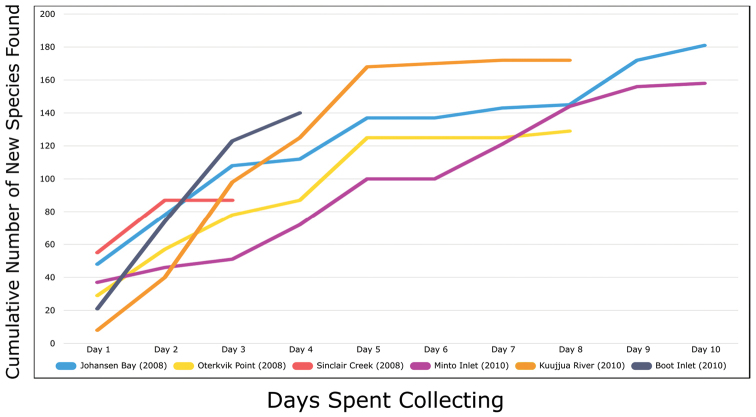
Species discovery curves for six sites that were intensively surveyed by Gillespie et al. for vascular plant biodiversity in 2008 (Johansen Bay, Oterkvik Point, Sinclair Creek) and 2010 (Boot Inlet, Kuujjua River, the head of Minto Inlet).

Species discovery curves for each of the six areas we aimed to document comprehensively in 2008 and 2010 each indicate a generally consistent increase in number of new species found with each additional day of exploration (Fig. 7). Curves for all six sites either reach or approach a plateau, indicating a slowing down in the number of new species documented on the last day(s) we spent in each area.

**Table 2.** Vascular plants recorded from Victoria Island. The table records whether or not each taxon is recorded in Northwest Territories and Nunavut, eight sites on the island that have been explored comprehensively (NWT: Ulukhaktok, Boot Inlet, Kuujjua River, the head of Minto Inlet; NU: Oterkvik Point, Johansen Bay, Sinclair Creek, Cambridge Bay) as well as Ovayok Territorial Park, Nunavut, the only protected area on Victoria Island. Occurrences at other sites are recorded in the annotated checklist and on the distribution maps. Taxa are listed by major clade, and then alphabetically by family.

Family	Taxon	Ulukhaktok	Boot Inlet	Kuujjua River	Head of Minto Inlet	Northwest Territories	Oterkvik Point	Johansen Bay	Sinclair Creek	Cambridge Bay	Ovayok Territorial Park	Nunavut
**Lycophytes**												
Lycopodiaceae	*Huperzia arctica* Sipliv.	•				▲		•				▲
**Monilophytes**												
Cystopteridaceae	*Cystopteris fragilis* (L.) Bernh.	•	•	•	•	▲	•	•			•	▲
Dryopteridaceae	*Dryopteris fragrans* (L.) Schott	•				▲		•				▲
Equisetaceae	Equisetum arvense subsp. alpestre (Wahlenb.) Schönsw. & Elven	•	•	•	•	▲		•	•	•	•	▲
	*Equisetum scirpoides* Michx.		•	•		▲						
	*Equisetum variegatum* Schleich. ex F.Weber & D.Mohr subsp. variegatum	•	•	•	•	▲	•	•	•	•	•	▲
Woodsiaceae	*Woodsia glabella* R.Br.	•		•	•	▲	•	•		•	•	▲
**Monocots**												
Cyperaceae	Carex aquatilis subsp. stans (Drejer) Hultén	•	•	•	•	▲	•	•	•	•	•	▲
	*Carex atrofusca* Schkuhr	•	•	•	•	▲	•	•	•	•	•	▲
	*Carex bicolor* All.			•		▲		•		•		▲
	Carex bigelowii subsp. lugens (Holm) T.V.Egorova	•			•	▲	•	•	•	•		▲
	Carex bigelowii Torr. ex Schwein. subsp. bigelowii						•	•		•		▲
	*Carex borealipolaris* S.R.Zhang	•	•	•	•	▲	•	•	•	•		▲
	Carex capillaris subsp. fuscidula (V.I.Krecz. ex T.V.Egorova) Á.Löve & D.Löve	•	•	•	•	▲	•	•	•	•		▲
	*Carex chordorrhiza* L.f.					▲				•		▲
	Carex fuliginosa subsp. misandra (R.Br.) Nyman	•	•	•	•	▲	•	•	•	•	•	▲
	*Carex glacialis* Mack.			•		▲	•					▲
	Carex glareosa Wahlenb. subsp. glareosa	•				▲	•	•		•		▲
	*Carex krausei* Boeckeler		•	•	•	▲	•	•				▲
	*Carex marina* Dewey	•	•	•	•	▲		•	•	•		▲
	*Carex maritima* Gunnerus	•	•	•	•	▲	•	•		•	•	▲
Cyperaceae	*Carex membranacea* Hook.	•	•	•	•	▲	•	•	•	•	•	▲
	*Carex microglochin* Wahlenb.	•				▲		•				▲
	*Carex myosuroides* Vill.	•	•	•	•	▲	•	•	•	•	•	▲
	*Carex nardina* Fr.	•	•	•	•	▲						▲
	Carex petricosa Dewey subsp. petricosa		•	•	•	▲						▲
	*Carex rariflora* (Wahlenb.) Sm.	•	•	•		▲	•	•		•		▲
	*Carex rupestris* All.	•	•	•	•	▲	•	•		•	•	▲
	*Carex saxatilis* L.	•	•	•	•	▲		•	•	•		▲
	Carex scirpoidea Michx. subsp. scirpoidea	•	•	•	•	▲	•	•	•	•	•	▲
	Carex simpliciuscula subsp. subholarctica (T.V.Egorova) Saarela	•	•	•	•	▲		•	•	•		▲
	*Carex subspathacea* Wormsk.	•		•	•	▲	•	•		•		▲
	*Carex ursina* Dewey	•	•	•	•	▲	•	•		•	•	▲
	*Carex vaginata* Tausch	•	•	•		▲		•	•	•		▲
	*Eriophorum angustifolium* Honck.	•	•	•	•	▲		•		•		▲
	*Eriophorum brachyantherum* Trautv. & C.A.Mey.		•	•	•	▲						▲
	*Eriophorum callitrix* C.A.Mey.	•		•	•	▲	•		•	•		▲
	Eriophorum russeolum subsp. albidum (F.Nyl.) Väre											▲
	Eriophorum scheuchzeri Hoppe subsp. scheuchzeri				•	▲				•		▲
	Eriophorum scheuchzeri subsp. arcticum M.S.Novos.	•	•	•	•	▲	•	•	•	•		▲
	*Eriophorum triste* (Th.Fr.) Hadač & Á.Löve	•	•	•	•	▲	•	•	•	•	•	▲
	Eriophorum vaginatum L. subsp. vaginatum	•	•	•	•	▲		•		•		▲
Juncaceae	Juncus arcticus subsp. alaskanus Hultén			•	•	▲		•		•		▲
	Juncus arcticus Willd. subsp. arcticus	•			•	▲	•			•		▲
	*Juncus biglumis* L.	•	•	•	•	▲	•	•	•	•		▲
	*Juncus leucochlamys* V.J.Zinger ex V.I.Krecz.	•		•	•	▲		•		•		▲
	Juncus triglumis subsp. albescens (Lange) Hultén	•	•	•	•	▲	•	•	•	•		▲
	*Luzula confusa* Lindeb.	•			•	▲	•	•		•		▲
Juncaceae	*Luzula nivalis* (Laest.) Spreng.	•	•	•	•	▲		•	•	•	•	▲
	*Luzula wahlenbergii* Rupr.							•				▲
Poaceae	*Alopecurus borealis* Trin.		•	•	•	▲	•	•	•	•	•	▲
	*Anthoxanthum arcticum* Veldkamp	•	•	•	•	▲	•	•	•	•		▲
	Anthoxanthum monticola (Bigelow) Veldkamp subsp. monticola											▲
	Anthoxanthum monticola subsp. alpinum (Sw. ex Willd.) Soreng							•		•		▲
	Anthoxanthum nitens (Weber) Y.Schouten & Veldkamp subsp. nitens	•				▲						
	Arctagrostis latifolia (R.Br.) Griseb. subsp. latifolia	•	•	•	•	▲	•	•	•	•	•	▲
	*Arctophila fulva* (Trin.) Andersson	•		•		▲				•		▲
	*Bromus pumpellianus* Scribn.											▲
	*Calamagrostis purpurascens* R.Br.	•	•	•	•	▲	•	•				▲
	Calamagrostis stricta subsp. groenlandica (Schrank) Á.Löve			•		▲		•				▲
	*Deschampsia brevifolia* R.Br.	•		•		▲	•		•			▲
	Deschampsia cespitosa (L.) P.Beauv. subsp. cespitosa											▲
	*Deschampsia sukatschewii* (Popl.) Roshev.			•		▲						
	*Dupontia fisheri* R.Br.	•	•	•	•	▲	•	•	•	•	•	▲
	Elymus alaskanus (Scribn. & Merr.) Á.Löve subsp. alaskanus	•	•	•	•	▲		•				▲
	Elymus alaskanus subsp. hyperarcticus (Polunin) Á.Löve & D.Löve	•	•	•	•	▲	•	•	•			▲
	*Festuca baffinensis* Polunin	•	•	•	•	▲	•	•	•	•	•	▲
	Festuca brachyphylla Schult. & Schult.f. subsp. brachyphylla	•		•	•	▲	•	•	•	•		▲
	*Festuca hyperborea* Holmen ex Fred.			•		▲						▲
	Festuca rubra subsp. arctica (Hack.) Govor.	•	•	•	•	▲	•	•		•		▲
	Festuca rubra L. subsp. rubra									•		▲
Poaceae	Leymus mollis subsp. villosissimus (Scribn.) Á.Löve & D.Löve	•		•	•	▲		•		•		▲
	*Lolium perenne* L.									•		▲
	*Phippsia algida* (Sol.) R.Br.	•		•	•	▲		•		•		▲
	*Pleuropogon sabinei* R.Br.				•	▲				•		▲
	Poa abbreviata R.Br. subsp. abbreviata			•	•	▲					•	▲
	Poa arctica R.Br. subsp. arctica	•		•	•	▲	•	•	•	•		▲
	Poa arctica subsp. caespitans Simmons ex Nannf.	•		•		▲		•		•		▲
	Poa glauca Vahl subsp. glauca	•	•	•	•	▲	•	•	•	•	•	▲
	Poa hartzii Gand. subsp. hartzii	•			•	▲	•	•		•		▲
	Poa pratensis L. subsp. pratensis									•		▲
	Poa pratensis subsp. alpigena (Lindm.) Hiitonen	•		•	•	▲	•	•	•	•	•	▲
	Poa pratensis subsp. colpodea (Th.Fr.) Tzvelev											▲
	*Puccinellia andersonii* Swallen	•	•		•	▲		•		•		▲
	*Puccinellia angustata* E.L.Rand & Redfield	•	•	•	•	▲	•			•		▲
	*Puccinellia arctica* (Hook.) Fernald & Weath.	•	•	•	•	▲	•	•		•		▲
	*Puccinellia banksiensis* Consaul						•	•	•			▲
	*Puccinellia bruggemannii* T.J.Sørensen											▲
	*Puccinellia nuttalliana* (Schult.) Hitchc.	•				▲				•		▲
	Puccinellia phryganodes subsp. neoarctica (Á.Löve & D.Löve) Elven	•	•	•	•	▲	•	•		•		▲
	Puccinellia tenella subsp. langeana (Berlin) Tzvelev	•	•	•	•	▲	•	•		•		▲
	*Puccinellia vaginata* (Lange) Fernald & Weath.		•		•	▲		•		•		▲
	*Puccinellia vahliana* (Liebm.) Scribn. & Merr.	•				▲				•		▲
	*Trisetum spicatum* (L.) K.Richt.	•	•	•	•	▲	•	•	•	•		▲
Potamogetonaceae	*Stuckenia vaginata* (Turcz.) Holub							•				▲
Potamogetonaceae	*Stuckenia filiformis* (Pers.) Börner											▲
Tofieldiaceae	*Tofieldia coccinea* Richardson	•	•	•	•	▲		•				▲
	*Tofieldia pusilla* (Michx.) Pers.	•	•	•	•	▲	•	•	•		•	▲
**Eudicots**												
Amaranthaceae	*Suaeda calceoliformis* (Hook.) Moq.		•		•	▲	•	•				▲
Asteraceae	Antennaria friesiana (Trautv.) Ekman subsp. friesiana	•	•			▲		•		•		▲
	Antennaria media subsp. compacta (Malte) Chmiel.	•	•	•	•	▲		•				▲
	Antennaria monocephala subsp. angustata (Greene) Hultén	•				▲				•		▲
	Arnica angustifolia Vahl subsp. angustifolia	•	•	•	•	▲		•		•		▲
	Artemisia borealis Pall. subsp. borealis	•			•	▲	•					▲
	Artemisia borealis subsp. richardsoniana (Besser) Korobkov	•		•	•	▲	•	•				▲
	*Artemisia hyperborea* Rydb.	•		•	•	▲						▲
	*Artemisia tilesii* Ledeb.							•		•		▲
	*Askellia pygmaea* (Ledeb.) Sennikov	•	•			▲		•	•			▲
	*Erigeron compositus* Pursh	•	•	•		▲	•	•				▲
	*Erigeron eriocephalus* J.Vahl				•	▲						▲
	*Erigeron humilis* Graham					▲		•		•	•	▲
	*Erigeron porsildii* G.L.Nesom & D.F.Murray	•				▲						
	*Eurybia sibirica* (L.) G.L.Nesom											▲
	*Hulteniella integrifolia* (Richardson) Tzvelev	•	•	•	•	▲		•		•		▲
	Petasites frigidus (L.) Fr. subsp. frigidus	•	•	•	•	▲				•		▲
	*Senecio lugens* Richardson											▲
	*Symphyotrichum pygmaeum* (Lindl.) Brouillet & Selliah		•	•	•	▲	•	•				▲
	*Taraxacum ceratophorum* (Ledeb.) DC.	•	•	•	•	▲	•	•		•		▲
	*Taraxacum holmenianum* Sahlin			•	•	▲		•	•	•		▲
Asteraceae	*Taraxacum hyparcticum* Dahlst.	•	•	•	•	▲	•	•		•		▲
	*Taraxacum phymatocarpum* J.Vahl	•	•	•	•	▲	•		•	•		▲
	*Taraxacum scopulorum* (A.Gray) Rydb.			•		▲	•					▲
	*Tephroseris frigida* (Richardson) Holub	•	•	•	•	▲		•				▲
	Tephroseris palustris subsp. congesta (R.Br.) Holub	•		•	•	▲		•	•	•		▲
	Tripleurospermum maritimum subsp. phaeocephalum (Rupr.) Hämet-Ahti	•		•		▲	•	•		•		▲
Betulaceae	*Betula glandulosa* Michx.	•	•	•		▲	•	•				▲
Boraginaceae	*Mertensia drummondii* (Lehm.) G.Don											▲
	Mertensia maritima subsp. tenella (Th.Fr.) Elven & Skarpaas	•	•	•	•	▲		•		•		▲
Brassicaceae	Braya glabella Richardson subsp. glabella	•	•	•	•	▲	•	•	•	•		▲
	Braya glabella subsp. purpurascens (R.Br.) Cody			•	•	▲				•		▲
	Braya humilis (C.A.Mey.) B.L.Rob. subsp. humilis	•	•		•	▲	•	•	•	•		▲
	Braya thorild-wulffii Ostenf. subsp. thorild-wulffii				•	▲						▲
	Braya thorild-wulffii subsp. glabrata J.G. Harris											▲
	*Cardamine bellidifolia* L.											▲
	*Cardamine digitata* Richardson	•	•	•	•	▲	•	•	•	•		▲
	*Cardamine polemonioides* Rouy	•		•	•	▲		•		•		▲
	*Cochlearia groenlandica* L.	•	•		•	▲		•	•	•		▲
	*Crucihimalaya bursifolia* (DC.) D.A.German & A.L.Ebel									•		▲
	*Descurainia sophioides* (Fisch. ex Hook.) O.E.Schulz	•		•	•	▲			•	•		▲
	*Draba arctica* J.Vahl	•				▲				•		▲
	*Draba cinerea* Adams	•	•	•	•	▲	•	•	•	•	•	▲
	*Draba corymbosa* R.Br. ex DC.	•	•	•	•	▲	•	•		•	•	▲
	*Draba fladnizensis* Wulfen	•				▲						▲
	*Draba glabella* Pursh	•	•		•	▲	•	•	•	•		▲
	*Draba juvenilis* Kom.	•				▲		•	•	•		▲
Brassicaceae	*Draba lactea* Adams	•		•	•	▲			•	•		▲
	*Draba micropetala* Hook.									•		▲
	*Draba nivalis* Lilj.	•				▲		•	•	•		▲
	*Draba norvegica* Gunn.									•		▲
	*Draba oblongata* R.Br. ex DC.	•			•	▲				•		▲
	*Draba oligosperma* Hook.											▲
	*Draba pauciflora* R.Br.											▲
	*Draba pilosa* Adams ex DC.		•	•		▲	•	•		•		▲
	*Draba simmonsii* Elven & Al-Shehbaz	•		•	•	▲				•	•	▲
	*Draba subcapitata* Simmons		•		•	▲				•	•	▲
	*Erysimum coarctatum* Fernald											▲
	*Erysimum pallasii* (Pursh) Fernald	•	•	•	•	▲	•	•	•	•		▲
	*Eutrema edwardsii* R.Br.	•	•	•	•	▲	•	•	•	•		▲
	*Parrya arctica* R.Br.	•	•	•	•	▲	•	•		•	•	▲
	*Parrya nudicaulis* (L.) Regel											▲
	*Physaria arctica* (Wormsk. ex Hornem.) O’Kane & Al-Shehbaz	•	•	•	•	▲	•	•	•	•	•	▲
Campanulaceae	*Campanula uniflora* L.		•	•	•	▲						▲
Caryophyllaceae	*Arenaria humifusa* Wahlenb.			•		▲						
	*Arenaria longipedunculata* Hultén							•				▲
	*Cerastium arcticum* Lange	•	•	•	•	▲	•					▲
	*Cerastium beeringianum* Cham. & Schltdl.	•		•	•	▲	•	•	•	•	•	▲
	*Cerastium regelii* Ostenf.		•	•		▲				•		▲
	Honckenya peploides subsp. diffusa (Hornem.) Hultén	•	•	•	•	▲	•	•	•	•		▲
	*Sabulina elegans* (Cham. & Schltdl.) Dillenb. & Kadereit	•		•	•	▲						▲
	*Sabulina rossii* (R.Br. ex Richardson) Dillenb. & Kadereit	•	•	•	•	▲				•	•	▲
	*Sabulina rubella* (Wahlenb.) Dillenb. & Kadereit	•	•	•	•	▲	•	•	•	•	•	▲
	*Sabulina stricta* (Sw.) Rchb.							•				▲
	*Sagina caespitosa* Lange											▲
	*Sagina nivalis* Fr.				•	▲				•		▲
Caryophyllaceae	*Silene acaulis* (L.) Jacq.	•	•	•	•	▲	•	•		•	•	▲
	Silene involucrata (Cham. & Schltdl.) Bocquet subsp. involucrata	•	•	•	•	▲	•	•	•	•		▲
	Silene involucrata subsp. tenella (Tolm.) Bocquet	•		•		▲				•		▲
	*Silene ostenfeldii* (A.E.Porsild) J.K.Morton		•		•	▲	•	•				▲
	Silene uralensis (Rupr.) Bocquet subsp. uralensis	•	•	•	•	▲	•	•	•	•		▲
	Silene uralensis subsp. arctica (Th.Fr.) Bocquet	•		•	•	▲	•	•		•	•	▲
	*Stellaria crassifolia* Ehrh.	•				▲		•		•		▲
	*Stellaria humifusa* Rottb.	•	•	•	•	▲		•		•		▲
	*Stellaria longipes* Goldie	•	•	•	•	▲	•		•	•	•	▲
Celastraceae	*Parnassia kotzebuei* Cham. ex Spreng.				•	▲		•				▲
Ericaceae	*Andromeda polifolia* L.							•				▲
	*Arctous alpina* (L.) Nied.	•			•	▲						
	*Arctous rubra* (Rehder & E.H.Wilson) Nakai	•	•	•	•	▲	•	•	•	•	•	▲
	Cassiope tetragona (L.) D.Don subsp. tetragona	•	•	•	•	▲	•	•		•	•	▲
	*Empetrum nigrum* L.							•				▲
	*Rhododendron lapponicum* (L.) Wahlenb.	•		•		▲	•	•				▲
	Orthilia secunda subsp. obtusata (Turcz.) Böcher	•		•		▲		•				▲
	Pyrola grandiflora Radius subsp. grandiflora	•	•	•	•	▲	•	•				▲
	Vaccinium vitis-idaea subsp. minus (Lodd., G. Lodd. & W. Lodd.) Hultén							•				▲
Fabaceae	*Astragalus alpinus* L.	•	•	•	•	▲	•	•		•		▲
	*Astragalus richardsonii* E.Sheld.	•	•	•	•	▲	•	•	•	•		▲
	*Hedysarum americanum* (Michx.) Britton	•	•	•	•	▲	•	•	•			▲
	Hedysarum boreale subsp. mackenziei (Richardson) S.L.Welsh	•	•	•	•	▲	•	•		•	•	▲
	Lupinus arcticus S.Watson subsp. arcticus											▲
	Oxytropis arctica R.Br. var. arctica	•	•	•	•	▲	•	•	•	•		▲
	*Oxytropis arctobia* Bunge	•	•	•	•	▲	•			•		▲
	Oxytropis deflexa var. foliolosa (Hook.) Barneby				•	▲						
Fabaceae	*Oxytropis maydelliana* Trautv.	•				▲	•	•	•	•		▲
	*Oxytropis varians* (Rydb.) K.Schum.	•				▲						▲
Gentianaceae	Gentianella propinqua (Richardson) J.M.Gillett subsp. propinqua	•	•	•	•	▲						▲
	Lomatogonium rotatum (L.) Fr. subsp. rotatum	•	•	•	•	▲		•				▲
Haloragaceae	*Myriophyllum sibiricum* Kom.							•		•		▲
Lentibulariaceae	*Pinguicula vulgaris* L.			•		▲		•				▲
Linaceae	Linum lewisii Pursh subsp. lewisii		•	•	•	▲						▲
Montiaceae	*Montia fontana* L.	•				▲						▲
Onagraceae	*Chamaenerion latifolium* (L.) Sweet	•	•	•	•	▲	•	•	•	•	•	▲
	*Epilobium arcticum* Sam.	•	•	•	•	▲		•	•	•		▲
Orchidaceae	*Corallorhiza trifida* Châtel.							•				▲
Orobanchaceae	*Castilleja elegans* Malte	•	•	•	•	▲	•	•	•	•		▲
	Castilleja pallida var. caudata (Pennell) B.Boivin									•		▲
	*Pedicularis albolabiata* (Hultén) Kozhevn	•	•	•	•	▲	•	•		•		▲
	*Pedicularis arctoeuropaea* (Hultén) Molau & D.F.Murray		•	•	•	▲		•	•	•		▲
	*Pedicularis capitata* Adams	•	•	•	•	▲	•	•	•	•	•	▲
	*Pedicularis hirsuta* L.					▲						
	*Pedicularis lanata* Willd. ex Cham. & Schltdl.	•	•	•	•	▲	•	•		•		▲
	Pedicularis langsdorffii subsp. arctica (R.Br.) Pennell ex Hultén	•	•	•	•	▲	•	•	•	•		▲
Papaveraceae	*Papaver cornwallisense* D.Löve		•	•	•	▲		•	•			▲
	*Papaver dahlianum* Nordh.	•	•	•		▲	•			•		▲
	*Papaver hultenii* Knaben	•	•	•	•	▲	•	•	•	•		▲
	*Papaver lapponicum* (Tolm.) Nordh.	•	•	•		▲				•		▲
Plantaginaceae	*Hippuris lanceolata* Retz.	•	•			▲	•	•		•		▲
	*Plantago canescens* Adams	•	•	•	•	▲		•		•		▲
Plumbaginaceae	*Armeria scabra* Pall. ex Roem. & Schult.	•	•	•	•	▲	•	•	•	•		▲
Polygonaceae	*Bistorta vivipara* (L.) Delarbre	•	•	•	•	▲	•	•		•	•	▲
	*Oxyria digyna* (L.) Hill	•	•	•	•	▲	•	•	•	•	•	▲
Primulaceae	Androsace chamaejasme subsp. andersonii (Hultén) Hultén	•	•	•		▲	•			•	•	▲
	*Androsace septentrionalis* L.	•	•	•	•	▲	•	•	•	•		▲
	*Primula stricta* Hornem.	•				▲	•	•				▲
Ranunculaceae	*Anemone parviflora* Michx.	•	•	•	•	▲	•	•	•	•	•	▲
	Caltha palustris subsp. radicans (T.F.Forst.) Hook.									•		▲
	*Halerpestes cymbalaria* (Pursh) Greene	•				▲	•	•				▲
	*Pulsatilla nuttalliana* (DC.) Spreng.											▲
	*Ranunculus arcticus* Richardson	•		•	•	▲	•	•		•	•	▲
	*Ranunculus codyanus* B.Boivin	•				▲				•		▲
	Ranunculus gmelinii DC. subsp. gmelinii	•		•		▲		•		•	•	▲
	Ranunculus hyperboreus Rottb. subsp. hyperboreus	•			•	▲				•		▲
	*Ranunculus nivalis* L.	•	•	•	•	▲				•		▲
	*Ranunculus pygmaeus* Wahlenb.	•		•		▲		•		•		▲
	*Ranunculus sabinei* R.Br.					▲						▲
	*Ranunculus sulphureus* Sol.											▲
Rosaceae	Dryas integrifolia Vahl subsp. integrifolia	•	•	•	•	▲	•	•	•	•		▲
	Potentilla anserina subsp. groenlandica Tratt.	•				▲		•				▲
	Potentilla arenosa (Turcz.) Juz. subsp. arenosa	•	•		•	▲	•	•		•		▲
	Potentilla arenosa subsp. chamissonis (Hultén) Elven & D.F.Murray	•			•	▲				•	•	▲
	Potentilla hyparctica Malte subsp. hyparctica					▲						
	*Potentilla nivea* L.	•		•		▲		•		•		▲
	*Potentilla pedersenii* (Rydb.) Rydb.	•		•	•	▲				•	•	▲
	Potentilla × prostrata Rottb.						•					▲
	*Potentilla pulchella* R.Br.	•			•	▲	•	•		•		▲
	*Potentilla subgorodkovii* Jurtzev	•		•	•	▲						▲
	*Potentilla subvahliana* Jurtzev	•	•		•	▲						▲
	*Potentilla tikhomirovii* Jurtzev					▲		•		•		▲
	*Potentilla uschakovii* Jurtzev	•	•	•	•	▲	•	•			•	▲
Rosaceae	*Potentilla vulcanicola* Juz.	•				▲						
	*Rubus chamaemorus* L.											▲
Rubiaceae	*Galium aparine* L.									•		▲
Salicaceae	Salix alaxensis (Andersson ex DC.) Coville var. alaxensis		•	•	•	▲		•				▲
	*Salix arctica* × *Salix polaris*	•	•	•		▲		•		•	•	▲
	*Salix arctica* Pall.	•	•	•	•	▲	•	•	•	•	•	▲
	*Salix arctophila* Cockerell ex A.Heller						•					▲
	Salix glauca var. stipulata Flod.							•		•	•	▲
	*Salix niphoclada* Rydb.	•	•	•	•	▲	•	•				▲
	Salix ovalifolia Trautv. var. ovalifolia											▲
	*Salix planifolia* Pursh											▲
	*Salix polaris* Wahlenb.			•	•	▲				•		▲
	*Salix reticulata* L.	•	•	•	•	▲	•	•		•	•	▲
	*Salix richardsonii* Hook.	•	•	•	•	▲	•	•	•	•	•	▲
Saxifragaceae	*Chrysosplenium rosendahlii* Packer	•		•		▲				•		▲
	*Chrysosplenium tetrandrum* Th.Fr.	•			•	▲				•		▲
	*Micranthes foliolosa* (R.Br.) Gornall	•				▲						▲
	*Micranthes hieraciifolia* (Waldst. & Kit. ex Willd.) Haw.									•		▲
	*Micranthes nivalis* (L.) Small	•		•	•	▲		•	•	•		▲
	*Micranthes tenuis* (Wahlenb.) Small	•		•	•	▲						
	Saxifraga rivularis subsp. arctolitoralis (Jurtzev & V.V.Petrovsky) M.H.Jørg. & Elven	•				▲		•		•		▲
	*Saxifraga aizoides* L.	•	•	•	•	▲	•	•	•	•	•	▲
	*Saxifraga cernua* L.	•	•	•	•	▲	•	•	•	•	•	▲
	*Saxifraga cespitosa* L.	•	•	•	•	▲	•	•	•	•		▲
	Saxifraga flagellaris subsp. platysepala (Trautv.) A.E.Porsild					▲						▲
	*Saxifraga hirculus* L.	•	•	•	•	▲	•	•	•	•	•	▲
	*Saxifraga hyperborea* R.Br.	•		•	•	▲		•		•		▲
	*Saxifraga oppositifolia* L.	•	•	•	•	▲	•	•		•		▲
	*Saxifraga rivularis* L.	•				▲				•		•
	*Saxifraga tricuspidata* Rottb.	•	•	•	•	▲	•	•	•	•		•
	**Total**	**194**	**139**	**176**	**173**	**237**	**127**	**181**	**85**	**183**	**57**	**277**

## Discussion

We recorded 272 species and 289 taxa on Victoria Island, including 21 taxa newly reported for the island. This represents an increase of 4.3% from the 277 taxa previously recorded from Victoria Island (Aiken et al. 2007, Gillespie et al. 2015, Saarela et al. 2017b), which includes several first records we previously reported from the island based on 2008 and 2010 collections. Eight taxa are newly recorded for the Canadian Arctic Archipelago, bringing the number of taxa known for the region to 383, a 2.1% increase relative to the 375 taxa previously known from the region (Gillespie et al. 2015). This increase in vascular plant diversity documented in the study area, based on new fieldwork and study and re-evaluation of herbarium material, is consistent with the results of our other floristic studies of Arctic areas, which followed the same approach (Saarela et al. 2013, Gillespie et al. 2015, Saarela et al. 2017b). It is likely there are few areas of the Canadian Arctic where focused botanical exploration would be unlikely to result in an increase in the number of documented species in a local flora.

The greatest regional vascular plant diversity on Victoria Island is recorded from Ulukhaktok (188 species, seven infraspecific taxa, one hybrid) and the next-greatest diversity is recorded from Cambridge Bay (176 species, 11 infraspecific taxa, one hybrid), with about 3.5% fewer taxa recorded than Ulukhaktok. This was an unexpected result, as we had predicted the Cambridge Bay area to be richer because (1) there has been more exploration and collecting there (1422 unique collections in our dataset) compared to Ulukhaktok (915 unique collections), and (2) the Cambridge Bay area, as we have defined it, including the area east of the community along the road to Ovayok Territorial Park and west of the community to the Augustus Hills area, is considerably larger (200 km^2^) than the Ulukhaktok area (16.5 km^2^) where collections have been made. In addition to different levels of species richness, the floras of the two areas are dissimilar. Although a total of 150 taxa are documented in both areas, 43 are recorded from Uluhaktok that are not known from Cambridge Bay and 33 from Cambridge Bay that are not known from Ulukhaktok; 61 taxa recorded on the island are not recorded from either area (Table 2). Some of the differences are likely attributable to variation in geology in the areas, with both acidic and calcareous rocks in the immediate Ulukhaktok area and primarily calcareous ones in the Cambridge Bay area. Species with a preference for granitic substrates recorded in Ulukhaktok include Anthoxanthum nitens subsp. nitens, *Arctous alpina*, *Carex nardina*, *Dryopteris fragrans*, *Huperzia arctica* and *Rhododendron lapponicum*; none of these is known from Cambridge Bay, though all but *Anthoxanthum nitens* are recorded elsewhere on southeastern Victoria Island in areas where granitic outcrops are known, such as the Ferguson Lake area north of Cambridge Bay. The flora of Ulukhaktok also contains a strong component of species with western or Beringian distributions that do not extend to southeastern Victoria Island or are not yet recorded from southeastern Victoria Island if they do extend that far eastwards on the island. Examples of such taxa are *Salix niphoclada*, Artemisia borealis subsp. richardsoniana, *Erigeron compositus*, *E. porsildii*, *Hedysarum americanum*, *Gentianella propinqua* and *Lomatogonium rotatum*. The flora of Cambridge Bay, reciprocally, includes taxa with eastern or amphi-Atlantic distributions not known from as far west as Ulukhaktok, such as Carex bigelowii subsp. bigelowii, as well as taxa that are at their northern limits along southern Victoria Island, such as *Carex chordorrhiza*, Castilleja pallida var. caudata, *Myriophyllum sibiricum* and Salix glauca var. stipulata. The 57 taxa recorded from Ovayok Territorial Park is an underrepresentation of true diversity within the park because no collectors have yet attempted to complete a comprehensive survey of the park flora, and at least a few taxa have been observed in the park that are not yet documented by vouchers, as noted in the annotated checklist.

The distribution maps presented here serve as updates to those produced over the decades (Porsild 1957, 1964, Porsild and Cody 1980, Aiken et al. 2007). Compared to these, our study is novel in that every mapped occurrence is supported by a voucher specimen (this is not the case in Porsild (1957) and Porsild and Cody (1980), for example) and we cite all voucher specimens. In most cases we were able to confirm occurrences of species on Victoria Island mapped in one or more of the earlier works by matching them with specimens. The maps also include all of our recent collections as well as numerous historical collections that were not considered in earlier treatments. Our study includes the first detailed distribution mapping on Victoria Island for some Arctic taxa whose circumscriptions have been revised in light of recent taxonomic study, including *Papaver* and *Potentilla*. In both of these genera, distribution maps covering the entire Canadian Arctic Archipelago and/or the Arctic mainland are not yet available. Distribution maps following recent taxonomy are available, however, for northern Quebec and Labrador for many taxa (Payette 2013, 2015, 2018).

There are few introduced vascular plant species in the Canadian Arctic Archipelago, and none that are considered to be invasive (Aiken et al. 2007). Accordingly, the vascular flora of Victoria Island comprises 98.6% native taxa. Only four taxa recorded on Victoria Island are non-native: three grasses, Festuca rubra subsp. rubra, *Lolium perenne*, Poa pratensis subsp. pratensis, and a bedstraw (*Galium aparine*). All of these were collected in 2017, in Cambridge Bay, growing in the same spot, where they were likely planted as part of a seed mixture. *Galium aparine* likely grew from seed contaminating the grass seed mixture; the taxon is likely extirpated, as we collected the single plant seen at the site. We do not know if any of these taxa persist at the site. Although there is currently no evidence for the occurrence of widespread invasion of non-native vascular plants on Victoria Island, Cambridge Bay and Ulukhaktok, in particular, should be regularly monitored for possible introductions and persistence of such taxa.

The numerous first records of taxa for Victoria Island reported by Gillespie et al. (2015) and here are variously based on collections made as part of our recent fieldwork, re-evaluation of existing herbarium material, and processing and study of historical collections that had not previously been available for scientific study. First records of taxa reported here based on our fieldwork include those collected by Bennett (Anthoxanthum monticola subsp. monticola, *Artemisia tilesii*, *Crucihimalaya bursifolia*), Gillespie et al. (*Deschampsia sukatschewii*, *Draba juvenilis*, *D. pilosa*, *D. simmonsii*, Potentilla × prostrata), Ponomarenko (Eriophorum russeolum subsp. albidum, *Stuckenia filiformis*) and Saarela (Festuca rubra subsp. rubra, *Galium aparine*, *Lolium perenne*, Poa pratensis subsp. pratensis). A subset of first records of species for Victoria Island reported here are based on a combination of recently collected material plus material newly processed from herbarium backlog that was collected years ago (Carex bigelowii subsp. bigelowii, *Taraxacum scopulorum*) or re-identified (*Draba fladnizensis*). Newly processed specimens from herbarium backlog also resulted in new first records for the island (Deschampsia cespitosa subsp. cespitosa, Potentilla hyparctica subsp. hyparctica), as did study of existing herbarium material, including some that was apparently overlooked in previous work (*Bromus pumpellianus* [previously misidentified], Salix ovalifolia var. ovalifolia [previously misidentified], *Senecio lugens*). These results underscore the importance of field exploration in combination with careful herbarium research, which is generally more time consuming than the field work component of floristic research, when attempting to characterize the flora of an area.

In addition to first records for Victoria Island, the current study documents many new sites for species previously recorded from one or more sites on the island. The majority of these are collections that close major or minor gaps in species’ known distributions. For example, our 2008 fieldwork across southern Victoria Island–in areas where no or few collections had previously been made–resulted, as expected, in collections that close numerous gaps in distribution for taxa otherwise documented elsewhere on the island, across the Canadian Arctic Archipelago and on the adjacent mainland. Our fieldwork on southeastern Victoria Island resulted in collections from many sites that had not previously been explored or documented (e.g., the Greiner Lake watershed, “Trunsky Lake”, “30-Mile Creek”). Some new site records reported here variously represent extensions to the known ranges of species, to the north (*Gentianella propinqua*, *Tofieldia pusilla*), south (*Festuca hyperborea*) and west (Juncus arcticus subsp. arcticus). In a few cases, we report additional collections for species newly reported for the island in Gillespie et al. (2015), based on additional recent collections (e.g., *Carex bicolor*) or previously unreported collections gathered decades ago but only now liberated from herbarium backlog (e.g., *Arenaria humifusa*, *Eriophorum brachyantherum*). Study of existing herbarium material resulted in “discovery” of many collections that were apparently not considered in previous Canadian Arctic floristic efforts, including collections made by A. Dutilly at Boot Inlet, R. Hainault at Mt. Lady Pelly and a subset of the collections made by J.D.H. Lambert at Long Lake. Review and processing of relevant material from the CAN backlog resulted in records from sites from which no or few collections have otherwise been made, including collections by M. Oldenburg from “Oldenburg Lake”, inland sites on the Prince Albert Peninsula, and Read Island. Similarly, the Edlund backlog material from Victoria Island reported here comprises collections from sites from which other collections were already processed at CAN and sites that were not previously represented in herbarium material, like some inland sites on the Storkerson Peninsula. The material collected at Cambridge Bay by Oldenburg in 1944 and Polunin in 1947, which was processed from the CAN backlog as part of this study and is newly published here, did not reveal any new records for the area. However, these collections, being 70+ years old, provide an important temporal element to understanding the flora of the Cambridge Bay region, as Oldenburg and Polunin were the first botanists (amateur and professional, respectively) to make extensive collections there. Polunin’s Cambridge Bay collection records a number of species not present among Oldenburg’s collection from the same area three years earlier, such as *Carex atrofusca*, C. bigelowii subsp. bigelowii, Carex capillaris subsp. fuscidula, *Carex myosuroides*, Equisetum variegatum subsp. variegatum and *Juncus biglumis*.

Thirty-nine taxa are known on the island from a single collection, seven from two collections and three from three collections. All 47 of these taxa may be considered rare on the island, and efforts should be made to discover additional populations. Many of the taxa known from a single collection have not been seen in decades, being known only from collections made in 1915 (*Eurybia sibirica*, *Mertensia drummondii*), 1945 (*Pedicularis hirsuta*), 1946 (Potentilla hyparctica subsp. hyparctica), 1949 (Anthoxanthum nitens subsp. nitens, *Montia fontana*), 1952 (*Potentilla vulcanicola*), 1959 (Braya thorild-wulffii subsp. glabrata, *Draba norvegica*), 1962 (Salix ovalifolia var. ovalifolia), 1964 (*Bromus pumpellianus*, *Erysimum coarctatum*, *Pulsatilla nuttalliana*, *Rubus chamaemorus*, *Salix planifolia*, *Senecio lugens*), 1986 (*Cardamine bellidifolia*) and 1987 (*Draba pauciflora*, *Sagina caespitosa*). The exact original collecting sites for all the collections made prior to the 1980s would likely be impossible to re-locate, since locality information on specimen labels is brief and imprecise. For example, the 1964 collections are part of a larger set of 48 specimens gathered by J.D.H. Lambert from “Long Lake” (Kellogok), a linear lake some 10 km in length with its northwestern end included in (or surrounded by) the southeastern portion of Ovayok Territorial Park. Additional information provided on the labels is brief, including plot numbers 21–27, the coordinates 69°07'N, 104°34'W, and the habitat “sedge meadow” on a subset of collections. The coordinates mark a spot about 750 m west of the mid-point of the lake. We do not know how accurate these coordinates are, nor if they were determined by the collector or secondarily by another worker based on the named locality. The recorded plot numbers suggest the collections were made as part of an ecological study, but we have not been able to align them with published or unpublished research. Other species recorded by Lambert from “Long Lake” include the rare species Lupinus arcticus subsp. arcticus (three collections) and the heaths *Empetrum nigrum*, Rhododendron tomentosum subsp. decumbens, *Vaccinium uliginosum* and V. vitis-idaea subsp. minus, most of which are uncommon on the island. The presence of the heaths indicates acidic substrate in the “Long Lake” area, which on southeastern Victoria Island is otherwise known only from the Wellington Bay area. Efforts should be undertaken to explore the “Long Lake” area to try and re-locate the occurrence(s) of these rare taxa, most of which should be relatively conspicuous, particularly if/when in flower, and which have not been seen in the area in over 50 years.

## Species discovery at 2008 and 2010 study areas

The species discovery curves for the six sites on Victoria Island at which we aimed to document all vascular plant diversity present indicate, not surprisingly, that species discovery is directly correlated with search intensity: as more days were spent at a site, more species were found. Search intensity is also a function of the number of searchers active in an area and their field botany skills, taxonomic expertise and knowledge of and experience with the flora under study. Some Arctic species are difficult to identify, especially in the field (high-powered magnification is often needed to observe diagnostic characters), such as those in the genera *Draba* and *Potentilla* and in groups that field botanists – in the Arctic and elsewhere – tend to be less familiar with, like the grasses (Poaceae) and sedges (Cyperaceae). Specialist knowledge is usually needed to locate and recognize diversity in such challenging groups in the field. Our own experience on Victoria Island serves as an example of this: the Canadian Arctic flora was brand new to one of us (J.M. Saarela) on our 2008 expedition. Reflecting on experience gained with the Canadian Arctic vascular plant flora in both the field and herbarium over the subsequent ten-year period, it is likely that some species present at sites studied in the plant families focused on by that individual (grasses, sedges, rushes) during that expedition were overlooked (J.M. Saarela, pers. obs.). Reciprocally, *Puccinellia* expert Laurie Consaul, who was also part of the 2008 expedition, focused on documenting diversity in that challenging genus on Victoria Island. Results, based on targeted search efforts, included her locating new populations of *P. banksiensis*, a species described as new to science that same year that was not then known from the island (Consaul et al. 2008a). This demonstrates how deep experience in a particular taxon, in the field and herbarium, can result in novel discoveries that would likely have otherwise been overlooked by workers less familiar with the group. We suggest the following general rule of thumb for collecting plants in the Canadian Arctic, especially when working in areas that are logistically difficult to access and unlikely to be re-visited by botanists: when one is unsure of the identity of a taxon in the field, and especially if one is unsure of whether or not they have already collected a particular taxon at a site or in an area, make a collection. This was our approach, for example, with the genus *Draba*, most species of which we could not reliably identify in the field. Nearly every time we encountered a *Draba*, we made a collection, which explains why one apparently common species, *D. cinerea*, is among the most collected on Victoria Island.

Vascular plant species diversity in the Canadian Arctic is correlated with habitat diversity. A large subset of species in the Arctic tend to be dominant and widespread, present wherever suitable habitat occurs; these species are easy to find. On the other hand, many vascular plant species in the Arctic tend to be uncommon on the landscape and occur in microhabitats that do not reflect the dominant vegetation in an area. Examples of microhabitats we encountered on Victoria Island with interesting vascular plant diversity included bird perches (rocks, cliffs), shallow freshwater ponds and south-facing slopes. Locating and searching as many microhabitats as possible results in discovery of the greatest number of species, as we found on Victoria Island. Accordingly, the general locations of our camp sites were chosen by targeting areas that appeared, on topographical maps, to be topographically diverse, ideally including local variation in elevation, aspect, moisture and geology. Ability to survey as many habitats as possible in an area is related to the amount of time available for searching and the diversity of the landscape. The number of days we spent in each area were determined based on our estimate of how long it would take to thoroughly explore the local habitat diversity, though other factors also affected this, such as availability of helicopter support and weather, which greatly affects logistical planning in the Arctic. Exactly where we were able to establish base camps was dependant on suitable areas to land a Twin Otter plane on the tundra in order to establish a camp, the availability of helicopter support for establishing a camp, logistical and financial support for plane and helicopter time, and weather.

The species discovery curves (Fig. 7) show that as the days progressed in each of our study areas, new species continued to be found, because we specifically targeted our searches in new habitats and areas. In most areas studied, the number of new species found either plateaued or approached plateau after several days of local exploration. The large spike in the number of new species recorded on day five in the Oterkvik Point area corresponds to travel to sites by helicopter, including stops at coastal areas where we collected numerous species not present inland in the area. More distant sites visited by helicopter staging from Oterkvik Point (Clouston Bay, Colville Mts.), however, are not included in the diversity count for that area because they are too far away to reasonably be considered as part of the Oterkvik Point region. The spike in the number of new species recorded in the Johansen Bay area on the ninth day in that area is a result of collections gathered during a ca. 16 kilometer (round trip) hike from our camp to the coast. Plateaus in the mid portions of some of the species discovery curves generally correspond to days that were spent processing field collections (i.e., pressing plants, taking tissue samples, recording field notes) made on the previous one or two days. Accordingly, no or few new collections representing species that had not yet been recorded at the site were made on those days because field exploration was limited. Species diversity discovered at the Sinclair Creek site was the lowest among the six intensive study areas because we spent only three days there, the least amount of time spent at any of the sites. Moreover, the total area covered by our brief exploration in the vicinity of Sinclair Creek was 1 km^2^, compared to the Johansen Bay and Oterkvik Pt. areas, which were 355 km^2^ and 212 km^2^, when helicopter sites are taken into account.

## Diversity in ecological zones

### Bioclimate subzones

Vascular plant biodiversity in the Arctic is correlated with summer warmth, with diversity declining substantially from south to north. A total of 75–150 species is expected in local floras across bioclimate subzone C and 125–250 in subzone D, based on research by Young (1971) and followed by CAVM Team (2003). Although Young (1971) did not explicitly define his concept of “local flora”, his examples indicate that the he was referring to floras of islands, parts of islands (e.g., the Inner Fjord District of Spitzbergen; coastal fringes of Ellesmere Island; the Eureka area of Ellesmere Island), groups of islands (e.g., Franz Josef archipelago), and geographically defined mainland areas like the Boothia and Melville peninsulas. The levels of diversity documented in seven local areas of Victoria Island that may be considered relatively comprehensively documented botanically are within the range of diversity estimated for local floras in subzone D, in which all the sites are located (Boot Inlet: 139 taxa; Cambridge Bay: 183; Johansen Bay: 181; Kuujjua River: 176; the head of Minto Inlet: 173; Oterkvik Point: 127; Ulukhaktok: 194). Subzone D is richer in vascular plant diversity than is subzone C on Victoria Island. A total of 280 taxa are recorded from the subzone D portion of Victoria Island, a level of diversity slightly higher than expected if this area were considered as a local flora. No local or regional areas within subzone C on Victoria Island are comprehensively documented, but the diversity recorded across the subzone (157 taxa) is just slightly higher than the maximum expected by Young (1971) for a local flora within the subzone. Many of the 134 species recorded in subzone D but not in subzone C on Victoria Island reach their known north limits in subzone D, as noted in the annotated checklist.

Comparisons of documented levels of vascular plant diversity among the various bioclimate and ecological zones defined on Victoria Island and with diversity recorded within the subzones elsewhere in the Canadian Arctic provides insight into how complete current documentation of the vascular flora is across the island. Several taxa on the island recorded from both bioclimate subzones C and D are known from single occurrences in subzone C. Although uncommon (or poorly documented) in subzone C on the island, all but one are common and/or known from multiple collections elsewhere in the subzone either in the eastern and northern Arctic (*Cystopteris fragilis*) or more or less throughout the subzone (western, eastern and northern) (Equisetum arvense subsp. alpestre, *Huperzia arctica*, *Woodsia glabella*). Their rareness in subzone C on the island may therefore be a result of collection bias, which seems likely for *E. arvense*, or lack of appropriate habitat, which seems likely for the fern taxa, which grow on acidic substrates that are uncommon on Victoria Island. The presence of *Carex vaginata* in subzone C is a borderline occurrence, as the species is known elsewhere in that subzone only from one area of Banks Island. Of the seven species recorded in subzone C but not in subzone D on Victoria Island, four occur elsewhere in subzone D in Canada: *Cardamine bellidifolia*, which is widespread in several parts of subzone D, and *Puccinellia bruggemannii*, *Ranunculus sabinei* and *R. sulphureus*, which are recorded mostly along the northern edge of subzone D (Aiken et al. 2007). One species is not known from subzone D (or E) elsewhere in Canada (*Draba pauciflora*), one is known only from one area of subzone D (Saxifraga flagellaris subsp. platysepala, recorded from southern Banks Island), and one (Deschampsia cespitosa subsp. cespitosa) is known only from subzones D and E in Canada (Aiken et al. 2007). The single occurrence of this last taxon on Victoria Island, collected at Washburn Lake, is ca. 1 km north of the subzone C/D boundary, and it may be equally appropriate to consider it to be within subzone D.

There is also considerable variation in levels of vascular plant diversity recorded within subzone C on Victoria Island. For example, within the Nunavut portion of subzone C, lower diversity (101 taxa) is recorded from the northern part of the subzone, here arbitrarily defined as comprising Storkerson Peninsula, the head of and west side of Hadley Bay, and Natkusiak Peninsula, than is recorded from the rest of the subzone in Nunavut. Of the species recorded from the more northerly Nunavut portion of subzone C, 18 are not recorded from the more southerly Nunavut portion of the subzone (Caltha palustris subsp. radicans, *Cardamine bellidifolia*, *Carex nardina*, *Cerastium arcticum*, *Chamaenerion latifolium*, *Eriophorum angustifolium*, *Micranthes foliolosa*, *Papaver dahlianum*, *Pleuropogon sabinei*, *Potentilla uschakovii*, *Puccinellia angustata*, *P. vaginata*, *P. vahliana*, Ranunculus hyperboreus subsp. hyperboreus, *R. nivalis*, *R. sulphureus*, *Sagina nivalis*, Saxifraga flagellaris subsp. platysepala). All but four of these (*Cardamine bellidifolia*, *Micranthes foliolosa*, *Ranunculus sulphureus* and Saxifraga flagellaris subsp. platysepala) are recorded in the Nunavut portion of subzone D on the island. It is therefore likely that the remaining 13 species occur within the southern Nunavut portion of subzone C, but are as yet unrecorded. Reciprocally, there are 43 species recorded in the southern Nunavut portion of subzone C on Victoria Island that are not recorded in the northern Nunavut portion of the subzone (*Armeria scabra*, Arnica angustifolia subsp. angustifolia, *Astragalus richardsonii*, Braya thorild-wulffii subsp. glabrata, *Carex atrofusca*, C. bigelowii subsp. lugens, *C. borealipolaris*, *C. chordorrhiza*, C. fuliginosa subsp. misandra, *C. maritima*, *C. saxatilis*, C. simpliciuscula subsp. subholarctica, Cassiope tetragona subsp. tetragona, Deschampsia cespitosa subsp. cespitosa, *Draba arctica*, *D. glabella*, *D. nivalis*, *D. pauciflora*, *D. simmonsii*, Elymus alaskanus subsp. alaskanus, Equisetum arvense subsp. alpestre, *Erigeron eriocephalus*, *Eriophorum brachyantherum*, *E. callitrix*, Festuca rubra subsp. arctica, Juncus triglumis subsp. albescens, *Oxytropis maydelliana*, *Papaver lapponicum*, *Potentilla subgorodkovii*, *Puccinellia arctica*, *Ranunculus codyanus*, R. gmelinii subsp. gmelinii, *R. pygmaeus*, Salix alaxensis var. alaxensis, *S. arctica* × *S. polaris*, *S. niphoclada*, *S. polaris*, *Saxifraga aizoides*, Silene uralensis subsp. arctica, *Symphyotrichum pygmaeum*, *Taraxacum ceratophorum*, *T. hyparcticum*, *Tofieldia pusilla*, *Woodsia glabella*). A subset of these are expected to be present within the northern Nunavut portion of the subzone, given their documented occurrences on nearby islands to the north and east (Aiken et al. 2007): Cassiope tetragona subsp. tetragona, Carex fuliginosa subsp. misandra, *Taraxacum hyparcticum*, *Draba nivalis*, *D. pauciflora*, *D. simmonsii*, Equisetum arvense subsp. alpestre, *Ranunculus pygmaeus*, Silene uralensis subsp. arctica. Others (*Arnica angustifolia*, *Carex maritima*, *Draba arctica*, *D. glabella*, *Erigeron eriocephalus*, *Ranunculus codyanus*, *Taraxacum ceratophorum*) that are recorded elsewhere in the Canadian Arctic Archipelago from central to western Melville Island, the eastern islands and the northern Queen Elizabeth Islands, but are apparently absent (if present, they are not yet recorded) from a broad area in the central Archipelago (Aiken et al. 2007), may or may not occur in the northern Nunavut portion of subzone C on Victoria Island; their absence there may be part of the distribution gap. The rest are at the edge of their range and/or reach their northern limit, at least in the western Archipelago, elsewhere on Victoria Island (Aiken et al. 2007); discovery of any of these on northeastern Victoria Island would represent range extensions.

Species are not distributed evenly throughout subzone D across southern Victoria Island. The composition of the vascular flora of the southeastern part of the island differs somewhat from the southwestern part. As a way to quantitatively assess this, we arbitrarily divided the subzone at Wellington Bay. We found that 178 species are known from both east and west of Wellington Bay, 216 are recorded from east of the bay and 224 from west of the bay. These differences are likely due to a combination of the historical biogeography of the region, in that a subset of species present on the southwestern part of the island, such as those with western or Beringian distributions that are at the edges of their ranges on western and southwestern Victoria Island, are likely absent on the southeastern part, and collection bias, in that some species that are present have not yet been documented in areas east or west of the bay. Species recorded from subzone D on southwestern but not southeastern Victoria Island that are not known from nor expected to occur on the southeastern part of the island, given knowledge of their broader distributions (Aiken et al. 2007), are *Arenaria longipedunculata*, Artemisia borealis subsp. richardsoniana, *Artemisia hyperborea*, Carex petricosa subsp. petricosa, *Draba oligosperma*, *Elymus alaskanus*, *Erigeron compositus*, *Eurybia sibirica*, Gentianella propinqua subsp. propinqua, *Halerpestes cymbalaria*, *Hedysarum americanum*, *Linum lewisii*, Lomatogonium rotatum subsp. rotatum, *Mertensia drummondii*, *Oxytropis varians*, *Parnassia kotzebuei*, *Parrya nudicaulis*, *Puccinellia banksiensis*, Salix alaxensis var. alaxensis, *Salix niphoclada*, *Silene ostenfeldii*, *Suaeda calceoliformis* and *Taraxacum scopulorum*. Discovery of any of these on southeastern Victoria Island would represent range extensions. Some that require acidic substrates (*Andromeda polifolia*, *Carex glacialis*, *Luzula wahlenbergii*) may be rare or absent. Species known from southwestern Victoria Island that are not yet recorded but likely to exist on the southeastern part of the island, given their broader distributions across the Canadian Arctic Archipelago (Aiken et al. 2007), are *Calamagrostis purpurascens*, C. stricta subsp. groenlandica, *Campanula uniflora*, *C. microglochin*, *Cerastium arcticum*, Potentilla anserina subsp. groenlandica, *P. × prostrata* and *Salix arctophila*. Species recorded in subzone D on southeastern but not southwestern Victoria Island that are not expected to be present on the southwestern part of the island, based on their known distributions (Aiken et al. 2007), include Anthoxanthum monticola subsp. monticola, *Cerastium regelii*, *Draba micropetala*, *D. norvegica*, *Pleuropogon sabinei* and *Sagina caespitosa*. Those expected to be present on the southwestern part of the island but not yet recorded there, based on their known distributions elsewhere on the island and/or across the Archipelago (Aiken et al. 2007), include Antennaria monocephala subsp. angustata, *Arctous alpina*, *Carex chordorrhiza*, *C. nardina*, *Chrysosplenium tetrandrum*, *Crucihimalaya bursifolia*, Petasites frigidus subsp. frigidus, Poa pratensis subsp. colpodea, *Puccinellia vahliana*, *Ranunculus codyanus*, Ranunculus hyperboreus subsp. hyperboreus, *Sagina nivalis*, *Salix polaris* and *Tofieldia coccinea*. Some species recorded from the southeastern part of the island are rare, known from a single collection, which, in a subset of cases, are the only records for the Canadian Arctic Archipelago: *Bromus pumpellianus*, Castilleja pallida var. caudata, Eriophorum russeolum subsp. albidum, *Erysimum coarctatum*, *Pulsatilla nuttalliana*, *Rubus chamaemorus*, Salix ovalifolia var. ovalifolia, *Salix planifolia*, *Senecio lugens* and *Stuckenia filiformis*.

### Northwest territories ecological regions

Our data indicate very low documented species diversity for most of the Northwest Territories ecological regions. With the exception of the Prince Albert Coastlands Low Arctic North ecoregion, which includes Ulukhaktok and the areas around Minto Inlet we intensively surveyed in 2010 (Kuujjua River, head of Minto Inlet and Boot Inlet) and that several other collectors have visited, low recorded diversity is due to collection bias rather than reflecting true levels of vascular plant diversity. Aside from our 2010 efforts, there has been no attempt to comprehensively document local floras at sites/areas within the ecological regions, and most have only been cursorily explored. For example, in the East Prince Albert Plain Mid-Arctic Ecoregion, spanning 9,866 km^2^, only six species are known, all recorded from one site, by S. Edlund, on the east side of Richard Collinson Inlet. Within the West Prince Albert Upland Mid-Arctic Ecoregion collections representing 37 taxa were been made by M. Oldenburg at a single site, “Oldenburg Lake”, all of which are newly reported here. In the Shaler Mountains Mid-Arctic Ecoregion 51 taxa are recorded, in the Wollaston Peninsula Mid-Arctic Ecoregion just 29 taxa are recorded, and collections representing 76 taxa have been made in the Tahiryuak Upland Mid-Arctic Ecoregion. Considerable field work will be needed to accurately document levels of vascular plant diversity in most of these regions, which may be considered underexplored botanically.

## Conclusion

This study has substantially increased our understanding of the diversity and distribution of the vascular plant flora of Victoria Island. The results represent a new baseline of knowledge on which continued exploration of the flora of the island can build. We have provided documentation of several taxa new to the island and increased knowledge of the spatial distribution of taxa across the island. Study of existing herbarium material, which was greatly facilitated by recent mass-digitization efforts at several institutions, revealed a large number of occurrence records that have been overlooked in previous efforts to document the vascular flora of Vascular Island. Nearly 50 taxa on the island are rare, known by three or fewer collections. Although more than 7000 unique collections of vascular plants have been made on Victoria Island, many areas remain unexplored or poorly explored botanically. Numerous species that are expected to occur within bioclimate subzones C on the island have not yet been recorded there, while many species reach their northern limits in bioclimate subzone D on the island. We expect (and hope) that many new collections of vascular plants will be gathered over the coming years, particularly related to CHARS research activity in the Cambridge Bay area, on southeastern Victoria Island and across the entire island, and that the information on the distribution and diversity of vascular plant diversity on the island presented here will help guide future documentation efforts. The complete, specimen-based dataset published here will allow future workers to generate updated distribution maps including new occurrence records. We strongly encourage all researchers conducting botanical or plant-related ecological research on Victoria Island and throughout the Canadian Arctic to document species occurrences with specimens, and to deposit the material in one or more publically accessible herbaria, so that the collections may contribute to the centuries long mission to document Arctic biodiversity.

## Annotated checklist

### Key to major groups

**Table:** 

1	Plants not producing flowers or seeds, reproducing by spores	**2**
–	Plants producing flowers and seeds	**4**
2	Stems conspicuously jointed, bearing at each node a whorl of small, scale-like leaves united at the base	** Equisetaceae **
–	Stems not conspicuously jointed, bearing green leaves or leaf-like structures, leaves not whorled, usually not scale-like	**3**
3	Leaves lanceolate to triangular, not divided; sporangia axillary	** Lycopodiaceae **
–	Leaves (fronds) pinnately divided; sporangia borne on lower surface of leaves	** Polypodiales **
4	Leaves usually parallel-veined; parts of the perianth usually in threes or sixes; seeds with a single cotyledon	**Monocots**
–	Leaves usually net-veined; parts of the perianth usually in fours or fives; seeds with two cotyledons	**Eudicots**

### Key to Polypodiales [adapted from Porsild (1964) and Porsild and Cody (1980)]:

**Table:** 

1	Fronds once pinnate, lower pinnae sometimes notched or toothed; sori with a divided, hair-like indusium	** Woodsiaceae **
–	Fronds 2–3 pinnate or pinnatifid; sori with a hooded lateral indusium, or without indusia	**2**
2	Sori with a distinct lateral indusium; fronds thin, somewhat translucent; stipe thin, fragile, more or less translucent, easily disarticulated from a lateral rhizome	** Cystopteridaceae **
–	Sori without an indusium; fronds stout; stipe stout, not fragile, firmly attached to a stout rootstock	** Dryopteridaceae **

### Key to Monocots

**Table:** 

1	Perianth absent or inconspicuous and of bracts, scales or bristles	**2**
–	Perianth present, in two distinct whorls	**4**
2	Flowers not enclosed in or subtended by bracts, scales or bristles; plants aquatic; leaves submersed or floating; stipules present, adnate to the blade for 2/3 to nearly the entire length of the stipule; inflorescences submersed	** Potamogetonaceae **
–	Flowers enclosed in or subtended by bracts, scales or bristles; plants usually terrestrial, rarely aquatic; leaves erect, rarely floating (*Arctophila*, *Pleuropogon*); stipules absent; inflorescences never submersed if plants aquatic	**3**
3	Stems round, hollow between nodes; leaves 2-ranked; leaf sheaths usually open, sometimes closed; anthers attached at the middle; fruit a caryopsis	** Poaceae **
–	Stems triangular or round, solid; leaves 3-ranked; leaf sheaths closed; anthers attached at the base; fruit an achene	** Cyperaceae **
4	Plants rootless and leafless, mycotrophic; flowers zygomorphic	** Orchidaceae **
–	Plants with roots and leaves, autotrophic; flowers actinomorphic	**5**
5	Perianth conspicuous, deciduous; tepals white, yellowish-green, greenish, pinkish cream or deep crimson; leaves equitant (having the base folded and partly enclosing the leaf above), laterally flattened	** Tofieldiaceae **
–	Perianth relatively inconspicuous, persistent; tepals green to brown or purplish black; leaves not equitant, dorsi-ventrally flattened or round	** Juncaceae **

### Key to Eudicots

**Table:** 

1	Plants aquatic	**2**
–	Plants terrestrial	**4**
2	Leaf blades entire	**Plantaginaceae (*Hippuris*)**
–	Leaf blades finely divided	**3**
3	Leaves in whorls; flowers unisexual, 4-merous, petals lacking or inconspicuous and pinkish	** Haloragaceae **
–	Leaves alternate; flowers bisexual, 5-merous, petals conspicuous, yellow or white	**Ranunculaceae (*Ranunculus*)**
4	Plants with woody or partly lignified creeping or erect stems (shrubs or sub-shrubs)	**5**
–	Plants herbaceous	**9**
5	Leaves finely divided or lobed	**Asteraceae (*Artemisia*)**
–	Leaves not divided or lobed, the margins at most crenulate or serrate	**6**
6	Petals absent; flowers arranged in catkins	**7**
–	Petals present; flowers variously arranged, not in catkins	**8**
7	Plants monoecious; fruits 2-winged samaras, 1-seeded, seeds lacking hairs	** Betulaceae **
–	Plants dioecious; fruits capsules, many seeded, seeds bearing a tuft of hairs	** Salicaceae **
8	Stamens 10 or fewer	**Ericaceae (*Andromeda* , *Arctous* , *Cassiope* , *Empetrum* , *Rhododendron* , *Vaccinium*)**
–	Stamens 50+	**Rosaceae (*Dryas*)**
9	Flowers arranged in dense heads on a common receptacle (the inflorescence having the appearance of a single flower)	** Asteraceae **
–	Flowers arranged variously, but not on a common receptacle	**10**
10	Flowers with a single perianth	**11**
–	Flowers with a double perianth	**15**
11	Leaves whorled	** Rubiaceae **
–	Leaves not whorled	**12**
12	Leaves divided or deeply lobed	**Ranunculaceae (*Anemone* , *Anemonastrum* , *Pulsatilla*)**
–	Leaves not deeply lobed or divided	**13**
13	Stipules present, fused and sheathing (ocrea)	** Polygonaceae **
–	Stipules absent	**14**
14	Annuals; fruit a utricle	** Amaranthaceae **
–	Perennials; fruit a capsule	**Saxifragaceae (*Chrysosplenium*)**
15	Petals fused	**16**
–	Petals distinct, not fused	**23**
16	Stems scapose	**17**
–	Stems leafy	**20**
17	Scape 1-flowered; plants carnivorous (leaves sticky and glandular, trapping and digesting insects)	** Lentibulariaceae **
–	Scape several-flowered; plants not carnivorous	**18**
18	Inflorescences simple, spicate	**Plantaginaceae (*Plantago*)**
–	Inflorescences compound	**19**
19	Inflorescences umbels; fruits capsular	** Primulaceae **
–	Inflorescences dense hemispheric heads of scorpioid cymes; fruits dry, enclosed by persistent calyces	** Plumbaginaceae **
20	Leaves alternate	**21**
–	Leaves opposite	**22**
21	Inflorescence a single flower; corolla campanulate; ovary inferior; fruit a capsule	** Campanulaceae **
–	Inflorescences corymbose; corolla tubular or funnel-form; ovary superior; fruit an aggregate of nutlets	** Boraginaceae **
22	Flowers actinomorphic	** Gentianaceae **
–	Flowers zygomorphic	** Orobanchaceae **
23	Cauline leaves opposite	**24**
–	Cauline leaves alternate, or plants with a basal rosette	**26**
24	Plants annual	** Montiaceae **
–	Plants perennial	**25**
25	Ovary inferior	**Onagraceae (*Epilobium*)**
–	Ovary superior	** Caryophyllaceae **
26	Flowers strongly zygomorphic	** Fabaceae **
–	Flowers actinomorphic or slightly zygomorphic	**27**
27	Pistils 2-several	**28**
–	Pistil 1	**31**
28	Perianth and androecium attached to the receptacle below the ovary (hypogynous)	**29**
–	Perianth and androecium fused at the base to form a hypanthium (perigynous)	**30**
29	Staminodes present	** Celastraceae **
–	Staminodes absent	** Ranunculaceae **
30	Stipules present	**Rosaceae (*Potentilla, Rubus*)**
–	Stipules absent	**Saxifragaceae (*Micranthes*)**
31	Petals 4	**32**
–	Petals 5	**34**
32	Ovary inferior	**Onagraceae (*Chamaenerion*)**
–	Ovary superior	**33**
33	Sepals 4; stamens 6, in two distinct whorls, outer ones short, inner ones long; ovary 2-locular	** Brassicaceae **
–	Sepals 2; stamens many, not in two distinct whorls; ovary 1-locular	** Papaveraceae **
34	Ovary 1-locular	**Saxifragaceae (*Saxifraga*)**
–	Ovary 2-several-locular	**35**
35	Leaves basal (or appearing so), blades ovate, broadly ovate, elliptic, orbiculate, round or subreniform; petals green, greenish white, yellowish white, white, pinkish, or reddish	**Ericaceae (*Orthilia, Pyrola*)**
–	Leaves cauline, blades linear, linear-lanceolate or linear-oblanceolate; petals usually blue, sometimes whitish	** Linaceae **

### Lycopods

**Lycopodiales**

**Lycopodiaceae [1/1]**

***Huperzia* Bernh. [1]**

**Figure 8.**
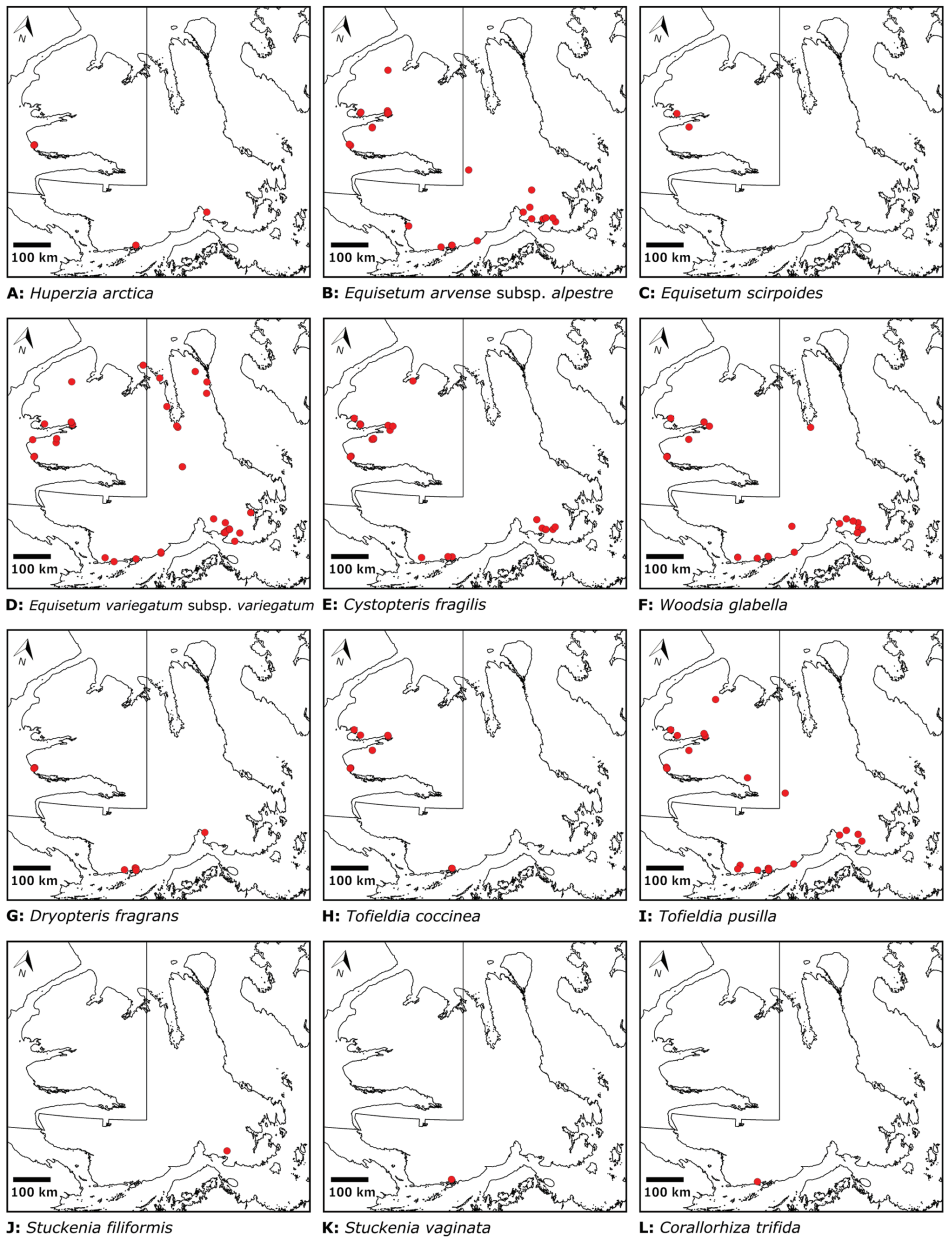
Species distribution maps. Lycopodiaceae: **A***Huperzia arctica*. Equisetaceae: **B**Equisetum arvense subsp. alpestre**C***Equisetum scirpoides***D**Equisetum variegatum subsp. variegatum. Cystopteridaceae: **E***Cystopteris fragilis*. Woodsiaceae: **F***Woodsia glabella*. Dryopteridaceae: **G***Dryopteris fragrans*. Tofieldiaceae: **H***Tofieldia coccinea***I***Tofieldia pusilla*. Potamogetonaceae: **J***Stuckenia filiformis***K***Stuckenia vaginata*. Orchidaceae: **L***Corallorhiza trifida*.

***Huperzia arctica*** (Grossh. ex Tolm.) Sipliv. (H. selago subsp. arctica (Grossh. ex Tolm.) Á.Löve *&* D.Löve, Lycopodium selago subsp. arcticum Grossh. ex Tolm.), Fig. 8A–Arctic fir clubmoss | Circumpolar?

Previously recorded from Ulukhaktok (Porsild 1955, 1957, 1964, Porsild and Cody 1980, Aiken et al. 2007). Thannheiser et al. (2001) additionally recorded it from Richardson I. and Johansen B. (conf.). Newly recorded from Ferguson L. [Tahiryuaq] and Mukta [?] L.; the latter site is not mapped as its location is unclear. The Ferguson L. site was noted as being more acidic than other sites in the areas visited in 2014, and the taxon was associated with ericaceous shrubs. The taxon was observed and photographed in 2015 by J. Wagner at the base of the hills above Long Point (69.1265, -105.4638) west of Cambridge Bay (image! BAB and JMS); a voucher from this locality is needed. Elsewhere in the Canadian Arctic known from Baffin, Coats, Devon, Ellesmere, Melville, Prince Patrick, Somerset and Southampton islands and mainland sites (Porsild and Cody 1980, Cody and Britton 1989, Cody et al. 1989, Korol 1992, Aiken et al. 2007, Dignard 2013d, Saarela et al. 2013, Saarela et al. 2017b). Recognition of *H. arctica* as distinct from the boreal *H. selago* (L.) Bernh. ex. Schrank *&* Mart. follows Elven et al. (2011), Gilman and Testo (2015) and Dignard (2013d). Wagner and Beitel (1993) did not map *H. selago* s.l. as occurring in the Canadian Arctic, despite numerous records from the region known prior to their treatment.

**NORTHWEST TERRITORIES. Ulukhaktok**: *Edlund 476*, *745* (CAN), *Porsild 17234*, *17235* (CAN). **NUNAVUT. Ferguson L. [Tahiryuaq**]: *Bennett et al. 14-0432* (BABY). **Johansen B.**: *Gillespie et al. 7958* (CAN, O). **Mukta [?] L.**: *Hainault 2124* (DAO).

### Monilophytes

**Equisetales**

**Equisetaceae [1/3]**

***Equisetum* L. [3]**

**Key to *Equisetum* [adapted from Porsild and Cody (1980) and Hauke (1993)]:**

**Table:** 

1	Stems annual (persisting one year or less), bearing whorls of branches; cones terminal on brown unbranched reproductive stems or green branched stems; cone apex rounded	**E. arvense subsp. alpestre**
–	Stems evergreen (persisting more than one year), unbranched or forking; cones terminal on green stems; cone apex pointed	**2**
2	Teeth 3 per sheath; stem ridges 6; stems inclined and tortuous	***E. scirpoides***
–	Teeth 3–12 per sheath; stem ridges same number as teeth; stems stiff and straight	***E. variegatum*** subsp. ***variegatum***

**Figure 9.**
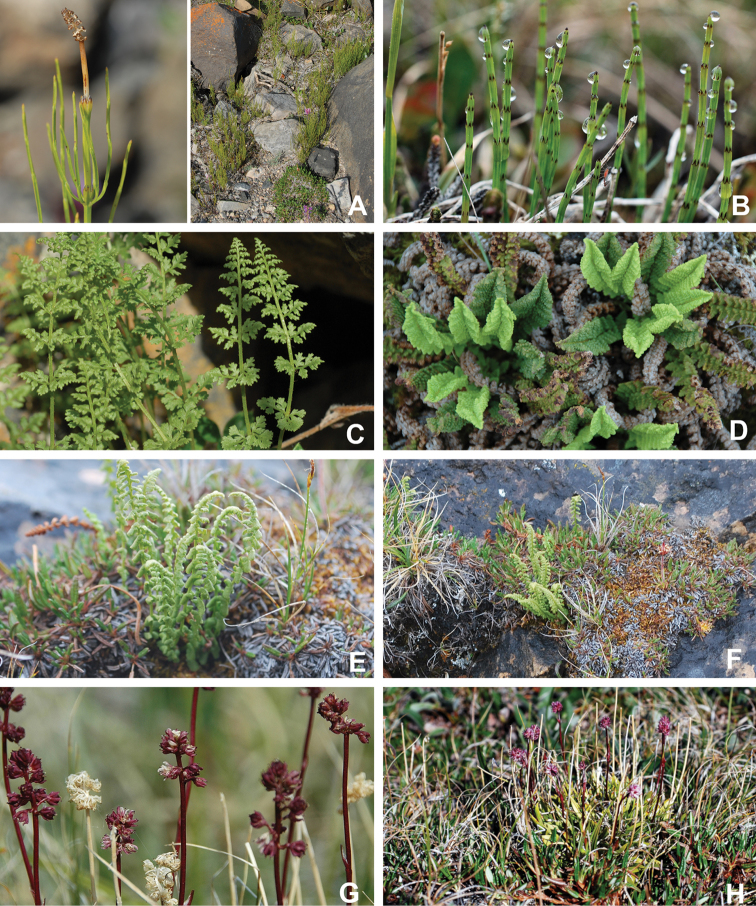
**A**Equisetum arvense subsp. alpestre strobilus (left) and habit (right), *Gillespie et al. 10109***B**Equisetum variegatum subsp. variegatum habit, *Gillespie et al. 8499***C***Cystopteris fragilis* habit, *Gillespie et al. 9990***D***Dryopteris fragrans* habit, Johansen Bay, 18 July 2008 **E***Woodsia glabella* habit **F***Woodsia glabella* habitat **G***Tofieldia coccinea* inflorescences, Kuujjua River, NT, 18 July 2010 **H***Tofieldia coccinea* habit, *Gillespie et al. 9749*. Photos **A**, **C** by L.J. Gillespie **B**, **D**, **G** by R.D. Bull **H** by J.M. Saarela and **E**, **F** by B.A. Bennett.

***Equisetum arvense*** subsp. ***alpestre*** (Wahlenb.) Schönsw. & Elven, Figs 8B, 9A–Alpine field horsetail | Circumpolar-alpine

Previously recorded from Cambridge Bay, Ferguson L., the head of Minto Inl. (Porsild obs., conf.), Mt. Pelly, inland from the head of Prince Albert S., Ulukhaktok (Porsild obs., conf.) and Walker B. (Porsild obs.) (Porsild 1955, 1957, 1964, Porsild and Cody 1980, Aiken et al. 2007). Thannheiser et al. (2001) additionally reported it from Johansen B. (conf.), Richardson I. and Surrey L. Newly recorded from Clouston B., Greiner L., Kuujjua R., Richard Collinson Inl., Sinclair Cr. and “Trunsky L.” Taxonomy follows Elven et al. (2011), who recognize northern plants as subsp. alpestre and more southerly ones as subsp. arvense. Their distributions have not been mapped for North America. *Equisetum arvense* s.l. is widespread across the Arctic mainland (Porsild and Cody 1980, Korol 1992, Cody et al. 2003, Dignard 2013c, Bennett 2015) and the Archipelago, where recorded from Axel Heiberg, Baffin, Banks, Devon, Ellesmere, King William, Somerset, Southampton and western Melville I. (Aiken et al. 2007). The apparent absence from the northeastern part of Victoria I. may be part of a broader distribution gap in the central Queen Elizabeth Islands and adjacent Prince of Wales I. (Aiken et al. 2007), assuming the taxon has not simply been overlooked in those areas.

**NORTHWEST TERRITORIES. Boot Inl.**: *Edlund 576* (CAN), *Gillespie et al. 9576* (ALA, CAN, MT, O), *9601* (ALA, CAN, O). **Kuujjua R.**: *Gillespie et al. 9797* (CAN, O), *9814* (CAN). **Minto Inl. (head)**: *Edlund 136* (CAN), *Gillespie et al. 10051* (ALA, CAN, MT, O, UBC, WIN), *10109* (ALTA, ari, CAN, MO, O, UBC, US, UTC, WIN), *10181*, *10226* (CAN). **Richard Collinson Inl.**: *Edlund 174* (CAN). **Ulukhaktok**: *Edlund 481*, *829* (CAN). **NUNAVUT. Cambridge Bay**: *Edlund & Argus 12863* (CAN), *Stephens 1100* (CAN, KANU). **Clouston B.**: *Gillespie et al. 7719*, *7757* (CAN). **Ferguson L. [Tahiryuaq**]: *Hainault 2140* (DAO). **Greiner L.**: *Ponomarenko VI-141*, *VI-203K* (CAN). **Johansen B.**: *Gillespie et al. 7935* (ALA, CAN, MT, O, UBC), *7961* (CAN, O), *8107* (CAN, O). **Ovayok TP**: *Stephens 1066* (CAN, KANU), *1109* (KANU), *1174* (CAN). **Prince Albert S. (head)**: *Edlund & Argus 12812* (CAN). “**Trunsky L.**”: *Bennett et al. 14-0395* (BABY), *14-0540* (CAN).

***Equisetum scirpoides*** Michx., Fig. 8C–Dwarf scouring-rush | Circumboreal–polar

The only previous record (unconfirmed) for Victoria I. is from Johansen B. (Thannheiser et al. 2001). Newly recorded from Boot Inl., where the species grew in a snowbed community at the base of a north-facing cliff above a lake, and the Kuujjua R. area, where it was gathered from the shore of a small round lake northeast of “Fish L.”. Elsewhere in the Canadian Arctic recorded from scattered sites on southern Baffin, Banks, Coats, King William and Southampton islands (Aiken et al. 2007) and the mainland (Porsild and Cody 1980, Cody et al. 1984a, Cody and Britton 1989, Cody et al. 1989, Dignard 2013c, Saarela et al. 2013, Saarela et al. 2017b).

**NORTHWEST TERRITORIES**: **Boot Inl.**: *Gillespie et al. 9616* (ALA, CAN). **Kuujjua R.**: *Gillespie et al. 9773* (CAN).

***Equisetum variegatum*** Schleich. subsp. ***variegatum***, Figs 8D, 9B–Variegated scouring-rush | Circumpolar-alpine

Previously recorded from Anderson B., Cambridge Bay, C. Wollaston, Ferguson L., Hadley B., Kuujjua R., the head of Minto Inl., Mt. Pelly, Namaycush L., Natkusiak P., the head of Prince Albert S. (Porsild obs.), Richard Collinson Inl., Storkerson P., Tahoe L. (Porsild obs.) and Ulukhaktok (Porsild 1955, Porsild and Cody 1980, Aiken et al. 2007). Thannheiser et al. (2001) additionally recorded it from Johansen B. (conf.) and Richardson I. Newly recorded from Albert Edward B., Boot Inl., Greiner L., Mt. Lady Pelly, Oterkvik Pt. and Sinclair Cr. Widespread throughout the Canadian Arctic islands, except those that comprise bioclimate subzone A, and across the mainland (Porsild and Cody 1980, Korol 1992, Aiken et al. 2007, Dignard 2013c, Saarela et al. 2013, Saarela et al. 2017b).

**NORTHWEST TERRITORIES. Boot Inl.**: *Gillespie et al. 9588* (CAN). **C. Wollaston**: *Edlund 28* (CAN). **Kuujjua R.**: *Edlund 643* (CAN), *Gillespie et al. 9826* (ALA, ari, CAN, O, UBC). **Minto Inl. (head)**: *Edlund 611*, *78* (CAN), *Gillespie et al. 10073* (ALA, CAN, MT, O, UBC), *9484* (CAN). **Natkusiak P.**: *Edlund 112*, *160* (CAN). **Richard Collinson Inl.**: *Edlund 175* (CAN). **Ulukhaktok**: *Edlund 753*, *802* (CAN), *Porsild 17796* (CAN). **NUNAVUT. Albert Edward B.**: *Ponomarenko* VI-260 (CAN). **Anderson B.**: *Edlund & Argus 12723* (CAN). **Cambridge Bay**: *Bennett 13-0558* (BABY, chars), *13-0619* (ALA), *Gillespie et al. 8499* (CAN), *Gould s.n.* (ALA), *Polunin s.n.* (CAN). **Ferguson L. [Tahiryuaq**]: *Hainault 2062* (DAO). **Greiner L.**: *Ponomarenko VI-133A* (CAN). **Hadley B.**: *Edlund 91*, *129*, *s.n.* (CAN). **Johansen B.**: *Gillespie et al. 8029* (CAN, MT, O). **Mt. Lady Pelly [Amaaqtuq**]: *Hainault 1842* (DAO). **Ovayok TP**: *Stephens 1066A*, *1175* (CAN). **Namaycush L.**: *Edlund 13* (CAN, US). **Natkusiak P.**: *Edlund 343* (CAN). **Oterkvik Pt.**: *Gillespie et al. 7505* (ALA, CAN, O), *Gillespie et al. 7688* (CAN). **Sinclair Cr.**: *Gillespie et al. 8260* (CAN), *8356* (CAN). **Storkerson P.**: *Edlund 213*, *285*, *299* (CAN).

### Polypodiales

**Cystopteridaceae [1/1]**

***Cystopteris* Bernh. [1]**

***Cystopteris fragilis*** (L.) Bernh., Figs 8E, 9C–Fragile fern | Cosmopolitan

Previously recorded from Cambridge Bay, Ferguson L., the head of Minto Inl., Mt. Pelly, the head of Prince Albert S. (Porsild obs.), Richard Collinson Inl., Ulukhaktok, Walker B. and Washburn L. (Porsild obs.) (Porsild 1955, 1957, 1964, Porsild and Cody 1980, Aiken et al. 2007). Thannheiser et al. (2001) additionally recorded it from Johansen B. (conf.) and Richardson I. Newly recorded from Boot Inl., Greiner L., Kuujjua R. and Oterkvik Pt. Elsewhere in the Canadian Arctic recorded from Axel Heiberg, Banks, Baffin, Coasts, Devon, Ellesmere, King William, Somerset and Southampton islands and across the mainland (Porsild and Cody 1980, Cody et al. 1989, Korol 1992, Cody et al. 2003, Cody and Reading 2005, Aiken et al. 2007, Dignard 2013a, Saarela et al. 2013, Saarela et al. 2017b).

**NORTHWEST TERRITORIES. Boot Inl.**: *Gillespie et al. 9596* (ALA, CAN, MT, O), *9608*, *9639*, *9690* (CAN). **Kuujjua R.**: *Gillespie et al. 9820* (CAN, O), *9851* (CAN), *9990* (ALA, CAN, O). **Minto Inl. (head)**: *Gillespie et al. 10216* (CAN, O), *10277* (ari, CAN, O), *Porsild 17351* (CAN). **Richard Collinson Inl.**: *Stretton 193* (DAO). **Ulukhaktok**: *Edlund 804*, *844* (CAN), *Porsild 17230* (CAN), *Saarela & Bull 1485* (CAN, O). **Walker B.**: *Porsild 17479* (CAN). **NUNAVUT. Ferguson L. [Tahiryuaq**]: *Jones & Hainault 3* (DAO). **Greiner L.**: *Ponomarenko VI-046*, *VI-192*, *VI-285* (CAN). **Johansen B.**: *Gillespie et al. 7962* (ALA, CAN, O), *8167* (CAN). **Ovayok TP**: *Gillespie et al. 8421* (CAN, UC), *Stephens 1171* (CAN), *1283* (CAN, KANU, 2 sheets). **Oterkvik Pt.**: *Gillespie et al. 7530* (CAN, UC, UTC).

### Woodsiaceae [1/1]

***Woodsia* R.Br. [1]**

***Woodsia glabella*** R.Br., Figs 8F, 9E, F–Smooth woodsia | Circumpolar–alpine

Previously recorded from Byron B., Cambridge Bay, Ferguson L., Hadley B., the head of Minto Inl., Mt. Pelly, the head of Prince Albert S. (Porsild obs.), Ulukhaktok and Walker B. (Porsild 1955, 1957, 1964, Porsild and Cody 1980, Aiken et al. 2007). Thannheiser et al. (2001) additionally recorded it from Johansen B. (conf.), Richardson I. and Surrey L. Newly recorded from Greiner L., Kuujjua R., Mt. Lady Pelly, Oterkvik Pt. and an inland site on south-central Victoria I. north of Byron B. Elsewhere in the Canadian Arctic recorded from Axel Heiberg, Baffin, Banks, Coats, Devon, Ellesmere, King William and Southampton islands and across the mainland (Porsild and Cody 1980, Korol 1992, Cody et al. 2003, Aiken et al. 2007, Dignard 2013e, Saarela et al. 2013, Saarela et al. 2017b).

**NORTHWEST TERRITORIES. Kuujjua R.**: *Gillespie et al. 9867* (ALA, CAN, O). **Minto Inl. (head)**: *Gillespie et al. 9446*, *10043* (CAN), *Porsild 17352* (CAN). **Ulukhaktok**: *Edlund 720*, *721*, *758*, *794* (CAN), *Porsild 17232*, *17233* (CAN). **Walker B.**: *Porsild 17480* (CAN). **NUNAVUT. Byron B.**: *Dushenko 24* (UVIC). **Cambridge Bay**: *Bennett et al. 13-0200* (BABY, chars), *Washburn 12* (CAN). **Ferguson L. [Tahiryuaq**]: *Bennett et al. 14-0426* (DAO), *Hainault 1954* (DAO), *Jones 2* (DAO). **Greiner L.**: *Ponomarenko VI-047* (CAN). **Hadley B.**: *Edlund 99* (CAN). **Johansen B.**: *Gillespie et al. 7902*, *7931* (CAN), *8141* (CAN, MT). **Mt. Lady Pelly [Amaaqtuq**]: *Hainault 1838* (DAO). **Ovayok TP**: *Gillespie et al. 7604* (CAN), *8423* (CAN, O), *Gould s.n.* (ALA), *Stephens 1160*, *1172* (CAN). **Oterkvik Pt.**: *Gillespie et al. 7813* (CAN). **South-central Victoria I.**: *Edlund & Argus 12875* (CAN).

### Dryopteridaceae [1/1]

***Dryopteris* Adans. [1]**

***Dryopteris fragrans*** (L.) Schott, Figs 8G, 9D–Fragrant wood fern | European (NE)–Asian–amphi-Beringian–North American (N)

Previously recorded from Ulukhaktok and Ferguson L. (Porsild 1955, 1957, 1964, Porsild and Cody 1980, Aiken et al. 2007). Thannheiser et al. (2001) additionally recorded it from Johansen B. (conf.), Richardson I. and Surrey L. Samples from Johansen Bay were included in a population genetic study of the species, in which high genetic variation among Arctic populations was found (Bouchard et al. 2017). Its restricted distribution on Victoria I. is likely due to its preference/requirement for acidic substrates. Elsewhere in the Canadian Arctic recorded from Banks, Baffin, Ellesmere and Southampton islands and numerous mainland sites (Porsild and Cody 1980, Cody et al. 1989, Korol 1992, Cody and Reading 2005, Aiken et al. 2007, Dignard 2013b, Saarela et al. 2013, Saarela et al. 2017b).

**NORTHWEST TERRITORIES. Ulukhaktok**: *Bandringa 342* (CAN, UBC), *Dutilly 18660* (MT), *Edlund 843* (CAN), *Porsild 17231* (CAN). **NUNAVUT. Ferguson L. [Tahiryuaq**]: *Hainault 1955* (DAO). **Johansen B.**: *Gillespie et al. 7901* (ALA, BABY, CAN, MT, O, UBC), *7986* (ALA, CAN, MT, O), *8024* (CAN, O), *8139* (ALA, CAN, O).

### Monocots

**Alismatales**

**Tofieldiaceae [1/2]**

***Tofieldia* Hudson [2]**

**Key to *Tofieldia* [adapted from Packer (2002)]**

**Table:** 

1	Bracts deeply 3-lobed; bracteoles absent; tepals white, cream, yellowish white or sometimes greenish white; capsules 2.5–3 mm; seeds 0.6–0.8 mm	***T. pusilla***
–	Bracts ovate, margins ± entire (sometimes absent distally); bracteoles 3-lobed; tepals white or pale pink, often tinged pink to deep purplish; capsules 2–2.3 mm; seeds ca. 1 mm	***T. coccinea***

***Tofieldia coccinea*** Richardson, Figs 8H, 9G, H–Northern tofieldia | Asian (N/C)–amphi-Beringian–North American (N)

Previously recorded from Ulukhaktok and Walker B. (Porsild 1955, 1957, 1964, Porsild and Cody 1980, Aiken et al. 2007). Thannheiser et al. (2001) additionally recorded it from the head of Minto Inl. (conf.) and Johansen B. (conf.). Newly recorded from Boot Inl. and Kuujjua R. Elsewhere in the Canadian Arctic recorded from one site on southern Banks I., Baffin and Devon islands and scattered mainland sites (Porsild and Cody 1980, Cody et al. 1989, Korol 1992, Cody and Reading 2005, Aiken et al. 2007, Gauthier 2013, Saarela et al. 2013, Saarela et al. 2017b). Both species may occur sympatrically, but *T. coccinea* tends to occur in drier sites (moist to dry) than *T. pusilla* (moist to wet).

**NORTHWEST TERRITORIES. Boot Inl.**: *Gillespie et al. 9671* (CAN). **Kuujjua R.**: *Gillespie et al. 9749* (ALA, CAN, O), *9809* (CAN, O), *9811* (ALA, ari, CAN, O). **Minto Inl. (head)**: *Gillespie et al. 10108* (CAN, MT), *10159* (CAN, O, UBC). **Ulukhaktok**: *Edlund 475*, *743* (CAN), *Porsild 17266*, *17267*, *17268* (CAN). **Walker B.**: *Porsild 17489* (CAN). **NUNAVUT. Johansen B.**: *Gillespie et al. 7941a* (ALA, CAN, MT, O, UBC), *7959a* (CAN, O).

***Tofieldia pusilla*** (Michx.) Pers., Fig. 8I–Small tofieldia | Circumpolar–alpine

Previously recorded from Ulukhaktok and Walker B. (Porsild 1955, 1957, 1964, Porsild and Cody 1980, Aiken et al. 2007) and newly recorded from Boot Inl., Ferguson L., Johansen B., Kuujjua R., the head of Minto Inl., Mt. Lady Pelly, Mt. Pelly, Oterkvik Pt., the head of Prince Albert S. and an inland site east thereof, Richard Collinson Inl. and Sinclair Cr. The site inland from the head of Richard Collinson Inl. is a northern range extension for the species in Canada. Elsewhere in the Canadian Arctic recorded from Baffin and Southampton islands and several mainland sites (Porsild and Cody 1980, Cody et al. 1989, Korol 1992, Cody and Reading 2005, Aiken et al. 2007, Gauthier 2013, Saarela et al. 2013, Saarela et al. 2017b).

**NORTHWEST TERRITORIES. Boot Inl.**: *Gillespie et al. 9590* (ALA, CAN, O). **Kuujjua R.**: *Gillespie et al. 9804* (ari, CAN). **Minto Inl. (head)**: *Edlund 157* (CAN), *Gillespie et al. 9477* (CAN, O). **Prince Albert S. (head)**: *Edlund 376* (CAN), *Edlund & Argus 12819* (CAN). **Richard Collinson Inl.**: *Edlund 674* (CAN). **Ulukhaktok**: *Edlund 449*, *744* (CAN), *Oldenburg 45-1598* (CAN), *Porsild 17269* (CAN). **Walker B.**: *Oldenburg 45-1507* (CAN), *Porsild 17490* (CAN). **NUNAVUT. Ferguson L. [Tahiryuaq**]: *Bennett et al. 14-0429* (CAN), *Hainault 2069*, *2091* (DAO), **Johansen B.**: *Gillespie et al. 7941b* (CAN), *7959b* (CAN, O), *8003* (ALA, CAN, MT, O, UBC), *8128* (ALA, CAN, MT, O). **Mt. Lady Pelly [Amaaqtuq**]: *Jones & Hainault 7*, *1844* (DAO). **Ovayok TP**: *Gillespie et al. 8432* (ALA, CAN, O). **Oterkvik Pt.**: *Gillespie et al. 7469* (CAN), *7668* (ALA, CAN, MT, O). **Sinclair Cr.**: *Gillespie et al. 8231* (ALA, CAN, MT, O, UBC).

### Potamogetonaceae [1/2]

***Stuckenia* Börner [2]**

**Key to *Stuckenia* [adapted from Haynes and Hellquist (2000) and Kaplan (2008)]**

**Table:** 

1	Leaf sheaths closed and tubular at base (connate) at least when young; stems freely branching proximally, sparsely branching distally; stipules with distinct ligules to 20 mm, especially on distal stipules; summit of midstem stipules tight to stem, about the ± same width as stem; fruit 2–3 mm	***S. filiformis***
–	Leaf sheaths open at base, even when young, often with shortly overlapping edges (convolute); stems freely branching proximally to distally; stipules without ligules or ligules to 2 mm on distal stipules; summit of midstem stipules inflated at least 2 times width of stem; fruit 3–3.8 mm	***S. vaginata***

***Stuckenia filiformis*** (Pers.) Börner (*Potamogeton filiformis* Pers., S. filiformis var. borealis (Raf.) H.St.John, S. filiformis subsp. alpina (Blytt) R.R.Haynes, Les *&* M.Král), Fig. 8J–Slender-leaved pondweed | Circumboreal–polar

Newly recorded for Victoria I., where discovered growing in a small lake (shallow water with *Hippuris lanceolata*) near Greiner L. in 2016. The site marks the northern edge of the species’ range in the central Canadian Arctic. Elsewhere in the Canadian Arctic Archipelago known only from Sylvia Grinnell Territorial Park on Baffin I. and two sites on Southampton I. (Calder 1951, Aiken et al. 2007). On the mainland Arctic known from the lower Coppermine R. area, a few sites along western Hudson Bay and northern Quebec (Korol 1992, Aiken et al. 2007, Garneau 2013, Saarela et al. 2017b).

**NUNAVUT. Greiner L.**: *Ponomarenko CB52* (CAN).

***Stuckenia vaginata*** (Turcz.) Holub (*Potamogeton vaginatus* Turcz., *S. subretusa* (Hagstr.) Holub), Fig. 8K–Big-sheathed pondweed | European (N)–Asian (C-NE)–North American

Known from a single locality in the vicinity of Johansen B., discovered in 2008; see Gillespie et al. (2015) for additional details, including photographs. This is the only record for the Canadian Arctic Archipelago. Elsewhere in the Canadian Arctic known from a few collections from the mainland Northwest Territories and southeastern Nunavut (Porsild 1950b, Porsild and Cody 1980, Cody et al. 2003, Saarela et al. 2013, Saarela et al. 2017b).

**NUNAVUT. Johansen B.**: *Gillespie et al. 8048* (ALA, ALTA, BABY, CAN, MT, O, UBC, US).

### Asparagales

**Orchidaceae [1/1]**

***Corallorhiza* Gagnebin [1]**

***Corallorhiza trifida*** Châtel., Fig. 8L–Early coralroot | Circumboreal-polar

Known from a single collection we made in 2008 at Johansen B.; see details in Gillespie et al. (2015), which includes photographs. Elsewhere in the Canadian Arctic recorded from a few sites on Baffin I. and across the mainland (Porsild and Cody 1980, Aiken et al. 2007, Saarela et al. 2013, Gillespie et al. 2015, Saarela et al. 2017b).

**NUNAVUT. Johansen B.**: *Gillespie et al. 8093* (CAN).

### Poales

**Juncaceae [2/7/8]**

**Key to Juncaceae**

**Table:** 

1	Capsules many-seeded; leaves glabrous, sheaths open	*** Juncus ***
–	Capsules 3-seeded; leaves hairy, sheaths closed	*** Luzula ***

### *Juncus* L. [4/5]

**Key to *Juncus* [adapted from Porsild and Cody (1980), Brooks and Clemants (2002) and Kirschner (2002c)]**

**Table:** 

1	Inflorescences lateral cymes, sympodial; bracts erect, terete, appearing to be continuation of culms; bracteoles 2, at base of perianth; basal leaves bladeless, cauline leaves absent; flowers borne singly, not in heads (*J. arcticus*)	**2**
–	Inflorescences terminal panicles or racemes of several heads or a single terminal head, sympodial or monopodial; bracteoles absent at base of perianth; basal leaves (at least some) usually with blade, cauline leaves present or absent; flowers in multiflowered heads	**3**
2	Flowers usually 2–5 per inflorescence; bract c. ¼–1/8 of culm	**J. arcticus subsp. arcticus**
–	Flowers 3–10 per inflorescence; bract 1/7–1/10 of culm	**J. arcticus subsp. alaskanus**
3	Plants strongly rhizomatous, culms solitary; inflorescences of 1–3(–5) heads, each 2–10-flowered; tepals lanceolate, 4.5–6.6 mm	***J. leucochlamys***
–	Plants cespitose, culms clustered; inflorescences single heads, each 1–2(–4)-flowered; tepals oblong or oblong-lanceolate, 2.5–5 mm	**4**
4	Primary bract much longer than inflorescence; capsule apex retuse; filaments 1–1.5 mm	***J. biglumis***
–	Primary bract nearly equal to or shorter than inflorescence; capsule apex obtuse, mucronate; filaments 2.5–4 mm	**J. triglumis subsp. albescens**

**Figure 10.**
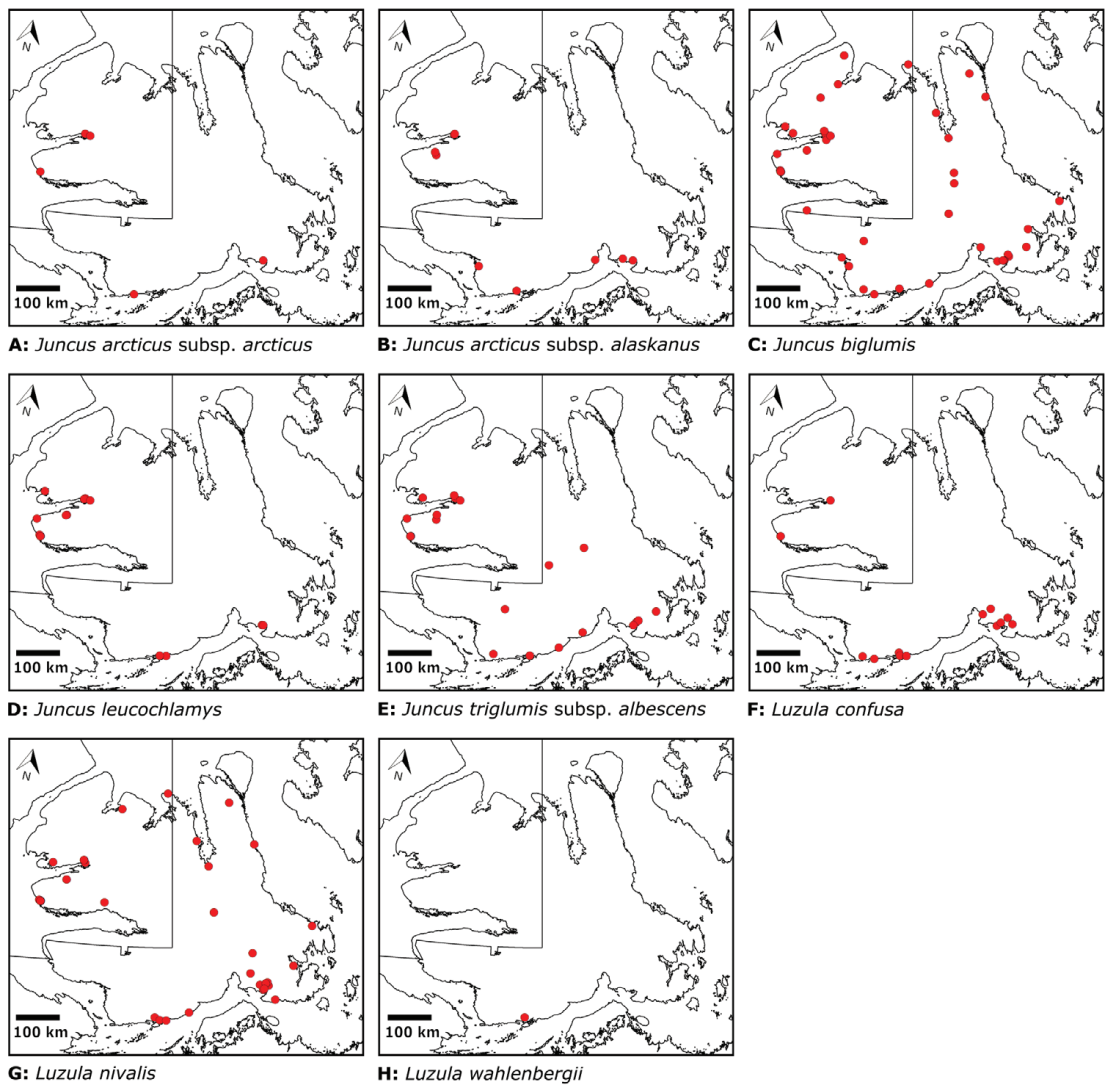
Species distribution maps. Juncaceae: **A**Juncus arcticus subsp. arcticus**B**Juncus arcticus subsp. alaskanus**C***Juncus biglumis***D***Juncus leucochlamys***E**Juncus triglumis subsp. albescens**F***Luzula confusa***G***Luzula nivalis***H***Luzula wahlenbergii*.

***Juncus arcticus*** Willd. subsp. ***arcticus***, Fig. 10A–Arctic rush | North American (NE)–amphi-Atlantic–European (N)–Asian (NW)

*Juncus arcticus* s.l. was previously recorded from Cambridge Bay, the head of Minto Inl. and Ulukhaktok (Porsild 1955, 1957, 1964, Porsild and Cody 1980, Aiken et al. 2007). We follow the taxonomy proposed by Kirschner (2002a), who recognized three subspecies (two in North America), with the caveat that assigning individuals to a taxon was sometimes difficult for Victoria I. material. These intermediate individuals may represent transitions between the subspecies (Elven et al. 2011), whose main distributions come together in the central Canadian Arctic. Subspecies arcticus is recorded from Cambridge Bay, the head of Minto Inl., Oterkvik Pt. and Ulukhaktok. These records extend the distribution of the taxon westward in Canada, and the subspecies is newly recorded for the Northwest Territories (see map in Kirschner 2002a: 166). All specimens from the eastern Canadian Arctic are considered to be this subspecies, recorded from Baffin and Southampton islands, a few sites on Ellesmere I., mainland Nunavut along western Hudson Bay and northern Quebec and Labrador (Aiken et al. 2007, Hay and Payette 2013).

**NORTHWEST TERRITORIES. Minto Inl. (head)**: *Edlund 164* (CAN), *Porsild 17375* (CAN). **Ulukhaktok**: *Edlund 778* (CAN). **NUNAVUT. Cambridge Bay**: *Saarela & Teeter 5289* (CAN). **Oterkvik Pt.**: *Gillespie et al. 7690* (CAN, O).

***Juncus arcticus*** subsp. ***alaskanus*** Hultén (J. balticus var. alaskanus (Hultén) A.E.Porsild), Fig. 10B–Alaska rush | Asian (NE)–amphi-Beringian–North American (NW)

Recorded from Kuujjua R., the head of Minto Inl., “30-Mile Cr.”, Cambridge Bay, Clouston B. and Johansen B. This western subspecies is the common one on the island. Elsewhere in the Canadian Arctic Archipelago it is recorded from Banks I., and on the mainland it extends to Bathurst Inl. and southeastern mainland Nunavut (Porsild and Cody 1980, Kirschner 2002c, Cody et al. 2003, Aiken et al. 2007, Saarela et al. 2017b).

**NORTHWEST TERRITORIES. Kuujjua R.**: *Edlund 651* (CAN), *Gillespie et al. 9911* (ALA, ari, CAN, O). **Minto Inl. (head)**: *Gillespie et al. 10150* (ALA, CAN, MT, O), *10229* (ALA, CAN, O). **NUNAVUT. “30-Mile Cr.**”: *Bennett et al. 14-0343* (BABY, chars). **Cambridge Bay**: *Edlund & Argus 12864* (CAN), *Gillespie et al. 8502* (CAN, O), *Stephens 1130* (CAN). **Clouston B.**: *Gillespie et al. 7752* (ALA, CAN, MT, O). **Johansen B.**: *Gillespie et al. 8111* (ALA, CAN, MT, O).

**Figure 11.**
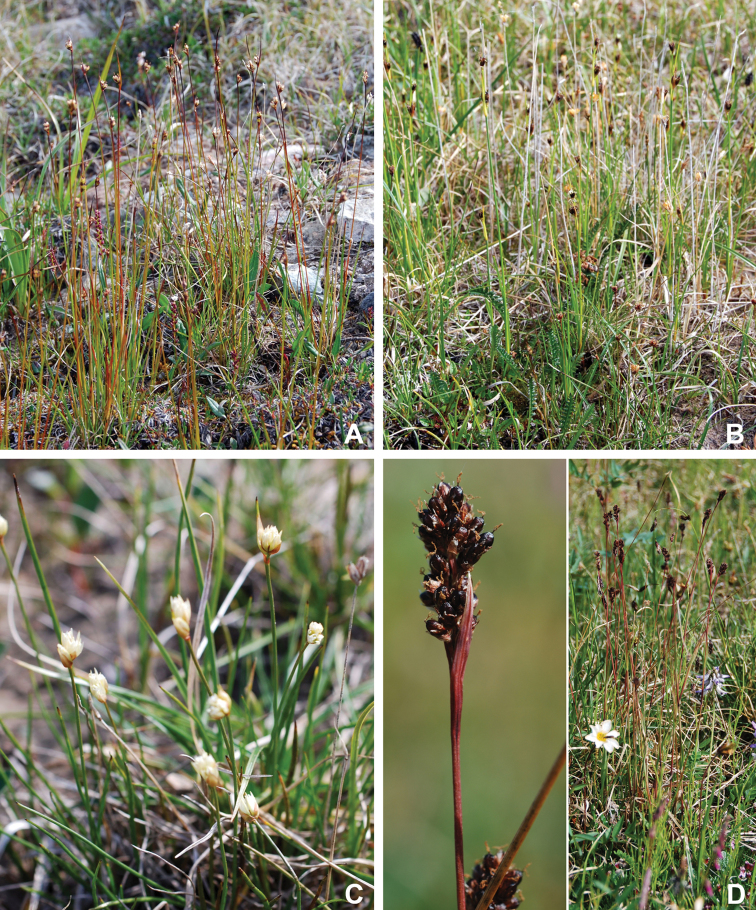
**A***Juncus biglumis* habit, Minto Inlet, NT, 22 July 2010 **B***Juncus leucochlamys* habit, *Gillespie et al. 9901***C**Juncus triglumis subsp. albescens habit, *Gillespie et al. 9859***D***Luzula nivalis* inflorescence (left) and habit (right), *Gillespie et al. 9667*. Photos by J.M. Saarela.

***Juncus biglumis*** L., Figs 10C, 11A–Two-flowered bog rush | Circumpolar-alpine

Previously recorded from Cambridge Bay, the head of Minto Inl., Natkusiak P., Richard Collinson Inl., Storkerson P., Washburn L. (Porsild obs., conf.) and Ulukhaktok (Porsild 1955, 1957, 1964, Porsild and Cody 1980, Aiken et al. 2007). Thannheiser et al. (2001) additionally recorded it from Johansen B. (conf.), Hadley B. (conf.), Richardson I., Surrey L. and Wellington B. Newly recorded from Albert Edward B., Boot Inl., C. Wollaston, Clouston B., Collinson P., Colville Mts., Ferguson L., Greiner L., Kuujjua R., Mt. Pelly, Namaycush L., “Oldenburg L.”, Oterkvik Pt., an inland site on Prince Albert P., Read I., Sinclair Cr., Walker B. and northwestern Wollaston P. Many of these collections close the conspicuous large gap across most of Victoria I. in a previous distribution map (Aiken et al. 2007). Widespread throughout the Canadian Arctic Archipelago and known from numerous mainland sites (Porsild and Cody 1980, Cody et al. 1989, Korol 1992, Aiken et al. 2007, Hay and Payette 2013, Saarela et al. 2013, Saarela et al. 2017b).

**NORTHWEST TERRITORIES. Boot Inl.**: *Gillespie et al. 9559* (ALA, CAN, MT, O). **C. Wollaston**: *Edlund 32* (CAN). **Kuujjua R.**: *Gillespie et al. 9778* (ALA, CAN, O). **Minto Inl. (head)**: *Edlund 121*, *162* (CAN), *Gillespie et al. 9501* (ALA, ari, CAN, O), *10296* (CAN, O), *17374*, *17376* (CAN). **Natkusiak P.**: *Edlund 77* (CAN). “**Oldenburg L.**”: *Oldenburg 45-1371* (CAN). **Prince Albert P.**: *Oldenburg 54*-*673* (MIN). **Richard Collinson Inl.**: *Edlund 700* (CAN). **Ulukhaktok**: *Edlund 357*, *462*, *755*, *800* (CAN), *Oldenburg 45-1601* (CAN), *Porsild 17262* (CAN), *Saarela & Bull 1484*, *1492* (CAN). **Walker B.**: *Oldenburg 45-1439* (CAN). **Wollaston P.**: *Oldenburg 54*-*494a* (MIN). **NUNAVUT. Albert Edward B.**: *Ponomarenko VI-253* (CAN). **Cambridge Bay**: *Bennett et al. 13-0240* (CAN, od), *Calder et al. 24173* (DAO), *Polunin s.n.* (CAN), *Stephens 1002*, *1158* (KANU, KSTC), *966* (KANU), *1108* (KSTC), *1037*, *1148*, *1206* (CAN, KANU, KSTC). **Clouston B.**: *Gillespie et al. 7748* (CAN). **Collinson P.**: *Edlund & Argus 12754* (CAN). **Colville Mts.**: *Gillespie et al. 7761* (CAN). **Ferguson L. [Tahiryuaq**]: *Hainault 2012* (DAO). **Greiner L.**: *Ponomarenko VI-230*, *VI-341B* (CAN). **Hadley B.**: *Edlund 66*, *144* (CAN). **Johansen B.**: *Gillespie et al. 7951* (CAN, O). **Ovayok TP**: *Bennett & Sullivan 13-0286* (chars). **Namaycush L.**: *Edlund 126* (CAN). **Oterkvik Pt.**: *Gillespie et al. 7689* (CAN), *7787* (ALA, CAN, O). **Read I.**: *Oldenburg 43-1008* (CAN). **Sinclair Cr.**: *Gillespie et al. 8238* (ALA, CAN, O). **Storkerson P.**: *Edlund 178*, *295* (CAN). **Washburn L.**: *Edlund & Argus 12795* (CAN), *Oldenburg 46-2217* (CAN).

***Juncus leucochlamys*** V.J.Zinger ex V.I.Krecz. | (*J. castaneus* Sm. pro parte., J. castaneus subsp. leucochlamys (V.J.Zinger ex V.I.Krecz.) Hultén), Figs 10D, 11B–Chestnut rush | Asian (N/C)–amphi-Beringian–North America (N)–amphi-Atlantic (W)

Previously recorded from Cambridge Bay, C. Wollaston, the head of Minto Inl. and Ulukhaktok (Porsild 1955, 1957, 1964, Porsild and Cody 1980). Thannheiser et al. (2001) additionally recorded it from Johansen B. (conf.), Hadley B., Richardson I., Surrey L. and Wellington B. Newly recorded from Kuujjua R., Murray Pt. and Walker B. Elsewhere in the Canadian Arctic recorded from Baffin, Devon, Ellesmere and Southampton islands and numerous mainland sites (Porsild and Cody 1980, Cody et al. 1989, Korol 1992, Cody and Reading 2005, Aiken et al. 2007, Hay and Payette 2013, Saarela et al. 2013, Saarela et al. 2017b). The populations on the northwest part of Victoria I. mark the species’ northwestern limit.

**NORTHWEST TERRITORIES. C. Wollaston**: *Edlund 65* (CAN). **Kuujjua R.**: *Gillespie et al. 9860* (CAN), *9901* (ari, CAN, O). **Minto Inl. (head)**: *Edlund 125* (CAN), *Gillespie et al. 10227* (ALA, CAN, MT, O), *Porsild 17377* (CAN). **Ulukhaktok**: *Edlund 359*, *505*, *799* (CAN), *Porsild 17263* (CAN). **Walker B.**: *Oldenburg 45-1438* (CAN). **NUNAVUT. Cambridge Bay**: *Edlund & Argus 12626* (CAN), *Oldenburg 44*-*899* (CAN). **Johansen B.**: *Gillespie et al. 8017* (ALA, CAN, MT, O). **Murray Pt.**: *Gillespie et al. 8201* (CAN).

***Juncus triglumis*** subsp. ***albescens*** (Lange) Hultén (*J. albescens* Lange, J. triglumis var. albescens Lange), Figs 10E, 11C–Northern white rush | Asian (N)–amphi-Beringian–North American (N)–amphi-Atlantic (W)

Previously recorded from Cambridge Bay, C. Peel, C. Wollaston, Hadley B., the head of Minto Inl., Mt. Bumpus, Namaycush L., east of the head of Prince Albert S. and Ulukhaktok. Thannheiser et al. (2001) additionally recorded it from Johansen B. (conf.), Surrey L. and Wellington B. Newly recorded from Boot Inl., Kuujjua R., the Greiner L. watershed, Oterkvik Pt. and Sinclair Cr. Elsewhere in the Canadian Arctic recorded from Axel Heiberg, Baffin, Banks, Coats, Devon, Ellesmere, King William and Southampton islands and several mainland sites (Porsild and Cody 1980, Cody et al. 1989, Korol 1992, Cody and Reading 2005, Aiken et al. 2007, Hay and Payette 2013, Saarela et al. 2013, Saarela et al. 2017b). There is a conspicuous gap in distribution in the central Canadian Arctic Archipelago (Brooks and Clemants 2002, Aiken et al. 2007).

**NORTHWEST TERRITORIES. Boot Inl.**: *Gillespie et al. 9555* (ALA, ari, CAN, MT, O, UBC). **C. Wollaston**: *Edlund 32*, *64* (CAN). **Kuujjua R.**: *Edlund 652* (CAN), *Gillespie et al. 9859* (CAN). **Minto Inl. (head)**: *Edlund 163* (CAN), *Gillespie et al. 10032* (CAN, O), *Porsild 17373* (CAN). **Ulukhaktok**: *Bliss s.n.* (ALTA), *Edlund 358*, *754*, *800*, *17261* (CAN), *Oldenburg 42-69* (CAN). **NUNAVUT. Cambridge Bay**: *Bennett et al. 13-0261* (chars, UBC), *Gillespie et al. 8440* (CAN, O), *Gould s.n.* (ALA), *Stephens 1035* (CAN, KANU, KSTC). **C. Peel**: *Edlund 2* (CAN). **Greiner L.**: *Ponomarenko VI*-231 (CAN). **Johansen B.**: *Gillespie et al. 8015* (CAN, O). **Mt. Bumpus**: *Edlund 177* (CAN). **Namaycush L.**: *Edlund 35* (CAN). **Oterkvik Pt.**: *Gillespie et al. 7788* (CAN, O). **Prince Albert S. (head)**: *Edlund & Argus 12822* (CAN). **Sinclair Cr.**: *Gillespie et al. 8336* (CAN).

### *Luzula* DC. [3]

**Key to *Luzula* [adapted from Swab (2000) and Kirschner (2002b)]**

**Table:** 

1	Inflorescences open, paniculate cymes, flowers solitary at the end of the capillary branches; seeds 1.2–1.6 mm	***L. wahlenbergii***
–	Inflorescences spike-like with flowers in heads; seeds 0.8–1.2 mm	**2**
2	Basal leaves ± flat, usually up to 5 cm, (2–)3–4 mm wide, tip obtuse, often slightly swollen, with a spinuliform mucro; cauline leaves (1–)2, 1–2(–3) cm; bracteole apices sparsely ciliate	***L. nivalis***
–	Basal leaves ± subcanaliculate to ± flat, up to 6–9 cm, 1.5–2.5 mm wide, tip acuminate; cauline leaves 1–2, usually 2–4 cm; bracteole apices fimbriate-ciliate	***L. confusa***

***Luzula confusa*** Lindeb., Fig. 10F–Northern woodrush | Circumpolar-alpine

Previously recorded from Cambridge Bay, Ferguson L. [Tahiryuaq], “Long L.”, the head of Minto Inl. and Ulukhaktok (Porsild 1955, 1957, 1964, Porsild and Cody 1980, Aiken et al. 2007). Thannheiser et al. (2001) additionally recorded it from Johansen B. (conf.), Richardson I. and Surrey L. Newly recorded from Greiner L. and Murray Pt. Observed in Ovayok TP by B.A.B. in 2013; a voucher is needed. Widespread throughout the Canadian Arctic (Porsild and Cody 1980, Cody et al. 1989, Korol 1992, Aiken et al. 2007, Hay and Payette 2013, Saarela et al. 2013, Saarela et al. 2017b). The distribution gap on the northeastern half of Victoria I. is likely an artefact of sampling, as the species is recorded from most adjacent islands to the north and east (Aiken et al. 2007).

**NORTHWEST TERRITORIES. Minto Inlet (head)**: *Porsild 17378* (CAN). **Ulukhaktok**: *Edlund 811*, *845* (CAN), *Porsild 17264* (CAN). **NUNAVUT. Cambridge Bay**: *Bennett et al. 13-0304* (ALA, BABY, CAN, chars, UBC), *14-0379* (UBC). **Ferguson L. [Tahiryuaq**]: *Bennett et al. 14-0409* (UBC), *Hainault 2074* (DAO). **Greiner L.**: *Ponomarenko VI-279B*, *VI-282B* (CAN). **Johansen B.**: *Gillespie et al. 7847* (ALA, CAN, MT, O), *7903*, *8028* (CAN, O). “**Long L.**”: *Lambert s.n.* (CAN). **Murray Pt.**: *Gillespie et al. 8207* (ALA, CAN, MT, O), *8208* (ALA, BABY, CAN, MT, O, US), *8202* (CAN, O). **Oterkvik Pt.**: *Gillespie et al. 7680* (ALA, CAN, MT, O), *7574* (ALA, CAN, O).

***Luzula nivalis*** (Laest.) Spreng. (*L. arctica* Blytt), Figs 10G, 11D–Arctic wood rush | Circumpolar-alpine

Previously recorded from Anderson B., Collinson P., Greely Haven, Hadley B., the head of Minto Inl., Namaycush L., Natkusiak P., Shaler Mts., Storkerson P. and Ulukhaktok (Porsild 1955, 1957, 1964, Porsild and Cody 1980, Aiken et al. 2007). Thannheiser et al. (2001) additionally recorded it from Johansen B. (conf.), Richardson I. and Surrey L. Newly recorded from Albert Edward B., Boot Inl., Ferguson L., Greiner L., Kuujjua R., Mt. Pelly, Murray Pt., the north side of Prince Albert S., Sinclair Cr. and “Trunsky L.” Widespread throughout the Canadian Arctic islands and across the mainland (Porsild and Cody 1980, Korol 1992, Aiken et al. 2007, Hay and Payette 2013, Saarela et al. 2013, Saarela et al. 2017b).

**NORTHWEST TERRITORIES. Boot Inl.**: *Gillespie et al. 9667* (ALA, CAN, MT, O). **Kuujjua R.**: *Gillespie et al. 9779* (ALA, ari, CAN, MT, O, UBC), *9979* (ALA, CAN, O). **Minto Inl. (head)**: *Edlund 161* (CAN), *Gillespie et al. 10013* (ALA, ari, CAN, MT, O), *9505* (CAN). **Natkusiak P.**: *Edlund 79* (CAN). **Prince Albert S. (N)**: *Oldenburg 46-2272* (CAN). **Shaler Mts.**: *Edlund 542* (CAN). **Ulukhaktok**: *Bliss s.n.* (ALTA), *Edlund 356*, *477*, *715* (CAN), *Oldenburg 42-85*, *45-1599* (CAN), *Porsild 17265* (CAN), *Saarela & Bull 1478* (CAN). **NUNAVUT. Albert Edward B.**: *Ponomarenko VI-335A* (CAN). **Anderson B.**: *Edlund & Argus 12711* (CAN). **Cambridge Bay**: *Oldenburg 44*-*927* (CAN), *Polunin s.n.* (CAN, 2 sheets), *Stephens 1042* (CAN, KSTC). **Collinson P.**: *Edlund & Argus 12765* (CAN). **Ferguson L. [Tahiryuaq**]: *Hainault 2043* (DAO). **Greely Haven**: *Fortier 93* (CAN). **Greiner L.**: *Ponomarenko VI-274*, *VI-279C*, *VI-298*, *VI-298A* (CAN). **Hadley B.**: *Edlund 112*, *s.n.* (CAN). **Johansen B.**: *Gillespie et al. 8172* (ALA, ALTA, BABY, CAN, MT, O, UBC, US), *8027* (ALA, CAN, O). **Ovayok TP**: *Gould s.n.* (ALA). **Murray Pt.**: *Gillespie et al. 8206* (ALA, CAN, O). **Namaycush L.**: *Edlund & Roncato-Spencer 56* (CAN). **Sinclair Cr.**: *Gillespie et al. 8230* (ALA, CAN, O). **Storkerson P.**: *Edlund 176* (CAN). “**Trunsky L.**”: *Bennett et al. 14-0392* (BABY, CAN).

***Luzula wahlenbergii*** Rupr., Fig. 10H–Wahlenberg’s woodrush | Circumpolar-alpine

Known from a single collection we made in 2008 at Johansen B.; see Gillespie et al. (2015). Elsewhere in Nunavut recorded from southern Baffin I. and numerous mainland sites (Porsild and Cody 1980, Aiken et al. 2007).

**NUNAVUT. Johansen B.**: *Gillespie et al. 8170* (CAN).

### Cyperaceae [2/33/35]

**Key to Cyperaceae [adapted from Ball et al. (2002)]**

**Table:** 

1	Flowers and achenes partially to completely enclosed in scalelike structure (perigynium); perigynium in axil of scale; flowers unisexual; perianth absent	*** Carex ***
–	Flowers and achenes not enclosed in scalelike perigynium; flowers in axil of scale; flowers usually bisexual, sometimes some, rarely all, flowers unisexual; perianth persistent, of 10–25 smooth, hairlike bristles	*** Eriophorum ***

### *Carex* L. [26/27]

**Key to *Carex* [adapted from Porsild and Cody (1980), Ball and Reznicek (2002), Ball (2002) and Toivonen (2002)]**

**Table:** 

1	Margins of perigynium open; terminal and distal spikelets usually 1-flowered, staminate; proximal spikelets 1-flowered and pistillate, or 2–4-flowered and bisexual with 1 pistillate flower proximally and 1–3 staminate flowers distally	**2**
–	Margins of perigynium fused; all spikelets 1-flowered	**4**
2	Inflorescences compound; basal leaf sheaths dull, base of blade usually persistent	***C. simpliciuscula*** subsp. ***subholarctica***
–	Inflorescences simple; basal leaf sheaths somewhat glossy, bladeless	**3**
3	Perigynia 2–3.5 mm, margins free to base; scales ovate, 2–3.5 mm; midvein distinct almost to tip; anthers 1–1.5 mm; achenes 2–2.8 mm	***C. myosuroides***
–	Perigynia 3.5–5.5 mm, margins connate near base; scales obovate-circular, 3.5–5 mm; midvein distinct only near base; anthers 2–3 mm; achenes 2.6–4 mm	***C. borealipolaris***
4	Spikes solitary	**5**
–	Spikes compound	**9**
5	Pistillate scales early deciduous; perigynia deflexed at maturity	***C. microglochin***
–	Pistillate scales persistent; perigynia not deflexed at maturity	**6**
6	Stigmas 2; plants densely cespitose	**7**
–	Stigmas 3; plants cespitose or loosely cespitose	**8**
7	Spike gynaecandrous (staminate flowers proximal, pistillate flowers distal); perigynia 1.5–2 mm; plants of seashores	***C. ursina***
–	Spike androgynous (pistillate flowers proximal, staminate flowers distal); perigynia 3–5 mm; plants of dry, exposed tundra	***C. nardina***
8	Plants dioecious, rarely monoecious; rhizomes short, sometimes inconspicuous; perigynia hairy	***C. scirpoidea*** subsp. ***scirpoidea***
–	Plants monoecious, spike androgynous; rhizomes long; perigynia glabrous	***C. rupestris***
9	Spikes all bisexual and sessile	**10**
–	Spikes mostly unisexual, the terminal one staminate or bisexual	**13**
10	Some or all spikes androgynous	**11**
–	Some or all spikes gynaecandrous	**12**
11	Plants rhizomatous, stolons lacking; perigynium beak 0.5–1 mm	***C. maritima***
–	Plants rhizomatous and long-stoloniferous (vegetative stems ascending to erect when young, becoming prostrate stolons at maturity); perigynium beak 0.3–0.6 mm	***C. chordorrhiza***
12	Plants loosely cespitose; culms erect, 10–15(–30) cm; spikes 2–3(–4); lateral spikes gynecandrous, containing 3–8 perigynia, oblong-clavate; perigynia elliptic, green-white proximally, pale brown distally, often brown in age; beak indistinct	***C. marina***
–	Plant densely cespitose; culms often arching, weak, 10–25 cm; spikes 2–4; lateral spikes pistillate, containing 5–10(–15) perigynia, oblong-linear; perigynia broadly elliptic-obovate to lanceolate, light to pale brown, often gray-brown at maturity; beak short	***C. glareosa***
13	Stigmas 2	**14**
–	Stigmas 3	**19**
14	Terminal spike gynecandrous	***C. bicolor***
–	Terminal spike staminate	**15**
15	Perigynia somewhat glossy, glabrous, more or less inflated, beak distinct; lateral spikes usually pendant	***C. saxatilis***
–	Perigynia dull, usually papillose, sometimes glabrous, not inflated, beak indistinct; lateral spikes erect	**16**
16	Pistillate scales with a prominent, scabrous awn on at least the proximal scales; leaf blades involute, 1–2 mm wide	***C. subspathacea***
–	Pistillate scales with apex acute, acuminate, or mucronate, lacking a prominent, scabrous awn; leaf blades not involute, the widest > 2 mm wide	**17**
17	Proximal bract longer than inflorescence (usually at least 1.5× as long)	***C. aquatilis*** subsp. ***stans***
–	Proximal bract shorter than or equal to inflorescence (***C. bigelowii***)	**18**
18	Perigynia green, spotted purple-black on apical 1/2, minutely papillose; stipe 0.15–0.45 mm; proximal pistillate spike loosely flowered, base often attenuate, less often cuneate	***C. bigelowii*** subsp. ***bigelowii***
–	Perigynia green, often white at maturity, uniformly purple-brown on apical 1/2, strongly or minutely papillose; stipe 0–0.15 mm; proximal pistillate spike densely flowered, base cuneate, less often attenuate	**C. bigelowii subsp. lugens**
19	Terminal spike gynaecandrous	**20**
–	Terminal spike staminate	**21**
20	Perigynia lanceolate, 3.3–5.5 mm, margins ciliate; pistillate scales ovate, 2.8–4.2 mm; staminate scales oblong-obovate or obovate, 3–5 mm; achenes 1.5–2 × 0.9–1 mm	**C. fuliginosa subsp. misandra**
–	Perigynia lanceolate to ovate-lanceolate, 1.5–3.3 mm, margins entire or serrulate; pistillate scales obovate or obovate-circular, 1.6–2.1 mm; staminate scales oblong-ovate 2–2.8 mm; achenes 1.1–1.4 × 0.6–0.9 mm	***C. krausei***
21	Inflorescence capitate, the lateral spikes short-peduncled	***C. glacialis***
–	Inflorescence not capitate	**22**
22	Pistillate spikes sessile or nearly sessile	***C. membranacea***
–	Pistillate spikes on peduncles as long or longer than the spikes	**23**
23	Plants tufted	**24**
–	Plants rhizomatous	**25**
24	Pistillate scales black with pale midvein, ovate or oblong-ovate, 3–4.8 × 0.9–1.6 mm; lateral spikes 4–7 mm wide; terminal spike 6–15 × 2–5 mm, usually over-topping lateral spikes, sometimes overlapping some of them	***C. atrofusca***
–	Pistillate scales medium to dark brown with pale midvein, ovate, obovate or obovate-circular, 2.3–3.5 × 0.8–1.2 mm; lateral spikes 3–4 mm wide; terminal spike 4–10 × 0.7–1.4 mm, level with or over-topped by some of the lateral spikes	**C. capillaris subsp. fuscidula**
25	Lateral spikes ± erect; proximal perigynia loosely arranged; perigynium beak distinct, (0.4–)0.6–1.8(–2.2) mm	***C. vaginata***
–	Lateral spikes drooping, at least the lowermost ones; proximal perigynia densely arranged; perigynium beak absent or indistinct, to 0.5 mm	**26**
26	Perigynia pale green; proximal bracts of inflorescence 0.5–2 cm; spikes 2–4	***C. rariflora***
–	Perigynia pale yellow proximally, brown or black distally; proximal bracts of inflorescences 0.5–14 cm; spikes 3–8	**C. petricosa subsp. petricosa**

**Figure 12.**
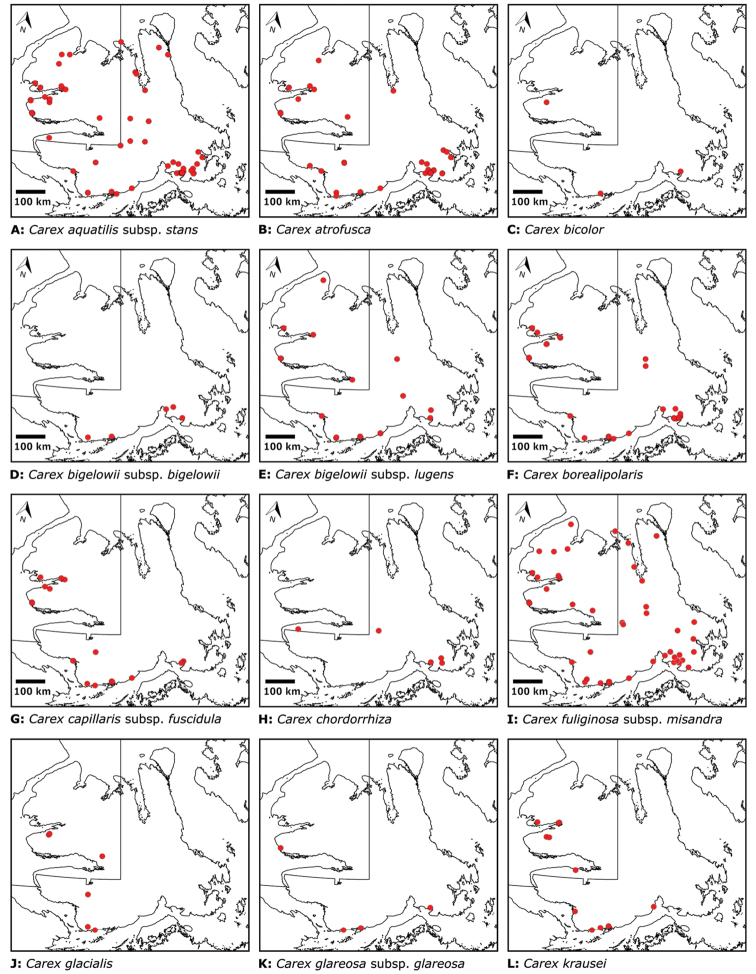
Species distribution maps. Cyperaceae: **A**Carex aquatilis subsp. stans**B***Carex atrofusca***C***Carex bicolor***D**Carex bigelowii subsp. bigelowii**E**Carex bigelowii subsp. lugens**F***Carex borealipolaris***G**Carex capillaris subsp. fuscidula**H***Carex chordorrhiza***I**Carex fuliginosa subsp. misandra**J***Carex glacialis***K**Carex glareosa subsp. glareosa**L***Carex krausei*.

**Figure 13.**
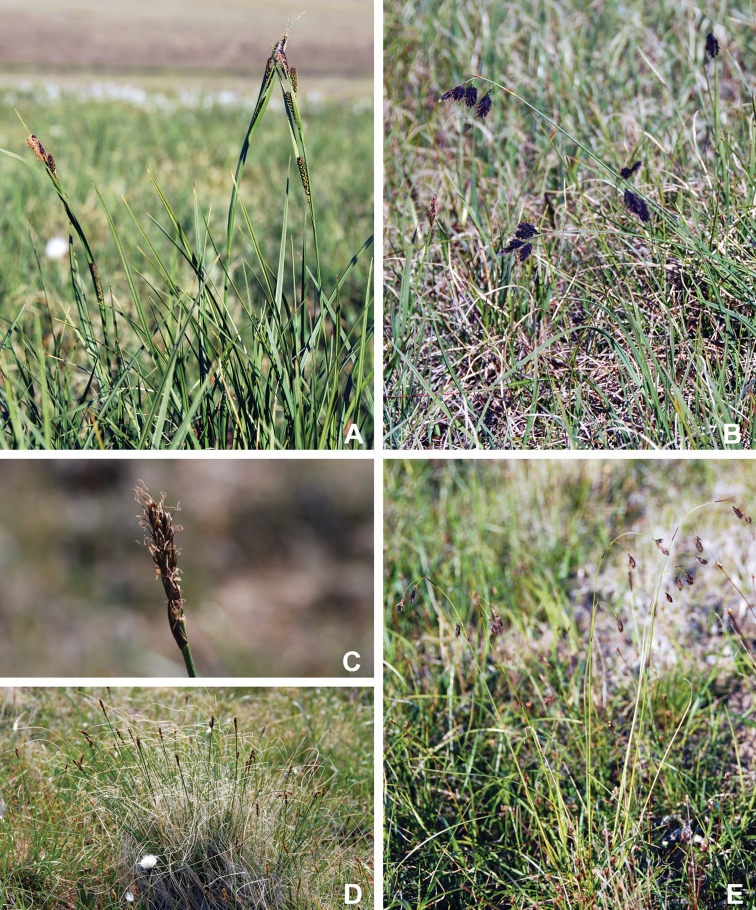
**A**Carex aquatilis subsp. stans habit, 18 July 2010 **B***Carex atrofusca* habit, head of Minto Inlet, 25 July 2010 **C***Carex borealipolaris* inflorescence, *Gillespie et al. 9900***D***Carex borealipolaris* habit, *Gillespie et al. 9900***E**Carex fuliginosa subsp. misandra habit, Kuujjua River, NT, 18 July 2010. Photos by J.M. Saarela.

***Carex aquatilis*** subsp. ***stans*** (Drejer) Hultén (C. aquatilis var. minor Boott, *C. stans* Drejer), Figs 12A, 13A–Arctic water sedge | Circumpolar-alpine

Previously recorded from Cambridge Bay, C. Wollaston, Hadley B., Kuujjua R., Mt. Bumpus, Namaycush L., Natkusiak P., north and southeast of the head of Prince Albert S., Richard Collinson Inl., Storkerson P., Tahiryuaq and Ulukhaktok (Porsild 1955, 1957, 1964, Aiken et al. 2007). Thannheiser et al. (2001) additionally recorded it from Johansen B. (conf.), Richardson I., Surrey L. and Wellington B. Newly recorded from Albert Edward B., Greiner L., Oterkvik Pt., Murray Pt., an inland site on Prince Albert P., Read I., Sinclair Cr., Walker B. and Wollaston P. Widespread throughout the Canadian Arctic islands and on the mainland (Porsild and Cody 1980, Cody et al. 1989, Korol 1992, Aiken et al. 2007, Saarela et al. 2013).

**NORTHWEST TERRITORIES. Boot Inl.**: *Dutilly 18706* (US), *Gillespie et al. 9562* (ALA, CAN, O). **C. Wollaston**: *Edlund 42* (CAN), *163* (CAN). **Kuujjua R.**: *Edlund 634* (CAN), *Gillespie et al. 9783* (ALA, CAN, MT, WIN), *9945* (ALA, ari, CAN, O, WIN), *9905* (ALA, CAN, MT, O). **Minto Inl. (head)**: *Edlund 135*, *148*, *171* (CAN), *Gillespie et al. 9492* (CAN, O), *10144* (ALA, CAN, O), *Porsild 17820* (CAN). **Prince Albert P.**: *Oldenburg 54*-*656* (MIN). **Richard Collinson Inl.**: *Edlund 132*, *689* (CAN). **Tahiryuaq**: *Edlund 390* (CAN). **Ulukhaktok**: *Bandringa 344* (CAN, UBC), *Dutilly 16847* (US), *Edlund 360*, *492*, *828*, *907* (CAN), *Oldenburg 45-1578* (CAN), *Porsild 17256* (CAN), *Svoboda 745029* (TRTE, UBC). **Walker B.**: *Oldenburg 45-1432*, *45-1436* (CAN). **Wollaston Pen.**: *Oldenburg 54-497* (MIN, UBC). **NUNAVUT. Albert Edward B.**: *Ponomarenko VI-252*, *VI-267* (CAN). **Cambridge Bay**: *Bennett et al. 14-0366* (MICH, UBC), *13-0183* (chars, DAO), *Calder et al. 24189* (DAO), *Consaul & Gillespie 1108* (CAN), *Edlund & Argus 12618* (CAN, TRTE), *Fortier 17* (CAN), *Gillespie et al. 8404* (ALA, BABY, CAN, MT, O), *Oldenburg 44*-*964* (CAN), *Polunin s.n.* (CAN, 2 sheets), *Ponomarenko VI-086A*, *VI-087* (CAN), *Porsild 17463* (CAN), *Smith & Sweatman 34A* (DAO), *Stephens 965* (ID, KANU), *956*, *1024*, *1198* (KANU), *1126* (KANU, KSTC), *958*, *1127*, *1197* (CAN, KANU), *1058* (CAN). **Ferguson L. [Tahiryuaq**]: *Bennett et al. 14-0417* (BABY, DAO, MICH), *Hainault 1929* (DAO), *Jones & Hainault 1866* (DAO). **Greiner L.**: *Ponomarenko VI-124C*, *VI-136*, *VI-149*, *VI-158*, *VI-190*, *VI-203C*, *VI-240*, *VI-329c* (CAN). **Hadley B.**: *Edlund 35*, *104*, *425* (CAN). **Johansen B.**: *Gillespie et al. 7916* (ALA, CAN, MT, O), *7906* (CAN, O). **Mt. Bumpus**: *Edlund 203*, *216*, *248* (CAN). **Murray Pt.**: *Gillespie et al. 8190* (ALA, BABY, CAN, MT, O). **Namaycush L.**: *Edlund 34*, *76*, *137*, *138* (CAN). **Oterkvik Pt.**: *Gillespie et al. 7479* (ALA, CAN, O), *7545* (CAN, O). **Prince Albert S. (head)**: *Edlund 100*, *24*, *93* (CAN). **Read I.**: *Oldenburg 43*-*1007b* (CAN). **Sinclair Cr.**: *Gillespie et al. 8299* (ALA, ALTA, BABY, CAN, MT, O, UBC, US). **Storkerson P.**: *Edlund 183*, *281* (CAN). **Tahoe L.**: *Porsild 17450* (CAN). **Tuktu R.**: *Gould s.n.* (ALA). **Washburn L.**: *Edlund & Argus 12799* (CAN, TRTE).

***Care***x ***atrofusca*** Schkuhr, Figs 12B, 13B–Dark brown sedge | Circumpolar–alpine

Previously recorded from Cambridge Bay, Ferguson L., Jonnessee L., the head of Minto Inl., Mt. Pelly, Tahiryuaq and Ulukhaktok (Porsild 1955, 1957, 1964, Porsild and Cody 1980, Aiken et al. 2007). Thannheiser et al. (2001) additionally recorded it from Johansen B. (conf.), Richardson I., Surrey L., Wellington B. and Mt. Pelly (conf.). Newly recorded from Albert Edward B., Boot Inl., Falaise B., Hadley B., Kuujjua R., Mt. Bumpus, Oterkvik Pt., Read I. and Sinclair Cr. Elsewhere in the Canadian Arctic recorded from Baffin, Banks, Coats, Ellesmere, Devon and Southampton islands and across the mainland (Porsild and Cody 1980, Korol 1992, Aiken et al. 2007, Saarela et al. 2013).

**NORTHWEST TERRITORIES. Boot Inl.**: *Dutilly 18705a* (US), *Gillespie et al. 9641* (ALA, CAN, MT, WIN). **Minto Inl. (head)**: *Gillespie et al. 9487* (CAN, O), *Porsild 17364* (CAN). **Kuujjua R.**: *Gillespie et al. 9775* (ALA, ari, CAN, MT, O, UBC). **Richard Collinson Inl.**: *Edlund 684* (CAN). **Tahiryuaq**: *Edlund 405* (CAN). **Ulukhaktok**: *Edlund 312*, *490* (CAN, ID), *725* (CAN), *Porsild 17247* (CAN). **NUNAVUT. Albert Edward B.**: *Ponomarenko VI-254A*, *VI-266* (CAN). **Cambridge Bay**: *Bennett et al. 13-0169* (BABY, chars, od, UBC), *Calder et al. 24156* (DAO), *Edlund & Argus 12670* (CAN, TRTE), *Gillespie et al. 8409* (ALA, BABY, CAN, MT, O), *Gould s.n.* (ALA), *Polunin s.n.* (CAN, 2 sheets), *Ponomarenko VI-080C*, *VI-085*, *VI-089A* (CAN), *Porsild 21600* (CAN), *Stephens 1039* (KANU, KSTC), *968* (CAN, KANU), *1057*, *1128* (CAN, KANU, KSTC). **Falaise B.**: *Eriksen et al. 951* (ALA). **Ferguson L. [Tahiryuaq**]: *Hainault 2057* (DAO), *Hainault & Jones 1869* (DAO). **Greiner L.**: *Ponomarenko VI-100C*, *VI-100D*, *VI-100E*, *VI-130*, *VI-135a*, *VI-154A* (CAN). **Hadley B.**: *Edlund 146* (CAN). **Jonnessee L.**: *Edlund & Argus 12779*, *12780* (mixed with *C. saxatilis*), *12782* (CAN). **Johansen B.**: *Gillespie et al. 7947* (ALA, BABY, CAN, MT, O). **Mt. Bumpus**: *Edlund 161*, *225* (CAN). **Ovayok TP**: *Gould s.n.* (ALA). **Oterkvik Pt.**: *Gillespie et al. 7478* (ALA, CAN, MT, O), *Gillespie et al. 7612* (ALA, CAN, O). **Read I.**: *Ross 10* (ALTA). **Sinclair Cr.**: *Gillespie et al. 8305* (ALA, BABY, CAN, MT, O, US).

***Carex bicolor*** Bellardi ex All., Fig. 12C–Bicoloured sedge | Circumpolar–alpine

Newly reported from Victoria I. and the western Canadian Arctic Archipelago based on a collection we made in 2008 in the Johansen B. area; see Gillespie et al. (2015). Two additional records from Victoria I. are newly reported here: one from Cambridge Bay collected in 2013 along the road to Ovayok (Mount Pelly), where it was growing in a *Carex aquatilis* fen surrounded by *Dryas integrifolia/Salix arctica* tundra with *Salix richardsonii*, *Saxifraga hirculus* and *Carex simpliciuscula*, and one from the Kuujjua R. area, collected in 1982. The latter specimen had been misidentified as *Carex rariflora*. Elsewhere in the Canadian Arctic recorded from southern Baffin, Coats and Nottingham islands and a few sites on mainland Nunavut and Northwest Territories (Porsild 1943, Porsild and Cody 1980, Cody et al. 1989, Gould and Walker 1997, Aiken et al. 2007, Saarela et al. 2013, Gillespie et al. 2015, Saarela et al. 2017b).

**NORTHWEST TERRITORIES. Kuujjua R.**: *Edlund 646* (CAN). **NUNAVUT. Cambridge Bay**: *Bennett et al. 13-0199* (BABY, CAN). **Johansen B.**: *Gillespie et al. 8118* (CAN).

***Carex bigelowii*** Torr. ex Schwein. subsp. ***bigelowii***, Fig. 12D–Bigelow’s sedge | North American–amphi-Atlantic

Newly recorded for Victoria Island, known from Cambridge Bay, Ferguson L., Johansen B. and Oterkvik Pt. The Ferguson L. records were not previously determined to subspecies. This is a primarily eastern Arctic taxon recorded elsewhere in the Canadian Arctic from numerous mainland sites as well as Baffin, Devon, Ellesmere and Southampton islands (Porsild and Cody 1980, Cody et al. 1989, Korol 1992, Aiken et al. 2007). It reaches its known western limit in the Canadian Arctic Archipelago in the study area and on the mainland along the Coppermine R. (Saarela et al. 2017b). Thannheiser et al. (2001) recorded *C. bigelowii* from six sites and *C. lugens* from one (Richardson I.). We find the former taxon to be rare on Victoria I. and the latter to be common. It is possible that the taxon concepts in that paper were inadvertently mixed up.

**NUNAVUT. Cambridge Bay**: *Polunin s.n.* (CAN). **Ferguson L. [Tahiryuaq**]: *Hainault 2077*, *2134* (DAO). **Johansen B.**: *Gillespie et al. 7876* (ALA, CAN, O). **Oterkvik Pt.**: *Gillespie et al. 7799* (ALA, CAN, MT, O).

***Carex bigelowii*** subsp. ***lugens*** (Holm) T.V.Egorova (*C. consimilis* Holm, *C. lugens* Holm), Fig. 12E–Spruce muskeg sedge | Eurasian–amphi-Beringian

Previously known from Ulukhaktok and the head of Prince Albert S. (Porsild 1955, 1957, 1964, Aiken et al. 2007). Thannheiser et al. (2001) additionally recorded it from Richardson I. Newly recorded from Cambridge Bay, the head of Minto Inl., Johansen B., Mt. Lady Pelly, “Oldenburg L.”, Oterkvik Pt., Read I., Sinclair Cr., Surrey L., Walker B. and Washburn L. Elsewhere in the Canadian Arctic recorded from Banks I. and some mainland sites, reaching its known eastern limit on central mainland Nunavut (Porsild and Cody 1980, Aiken et al. 2007, Saarela et al. 2013, Saarela et al. 2017b). This subspecies, a western Arctic taxon, is more common than subsp. bigelowii on Victoria I.

**NORTHWEST TERRITORIES. Minto Inl. (head)**: *Gillespie et al. 10269* (ALA, ari, CAN, O, UBC, WIN), *10270* (CAN, O), *10282* (CAN, WIN). “**Oldenburg L.**”: *Oldenburg 45-1373* (CAN). **Prince Albert S.**: *Porsild 17436* (CAN). **Ulukhaktok**: *Edlund 910* (CAN), *Oldenburg 42-83*, *42-86*, *45-1561* (CAN). *Porsild 17248* (CAN), *Saarela & Bull 1474* (ALA, CAN, MT, O). **Walker B.**: *Oldenburg 45-1415* (CAN). **NUNAVUT. Cambridge Bay**: *Oldenburg 44-905A*, *44-961* (CAN), *Saarela & Teeter 5284* (CAN). **Johansen B.**: *Gillespie et al. 7952* (ALA, BABY, CAN, MT, O), *8049* (ALA, BABY, CAN, MT, O, US), *7845* (ALA, CAN, O). **Mt. Lady Pelly [Amaaqtuq**]: *Jones & Hainault 34a* (DAO). **Oterkvik Pt.**: *Gillespie et al. 7557* (ALA, CAN, MT, O), *7784* (ALA, CAN, MT, O), *7607* (CAN, O). **Read I.**: *Oldenburg 42-494*, *43-1005*, *43-893*, *43-971* (CAN). **Sinclair Cr.**: *Gillespie et al. 8308* (ALA, ALTA, BABY, CAN, MT, O, UBC, US), *8244* (CAN). **Surrey L.**: *Edlund & Argus 12805* (CAN). **Washburn L.**: *Oldenburg 46-2219* (CAN).

***Carex borealipolaris*** S.R.Zhang (*Kobresia sibirica* (Turcz. ex Ledeb.) Boeckeler, *K. hyperborea* A.E.Porsild), Figs 12F, 13C, D–Siberian bog sedge | Asian (N/C)–amphi-Beringian

Previously recorded from Cambridge Bay, Namaycush L., Ulukhaktok and Walker B. (Porsild 1955, 1957, 1964, Aiken et al. 2007). Thannheiser et al. (2001) additionally recorded it from the head of Minto Inl. (conf.), Richardson I., Johansen B. (conf.), Hadley B. and Mt. Pelly. Newly recorded from Boot Inl., Greiner L., Kuujjua R., Murray Pt., Oterkvik Pt., Read I., Sinclair Cr. and Washburn L. Elsewhere in the Canadian Arctic recorded from scattered sites on mainland Nunavut and Northwest Territories (Porsild and Cody 1980, Aiken et al. 2007, Saarela et al. 2013, Saarela et al. 2017b).

**NORTHWEST TERRITORIES. Boot Inl.**: *Gillespie et al. 9557* (ALA, CAN, O, WIN). **Kuujjua R.**: *Gillespie et al. 9729* (ALA, ari, CAN, MT, O), *9782* (ALA, CAN, MT, O), *9900* (ALA, ari, CAN, MT, O, UBC, US, WIN). **Minto Inl. (head)**: *Gillespie et al. 10284* (CAN, O), *10297* (ALA, CAN, O, WIN). **Ulukhaktok**: *Edlund 859* (CAN), *Porsild 17260* (ALTA, CAN), *Saarela & Bull 1495* (ALA, CAN, O). **Walker B.**: *Porsild 17487* (CAN). **NUNAVUT. Cambridge Bay**: *Bennett et al. 13-0188* (BABY, chars, od), *13-0611* (CAN), *Consaul & Gillespie 1127* (CAN), *Dutilly & Duman 37110* (US), *Edlund & Argus 12688* (CAN), *Gillespie et al. 8371* (ALA, BABY, CAN, MT, O), *Oldenburg 44-920* (CAN), *Polunin s.n.* (CAN, 2 sheets), *Ponomarenko VI-086B*, *VI-090A* (CAN), *Porsild 21608* (CAN). **Ferguson L. [Tahiryuaq**]: *Hainault 2135* (DAO), *Jones & Hainault 1863A* (DAO). **Greiner L.**: *Ponomarenko VI-302* (CAN). **Johansen B.**: *Gillespie et al. 7950* (ALA, BABY, CAN, MT, O, UBC, US), *7840* (CAN, O). **Murray Pt.**: *Gillespie et al. 8205* (CAN, O). **Namaycush L.**: *Edlund & Roncato-Spencer 109* (CAN). **Oterkvik Pt.**: *Gillespie et al.* 7556 (ALA, BABY, CAN, MT, O, US, WIN). **Read I.**: *Oldenburg 43-986* (CAN). **Sinclair Cr.**: *Gillespie et al.* 8229 (ALA, CAN, O). **Washburn L.**: *Oldenburg 46-2221* (CAN).

***Carex capillaris*** subsp. ***fuscidula*** (V.I.Krecz. ex T.V.Egorova) Á.Löve & D.Löve, Fig. 12G–Hair sedge | Circumpolar-alpine

Previously recorded from Cambridge Bay, Read I., Tahoe L. (Porsild obs.), Ulukhaktok (Porsild obs., conf.) and Walker B. (Porsild obs.) (Porsild 1955, 1957, 1964, Porsild and Cody 1980, Aiken et al. 2007). Thannheiser et al. (2001) recorded C. capillaris subsp. capillaris (probably including both subsp. fuscidula and *C. krausei*) from Johansen B. (conf.) and the head of Minto Inl. (conf.). Newly recorded from Boot Inl., Kuujjua R., Mt. Bumpus, Oterkvik Pt. and Sinclair Cr. Elsewhere in the Canadian Arctic recorded from Axel Heiberg, Baffin, Banks, Ellesmere, Somerset and Southampton islands, scattered sites on mainland Nunavut and Northwest Territories, and northern Quebec and Labrador (Porsild and Cody 1980, Aiken et al. 2007, Saarela et al. 2013, Sokoloff 2015, Saarela et al. 2017b).

**NORTHWEST TERRITORIES. Boot Inl.**: *Gillespie et al. 9675* (CAN). **Kuujjua R.**: *Gillespie et al. 9774* (CAN, MT, O), *9946* (ALA, CAN, MT, WIN). **Minto Inl. (head)**: *Gillespie et al. 10240* (ALA, CAN, MT, O), *10281* (CAN, O). **Ulukhaktok**: *Edlund 313*, *772*, *815*, *896* (CAN). **NUNAVUT. Cambridge Bay**: *Bennett et al. 13-0184* (BABY, chars), *Polunin s.n.* (CAN), *Stephens 1191* (CAN). **Johansen B.**: *Gillespie et al. 7844* (CAN), *7987* (CAN). **Mt. Bumpus**: *Edlund 284* (CAN). **Oterkvik Pt.**: *Gillespie et al. 7571* (ALA, CAN, O), *7685* (CAN, O). **Sinclair Cr.**: *Gillespie et al. 8306a* (CAN). **Read I.**: *Oldenburg 43-888*, *43-995* (CAN), *Porsild 17187* (CAN).

***Carex chordorrhiza*** L.f., Fig. 12H–Creeping sedge | Circumboreal-polar

Previously recorded from Tahoe L., where collected in 1949, and a lake edge 1.6 km northeast of Cambridge Bay (Porsild 1955, 1957, 1964, Aiken et al. 2007). Newly recorded from Greiner L. and the vicinity of a pingo on Wollaston P. Targeted efforts were made by B.A. Bennett to rediscover this species near Cambridge Bay during 2013 and 2014 fieldwork, but it was not found. Elsewhere in the Canadian Arctic recorded from southern Baffin I. and mainland sites (Porsild and Cody 1980, Cody et al. 1989, Aiken et al. 2007, Saarela et al. 2013, Bennett 2015, Saarela et al. 2017b). The Victoria I. populations mark the northern edge of the species’ range.

**NORTHWEST TERRITORIES. Wollaston P.**: *Oldenburg 54-504A* (MIN). **NUNAVUT. Cambridge Bay**: *Stephens 1279* (CAN). **Greiner L.**: *Ponomarenko VI-122*, *VI-178A* (CAN). **Tahoe L.**: *Porsild 17449* (CAN).

***Carex fuliginosa*** subsp. ***misandra*** (R.Br.) Nyman (*C. misandra* R.Br.), Figs 12I, 13E–Short leaf sedge | Circumpolar-alpine

Previously recorded from Anderson B., Byron B., Cambridge Bay, the head of Minto Inl., Natkusiak P., Namaycush L., N of a large lake in the Ekalluk River system about 90 km NNE of Cambridge Bay, Mt. Bumpus, Storkerson P., Tahiryuaq, Ulukhaktok and Walker B. (Porsild 1955, 1957, 1964, Porsild and Cody 1980, Aiken et al. 2007). Thannheiser et al. (2001) additionally recorded it at Johansen B. (conf.), Mt. Pelly. (conf.), Richardson I., Surrey L. and Wellington B. Newly recorded from “30-Mile Cr.”, Albert Edward B., Boot Inl., Ferguson L., Greiner L., Kuujjua R., “Oldenburg L.”, Oterkvik Pt., the north side and inland from the head of Prince Albert S., Read I., Richard Collinson Inl., Sinclair Cr., Washburn L. and a site 90 km NNE of Cambridge Bay. Widespread throughout the Canadian Arctic (Porsild and Cody 1980, Cody et al. 1989, Korol 1992, Aiken et al. 2007, Saarela et al. 2013, Saarela et al. 2017b).

**NORTHWEST TERRITORIES. Boot Inl.**: *Gillespie et al. 9668* (ari, CAN, MT, O), *9669* (CAN), *9676* (CAN, O). **Kuujjua R.**: *Gillespie et al. 9726* (CAN, O). **Minto Inl. (head)**: *Edlund 91* (CAN), *Gillespie et al. 9488* (ALA, CAN, MT, O). **Natkusiak P.**: *Edlund 78* (CAN). “**Oldenburg L.**”: *Oldenburg 45-1372* (CAN). **Prince Albert P.**: *Oldenburg 54-239*, *54*-*663* (UBC). **Prince Albert S. (head)**: *Weerstra 32*, *33* (DAO). **Prince Albert S. (N)**: *Oldenburg 46-2274* (CAN). **Richard Collinson Inl.**: *Edlund 685* (CAN). **Tahiryuaq**: *Edlund 405* (CAN). **Ulukhaktok**: *Bliss s.n.* (ALTA), *Edlund 487* (CAN, ID), *728* (CAN), *Oldenburg 42-84*, *45-1570*, *45-1574* (CAN), *Porsild 17251*, *17252* (CAN), *Saarela & Bull 1494* (ALA, CAN, MT, O, WIN). **Walker B.**: *Oldenburg 45-1417*, *45-1419*, *45-1422* (CAN), *Porsild 17483*, *17484* (CAN). **NUNAVUT. “30-Mile Cr.**”: *Bennett et al. 14-0354* (UBC, CAN). **Albert Edward B.**: *Ponomarenko VI-255* (CAN). **Anderson B.**: *Edlund & Argus 12718* (CAN). **Byron B.**: *Dushenko 8* (UVIC). **Cambridge Bay**: *Bennett et al. 14-0321* (UBC), *13-0185* (BABY, chars, od), *Calder et al. 24167*, *24168* (DAO), *Gillespie et al. 8365* (ALA, BABY, CAN, MT, O, UBC, US), *Oldenburg 44-911*, *44-913*, *44-966* (CAN), *Polunin s.n.* (CAN, 2 sheets), *Ponomarenko VI-080D*, *VI-086E*, *VI-095* (CAN), *Porsild 21602* (DAO), *Stephens 1185* (KANU, KSTC), *997* (KSTC), *1043*, *1044* (CAN, KANU, KSTC), *1188* (CAN, KANU), *Thomson s.n.* (WIS), *Washburn 4*, *6*, *27* (CAN). **Ekalluk R.**: *Edlund & Argus 12741* (CAN). **Eastern Victoria I.**: *Lee & Kittle s.n.* (CAN). **Ferguson L. [Tahiryuaq**]: *Bennett et al. 14-0416* (CAN, DAO, chars, od). **Greiner L.**: *Ponomarenko VI-035A*, *VI-101b*, *VI-121*, *VI-131A*, *VI-151*, *VI-177*, *VI-238B* (CAN). **Hadley B.**: *Edlund 103*, *s.n.* (CAN). **Johansen B.**: *Gillespie et al. 7943* (ALA, BABY, CAN, MT, O, US), *8018* (ALA, BABY, CAN, MT, O), *8171* (ALA, CAN, O). **Mt. Bumpus**: *Edlund 224*, *253* (CAN). **Ovayok TP**: *Bennett & Sullivan 13-0288* (BABY, chars), *Gillespie et al. 8425* (CAN, O), *Stephens 1045* (KANU), *1129* (ID, KANU). **Namaycush L.**: *Edlund 133* (CAN), *Edlund & Roncato-Spencer 105* (CAN). **Natkusiak P.**: *Edlund 311* (CAN). **Oterkvik Pt.**: *Gillespie et al. 7606* (ALA, CAN, O), *7643* (ALA, CAN, O). **Prince Albert S.**: *Weerstra 27* (DAO). **Read I.**: *Oldenburg 43-1011*, *43-943* (CAN). **Sinclair Cr.**: *Gillespie et al. 8306b* (CAN, O). **Storkerson P.**: *Edlund 187* (CAN). **Washburn L.**: *Oldenburg 46-2227* (CAN).

***Carex glacialis*** Mack., Fig. 12J–Glacier sedge | Circumpolar–alpine

Previously known from near the head of Prince Albert S. (mapped erroneously in Aiken et al. (2007) on north-central Victoria I.) and Ulukhaktok (Porsild obs.) (Porsild 1955, 1957, 1964, Porsild and Cody 1980, Aiken et al. 2007). Newly recorded from Kuujjua R., Colville Mts. and Oterkvik Pt. At one site in the Kuujjua R. area, the species was common in dry, almost bare soil among bedrock-dominated tundra growing with *Deschampsia brevifolia*, *Dryas integrifolia* and *Salix arctica*, in an area dominated by sand dunes. Elsewhere in the Canadian Arctic recorded from Axel Heiberg, Baffin, Coats, Ellesmere and Nottingham islands, and numerous mainland sites (Porsild 1964, Porsild and Cody 1980, Aiken et al. 2007, Saarela et al. 2017b).

**NORTHWEST TERRITORIES. Kuujjua R.**: *Gillespie et al. 9909* (ALA, CAN, WIN), *9978* (CAN, O). **Prince Albert S.**: *Porsild 17365* (CAN). **NUNAVUT. Colville Mts.**: *Gillespie et al. 7763* (CAN). **Oterkvik Pt.**: *Gillespie et al. 7608* (ALA, BABY, CAN, MT, O), *7675* (ALA, CAN, O).

***Carex glareosa*** Wahlenb. subsp. ***glareosa*** (C. glareosa var. amphigena Fernald), Fig. 12K–Gravel sedge | Circumpolar

Previously recorded from Ulukhaktok (Porsild 1955, 1957, 1964, Porsild and Cody 1980, Aiken et al. 2007). Thannheiser et al. (2001) additionally recorded it from Johansen B. (conf.). Newly recorded from Cambridge Bay (a collection previously determined by Porsild as *C. amblyorhyncha* (=*C. marina*)), where gathered in 1949 near the Hudson’s Bay Post and not recorded in the area since, and Oterkvik Pt. Elsewhere in the Canadian Arctic known from Baffin, Coats, Devon, Ellesmere and Southampton (CAN 583988) islands and mainland sites (Porsild and Cody 1980, Cody et al. 1984a, Cody et al. 1989, Korol 1992, Aiken et al. 2007, Saarela et al. 2013, Saarela et al. 2017b). This is a seashore species that, when present, is generally conspicuous.

**NORTHWEST TERRITORIES. Ulukhaktok**: *Porsild 17249* (ALTA, CAN). **NUNAVUT. Cambridge Bay**: *Porsild 17464* (CAN). **Johansen B.**: *Gillespie et al. 8020* (ALA, CAN, O). **Oterkvik Pt.**: *Gillespie et al. 7683* (CAN, O).

**Figure 14.**
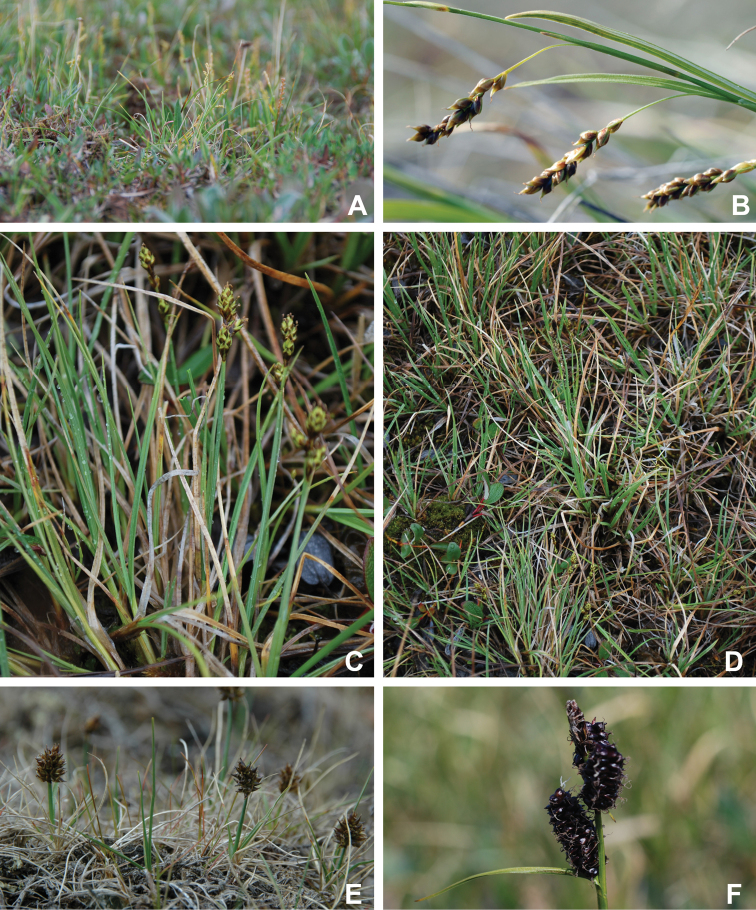
**A***Carex krausei* habit, *Gillespie et al. 9977***B***Carex krausei* inflorescence, *Gillespie et al. 8114***C***Carex marina* habit **D***Carex marina* habitat **E***Carex maritima* habit, Johansen Bay, NU, 18 July 2008 **F***Carex membranacea* inflorescence. Photos **A**, **F** by J.M. Saarela **C**, **D** by B.A. Bennett and **B**, **E** by R.D. Bull.

***Carex krausei*** Boeckeler (C. capillaris subsp. robustior (Lange) Böcher), Figs 12L, 14A, B–Krause’s sedge | Circumpolar–alpine

Previously recorded from Ulukhaktok and the south side of Prince Albert S. (Aiken et al. 2007). Some of the reports of C. capillaris subsp. capillaris by Thannheiser et al. (2001) may be this species; vouchers require confirmation. Newly recorded from “30-Mile Cr.”, Boot Inl., Clouston B., Kuujjua R., the head of Minto Inl., Johansen B. and Oterkvik Pt. Elsewhere in the Canadian Arctic recorded from Axel Heiberg, Baffin, Banks, Coats, Ellesmere and Southampton islands and mainland sites (Porsild and Cody 1980, Gould and Walker 1997, Aiken et al. 2007, Saarela et al. 2013, Saarela et al. 2017b). The Working Group on General Status of NWT Species (2016) recorded the status of this species in Northwest Territories as “Undetermined”. The eight populations from the Northwest Territories portion of Victoria I. reported here, seven reported from Tuktut Nogait National Park and vicinity (Saarela et al. 2013) and at least nine other records of this species in the National Herbarium of Canada from mainland Northwest Territories, which have been revised (by J.M. Saarela) following current taxonomy, should be sufficient evidence to revise the status to Secure in the next version.

**NORTHWEST TERRITORIES. Boot Inl.**: *Gillespie et al. 9541* (ALA, ari, CAN, MT, O, UBC), *9675* (CAN). **Kuujjua R.**: *Edlund 647* (CAN), *Gillespie et al. 9977* (ALA, CAN, MT, O). **Minto Inl. (head)**: *Edlund 623* (CAN), *Gillespie et al. 10157* (ALA, CAN, MT, O), *10230* (ALA, ari, CAN, MT, O, UBC, US, WIN). **Prince Albert S. (S)**: *Edlund 534* (CAN). **NUNAVUT. “30-Mile Cr.**”: *Bennett et al. 14-0653* (BABY). **Clouston B.**: *Gillespie et al. 7747* (CAN, O). **Johansen B.**: *Gillespie et al. 7995* (ALA, BABY, CAN, MT, O, UBC), *8114* (ALA, ALTA, BABY, CAN, MT, O, UBC, US), *7843* (CAN, O). **Oterkvik Pt.**: *Gillespie et al. 7686* (CAN, O), 7710 (CAN).

**Figure 15.**
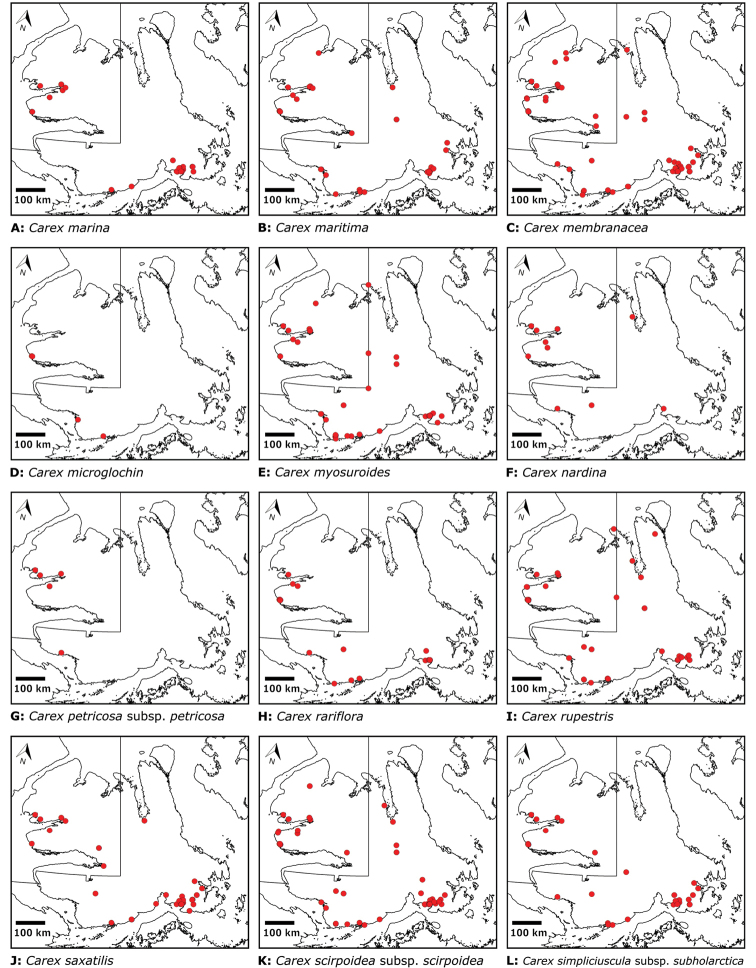
Species distribution maps. Cyperaceae: **A***Carex marina***B***Carex maritima***C***Carex membranacea***D***Carex microglochin***E***Carex myosuroides***F***Carex nardina***G**Carex petricosa subsp. petricosa**H***Carex rariflora***I***Carex rupestris***J***Carex saxatilis***K**Carex scirpoidea subsp. scirpoidea**L**Carex simpliciuscula subsp. subholarctica.

***Carex marina*** Dewey (*C. amblyorhyncha* V.I.Krecz.), Figs 15A, 14C, D – Sea sedge | Circumpolar-alpine

Previously recorded from Cambridge Bay, the head of Minto Inl. and Ulukhaktok (Porsild obs., conf.) (Porsild 1955, 1957, 1964, Porsild and Cody 1980, Aiken et al. 2007). Thannheiser et al. (2001) additionally recorded it from Johansen B. (conf.) and Richardson I. The specimen cited from Cambridge Bay (*Porsild 17466*) (Porsild 1955) apparently does not exist; another specimen with the same collection number at CAN is *Carex scirpoidea*, also cited by Porsild (1955). Whatever the reason for the error, more recent collections confirm the presence of the species in the Cambridge Bay area, though it was not mapped from there by Aiken et al. (2007). Newly recorded from Boot Inl., Ferguson L., Greiner L., Kuujjua R. and Sinclair Cr. Elsewhere in the Canadian Arctic recorded from Axel Heiberg, Baffin, Banks, Ellesmere, Nottingham and Southampton islands, as well as mainland sites (Porsild and Cody 1980, Aiken et al. 2007, Saarela et al. 2013, Saarela et al. 2017b).

**NORTHWEST TERRITORIES. Boot Inl.**: *Gillespie et al. 9640* (ALA, ari, CAN, MT, O). **Kuujjua R.**: *Gillespie et al. 9776* (ALA, CAN, O). **Minto Inl. (head)**: *Gillespie et al. 9504* (ALA, CAN, MT, O), *10039* (ALA, ari, CAN, MT, O, UBC, US, WIN), *9491* (CAN, O, WIN), *10295* (ALA, CAN, O), *Porsild 17366* (CAN). **Ulukhaktok**: *Edlund 489*, *518* (CAN), *Oldenburg 42-102*, *42-68*, *42-99C* (CAN). **NUNAVUT. Cambridge Bay**: *Bennett et al. 13-0204* (BABY, chars, MICH, od, UAC), *14-0367* (CAN, MICH), *13-0565* (MICH), *Gillespie et al. 8411* (ALA, BABY, CAN, MT, O), *Ponomarenko VI-088* (CAN), *Stephens 1199* (CAN, KANU). **Ferguson L. [Tahiryuaq**]: *Hainault* 2078 (DAO). **Greiner L.**: *Ponomarenko VI*-100B, *VI-110*, *VI-135c*, *VI-178*, *VI-189A*, *VI-315* (CAN). **Johansen B.**: *Gillespie et al. 7914* (ALA, CAN, MT, O). **Sinclair Cr.**: *Gillespie et al.* 8337 (CAN).

***Carex maritima*** Gunnerus, Figs 15B, Figs 14E–Maritime sedge | Circumpolar-alpine

Previously recorded from Albert Edward B., Cambridge Bay, Hadley B., Kuujjua R., the head of Minto Inl., Mt. Pelly, Namaycush L., the head of Prince Albert S., Read I., Richard Collinson Inl. and Ulukhaktok (Porsild 1955, 1957, 1964, Porsild and Cody 1980, Aiken et al. 2007). Thannheiser et al. (2001) additionally recorded it from Johansen B. (conf.) and Wellington B. Newly recorded from Albert Edward B., Boot Inl., Clouston B., Greiner L., Kuujjua R. and Oterkvik Pt. Elsewhere in the Canadian Arctic recorded from Baffin, Banks, Coats, Devon and Southampton islands, and across the mainland (Porsild and Cody 1980, Cody et al. 1989, Korol 1992, Cody et al. 2003, Aiken et al. 2007, Saarela et al. 2013, Saarela et al. 2017b).

**NORTHWEST TERRITORIES. Boot Inl.**: *Gillespie et al. 9651* (CAN, O). **Kuujjua R.**: *Edlund 644* (CAN), *Gillespie et al. 9943* (CAN, O). **Minto Inl. (head)**: *Edlund 132*, *176* (CAN), *Gillespie et al. 10228* (CAN, O, WIN), *10283* (ari, CAN). **Prince Albert S.**: *Porsild 17435* (CAN). **Richard Collinson Inl.**: *Edlund 691* (CAN). **Ulukhaktok**: *Edlund 347*, *457*, *775* (CAN), *Porsild 17250* (CAN), *Saarela & Bull 1457* (ALA, CAN, O). **NUNAVUT. Albert Edward B.**: *Edlund 12746* (CAN, TRTE), *Ponomarenko VI-336A* (CAN). **Cambridge Bay**: *Bennett et al. 13-0163* (ALA, BABY, CAN, chars, UBC), *Consaul & Gillespie 1120* (CAN), *Edlund & Argus 12692* (CAN), *Gillespie et al. 8370*, *8491* (CAN, O), *Oldenburg 44*-*924* (CAN), *Polunin s.n.* (CAN), *Porsild 21601* (CAN), *Stephens 1142* (CAN). **Clouston B.**: *Gillespie et al. 7750* (ALA, CAN, O). **Greiner L.**: *Ponomarenko VI-035C*, *VI-291A* (CAN). **Hadley B.**: *Edlund 113* (CAN). **Johansen B.**: *Gillespie et al. 7917* (ALA, BABY, CAN, MT, O, UBC, US), *7908* (CAN, O), *8016* (CAN, O). **Ovayok TP**: *Stephens 1184* (CAN). **Murray Pt.**: *Gillespie et al. 8196* (CAN, O). **Namaycush L.**: *Edlund 134*, *135*, *18*, *31* (CAN). **Oterkvik Pt.**: *Gillespie et al. 7613* (ALA, CAN, O). **Read I.**: *Oldenburg 42-487*, *42-492*, *43-1009*, *43-882*, *43-884*, *43-966* (CAN), *Porsild 17188* (CAN).

***Carex membranacea*** Hook., Figs 15C, 14F–Fragile sedge | Amphi-Beringian–North America (N)

Previously recorded from Byron B., C. Wollaston, Ferguson L., Kuujjua R., Mt. Bumpus, Natkusiak P., the head of Prince Albert S., Richard Collinson Inl., Tahiryuaq and Ulukhaktok (Porsild 1955, 1957, 1964, Porsild and Cody 1980, Ford and Ball 1992, Aiken et al. 2007). Thannheiser et al. (2001) additionally recorded it from Johansen B. (conf.), Richardson I., Surrey L. and Wellington B. Newly recorded from Albert Edward B., Boot Inl., Falaise B., Ferguson L., Murray Pt., Oterkvik Pt., Namaycush L., an inland site on Prince Albert P., Read I., Sinclair Cr., Tuktu R., Walker B. and Washburn L. Widespread throughout the Canadian Arctic (Porsild and Cody 1980, Cody et al. 1989, Ford and Ball 1992, Korol 1992, Aiken et al. 2007, Saarela et al. 2013, Saarela et al. 2017b).

**NORTHWEST TERRITORIES. Boot Inl.**: *Gillespie et al. 9551* (ALA, ari, CAN, MT, O, UBC). **C. Wollaston**: *Edlund 35*, *51*, *150* (CAN). **Kuujjua R.**: *Edlund 635* (CAN), *Gillespie et al.* 9727 (ALA, CAN, MT, O). **Minto Inl. (head)**: *Edlund 90*, *133* (CAN), *Gillespie et al. 10036* (ALA, CAN, MT, O), *10037* (ALA, ari, CAN, MT, O, UBC, WIN), *9493* (ALA, CAN, O), *Porsild 17367* (CAN). **Prince Albert P.**: *Oldenburg 54-658* (UBC). **Prince Albert S. (head)**: *Edlund 382* (CAN). **Richard Collinson Inl.**: *Edlund 676*, *690* (CAN). **Tahiryuaq**: *Edlund 401* (CAN). **Ulukhaktok**: *Edlund 717*, *908* (CAN), *Oldenburg 42-71*, *45-1575*, *45-1580* (CAN), *Saarela & Bull 1481* (ALA, CAN, O), *1499* (ALA, CAN, O). **Walker B.**: *Oldenburg 45-1428*, *45-1429*, *45-1432B* (CAN). **NUNAVUT. Albert Edward B.**: *Ponomarenko VI-268* (CAN). **Byron B.**: *Dushenko* (UVIC). **Cambridge Bay**: *Bennett et al. 13-0181* (CAN, chars, od), *Calder et al. 24166* (DAO), *Consaul & Gillespie 1107*, *1126* (CAN), *Edlund & Argus 12622* (CAN), *Gillespie et al. 8361* (ALA, CAN, O), *Oldenburg 44-918*, *44-959* (mixed with *Carex saxatilis*) (CAN), *Polunin s.n.* (CAN, 2 sheets), *Ponomarenko VI-080B* (CAN), *Porsild 17465*, *21514* (CAN), *Stephens 1151* (CAN), *Thomson s.n.* (WIS). **Falaise B.**: *Eriksen et al. 967* (ALA). **Ferguson L. [Tahiryuaq**]: *Hainault 2065*, *2066* (DAO), *Jones & Hainault 1862* (DAO). **Greiner L.**: *Ponomarenko VI-100F*, *VI-101a*, *VI-110A*, *VI-110B*, *VI-110C*, *VI-125*, *VI-130b*, *VI-170*, *VI-225*, *VI-229*, *VI-241B*, *VI-295A*, *VI-323* (CAN). **Johansen B.**: *Edlund & Argus 12781* (CAN, TRTE), *Gillespie et al. 7945* (ALA, ALTA, BABY, CAN, MT, O, UBC, US). **Mt. Bumpus**: *Edlund 219* (CAN). **Mt. Lady Pelly [Amaaqtuq**]: *Jones & Hainault 34a* (DAO). **Ovayok TP**: *Stephens 1162* (CAN). **Murray Pt.**: *Gillespie et al. 8204* (ALA, CAN, O). **Namaycush L.**: *Edlund* 32 (CAN). **Natkusiak P.**: *Edlund 332* (CAN). **Oterkvik Pt.**: *Gillespie et al. 7555* (ALA, BABY, CAN, MT, O, US), *7646* (CAN, O), *7791* (ALA, CAN, O). **Read I.**: *Oldenburg 42-488*, *43-1007*, *43-889*, *43-922*, *43-944*, *45-1578* (CAN). **Sinclair Cr.**: *Gillespie et al. 8300* (ALA, BABY, CAN, MO, MT, O, UBC, US). **Tuktu R.**: *Gould s.n.* (ALA). **Washburn L.**: *Oldenburg 46-2223*, *46-2224*, *46-222*8 (CAN).

***Carex microglochin*** Wahlenb., Fig. 15D–Bristle sedge | American Beringian–North American–amphi-Atlantic–European (N/C) *&* Asian (C)

Previously recorded from Ulukhaktok (Aiken et al. 2007). Thannheiser et al. (2001) additionally recorded it from Johansen B. (conf.). In this area we found the species growing in a wet sedge meadow near the mouth of Mackenzie Creek; it was uncommon. Newly recorded from Clouston B., where it grew in wet mud at the edge of a dried up pond. Elsewhere in the Canadian Arctic recorded from southern Baffin and Banks islands and a few mainland sites (Polunin 1939, Porsild and Cody 1980, Aiken et al. 2007, Saarela et al. 2013, Saarela et al. 2017b). The scattered sites across the southern portion of the Canadian Arctic Archipelago mark the northern limit of the species in Canada.

**NORTHWEST TERRITORIES. Ulukhaktok**: *Edlund 771*, *822* (CAN). **NUNAVUT. Clouston B.**: *Gillespie et al. 7751* (CAN), *7754* (CAN, O). **Johansen B.**: *Gillespie et al. 8108* (ALA, CAN, MT, O).

**Figure 16.**
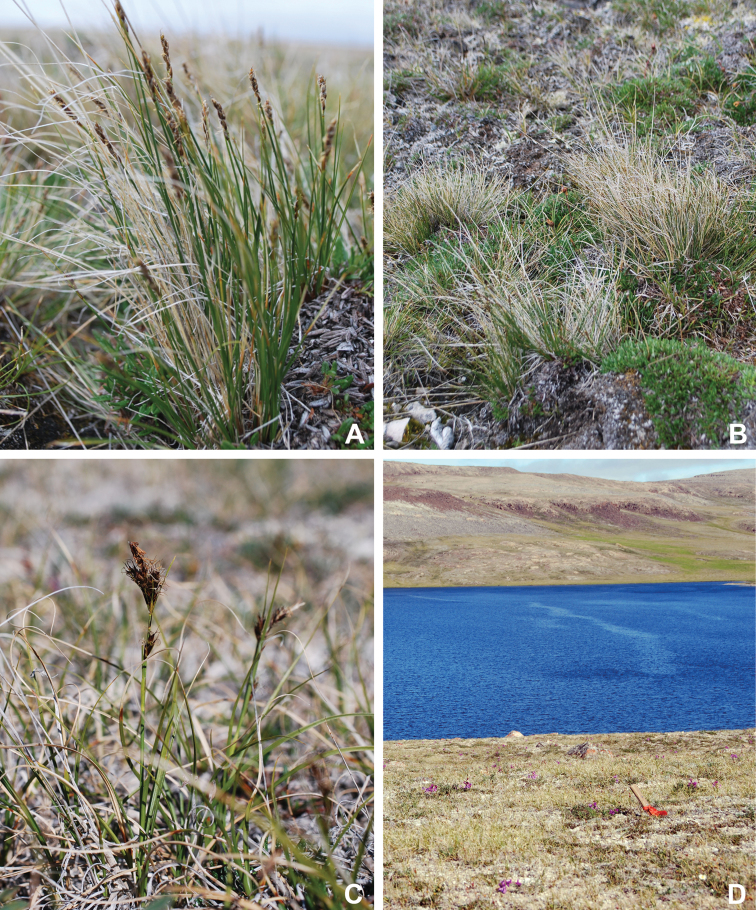
**A***Carex myosuroides* habit **B***Carex myosuroides* habitat **C**Carex petricosa subsp. petricosa habit, *Gillespie et al. 9657***D**Carex petricosa subsp. petricosa habitat, *Gillespie et al. 9657*. Photos **A**, **B** by B.A. Bennett and **C**, **D** by J.M. Saarela.

***Carex myosuroides*** Vill. (*Kobresia myosuroides* (Vill.) Fiori.), Figs 15E, 16A, B–Mouse-tail bog sedge | Circumpolar-alpine

Porsild (1955) reported this taxon from Read I., three sites (Wollaston P., Ulukhaktok [conf.] and Minto Inl. [conf.]) for which he did not cite specimens, and noted observations of the species at Walker B. (conf.), the head of Prince Albert S. and Cambridge Bay. He subsequently mapped the species at all these sites (Porsild 1957, 1964, Porsild and Cody 1980). Aiken et al. (2007) recorded it from Anderson B., Cambridge Bay, Mt. Bumpus, Ulukhaktok and Kuujjua R. Thannheiser et al. (2001) additionally recorded it from Mt. Pelly, Surrey L. and Wellington B. Newly recorded from Boot Inl., Greiner L., Natkusiak P., Oterkvik Pt., an inland site SE of the head of Prince Albert S., Richard Collinson Inl., Tahiryuaq, Sinclair Cr. and Washburn L. Elsewhere in the Canadian Arctic recorded from Baffin, Banks, Devon, Ellesmere, Melville, Somerset and Southampton islands, as well as a few mainland sites (Porsild and Cody 1980, Aiken et al. 2007, Saarela et al. 2013, Saarela et al. 2017b).

**NORTHWEST TERRITORIES. Boot Inl.**: *Gillespie et al. 9544* (ALA, CAN, O, WIN). **Burns L. (S)**: *Edlund 74* (CAN). **Kuujjua R.**: *Gillespie et al. 9723* (ALA, CAN, MT, O), *Dutilly 18818* (DAO). **Minto Inl. (head)**: *Edlund 102* (CAN), *Gillespie et al. 9495* (ALA, ari, CAN, MT, O, UBC), *10156* (ALA, CAN, O, WIN). **Natkusiak P.**: *Edlund 170* (CAN). **Richard Collinson Inl.**: *Edlund 682* (CAN). **Tahiryuaq**: *Edlund 561*, *562* (CAN). **Ulukhaktok**: *Edlund 337*, *432*, *444*, *469*, *660*, *770*, *860* (CAN), *Saarela & Bull 1417* (CAN, O). **Walker B.**: *Oldenburg 45-1409* (CAN). **NUNAVUT. Anderson B.**: *Edlund & Argus 12719* (CAN). **Cambridge Bay**: *Bennett et al. 13-0241* (chars, od), *Calder et al. 24211* (DAO), *Dutilly 28096* (US), *Edlund & Argus 12880* (CAN), *Gillespie et al. 8360* (ALA, CAN, MT, O), *Gould s.n.* (ALA), *Polunin s.n.* (CAN, 2 sheets), *Porsild 21609* (CAN), *Stephens 1106* (CAN, KSTC). **Clouston B.**: *Gillespie et al. 7742* (ALA, CAN, MT, O). **Greiner L.**: *Ponomarenko VI-145* (CAN), **Johansen B.**: *Gillespie et al. 7835* (ALA, CAN, MT, O), *8086* (ALA, BABY, CAN, MT, O, UBC, US), *8147* (CAN, MT, UBC), *7909* (CAN, O). **Mt. Bumpus**: *Edlund 232*, *42* (CAN). **Namaycush L.**: *Edlund 42*, *164* (CAN). **Oterkvik Pt.**: *Gillespie et al. 7487* (ALA, BABY, CAN, MT, O, US), *7547* (ALA, ALTA, BABY, CAN, MT, O, UBC, US), *7552* (ALA, CAN, MT, O), *7618* (ALA, BABY, CAN, MT, O), *7579* (ALA, CAN, O), *7581* (CAN). **Sinclair Cr.**: *Gillespie et al. 8303* (ALA, CAN, O). **Read I.**: *Oldenburg 43-1010*, *43*-885, *43-891* (CAN), *Porsild 17191* (CAN). **Washburn L.**: *Oldenburg 46-2221* (CAN).

***Carex nardina*** Fr. (C. nardina var. atriceps Kük.), Fig. 15F–Nard sedge | Amphi-Beringian–North American–amphi-Atlantic (W)

Previously recorded from Mt. Bumpus, and Walker B. (Porsild 1955, 1957, 1964, Porsild and Cody 1980, Aiken et al. 2007). Thannheiser et al. (2001) additionally recorded it from Cambridge Bay, Johansen B., Mt. Pelly, Ulukhaktok (conf.) and Wellington B. Newly recorded from Boot Inl., Falaise B., Ferguson L., Hadley B., Kuujjua R. and the head of Minto Inl. Elsewhere in the Canadian Arctic recorded from Baffin, Banks, Devon, Ellesmere, Mansel, Melville, Nottingham and Southampton islands, as well as mainland sites (Porsild and Cody 1980, Cody et al. 1989, Cody and Reading 2005, Aiken et al. 2007, Saarela et al. 2013, Saarela et al. 2017b).

**NORTHWEST TERRITORIES. Boot Inl.**: *Gillespie et al. 9666* (ALA, ari, CAN, MT, O, UBC). **Kuujjua R.**: *Gillespie et al. 9966* (ALA, CAN, MT, O). **Minto Inl. (head)**: *Gillespie et al. 10221* (ALA, CAN, MT, WIN), *9506* (ALA, CAN, O), *9789* (ALA, ari, CAN, MT, O). **Ulukhaktok**: *Svoboda 745004* (TRTE, UBC). **Walker B.**: *Porsild 17485* (CAN). **NUNAVUT. Falaise B.**: *Eriksen et al. 997* (ALA). **Ferguson L. [Tahiryuaq**]: *Bennett et al. 14-0430* (BABY). **Hadley B.**: *Edlund 81* (CAN). **Mt. Bumpus**: *Edlund 274* (CAN).

***Carex petricosa*** Dewey subsp. ***petricosa*** (Carex petricosa var. petricosa), Figs 15G, 16C, D–Rock-dwelling sedge | Amphi-Beringian–Cordilleran *&* North America (NE)

A rare taxon known in the Canadian Arctic Archipelago only from western Victoria I., previously recorded from Walker B. (Porsild 1955, Aiken et al. 2007). Thannheiser et al. (2001) additionally recorded it from Ulukhaktok. A site to the north of Walker B., mapped in Aiken *et al.* (2007) based on the map in Porsild and Cody (1980), is likely a misrepresentation of the Walker B. collection. A site on the north shore of Prince Albert S. was mapped by Ball and Zoladz (1994); we have not seen a voucher. Newly recorded from Falaise B., Boot Inl., the head of Minto Inl. and Kuujjua R. At the last three sites this rhizomatous taxon grew in dry rocky tundra and slopes typically associated with *Carex rupestris* and *Dryas integrifolia*. On the mainland this western taxon (Ball and Zoladz 1994) is recorded as far east as the Coppermine R. valley (Saarela et al. 2017b).

**NORTHWEST TERRITORIES. Boot Inl.**: *Gillespie et al. 9657* (ALA, ALTA, ari, CAN, MT, O, UBC, US, WIN). **Kuujjua R.**: *Gillespie et al. 9722* (ALA, ALTA, ari, CAN, MT, O, UBC, US, WIN), *9988* (ALA, CAN, O). **Minto Inl. (head)**: *Gillespie et al. 10097* (ALA, ari, CAN, MT, O, UBC). **Walker B.**: *Oldenburg 45-1423* (CAN), *Porsild 17486* (CAN). **NUNAVUT. Falaise B.**: *Eriksen et al. 1000* (ALA).

**Figure 17.**
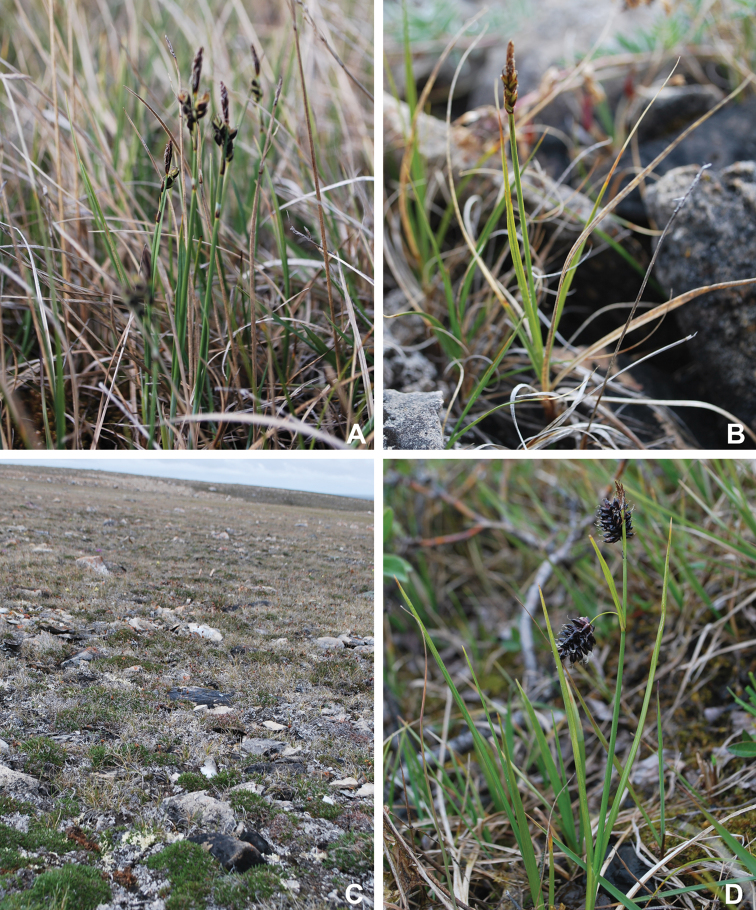
**A***Carex rariflora* habit **B***Carex rupestris* habit **C***Carex rupestris* habitat **D***Carex saxatilis* habit. Photos by B.A. Bennett.

***Carex rariflora*** (Wahlenb.) Sm., Figs 15H, 17A–Loose-flowered alpine sedge | Circumpolar

Previously recorded from Cambridge Bay and Ulukhaktok (Porsild 1955, 1957, 1964, Porsild and Cody 1980, Aiken et al. 2007). Thannheiser et al. (2001) additionally recorded it from Johansen B. (conf.), Richardson I. and Surrey L. A specimen mapped from the Kuujjua R. area in Aiken et al. (2007) has been re-determined as *C. bicolor*. Newly recorded from Boot Inl., Ferguson L., Kuujjua R., Mt. Bumpus and Oterkvik Pt. Elsewhere in the Canadian Arctic recorded from Baffin, Coats and Southampton islands and mainland sites (Porsild and Cody 1980, Cody et al. 1989, Korol 1992, Cody and Reading 2005, Aiken et al. 2007, Saarela et al. 2013, Saarela et al. 2017b). The sites around Minto Inl. mark the species’ northern limit in Canada.

**NORTHWEST TERRITORIES. Boot Inl.**: *Gillespie et al. 9548* (ALA, ari, CAN, MT, WIN). **Kuujjua R.**: *Gillespie et al. 9785* (ALA, CAN, MT, O), *9944* (CAN). **Ulukhaktok**: *Edlund 517*, *730*, *759*, *808* (CAN), *Porsild 17253* (CAN). **NUNAVUT. Cambridge Bay**: *Bennett et al. 14-0368* (CAN, DAO, UBC), *13-0238* (BABY, chars, UBC), *Edlund & Argus 12697* (CAN), *Gillespie et al. 8408* (ALA, BABY, CAN, MT, O, US), *Polunin s.n.* (CAN, 2 sheets), *Stephens 1131* (CAN). **Falaise B.**: *Eriksen et al. 948* (ALA). **Ferguson L. [Tahiryuaq**]: *Hainault 1870* (DAO). **Johansen B.**: *Gillespie et al. 7918* (ALA, CAN, O), *8112* (CAN, O). **Mt. Bumpus**: *Edlund 249* (CAN). **Oterkvik Pt.**: *Gillespie et al. 7650* (CAN, O).

***Carex rupestris*** All., Figs 15I, 17B, C–Rock sedge | Circumpolar-alpine

Previously recorded from Cambridge Bay, C. Wollaston, the head of Minto Inl., Mt. Bumpus, Namaycush L., Natkusiak P., the head of Prince Albert S., Read I., Richard Collinson Inl., Storkerson P. and Ulukhaktok (Porsild 1955, 1957, 1964, Porsild and Cody 1980, Aiken et al. 2007). Thannheiser et al. (2001) additionally recorded it from Hadley B. (conf.), Johansen B. (conf.), Mt. Pelly (conf.), Surrey L. and Wellington B. Newly recorded from Boot Inl., Colville Mts., Ferguson L., Greiner L. and Tahiryuaq. Elsewhere in the Canadian Arctic recorded from Baffin, Banks, Ellesmere, Melville, Somerset and Southampton islands and mainland sites (Porsild and Cody 1980, Korol 1992, Aiken et al. 2007, Saarela et al. 2013, Saarela et al. 2017b).

**NORTHWEST TERRITORIES. Boot Inl.**: *Gillespie et al. 9670* (ari, CAN, O). **C. Wollaston**: *Edlund 34*, *44* (CAN). **Kuujjua R.**: *Gillespie et al. 9721* (ALA, CAN, WIN). **Minto Inl. (head)**: *Edlund 65* (CAN), *Gillespie et al. 10222* (CAN). **Natkusiak P.**: *Edlund 110* (CAN). **Tahiryuaq**: *Edlund 560* (CAN). **Ulukhaktok**: *Bandringa 312* (CAN, UBC), *Dutilly 18655* (US), *Edlund 339*, *470*, *716*, *856* (CAN), *Oldenburg 42-99E* (CAN), *Porsild 17254* (CAN). **NUNAVUT. Cambridge Bay**: *Bennett et al. 13-0187* (BABY, chars, UBC), *Calder et al. 24151* (DAO), *Edlund & Argus 12634* (CAN), *Gillespie et al. 8364* (ALA, CAN, MT, O), *Gould s.n.* (ALA), *Oldenburg 44-912* (CAN), *Polunin s.n.* (CAN, 2 sheets), *Porsild 21604* (CAN), *Stephens 1105* (CAN, KANU, KSTC). **Colville Mts.**: *Gillespie et al. 7762* (CAN). **Ferguson L. [Tahiryuaq**]: *Hainault 2133* (DAO). **Greiner L.**: *Ponomarenko VI*-*035B*, *VI-119A*, *VI-121C*, *VI-172b* (CAN). **Hadley B.**: *Edlund 30*, *141* (2 sheets) (CAN). **Johansen B.**: *Gillespie et al. 7839* (CAN), *7989* (ALA, CAN, O). **Mt. Bumpus**: *Edlund 236*, *173*, *272* (CAN). **Ovayok TP**: *Ponomarenko VI-284* (CAN), *Stephens 1186* (CAN, KANU, KSTC). **Namaycush L.**: *Edlund 41*, *122*, *167* (CAN). **Oterkvik Pt.**: *Gillespie et al. 7493* (BABY, CAN, O), *7509* (CAN, O), *7674* (ALA, CAN, O). **Storkerson P.**: *Edlund 188* (CAN). **Read I.**: *Oldenburg 43-987* (CAN), *Porsild 17189* (CAN).

***Carex saxatilis*** L. (C. saxatilis var. rhomalea Fernald, C. saxatilis subsp. laxa (Trautv.) Kalela, *C. physocarpa* J.Presl & C.Presl), Figs 15J, 17D–Russet sedge | Circumboreal-polar

Previously recorded Anderson B., Cambridge Bay, Hadley B., Jonnessee L., the head of Minto Inl. and Tahiryuaq (Porsild 1955, 1957, 1964, Porsild and Cody 1980, Ford and Ball 1992, Aiken et al. 2007). Thannheiser et al. (2001) additionally recorded it from Johansen B. (conf.), Richardson I. and Surrey L. Newly recorded from “30-Mile Cr.”, Albert Edward B., Boot Inl., Ferguson L., Greiner L., Mt. Bumpus, Mt. Lady Pelly, Sinclair Cr. and Walker B. Elsewhere in the Canadian Arctic recorded from Baffin, Banks, Coats, Ellesmere and Southampton islands and mainland sites (Porsild and Cody 1980, Ford and Ball 1992, Korol 1992, Aiken et al. 2007, Saarela et al. 2013, Saarela et al. 2017b).

**NORTHWEST TERRITORIES. Boot Inl.**: *Gillespie et al. 9554* (ALA, ari, CAN, MT, UBC, WIN). **Kuujjua R.**: *Gillespie et al. 9784* (ALA, CAN, O). **Minto Inl. (head)**: *Gillespie et al. 9494* (ALA, ari, CAN, MT, O, UBC), *10034* (ALA, ari, CAN, MT, O, UBC), *Porsild 17368* (CAN). **Prince Albert S. (head)**: *Porsild 17437* (CAN). **Tahiryuaq**: *Edlund 402*, *403*, *404* (CAN). **Ulukhaktok**: *Edlund 491* (CAN). **Walker B.**: *Oldenburg 45-1431* (CAN). **NUNAVUT. “30-Mile Cr.**”: *Bennett et al. 14-0340* (UBC). **Albert Edward B.**: *Ponomarenko VI-266b* (CAN). **Anderson B.**: *Edlund & Argus 12712* (CAN, TRTE), *12726* (CAN, TRTE). **Cambridge Bay**: *Bennett et al. 13-0251* (BABY, chars, UBC, od), *Calder et al. 24169* (DAO), *Edlund & Argus 12684* (CAN, TRTE), *Gillespie et al. 8362* (ALA, BABY, CAN, MT, O), *8405* (ALA, BABY, CAN, MT, O, US), *Oldenburg 44-959* (CAN, mixed with *Carex membranacea*), *Polunin s.n.* (CAN, 2 sheets), *Ponomarenko VI-084* (CAN), *Smith & Sweatman 34B* (US, DAO), *Stephens 1023*, *1059* (CAN). **Ferguson L. [Tahiryuaq**]: *Hainault 2012*, *2019* (DAO). **Greiner L.**: *Ponomarenko VI-109*, *VI-124a*, *VI-166B*, *VI-176*, *VI-238*, *VI-241*, *VI-295B*, *VI-303* (CAN). **Hadley B.**: *Edlund 117* (CAN). **Johansen B.**: *Gillespie et al. 8047* (ALA, ALTA, BABY, CAN, MT, O, UBC, US). **Jonnessee L.**: *Edlund & Argus 12780* (CAN, mixed with *C. atrofusca*). **Mt. Bumpus**: *Edlund 250* (CAN). **Mt. Lady Pelly [Amaaqtuq**]: *Hainault 1843* (DAO). **Sinclair Cr.**: *Gillespie et al. 8245* (ALA, CAN, O), *8301* (ALA, ALTA, BABY, CAN, MT, O, UBC, US).

**Figure 18.**
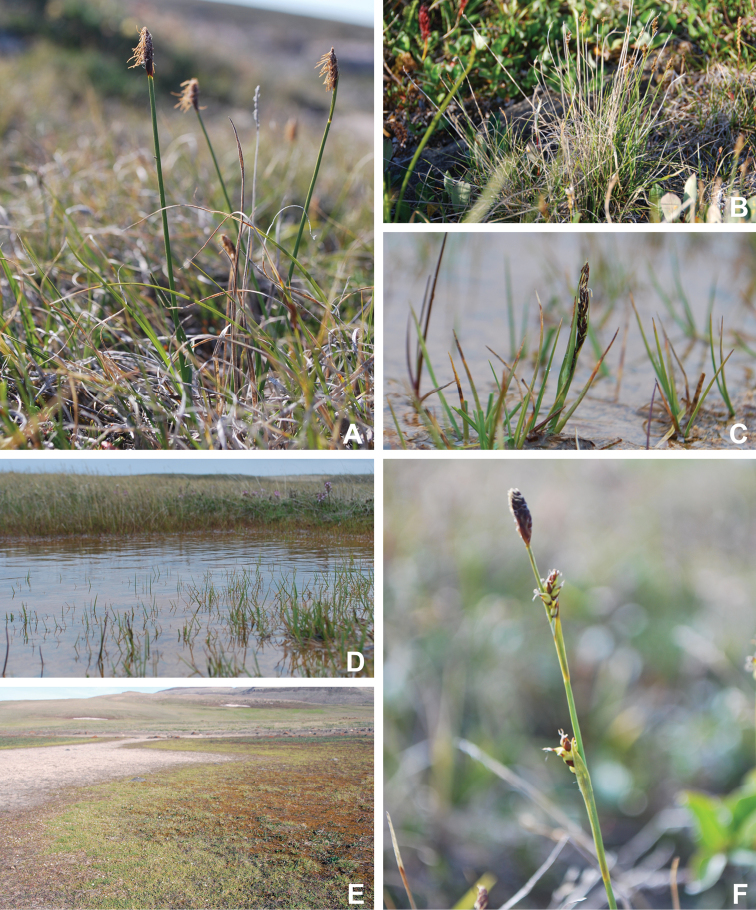
**A**Carex scirpoidea subsp. scirpoidea habit, staminate plants **B**Carex simpliciuscula subsp. subholarctica habit, Kuujjua River, NT, 18 July 2010 **C***Carex subspathacea* habit **D***Carex subspathacea* habitat **E***Carex ursina* habitat, *Gillespie et al. 9964***F***Carex vaginata* inflorescence. Photos **A**, **C**, **D**, **F** by B.A. Bennett and **B**, **E** by J.M. Saarela.

***Carex scirpoidea*** Michx. subsp. ***scirpoidea***, Figs 15K, 18A–Scirpus sedge | Amphi-Beringian–North America (N)–amphi-Atlantic (W)

Previously recorded from Cambridge Bay, Ferguson L., Kuujjua R., “Long L.”, the head of Minto Inl., Mt. Bumpus, Namaycush L., the head of Prince Albert S., Richard Collinson Inl. and Ulukhaktok (Porsild 1955, 1957, 1964, Porsild and Cody 1980, Aiken et al. 2007)(Dunlop and Crow 1999). Thannheiser et al. (2001) additionally recorded it from Johansen B. (conf.), Mt. Pelly (conf.), Richardson I., Surrey L. and Wellington B. Newly recorded from Boot Inl., C. Wollaston, Clouston B., Colville Mts., Greiner L., Murray Pt., Oterkvik Pt., Read I., Sinclair Cr., “Trunsky L.” and Washburn L. Elsewhere in the Canadian Arctic recorded from Baffin, Banks, Coats, Devon, Ellesmere, King William and Southampton islands and mainland sites (Porsild and Cody 1980, Cody et al. 1989, Korol 1992, Dunlop and Crow 1999, Cody and Reading 2005, Aiken et al. 2007, Saarela et al. 2013, Saarela et al. 2017b).

**NORTHWEST TERRITORIES. Boot Inl.**: *Gillespie et al. 9558* (ALA, ari, CAN, MT, O, UBC), *9669* (ari, CAN, MT, O). **C. Wollaston**: *Edlund 9*, *68*, *69* (CAN). **Diamond Jenness P.**: *Edlund 633* (CAN). **Kuujjua R.**: *Gillespie et al. 9780* (ALA, ALTA, ari, CAN, MT, O, UBC, US), *9781* (CAN, O). **Minto Inl. (head)**: *Edlund 92* (2 sheets), *130* (CAN), *Gillespie et al. 9502* (ALA, CAN, MT, O), *9503* (CAN, O). **Prince Albert S. (head)**: *Edlund 400* (CAN, US). **Richard Collinson Inl.**: *Edlund 171*, *181* (CAN). **Ulukhaktok**: *Bliss s.n.* (ALTA), *Edlund 311*, *342*, *726* (CAN), *Oldenburg 42-56*, *42-66*, *45-1577* (CAN), *Porsild 17255* (CAN), *Saarela & Bull 1498* (ALA, CAN, O, WIN). **Walker B.**: *Oldenburg 46-2226* (CAN). **NUNAVUT. Cambridge Bay**: *Bennett et al. 13-0186* (BABY, chars, od), *Calder et al. 24152* (DAO), *Consaul & Gillespie 1111* (CAN), *Gillespie et al.* 8363 (ALA, CAN, MT, O), *Gould s.n.* (ALA), *Oldenburg 44-906*, *44-915* (CAN), *Polunin s.n.* (CAN), *Ponomarenko VI-060*, *VI-080f* (CAN), *Porsild 17466* (CAN), *Stephens 953* (ID), *999*, *1001* (KSTC), *998* (CAN, KSTC), *959*, *1189*, 1190 (CAN), *Washburn 13* (CAN). **Clouston B.**: *Gillespie et al. 7743* (ALA, CAN, O). **Colville Mts.**: *Gillespie et al. 7764* (ALA, CAN, MT, O). **Ferguson L. [Tahiryuaq**]: *Hainault 2139* (DAO). **Greiner L.**: *Ponomarenko VI-172*, *VI-119B*, *VI-213*, *VI-225A*, *VI-239*, *VI-295* (CAN). **Hadley B.**: *Edlund 82*, *98* (CAN). **Johansen B.**: *Gillespie et al. 7834* (ALA, BABY, CAN, MT, O), *8160* (CAN). “**Long L.**”: *Lambert s.n.* (CAN). **Mt. Bumpus**: *Edlund 172*, *28*2 (CAN). **Ovayok TP**: *Stephens 999*, *1001* (KSTC). **Murray Pt.**: *Gillespie et al. 8203* (CAN, O). **Namaycush L.**: *Edlund 123*, *124* (CAN), *Edlund & Roncato-Spencer 106* (CAN). **Oterkvik Pt.**: *Gillespie et al. 7470* (ALA, BABY, CAN, MT, O, US), *7474* (CAN), *7502* (CAN). **Read I.**: *Oldenburg 42-481*, *42-490*, *45-1586* (CAN). **Sinclair Cr.**: *Gillespie et al. 8304* (ALA, CAN, O). “**Trunsky L.**”: *Bennett et al. 14-0388* (UBC). **Washburn L.**: *Oldenburg 46-2222* (CAN).

***Carex simpliciuscula*** subsp. ***subholarctica*** (T.V.Egorova) Saarela (Kobresia simpliciuscula subsp. subholarctica T.V.Egorova), Figs 15L, 18B–Simple bog sedge | Asian (NE)–amphi-Beringian–North America (N)–amphi-Atlantic (W)

Previously recorded from Ulukhaktok, the head of Minto Inl., Walker B. and Tahoe L. (Porsild 1955, 1957, 1964, Porsild and Cody 1980, Aiken et al. 2007). Thannheiser et al. (2001) recorded it only from Surrey L. Newly recorded from Albert Edward B., Boot Inl., Falaise B., Greiner L., Johansen B., Kuujjua R., Mt. Bumpus, near the head of Prince Albert S. and Sinclair Cr. Elsewhere in the Canadian Arctic recorded from Baffin, Banks, Devon, Ellesmere and Southampton islands and mainland sites (Porsild and Cody 1980, Cody et al. 1989, Aiken et al. 2007, Saarela et al. 2013, Saarela et al. 2017b).

**NORTHWEST TERRITORIES. Boot Inl.**: *Gillespie et al. 9549* (CAN, O). **Kuujjua R.**: *Gillespie et al. 9728* (CAN, O). **Minto Inl. (head)**: *Gillespie et al. 9490* (ALA, CAN, O), *Porsild 17372* (CAN). **Prince Albert S. (head)**: *Edlund 406* (CAN). **Ulukhaktok**: *Edlund 338*, *339* (CAN), *468*, *831* (CAN, US), *Oldenburg 42-99D*, *45-1562* (CAN), *Saarela & Bull 1500* (CAN, O, WIN). **Walker B.**: *Oldenburg 45-1408* (CAN), *Porsild 17488* (CAN). **NUNAVUT. Albert Edward B.**: *Ponomarenko VI-251*, *VI-265* (CAN). **Cambridge Bay**: *Bennett et al. 13-0557* (CAN), *13-0562* (UBC, chars), *Gillespie et al. 8410* (ALA, BABY, CAN, MT, O, UBC), *Stephens 1132* (KSTC). **Falaise B.**: *Eriksen et al. 947* (ALA). **Ferguson L. [Tahiryuaq**]: *Hainault & Jones 1863*, *1867* (DAO). **Greiner L.**: *Ponomarenko VI-100G*, *VI-114a*, *VI-135b*, *VI-152*, *VI-173*, *VI-235*, *VI-301* (CAN). **Johansen B.**: *Gillespie et al. 7946* (ALA, BABY, CAN, MT, O), *8019* (ALA, BABY, CAN, MT, O). **Mt. Bumpus**: *Edlund 283* (CAN). **Murray Pt.**: *Gillespie et al. 8209* (ALA, CAN, O). **Sinclair Cr.**: *Gillespie et al. 8343* (CAN). **Tahoe L.**: *Porsild 17452* (CAN).

**Figure 19.**
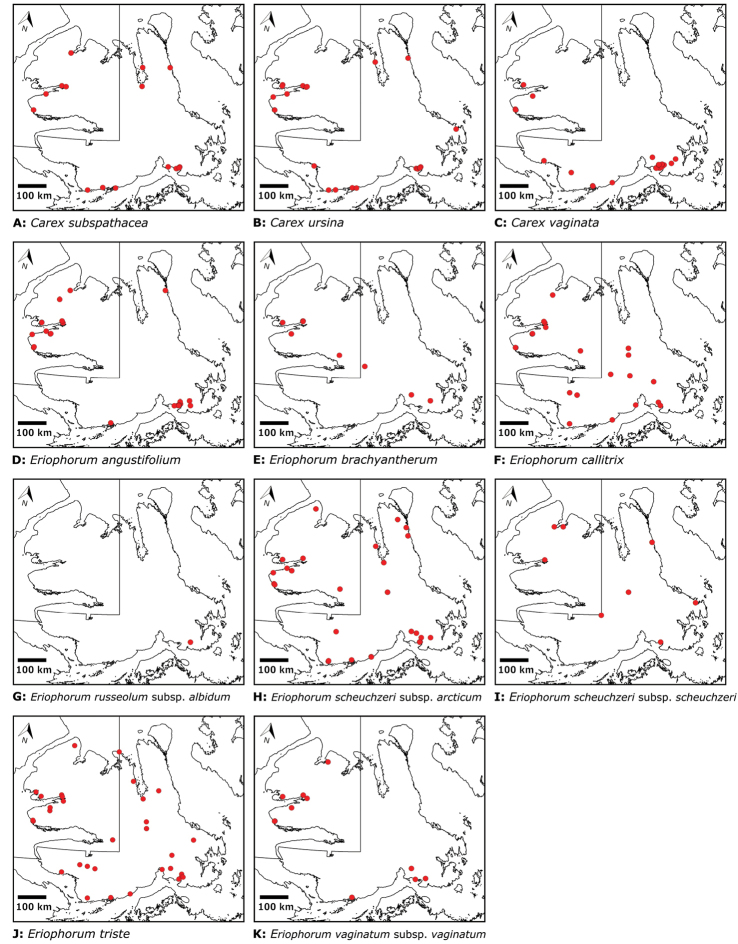
Species distribution maps. Cyperaceae: **A***Carex subspathacea***B***Carex ursina***C***Carex vaginata***D***Eriophorum angustifolium***E***Eriophorum brachyantherum***F***Eriophorum callitrix***G**Eriophorum russeolum subsp. albidum**H**Eriophorum scheuchzeri subsp. arcticum**I**Eriophorum scheuchzeri subsp. scheuchzeri**J***Eriophorum triste***K**Eriophorum vaginatum subsp. vaginatum.

***Carex subspathacea*** Wormsk., Figs 19A, 18C, D–Hoppner’s sedge | Circumpolar

Previously recorded from Cambridge Bay, Hadley B., the head of Minto Inl., Richard Collinson Inl., Storkerson P., Ulukhaktok and Read I. (Porsild obs.) (Porsild 1955, 1957, 1964, Porsild and Cody 1980, Aiken et al. 2007). Thannheiser et al. (2001) additionally recorded it from Johansen B. (conf.), Surrey L. and Wellington B. Newly recorded from Kuujjua R., Murray Pt. and Oterkvik Pt. Elsewhere in the Canadian Arctic recorded from Baffin, Banks, Devon, Ellesmere, King William and Southampton islands and scattered mainland sites (Porsild and Cody 1980, Korol 1992, Cody and Reading 2005, Aiken et al. 2007, Saarela et al. 2013, Saarela et al. 2017b).

**NORTHWEST TERRITORIES. Kuujjua R.**: *Gillespie et al. 9941* (ALA, ari, CAN, O, WIN). **Minto Inl. (head)**: *Gillespie et al. 10239* (CAN), *Porsild 17369* (CAN). **Richard Collinson Inl.**: *Edlund 692* (CAN). **Ulukhaktok**: *Edlund 348* (CAN). **NUNAVUT. Cambridge Bay**: *Bennett et al. 13-0271* (BABY, CAN, chars, od), *Edlund & Argus 12871* (CAN, TRTE), *Gillespie et al. 8379* (ALA, BABY, CAN, MT, O), *Polunin s.n.* (CAN), *Porsild 21605* (CAN), *Stephens 1153*, *1274* (CAN). **Hadley B.**: *Edlund 130*, *315* (CAN). **Johansen B.**: *Gillespie et al. 8113* (ALA, CAN, MT, O). **Murray Pt.**: *Gillespie et al. 8192* (CAN, O), *8194* (CAN, O). **Oterkvik Pt.**: *Gillespie et al. 7633* (CAN). **Storkerson P.**: *Edlund 306* (CAN).

***Carex ursina*** Dewey, Figs 19B, 18E–Bear sedge | Circumpolar

Previously recorded from Cambridge Bay, C. Wollaston, Collinson P., Hadley B., the head of Minto Inl., Read I. and Ulukhaktok (Porsild 1955, 1957, 1964, Porsild and Cody 1980, Aiken et al. 2007). Thannheiser et al. (2001) additionally recorded it from Johansen B. (conf.) and Wellington B. Newly recorded from Boot Inl., Kuujjua R., Murray Pt. and Oterkvik Pt. Elsewhere in the Canadian Arctic recorded from Axel Heiberg, Baffin, Banks, Coats, Devon, Eglinton, Ellesmere, King William, Melville, Prince Charles, Prince Patrick, Somerset and Southampton islands and a few mainland sites (Porsild and Cody 1980, Korol 1992, Aiken et al. 2007, Saarela et al. 2013, Saarela et al. 2017b).

**NORTHWEST TERRITORIES. Boot Inl.**: *Dutilly 18718* (US), *Gillespie et al. 9664* (ALA, ari, CAN, MT, O). **C. Wollaston**: *Edlund 14* (CAN). **Kuujjua R.**: *Gillespie et al. 9942* (ALA, CAN, O). **Minto Inl. (head)**: *10274* (ALA, ari, CAN, MT, O, UBC, WIN), *10238* (ALA, CAN, O), *Porsild 17370* (CAN). **Ulukhaktok**: *Dutilly 18653* (MT, US), *Edlund 349* (CAN), *Saarela & Bull 1450* (CAN, O). **NUNAVUT. Cambridge Bay**: *Bennett 14-0302* (UBC), *13-0242* (chars, od), *Dutilly 28084* (US), *Gillespie et al. 8484* (ALA, CAN, MT, O), *8375* (CAN), *Polunin s.n.* (CAN), *Porsild 21606* (CAN), *Scotter s.n.* (ALTA). **Collinson P.**: *Edlund & Argus* 12757 (CAN). **Hadley B.**: *Edlund 313* (CAN). **Johansen B.**: *Gillespie et al. 8021* (ALA, CAN, O). **Murray Pt.**: *Gillespie et al. 8198* (ALA, CAN, MT, O). **Oterkvik Pt.**: *Gillespie et al. 7619* (ALA, ALTA, BABY, CAN, MT, O, UBC, US), *7706* (CAN, O). **Storkerson P.**: *Edlund 298* (CAN).

***Carex vaginata*** Tausch, Figs 19C, 18F–Sheathed sedge | Circumboreal-polar

Previously recorded from Cambridge Bay, the north side of Prince Albert S. (Porsild obs.), Ulukhaktok and southwestern Wollaston P. (Porsild 1955, 1957, 1964, Porsild and Cody 1980, Aiken et al. 2007). Thannheiser et al. (2001) additionally recorded it from Johansen B. (conf.) and Richardson I. Newly recorded from Boot Inl., Falaise B., Ferguson L., Greiner L., Kuujjua R. and Sinclair Cr. Elsewhere in the Canadian Arctic recorded from southern Baffin, Banks and Southampton islands, and across the mainland (Porsild and Cody 1980, Cody et al. 1984a, Cody et al. 1989, Korol 1992, Cody and Reading 2005, Aiken et al. 2007, Saarela et al. 2013, Saarela et al. 2017b).

**NORTHWEST TERRITORIES. Boot Inl.**: *Gillespie et al. 9665* (CAN, O). **Kuujjua R.**: *Gillespie et al. 9787* (ALA, CAN, O, WIN). **Ulukhaktok**: *Edlund 488*, *729* (CAN), *Oldenburg 42-62*, *45-1571* (CAN), *Porsild 17828* (CAN). **NUNAVUT. Cambridge Bay**: *Bennett et al. 13-0191* (BABY, chars), *Consaul & Gillespie 1125* (CAN), *Edlund 746* (CAN), *Gillespie et al. 8369* (ALA, BABY, CAN, MT, O), *Gould s.n.* (ALA), *Ponomarenko* VI-086D, VI-089B, VI-090C (CAN). **Falaise B.**: *Eriksen et al. 1001* (ALA). **Ferguson L. [Tahiryuaq**]: *Hainault 2064* (DAO). **Greiner L.**: *Ponomarenko VI-034*, *VI-049*, *VI-100A*, *VI-101*, *VI-114b*, *VI-114c*, *VI-179*, *VI-236* (CAN). **Johansen B.**: *Gillespie et al. 7877* (ALA, BABY, CAN, MT, O), *7953* (ALA, CAN, O). **Sinclair Cr.**: *Gillespie et al. 8342* (ALA, CAN, O). **Wollaston P. (SW)**: *Edlund 522* (CAN).

### *Eriophorum* L. [7/8]

**Key to *Eriophorum* [adapted from Ball and Wujek (2002), Cayouette (2004) and Elven et al. (2011)]**

Note: In *Eriophorum*, the head-like structures are spikelets.

**Table:** 

1	Spikelets 2 or more, spreading or nodding, subumbellate or capitate, subtended by 1 or more blade-bearing involucral bracts, sometimes reduced to sheaths	**2**
–	Spikelets solitary, erect, without blade-bearing involucral bracts	**3**
2	Peduncles smooth, drooping, up to 5–10 cm; lowermost involucral bract cylindrical, flowering spikelets oblong-ovoid or oblong-elliptical; fruiting spikelets bell-shaped or narrowly bell-shaped; scales brownish grey, greyish, reddish or ferrugineous with white margins; anthers (2.5–)3–4(–5) mm; achenes oblong-obovoid or oblong-elliptical, (2.5–)2.8–3(–3.5) mm	***E. angustifolium***
–	Peduncles scabrous, arcuate, up to 2 cm; lowermost involucral bract funnel-shaped, flowering spikelets ovoid to almost spherical; fruiting spikelets obovoid; scales blackish, without whitish margins; anthers (1.8–)2.5–2.8(–3) mm; achenes widely obovoid, 2–2.5 mm	***E. triste***
3	Plants rhizomatous, forming mats; culms solitary; empty proximal scales of spikelets usually not more than 7	**4**
–	Plants cespitose; culms densely tufted; empty proximal scales of spikelets usually 10 or more	**6**
4	Medial scales of spikelets (0.8–)1.0–2.4 mm wide, acute, 0.25–0.6 mm wide at 0.2 mm below the apex, widest mostly at the middle or above, with well developed hyaline margins; anthers (1.3–)1.5–3.1 mm; achenes ellipsoid or obovoid, scabrous or glabrous, beak base 0.1–0.2 mm wide	**E. russeolum subsp. albidum**
–	Medial scales of spikelets 0.3–1.5(–1.7) mm wide, acuminate to narrowly acuminate, 0.05–0.3(–0.4) mm wide at 0.2 mm below the apex, widest below the middle or close to the base, with frequently reduced hyaline margins; anthers 0.35–1.6 mm; achenes narrowly obovoid, always glabrous, beak base 0.05–0.1 mm wide (*E. scheuchzeri*)	**5**
5	Spikelets hemispherical; proximal fertile scales of spikelets dark, with dark margins or reduced hyaline margins sharply differentiated from the darker parts; medial scales narrowly acuminate (usually 0.1 mm wide at 0.2 mm below the apex), 0.3–0.7(–0.9) mm wide near the middle; mature achenes beige brown to olive-brown, slightly lustrous	**E. scheuchzeri subsp. scheuchzeri**
–	Spikelets spherical; proximal fertile scales of spikelets bicoloured, with lower and medial parts dark but gradually passing to various tones of gray and conspicuous marginal and apical hyaline areas; medial scales acuminate (usually 0.2 mm wide at 0.2 mm below the apex), (0.5–)0.7–1.4(–1.6) mm wide near the middle; mature achenes orange-brown to dark reddish-brown, mostly dull	**E. scheuchzeri subsp. arcticum**
6	Proximal scales spreading or reflexed in fruit, with white-hyaline margins to 1 mm wide; anthers 2–3 mm; distal sheaths on culms inflated; usually forming large, dense tussocks	**E. vaginatum subsp. vaginatum**
–	Proximal scales appressed to ascending, without conspicuous whitish margins; anthers 0.5–2 mm; distal sheaths on culms inflated or not; forming small, loose or compact tussocks	**7**
7	Culms 5–30 cm; distal leaf usually above the middle of the culm, sheath strongly inflated; proximal scales of spikelets black or dark gray, ovate, 3–4 mm wide, veins more or less reaching the margins; perianth bristles shiny white; achenes ellipsoid-obovoid, 1.8–2.1 mm	***E. callitrix***
–	Culms 30–70 cm, rarely shorter; distal leaf usually above the middle of the culm, sheath not or weakly inflated; proximal scales of spikelets dark gray, lanceolate, 2–3 mm wide, veins not reaching the margins; perianth bristles creamy white; achenes oblanceoloid, (1.8–)2–2.3(–2.7) mm	***E. brachyantherum***

**Figure 20.**
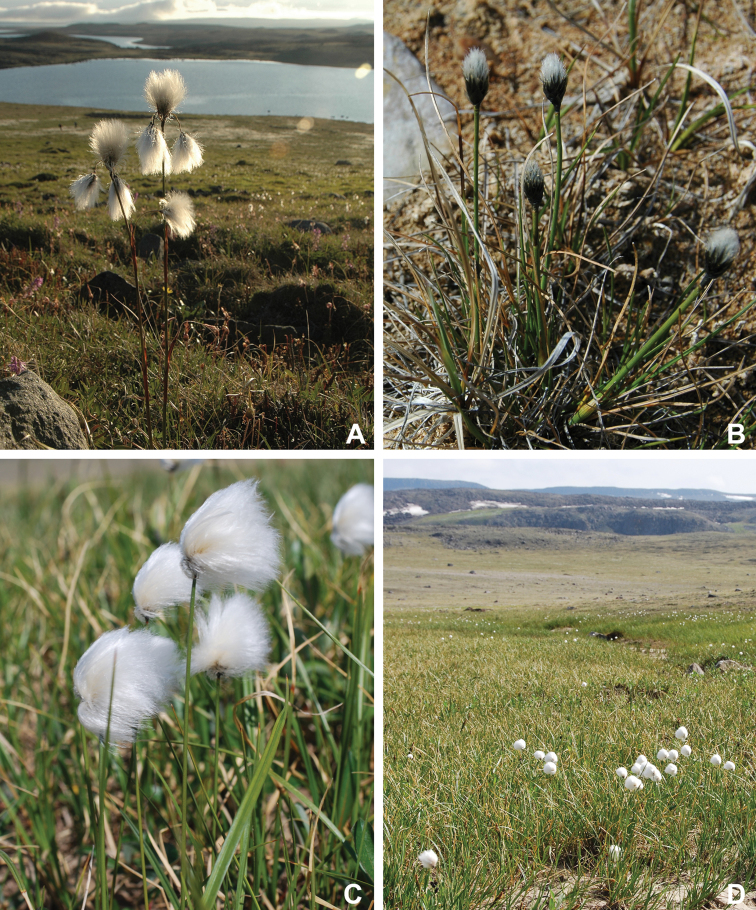
**A***Eriophorum angustifolium* habit, *Gillespie et al. 9984***B***Eriophorum callitrix* habit, *Gillespie et al. 7477***C**Eriophorum scheuchzeri subsp. arcticum inflorescences **D**Eriophorum scheuchzeri subsp. arcticum habitat, *Gillespie et al 9857 & 9861*. Photos **A**, **B** by R.D. Bull and **C**, **D** by J.M. Saarela.

***Eriophorum angustifolium*** Honck., Figs 19D, 20A–Narrow-leaved cottongrass | Circumboreal-polar

Previously recorded from Cambridge Bay, Storkerson P. and Ulukhaktok (Porsild and Cody 1980, Aiken et al. 2007). Two specimens previously identified as this species from the head of Prince Albert S. (*Bezener 45*, DAO; *Weerstra 28*, DAO), mapped in Aiken et al. (2007), have been redetermined as *E. triste*. Similarly, a record mapped as this species from the west side of Hadley B. (*Edlund* 37, CAN) is *E. triste*. Thannheiser et al. (2001) additionally recorded it from Johansen B. (conf.), Hadley B., the head of Minto Inl. (conf.), Richardson I. and Surrey L. Newly recorded from Boot Inl., Greiner L., Kuujjua R., an inland site on Prince Albert P. and Richard Collinson Inl. Elsewhere in the Canadian Arctic recorded from Baffin, Banks, Coats, Ellesmere, Melville, Prince of Wales, Somerset and Southampton islands, and across the mainland (Porsild and Cody 1980, Cody et al. 1989, Cody and Reading 2005, Aiken et al. 2007, Saarela et al. 2013, Saarela et al. 2017b)

**NORTHWEST TERRITORIES. Boot Inl.**: *Dutilly 18710* (US), *18709* (DAO), *Gillespie et al. 9552* (CAN, O). **C. Wollaston**: *Edlund 33* (CAN). **Kuujjua R.**: *Dutilly 18788* (US), *Gillespie et al. 9984* (ALA, ari, CAN, MT, O), *9777* (CAN, O, WIN). **Minto Inl. (head)**: *Edlund 143* (CAN), *Gillespie et al. 9489* (ALA, CAN, MT, O), *9458* (ALA, CAN, O, WIN). **Prince Albert P.**: *Oldenburg 54-660* (UBC). **Richard Collinson Inl.**: *Edlund 708* (CAN). **Ulukhaktok**: *Edlund 315* (CAN, ID), *Salokangas 26* (CAN, UBC). **NUNAVUT. Cambridge Bay**: *Bennett et al. 13-0182* (BABY, UBC, chars), *Edlund & Argus 12614* (CAN), *Gillespie et al. 8406* (ALA, ALTA, BABY, CAN, MT, O, UBC, US), *Oldenburg 44*-*891* (CAN), *Polunin s.n.* (CAN, 2 sheets), *Ponomarenko VI-090B* (CAN), *Scotter s.n.* (ALTA), *Stephens 857* (CAN, KANU, KSTC), *957* (CAN, KANU, KSTC), *1021* (CAN, KANU, KSTC), *1107* (CAN, KANU, KSTC). **Greiner L.**: *Ponomarenko VI-034A*, *VI-124b*, *VI-166A* (CAN). **Johansen B.**: *Gillespie et al. 7913* (ALA, CAN, MT, O). **Storkerson P.**: *Edlund 287* (CAN).

***Eriophorum brachyantherum*** Trautv. & C.A.Mey., Fig. 19E–Closed-sheath cottongrass | Circumboreal-polar

Newly reported from Victoria I. and the western Canadian Arctic Archipelago in Gillespie et al. (2015), based on collections we made in 2010 in the Minto Inl. area. Four additional records for Victoria I. are reported here, from northeast of the head of Prince Albert S., the Kagloryuak R. area east of the head of Prince Albert S. (*Edlund & Argus 12829*, previously determined as *E. callitrix*), Ferguson L. and the Greiner L. watershed ca. 43 km ENE of Cambridge Bay. Elsewhere in the Canadian Arctic recorded from Baffin and Southampton islands and scattered mainland sites (Porsild and Cody 1980, Korol 1992, Aiken et al. 2007, Saarela et al. 2013, Saarela et al. 2017b).

**NORTHWEST TERRITORIES. Boot Inl.**: *Gillespie et al. 9673* (ALA, CAN, MT, O). **Kuujjua R.**: *Gillespie et al. 9899* (ALA, CAN, mixed with *E. callitrix*, MT, O), *Gillespie et al. 9982* (ALA, CAN, MT, O). **Minto Inl. (head)**: *Gillespie et al. 10102* (ALA, ALTA, ari, CAN, MT, O, UBC, US, WIN), *9485* (ALA, CAN, MT, O), *10305* (ALA, ari, CAN, MT, O, UBC, WIN), *10091* (CAN). **Prince Albert S. (head)**: *Edlund 391* (CAN). **NUNAVUT. Ferguson L. [Tahiryuaq**]: *Hainault 2076* (DAO). **Greiner L.**: *Ponomarenko VI-180* (CAN). **Kagloryuak R.**: *Edlund & Argus 12829* (CAN).

***Eriophorum callitrix*** Cham., Figs 19F, 20B–Arctic cottongrass | Asian (N)–amphi-Beringian–North American (N)

Previously recorded from Byron B., Cambridge Bay, Mt. Bumpus, Namaycush L., Richard Collinson Inl., Tahiryuaq, Tahoe L., Ulukhaktok and Washburn L. (Porsild 1955, 1957, 1964, Porsild and Cody 1980, Aiken et al. 2007). A collection (*Edlund & Argus 12829*) mapped from east of the head of Prince Albert S. (Aiken et al. 2007) has been redetermined as *E. brachyantherum*. Thannheiser et al. (2001) additionally recorded it from Johansen B. (conf.), the head of Minto Inl. (conf.) and Surrey L. Newly recorded from “30-Mile Cr.”, Colville Mts., Greiner L., Kuujjua R., Oterkvik Pt. and “Trunsky L.” Elsewhere in the Canadian Arctic recorded from Baffin, Banks, Devon, Somerset and Southampton islands and mainland sites (Porsild and Cody 1980, Cody et al. 1989, Korol 1992, Gould and Walker 1997, Aiken et al. 2007, Saarela et al. 2013, Saarela et al. 2017b).

**NORTHWEST TERRITORIES. Kuujjua R.**: *Gillespie et al. 9981* (CAN, MT, O), *9899* (CAN, mixed with *E. brachyantherum*), *9772b* (CAN, O, WIN). **Minto Inl. (head)**: *Edlund 174* (CAN, 2 sheets), *Gillespie et al. 10103* (CAN, MT, O, WIN), *10288* (ALA, CAN, O). **Richard Collinson Inl.**: *Edlund 671* (CAN). **Tahiryuaq**: *Edlund 392* (CAN). **Ulukhaktok**: *Edlund 824* (CAN), *Porsild* 17257 (ALTA, CAN), 17258 (CAN). **NUNAVUT. “30-Mile Cr.**”: *Bennett et al. 14-0344* (CAN). **Byron B.**: *Dushenko 15* (UVIC). **Cambridge Bay**: *Stephens 1201* (CAN, KSTC). **Colville Mts.**: *Gillespie et al. 7760* (ALA, CAN, O). **Greiner L.**: *Ponomarenko VI-106* (CAN), **Mt. Bumpus**: *Edlund 190*, *269* (CAN). **Namaycush L.**: *Edlund 168* (CAN). **Oterkvik Pt.**: *Gillespie et al. 7477* (ALA, BABY, CAN, MT, O). **Sinclair Cr.**: *Gillespie et al. 8247* (ALA, BABY, CAN, MT, O). **Tahoe L.**: *Porsild 17451* (CAN). “**Trunsky L.**”: *Bennett et al. 14-0403* (CAN). **Washburn L.**: *Oldenburg 46-2230* (CAN), *Porsild 17456* (CAN).

***Eriophorum russeolum*** subsp. ***albidum*** (F.Nyl.) Väre (E. russeolum subsp. leiocarpum M.S.Novos. *nom. illeg.*), Fig. 19G–Smooth-fruited russet cottongrass | Asian (N/C)–amphi-Beringian–North American (N)

Newly recorded from Victoria Island based on a single collection gathered in the Greiner L. watershed ca. 45 km ESE of Cambridge Bay, where it grew in a lake shore fen in a *Carex aquatilis–Drepanocladus* community. Elsewhere in the Canadian Arctic recorded from Baffin, Banks and Bylot islands and mainland sites (Porsild and Cody 1980, Cayouette 2004, Aiken et al. 2007, Saarela et al. 2013).

**NUNAVUT**. **Greiner L.**: *Ponomarenko VI-126* (CAN).

***Eriophorum scheuchzeri*** Hoppe

The collections listed below have not been determined to subspecies and are not mapped. Thannheiser et al. (2001) recorded the species from Minto Inl., Richardson I. and Johansen and Hadley B.

**NORTHWEST TERRITORIES. Ulukhaktok**: *Bliss s.n.* (ALTA, 2 sheets). **NUNAVUT. Cambridge Bay**: *Bennett 13-0220* (BABY, chars), *Bennett et al. 14-0310* (UBC), *Smith & Sweatman 42* (MT, US). **Tuktu R.**: *Gould s.n.* (ALA).

***Eriophorum scheuchzeri*** subsp. ***arcticum*** M.S.Novos., Figs 19H, 20C, D–Scheuchzer’s cottongrass | Circumpolar

Previously recorded from Cambridge Bay, Namaycush L., Storkerson P., Tahiryuaq and Ulukhaktok (Cayouette 2004, Aiken et al. 2007). Newly recorded from Boot Inl., C. Wollaston, Ferguson L., Greiner L., Hadley B., Johansen B., Mt. Bumpus, “Oldenburg L.”, Oterkvik Pt. and Sinclair Cr. Elsewhere in the Canadian Arctic recorded from Axel Heiberg, Baffin, Banks, Coast, Devon, Ellesmere, Mansel, Melville, Nottingham, Prince Charles, Prince Patrick, Resolution, Somerset and Southampton islands (Cayouette 2004, Aiken et al. 2007) and mainland sites (Cayouette 2004, Saarela et al. 2013, Saarela et al. 2017b).

**NORTHWEST TERRITORIES. Boot Inl.**: *Gillespie et al. 9561* (ALA, CAN, O). **C. Wollaston**: *Edlund 63* (CAN). **Kuujjua R.**: *Dutilly 19798* (DAO), *Gillespie et al. 9857* (ALA, CAN, O). **Minto Inl. (head)**: *Gillespie et al. 10035b* (CAN, O, WIN), *9861* (ALA, CAN, MT, O). “**Oldenburg L.**”: *Oldenburg 45-1374* (CAN). **Tahiryuaq**: *Edlund 388* (CAN). **Ulukhaktok**: *Edlund 498* (CAN), *Gray & Gibbard 32* (DAO). **NUNAVUT. Cambridge Bay**: *Gillespie et al. 8407* (ALA, CAN, O), *Polunin s.n.* (CAN, 2 sheets), *Stephens 1036* (CAN, KSTC), *Sweatman & Smith 11A* (DAO). **Ferguson L. [Tahiryuaq**]: *Hainault 1976* (DAO), *Jones 4* (DAO). **Greiner L.**: *Ponomarenko VI-187*, *VI-318* (CAN). **Hadley B.**: *Edlund 65*, *145* (CAN). **Johansen B.**: *Gillespie et al. 7911* (CAN). **Mt. Bumpus**: *Edlund 280* (CAN). **Namaycush L.**: *Edlund 131*, *136*, *28* (CAN). **Oterkvik Pt.**: *Gillespie et al. 7477* (ALA, BABY, CAN, MT, O), *7554* (CAN, O), *7785* (ALA, CAN, O). **Sinclair Cr.**: *Gillespie et al. 8298* (ALA, BABY, CAN, MT, O), *Gillespie et al. 8246* (ALA, CAN, O). **Storkerson P.**: *Edlund 189*, *288*, *293* (CAN).

***Eriophorum scheuchzeri*** Hoppe subsp. ***scheuchzeri***, Fig. 19I–Scheuchzer’s cottongrass | Circumpolar–alpine

Previously recorded from Collinson P., the head of Minto Inl., a site SE of the head of Prince Albert S., Richard Collinson Inl. and Storkerson P. (Aiken et al. 2007). Newly recorded from Cambridge Bay and Namaycush L. Elsewhere in the Canadian Arctic recorded from Axel Heiberg, Baffin, Banks, Coats, Cornwallis, Devon, Ellesmere, King William, Melville, Nottingham, Prince of Wales, Prince Charles, Prince Patrick, Somerset and Southampton islands and mainland sites (Cayouette 2004, Aiken et al. 2007).

**NORTHWEST TERRITORIES. Minto Inl. (head)**: *Edlund 165* (CAN). **Richard Collinson Inl.**: *P. Jenness 11* (CAN), *Stretton 211* (DAO). **NUNAVUT. Cambridge Bay**: *Oldenburg 44*-*890B* (CAN). **Collinson P.**: *Edlund & Argus 12768* (CAN). **Namaycush L.**: *Edlund & Roncato-Spencer 22* (CAN). **Prince Albert S. (head)**: *Edlund 91* (CAN). **Storkerson P.**: *Edlund 310* (CAN).

**Figure 21.**
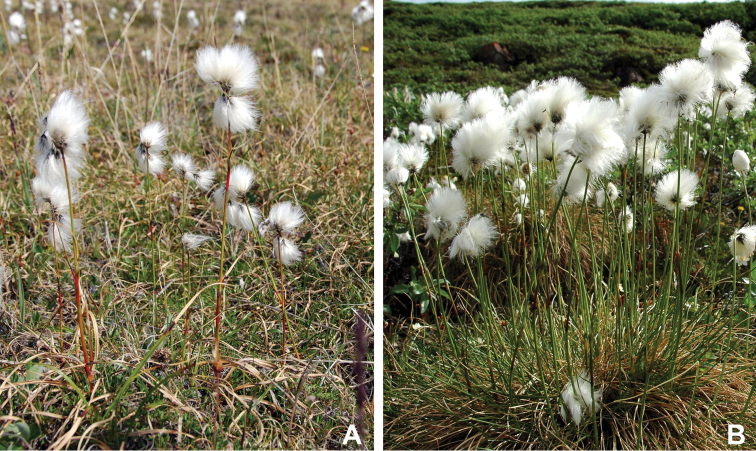
**A***Eriophorum triste* habit **B**Eriophorum vaginatum subsp. vaginatum habit, *Gillespie et al. 7948*. Photo **A** by J.M. Saarela and **B** by R.D. Bull.

***Eriophorum triste*** (Th.Fr.) Hadač *&* Á.Löve (E. angustifolium subsp. triste (Th.Fr.) Hultén), Figs 19J, 21A–Tall cottongrass | Amphi-Beringian (E)–North American (N)–amphi-Atlantic (W)

Previously recorded from Cambridge Bay, the head of Minto Inl., Mt. Bumpus, Namaycush L. and Ulukhaktok. Thannheiser et al. (2001) additionally recorded it from Johansen B. (conf.), Hadley B. (conf.), Surrey L. and Wellington B. Newly recorded from Boot Inl., Colville Mts., Ferguson L., Greiner L., Kuujjua R., Natkusiak P., “Oldenburg L.”, the head of Prince Albert S., Storkerson P., Walker B., Washburn L. and Wollaston P. Widespread across the Canadian Arctic Archipelago and across the mainland (Porsild and Cody 1980, Cody and Reading 2005, Aiken et al. 2007, Saarela et al. 2013, Saarela et al. 2017b).

**NORTHWEST TERRITORIES. Boot Inl.**: *Gillespie et al. 9553* (ALA, CAN, O). **Kuujjua R.**: *Edlund 641* (CAN), *Gillespie et al. 9772a* (ALA, CAN, MT, O). **Minto Inl. (head)**: *Edlund* 143 (CAN), *Gillespie et al. 9459* (ALA, CAN, WIN), *Gillespie et al. 10287* (CAN, O). **Natkusiak P.**: *Edlund 158* (CAN). “**Oldenburg L.**”: *Oldenburg 45-1376* (CAN). **Prince Albert S. (head)**: *Bezener 45* (DAO), *Weerstra 28*, *29* (DAO). **Ulukhaktok**: *Dutilly 18651* (US), *Edlund 445* (CAN), *Oldenburg 42-83A*, *45-1587* (CAN), *Saarela & Bull 1505* (CAN, O), *Svoboda 745027* (UBC), **Walker B.**: *Oldenburg 45-1442* (CAN). **NUNAVUT. Cambridge Bay**: *Porsild 21607* (CAN), *Stephens 964* (CAN, KANU), *1183* (CAN, KANU, KSTC), *Washburn 14* (CAN). **Colville Mts.**: *Gillespie et al. 7759* (ALA, BABY, CAN, MT, O). **Eastern Victoria I.**: *Lee & Kittle s.n.* (DAO). **Falaise B.**: *Eriksen et al. 966* (ALA). **Ferguson L. [Tahiryuaq**]: *Hainault 1974*, *2070*, *2137* (DAO). **Greiner L.**: *Ponomarenko VI-227* (CAN). **Hadley B.**: *Edlund 37*, *102* (CAN). **Johansen B.**: *Gillespie et al. 7944* (ALA, CAN, MT, O). **Mt. Bumpus**: *Edlund 189* (CAN). **Ovayok TP**: *Gould s.n.* (ALA), *Stephens 864* (CAN, KANU). **Namaycush L.**: *Edlund & Roncato-Spencer 19*, *55*, *63* (CAN). **Oterkvik Pt.**: *Gillespie et al. 7480* (ALA, ALTA, BABY, CAN, MT, O, UBC, US). **Sinclair Cr.**: *Gillespie et al. 8341* (ALA, BABY, CAN, MT, O). **Storkerson P.**: *Edlund 174* (CAN). “**Trunsky L.**”: *Bennett et al. 14-0402* (BABY, CAN). **Washburn L.**: *Oldenburg 46-2231* (CAN). **Wollaston P.**: *D. Jenness 659* (CAN).

***Eriophorum vaginatum*** L. subsp. ***vaginatum***, Figs 19K, 21B–Sheathed cottongrass | European–Asian–amphi-Beringian–North American (NW)

Previously recorded from Cambridge Bay, “Long L.”, the head of Minto Inl., the north side of Prince Albert S. (Porsild obs.), Tahoe L. (Porsild obs.) and Ulukhaktok (Porsild 1955, 1957, 1964, Porsild and Cody 1980, Aiken et al. 2007). Newly recorded from Boot Inl., Ferguson L., Kuujjua R., Johansen B. and Richard Collinson Inl. Elsewhere in the Canadian Arctic recorded from Banks and Melville islands and mainland sites (Porsild and Cody 1980, Cody et al. 1984a, Cody and Reading 2005, Aiken et al. 2007, Saarela et al. 2013, Saarela et al. 2017b). In the eastern Canadian Arctic this taxon is replaced by E. vaginatum subsp. spissum (Fernald) Hultén.

**NORTHWEST TERRITORIES. Boot Inl.**: *Gillespie et al. 9547* (CAN, MT, O). **Kuujjua R.**: *Gillespie et al. 9768* (ALA, ari, CAN, MT, O, UBC). **Minto Inl. (head)**: *Gillespie et al. 10033* (ALA, ari, CAN, O, UBC, US), *Porsild 17371* (CAN). **Richard Collinson Inl.**: *Edlund 546* (CAN). **Ulukhaktok**: *Dutilly 18650* (CAN, US), *Edlund 712* (CAN), *Porsild 17259* (CAN). **NUNAVUT. Cambridge Bay**: *Bennett et al. 13-0301* (CAN, chars). **Ferguson L. [Tahiryuaq**]: *Hainault 1957* (DAO). **Johansen B.**: *Gillespie et al. 7948* (ALA, ALTA, BABY, CAN, MT, O, UBC, US), *7949* (ALA, ALTA, BABY, CAN, MT, O, UBC, US). “**Long L.**”: *Lambert s.n.* (CAN, 2 sheets).

### Poaceae [17/38/44]

**Key to Poaceae [adapted from Barkworth (2007b)]**

**Table:** 

1	Spikelets sessile, inflorescences spikes or spikelike racemes	**2**
–	Spikelets pedicellate, inflorescences panicles or racemes	**5**
2	Distal portion of glumes hyaline; upper lemma margin hyaline; palea keels each with 2 awns	***Pleuropogon sabinei***
–	Glumes and lemmas membranous or firm, not hyaline; palea keels without awns	**3**
3	Spikelets edgewise to rachis; most spikelets with 1 glume, only the terminal spikelet with 2 glumes	***Lolium perenne***
–	Spikelets broadside to the rachis; all spikelets with 2 glumes	**4**
4	Spikes usually with 1 spikelet per node, occasionally with 2 at the lower nodes; glumes oblanceolate or obovate, 4–8 mm, glabrous or hairy, hairs 0.3–0.5 mm; lemmas 7–11 mm	***Elymus alaskanus***
–	Spikes with 2 spikelets per node; glumes lanceolate, 9–34 mm, strigillose, pilose or villous; lemmas 11–20 mm	**Leymus mollis subsp. villosissimus**
5	Spikelets 1-flowered (rarely 2-flowered in *Arctagrostis latifolia*)	**6**
–	Spikelets 2–many-flowered	**9**
6	Lemmas awned; glumes equalling or exceeding the lemmas	**7**
–	Lemmas unawned or mucronate; glumes shorter than the lemmas	**8**
7	Glumes densely pilose, connate in lower 1/8; rachilla not prolonged beyond the base of the distal floret; calluses glabrous; disarticulating below the glumes	***Alopecurus borealis***
–	Glumes glabrous or scabrous, free; rachilla prolonged beyond the base of the distal floret; calluses hairy; disarticulating above the glumes	*** Calamagrostis ***
8	Plants rhizomatous, usually coarse and robust; culms erect, 10–150 cm tall; blades 1–36 cm × 1.5–15 mm; ligules 2–7(–15) mm; inflorescences 2.5–35(–44) cm; spikelets 3–6.5 mm; glumes not caducous	**Arctagrostis latifolia subsp. latifolia**
–	Plants cespitose or mat–like, diminutive; culms erect or procumbent, (2–)3.5–15 cm tall; blades 0.6–2.8 cm × 1.2–3 mm; ligules 0.3–1(–1.6) mm; inflorescences (0.5–)1–2(–3) cm; spikelets (1–)1.4–1.8 mm; glumes caducous	***Phippsia algida***
9	Spikelets with 2 sterile florets below a bisexual floret; fresh leaves sweet-smelling when crushed	*** Anthoxanthum ***
–	Spikelets with bisexual florets, proximal sterile florets lacking; fresh leaves not sweet-smelling when crushed	**10**
10	One or both glumes exceeding the lowest floret, sometimes exceeding the distal floret	**11**
–	Both glumes shorter than or subequal to the lowest floret	**14**
11	Leaf sheaths closed for at least 1/2 their length	**12**
–	Leaf sheaths open for most of their length	**13**
12	Lemma apices obtuse; paleas subequal to the lemmas; glumes 1.5–4(–5) mm	***Arctophila fulva***
–	Lemma apices acute to acuminate; paleas shorter than the lemmas; glumes 4–8.5(–9) mm	***Dupontia fisheri***
13	Lemma apices truncate, erose to 2–4-toothed; awns arising at or below midlength of lemma; spikelets shiny	*** Deschampsia ***
–	Lemma apices acute, bifid; awns arising above midlength of lemma; spikelets not shiny	***Trisetum spicatum***
14	Lower lemmas with awns longer than 2 mm	**15**
–	Lower lemmas unawned, mucronate, or with awns up to 2 mm	**17**
15	Awns bent, arising from the upper 1/3 of the lemma	***Trisetum spicatum***
–	Awns straight, arising terminally or subterminally	**16**
16	Lemmas 9–16 mm; cauline leaf sheaths closed for at least 3/4 their length at maturity; spikelets 16–32 mm	***Bromus pumpellianus***
–	Lemmas 2.5–7.5(–8) mm; cauline leaf sheaths open, or closed for ca. ½ or less their length at maturity; spikelets 3–14.5 mm	*** Festuca ***
17	Sheaths closed for more than 1/2 their length; plants aquatic	*** Arctophila ***
–	Sheaths closed for 1/10 to ½ their length; plants not aquatic	**18**
18	Lemma veins more or less parallel distally, conspicuous; glumes usually distinctly shorter than the lowest lemma in the spikelets, sometimes only slightly shorter (*P. vahliana*), leaf blade apices not prow–tipped	*** Puccinellia ***
–	Lemmas veins converging distally, usually inconspicuous, sometimes conspicuous; glumes slightly shorter than the lowest lemma; leaf blade apices prow-tipped	*** Poa ***


### *Alopecurus* L. [1]

**Figure 22.**
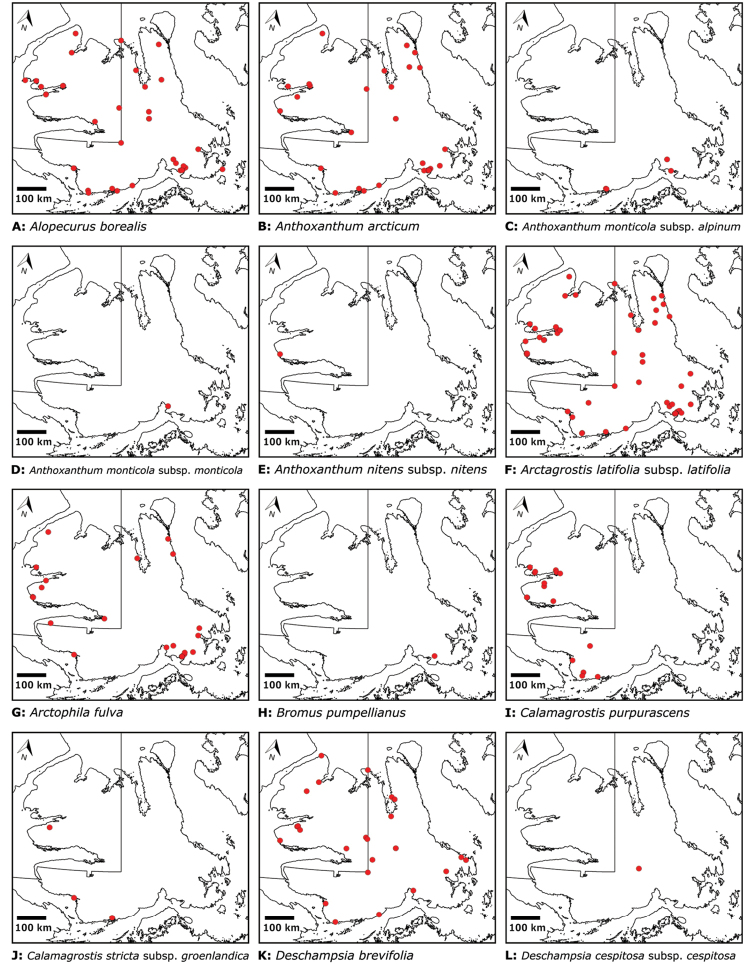
Species distribution maps. Poaceae: **A***Alopecurus borealis***B***Anthoxanthum arcticum***C**Anthoxanthum monticola subsp. alpinum**D**Anthoxanthum monticola subsp. monticola**E**Anthoxanthum nitens subsp. nitens**F**Arctagrostis latifolia subsp. latifolia**G***Arctophila fulva***H***Bromus pumpellianus***I***Calamagrostis purpurascens***J**Calamagrostis stricta subsp. groenlandica**K***Deschampsia brevifolia***L**Deschampsia cespitosa subsp. cespitosa.

**Figure 23.**
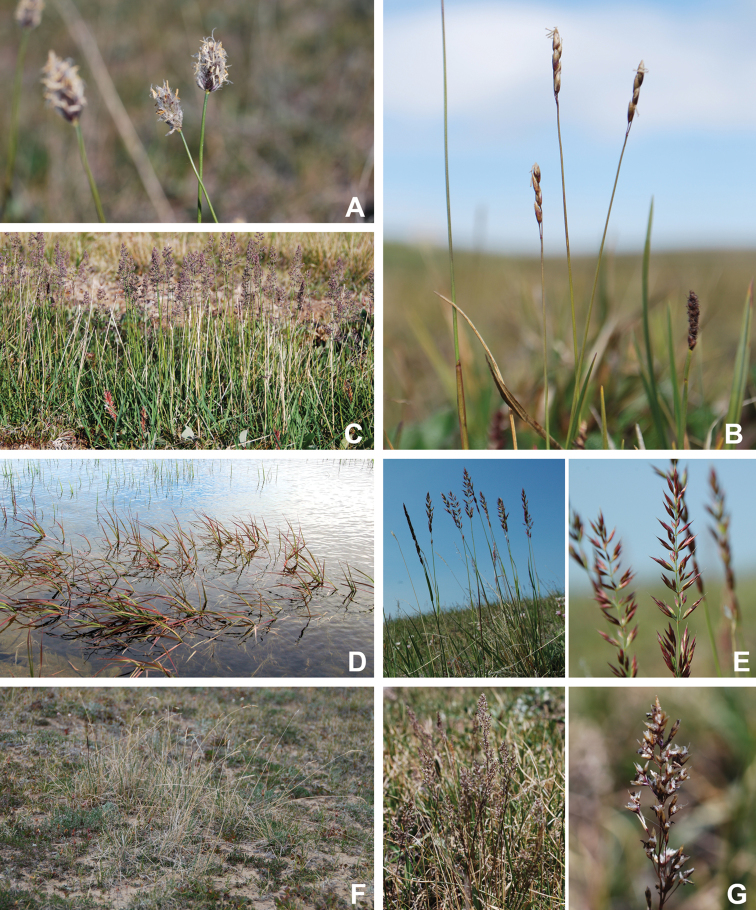
**A***Alopecurus borealis* inflorescences, *Gillespie et al. 9650***B***Anthoxanthum arcticum* habit **C**Arctagrostis latifolia subsp. latifolia habit, *Gillespie et al. 9983***D***Arctophila fulva* habit, *Gillespie et al. 8441***E***Calamagrostis purpurascens* habit (left), inflorescence (right), *Gillespie et al. 8115***F**Calamagrostis stricta subsp. groenlandica habit, *Gillespie et al. 9908***G***Deschampsia brevifolia* habit (left), inflorescence (right), *Gillespie et al. 9856*. Photos **A**, **C**, **F**, **G** by J.M. Saarela, **B** by B.A. Bennett and **D**, **E** by R.D. Bull.

***Alopecurus borealis*** Trin. (*A. alpinus* Sm., *nom. illeg.*, *A. magellanicus* Lam. *s.l.*), Figs 22A, 23A–Alpine foxtail | Circumpolar-alpine

Previously recorded from Berkeley Pt., Cambridge Bay, Natkusiak P., Storkerson P., the head of Minto Inl., the head of Prince Albert S., Read I., Tahiryuaq, Ulukhaktok (Porsild obs.) and northwestern Wollaston P. (Porsild obs.) (Porsild 1955, 1957, 1964, Porsild and Cody 1980, Aiken et al. 2007). Thannheiser et al. (2001) additionally reported it from Hadley B. (conf.), Johansen B. (conf.), Wellington B. and Surrey L. Newly recorded from Albert Edward B., Boot Inl., De Haven Pt., Kuujjua R., Ferguson L., Greiner L., Mt. Pelly, “Oldenburg L.”, Oterkvik Pt., Richard Collinson Inl., Sinclair Cr., Walker B. and Washburn L. Widespread throughout the Canadian Arctic (Porsild and Cody 1980, Korol 1992, Aiken et al. 2007, Saarela et al. 2017b).

**NORTHWEST TERRITORIES. Berkeley Pt.**: *Stretton 88* (DAO). **Boot Inl.**: *Gillespie et al. 9650* (CAN, US). **Minto Inl. (head)**: *Edlund 151* (CAN), *Gillespie et al. 10145* (CAN, O). **Kuujjua R.**: *Dutilly 18813* (DAO, QFA), *Gillespie et al. 9947* (CAN). **Prince Albert S. (head)**: *Edlund 97*, *374* (CAN). “**Oldenburg L.**”: *Oldenburg 45-1365* (CAN). **Richard Collinson Inl.**: *Edlund 705* (CAN). **Tahiryuaq**: *Edlund 552* (CAN). **Walker B.**: *Oldenburg 45*-1454 (CAN). **NUNAVUT. Albert Edward B.**: *Ponomarenko VI-336B* (CAN). **Cambridge Bay**: *Bennett et al. 13-0194* (BABY, chars, od), *Boulva 65*-*44* (QFA), *Dutilly 28092* (QFA), *Edlund & Argus 12699* (CAN), *Gillespie et al. 8403* (ALA, CAN, MT), *Oldenburg 44*-*901* (CAN), *Polunin s.n.* (CAN), *Stephens 1147* (CAN), *Sweatman & Smith 12* (DAO), *Washburn 30* (CAN). **De Haven Pt.**: *Bernard s.n.* (QFA). **Ferguson L. [Tahiryuaq**]: *Ducruc s.n.* (DAO), *Hainault 2031* (DAO). **Greiner L.**: *Ponomarenko VI-296*, *VI-306***(CAN). Hadley B.**: *Edlund 34*, *115* (CAN). **Johansen B.**: *Gillespie et al. 7838* (ALA, CAN, O). **Ovayok TP**: *Gould s.n.* (ALA). **Murray Pt.**: *Gillespie et al. 8210* (CAN, O). **Namaycush L.**: *Edlund 10*, *s.n.* (CAN), *Edlund & Roncato-Spencer 48*, *61* (CAN), *Roncato-Spencer 16* (CAN). **Natkusiak P.**: *Edlund 75*, *126* (CAN). **Oterkvik Pt.**: *Gillespie et al. 7568* (ALA, CAN, MT, O, US), *7615* (ALA, CAN, MT, O). **Read I.**: *Oldenburg 42-483*, *43-947* (CAN), *Porsild 17186* (CAN), *Ross 11* (ALTA). **Sinclair Cr.**: *Gillespie et al. 8236* (ALA, CAN, O). **Storkerson P.**: *Edlund 184*, *212* (CAN). **Washburn L.**: *Oldenburg 46-2188* (CAN).

### *Anthoxanthum* L. [3/4]

**Key to *Anthoxanthum* [adapted from Allred and Barkworth (2007)]**

**Table:** 

1	Staminate lemmas awned, the awns of the upper staminate florets 4.5–10.5 mm; plants densely to loosely tufted, with rhizomes rarely more than 2 cm (*A. monticola*)	**2**
–	Staminate lemmas unawned or with an awn no more than 1 mm; plants long-rhizomatous	**3**
2	Awns of the upper staminate florets 5–10.5 mm, attached from near the base to about midlength; awn usually strongly geniculate, the lower portion usually twisted, with 2–4 gyres	**A. monticola subsp. alpinum**
–	Awns of the upper staminate florets 4.5–7 mm, attached at or above midlength, not or only weakly geniculate, the lower portion not twisted or twisted with 1–2 gyres	**A. monticola subsp. monticola**
3	Panicles spikelike, 1–3(–4.5) × 0.3–0.5 cm, with 1–2 spikelets per branch; rhizomes 0.3–1 mm thick	***A. arcticum***
–	Panicles open, pyramidal, (2–)4–9(–12.5) × (1.5–)2–5(–7) cm, the longer branches usually with 3 or more spikelets; rhizomes 0.7–2 mm thick	***A. nitens***

***Anthoxanthum arcticum*** Veldkamp (*Hierochloe pauciflora* R.Br.), Figs 22B, 23B–Arctic sweetgrass | Asian (N)–amphi-Beringian–North American (N)

Previously known from Cambridge Bay, the head of Minto Inl., the head of Prince Albert S., Wollaston P. (Porsild obs.), Storkerson P., Ulukhaktok (Porsild obs., conf.), and just north of Collinson P. (Porsild 1955, 1957, 1964, Porsild and Cody 1980, Aiken et al. 2007). We have not seen a voucher from a site mapped in the Johansen B. area by Porsild and Cody (1980) and Aiken et al. (2007), but our collection from the vicinity confirms its presence there. Thannheiser et al. (2001) additionally reported it from Hadley B. (conf.), Richardson I. and Wellington B. Newly recorded from Albert Edward B., Boot Inl., Ferguson L., Greiner L., Kuujjua R. (including a site near its headwaters near the territorial border), “Oldenburg L.”, Murray Pt., Oterkvik Pt., Read I. and Sinclair Cr. Elsewhere in the Canadian Arctic recorded from Banks, Baffin, Bathurst, Coats, Cornwallis, Devon, Ellesmere, Melville, Prince Charles, Prince Patrick, Somerset and Southampton islands and scattered mainland sites where the species reaches its southern limit (Porsild and Cody 1980, Korol 1992, Gould and Walker 1997, Aiken et al. 2007, Allred and Barkworth 2007, Saarela et al. 2013, Saarela et al. 2017b).

**NORTHWEST TERRITORIES. Boot Inl.**: *Gillespie et al. 9655* (CAN, O). **Kuujjua R.**: *Edlund 548* (CAN, US), *Gillespie et al. 9788* (ari, CAN, O). **Minto Inl. (head)**: *Edlund 118* (CAN), *Gillespie et al. 10038* (CAN, MT, O, US). “**Oldenburg L.**”: *Oldenburg 45-1369* (CAN). **Prince Albert S.**: *Porsild 17432* (CAN). **Ulukhaktok**: *Edlund 503* (CAN, US), *769* (CAN), *Saarela & Bull 1490* (ALA, CAN, O). **NUNAVUT. Albert Edward B.**: *Ponomarenko VI-247* (CAN). **Cambridge Bay**: *Bennett et al. 13-0172* (BABY, UBC, chars, od), *Consaul & Gillespie 1101* (CAN), *Edlund & Argus 12685* (CAN), *Gillespie et al. 8503* (ALA, CAN, MT, O), *Oldenburg 44*-*965* (CAN), *Polunin s.n.* (CAN), *Ponomarenko VI-091* (CAN), *Porsild 17462* (CAN), *21588* (CAN), *Stephens 945*, *1020* (CAN). **Ferguson L. [Tahiryuaq**]: *Ducruc s.n.* (QFA). **Greiner L.**: *Ponomarenko VI-186* (CAN). **Hadley B.**: *Edlund 127*, *s.n.* (CAN). **Johansen B.**: *Gillespie et al. 7905* (ALA, CAN, O). **Murray Pt.**: *Gillespie et al. 8191* (ALA, CAN, O). **Namaycush L.**: *Edlund 26*, *127* (CAN). **Oterkvik Pt.**: *Gillespie et al. 7614* (ALA, CAN, MT, O). **Read I.**: *Oldenburg 43-903* (CAN). **Sinclair Cr.**: *Gillespie et al. 8307* (ALA, BABY, CAN, MT, O, US). **Storkerson P.**: *Edlund 235*, *286*, *303* (CAN).

***Anthoxanthum monticola*** subsp. ***alpinum*** (Sw. ex Willd.) Soreng (*Hierochloe alpina* (Sw.) Roem. *&* Schult.), Fig. 22C–Bent-awned alpine sweet grass | Circumpolar-alpine

This acidophile was previously recorded from Cambridge Bay, Ulukhaktok and a site on south-central Victoria I. (Porsild and Cody 1980, Aiken et al. 2007). We have only located the voucher for the Cambridge Bay record, collected on terraces 2 km north of Long Point, west of the hamlet. The south-central record may be based on a John Rae collection from the south coast of Victoria I. Thannheiser et al. (2001) additionally recorded it from Johansen B. (conf.) and Richardson I. Newly reported from Ferguson L., where in 1962 it was found along the north shore of the west end of the lake, where acidic substrates are present. Our two collections from Johansen B. were gathered in rocky hills just south of a small lake out of which the Nakoyoktok R. flows and among rocks near the river outflow. Elsewhere in the Canadian Arctic recorded from northern Banks, Baffin, Cornwallis, Devon, Ellesmere, Melville, Prince Patrick, Somerset and Southampton islands, and across the mainland (Porsild and Cody 1980, Cody et al. 1989, Korol 1992, Aiken et al. 2007, Saarela et al. 2013, Bennett 2015, Saarela et al. 2017b).

**NUNAVUT. Cambridge Bay**: *Consaul & Gillespie 1135* (CAN). **Ferguson L. [Tahiryuaq**]: *Hainault 1953* (DAO, UAC). **Johansen B.**: *Gillespie et al. 7985* (ALA, ALTA, BABY, CAN, MT, O, US), *7846* (ALA, CAN, MT, O).

***Anthoxanthum monticola*** (Bigelow) Veldkamp subsp. ***monticola*** (A. monticola subsp. orthanthum (T.J.Sørensen) G.C.Tucker, *Hierochloë orthantha* T.J.Sørensen), Fig. 22D–Alpine sweetgrass | North American (NE)

Newly recorded for Victoria I., and the first record for the Canadian Arctic Archipelago. The taxon was collected twice in the vicinity of Ferguson L. in 2014. At one site it was growing in dry *Dryas integrifolia*–*Carex rupestris*–*Cassiope tetragona* dominated tundra over limestone morainal till modified with marble and some granite, associated with *Salix arctica*, *Carex vaginata*, C. fuliginosa subsp. misandra, *Pedicularis capitata*, and *Vaccinium uliginosum*. At the other site it was growing in a nutrient enriched crevice of a marbled limestone ridge, with *Saxifraga tricuspidata*, *Potentilla*, *Luzula confusa*, *Carex rupestris*, *Sabulina rubella*, and *Draba cinerea*. This is a northeastern North American taxon that, aside from the collection reported here, which is a considerable range extension, reaches the eastern side of Hudson Bay (Allred and Barkworth 2007).

**NUNAVUT. Ferguson L. [Tahiryuaq**]: *Bennett et al. 14-0410* (CAN), *14-0415* (BABY, chars, DAO, od).

***Anthoxanthum nitens*** (Weber) Y.Schouten *&* Veldkamp subsp. ***nitens*** (*Hierochloe odorata* (L.) Wahlenb.), Fig. 22E–Vanilla sweetgrass | Amphi-Atlantic–European–Asian (W)

Recorded from Ulukhaktok by Porsild (1955) and mapped there in subsequent treatments (Porsild 1957, 1964, Porsild and Cody 1980). This is the only record of the species from Victoria I., the Canadian Arctic Archipelago and the Northwest Territories of which we are aware. Aiken et al. (2007) did not treat the taxon for the Canadian Arctic Archipelago. Some authors, such as Porsild and Cody (1980), included *Anthoxanthum hirtum* (syns. *Hierochloe hirta* (Schrank) Borbás, H. odorata subsp. hirta (Schrank) Tzvelev) in their concept of *A. nitens* (syn. *H. odorata*), while others recognized them as distinct species (Weimarck 1971, Allred and Barkworth 2007, Elven et al. 2011); we follow the latter circumscription here. Anthoxanthum nitens subsp. nitens is an amphi-Atlantic taxon that elsewhere reaches the Canadian Arctic in at least northern Labrador (Weimarck 1971) and the Belcher Islands (e.g., *Consaul et al.* 3787, CAN 600006). The Ulukhaktok record is far out of the typical range of the species.

**NORTHWEST TERRITORIES. Ulukhaktok**: *Porsild 17240* (CAN).

### *Arctagrostis* Griseb. [1]

***Arctagrostis latifolia*** (R.Br.) Griseb. subsp. ***latifolia***, Figs 22F, 23C–Polargrass, Arctic grass | Circumpolar-alpine

Previously recorded from Cambridge Bay, Greely Haven, the head of Minto Inl., N of a large lake in the Ekalluk River system about 90 km NNE of Cambridge Bay, Prince Albert S., Tahoe L., Ulukhaktok and Walker B. (Porsild 1955, 1957, 1964, Porsild and Cody 1980, Aiken and Lefkovitch 1990, Aiken et al. 2007). Thannheiser et al. (2001) additionally recorded it from Hadley B. (conf.), Johansen B. (conf.), Mt. Pelly (conf.), Richardson I., Surrey L. and Wellington B. Newly recorded from Boot Inl., south of Burns L., C. Wollaston, Clouston B., Ferguson L., Greiner L., Kuujjua R., Mt. Bumpus, Namaycush L., Natkusiak P., “Oldenburg L.”, Oterkvik Pt., Read I., Richard Collinson Inl., Sinclair Cr. and Washburn L. Widespread across the Canadian Arctic Archipelago and adjacent mainland (Porsild and Cody 1980, Cody et al. 1989, Aiken and Lefkovitch 1990, Korol 1992, Aiken et al. 2007, Saarela et al. 2013, Saarela et al. 2017b).

**NORTHWEST TERRITORIES. Boot Inl.**: *Gillespie et al. 9550* (ALA, CAN, MT, O). **Burns L. (S)**: *Edlund 61b*, *23*, *95* (CAN). **C. Wollaston**: *Edlund 56* (CAN). **Kuujjua R.**: *Dutilly 18814b* (DAO, QFA), *Gillespie et al. 9770* (CAN, MT, O), *9983* (ALA, CAN, O). **Minto Inl. (head)**: *Edlund* 172 (CAN), *Gillespie et al. 10268* (CAN, MT, O, US), *9486* (ALA, CAN, US), *10289* (CAN, O), *Porsild 17354* (CAN). “**Oldenburg L.**”: *Oldenburg 45-1366* (CAN). **Richard Collinson Inl.**: *Edlund 675* (CAN), *Stretton 209*, *210* (DAO). **Ulukhaktok**: *Dutilly 18648* (DAO, QFA), *Edlund 340*, *367A*, *472*, *473*, *826*, *870*, *884* (CAN), *Oldenburg 42-75*, *45-1589* (CAN), *Saarela & Bull 1476* (CAN, O), *Salokangas 25* (CAN, UBC). **Walker B.**: *Oldenburg 45-1449* (CAN), *Porsild 17482* (CAN). **NUNAVUT. Cambridge Bay**: *Bennett et al. 13-0197* (chars, od, V), *Beschel 13480* (CAN), *Edlund & Argus 12615*, *12636*, *12637* (CAN), *Fortier 10* (CAN), *Gillespie et al. 8373* (ALA, CAN, MT, O), *8402* (AKUR, ALA, CAN, MT, O, US), *8391* (CAN), *Gould s.n.* (ALA), *Porsild 21584* (CAN), *Oldenburg 44-884*, *44-894*, *44-896*, *44-963* (CAN), *Polunin s.n.* (CAN), *Saarela 5301* (CAN), *Smith & Sweatman 33* (CAN), *Smith & Sweatman 35* (DAO), *Stephens 1095* (CAN), *Sutton 856* (CAN). **Clouston B.**: *Gillespie et al. 7741* (ALA, CAN, MT, O). **Ekalluk R.**: *Edlund & Argus 12740* (CAN), *Lee & Kittle s.n.* (CAN). **Ferguson L. [Tahiryuaq**]: *Ducruc s.n.* (QFA), *Hainault 1873* (DAO), *2087* (DAO, UAC). **Greely Haven**: *Fortier 97* (CAN). **Greiner L.**: *Ponomarenko VI-229A* (CAN). **Hadley B.**: *Edlund 68*, *116*, *123* (CAN). **Johansen B.**: *Gillespie et al. 7875* (ALA, CAN, MT, O). “**Long L.**”: *Lambert s.n.* (CAN). **Mt. Bumpus**: *Edlund 195* (CAN). **Ovayok TP**: *Dutilly 28090* (CAN, QFA). **Namaycush L.**: *Edlund & Roncato-Spencer 66* (CAN). **Natkusiak P.**: *Edlund 106*, *161* (CAN). **Oterkvik Pt.**: *Gillespie et al. 7544* (ALA, CAN, O), *7553* (CAN, O). **Read I.**: *Oldenburg 43-1012*, *43-1022*, *43-948* (CAN). **Sinclair Cr.**: *Gillespie et al. 8302* (ALA, CAN, MT, O). **Storkerson P.**: *Edlund 177*, *280*, *346* (CAN), *Stretton 227* (DAO). **Washburn L.**: *Edlund & Argus 12793* (CAN), *Oldenburg 46-2189*, *46-2192*, *46-2193* (CAN).

### *Arctophila* (Rupr.) Rupr. ex Andersson [1]

***Arctophila fulva*** (Trin.) Andersson (*Colpodium fulvum* (Trin.) Griseb.), Figs 22G, 23D–Pendent grass | Circumpolar

Previously recorded from Cambridge Bay, Ferguson L., Kuujjua R., the head of Prince Albert S., Storkerson P. and Ulukhaktok (Porsild 1955, 1957, 1964, Porsild and Cody 1980, Aiken et al. 2007). Thannheiser et al. (2001) additionally reported it from Johansen B., Mt. Pelly, Richardson I., Surrey L. and Wellington B. We have not seen a specimen supporting a record mapped by Aiken et al. (2007) from Albert Edward B., but the species is confirmed there based on a recent collection. Newly recorded from Greiner L., Prince Albert P., Read I., Walker B. and northwestern Wollaston P. Elsewhere in the Canadian Arctic recorded from Baffin, Banks, Coats, Eglinton, Melville, Prince Charles, Prince Patrick and Southampton islands and scattered mainland sites (Porsild and Cody 1980, Korol 1992, Aiken et al. 2007, Saarela et al. 2013, Saarela et al. 2017b).

**NORTHWEST TERRITORIES. SE of Armstrong Pt.**: *Edlund 588* (CAN). **Kuujjua R.**: *Dutilly 18805* (CAN, DAO, MT), *Edlund 628* (CAN). **Prince Albert S. (head)**: *Porsild 17430* (CAN). **Ulukhaktok**: *Edlund 512*, *515*, *756* (CAN), *Porsild 17237* (CAN). **Walker B.**: *Oldenburg 45-1447* (CAN). **NUNAVUT. Albert Edward B.**: *Ponomarenko VI-254* (CAN). **Cambridge Bay**: *Bennett 13-0225* (chars, od), *Dutilly 37148* (QFA), *Gillespie et al. 8419* (ALA, ALTA, BABY, CAN, MT, O, UBC, US), *8441* (ALA, CAN, O), *Oldenburg 44-894*, *44-951* (CAN), *Polunin s.n.* (CAN, 2 sheets), *Porsild 21585* (CAN), *Stephens 1125* (CAN). **Ferguson L. [Tahiryuaq**]: *Hainault 1931*, *2015* (DAO). **Greiner L.**: *Ponomarenko VI-188*, *VI-341A* (CAN). **Hadley B.**: *Edlund 66* (CAN). **Read I.**: *Oldenburg 43-1021* (CAN). **Storkerson P.**: *Edlund 291*, *301* (CAN). **Wollaston P.**: *Oldenburg 54*-*500* (CAN, QFA, UBC).

### *Bromus* L. [1]

***Bromus pumpellianus*** Scribn., Fig. 22H–Pumpelly’s brome | European (NE)–Asian (N/C)–amphi-Beringian–North American

Newly recorded for Victoria I., based on a collection from “Long L.” gathered in 1964. This record was not mentioned in subsequent treatments. Pavlick and Anderton (2007) mapped a collection on Victoria I. from the vicinity of C. Baring at the western end of the Wollaston P., for which we are not aware of a voucher specimen. In the Canadian Arctic this species has a western distribution, known from the mainland as far east as Bathurst Inl. and along the Kazan R. (Porsild and Cody 1980, Saarela et al. 2013, Bennett 2015, Saarela et al. 2017b). The “Long L.” collections extend the species’ range slightly to the northwest, with respect to known sites in the vicinity of Bathurst Inl., and is the only record from the Canadian Arctic Archipelago.

**NUNAVUT. “Long L.**”: *Lambert s.n.* (CAN) (Suppl. material 4).

### *Calamagrostis* Adans. [2]

**Key to *Calamagrostis* [adapted from Marr et al. (2007) and J.M. Saarela, unpubl. data]**

**Table:** 

1	Spikelets (4.5–)5.5–6.5(–8) mm; awns (4.5–)6–7(–9) mm, usually exserted beyond the margins of the glumes; callus 0.2–0.4(–0.6)× as long as the lemmas; leaf blade adaxial surface usually densely long-hairy, rarely sparsely hairy	***C. purpurascens***
–	Spikelets 2–2.5(–3) mm; awns 1.5–2.5 mm, equaling or exserted slightly beyond the margins of the glumes; callus hairs (0.5–)0.7–0.9× as long as the lemmas; leaf blade adaxial surface usually scabrous, rarely smooth, sometimes puberulent	**C. stricta subsp. groenlandica**

***Calamagrostis purpurascens*** R.Br. subsp. ***purpurascens***, Figs 22I, 23E–Purple reedgrass | Asian (NE)–amphi-Beringian–Cordilleran–North American–amphi-Atlantic (W)

Previously recorded from the head of Minto Inl., Mt. Bumpus and Ulukhaktok (Porsild 1955, 1957, 1964, Porsild and Cody 1980, Aiken et al. 2007). Newly recorded from Boot Inl., Clouston B., Johansen B., Kuujjua R., the north side of Prince Albert S., Oterkvik Pt. and Walker B. Elsewhere in the Canadian Arctic recorded from scattered sites on Baffin, Banks, Ellesmere and Melville islands, and scattered mainland sites (Porsild and Cody 1980, Cody and Reading 2005, Aiken et al. 2007, Saarela et al. 2013, Saarela et al. 2017b). There is a large distribution gap in the central Canadian Arctic Archipelago, including the eastern half of Victoria I. (Porsild and Cody 1980, Aiken et al. 2007).

**NORTHWEST TERRITORIES. Boot Inl.**: *Gillespie et al. 9543a* (ALA, ari, CAN, MT, O, UBC), *9542* (CAN), *9658* (ALA, CAN, O). **Kuujjua R.**: *Edlund 663* (CAN), *Gillespie et al. 9717* (ALA, ari, CAN, MT, O, UBC, US, WIN). **Minto Inl. (head)**: *Gillespie et al. 10015* (ari, CAN, MO, MT, UBC, US, WIN), *10158* (ALA, CAN, MT, US), *Porsild 17355* (CAN). **Prince Albert S. (N)**: *Edlund 446* (CAN). **Ulukhaktok**: *Edlund 284*, *341*, *486*, *845*, *852*, *883* (CAN), *Gray & Gibbard 42* (DAO), *Oldenburg 42-78* (CAN), *Porsild 17238* (CAN), *Saarela & Bull 1475* (ALA, CAN, US). **Walker B.**: *Oldenburg 45-1450* (CAN). **NUNAVUT. Clouston B.**: *Gillespie et al. 7740* (ALA, CAN, MT, O). **Johansen B.**: *Gillespie et al. 8115* (ALA, BABY, CAN, MT, O, US). **Mt. Bumpus**: *Edlund 233* (CAN). **Oterkvik Pt.**: *Gillespie et al. 7665* (CAN, O), *7809* (ALA, CAN, O)

***Calamagrostis stricta*** subsp. ***groenlandica*** (Schrank) Á.Löve (C. neglecta subsp. groenlandica (Schrank) Matuszk., C. stricta subsp. stricta s.l.), Figs 22J, 23F–Slim-stemmed reedgrass | Circumpolar

Not recorded from Victoria I. by Aiken et al. (2007) nor Porsild and Cody (1980), but reported from Ulukhaktok by Thannheiser et al. (2001). Newly recorded from Kuujjua R., Johansen B. and Read I. At the Kuujjua R. site, it grew on sandy tundra with *Arctous rubra*, *Astragalus alpinus*, *Dryas integrifolia* and *Physaria arctica*. Elsewhere in the Canadian Arctic recorded from Baffin, Banks, Melville and Prince Patrick islands (Aiken et al. 2007, Gillespie et al. 2015) and mainland sites (Saarela et al. 2017b).

**NORTHWEST TERRITORIES. Kuujjua R.**: *Gillespie et al. 9908* (CAN, O). **NUNAVUT. Johansen B.**: *Gillespie et al. 8052* (ALA, CAN, MT, O, US), *7996* (CAN, O), *8053* (CAN). **Read I.**: *Oldenburg 43-898*, *43-938*, *43-1020* (mixed with *Trisetum spicatum*) (CAN).

### *Deschampsia* [3]

**Key to *Deschampsia* [adapted from Barkworth (2007a)]**

**Table:** 

1	Panicles usually dense, oblong-ovate to narrowly cylindrical, branches usually stiff, erect to ascending, straight; spikelets strongly imbricate, often rather densely clustered on the ends of the branches, sometimes evenly distributed on the branches; glumes and lemmas usually dark purple proximally for over more than 1/2 their surface	***D. brevifolia***
–	Panicles usually open and pyramidal, sometimes closed and ovate; branches spreading, divergent or reflexed; spikelets usually not or only moderately imbricate, not in dense clusters at the ends of the branches; glumes and lemmas usually dark purple proximally over less than 1/2 their surface	**2**
2	Branches flexuous; basal blades with 3–5 ribs; all blades of the current year usually strongly involute and hairlike, 0.3–0.5(–0.8) in diameter; panicles 3.5–17 ×1.5–9 cm	***D. sukatschewii***
–	Branches usually strongly divergent, sometimes strongly ascending, straight to slightly flexuous; usually at least some blades flat and 1–4 mm wide, the majority folded or rolled and 0.5–1 mm in diameter; panicles 8–30 × 4–30 cm	***D. cespitosa***

***Deschampsia brevifolia*** R.Br. (D. cespitosa subsp. brevifolia (Griseb.) Tzvelev, *nom. illeg.*, D. cespitosa subsp. septentrionalis Chiapella), Figs 22K, 23G–Arctic hairgrass | Asian (N)–amphi-Beringian–North American (N)

Previously recorded from Minto Inl., Natkusiak P., Namaycush L. and Collinson P. (Porsild 1955, 1957, 1964, Porsild and Cody 1980, Aiken et al. 2007). Thannheiser et al. (2001) additionally recorded it from Hadley B. (conf.) and Richardson I. Newly recorded from Albert Edward B., south of Burns L., Kuujjua R., Peel Pt., Ulukhaktok, Clouston B., Hadley B., Oterkvik Pt., sites north, east and southeast of the head of Prince Albert S., an inland site on Prince Albert P., Richard Collinson Inl. and Sinclair Cr. Widespread throughout most of the Canadian Arctic Archipelago, but apparently rare on most of Baffin I. (Aiken et al. 2007), and known from a few mainland sites (Porsild and Cody 1980, Cody 1996a, Saarela et al. 2017b)

**NORTHWEST TERRITORIES. Burns L. (S)**: *Edlund 64*, *565* (CAN). **Kuujjua R.**: *Gillespie et al. 9856* (ALA, ALTA, ari, CAN, MOMT, O, UBC, US, UTCWIN, WTU), *9855* (CAN), *9973* (CAN), *9902* (ALA, ALTA, ari, CAN, MO, NFLD, MT, O, UBC, US, UTC, V, WIN, WTU), *9864* (CAN, O). **Peel Pt.**: *Edlund 425* (CAN). **Prince Albert P.**: *Oldenburg 54-666* (UBC). **Prince Albert S. (head)**: *Edlund 387* (CAN). **Richard Collinson Inl.**: *Edlund 767* (CAN). **Ulukhaktok**: *Edlund 367* (CAN, US). **NUNAVUT. Albert Edward B.**: *Edlund & Argus 12744* (CAN). **Clouston B.**: *Gillespie et al. 7745* (CAN). **Collinson P.**: *Edlund & Argus 12753*, *12767* (CAN). **Ferguson L. [Tahiryuaq**]: *Jones 6* (DAO). **Hadley B.**: *Edlund 132*, *235*, *316*, *332* (CAN). **Namaycush L.**: *Edlund 38*, *141*, *143* (CAN), *Edlund & Roncato-Spencer 50*, *70* (CAN). **Natkusiak P.**: *Edlund 111*, *122* (CAN). **Oterkvik Pt.**: *Gillespie et al. 7610* (ALA, ALTA, BABY, CAN, MT, O, UBC, US). **Prince Albert S. (head)**: *Edlund & Argus 12821* (CAN), *Edlund 101* (CAN). **Sinclair Cr.**: *Gillespie et al. 8309* (ALA, ALTA, BABY, CAN, MT, O, UBC, US).

***Deschampsia cespitosa*** (L.) P. Beauv. subsp. ***cespitosa***, Fig. 22L–Tufted hairgrass | Circumboreal

Newly recorded for Victoria I. and the Canadian Arctic Archipelago. The specimen was gathered at the NW end of Washburn L., growing in *Dryas* tundra on silty clay terraces.

**NUNAVUT. Washburn L.**: *Edlund & Argus 12796* (CAN).

**Figure 24.**
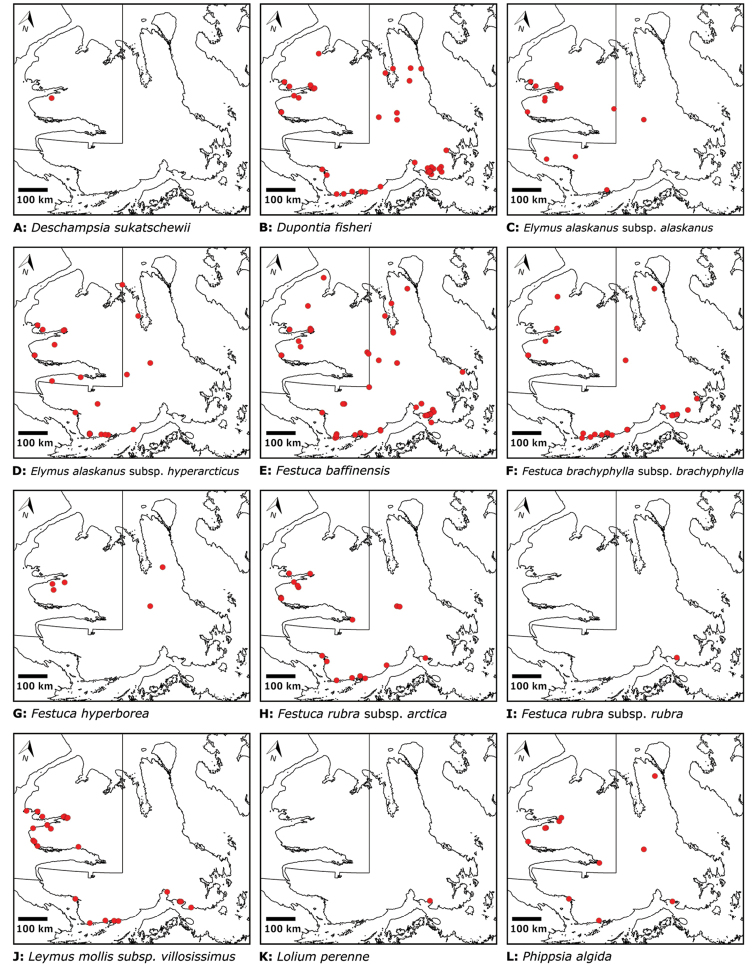
Species distribution maps. Poaceae: **A***Deschampsia sukatschewii***B***Dupontia fisheri***C**Elymus alaskanus subsp. alaskanus**D**Elymus alaskanus subsp. hyperarcticus**E***Festuca baffinensis***F**Festuca brachyphylla subsp. brachyphylla**G***Festuca hyperborea***H**Festuca rubra subsp. arctica**I**Festuca rubra subsp. rubra**J**Leymus mollis subsp. villosissimus**K***Lolium perenne***L***Phippsia algida*.

**Figure 25.**
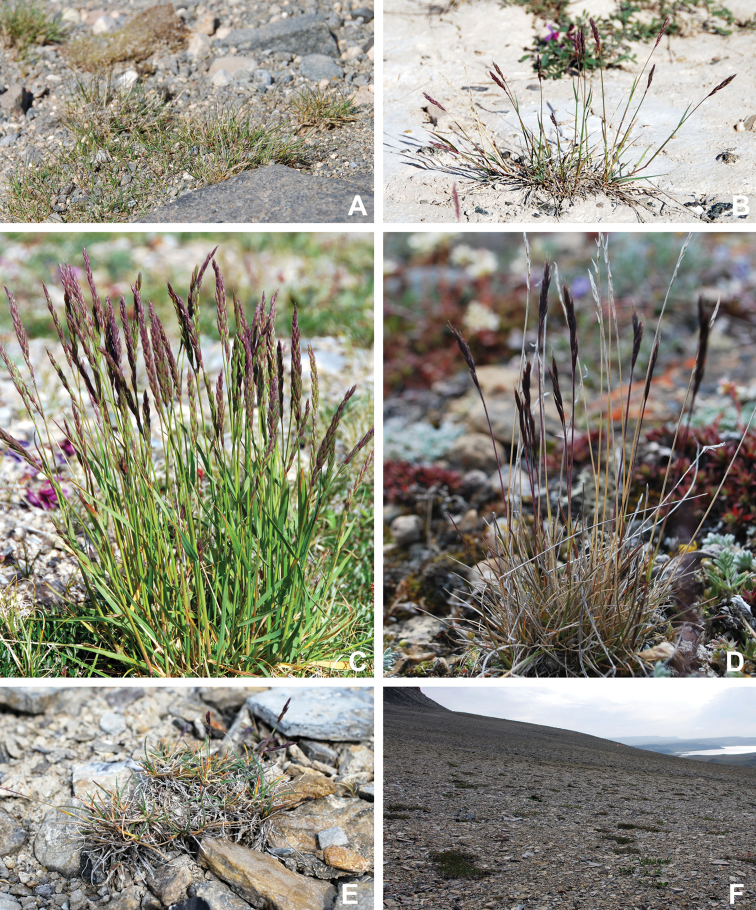
**A***Deschampsia sukatschewii* habitat, *Gillespie et al. 9903***B**Elymus alaskanus subsp. alaskanus habit, *Gillespie et al. 9718***C**Elymus alaskanus subsp. hyperarcticus habit, Minto Inlet, NT, 23 July 2010 **D***Festuca baffinensis* habit **E***Festuca hyperborea* habit, *Gillespie et al. 9863***F***Festuca hyperborea* habitat, *Gillespie et al. 9863*. Photos **A–C**, **E**, **F** by J.M. Saarela and **D** by B.A. Bennett.

***Deschampsia sukatschewii*** (Popl.) Roshev. (*D. pumila* (Griseb.) Ostenf., *nom. illeg*.), Figs 24A, 25A–Dwarf hairgrass | Circumpolar

Newly recorded for Victoria I. where known from a single collection. We collected the taxon along the base of cliffs on the south shore of “Fish L.” on the lower Kuujjua R., where it grew in a hummocky, moderately well-drained meadow. Elsewhere in the Canadian Arctic Archipelago recorded from Banks, Baffin, Devon, Ellesmere, Prince Charles and Prince Patrick islands and a few mainland sites (Porsild and Cody 1980, Aiken et al. 2007, Barkworth 2007a, Saarela et al. 2013, Saarela et al. 2017b). This species was assessed as May Be at Risk by the Working Group on General Status of NWT Species (2011) but the status was revised to Secure by Working Group on General Status of NWT Species (2016) in light of new information on the species in the territory.

**NORTHWEST TERRITORIES. Kuujjua R.**: *Gillespie et al. 9903* (ALA, CAN, O, US)

### *Dupontia* R.Br. [1]

***Dupontia fisheri*** R.Br. (D. fisheri subsp. psilosantha (Rupr.) Hultén), Fig. 24B–Fisher’s tundra grass | Circumpolar

Previously recorded from Cambridge Bay, the head of Prince Albert S., Richard Collinson Inl., Storkerson P. and Ulukhaktok (Porsild 1955, 1957, 1964, Porsild and Cody 1980, Aiken et al. 2007). Reported from all sites studied by Thannheiser et al. (2001) except Mt. Pelly; occurrences at Johansen B. and Hadley B. are confirmed by other collections. Newly recorded from Albert Edward B., Boot Inl., Clouston B., Ferguson L. [Tahiryuaq], Greiner L., Kuujjua R., Mt. Pelly, Murray Pt., Namaycush L., Oterkvik Pt., Read I., Sinclair Cr., Tuktu R., Walker B. and Washburn L. Widespread throughout the Canadian Arctic (Porsild and Cody 1980, Aiken et al. 2007, Saarela et al. 2013, Saarela et al. 2017b).

**NORTHWEST TERRITORIES. Boot Inl.**: *Gillespie et al. 9642* (ALA, CAN, O). **Kuujjua R.**: *Gillespie et al. 9769* (CAN, MT, O), *9939* (ALA, CAN, O, US). **Minto Inl. (head)**: *Edlund 149* (CAN), *Gillespie et al. 10041* (ALA, CAN, O), *10271* (CAN, O), *Porsild 17356*, *17357* (CAN). **Richard Collinson Inl.**: *Edlund 687* (CAN). **Ulukhaktok**: *Edlund 234*, *304*, *512A*, *513*, *514*, *653*, *765*, *898* (CAN), *Porsild 17239* (CAN), *Ross 21* (ALTA), *Saarela & Bull* 1447 (CAN, O). **Walker B.**: *Oldenburg 45-1445*, *45-1446* (CAN). **NUNAVUT. Albert Edward B.**: *Ponomarenko VI-250* (CAN). **Cambridge Bay**: *Bennett 13-0222* (chars, od, UBC), *13-0664* (CAN), *Boulva 65*-*41* (QFA), *Calder et al. 24192* (DAO), *Consaul & Gillespie 1114* (CAN), *Edlund & Argus 12616* (CAN), *Gillespie et al. 8372* (ALA, BABY, CAN, MT, O, UBC, US), *Oldenburg 44*-*896*, *44*-*963* (CAN), *Polunin s.n.* (CAN, 2 sheets), *Porsild 21586* (CAN), *Saarela & Teeter 5282* (CAN), *Stephens 1138*, *1159*, *1200* (CAN). **Clouston B.**: *Gillespie et al. 7749* (ALA, CAN, O, UBC). **Ferguson L. [Tahiryuaq**]: *Hainault 2004* (DAO), *2016* (UAC). **Greiner L.**: *Ponomarenko VI-107*, *VI-140B*, *VI-147*, *VI-185*, *VI-203D*, *VI-293* (CAN). **Hadley B.**: *Edlund 67* (DAO, QFA), *321* (CAN). **Johansen B.**: *Gillespie et al. 8014* (ALA, CAN, MT, O), *8110* (ALA, CAN, MT, O). **Ovayok TP**: *Gould s.n.* (ALA). **Murray Pt.**: *Gillespie et al. 8193* (CAN, O). **Namaycush L.**: *Edlund 132* (CAN). **Oterkvik Pt.**: *Gillespie et al. 7679* (ALA, CAN, MT, O), *7617* (CAN), *7684* (CAN). **Read I.**: *Oldenburg 42-482*, *43-1026*, *43-1027* (CAN). **Sinclair Cr.**: *Gillespie et al. 8235* (ALA, BABY, CAN, MT, O, US). **Storkerson P.**: *Edlund 172* (CAN, US), *Edlund 302*, *348* (CAN). **Tuktu R.**: *Gould s.n.* (ALA). **Washburn L.**: *Oldenburg 46-2193* (CAN).

### *Elymus* L. [1/2]

**Key to *Elymus* [adapted from Barkworth et al. (2007)]**

**Table:** 

1	Glumes and lemmas glabrous, scabrous or sparsely hairy, hairs to about 0.2 mm	**E. alaskanus subsp. alaskanus**
–	Glumes and lemmas densely hairy, hairs 0.2–0.5 mm	**E. alaskanus subsp. hyperarcticus**

***Elymus alaskanus*** (Scribn. *&* Merr.) Á.Löve subsp. ***alaskanus*** (*Agropyron alaskanum* Scribn. & Merrill, *A. violaceum* (Hornem.) Lange pro parte, *A. latiglume* (Scribn. *&* Sm.) Rydb. pro parte, *E. violaceus* (Hornem.) Feilberg pro parte), Figs 24C, 25B–Alaska wildrye | Amphi-Beringian (E)

Previously recorded from the head of Minto Inl., Wollaston P. and Ulukhaktok (Porsild 1955, 1957, 1964) (specimens re-determined here based on current taxonomic concepts), but the species was not mapped for Victoria I. in Porsild and Cody (1980). Aiken et al. (2007) did not distinguish subspecies, and of the additional sites mapped there, specimens from Boot Inl., Cambridge Bay and Namaycush L. are this taxon. Newly recorded from Burns L. and Kuujjua R. Elsewhere in the Canadian Arctic known from mainland sites and Ellesmere I. (Barkworth et al. 2007, Saarela et al. 2013, Saarela et al. 2017b).

**NORTHWEST TERRITORIES. Boot Inl.***Dutilly 18711* (CAN, QFA), *18712*, *18713* (QFA). **Burns L. (S)**: *Edlund 556* (CAN). **Kuujjua R.**: *Edlund 658* (CAN), *Gillespie et al. 9718* (ALA, ari, CAN, O, UBC, US, WIN). **Minto Inl. (head)**: *Gillespie et al. 10009* (ALA, CAN, MT, O), *10151* (ALA, CAN, MT, O), *10143* (ALA, CAN, O), *10280* (CAN, O), *Porsild 17353* (CAN). **Ulukhaktok**: *Porsild 17236* (CAN). **Walker B.**: *Oldenburg 45-1451* (CAN), *Porsild 17794* (CAN). **NUNAVUT. Johansen B.**: *Gillespie et al. 7882* (ALA, BABY, CAN, MT, O, US, V), *7904* (ALA, CAN, O, V). **Namaycush L.**: *Edlund 17* (mixed with E. alaskanus subsp. hyperarcticus), *39*, *147* (CAN). **Wollaston P.**: *D. Jenness 405*, *406* (CAN).

***Elymus alaskanus*** subsp. ***hyperarcticus*** (Polunin) Á.Löve *&* D.Löve (Agropyron violaceum var. hyperarcticum Polunin), Figs 24D, 25C–Tundra wildrye | Asian (N/C)–amphi-Beringian–North American (N)

Previously recorded from Walker B. (Porsild 1955, 1957, 1964) (specimens re-determined here based on modern taxonomic concepts), but the species was not mapped for Victoria I. in Porsild and Cody (1980). Thannheiser et al. (2001) reported it from Johansen B. (conf.). Specimens mapped (to species level) from Mt. Bumpus and Namaycush L. in Aiken et al. (2007) are this taxon. Newly recorded from Boot Inl., Hadley B., Kuujjua R., the head of Minto Inl., Oterkvik Pt., the south side of Prince Albert S. and Sinclair Cr. Elsewhere in the Canadian Arctic widespread across the islands and mainland (Barkworth et al. 2007, Saarela et al. 2013, Saarela et al. 2017b).

**NORTHWEST TERRITORIES. Boot Inl.**: *Gillespie et al. 9538* (ALA, ari, CAN, MT, O, UBC, US). **Kuujjua R.**: *Gillespie et al. 9976* (CAN, O). **Minto Inl. (head)**: *Edlund 112* (CAN), *Gillespie et al. 10224* (ALA, CAN, O). **Prince Albert S. (S)**: *Edlund 537* (CAN). **Ulukhaktok**: *Edlund 305*, *766* (CAN), *Oldenburg 45-1588* (CAN), *Saarela & Bull 1421* (ALA, CAN, O, US). **Walker B.**: *Porsild 17481* (CAN). **Wollaston P.**: *Oldenburg 54*-*506* (UBC). **NUNAVUT. Hadley B.**: *Edlund 19* (CAN). **Johansen B.**: *Gillespie et al. 8081* (CAN, O, V), *8116* (CAN, O, V), *8152* (ALA, CAN, MT, O, V). **Mt. Bumpus**: *Edlund 271* (CAN). **Namaycush L.**: *Edlund 17* (CAN, mixed with E. alaskanus subsp. alaskanus). **Natkusiak P.**: *Edlund 157* (CAN). **Oterkvik Pt.**: *Gillespie et al. 7582*, *7811* (CAN), *7533* (CAN, V), *7558* (CAN, V), *7786* (CAN, O, V). **Prince Albert S. (head)**: *Edlund & Argus 12807* (CAN). **Sinclair Cr.**: *Gillespie et al. 8338* (CAN, O, V).

### *Festuca* L. [4/5]

**Key to *Festuca* [adapted from Aiken et al. (1995) and Darbyshire and Pavlick (2007)]:**

**Table:** 

1	Plants rhizomatous (*F. rubra*)	**2**
–	Plants cespitose, rhizomes lacking	**3**
2	Lemmas usually moderately to densely pilose, rarely glabrous, (4–)4.5–6(–6.5) mm; awns (0.2–)0.5–1.6(–2.5) mm; inflorescences (2–)3.5–7 cm, usually congested, sometimes open, branches scabrous or pilose	**F. rubra subsp. arctica**
–	Lemmas usually glabrous, sometimes pubescent, (4–)6–7.5(–8) mm; awns 0.6–3.2(–4) mm; inflorescences 7–12 cm, open, branches scabrous	**F. rubra subsp. rubra**
3	Culms densely pubescent or pilose below the inflorescences; anthers 0.3–0.7(–1.1) mm; ovary apex with a few sparse hairs	***F. baffinensis***
–	Culms usually glabrous and smooth below the inflorescences, occasionally slightly scabrous or sparsely puberulent; anthers (0.3–)0.4–1.3 mm; ovary apex glabrous	**4**
4	Inflorescences 1.5–4(–5.5) cm; flag leaf sheaths not inflated; flag leaf blades (3–)10–30 mm; leaf blades often curved or somewhat falcate; upper glume 2.9–4.6 mm, lanceolate; lemmas 3–5.2 mm	***F. brachyphylla***
–	Inflorescences 1–2(–2.5) cm; flag leaf sheaths usually somewhat inflated; flag leaf blades 2–5(–8) mm; leaf blades usually straight; upper glume 2.2–3.2 mm, obovate; lemmas 2.9–4 mm	***F. hyperborea***

***Festuca baffinensis*** Polunin, Fig. 24E, 25D–Baffin Island fescue | Asian (NE)–Amphi-Beringian–North American–amphi-Atlantic

Previously recorded from south of Burns L., Cambridge Bay, C. Colborne, the head of Minto Inl., Mt. Bumpus, Mt. Pelly, Namaycush L., Storkerson P. and Ulukhaktok (Porsild 1955, 1957, 1964, Porsild and Cody 1980, Aiken et al. 1995, Aiken et al. 2007). Thannheiser et al. (2001) additionally recorded it from Hadley B. (conf.), Johansen B. (conf.), Richardson I. and Surrey L. Newly recorded from Boot Inl., Greiner L., Murray Pt., “Oldenburg L.”, Oterkvik Pt., an inland site on Prince Albert P., southeast of the head of Prince Albert S., Read I., Sinclair Cr., Storkerson P. and Tuktu R. Elsewhere in the Canadian Arctic recorded from Axel Heiberg, Banks, Baffin, Bathurst, Bylot, Cornwallis, Devon, King William, Melville, Prince Charles, Prince Patrick, Somerset and Southampton islands and numerous mainland sites (Porsild and Cody 1980, Aiken et al. 1995, Aiken et al. 2007, Darbyshire and Pavlick 2007, Saarela et al. 2013, Saarela et al. 2017b).

**NORTHWEST TERRITORIES. Boot Inl.**: *Dutilly 18708* (DAO, QFA, od), *18719* (CAN), *Gillespie et al. 9556* (ALA, ari, CAN, MT, UBC, US). **Burns L. (S)**: *Edlund 48*, *564* (CAN). **Kuujjua R.**: *Gillespie et al. 9725* (CAN), *9963* (ALA, CAN, O). **Minto Inl. (head)**: *Edlund 101*, *114*, *610*, *625* (CAN), *Gillespie et al. 10105* (ALA, CAN, MT, O), *10148* (ALA, ari, CAN, MT, O, UBC), *10153* (ALA, ari, CAN, MT, O, UBC, WIN), *10123* (CAN), *10225* (CAN, O). “**Oldenburg L.**”: *Oldenburg 45-1368* (CAN). **Prince Albert P.**: *Oldenburg 54*-*667* (UBC). **Ulukhaktok**: *Edlund 332*, *484* (CAN), *Gray & Gibbard 46* (DAO), *Saarela & Bull 1464* (CAN, O), *1488* (ALA, CAN, O). **NUNAVUT. Cambridge Bay**: *Argus & Edlund 12638*, *12888* (CAN), *Bennett et al. 13-0316* (ALA, DAO, MO, UBC), *13*-0274 (CAN, chars, od), *Boulva 65*-*45* (QFA), *Calder et al. 24193* (DAO), *Dutilly 28094* (CAN, QFA), *Edlund s.n.* (CAN), *Edlund & Argus 12639*, *12698* (CAN), *Gibson 7072* (ALTA, 2 sheets, CAN, UAC), *Gillespie et al. 8374*, *8469* (ALA, CAN, MT, O, US), *Polunin s.n.* (CAN, 2 sheets), *Ponomarenko VI-070*, *VI-097* (CAN), *Porsild 21587* (CAN), *Scotter s.n.* (ALTA), *Smith & Sweatman 41* (CAN), *Stephens 1025*, *1083* (CAN, KANU). **C. Colborne.**: *Edlund & Argus 12733* (CAN). **Collinson P.**: *Edlund & Argus 12766* (CAN). **Ferguson L. [Tahiryuaq**]: *Bennett et al. 14-0425b* (od), *Hainault 1960*, *2141* (DAO). **Greiner L.**: *Ponomarenko VI-277* (CAN). **Hadley B.**: *Edlund 62*, *138*, *154* (CAN). **Johansen B.**: *Gillespie et al. 7833* (ALA, CAN, MT, O), *7956* (ALA, BABY, CAN, MT, O, US), *8075* (CAN). **Mt. Bumpus**: *Edlund 281*, *422* (CAN). **Ovayok TP**: *Gillespie et al. 8435* (CAN, O), *Stephens 1187* (CAN, KANU). **Murray Pt.**: *Gillespie et al. 8212* (ALA, CAN, O). **Namaycush L.**: *Edlund 15*, *16* (CAN). **Oterkvik Pt.**: *Gillespie et al. 7621* (ALA, CAN, MT, O), *7546*, *7580*, *7642* (CAN). **Prince Albert S. (head)**: *Edlund 88*, *103* (CAN). **Read I.**: *Oldenburg 42-481A*, *43-1017*, *43-1018*, *43-1018A*, *43-902* (CAN). **Sinclair Cr.**: *Gillespie et al. 8223* (ALA, BABY, CAN, MT, O, US), *8243* (ALA, CAN, MT, O, US), *Gillespie et al. 8249* (ALA, CAN, O). **Storkerson P.**: *Edlund 204*, *339* (CAN). **Tuktu R.**: *Gould s.n.* (ALA).

***Festuca brachyphylla*** Schult. & Schult.f. subsp. ***brachyphylla***, Fig. 24F–Alpine fescue | Circumpolar–alpine

Previously recorded from Byron B., Cambridge Bay, the head of Minto Inl., the head of Prince Albert S. (Porsild obs.), Read I. (Porsild obs.), and Ulukhaktok (Porsild 1955, 1957, 1964, Porsild and Cody 1980, Aiken et al. 1995, Aiken et al. 2007). Thannheiser et al. (2001) additionally recorded it from Johansen B. (conf.) and Wellington B. Newly recorded from Albert Edward B., Greiner L., Kuujjua R., Richard Collinson Inl., Ulukhaktok, Ferguson L., Murray Pt., Oterkvik Pt., Sinclair Cr., Storkerson P. and Tuktu R. Elsewhere in the Canadian Arctic recorded from Axel Heiberg, Banks, Baffin, Bathurst, Bylot, Cornwallis, Devon, Ellef Ringness, Fitzwilliam Owen, King William, Melville, Prince Charles, Prince Patrick, Somerset and Southampton islands and numerous mainland sites (Porsild and Cody 1980, Cody et al. 1989, Korol 1992, Aiken et al. 1995, Cody and Reading 2005, Aiken et al. 2007, Saarela et al. 2013, Saarela et al. 2017b).

**NORTHWEST TERRITORIES. Kuujjua R.**: *Gillespie et al. 9750* (ALA, CAN, O). **Minto Inl. (head)**: *Gillespie et al. 10095a* (ari, CAN, O, US). **Richard Collinson Inl.**: *Edlund 168* (CAN). **Ulukhaktok**: *Edlund 855* (CAN), *Oldenburg 45-1593* (CAN), *Saarela & Bull 1468* (CAN, O). **NUNAVUT. Albert Edward B.**: *Ponomarenko VI*-338, *VI*-335B (CAN). **Byron B.**: *Dushenko 13* (UVIC). **Cambridge Bay**: *Bennett et al. 13-0213* (chars), *Edlund & Argus 12644* (CAN), *Gillespie et al. 8456* (ALA, CAN, MT, O), *Polunin s.n.* (CAN), *Ponomarenko VI-077* (CAN). **Ferguson L. [Tahiryuaq**]: *Bennett et al. 14-0425a* (UBC). **Greiner L.**: *Ponomarenko VI-161A* (CAN). **Johansen B.**: *Gillespie et al. 7832* (ALA, CAN, MT, O), *7988* (ALA, BABY, CAN, MT, O, US), *8083* (CAN, O), *8161* (CAN, O). **Murray Pt.**: *Gillespie et al. 8233* (CAN, O). **Oterkvik Pt.**: *Gillespie et al. 7584* (ALA, BABY, CAN, MT, O, US), *7639* (CAN, O), *7681* (ALA, CAN, O). **Sinclair Cr.**: *Gillespie et al. 8228* (ALA, BABY, CAN, MT, O, UBC, US), *8347* (ALA, CAN, MT, O, US). **Storkerson P.**: *Edlund 194* (CAN). **Tuktu R.**: *Gould s.n.* (ALA).

***Festuca hyperborea*** Holmen ex Fred., Figs 24G, 25E, F–High Arctic fescue | Circumpolar

Thannheiser et al. (2001) recorded this taxon from the head of Minto Inl., Richardson I. and Ulukhaktok; the Richardson I. record, if confirmed, would be the southernmost record in the western Canadian Arctic. Aiken et al. (2007) did not record the taxon for Victoria I. Five collections of the taxon are here confirmed for the island, from Kuujjua R., Namaycush L. and Storkerson P. We found the species at two sites in the Kuujjua R. area. One was east of “Fish L.” growing on a gentle slope below a steep scree slope on a large conical hill, on stony, very sparsely vegetated, mostly pale, grey sedimentary rock. The other was the base of north-facing cliffs 68 km ENE of Ulukhaktok, growing on a lower boulder slope at 240 m a.s.l. with *Dryas integrifolia* and *Oxyria digyna*. These collections and the one from the Namaycush L. area close a distribution gap between southern Banks I. and Steffanson I., from which numerous occurrences are known (Aiken et al. 2007). *Edlund 678* was previously misidentified as *F. brachyphylla*, and *Edlund* nos. *36a* and *175* are specimens only recently acquired into the permanent collection at CAN. Elsewhere in the Canadian Arctic recorded from Baffin, Banks, Prince of Wales, Southampton and Steffanson islands, most of the Queen Elizabeth islands, and the Boothia P. (Aiken et al. 1995, Aiken et al. 2007).

**NORTHWEST TERRITORIES. Kuujjua R.**: *Edlund 678* (CAN), *Gillespie et al. 9863* (ari, CAN, MT, O, UBC, US), *9952* (CAN). **NUNAVUT. Namaycush L.**: *Edlund 36a* (CAN). **Storkerson P.**: *Edlund 175* (CAN).

**Figure 26.**
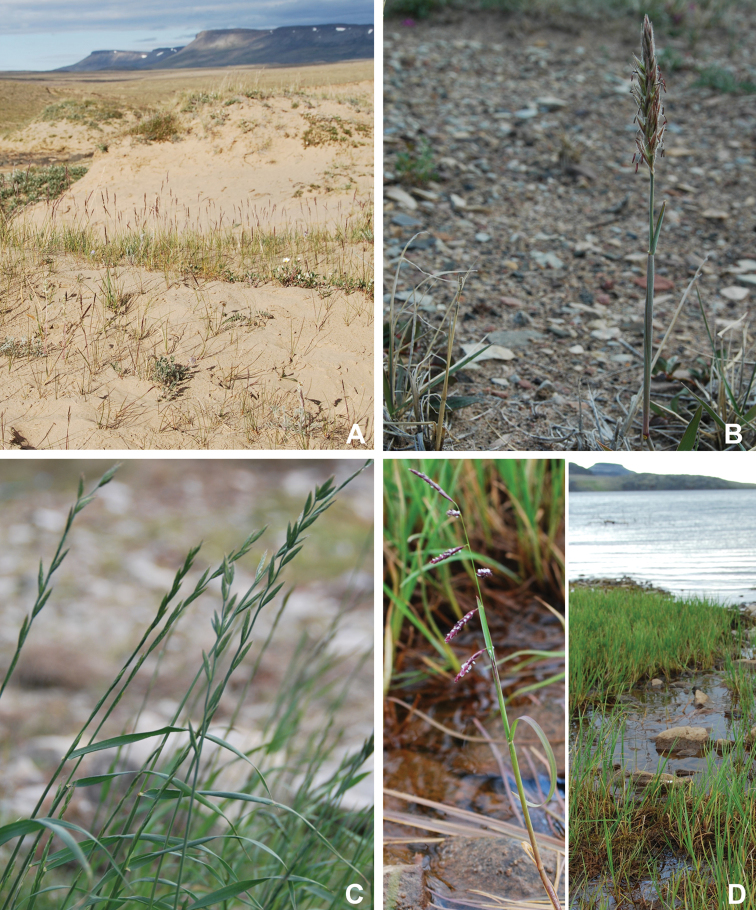
**A**Festuca rubra subsp. arctica habitat, *Gillespie et al. 9913***B**Leymus mollis subsp. villosissimus habit, *Gillespie et al. 8098***C***Lolium perenne* habit, *Saarela & Teeter 9674***D***Pleuropogon sabinei* habit (left) and habitat (right), *Gillespie et al. 9865*. Photos **A**, **C**, **D** by J.M. Saarela and **B** by R.D. Bull.

***Festuca rubra*** subsp. ***arctica*** (Hack.) Govor. (*F. richardsonii* Hook., F. rubra subsp. richardsonii (Hook.) Hultén), Figs 24H, 26A–Richardson’s red fescue | Circumpolar

Previously recorded from the head of Prince Albert S. and Ulukhaktok, as F. rubra var. arenaria (Osbeck) Fr. (a non-Arctic taxon) (Porsild 1955, 1957, 1964), *F. rubra* L. (Porsild and Cody 1980) and F. rubra subsp. richardsonii (Aiken et al. 2007). Thannheiser et al. (2001) additionally recorded it from Cambridge Bay (conf.), the head of Minto Inl. (conf.), Mt. Pelly, Richardson I. and Surrey L. Newly recorded from Boot Inl., Byron B., Kuujjua R., Murray Pt., Namaycush L., Oterkvik Pt. and Read I. The single site known in the Cambridge Bay area is west of the hamlet in the hills above Long Pt., where the species grew on a calcareous kame in *Dryas integrifolia*–*Salix arctica* tundra with Potentilla (sect. Rubricaules), *Poa arctica*, *P. glauca*, *Saxifraga tricuspidata* and *Oxytropis arctica*. Elsewhere in the Canadian Arctic known from Banks and Melville islands as well as mainland sites from Yukon to northern Quebec (Porsild and Cody 1980, Aiken and Darbyshire 1990, Cody and Reading 2005, Aiken et al. 2007, Saarela et al. 2013, Saarela et al. 2017b).

**NORTHWEST TERRITORIES. Boot Inl.**: *Gillespie et al. 9653* (ALA, ari, CAN, MT, O), *9652* (ALA, ari, CAN, O, US). **Kuujjua R.**: *Edlund 656* (CAN), *Gillespie et al. 9938* (ALA, ari, CAN, MT, O), *9913* (ALA, ari, CAN, O, UBC, US). **Minto Inl. (head)**: *Gillespie et al. 10223* (ALA, CAN, O). **Prince Albert S. (head)**: *Porsild 17431* (CAN). **Ulukhaktok**: *Edlund 342*, *479*, *493*, *768*, *871*, *886*, *900*, *901*, *367B*, *789B* (CAN), *Saarela & Bull 1443* (ALA, CAN, O). **NUNAVUT. Byron B.**: *Edlund & Argus 12849* (CAN). **Cambridge Bay**: *Bennett et al. 13-0311* (ALA, CAN, chars, od). **Clouston B.**: *Gillespie et al. 7744* (ALA, CAN, MT, O), *Saarela & Teeter 5297* (CAN). **Johansen B.**: *Gillespie et al. 7837* (ALA, BABY, CAN, MT, O, US), *7885* (ALA, ALTA, BABY, CAN, MT, O, UBC, US), *7884* (CAN), *8117* (CAN, O). **Murray Pt.**: *Gillespie et al. 8197* (ALA, CAN, mixed with Poa hartzii subsp. hartzii, MT, O, US). **Namaycush L.**: *Edlund 36b*, *39*, *140*, *165* (CAN), *Edlund & Argus 12843* (CAN). **Oterkvik Pt.**: *Gillespie et al. 7631* (ALA, CAN, MT, O, US), *7655* (ALA, CAN, MT, O). **Read I.**: *Oldenburg 43-1014*, *43-949* (CAN).

***Festuca rubra*** L. subsp. ***rubra***, Fig. 24I–Red fescue | Circumboreal-polar

Newly recorded for Victoria I. from Cambridge Bay, where found in 2017 growing in highly disturbed ground in the community with *Descurainia sophioides*, Poa pratensis subsp. pratensis and *Lolium perenne*; the three grasses were almost certainly seeded. Persistence of the species at the site beyond 2017 requires confirmation. Elsewhere in the Canadian Arctic known from a few other sites, including Iqaluit and Clyde R. on Baffin I., Eglinton I. (needs confirmation) and scattered mainland sites (Porsild and Cody 1980, Aiken and Darbyshire 1990, Gould and Walker 1997, Saarela et al. 2017b).

**NUNAVUT. Cambridge Bay**: *Saarela & Teeter 5297* (CAN).

### *Leymus* Hochst. [1]

***Leymus mollis*** subsp. ***villosissimus*** (Scribn.) Á.Löve *&* D.Löve (Elymus arenarius subsp. villosissimus (Scribn.) Á.Löve), Figs 24J, 26B–Arctic lymegrass | Asian (NE)–amphi-Beringian–North American (N)

Previously recorded from Cambridge Bay (Porsild obs., conf.), the head of Minto Inl., the north side of Prince Albert S., Read I. (Porsild obs., conf.) and Ulukhaktok (Porsild 1955, 1957, 1964, Porsild and Cody 1980, Aiken et al. 2007). Thannheiser et al. (2001) additionally recorded it from Johansen B. (conf.). Newly recorded from Anderson B., Boot Inl., C. Wollaston, Ferguson L., Freshwater Bay, Kuujjua R., Murray Pt., Oterkvik Pt. and Walker B. The inland site in the vicinity of the Kuujjua R. is a large sand dune area where the species was common, growing with *Salix alaxensis*, *Symphyotrichum pygmaeum*, *Arctous rubra*, *Armeria scabra* and *Calamagrostis purpurascens*. Elsewhere in the Canadian Arctic recorded from Banks, Baffin, King William and Southampton islands and across the mainland (Aiken et al. 2007, Saarela et al. 2013, Saarela et al. 2017b).

**NORTHWEST TERRITORIES. Berkeley Pt.**: *Stretton 87* (DAO). **Boot Inl.**: *Gillespie et al. 9643* (ALA, CAN, MT, O). **C. Wollaston**: *Edlund 15* (CAN). **Freshwater Bay**: *Stretton 76* (DAO). **Kuujjua R.**: *Dutilly 18832* (CAN, QFA, MT), *Gillespie et al. 9937* (ALA, ari, CAN, MT, UBC, US, WIN), *9912* (ALA, CAN, O, US). **Minto Inl. (head)**: *Edlund 173* (CAN), *Gillespie et al. 10232* (ALA, CAN, MT, O), *10267* (ALA, CAN, O), *Porsild 17358* (CAN). **Prince Albert S. (N)**: *Stretton 44* (DAO). **Ulukhaktok**: *Edlund 303*, *784* (CAN), *Pokiak 35* (CAN), *Saarela & Bull 1466* (ALA, ari, CAN, MT, O). **Walker B.**: *Oldenburg 45-1453* (CAN). **NUNAVUT. Anderson B.**: *Edlund & Argus 12701* (CAN). **Cambridge Bay**: *Bennett et al. 13-0564* (BABY, UBC), *13-0231* (ALA, chars, UBC), *Gillespie et al. 8461* (ALA, CAN, O), *Stephens 1255* (CAN). **Ferguson L. [Tahiryuaq**]: *Hainault 1986* (DAO). **Johansen B.**: *Gillespie et al. 8013* (ALA, CAN, MT, O, US), *8098* (ALA, CAN, MT, O, US). **Murray Pt.**: *Gillespie et al. 8189* (ALA, BABY, CAN, MT, O, UBC, US). **Oterkvik Pt.**: *Gillespie et al. 7611* (ALA, CAN, MT, O, US). **Read I.**: *Oldenburg 43-1033*, *43-884*, *43-894*, *43-921*, *43-952* (CAN).

### *Lolium* L. [1]

***Lolium perenne*** L., Figs 24K, 26C–Perennial ryegrass

Newly recorded from Cambridge Bay, where found in 2017 growing in highly disturbed ground in the community with *Descurainia sophioides*, Festuca rubra subsp. rubra and Poa pratensis subsp. pratensis; the three grasses were almost certainly seeded. Persistence of the species at the site beyond 2017 requires confirmation. Elsewhere in the Canadian Arctic Archipelago known only from Iqaluit, where it was seeded (Aiken et al. 2007).

**NORTHWEST TERRITORIES. Cambridge Bay**: *Saarela & Teeter 5294* (CAN)

### *Phippsia* (Trin.) R.Br. [1]

***Phippsia algida*** (Sol.) R.Br., Fig. 24L–Icegrass | Circumpolar

Previously recorded from the head of Minto Inl., Namaycush L., the head of Prince Albert S., Storkerson P. and Ulukhaktok. Thannheiser et al. (2001) additionally recorded it from Johansen B. (conf.) and Surrey L. Newly recorded from Cambridge Bay (Long Point area), Kuujjua R. and Read I. The single site known in the Cambridge Bay area is a sandy drainage channel above Long Point, west of the hamlet, where the species grew in wet sand along a stream with no immediately associated vegetation. Widespread throughout the Canadian Arctic Archipelago and scattered adjacent mainland sites area (Porsild and Cody 1980, Cody et al. 1989, Consaul and Aiken 2007, Saarela et al. 2017b).

**NORTHWEST TERRITORIES. Kuujjua R.**: *Gillespie et al. 9895* (ALA, CAN, O, US), *10001* (CAN). **Minto Inl. (head)**: *Gillespie et al. 10285* (CAN), *Porsild 17359* (CAN). **Prince Albert S.**: *Porsild 17433* (CAN). **Ulukhaktok**: *Edlund 801* (CAN). **NUNAVUT. Cambridge Bay**: *Bennett 13-0337* (CAN). **Johansen B.**: *Gillespie et al. 8126* (CAN, O). **Namaycush L.**: *Edlund & Roncato-Spencer 108* (CAN). **Read I.**: *Oldenburg 43-890* (CAN). **Storkerson P.**: *Edlund 242* (CAN).

### *Pleuropogon* R.Br. [1]

**Figure 27.**
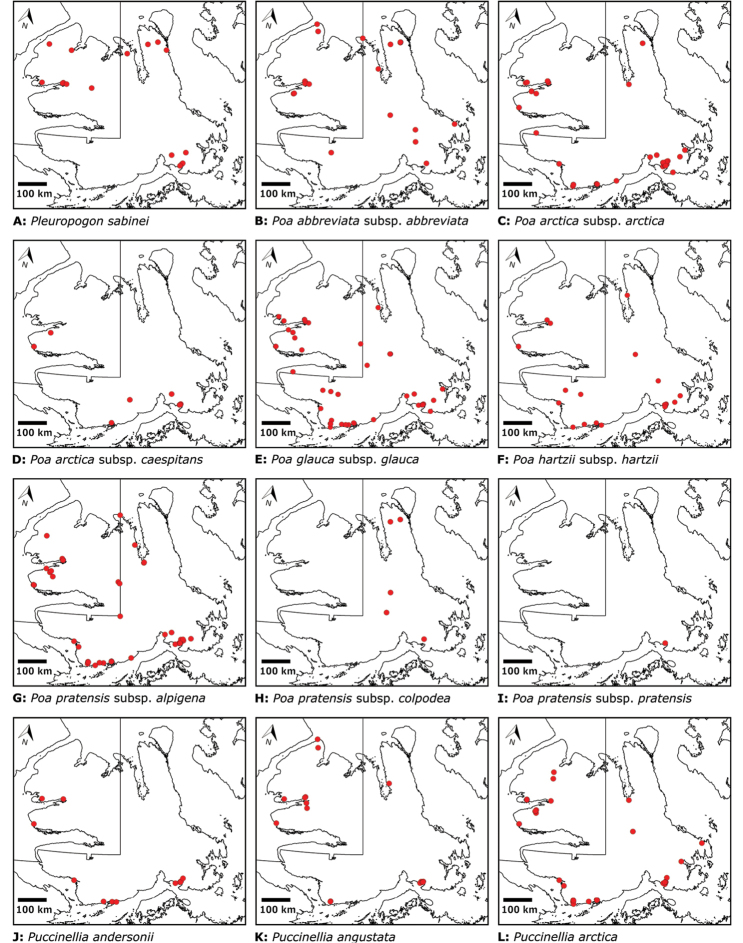
Species distribution maps. Poaceae: **A***Pleuropogon sabinei***B**Poa abbreviata subsp. abbreviata**C**Poa arctica subsp. arctica**D**Poa arctica subsp. caespitans**E**Poa glauca subsp. glauca**F**Poa hartzii subsp. hartzii**G**Poa pratensis subsp. alpigena**H**Poa pratensis subsp. colpodea**I**Poa pratensis subsp. pratensis**J***Puccinellia andersonii***K***Puccinellia angustata***L***Puccinellia arctica*.

***Pleuropogon sabinei*** R.Br., Figs 27A, 26D–Sabine’s semaphore grass | Circumpolar

Previously recorded from Cambridge Bay, Ferguson L. near the mouth of Ekalluk R., the head of Minto Inl. and a site (“Jackpot L.”) 60 miles east thereof, Richard Collinson Inl. and Storkerson P. (Porsild 1955, 1957, 1964, Porsild and Cody 1980, Aiken et al. 2007). Thannheiser et al. (2001) additionally recorded it from Hadley B. and Surrey L. Newly recorded from Boot Inl., Natkusiak P. and SE of Armstrong Pt. Elsewhere in the Canadian Arctic recorded from throughout the Canadian Arctic Archipelago and scattered mainland sites along western Hudson Bay and eastwards (Porsild and Cody 1980, Cody et al. 1989, Korol 1992).

**NORTHWEST TERRITORIES. SE of Armstrong Pt.**: *Edlund 587* (CAN). **Boot Inl.**: *Gillespie et al. 9674* (CAN, UTC). **Minto Inl. (head)**: *Edlund 115* (CAN), *Gillespie et al. 10231* (ALA, CAN, O, UTC), *Porsild 17360*, *17501* (CAN). **Richard Collinson Inl.**: *Edlund 693* (CAN). **NUNAVUT. Cambridge Bay**: *Bennett et al. 13-0179* (BABY, chars), *Porsild 21589* (CAN), *Stephens 1062* (CAN), *1103* (KSTC), *1169* (CAN). **Ferguson L. [Tahiryuaq**]: *Edlund & Argus 12772* (CAN), *Hainault 1994* (DAO). **Natkusiak P.**: *Gould s.n.* (ALA). **Storkerson P.**: *Edlund 190*, *242*, *290* (CAN).

### *Poa* L. [5/8]

**Key to *Poa* [adapted from Soreng (2007)]**

**Table:** 

1	Plants rhizomatous	**2**
–	Plants cespitose, lacking rhizomes	**5**
2	Lemmas 3–6 mm, short-villous to softly puberulent between veins proximally; paleas softly puberulent between keels; spikelets (3.5–)4.5–8 mm; panicles ovoid to broadly pyramidal, usually open	**P. arctica subsp. arctica**
–	Lemmas 2.5–4.3 mm, glabrous between veins; paleas glabrous between keels; spikelets 4–5.5 mm [3.5–6(–7) mm in subsp. pratensis]; panicles narrowly pyramidal or contracted [broadly pyramidal, open or somewhat contracted in subsp. pratensis] (*Poa pratensis*)	**3**
3	At least some spikelets bulbiferous	**P. pratensis subsp. colpodea**
–	Spikelets not bulbiferous	**4**
4	Panicle branches smooth or almost smooth; panicles with (1–)2–5(–7) branches per node, branches steeply ascending to eventually spreading or somewhat reflexed; plants usually loosely, sometimes moderately, tufted, culms usually solitary	**P. pratensis subsp. alpigena**
–	Panicles branches more or less scabrous; panicles with 3–5(–7) branches per node, branches spreading to somewhat reflexed; plants densely to loosely tufted, often forming turf, culms clustered	**P. pratensis subsp. pratensis**
5	Anthers all well developed, 0.1–1(–1.2) mm	**P. abbreviata subsp. abbreviata**
–	Anthers well developed or aborted, (1–)1.2–2.5 mm, sometimes aborted anthers shorter	**6**
6	Sheaths closed for (1/5)1/3 their length; panicles lax to erect, pyramidal, open; branches ascending or widely spreading, sinuous and flexuous to fairly straight	***P. arctica*** subsp. ***caespitans***
–	Sheaths closed for 1/10–1/5 their length; panicles erect, narrowly lanceoloid to ovoid, contracted to somewhat open; branches erect, ascending or weakly spreading, straight or fairly straight	**7**
7	Plants usually glaucous; glume keels distinct; lemmas 2.5–4 mm, distinctly keeled, keels and marginal veins short-villous, lateral veins obscure, usually sparsely softly puberulent to short-villous, intercostal regions glabrous or puberulent, margins not scarious; calluses glabrous or webbed, webs from minute to more than 1/2 the lemma length; culm bases straight or slightly decumbent; anthers fertile, rarely aborted late in development	**P. glauca subsp. glauca**
–	Plants not glaucous; glume keels indistinct; lemmas (3.3–)3.9–5.4 mm, usually weakly keeled, more or less evenly and somewhat loosely to densely hairy over the proximal 1/3–1/2, hairs usually longer than 0.5 mm, smooth distally, margins broadly scarious; calluses usually with a crown of hairs, hairs to 2 mm; culm bases decumbent; anthers usually aborted late in development and sterile	**P. hartzii subsp. hartzii**

**Figure 28.**
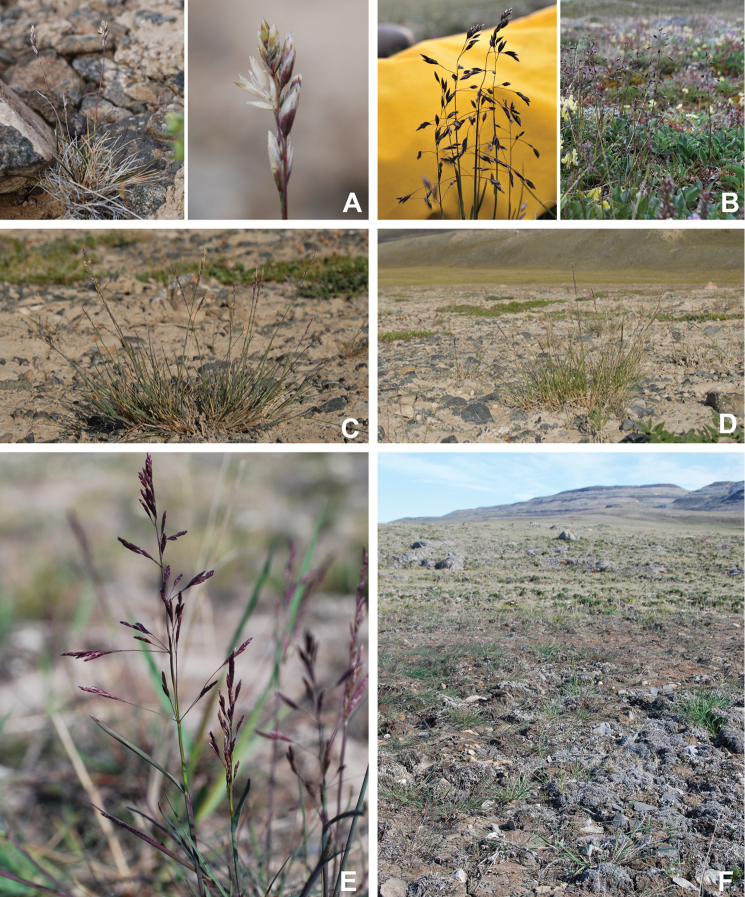
**A**Poa abbreviata subsp. abbreviata habit (left) and inflorescence (right), *Gillespie et al. 9865***B**Poa arctica subsp. arctica inflorescence (left) and habit (right) **C**Poa glauca subsp. glauca habit, *Gillespie et al. 10079***D**Poa hartzii subsp. hartzii habit, *Gillespie et al. 10078***E***Puccinellia arctica* inflorescence, *Gillespie et al. 9660***F***Puccinellia arctica* habitat, *Gillespie et al. 9660*. Photos **A**, **E**, **F** by J.M. Saarela **B** by B.A. Bennett and **C**, **D** by L.J. Gillespie.

***Poa abbreviata* R.Br.** subsp. ***abbreviata***, Figs 27B, 28A–Abbreviated bluegrass | Nearly circumpolar

Previously recorded from Collinson P., the head of Minto Inl., Namaycush L., Natkusiak P., Peel Pt., Storkerson P., Ulukhaktok, an unnamed lake ca. 60 mi. N of Cambridge Bay and Wollaston P. (Macoun and Holm 1921, Porsild 1955, 1957, 1964, Porsild and Cody 1980, Aiken et al. 2007). We were not able to locate the voucher from Wollaston P. collected by Johansen and Jenness (Macoun and Holm 1921). Thannheiser et al. (2001) additionally recorded it from Hadley B. (conf.) and Surrey L. Newly recorded from Colville Mts., Kuujjua R., “Oldenburg L.”, Mt. Pelly and “Trunsky L.” Elsewhere in the Canadian Arctic widespread across the Arctic islands and recorded on the adjacent Melville P. but conspicuously absent from the southern half of Baffin I. (Aiken *et al.* 2007).

**NORTHWEST TERRITORIES. Kuujjua R.**: *Gillespie et al. 9980* (ALA, ALTA, ari, CAN, MO, O, UBC, US, WIN), *9865* (ALA, CAN, O), *9863* (CAN). **Minto Inl. (head)**: *Edlund 134* (CAN), *Gillespie et al. 10094* (ALA, ari, CAN, MT, O, UBC), *10077* (CAN, O), *10202* (CAN), *Porsild 17361* (CAN). “**Oldenburg L.**”: *Oldenburg 45-1363*, *45-1364* (CAN). **Peel Pt.**: *Edlund 418* (CAN). **NUNAVUT. Collinson P.**: *Edlund & Argus 12760* (CAN). **Colville Mts.**: *Gillespie et al. 7780* (ALA, BABY, CAN, MT, O, UBC, US). **Hadley B.**: *Edlund 64* (CAN). **Ovayok TP**: *Bennett et al. 13-0620* (BABY). **Namaycush L.**: *Edlund & Roncato-Spencer 57* (CAN). **Natkusiak P.**: *Edlund 167* (CAN). **Unnamed lake ca. 60 mi. N of Cambridge Bay**: *Porsild 17472* (CAN). **Storkerson P.**: *Edlund 199*, *205*, *228*, *246* (CAN). “**Trunsky L.**”: *Bennett et al. 14-0397* (BABY).

***Poa arctica*** R.Br. subsp. ***arctica***, Figs 27C, 28B–Arctic bluegrass | Circumpolar–alpine

Previously recorded from Cambridge Bay, the head of Prince Albert S. (Porsild obs.), Kuujjua R., Storkerson P., Ulukhaktok and northwestern Wollaston P. (Porsild 1955, 1957, 1964, Porsild and Cody 1980, Aiken et al. 2007). A collection mapped from Natkusiak P. (*Edlund 98*) in Aiken et al. (2007) has been redetermined as P. pratensis subsp. alpigena. Thannheiser et al. (2001) additionally recorded it from the head of Minto Inl. (conf.), Richardson I., Johansen B. (conf.), Hadley B. (conf.), Surrey L. and Wellington B. Newly recorded from Albert Edward B., Anderson B., Boot Inl., Ferguson L., Greiner L., Oterkvik Pt., Read I. and Sinclair Cr. Widespread throughout the Canadian Arctic (Porsild and Cody 1980, Cody et al. 1984a, Cody et al. 1989, Korol 1992, Aiken et al. 2007, Saarela et al. 2013, Saarela et al. 2017b).

**NORTHWEST TERRITORIES. Boot Inl.**: *Edlund 580* (CAN), *Gillespie et al. 9543b* (ALA, CAN, MT, O, US). **Kuujjua R.**: *Dutilly 18808* (DAO, QFA, 2 sheets), *Gillespie et al. 9786* (CAN, US), *9924* (CAN, O). **Minto Inl. (head)**: *Edlund 109*, *624* (CAN), *Gillespie et al. 10042* (ALA, CAN, MT, O), *10054b* (CAN, O, US). **Ulukhaktok**: *Edlund 485*, *731*, *857*, *905*, *906* (CAN), *Porsild 17241* (ALTA, CAN). **Wollaston P.**: *Oldenburg 54*-*501A* (MIN). **NUNAVUT. Albert Edward B.**: *Ponomarenko VI-337* (CAN). **Anderson B.**: *Edlund & Argus 12707* (CAN). **Cambridge Bay**: *Bennett et al. 13-0255* (BABY, US), *14-0314* (UBC), *Gillespie 5827*, 5829, *5830*, *5842* (CAN), *Gillespie et al. 8359* (ALA, ALTA, BABY, CAN, MT, O, UBC, US), *Oldenburg 44*-*883* (CAN), *Polunin s.n.* (CAN, 2 sheets), *Stephens 1152A* (CAN). **Ferguson L. [Tahiryuaq**]: *Hainault 2003* (DAO, UAC, mixed with Poa pratensis subsp. alpigena), *Jones & Hainault 2054* (DAO). **Greiner L.**: *Ponomarenko VI-116*, *VI-243*, *VI-273*, *VI-279A*, *VI-320* (CAN). **Hadley B.**: *Edlund 139* (CAN). **Johansen B.**: *Gillespie et al. 7972* (ALA, ALTA, BABY, CAN, MT, O, UBC, US), *7842* (ALA, CAN, O), *7954-1* (CAN, US, O), *7954-2* (CAN), *7578* (ALA, CAN, O). **Oterkvik Pt.**: *Gillespie et al. 7596* (CAN, O), *7802* (CAN). **Read I.**: *Oldenburg 43-1015*, *43-1016*, *43-1156*, *43-896*, *43-942* (CAN). **Sinclair Cr.**: *Gillespie et al. 8248* (ALA, CAN, MT, O). **Storkerson P.**: *Edlund 229* (CAN).

***Poa arctica*** subsp. ***caespitans*** Simmons ex Nannf., Fig. 27D–High arctic bluegrass | North American (NE)–amphi-Atlantic–European (N)

Previously recorded from Cambridge Bay and Ulukhaktok (Porsild and Cody 1980, Aiken et al. 2007). Thannheiser et al. (2001) additionally recorded it from Hadley B., Mt. Pelly, Richardson I., Surrey L. and Wellington B. Newly recorded from Kuujjua R., Ferguson L., Johansen B. and south-central Victoria I. Elsewhere in the Canadian Arctic recorded from Axel Heiberg, Baffin, Devon, Ellef Ringnes, King William, Prince Patrick and Southampton islands and scattered mainland sites (Porsild and Cody 1980, Aiken et al. 2007).

**NORTHWEST TERRITORIES. Kuujjua R.**: *Gillespie et al. 9906* (CAN). **Ulukhaktok**: *Edlund 812* (CAN), *Saarela & Bull 1430* (ALA, ari, CAN, MT, O). **NUNAVUT. Cambridge Bay**: *Calder et al. 24153*, *24202* (DAO), *Gillespie 5843* (CAN), *Gillespie et al. 8497* (ALA, BABY, CAN, MT, O, UBC, US), *Gillespie & Consaul 6316* (CAN, US), *Stephens 1094* (CAN, KANU), *1152* (CAN, KANU, KSTC), *Washburn 31* (CAN). **Ferguson L. [Tahiryuaq**]: *Hainault 2073* (DAO). **Johansen B.**: *Gillespie et al. 7922* (CAN, O). **South-central Victoria I.**: *Edlund & Argus 12874* (CAN).

***Poa glauca*** Vahl subsp. ***glauca***, Figs 27E, 28C–Glaucus bluegrass | Circumpolar–alpine

Previously recorded from Anderson B., south of Burns L., Cambridge Bay, Ferguson L., the head of Minto Inl., Mt. Bumpus, Namaycush L., the north side of Prince Albert S., Ulukhaktok and Wollaston P. (Macoun and Holm 1921, Porsild 1955, 1957, 1964, Porsild and Cody 1980, Aiken et al. 2007). Thannheiser et al. (2001) additionally recorded it from Johansen B. (conf.), Mt. Pelly (conf.), Surrey L. and Wellington B. Newly recorded from Boot Inl., Colville Mts., Clouston B., Greiner L., Hadley B., Oterkvik Pt., Sinclair Cr. and Walker B. Widespread across the Canadian Arctic (Porsild and Cody 1980, Cody et al. 1989, Korol 1992, Cody and Reading 2005, Aiken et al. 2007, Saarela et al. 2013, Saarela et al. 2017b).

**NORTHWEST TERRITORIES. Boot Inl.**: *Dutilly 18707* (DAO, QFA), *Gillespie et al. 9560* (ALA, ari, CAN, MT, O, UBC, US, WIN). **Burns L. (S)**: *Edlund 567* (CAN). **Kuujjua R.**: *Dutilly 18809* (DAO, QFA), *18811* (DAO, MT), *18812* (QFA), *Gillespie et al. 9724* (ALA, ari, CAN, MT, O, UBC, WIN), *9751* (CAN), *Gillespie et al. 9964* (ALA, ari, CAN, MT, O, UBC). **Minto Inl. (head)**: *Edlund 100*, *116*, *627* (CAN), *Gillespie et al. 10011* (ALA, CAN, MT, O, US), *9500*, *10023* (CAN), *Porsild 17362* (ALA, CAN). **Prince Albert S. (N)**: *Edlund 442*, *443* (CAN), *Edlund & Argus 12808* (CAN). **Ulukhaktok**: *Edlund 882*, *902*, *906* (CAN), *Gray & Gibbard 51* (DAO), *Saarela & Bull 1459* (ALA, ari, CAN, MT, O, UBC), *1428* (ALA, CAN, O). **Walker B.**: *Oldenburg 45-1456* (CAN). **Wollaston P.**: *Oldenburg 54*-*203*, *54*-*503* (MIN). **NUNAVUT. Anderson B.**: *Edlund & Argus 12706* (ALA, CAN). **Cambridge Bay**: *Bennett 14-0305* (BABY, US), *13-0237* (BABY), *Dutilly 28091* (DAO, QFA), *28093* (QFA, 2 sheets), *Edlund & Argus 12617* (ALA, CAN), *12635* (ALA, CAN), *Gillespie 5821*, *5822*, *5823*, *5831*, *5834*, *5841* (CAN), *Oldenburg 44-887* (CAN), *Polunin s.n.* (CAN), *Porsild 21591*, *21592* (CAN), *Smith & Sweatman 35*, *37* (CAN, MO). **Clouston B.**: *Gillespie et al. 7735a* (ALTA, CAN, O). **Colville Mts.**: *Gillespie et al. 7781-1* (ALTA, CAN), *7781-2* (CAN, US). **Ferguson L. [Tahiryuaq**]: *Hainault 2041*, *2081*, *2128* (DAO, UAC), *2018* (UAC). **Greiner L.**: *Ponomarenko VI-159* (CAN). **Hadley B.**: *Edlund 25* (CAN). **Johansen B.**: *Gillespie et al. 8149* (ALTA), *7841* (ALA, ALTA, CAN, MT, O, UBC, US), *7915* (ALA, BABY, CAN, MT, O, UBC, US), *8082* (ALA, CAN, MT, O, US), *8149-2* (CAN, US, UBC, MT), *7990* (ALA, CAN, O), *7841-2* (CAN, ALTA, US), *8105*-*1* (CAN, ALTA, UBC, US), *8105-2* (CAN), *8149-1* (CAN, US, O, ALA). **Mt. Bumpus**: *Edlund 166*, *239*, *270* (CAN). **Mount Pelly**: *Ponomarenko VI-337A* (CAN). **Namaycush L.**: *Edlund 28* (CAN), *Edlund & Roncato-Spencer 67*, *68*, *72*, *73* (CAN). **Oterkvik Pt.**: *Gillespie et al. 7595* (ALA, BABY, CAN, MT, O, UBC, US), *7662* (ALA, ALTA, BABY, CAN, MT, O, UBC, US), *7632* (CAN, O, US), *7532-1* (CAN), *7532-2* (CAN). **Sinclair Cr.**: *Gillespie et al. 8351* (ALA, BABY, CAN, MT, O, UBC, US). **Wollaston P.**: *Johansen & D. Jenness 402* (CAN).

***Poa hartzii*** Gand. subsp. ***hartzii***, Figs 27F, 28D–Hartz’s bluegrass | Asian Beringian?–North American (N)–amphi-Atlantic (W)

Previously recorded from Cambridge Bay, Hadley B., Namaycush L., Ulukhaktok and Wollaston P. (Porsild 1955, 1957, 1964, Aiken et al. 2007). Thannheiser et al. (2001) additionally recorded it from Richardson I. Newly recorded from Greiner L., Johansen B., the head of Minto Inl., Mt. Bumpus, Murray Pt., Oterkvik Pt., Read I. and “Trunsky L.” Pseudoviviparous plants are here included in subsp. hartzii instead of treated as subsp. vrangelica (type from Wrangel Island, Russia), as recently recognized (Soreng 2007, Elven et al. 2011), because pseudoviviparous plants in the Canadian Arctic usually occur in the same population as non-pseudoviviparous ones. The reverse is not true, however, as many populations are entirely non-pseudoviviparous. Pseudovivipary in *Poa hartzii* is apparently uncommon on Victoria I., known from only two collections. Elsewhere in the Canadian Arctic recorded from Axel Heiberg, Baffin (Clyde Inl. area), Banks, Cornwallis, Devon and Melville islands, and scattered sites along the mainland Northwest Territories coast and northern Quebec (Cayouette 1984, Soreng 1991, Gillespie et al. 1997, Aiken et al. 2007, Soreng 2007, Soreng and Gillespie 2007, Saarela et al. 2013).

**NORTHWEST TERRITORIES. Minto Inl. (head)**: *Gillespie et al. 10078* (CAN, O), *10245* (CAN), *10299* (CAN, O, US). **Ulukhaktok**: *Edlund 904* (CAN), *Saarela & Bull 1416* (ALA, CAN, MT, O). **NUNAVUT. Cambridge Bay**: *Edlund & Argus 12696* (CAN), *Gillespie 5824*, *5832*, *5832*, *5833*, *5849* (CAN), *Gillespie & Consaul 6323*, *6333*, *6338*, *6351*, *6319* (CAN), *Gillespie et al. 8392* (ALA, BABY, CAN, MT, O, UBC, US), *8495* (ALA, CAN, MT, O, US), *Polunin s.n.* (CAN, 2 sheets), *Stephens 1084* (CAN, KSTC). **Greiner L.**: *Ponomarenko VI-245*, *VI-197* (CAN). **Hadley B.**: *Edlund 338*, *328* [pseudoviviparous form] (CAN). **Johansen B.**: *Gillespie et al. 8148-6* (MT), *7841-1* (CAN, US, O, ALA, MT, BC), *8148-1* (CAN, UBC), *8148*-*2* (ALTA, BABY, CAN). **Mt. Bumpus**: *Edlund 169* (CAN). **Murray Pt.**: *Gillespie et al. 8197* (CAN, mixed with Festuca rubra subsp. arctica). **Namaycush L.**: *Edlund & Argus 12844* (CAN). **Oterkvik Pt.**: *Gillespie et al. 7616a* (CAN). **Read I.**: *Oldenburg 43-1013*, *43-897*, *43-949b* (CAN). “**Trunsky L.**”: *Bennett et al. 14-0681* (US). **Wollaston P.**: *D. Jenness 402*, *407* [pseudoviviparous form] (CAN).

***Poa pratensis*** subsp. ***alpigena*** (Lindm.) Hiitonen (*P. alpigena* Lindm.), Fig. 27G–Alpine meadow bluegrass | Circumboreal-polar

Previously recorded from south of Burns L., Cambridge Bay, the head of Minto Inl., southeast of the head of Prince Albert S. and Ulukhaktok (Porsild 1955, 1957, 1964, Porsild and Cody 1980, Aiken et al. 2007). Thannheiser et al. (2001) additionally recorded it from Richardson I., Surrey L. and Wellington B. Newly recorded from Clouston B., Ferguson L., Hadley B., Johansen B., Greiner L., Mt. Pelly, Natkusiak P., Oterkvik Pt., Read I. and Sinclair Cr. Widespread throughout the Canadian Arctic Archipelago and known from scattered collections from the mainland Arctic (Porsild and Cody 1980, Cody et al. 1984a, Cody et al. 1989, Korol 1992, Gould and Walker 1997, Cody and Reading 2005, Soreng 2007, Saarela et al. 2013, Saarela et al. 2017b).

**NORTHWEST TERRITORIES. Burns L. (S)**: *Edlund 566*, *62a* (CAN). **Kuujjua R.**: *Dutilly 18806* (QFA), *Gillespie et al. 9858* (CAN, O), *9915* (ALA, CAN, O), *9965* (ALA, CAN, O, US). **Minto Inl. (head)**: *Edlund 98* (CAN), *Gillespie et al. 10152* (ALA, ari, CAN, MT, O, UBC), *10147* (ari, CAN, MT, O, UBC, US), *10121* (CAN, O), *10093b* (CAN, O, US). **Prince Albert P.**: *Oldenburg 54*-*243A* (MIN). **Ulukhaktok**: *Edlund 88*, *789*, *810*, *872* (CAN), *Oldenburg 45-1596* (CAN), *Saarela & Bull 1460* (ALA, ari, CAN, MT, O). **NUNAVUT. Cambridge Bay**: *Bennett 13-0219* (US), *13-0164* (BABY, CAN, chars), *Calder et al. 24194* (DAO), *Dutilly 37129* (QFA), *Edlund & Argus 12640*, *12690*, *12695* (CAN), *Oldenburg 44*-*935* (CAN), *Polunin s.n.* (CAN, 2 sheets), *Smith & Sweatman 35* (DAO, QFA), *38* (DAO), *Stephens 1101* (CAN). **Clouston B.**: *Gillespie et al. 7725* (ALA, CAN, MT, O). **Ferguson L. [Tahiryuaq**]: *Hainault 2003* (DAO, UAC, mixed with Poa arctica subsp. arctica), *2052* (DAO). **Greiner L.**: *Ponomarenko VI-045*, *VI-071*, *VI-161*, *VI-331* (CAN). **Hadley B.**: *Edlund 52*, *111*, *153* (CAN). **Johansen B.**: *Gillespie et al. 7883*, *8067*, *8150* (ALA, CAN, MT, O), *7955-2* (ALA, CAN, MT, O), *8090* (ALA, ALTA, BABY, CAN, US). **Ovayok TP**: *Gould s.n.* (ALA). **Natkusiak P.**: *Edlund 98* (CAN). **Oterkvik Pt.**: *Gillespie et al. 7789* (ALA, CAN, MT, O, UBC, US), *7564* (CAN, O, US), *7575* (CAN, O, US), *7583* (CAN, O), *7673* (CAN), *7798* (CAN, O, US). **Prince Albert S. (head)**: *Edlund 104* (CAN). **Read I.**: *Oldenburg 42-485*, *43-988* (CAN). **Sinclair Cr.**: *Gillespie et al. 8346* (ALA, CAN, MT, O).

***Poa pratensis*** subsp. ***colpodea*** (Th.Fr.) Tzvelev (P. alpigena var. colpodea (Th.Fr.) Schol.), Fig. 27H–Bulbiferous kentucky bluegrass | Circumpolar

Porsild (1955) observed this taxon at Ulukhaktok and the head of Minto Inl. and later mapped these unvouchered records (Porsild 1957, 1964, Porsild and Cody 1980). Aiken et al. (2007) additionally recorded it from Cambridge Bay (voucher not located), Storkerson P. and Washburn L. Thannheiser et al. (2001) additionally recorded it from Hadley B. Newly recorded from Greiner L. and Namaycush L. This viviparous taxon is widespread across the Canadian Arctic Archipelago and rare on the adjacent mainland (Aiken et al. 2007).

**NUNAVUT. Greiner L.**: *Ponomarenko VI-297* (CAN). **Namaycush L.**: *Edlund* 29 (CAN). **Storkerson P.**: *Edlund 207*, *245* (CAN). **Washburn L.**: *Edlund & Argus 12794* (CAN).

***Poa pratensis*** L. subsp. ***pratensis***, Fig. 27I–Kentucky bluegrass | European–Asian?

Newly recorded from Cambridge Bay, the first record for Victoria I. and the Canadian Arctic Archipelago. This non-native taxon was found growing in highly disturbed ground in the hamlet in 2017 with *Descurainia sophioides*, Festuca rubra subsp. rubra and *Lolium perenne*; the three grasses were almost certainly seeded. Persistence of the species at the site beyond 2017 requires confirmation.

**NUNAVUT. Cambridge Bay**: *Saarela & Teeter 5299* (CAN).

### *Puccinellia* Parl. [11]

**Key to *Puccinellia* [adapted from Davis and Consaul (2007), Consaul et al. (2008a) and Consaul et al. (2008b)]**

**Table:** 

1	Plants stoloniferous perennials, forming low, often extensive mats; most plants lacking inflorescences, the spikelets, when present, usually not producing mature pollen or caryopses	**P. phryganodes subsp. neoarctica**
–	Plants annual, biennial, or cespitose perennials, sometimes stoloniferous but not mat-forming; plants reproducing sexually, forming mature pollen and caryopses	**2**
2	Palea veins with curly, intertwined hairs proximally, scabrous distally	**3**
–	Palea veins glabrous, shortly ciliate or with fewer than five longer hairs proximally, never with curly intertwined hairs, scabrous or smooth distally	**5**
3	Pedicels smooth; apical margins of the lemmas smooth, veins obscure or distinct	***P. vahliana***
–	Pedicels scabrous; apical margins of the lemmas scabrous, sometimes minutely so, veins obscure	**4**
4	Lemmas 3.5–5.2 mm; panicles (4–)5–13 cm; rachilla between first and second lemma 1–1.7 mm	***P. angustata***
–	Lemmas 2.8–3.8 mm; panicles 1–4 cm; rachilla between first and second lemma 0.8–1.3 mm	***P. bruggemannii***
5	Lemma margins smooth or with a few scabrules at and near the apices	**6**
–	Lemma margins densely scabrous at and near the apices	**8**
6	Lemmas 2–2.5 mm, usually purple with whitish margins, veins distinct, apices obtuse to truncate; lemmas and palea veins smooth and glabrous; pedicels smooth	***P. tenella***
–	Lemmas 2.4–4.6 mm, variously coloured, margins not white, veins obscure to distinct, apices acute to truncate; lemmas and palea veins glabrous or hairy on the lower portion, often scabrous distally; pedicels smooth or scabrous	**7**
7	Panicles with (2)3–5 branches at the lowest node; lemmas 2.5–3.7 mm, veins obscure to distinct, apices entire or slightly erose; anthers (0.9–)1.2–2.2 mm	***P. arctica***
–	Panicles usually with 2 branches at the lowest node; lemmas 3–4.5 mm, veins obscure, apices irregularly serrate or erose; anthers 0.8–1.2 mm	***P. andersonii***
8	Anthers 1.2–2.2 mm	***P. arctica***
–	Anthers 0.4–1.2 mm	**9**
9	Lemmas 2.8–4 mm; panicles usually barely exserted from the sheaths	***P. vaginata***
–	Lemmas 1.7–2.8 mm; panicles usually distinctly exserted from the sheaths	**10**
10	Plants 50–80 cm tall; panicles 11–25 cm, diffuse, branches lax; lemmas 2–2.8 mm	***P. nuttalliana***
–	Plants 5–20 cm tall; panicles 2–8 cm, slender, branches usually erect; lemmas 1.7–2.3 mm	***P. banksiensis***

***Puccinellia andersonii*** Swallen, Fig. 27J–Anderson’s alkaligrass | North American (N)

Previously recorded from Cambridge Bay and Ulukhaktok (Porsild 1955, 1957, 1964, Porsild and Cody 1980, Aiken et al. 2007). A record mapped from Hadley B. (*Edlund 329*) in Aiken et al. (2007) has been redetermined to *P. bruggemannii*. Thannheiser et al. (2001) additionally recorded it from Hadley B., Johansen B. (conf.), the head of Minto Inl. (conf.) and Richardson I. Newly recorded from Boot Inl., Murray Pt. and Read I. Elsewhere in the Canadian Arctic recorded from Axel Heiberg, Banks, Baffin, Devon, Ellesmere, King William, Melville and Prince Patrick islands and a few mainland sites (Porsild and Cody 1980, Aiken et al. 2007, Saarela et al. 2013). This is a littoral species.

**NORTHWEST TERRITORIES. Boot Inl.**: *Dutilly 18717* (QFA). **Minto Inl. (head)**: *Edlund 160A* (CAN). **Ulukhaktok**: *Porsild 17245* (ALTA, CAN). **NUNAVUT. Cambridge Bay**: *Gillespie & Consaul 6324*, *6325* (CAN), *Gillespie et al. 8377* (ALA, CAN, MT, O, US), 8376 (ALA, ALTA, BABY, CAN, MT, O, UBC, US), *Polunin s.n.* (CAN), *Ponomarenko VI-068*, *VI-328* (CAN). **Johansen B.**: *Gillespie et al. 8022b* (CAN), *8138b* (CAN, O). **Murray Pt.**: *Gillespie et al. 8222b* (CAN). **Read I.**: *Oldenburg 42-493*, *43-940* (CAN)

***Puccinellia angustata*** E.L.Rand & Redfield, Fig. 27K–Narrow alkaligrass | Circumpolar

Previously recorded from Cambridge Bay, Kuujjua R., the head of Minto Inl. and Storkerson P. (Porsild 1955, 1957, 1964, Porsild and Cody 1980, Aiken et al. 2007). Newly recorded from Boot Inl., “Oldenburg L.”, Oterkvik Pt., Peel Pt. and Ulukhaktok. Elsewhere in the Canadian Arctic recorded across the islands and on the Melville P. (Aiken 2007, Consaul et al. 2008b). The species reaches its southern limit in the western Canadian Arctic on Victoria I. in the Cambridge Bay and Oterkvik Pt. areas. This is a non-littoral species.

**NORTHWEST TERRITORIES. Boot Inl.**: *Gillespie et al. 9656* (CAN, NFLD). **Kuujjua R.**: *Edlund* 681 (CAN). **Minto Inl. (head)**: *Gillespie et al. 10079* (CAN, US), *10096, 10301* (CAN, NFLD, O, US), *10294* (CAN, NFLD, US), *10298* (CAN, NFLD). “**Oldenburg L.**”: *Oldenburg 45-1362* (CAN). **Peel Pt.**: *Edlund 426* (CAN). **Ulukhaktok**: *Saarela & Bull 1493* (CAN, O, US). **NUNAVUT. Cambridge Bay**: *Bennett 13-0266* (BABY, CAN), *Edlund & Argus 12654*, *12694* (CAN), *Gillespie 5836* (CAN), *Gillespie & Consaul 6313*, *6329*, *6337* (CAN), *8446* (CAN), *Gillespie et al. 8473* (CAN, O), *Polunin s.n.* (CAN, 2 sheets), *Porsild 21593*, *21594*, *21595*, *21598* (CAN), *Stephens 1074* (CAN). **Oterkvik Pt.**: *Gillespie et al. 7559*, *7567* (CAN). **Storkerson P.**: *Edlund 330* (CAN).

***Puccinellia arctica*** (Hook.) Fernald *&* Weath. (*P. agrostidea* T.J.Sørensen, *P. poacea* T.J.Sørensen), Figs 27L, Fig. 28E, F–Arctic alkaligrass | American Beringian–North American

Previously recorded from Cambridge Bay, Collinson P. and Namaycush L. (Porsild and Cody 1980, Aiken et al. 2007). Thannheiser et al. (2001) additionally recorded it from Richardson I., Ulukhaktok (conf.) and Wellington B. Newly recorded from Boot Inl., Clouston B., Johansen B., Kuujjua R., the head of Minto Inl., Oterkvik Pt., Read I. and Richard Collinson Inl. Elsewhere in the Canadian Arctic recorded from Axel Heiberg, Banks and Ellesmere islands (Aiken et al. 2007) and scattered mainland collections (Cody et al. 1984a, Gould and Walker 1997, Davis and Consaul 2007, Saarela et al. 2013, Saarela et al. 2017b). This is a non-littoral species.

**NORTHWEST TERRITORIES. Boot Inl.**: *Gillespie et al. 9635*, *9646* (CAN, NFLD, US), *9654* (CAN), *9659* (CAN, NFLD), *9660* (ALA, CAN, NFLD, O), *9661* (CAN, NFLD, US). **Kuujjua R.**: *Edlund 659* (CAN), *Gillespie et al. 9719* (ALA, CAN, NFLD, O, US), *9720* (CAN, NFLD), *9910* (CAN, NFLD, US), *9914* (CAN, O). **Minto Inl. (head)**: *Gillespie et al. 10279* (CAN, NFLD, US). **Richard Collinson Inl.**: *Edlund 683*, *696* (CAN). **Ulukhaktok**: *Edlund 368*, *767*, *858*, *868*, *873* (CAN). **NUNAVUT. Cambridge Bay**: *Gillespie & Consaul 6321*, *6322*, *6335*, *6336*, *6342*, *6343* (CAN), *Gillespie et al. 8487*, *8506* (CAN), *Polunin s.n.* (CAN), *Porsild 21597*, *21599* (CAN), *Stephens 1096* (CAN). **Clouston B.**: *Gillespie et al. 7735b*, *7746* (CAN, O), *7735b-4* (ALA, CAN). **Collinson P.**: *Edlund & Argus 12759* (CAN). **Greiner L.**: *Ponomarenko VI*-*321*, *VI-336C* (CAN). **Hadley B.**: *Edlund 120* (cf.) (CAN) **Johansen B.**: *Gillespie et al. 7993-1* (ALA, CAN, MT, O, UBC, US), *7878*, *7879, 8131* (CAN, O), *7880* (ALA, CAN, O), *8001-1* (CAN). **Namaycush L.**: *Edlund 37*, *142* (CAN). **Oterkvik Pt.**: *Gillespie et al. 7569*, *7581*, *7589*, *7616b*, *7636*, *7793* (CAN). **Read I.**: *Oldenburg 42-486*, *43-892* (CAN).

**Figure 29.**
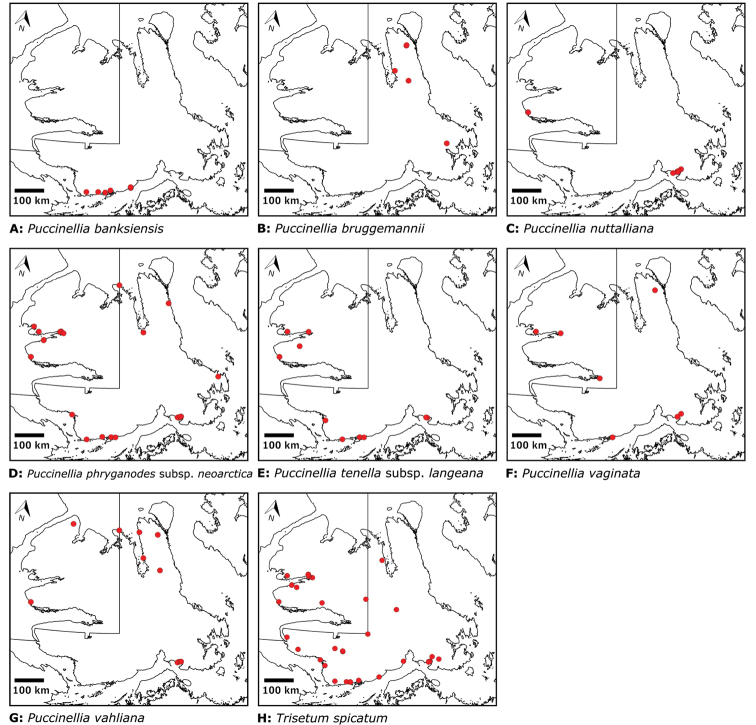
Species distribution maps. Poaceae: **A***Puccinellia banksiensis***B***Puccinellia bruggemannii***C***Puccinellia nuttalliana***D**Puccinellia phryganodes subsp. neoarctica**E**Puccinellia tenella subsp. langeana**F***Puccinellia vaginata***G***Puccinellia vahliana***H***Trisetum spicatum*.

***Puccinellia banksiensis*** Consaul, Fig. 29A–Banks island alkaligrass | American Beringian

Known from six localities discovered in 2008 along southern Victoria I. in the vicinities of Johansen Bay, Oterkvik Pt. and Sinclair Cr.; see details in Gillespie et al. (2015), including photographs. This species was assessed as May Be at Risk by Working Group on General Status of NWT Species (2016).

**NORTHWEST TERRITORIES. Johansen B.**: *Gillespie et al. 8055*, *8077*, *8146*, *8055-2* (CAN). **Oterkvik Pt.**: *Gillespie et al. 7549* (CAN). **Sinclair Cr.**: *Gillespie et al. 8261* (ALA, CAN, MT, O), *8339* (ALA, CAN, MT, O, UBC, US), *8240* (CAN).

***Puccinellia bruggemannii*** T.J.Sørensen, Fig. 29B – Bruggemann’s alkaligrass | North American (N)

Previously recorded from Albert Edward B., Hadley B. and Storkerson P. (Aiken 2007, Consaul et al. 2008b). Porsild and Cody (1980) mapped it for the Cambridge Bay area but we have not seen a supporting voucher. A collection (*Edlund 160*) from the head of Minto Inl. mapped by Aiken et al. (2007) and Consaul et al. (2008b) has been redetermined as *P. andersonii*. This is a High Arctic taxon that reaches its southern limit in the Albert Edward B. area of Victoria I. and adjacent King William I. (Consaul et al. 2008b). It is otherwise known from Somerset and most of the Queen Elizabeth islands (Aiken et al. 2007). In Northwest Territories the species is given a status rank of Sensitive (Working Group on General Status of NWT Species 2016). It is not known from the Northwest Territories portion of Victoria I.

**NUNAVUT. Albert Edward B.**: *Edlund & Argus 12743* (CAN). **Hadley B.**: *Edlund 329* (CAN). **Storkerson P.**: *Edlund 195*, *214*, *227* (CAN).

**Figure 30.**
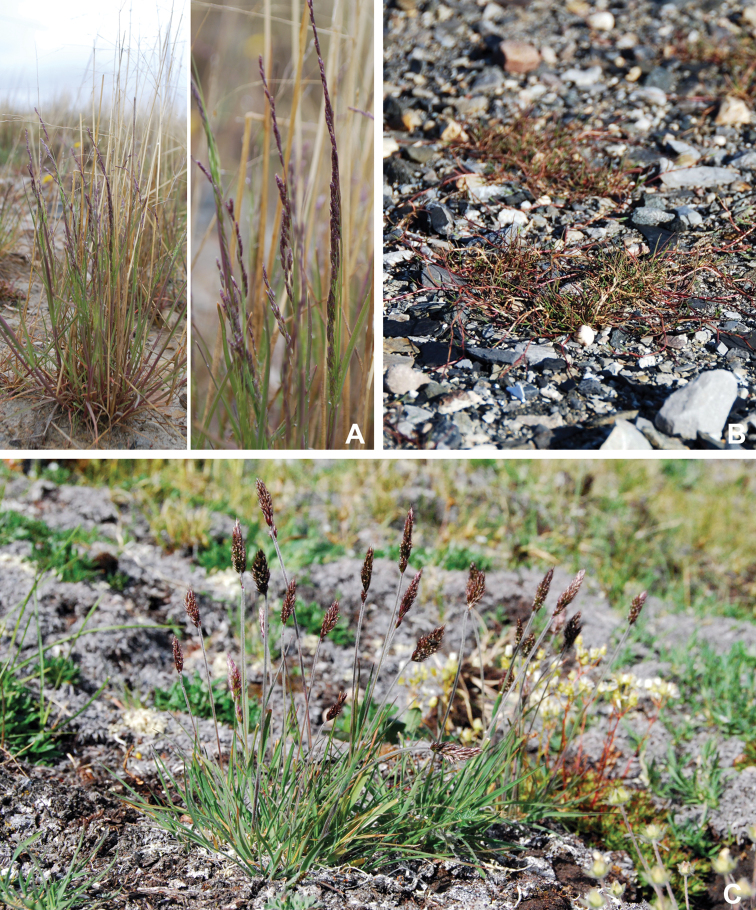
**A***Puccinellia nuttalliana* habit (left) and inflorescences (right) **B**Puccinellia phryganodes subsp. neoarctica habit, *Gillespie et al. 9644j***C***Trisetum spicatum* habit, *Gillespie et al. 10092*. Photos **A** by B.A. Bennett and **B**, **C** by J.M. Saarela.

***Puccinellia nuttalliana*** (Schult.) Hitchc. (*P. borealis* Swallen, *P. deschampsioides* T.J.Sørensen, *P. interior* T.J.Sørensen), Figs 29C, 30A–Nuttall’s alkali grass | Amphi-Pacific/Beringian–North American

Previously recorded from Cambridge Bay and Ulukhaktok (Porsild and Cody 1980, Aiken et al. 2007), the only places where we also made collections. The specimen known from Cambridge Bay by Porsild (*Stephens* 1074) has been re-determined to *P. angustata*. Elsewhere in the Canadian Arctic recorded from scattered mainland sites (Gould and Walker 1997, Davis and Consaul 2007, Saarela et al. 2013, Saarela et al. 2017b), and widely distributed below tree line in northwestern North America (Porsild and Cody 1980, Cody 2000). A non-littoral species. This conspicuous species may have been introduced into the two communities on the island. The first collection from Cambridge Bay was gathered by Gillespie in 1994 and the first ones from Ulukhaktok by Edlund in 1982.

**NORTHWEST TERRITORIES. Ulukhaktok**: *Edlund 807*, *874*, *877*, *879* (CAN, US), *Saarela & Bull 1444*, *1445* (CAN, NFLD, US), *1446* (CAN, NFLD, O, US). **Cambridge Bay**: *Bennett et al. 13-0535* (CAN), *13-0263* (ALA, CAN), *13-0283* (ALA, CAN, DAO, UBC), *13-0284b* (ALA, DAO), *Gillespie 5844* (CAN), *Gillespie & Consaul 6327*, *6340*, *6348* (CAN), *Gillespie et al. 8381* (ALA, CAN, MT, O, UBC, US), *8445* (ALA, CAN, O), *Ponomarenko VI-069* (CAN), *Saarela & Teeter 5285* (CAN).

***Puccinellia phryganodes*** subsp. ***neoarctica*** (Á.Löve *&* D.Löve) Elven, Figs 29D, 30B–Creeping alkaligrass | North American (N)

Reported by Porsild (1955) and Porsild (1957) from Ulukhaktok, the head of Minto Inl., Prince Albert S. and Read I.; of these sites he vouchered only Ulukhaktok. Aiken et al. (2007) mapped these sites (with a more recent collection confirming occurrence at Minto Inl.), as well as records from Cambridge Bay, Collinson P., and the east side of Storkerson P. Thannheiser et al. (2001) additionally recorded it from Johansen B. (conf.), Hadley B. (conf.), Richardson I. and Wellington B. (conf., from mouth of Ferguson R. at Wellington B.; *Hainault 2127*) Newly recorded from Boot Inl., Kuujjua R., Murray Pt, Oterkvik Pt. and Walker B. Widespread throughout the Canadian Arctic Archipelago and recorded from several mainland sites along the north coast (Porsild and Cody 1980, Cody et al. 1989, Korol 1992, Aiken et al. 2007, Saarela et al. 2013, Saarela et al. 2017b). This is a littoral species usually forming dense mats (spreading by stolons) at or just below the high tide line.

**NORTHWEST TERRITORIES. Boot Inl.**: *Gillespie et al. 9644a* (CAN, NFLD). **Kuujjua R.**: *Dutilly 18820* (QFA), *Gillespie et al. 9940* (CAN). **Minto Inl. (head)**: *Edlund 160* (CAN), *Gillespie et al. 10234*, *10235a*, *10235b* (CAN), *10237a* (CAN, NFLD), *10237b*, *10272a*, *10272b* (CAN). **Natkusiak P.**: *Edlund 70* (CAN). **Ulukhaktok**: *Edlund 316*, *897* (CAN), *Porsild 17242*, *17243* (CAN). **Walker B.**: *Oldenburg 45-1455* (CAN). **NUNAVUT. Cambridge Bay**: *Bennett et al. 14-0377* (BABY), *13-0278* (chars, od), *Gillespie 5850* (CAN), *Gillespie et al. 8485*, *8466* (CAN), *8478* (CAN, O), *Stephens 1207* (CAN). **Collinson P.**: *Edlund & Argus 12758* (CAN). **Ferguson L. [Tahiryuaq**]: *Hainault 2127* (UAC). **Hadley B.**: *Edlund 119* (CAN). **Johansen B.**: *Gillespie et al. 8023*, *8138a* (CAN). **Murray Pt.**: *Gillespie et al. 8220* (CAN). **Oterkvik Pt.**: *Gillespie et al. 7635* (CAN). **Read I.**: *Oldenburg 43-1028* (CAN). **Storkerson P.**: *Edlund 296* (CAN).

***Puccinellia tenella*** subsp. ***langeana*** (Berlin) Tzvelev, Fig. 29E–Lange’s alkaligrass | Amphi-Beringian?–North American (N)

Thannheiser et al. (2001) recorded this taxon from Minto Inl. and Johansen B., areas where we also made collections. Newly recorded from Boot Inl., Kuujjua R., Cambridge Bay, Clouston B., Murray Pt. and Oterkvik Pt. These records close a conspicuous gap between the few known sites on Banks I. and the adjacent mainland (Aiken et al. 2007, Saarela et al. 2013) and scattered coastal sites along Melville and Simpson peninsulas, Hudson Bay, northern Quebec and Labrador and Baffin, Coats, Devon and Southampton islands (Aiken *et al.* 2007). A littoral species occurring above the high tide line. In Northwest Territories the species is given a status rank of Undetermined (Working Group on General Status of NWT Species 2016). The eight populations reported here from the Northwest Territories portion of Victoria I. indicate that a status of Secure may be appropriate.

**NORTHWEST TERRITORIES. Boot Inl.**: *Gillespie et al. 9648*, *9644b* (CAN). **Kuujjua R.**: *Gillespie et al. 9975* (CAN, NFLD, O, US). **Minto Inl. (head)**: *Gillespie et al. 10198, 10236*, (CAN), *10233* (CAN, NFLD, US). **Ulukhaktok**: *Edlund 894*, *903* (CAN). **NUNAVUT. Cambridge Bay**: *Gillespie et al. 8468*, *8486* (CAN). **Clouston B.**: *Gillespie et al. 7728a*, *7728b* (CAN). **Johansen B.**: *Gillespie et al. 8022a* (CAN). **Murray Pt.**: *Gillespie et al. 8222b*, *c* (CAN). **Oterkvik Pt.**: *Gillespie et al. 7707* (ALA, CAN, O).

***Puccinellia vaginata*** (Lange) Fernald & Welsh, Fig. 29F–Sheathed alkaligrass | Amphi-Beringian–North American (N)

Previously recorded from the head of Prince Albert S. (Porsild 1955, 1957, 1964, Porsild and Cody 1980, Aiken et al. 2007). Thannheiser et al. (2001) additionally recorded it from Cambridge Bay (conf.), Johansen B. and Ulukhaktok. Newly recorded from Boot Inl., Murray Pt. and Storkerson P. Elsewhere in the Canadian Arctic recorded from Baffin, Bylot, Devon, Ellesmere and Southampton islands and scattered mainland sites (Porsild and Cody 1980, Aiken et al. 2007, Davis and Consaul 2007, Saarela et al. 2013, Saarela et al. 2017b). A littoral species. This species was assessed as Sensitive by the Working Group on General Status of NWT Species (2011) but the status was revised to Secure by Working Group on General Status of NWT Species (2016) in light of new information on the species in the territory.

**NORTHWEST TERRITORIES. Boot Inl.**: *Gillespie et al. 9647*, *9649* (CAN). **Minto Inl. (head)**: *Gillespie et al. 10244*, *10273* (CAN, NFLD, O). **Prince Albert S. (head)**: *Porsild 17434* (CAN). **NUNAVUT. Cambridge Bay**: *Bennett 13-0284a* (ALA, CAN), *Edlund & Argus 12653* (CAN). **Murray Pt.**: *Gillespie et al. 8221* (CAN). **Storkerson P.**: *Edlund 226* (CAN).

***Puccinellia vahliana*** (Liebm.) Scribn. & Merr (*Colpodium vahlianum* (Liebm.) Nevski), Fig. 29G–Vahl’s alkaligrass | North American (N)–Amphi-Atlantic

Previously recorded from Cambridge Bay, Hadley B. and Ulukhaktok (Porsild 1955, 1957, 1964, Aiken et al. 2007); we have not seen supporting vouchers from sites mapped by Aiken et al. (2007) inland of the head of Minto Inl. and east of Namaycush L. Thannheiser et al. (2001) additionally recorded it from the head of Minto Inl. Newly recorded from Natkusiak P., “Oldenburg L.” and Storkerson P. Widespread throughout the Canadian Arctic Archipelago and known from scattered sites on the mainland (Aiken et al. 2007, Saarela et al. 2013).

**NORTHWEST TERRITORIES. Natkusiak P.**: *Edlund 95*, *96*, *97* (CAN). “**Oldenburg L.**”: *Oldenburg 45-1367* (CAN). **Ulukhaktok**: *Porsild 17244* (CAN). **NUNAVUT. Cambridge Bay**: *Edlund & Argus 12881* (CAN), *Gillespie et al. 8444* (CAN, O), *8467* (CAN), *Polunin s.n.* (CAN), *Stephens 1073* (CAN). **Hadley B.**: *Edlund 317*, *375* (CAN). **Storkerson P.**: *Edlund 206*, *278* (CAN).

### *Trisetum* Pers. [1]

***Trisetum spicatum*** (L.) K.Richt., Figs 29H, Fig. 30C–Narrow false-oat | Circumpolar-alpine

Previously recorded from south of Burns L., Cambridge Bay, Hadley B., the head of Minto Inl., Mt. Bumpus, Namaycush L., southeast of the head of Prince Albert S., Read I., Ulukhaktok and Wollaston P. (Porsild 1955, 1957, 1964, Porsild and Cody 1980, Aiken et al. 2007). Thannheiser et al. (2001) additionally recorded it from Johansen B. (conf.) and Surrey L. Newly recorded from “30-Mile Cr.”, Boot Inl., Clouston B., Colville Mts., Greiner L., Oterkvik Pt., the north side of Prince Albert S. and Sinclair Cr. Widespread throughout the Canadian Arctic (Porsild and Cody 1980, Cody et al. 1989, Korol 1992, Cody and Reading 2005, Aiken et al. 2007, Saarela et al. 2013, Saarela et al. 2017b). This species was recently transferred to the genus *Koeleria* Pers., as *K. spicata* (L.) Barberá, Quintanar, Soreng & P.M.Peterson, in light of polyphyly of *Trisetum* Pers. (Saarela et al. 2017a, Barberá et al. 2019).

**NORTHWEST TERRITORIES. Boot Inl.**: *Edlund 575* (CAN), *Gillespie et al. 9539* (ALA, ari, CAN, MT, O, UBC, US). **Burns L. (S)**: *Edlund 563* (CAN). **Kuujjua R.**: *Dutilly 18814* (DAO), *Gillespie et al. 9948* (ALA, CAN, MT, O), *9862* (CAN). **Minto Inl. (head)**: *Edlund 97*, *613* (CAN), *Gillespie et al. 10092* (ALA, CAN, MT, O, US), *10010* (ALA, CAN, O), *10146* (CAN, O), *Porsild 17363* (CAN). **Prince Albert S. (N)**: *Oldenburg 46-2275* (CAN). **Ulukhaktok**: *Edlund 833* (CAN), *Porsild 17246* (CAN), *Saarela & Bull 1458* (ari, CAN, MT, O). **NUNAVUT. “30-Mile Cr.**”: *Bennett et al. 14-0357* (BABY). **Cambridge Bay**: *Bennett et al. 13-0279* (BABY, chars, od, UBC), *Edlund & Argus 12693* (CAN), *Gillespie et al. 8390* (ALA, CAN, MT, O), *Polunin s.n.* (CAN), *Stephens 1256* (CAN, KSTC). **Clouston B.**: *Gillespie et al. 7734* (CAN, O). **Colville Mts.**: *Gillespie et al. 7779* (CAN). **Greiner L.**: *Ponomarenko VI-209*, *VI-276* (CAN). **Hadley B.**: *Edlund 50* (CAN). **Johansen B.**: *Gillespie et al. 7836* (ALA, CAN, O), *8091* (CAN), *8140* (CAN, O), *8151* (CAN, O). **Mt. Bumpus**: *Edlund 231*, *232*, *256* (CAN). **Namaycush L.**: *Edlund 40* (CAN). **Oterkvik Pt.**: *Gillespie et al.* 7808 (ALA, CAN, O). **Prince Albert S. (head)**: *Edlund 102* (CAN). **Read I.**: *Oldenburg 43-1020* (mixed with Calamagrostis stricta subsp. groenlandica), *43-895* (CAN). **Sinclair Cr.**: *Gillespie et al. 8350* (ALA, CAN, O). **Wollaston P.**: *D. Jenness 350a* (CAN, 2 sheets).

### Eudicots

**Ranunculales**

**Ranunculaceae [6/13]**

**Key to Ranunculaceae:**

**Table:** 

1	Fruit a follicle; ovules 15–35 per pistil	***Caltha palustris***
–	Fruit an achene; ovules 1 per pistil	**2**
2	Inflorescences with 1 or more pairs (opposite) or whorls of involucral bracts, these leaf-like or calyx-like	**3**
–	Inflorescences without involucral bracts	**5**
3	Achene beak 20 mm or more, plumose	***Pulsatilla nuttalliana***
–	Achene beak 6 mm or less, glabrous or pubescent, not plumose	**4**
4	Basal leaves simple, segments primarily 3, lateral segments unlobed or 1×-lobed; sepals yellow; achenes ovoid to oblong, 3–4 mm, glabrous, beak recurved, 4–6 mm	***Anemonastrum richardsonii***
–	Basal leaves ternate; sepals white or tinged blue; achenes obovoid, 2–2.5 mm, densely woolly, beak straight, 1–2.2 mm	***Anemone parviflora***
5	Leaves unlobed; plants stoloniferous; achenes with a distinct longitudinal vein or veins on lateral faces	***Halerpestes cymbalaria***
–	Leaves deeply lobed; plants tufted or with creeping leafy stems; achenes without distinct longitudinal veins on lateral faces	*** Ranunculus ***

### *Anemone* L. [1]

**Figure 31.**
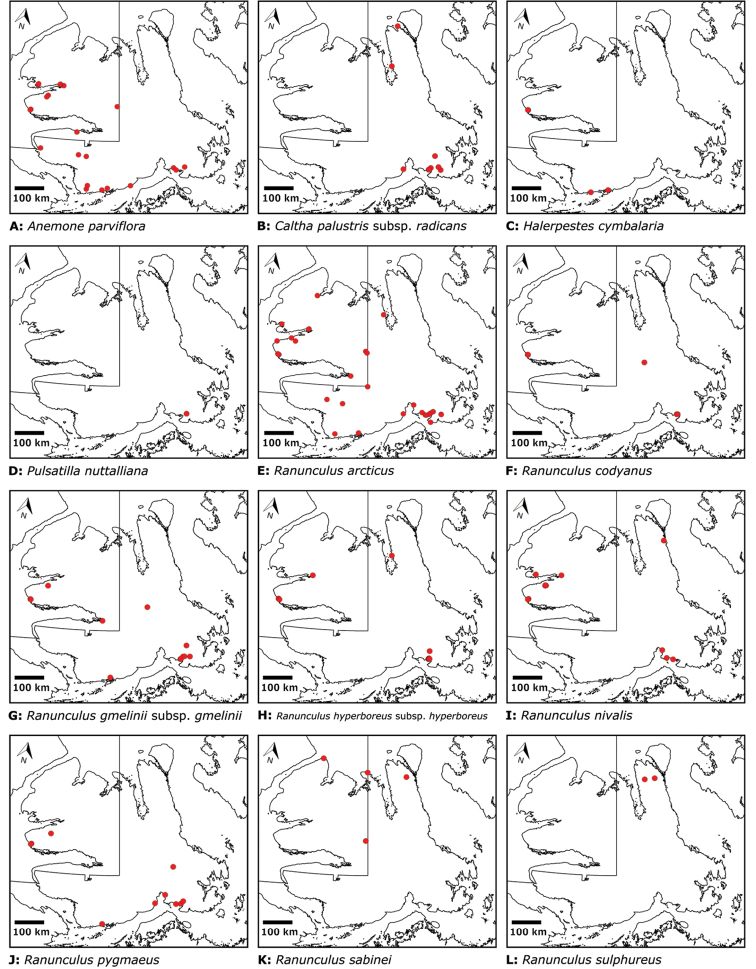
Species distribution maps. Ranunculaceae: **A***Anemone parviflora***B**Caltha palustris subsp. radicans**C***Halerpestes cymbalaria***D***Pulsatilla nuttalliana***E***Ranunculus arcticus***F***Ranunculus codyanus***G**Ranunculus gmelinii subsp. gmelinii**H**Ranunculus hyperboreus subsp. hyperboreus**I***Ranunculus nivalis***J***Ranunculus pygmaeus***K***Ranunculus sabinei***L***Ranunculus sulphureus*.

**Figure 32.**
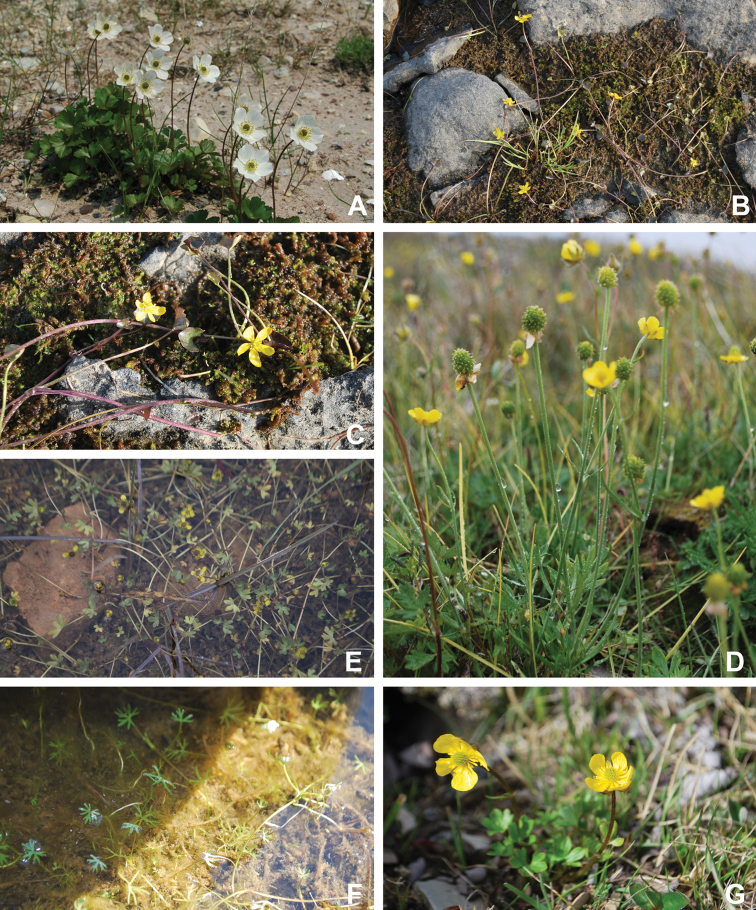
**A***Anemone parviflora* habit *Gillespie et al. 9644***B**Caltha palustris subsp. radicans habit **C**Caltha palustris subsp. radicans inflorescences **D***Ranunculus arcticus* habit **E**Ranunculus hyperboreus subsp. hyperboreus habit **F**Ranunculus gmelinii subsp. gmelinii habit **G***Ranunculus nivalis* habit. Photos **A** by R.D. Bull and **B–G** by B.A. Bennett.

***Anemone parviflora*** Michx., Figs 31A, 32A–Small-flowered anemone | Amphi-Beringian (E)–North American (N)

Previously recorded from Boot Inl., south of Burns L., Cambridge Bay (known only from Augustus Hills and the hills above Long Point), the head of Minto Inl., Mt. Pelly, south side of Prince Albert S., Wollaston P. and Ulukhaktok (Macoun and Holm 1921, Porsild 1955, 1957, 1964, Porsild and Cody 1980, Aiken et al. 2007). Thannheiser et al. (2001) additionally recorded it from Johansen B. (conf.) and Richardson I. Newly recorded from Colville Mts., Kugaluk R., Kuujjua R., Oterkvik Pt. and Sinclair Cr. Elsewhere in the Canadian Arctic recorded from Banks I. and across the mainland (Cody and Reading 2005, Aiken et al. 2007, Saarela et al. 2013, Blondeau 2015e, Saarela et al. 2017b). The Victoria I. populations mark the northeastern limit of its range.

**NORTHWEST TERRITORIES. Boot Inl.**: *Edlund 581* (CAN), *Gillespie et al. 9597* (CAN, O), *9620* (ALA, CAN, O). **Burns L. (S)**: *Edlund 555* (CAN). **Kuujjua R.**: *Gillespie et al. 9830* (CAN, WIN), *9891* (ALA, ari, CAN, MT). **Minto Inl. (head)**: *Edlund 74* (CAN), *Gillespie et al. 10136* (CAN, MT, O, WIN), *Porsild 17389* (CAN). **Prince Albert S. (S)**: *Edlund 532*, *536* (CAN). **Ulukhaktok**: *Edlund 834* (CAN), *Porsild 17287* (ALTA, CAN). **NUNAVUT. Cambridge Bay**: *Bennett et al. 13-0305* (BABY, CAN, chars), *Edlund & Argus 12856* (ALA, CAN). **Colville Mts.**: *Gillespie et al. 7765* (CAN, O). **Johansen B.**: *Gillespie et al. 8100* (ALA, ALTA, BABY, CAN, MT, O, UBC), *8166* (CAN, O). **Kugaluk R.**: *Edlund & Nixon 206* (CAN). **Ovayok TP**: *Stephens 1161* (CAN, KANU, KSTC). **Oterkvik Pt.**: *Gillespie et al. 7661* (ALA, BABY, CAN, MT, O, UBC), *7807* (ALA, CAN, O). **Sinclair Cr.**: *Gillespie et al. 8297* (CAN). **Wollaston P.**: *D. Jenness 653* (CAN).

### *Anemonastrum* Holub [1]

***Anemonastrum richardsonii*** (Hook.) Mosyakin (*Anemone richardsonii* Hook.)–Richardson’s anemone | Asian (NE)–amphi-Beringian–North American (N)

Previously reported from the “south coast” of Victoria I., gathered by Rae in 1851. Porsild (1955) confirmed the identification of the sheet at K, which he described as including five plants, of which four are this species and one *Anemone parviflora*. We accept the record on Porsild’s authority. This record was not mapped in Aiken et al. (2007), nor is it mapped here because its precise location is unknown. A collection from Minto Inlet gathered by Anderson and reported in Hooker (1856) was redetermined to *A. parviflora* (Porsild 1955). Elsewhere in the Canadian Arctic recorded from Banks I. and across the mainland (Porsild and Cody 1980, Cody and Reading 2005, Aiken et al. 2007, Saarela et al. 2013, Blondeau 2015e, Saarela et al. 2017b).

**NUNAVUT**. **South coast**: *Rae s.n.* (K, det. A.E. Porsild).

### *Caltha* L. [1]

***Caltha palustris*** subsp. ***radicans*** (T.F.Forst.) Hook., Figs 31B, 32B, C–Marsh marigold | European (N)–Asian (N)–amphi-Beringian–North American (NW)

Previously recorded from Cambridge Bay, Ferguson L., the head of Minto Inl. (Porsild obs.), Storkerson P. and Ulukhaktok (Porsild obs.) (Simmons 1913, Porsild 1955, 1957, 1964, Porsild and Cody 1980, Aiken et al. 2007). Thannheiser et al. (2001) additionally recorded it from Johansen B., Richardson I., Surrey L. and Wellington B. Newly recorded from “30-Mile Cr.”, Greiner L. and Hadley B. Elsewhere in the Canadian Arctic recorded from Banks, King William and Melville islands and mainland sites east to Hudson Bay (Porsild and Cody 1980, Gould and Walker 1997, Cody et al. 2003, Cody and Reading 2005, Aiken et al. 2007, Saarela et al. 2013, Saarela et al. 2017b).

**NUNAVUT. “30-Mile Cr.**”: *Bennett et al. 14-0364* (BABY, UBC). **Cambridge Bay**: *Bennett et al. 13-0161* (CAN, chars, O, UBC), *Calder et al. 24175* (DAO), *Hainault 3214* (DAO), *Oldenburg 44-922* (CAN), *Porsild 21616* (CAN), *Stephens 961* (CAN, KSTC), *Sweatman & Smith 14* (CAN, DAO). **Ferguson L. [Tahiryuaq**]: *Edlund & Argus 12771* (CAN). **Greiner L.**: *Ponomarenko VI-141A*, *VI-203* (CAN). **Hadley B.**: *Edlund 323* (CAN). **Storkerson P.**: *Edlund 374* (CAN).

### *Halerpestes* Greene [1]

***Halerpestes cymbalaria*** (Pursh) Greene (*Cyrtorhyncha cymbalaria* (Pursh) Britton, *Ranunculus cymbalaria* Pursh), Fig. 31C–Northern seaside buttercup | Asian (N/C) & North American

Previously recorded only from Ulukhaktok (Porsild 1955, 1957, 1964, Porsild and Cody 1980, Aiken et al. 2007), and newly recorded from Johansen B. and Oterkvik Pt. At Johansen B., this species grew along the coast on a narrow stony shoreline in the beach to tundra transition zone, with *Symphyotrichum pygmaeum* and the typical seashore species *Potentilla anserina* and *Tripleurospermum maritimum*. At Oterkvik Pt. it grew in cracks in rock near the coast within the salt spray zone. The Victoria I. populations mark the northern limit of its distribution in Canada. Elsewhere in the Canadian Arctic recorded from scattered mainland sites (Porsild and Cody 1980, Korol 1992, Cody 1996a, Saarela et al. 2013, Saarela et al. 2017b); not known from any other Canadian Arctic islands.

**NORTHWEST TERRITORIES. Ulukhaktok**: *Edlund 290* (CAN), *Porsild 17288* (CAN). **Johansen B.**: *Gillespie et al. 8005* (ALA, CAN, MT, O, UBC). **Oterkvik Pt.**: *Gillespie et al. 7682* (ALA, CAN, O), *7705* (CAN).

### *Pulsatilla* Mill. [1]

***Pulsatilla nuttalliana*** (DC.) Berchtold ex J.Presl (Anemone patens subsp. multifida Hultén), Fig. 31D–Prairie pasqueflower | Asian (N/C)–amphi-Beringian–North American (NW)

Known on Victoria I. only from “Long L.” (Aiken et al. 2007). Elsewhere in the Canadian Arctic recorded from southern Banks I. and a few mainland Northwest Territories sites (Porsild and Cody 1980, Aiken et al. 2007). Elsewhere in Nunavut known only from Akimiski I. (Blaney and Kotanen 2001). Taxonomy follows Mosyakin (2016).

**NUNAVUT. “Long L.**”: *Lambert s.n.* (CAN) (Suppl. material 5).

### *Ranunculus* L. [8]

**Key to *Ranunculus* [adapted from Porsild and Cody (1980) and Whittemore (1997)]:**

**Table:** 

1	Plants aquatic or amphibious; leafy stems creeping and rooting at nodes or floating in water, then rootless	**2**
–	Plants terrestrial or paludal; leafy stems erect or if decumbent rooting only at base, never floating	**4**
2	Plants aquatic; stems floating; leaves filiform-dissected; petals white with small yellow claw; achenes with strong, coarse wrinkles	***R. codyanus***
–	Plants amphibious; stems creeping or floating; leaves lobed, divided or dissected, but never filiform-dissected; petals yellow; achenes smooth	**3**
3	Leaf blades reniform to broadly flabellate, 0.3–1.2 cm, deeply 3-lobed or -parted, lobes undivided or lateral lobes cleft, terminal segment entire or distally crenulate; receptacle glabrous; petals 2–4 × 1–3 mm; nectary-scale a low crescent-shaped ridge surrounding nectary, nectary on petal surface; styles 0.1–0.2 mm	***R. hyperboreus***
–	Leaf blades reniform to circular, 0.6–6.5 cm, 3-parted, segments again 1–3×-lobed to -dissected; receptacle sparsely hispid; petals 3–7 × 2–5 mm; nectary-scale a free flap, crescent-shaped, funnel-shaped, or flaplike, nectary on surface of flap; styles 0.2–0.4 mm	***R. gmelinii***
4	Abaxial surface of sepals with dense brown pubescence	**5**
–	Abaxial surface of sepals glabrous or with colorless hairs	**6**
5	Receptacle glabrous; basal leaf blades 3-parted, at least lateral segments again lobed or with toothed margins	***R. nivalis***
–	Receptacle brown-pilose; basal leaf blades usually shallowly lobed, or unlobed with crenate margins	***R. sulphureus***
6	Petals 7–15 mm; leaf blades pedately (5–)7(–9)-parted or -divided, segments undivided or again lobed or parted; flowering stems 6–33(–45) cm	***R. arcticus***
–	Petals 1–8 mm; leaf blades 3-lobed or -divided, segments undivided or again lobed; flowering stems 0.6–12 cm	**7**
7	Petals 1.2–3.5 × 1.1–2.8 mm; sepals 2–4 × 1.2–1.6 mm; pedicels glabrous or pubescent; flowering stems 0.6–3.5 cm (sometimes longer in fruit); leaf blades 0.45–0.9 × 0.6–1.3 cm; heads of achenes nearly globose to cylindric, 2.5–7 × 2.5–5 mm	***R. pygmaeus***
–	Petals 5–8 × 3–4 mm; sepals 4–7 × 2–3 mm; pedicels pilose; flowering stems 1–12 cm; leaf blades 0.9–3 × 0.8–3.4 cm; heads of achenes cylindric, 6–9 × 4 mm	***R. sabinei***

***Ranunculus arcticus*** Richardson (R. pedatifidus var. affinis (R.Br.) L.D.Benson, R. pedatifidus var. leiocarpus (Trautv.) Fernald), Figs 31E, 32D–Northern buttercup | Circumpolar–alpine

Previously recorded from Cambridge Bay, south of Burns L., C. Colborne, the head of Minto Inl., Mt. Bumpus, Mt. Pelly, the head and southeast of the head of Prince Albert S., Richard Collinson Inl., Ulukhaktok and Wollaston P. (Macoun and Holm 1921, Porsild 1955, 1957, 1964, Porsild and Cody 1980). Newly recorded from “30-Mile Cr.”, C. Wollaston, Hadley B., Johansen B., Oterkvik Pt., Kuujjua R. and Walker B. Elsewhere in the Canadian Arctic recorded from Axel Heiberg, Baffin, Banks, Coats, Ellesmere, Fitzwilliam Owen, Melville and Southampton islands, and across the mainland (Porsild and Cody 1980, Korol 1992, Aiken et al. 2007, Saarela et al. 2013, Blondeau 2015e, Saarela et al. 2017b). Absence from eastern Victoria I. may be part of a broader distribution gap in the central Arctic islands (Aiken et al. 2007).

**NORTHWEST TERRITORIES. Burns L. (S)**: *Edlund 53*, *559* (CAN). **C. Wollaston**: *Edlund 55* (CAN). **Kuujjua R.**: *Gillespie et al. 9876* (CAN, O), *9926* (CAN). **Minto Inl. (head)**: *Edlund 50* (CAN), *Gillespie et al. 10112* (ALA, CAN, O), *10139* (CAN). **Prince Albert S. (head)**: *Edlund 156* (CAN), *Porsild 17441* (CAN). **Richard Collinson Inl.**: *Edlund 605*, *701*, *706* (CAN). **Ulukhaktok**: *Edlund 508*, *816* (CAN), *Oldenburg 45-1661* (CAN, GH), *Saarela & Bull 1453* (CAN, O, WIN). **Walker B.**: *Oldenburg 45-1533A* (CAN, GH). **NUNAVUT. “30-Mile Cr.**”: *Bennett et al. 14-0347* (UBC). **Cambridge Bay**: *Bennett 13-0223* (ALA, BABY, chars, DAO, UBC), *Edlund & Argus 12857* (CAN), *Gillespie et al. 8457* (CAN, O), *Ponomarenko VI-307* (CAN), *Stephens 1098* (CAN, KSTC), *1262* (CAN, KANU, KSTC), *976* (CAN, KANU, KSTC). **C. Colborne**: *Edlund & Argus 12734* (CAN). **Ferguson L. [Tahiryuaq**]: *Hainault 1901* (DAO). **Greiner L.**: *Ponomarenko VI-120*, *VI-146* (CAN). **Hadley B.**: *Edlund 80* (CAN). **Johansen B.**: *Gillespie et al. 7816* (ALA, CAN, MT, O). **Mt. Bumpus**: *Edlund 255* (CAN). **Ovayok TP**: *Gillespie et al. 8429* (ALA, ALTA, BABY, CAN, MT, O, UBC, US), *Stephens 1163* (CAN, KANU, KSTC). **Oterkvik Pt.**: *Gillespie et al. 7535* (ALA, CAN, O). **Prince Albert S. (head)**: *Edlund 84* (CAN). **Wollaston P.**: *D. Jenness 654* (CAN).

***Ranunculus codyanus*** B.Boivin, Fig. 31F–Cody’s buttercup | Amphi-Beringian (E)–North American (N)

Previously recorded from Cambridge Bay (Aiken et al. 2007) and newly recorded from Namaycush L. and Ulukhaktok. Thannheiser et al. (2001) additionally recorded it from Johansen B., Surrey L. and Wellington B. Taxonomy follows Wiegleb et al. (2017), who revised Ranunculus sect. Batrachium from a global perspective. Plants now recognized as this taxon were previously treated under R. aquatilis var. diffusus With., R. aquatilis var. eradicatus Laest. and *R. subrigidus* W.G.Drew.

**NORTHWEST TERRITORIES. Ulukhaktok**: *Edlund 510*, *817* (CAN), *820* (CAN, US), *Oldenburg 45-1660* (CAN). **NUNAVUT. Cambridge Bay**: *Edlund & Argus 12879* (CAN), *Stephens 1055* (CAN, KSTC), *1097* (CAN). **Namaycush L.**: *Edlund 169* (CAN).

***Ranunculus gmelinii*** DC. subsp. ***gmelinii***, Figs 31G, 32F–Gmelin’s buttercup | European (NE)–Asian (N/C)–amphi-Beringian–North American (NW)

Previously recorded from Cambridge Bay, Ferguson L., Namaycush L., the head of Prince Albert S. and Ulukhaktok (Porsild 1955, 1957, 1964, Porsild and Cody 1980, Aiken et al. 2007). Thannheiser et al. (2001) additionally recorded it from Johansen B. (conf.), Surrey L. and Wellington B. Newly recorded from Kuujjua R., Greiner L. and Mt. Pelly. Elsewhere in the Canadian Arctic recorded from Banks, Eglinton, Fitzwilliam Owen, Melville and Prince Patrick islands and scattered sites across the mainland (Korol 1992, Cody 1996a, Aiken et al. 2007, Saarela et al. 2013, Blondeau 2015e, Saarela et al. 2017b).

**NORTHWEST TERRITORIES. Kuujjua R.**: *Gillespie et al. 9875* (CAN). **Prince Albert S. (head)**: *Porsild 17440* (CAN). **Ulukhaktok**: *Edlund 760*, *830* (CAN). **NUNAVUT. Cambridge Bay**: *Oldenburg 44*-*948B* (CAN), *Porsild 21617* (CAN), *Stephens 1099* (CAN, KSTC). **Ferguson L. [Tahiryuaq**]: *Edlund & Argus 12776* (CAN). **Greiner L.**: *Ponomarenko VI-203L* (CAN). **Johansen B.**: *Gillespie et al. 8072* (ALA, CAN, O). **Ovayok TP**: *Bennett et al. 13-0296* (chars, od), *Stephens 1065* (CAN, KSTC). **Namaycush L.**: *Edlund & Roncato-Spencer 116* (CAN).

***Ranunculus hyperboreus*** Rottb. subsp. ***hyperboreus***, Figs 31H, 32E–Far-northern buttercup | Circumpolar-alpine

Previously recorded from Cambridge Bay, Hadley B., the head of Minto Inl., Mt. Lady Pelly and Ulukhaktok (Porsild 1955, 1957, 1964, Porsild and Cody 1980, Aiken et al. 2007). Thannheiser et al. (2001) additionally recorded it from Johansen B., Surrey L. and Wellington B. Elsewhere in the Canadian Arctic recorded from Banks, Bathurst, Bylot, Coats, Cornwallis, Devon, Eglinton, Ellef Ringnes, Ellesmere, Igloolik, King William, Melville, Prince Charles, Prince Patrick, Resolution, Somerset and Southampton islands, and across the across the mainland (Porsild and Cody 1980, Cody et al. 1984a, Cody et al. 1989, Korol 1992, Aiken et al. 2007, Saarela et al. 2013, Blondeau 2015e, Saarela et al. 2017b).

**NORTHWEST TERRITORIES. Minto Inl. (head)**: *Porsild 17390* (CAN). **Mt. Lady Pelly [Amaaqtuq**]: *Jones & Hainault s.n.* (DAO). **Ulukhaktok**: *Edlund 509*, *511*, *818*, *821* (CAN), *Saarela & Bull 1449* (ALA, CAN, O, WIN). **NUNAVUT. Cambridge Bay**: *Bennett et al. 13-0265* (ALA, CAN, chars, od, UBC), *Edlund & Argus 12691* (CAN), *Gillespie et al. 8494* (ALA, ALTA, BABY, CAN, MT, O, UBC, US), *8501* (ALA, BABY, CAN, MT, O, UBC), *Oldenburg 44*-*948A* (CAN), *Saarela & Teeter 5286* (CAN), *Stephens 1149* (CAN, KSTC). **Hadley B.**: *Edlund 322* (CAN).

***Ranunculus nivalis*** L., Figs 31I, 32G–Snow buttercup | Circumpolar

Previously recorded from Cambridge Bay, the head of Minto Inl., Storkerson P. and Ulukhaktok (Porsild 1955, 1957, 1964, Porsild and Cody 1980, Aiken et al. 2007). Thannheiser et al. (2001) additionally recorded it from Johansen B. Newly recorded from Boot Inl. and Kuujjua R. A collection mapped in Aiken et al. (2007) as this species (*Edlund 558*) from Burns L. has been redetermined as *R. sabinei*. Elsewhere in the Canadian Arctic recorded from Axel Heiberg, Baffin, Banks, Bathurst, Bylot, Cameron, Devon, Digges, Eglinton, Ellef Ringnes, Ellesmere, Lougheed, Massey, Melville, Nottingham, Prince Patrick, Somerset, Southampton, Upper Savage and West Foxe islands and across the mainland (Porsild and Cody 1980, Cody et al. 2003, Aiken et al. 2007, Saarela et al. 2013, Blondeau 2015e, Saarela et al. 2017b).

**NORTHWEST TERRITORIES. Boot Inl.**: *Gillespie et al. 9611* (CAN, O). **Kuujjua R.**: *Gillespie et al. 9757*, *9999* (CAN), *9816* (ALA, ari, CAN, O, US). **Minto Inl. (head)**: *Porsild 17391* (CAN). **Ulukhaktok**: *Edlund 724*, *837* (CAN), *Porsild 17289* (CAN). **NUNAVUT. Cambridge Bay**: *Bennett et al. 13-0306* (ALA, CAN), *Edlund & Argus 12872* (CAN). **Ferguson L. [Tahiryuaq**]: *Hainault 2002* (DAO). **Storkerson P.**: *Edlund 284* (CAN).

***Ranunculus pygmaeus*** Wahlenb., Fig. 31J–Pygmy buttercup | Circumpolar–alpine

Previously recorded from Boot Inl. (*Edlund 586*, CAN, voucher not located), Cambridge Bay, Ulukhaktok and an unnamed lake ca. 60 mi. N of Cambridge Bay (Porsild 1955, 1957, 1964, Porsild and Cody 1980, Aiken et al. 2007). Thannheiser et al. (2001) additionally recorded it from Johansen B. (conf.) and Surrey L. Newly recorded from “30-Mile Cr.”, Ferguson L. and Kuujjua R. Elsewhere in the Canadian Arctic recorded from Baffin, Banks, Coats, Devon, Digges, Eglinton, Ellesmere, King William, Mackenzie King, Resolution and Southampton islands and across the mainland (Porsild and Cody 1980, Cody et al. 1989, Cody et al. 2003, Aiken et al. 2007, Saarela et al. 2013, Blondeau 2015e, Saarela et al. 2017b).

**NORTHWEST TERRITORIES. Kuujjua R.**: *Gillespie et al. 9974* (CAN). **Ulukhaktok**: *Edlund 805*, *836* (CAN). **NUNAVUT. “30-Mile Cr.**”: *Bennett et al. 14-0334* (BABY). **Cambridge Bay**: *Bennett et al. 13-0307* (ALA, CAN), *Polunin s.n.* (CAN), *Stephens 1181* (CAN, KSTC). **Ferguson L. [Tahiryuaq**]: *Hainault 1995* (DAO). **Johansen B.**: *Gillespie et al. 8096*, *8133* (CAN). **Unnamed lake ca. 60 mi. N of Cambridge Bay**: *Porsild 17474* (CAN).

***Ranunculus sabinei*** R.Br., Fig. 31K–Sabine’s buttercup | Asian (N)–amphi-Beringian–North American (N)

Previously recorded only from Peel Pt. (Aiken et al. 2007). Thannheiser et al. (2001) additionally recorded it from Richardson I. Newly recorded from south of Burns L., Natkusiak P. and Storkerson P. Elsewhere in the Canadian Arctic recorded from Amund Ringnes, Axel Heiberg, Baffin (rare), Banks, Bathurst, Eglinton, Ellef Ringnes, Ellesmere, Fitzwilliam Owen, Jenny Lind, King William, Lougheed, Meighen, Melville, Prince of Wales, Prince Patrick and Steffanson islands and a few sites along the mainland coast east to Melville Peninsula (Porsild and Cody 1980, Aiken et al. 2007).

**NORTHWEST TERRITORIES. Burns L. (S)**: *Edlund 558* (CAN). **Natkusiak P.**: *Edlund 102* (CAN). **Peel Pt.**: *Edlund 427* (CAN). **Storkerson P.**: *Edlund 191* (CAN).

***Ranunculus sulphureus*** Sol., Fig. 31L–Sulphur buttercup | Circumpolar-alpine

Known only from Storkerson P. (Aiken et al. 2007). Elsewhere in the Canadian Arctic recorded from Axel Heiberg, Baffin, Banks, Bathurst, Bylot, Cameron, Coats, Cornwallis, Devon, Eglinton, Ellef Ringness, Ellesmere, Fitzwilliam Owen, Igloolik, Jenny Lind, King William, Meighen, Melville, Prince Charles, Prince of Wales, Prince Patrick Somerset, Southampton and Stefansson islands and a few mainland sites (Porsild and Cody 1980, Aiken et al. 2007).

**NUNAVUT. Storkerson P.**: *Edlund 168*, *247* (CAN).

### Papaveraceae [1/4]

***Papaver* L. [4]**

Taxonomy is based on a revision of Arctic island material of *Papaver* proposed by Solstad (2009); see also Aiken et al. (2007) and Elven et al. (2011). Distribution maps based on the revised taxonomy have not been published. Collections of *Papaver*, variously treated as *P. radicatum* Rottb. (Porsild 1955, 1957, Porsild and Cody 1980), *P. cornwallisense* (Porsild and Cody 1980) and *Papaver* spp. (Aiken et al. 2007) have been previously reported from Cambridge Bay, Storkerson P., Richard Collinson Inl., Ulukhaktok, Kuujjua R., Namaycush L., the north side and head of Prince Albert S., and Namaycush L. Material reported here was identified using an unpublished key (H. Solstad and R. Elven, pers. comm.).

**Figure 33.**
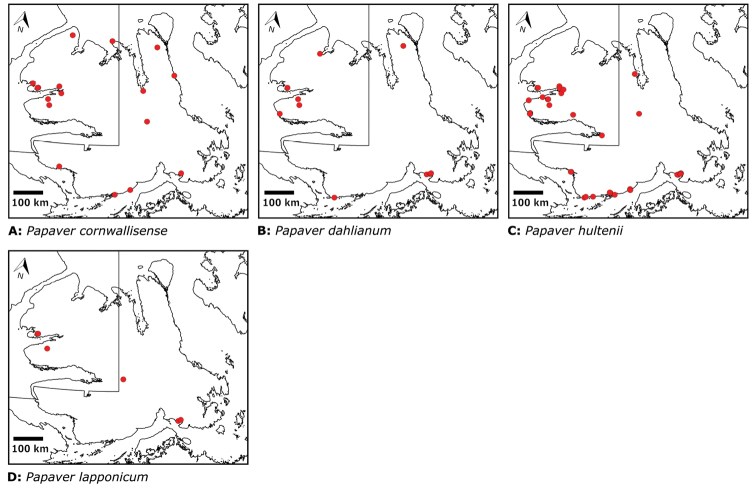
Species distribution maps. Papaveraceae: **A***Papaver cornwallisense***B***Papaver dahlianum***C***Papaver hultenii***D***Papaver lapponicum*.

***Papaver cornwallisense*** D.Löve, Fig. 33A–Cornwallis Island poppy | North American (N)–amphi-Atlantic (W)?

A collection (*P. Jenness 22*) mapped from Richard Collinson Inl. in Porsild and Cody (1980) has been redetermined as *P. dahlianum*, a taxon Porsild did not recognize. Recorded from Boot Inl., Falaise B., Greiner L., Greely Haven, Hadley B., Kuujjua R., the head of Minto Inl., Murray Pt., Namaycush L., Natkusiak P., “Oldenburg L.”, Sinclair Cr., Storkerson P. and Walker B.

**NORTHWEST TERRITORIES. Boot Inl.**: *Gillespie et al. 9636* (ALA, CAN, O), *9692* (CAN). **Kuujjua R.**: *Gillespie et al. 9840*, *9950* (CAN). **Minto Inl. (head)**: *Gillespie et al. 10090* (ALA, CAN, O), *10254* (CAN). **Natkusiak P.**: *Edlund 115* (cf.) (CAN). “**Oldenburg L.**”: *Oldenburg 45-1395* (CAN). **Walker B.**: *Oldenburg 45-1506* (CAN). **NUNAVUT. Falaise B.**: *Eriksen et al. 972* (ALA, O). **Greiner L.**: *Ponomarenko VI-043* (CAN). **Greely Haven**: *Fortier 94* (CAN). **Hadley B.**: *Edlund 148* (CAN). **Murray Pt.**: *Gillespie et al. 8188* (CAN, O). **Namaycush L.**: *Edlund & Roncato-Spencer 49* (CAN). **Sinclair Cr.**: *Gillespie et al. 8290* (CAN, O). **Storkerson P.**: *Edlund 165* (CAN).

***Papaver dahlianum*** Nordh. (P. radicatum subsp. polare Tolm., P. dahlianum subsp. polare (Tolm.) Elven), Fig. 33B, 34A–Polar poppy | North American (N)–amphi-Atlantic–European (N)-Asian (NW)

**Figure 34.**
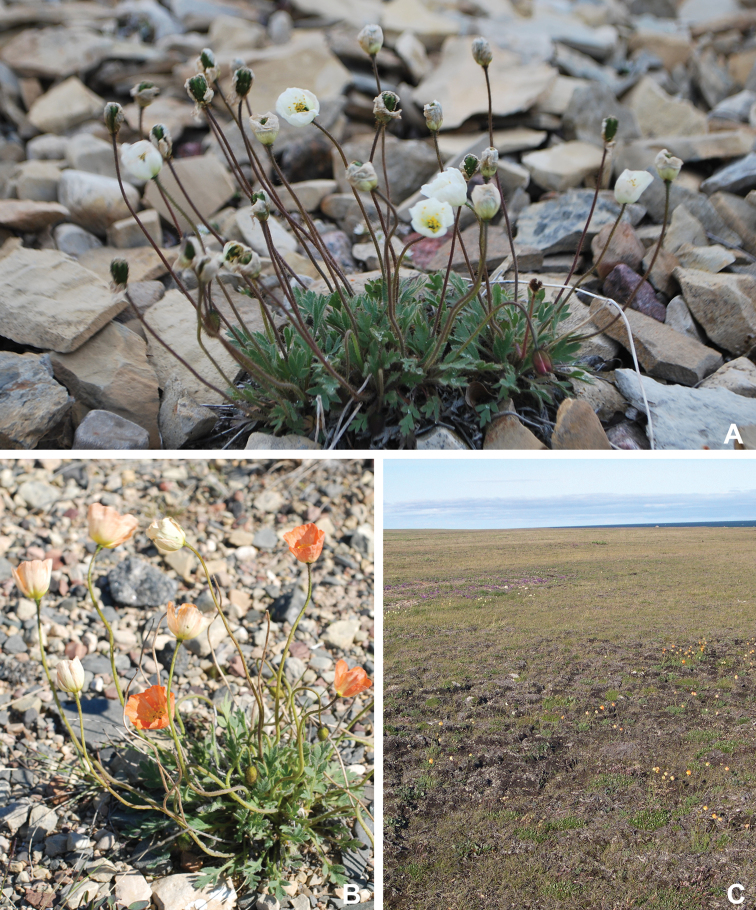
**A***Papaver dahlianum* habit **B***Papaver hultenii* habit, *Gillespie et al. 8239***C***Papaver hultenii* habitat, *Gillespie et al. 8239*. Photos **A** by B.A. Bennett and **B**, **C** by L.J. Gillespie.

Recorded from Boot Inl., Cambridge Bay, Greiner L., Kuujjua R., Oterkvik Pt., Richard Collinson Inl., Storkerson Pen. and Ulukhaktok.

**NORTHWEST TERRITORIES. Boot Inl.**: *Gillespie et al. 9694* (CAN), *9708b* (ari, CAN, MT, O). **Kuujjua R.**: *Gillespie et al. 9846*, *9962* (ALA, CAN, O). **Richard Collinson Inl.**: *P. Jenness 22* (CAN). **Ulukhaktok**: *Saarela & Bull 1465* (CAN). **NUNAVUT. Cambridge Bay**: *Bennett et al. 13-0233* (chars), *Oldenburg 44*-*929* (CAN), *Polunin s.n.* (CAN). **Greiner L.**: *Ponomarenko VI-037* (CAN). **Oterkvik Pt.**: *Gillespie et al. 7713* (CAN, O). **Storkerson P.**: *P. Jenness 36* (CAN).

***Papaver hultenii*** Knaben, Figs 33C, 34B, C–Hultén’s poppy | Amphi-Beringian?–North American (NW)

Porsild and Cody (1980) recognized this species, but did not record any occurrences of it on Victoria Island. Records mapped as *P. radicatum* in Porsild and Cody (1980) from Cambridge Bay, the head of Minto Inlet, the head of Prince Albert S., Read I. and Ulukhaktok have been redetermined as this species. The species is additionally recorded from Boot Inl., C. Wollaston, Hadley B., Johansen B., Kuujjua R., Murray Pt., Namaycush L., Oterkvik Pt. and Sinclair Cr. Although petals are typically yellow in the species, we encountered plants with orange petals at Sinclair Cr.

**NORTHWEST TERRITORIES. Boot Inl.**: *Gillespie et al. 9524a*, *9693* (CAN). **C. Wollaston**: *Edlund 48* (CAN). **Kuujjua R.**: *Gillespie et al. 9828* (ALA, CAN, MT, O), *9850, 9854b* (ari, CAN, O), *9854a, 9957* (ALA, CAN, O), *9931* (CAN). **Minto Inl. (head)**: *Edlund 120* (CAN), *Gillespie et al. 10008, 10257* (ALA, CAN, O), *10111* (ALA, ari, CAN, MT, O, WIN), *Porsild 17392* (CAN). **Prince Albert S. (N)**: *Oldenburg 46-2279* (CAN). **Prince Albert S. (head)**: *Porsild 17443* (CAN). **Ulukhaktok**: *Bandringa 304* (CAN), *Edlund 302*, *752* (CAN), *Oldenburg 42-30*, *45-1546* (CAN), *Ross 16* (ALTA), *16A* (GH), *Saarela & Bull 1456* (ALA, ALTA, ari, CAN, MO, MT, O, UBC, US, UTC, V, WIN, WTU). **NUNAVUT. Cambridge Bay**: *Bennett et al. 13-0272* (CHAR), *Edlund & Argus 12661* (CAN), *Gillespie et al. 8394*, *8481a*, *8481b* (CAN, O), *8454* (CAN), *8496* (ALA, CAN, O), *Porsild 21618* (CAN), *Smith & Sweatman 40* (CAN), *Stephens 932* (CAN, KSTC), *Sweatman & Smith 25* (CAN), *Washburn 39* (cf.) (CAN). **Hadley B.**: *Edlund 55*, *56* (CAN). **Johansen B.**: *Gillespie et al. 7899*, *8051* (CAN), *7969* (CAN, O). **Murray Pt.**: *Gillespie et al. 8211* (CAN, O). **Namaycush L.**: *Edlund & Roncato-Spencer 120* (CAN). **Oterkvik Pt.**: *Gillespie et al. 7624*, *7641* (CAN, O), *7652* (CAN), *7697* (ALA, CAN, MT, O, UBC). **Read I.**: *Oldenburg 43-904* (GH), *43-978* (CAN, GH), *Porsild 17200* (CAN), *Ross 28A* (GH), *28B* (ALTA, 2 sheets). **Sinclair Cr.**: *Gillespie et al. 8232*, *8239*, *8270*, *8354* (CAN, O), *8296* (ALA, CAN, MT, O), *8353* (AKUR, CAN, O), *8357* (AKUR, CAN).

***Papaver lapponicum*** (Tolm.) Nordh., Fig. 33D–Lapland poppy | North American (N)–amphi-Atlantic–European (N)–Asian (N)

Recorded from Boot Inl., Cambridge Bay, Kuujjua R., and the head of Prince Albert S. Subspecies (Elven et al. 2011) are not recognized here.

**NORTHWEST TERRITORIES. Boot Inl.**: *Gillespie et al. 9524b*, *9708a* (CAN), *9573* (ari, CAN, O). **Kuujjua R.**: *Edlund 626* (CAN). **NUNAVUT. Cambridge Bay**: *Gillespie et al. 8482* (AKUR, CAN, O), *Stephens 1203* (CAN, KSTC). **Prince Albert S. (head)**: *Edlund & Argus 12806* (CAN).

### Saxifragales

**Saxifragaceae [3/15]**

**Key to genera of Saxifragaceae [adapted from Wells and Elvander (2009)]:**

**Table:** 

1	Sepals 4; petals absent; stamens 2–8, usually 4 or 8	*** Chrysosplenium ***
–	Sepals 5; petals present; stamens 10	**2**
2	Flowering stems naked	*** Micranthes ***
–	Flowering stems leafy	*** Saxifraga ***


### *Chrysosplenium* L. [2]

**Key to *Chrysosplenium* [adapted from Packer (1963) and Freeman and Levsen (2007)]**

**Table:** 

1	Sepals ± equal, usually erect, sometimes spreading; stamens (3–)4, 0.3–0.4 mm; styles 0.2–0.3 mm; seeds 0.5–0.8 mm	***C. tetrandrum***
–	Sepals unequal, outer pair broader, spreading; stamens 5–8, 0.5–0.8 mm; styles 0.3–0.4(–0.5) mm; seeds 0.8–1.1. mm	***C. rosendahlii***

**Figure 35.**
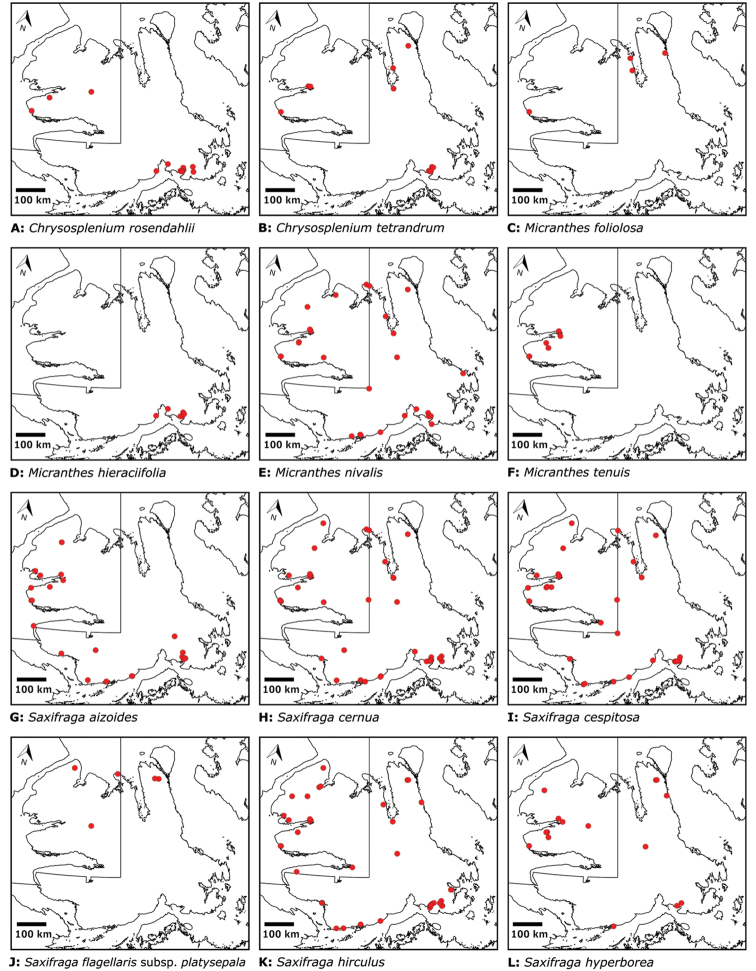
Species distribution maps. Saxifragaceae: **A***Chrysosplenium rosendahlii***B***Chrysosplenium tetrandrum***C***Micranthes foliolosa***D***Micranthes hieraciifolia***E***Micranthes nivalis***F***Micranthes tenuis***G***Saxifraga aizoides***H***Saxifraga cernua***I***Saxifraga cespitosa***J**Saxifraga flagellaris subsp. platysepala**K***Saxifraga hirculus***L***Saxifraga hyperborea*.

**Figure 36.**
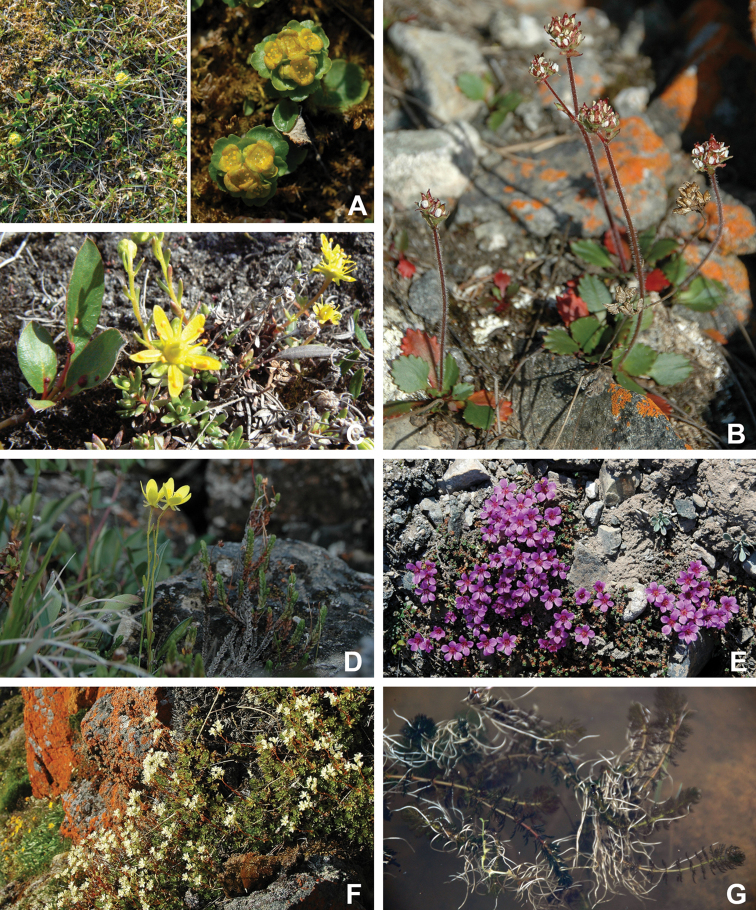
**A***Chrysosplenium rosendahlii* habitat (left) and habit (right), *Gillespie et al. 9753***B***Micranthes nivalis* habit, *Gillespie et al. 9995***C***Saxifraga aizoides* habit, Boot Inlet, NT, 12 July 2010 **D***Saxifraga hirculus* habit, Kuujjua River, NT, 18 July 2010 **E***Saxifraga oppositifolia* habit, *Gillespie et al. 9682***F***Saxifraga tricuspidata* habit, Kuujjua River, NT, 18 July 2010 **G***Myriophyllum sibiricum* habit, Johansen Bay, NU, 18 July 2008. Photos **A** by L.J. Gillespie **B**, **D–G** by R.D. Bull and **C** by P.C. Sokoloff.

***Chrysosplenium rosendahlii*** Packer, Figs 35A, 36A–Rosendahl’s golden-saxifrage | Amphi-Beringian–North American (N)

Aiken et al. (2007) included this species in *C. tetrandrum*. Known from “30-Mile Cr.”, Cambridge Bay, Greiner L., “Jackpot L.”, Kuujjua R. and Ulukhaktok. Elsewhere in the Canadian Arctic recorded from Baffin, Banks, King William, Somerset and Southampton islands and scattered mainland sites (Packer 1963, Saarela et al. 2017b). Freeman and Levsen (2007) did not include Northwest Territories in its distribution. The collections cited here from the Northwest Territories portion of Victoria I., as well as in the protologue (Packer 1963), confirm its presence in the territory.

**NORTHWEST TERRITORIES. “Jackpot L.**”: *Porsild 17503* (CAN). **Kuujjua R.**: *Gillespie et al. 9753* (ALA, CAN, O). **Ulukhaktok**: *Edlund 501* (CAN). **NUNAVUT. “30-Mile Cr.**”: *Bennett et al. 14-0329* (BABY). **Cambridge Bay**: *Bennett 13-0228* (BABY, chars), *13-0170* (od), *Consaul & Gillespie 1109* (CAN, mixed with *C. tetrandrum*), *Edlund & Argus 12619* (CAN), *Gillespie et al. 8498* (ALA, CAN, MT, O), *Porsild 21632* (CAN), *Stephens 949* (CAN, KANU), *Sweatman & Smith 5* (CAN). **Greiner L.**: *Ponomarenko VI-082*, *VI-137*, *VI-182* (CAN).

***Chrysosplenium tetrandrum*** Th.Fr., Fig. 35B–Northern golden-saxifrage | Circumpolar & Cordilleran

Previously recorded from Cambridge Bay, Hadley B., the head of Minto Inl., Storkerson P. and Ulukhaktok. Elsewhere in the Canadian Arctic recorded from Baffin, Banks, Bylot, Coats, Cornwallis, Devon, Digges, Eglinton, southern Ellesmere, Igloolik, King William, Melville, Prince of Wales, Somerset and Southampton islands, and across the mainland (Packer 1963, Saarela et al. 2013).

**NORTHWEST TERRITORIES. Minto Inl. (head)**: *Edlund 178* (CAN), *Gillespie et al. 10142* (ALA, CAN, O). **Ulukhaktok**: *Edlund 813* (CAN). **NUNAVUT. Cambridge Bay**: *Consaul & Gillespie 1109* (CAN, mixed with *C. rosendahlii*), *Edlund & Argus 12877* (CAN), *Polunin s.n.* (CAN), *Porsild 17470* (CAN), *Stephens 1124* (CAN, KANU), *878* (CAN). **Greiner L.**: *Ponomarenko VI-329a*, *VI-341C* (CAN). **Hadley B.**: *Edlund 87*, *314* (CAN). **Storkerson P.**: *Edlund 236* (CAN).

### *Micranthes* Haworth [4]

**Key to *Micranthes* [adapted from Healy and Gillespie (2004 [2005]) and Brouillet and Elvander (2009)]**

**Table:** 

1	Inflorescences with all or some flowers replaced with bulbils; basal leaves oblanceolate	***M. foliolosa***
–	Inflorescences without bulbils; basal leaves elliptic, oblong, obovate or ovate	**2**
2	Inflorescences constricted, spikelike thyrses, (2–)3–10 cm; ovary 1/2+ inferior	***M. hieraciifolia***
–	Inflorescences congested, capitate thyrses or ± capitate cymes, 0.5–2(–4) cm; ovary 1/2 inferior, appearing more superior in fruit	**3**
3	Inflorescence one to several dense head-like clusters of numerous flowers; petals white or essentially so (sometimes becoming pink with age); flowering stem (0.5–)1–2.5 mm wide, moderately to densely hairy, with conspicuous long coarse white hairs; plant usually robust in appearance	***M. nivalis***
–	Inflorescence an open cyme of fewer flowers, flowers on distinct pedicels; petals pink or less often white; flowering stem 0.3–1 mm wide, usually sparsely hairy, with short fine hairs that are usually inconspicuous; plant delicate in appearance	***M. tenuis***

***Micranthes foliolosa*** (R.Br.) Gornall (*Saxifraga foliolosa* R.Br.), Fig. 35C–Leafy-stemmed saxifrage | Circumpolar

Previously recorded from Hadley B., Storkerson P. and Ulukhaktok (Porsild 1955, 1957, 1964, Aiken et al. 2007). Elsewhere in the Canadian Arctic widespread across the Queen Elizabeth islands, Baffin, Banks, Coats, King William and Southampton islands, and across the mainland (Porsild and Cody 1980, Cody et al. 1989, Cody et al. 2003, Aiken et al. 2007, Saarela et al. 2013, Blondeau 2015f, Saarela et al. 2017b).

**NORTHWEST TERRITORIES. Ulukhaktok**: *Porsild 17307* (ALTA, CAN). **NUNAVUT. Hadley B.**: *Edlund 38*, *342* (CAN). **Storkerson P.**: *Edlund 289* (CAN).

***Micranthes hieraciifolia*** (Waldst. *&* Kit. ex Willd.) Haw. (*Saxifraga hieraciifolia* Waldst. & Kit. ex Willd.), Fig. 35D–Hawkweed-leaved saxifrage | Circumpolar-alpine

Previously recorded from Cambridge Bay (Porsild and Cody 1980, Aiken et al. 2007). Newly recorded from “30-Mile Cr.”, Ferguson L. and Greiner L. Elsewhere in the Canadian Arctic recorded from Axel Heiberg, Baffin, Devon, Ellesmere, King William, Prince Charles and Somerset islands and scattered sites on the mainland (Porsild and Cody 1980, Aiken et al. 2007).

**NUNAVUT. “30-Mile Cr.**”: *Bennett et al. 14-0350* (CAN, UBC). **Cambridge Bay**: *Bennett 13-0230* (BABY, CAN, chars), *Oldenburg 44-955* (CAN), *Polunin s.n.* (CAN), *Stephens 1258*, *1287* (CAN). **Ferguson L. [Tahiryuaq**]: *Bennett et al. 14-0422* (BABY, CAN). **Greiner L.**: *Ponomarenko VI-030a* (CAN).

***Micranthes nivalis*** (L.) Small (*Saxifraga nivalis* L.), Figs 35E, 36B–Snow saxifrage | Circumpolar–alpine

Previously recorded from Cambridge Bay, C. Colborne, Collinson P., Ulukhaktok, the head of Minto Inl., Hadley B., Natkusiak P., Storkerson P. and Richard Collinson Inl. (Porsild 1955, 1957, 1964, Porsild and Cody 1980, Healy and Gillespie 2004, Aiken et al. 2007). Thannheiser et al. (2001) additionally recorded it from Johansen B. (conf.), Richardson I. and Surrey L. Newly recorded from “30-Mile Cr.”, Ferguson L., Greiner L., Kuujjua R., an inland site on Prince Albert P., the north side of Prince Albert S., Sinclair Cr. and Washburn L. Widespread across the Canadian Arctic islands and mainland (Porsild and Cody 1980, Cody et al. 1989, Cody et al. 2003, Aiken et al. 2007, Saarela et al. 2013, Blondeau 2015f, Saarela et al. 2017b).

**NORTHWEST TERRITORIES. Kuujjua R.**: *Gillespie et al. 9845*, *9995* (CAN). **Minto Inl. (head)**: *Edlund 594* (CAN), *Gillespie et al. 10054* (CAN). **Natkusiak P.**: *Edlund 104* (CAN). **Prince Albert P.**: *Oldenburg 54*-*688* (GH, UBC). **Prince Albert S. (N)**: *Oldenburg 46-2281* (CAN). **Richard Collinson Inl.**: *Edlund 544* (CAN). **Ulukhaktok**: *Edlund 719* (CAN), *Porsild 17309* (CAN). **NUNAVUT. “30-Mile Cr.**”: *Bennett et al. 14-0346* (BABY). **Cambridge Bay**: *Bennett et al. 13-0308* (BABY, CAN), *Oldenburg 44*-*933* (CAN, GH), *Polunin s.n.* (CAN), *Stephens 1276*, *975* (CAN). **C. Colborne**: *Edlund & Argus 12730* (CAN). **Collinson P.**: *Edlund & Argus 12762* (CAN). **Ferguson L. [Tahiryuaq**]: *Bennett et al. 14-0424* (ALA, BABY). **Greiner L.**: *Ponomarenko VI-272* (CAN). **Hadley B.**: *Edlund 93*, *s.n.* (CAN). **Johansen B.**: *Gillespie et al. 7828*, *7975* (CAN), *8142* (CAN, O). **Natkusiak P.**: *Edlund 84* (CAN). **Prince Albert S. (head)**: *Edlund 76* (CAN). **Sinclair Cr.**: *Gillespie et al. 8277* (CAN). **Storkerson P.**: *Edlund 198* (CAN). **Washburn L.**: *Oldenburg 46-2178* (CAN).

***Micranthes tenuis*** (Wahlenb.) Small, Fig. 35F–Slender saxifrage | Circumpolar

Previously recorded from Cambridge Bay and Ulukhaktok (Healy and Gillespie 2004, Aiken et al. 2007); we have not seen a voucher for the former record. Newly recorded from Kuujjua R. and the head of Minto Inl. Elsewhere in the Canadian Arctic recorded from Axel Heiberg, Baffin, Bathurst, Cornwallis, Devon, Ellef Ringnes, Ellesmere, Lougheed, Melville, Prince Patrick, Southampton and Somerset islands and northern Quebec and Labrador (Porsild and Cody 1980, Aiken et al. 2007).

**NORTHWEST TERRITORIES. Kuujjua R.**: *Gillespie et al. 9896*, *9951* (CAN). **Minto Inl. (head)**: *Gillespie et al. 10163*, *10286* (CAN). **Ulukhaktok**: *Edlund 719*, *875* (CAN).

### *Saxifraga* L. [9]

**Key to *Saxifraga* [adapted from Porsild and Cody (1980) and Brouillet and Elvander (2009)]**

**Table:** 

1	Leaves opposite, imbricated; plants low, mat-forming and trailing; flowers pink-purple	***S. oppositifolia***
–	Leaves alternate; plants tufted, cespitose or sometimes mat-forming; flowers white, purplish-white or yellow	**2**
2	Basal leaves entire; petals yellow	**3**
–	Basal leaves toothed or lobed; petals white or purplish-white	**5**
3	Plants stoloniferous, reddish; inflorescences with purplish-tipped stipitate glands; petals not spotted	**S. flagellaris subsp. platysepala**
3	Plants not stoloniferous (or rarely with very short stolons), green; inflorescences glabrous or clear-tipped stipitate-glandular or reddish-brown villous; petals often with orange spots	**4**
4	Basal leaves linear to narrowly oblong, succulent, petioles absent; plants matted or cushion forming; leaf blade margins usually spinose-ciliate; ovary ½ inferior	***S. aizoides***
–	Basal leaves oblanceolate, thin or slightly fleshy, petioles present; plants loosely tufted; leaf blade margins eciliate or sparsely reddish brown-ciliate; ovary superior	***S. hirculus***
5	Inflorescences with most flowers replaced by red bulbils, sometimes with one large white flower borne on the tip of the flowering stem	***S. cernua***
–	Inflorescences lacking bulbils	**6**
6	Basal leaves distinctly petiolate, blades orbicular or reniform with 3–5 lobes	**7**
–	Basal leaves not petiolate, blades narrowly cuneate or cuneate-flabellate, sharply 3-toothed or 3–5-lobed	**8**
7	Underground stolons present; flowering stems usually about same length as leaves, not or only somewhat exserted above leaves; basal leaf blades mostly 5-lobed; petals white	**S. rivularis subsp. arctolitoralis**
–	Underground stolons absent; flowering stems usually much longer than and much exserted above leaves; basal leaf blades mostly 3–5-lobed; petals white to pale purplish, usually with purplish veins	***S. hyperborea***
8	Basal leaf blades narrowly cuneate, sharply 3-toothed (rarely with a single apical tooth); petals white with yellow to dark orange spots	***S. tricuspidata***
–	Basal leaf blades cuneate-flabellate, with 3–5 soft, rounded lobes; petals white without spots	***S. cespitosa***

***Saxifraga aizoides*** L., Figs 35G, 36C–Yellow mountain saxifrage | North American (N)–amphi-Atlantic–European

Previously recorded from Byron B., Cambridge Bay, Mt. Bumpus, Ulukhaktok, Richard Collinson Inl. and Wollaston P. (Porsild obs.) (Porsild 1955, 1957, 1964, Porsild and Cody 1980, Aiken et al. 2007). Thannheiser et al. (2001) additionally recorded it from the head of Minto Inl. (conf.), Johansen B. (conf.), Mt. Pelly (conf.) and Surrey L. Newly recorded from Boot Inl., C. Baring, C. Wollaston, Falaise Bay, Greiner L., Kuujjua R., Oterkvik Pt., Sinclair Cr., “Trunsky L.” and Walker B. Elsewhere in the Canadian Arctic recorded from scattered sites on Baffin, Banks, Coats, Ellesmere, Prince Patrick, Resolution and Southampton islands, and across the mainland (Porsild and Cody 1980, Korol 1992, Aiken et al. 2007, Saarela et al. 2013, Blondeau 2015f, Saarela et al. 2017b).

**NORTHWEST TERRITORIES. Boot Inl.**: *Gillespie et al. 9522* (CAN, O). **C. Baring**: *Edlund 411* (CAN). **C. Wollaston**: *Edlund 36* (CAN). **Kuujjua R.**: *Gillespie et al. 9824* (ALA, CAN, O). **Minto Inl. (head)**: *Gillespie et al. 10099* (ALA, ari, CAN, MT, O, WIN), *10262* (ALA, CAN, MT). **Richard Collinson Inl.**: *Edlund 178* (CAN), *Stretton 194* (DAO). **Ulukhaktok**: *Edlund 450* (CAN), *Saarela & Bull 1497* (CAN). **Walker B.**: *Oldenburg 45-1528A* (CAN, GH). **NUNAVUT. Byron B.**: *Dushenko 26* (UVIC). **Cambridge Bay**: *Bennett et al. 13-0534* (chars, UBC), *Stephens 1170* (CAN). **Falaise B.**: *Eriksen et al. 938* (ALA). **Greiner L.**: *Ponomarenko VI-288* (CAN). **Johansen B.**: *Gillespie et al. 8084* (CAN). **Mt. Bumpus**: *Edlund 247* (CAN). **Ovayok TP**: *Gillespie et al. 8433* (ALA, BABY, CAN, MT, O, UBC), *Gould s.n.* (ALA). **Oterkvik Pt.**: *Gillespie et al. 7590* (ALA, CAN, O). **Sinclair Cr.**: *Gillespie et al. 8324* (CAN, O). “**Trunsky L.**”: *Bennett et al. 14-0399* (CAN).

***Saxifraga cernua*** L., Fig. 35H–Nodding saxifrage | Circumpolar–alpine

Previously recorded from Byron B., Cambridge Bay, Hadley B., Mt. Bumpus, Mt. Lady Pelly, the head of Prince Albert S. (Porsild obs.), Richard Collinson Inl., Wollaston P. (Porsild obs.) and Ulukhaktok. Thannheiser et al. (2001) additionally recorded it from Johansen B. (conf.), Mt. Pelly, Richardson I. and Wellington B. Newly recorded from Boot Inl., Kuujjua R., Natkusiak P., Prince Albert P., the north side of Prince Albert S., Tahiryuaq, Ferguson L., Greiner L., Murray Pt., Oterkvik Pt., Read I., Sinclair Cr., Storkerson P. and Washburn L. Widespread across the Canadian Arctic islands and mainland (Porsild and Cody 1980, Cody et al. 1989, Korol 1992, Cody et al. 2003, Aiken et al. 2007, Saarela et al. 2013, Blondeau 2015f, Saarela et al. 2017b).

**NORTHWEST TERRITORIES. Boot Inl.**: *Gillespie et al. 9565* (CAN). **Kuujjua R.**: *Gillespie et al. 9812* (CAN). **Minto Inl. (head)**: *Edlund 110*, *56*, 592 (CAN), **Minto Inl. (head)**: *Gillespie et al. 10045* (CAN), *10179* (ALA, CAN, MT, O). **Natkusiak P.**: *Edlund 121*, *82* (CAN). “**Oldenburg L.**”: *Oldenburg 45-1347* (CAN. GH). **Prince Albert P.**: *Oldenburg 54-693* (GH). **Prince Albert S. (N)**: *Oldenburg 46-2283* (CAN). **Richard Collinson Inl.**: *Stretton 194* (DAO). **Tahiryuaq**: *Edlund 158* (CAN). **Ulukhaktok**: *Edlund 504*, *792* (CAN), *Oldenburg 42-7*, *45-1656*, *45-1657* (CAN), *Ross 14* (ALTA), *14A*GH). **NUNAVUT. Byron B.**: *Dushenko 26* (UVIC). **Cambridge Bay**: *Bennett 13-0244* (chars, od), *Bennett et al. 14-0316* (CAN, UBC), *Calder et al. 24163* (DAO), *Dutilly 28057* (DAO), *Edlund & Argus 12646*, *12666* (CAN), *Gillespie et al. 8368* (CAN), *Oldenburg 44*-*929A* (CAN, GH), *Parker & Jonsdottir 9088* (ALA), *Polunin s.n.* (CAN, 2 sheets), *Ponomarenko VI-307B* (CAN), *Stephens 970*, *1026* (CAN), *Sweatman & Smith 30* (CAN, DAO). **Ferguson L. [Tahiryuaq**]: *Jones* 17 (DAO). **Greiner L.**: *Ponomarenko VI-048*, *VI-139*, *VI-140A*, *VI-155*, *VI-203H*, *VI-212* (CAN). **Hadley B.**: *Edlund 136*, *20*, *94* (CAN). **Johansen B.**: *Gillespie et al. 8032* (CAN). **Mt. Bumpus**: *Edlund 238* (CAN). **Mt. Lady Pelly [Amaaqtuq**]: *Jones 13a* (DAO). **Murray Pt.**: *Gillespie et al. 8177* (CAN, O). **Natkusiak P.**: *Edlund 105* (CAN). **Oterkvik Pt.**: *Gillespie et al. 7539* (ALA, ALTA, BABY, CAN, MT, O, UBC). **Sinclair Cr.**: *Gillespie et al. 8284* (CAN, O). **Read I.**: *Ross 16A* (GH). **Storkerson P.**: *Edlund 197* (CAN). **Washburn L.**: *Oldenburg 46-2175* (CAN).

***Saxifraga cespitosa*** L., Fig. 35I–Tufted saxifrage | Circumpolar–alpine

Previously recorded from Cambridge Bay, C. Wollaston, Hadley B., Natkusiak P., the head of Prince Albert S. and a site southeast thereof, Storkerson P. and Ulukhaktok (Porsild 1955, 1957, 1964, Porsild and Cody 1980, Aiken et al. 2007). Thannheiser et al. (2001) additionally recorded it from Surrey L. Newly recorded from “30-Mile Cr.”, Boot Inl., south of Burns L., Greiner L., Kuujjua R., Murray Pt., “Oldenburg L.”, Oterkvik Pt., Prince Albert P., Read I. and Sinclair Cr. Widespread across the Canadian Arctic islands, and across the mainland (Porsild and Cody 1980, Aiken et al. 2007, Saarela et al. 2013, Blondeau 2015f).

**NORTHWEST TERRITORIES. Boot Inl.**: *Gillespie et al. 9696* (CAN, O). **Burns L. (S)**: *Edlund 45* (CAN). **C. Wollaston**: *Edlund 12*, *120* (CAN). **Kuujjua R.**: *Gillespie et al. 9866* (CAN), *Stretton 51* (DAO). “**Oldenburg L.**”: *Oldenburg 45-1346A* (CAN, GH). **Minto Inl. (head)**: *Edlund 111* (CAN), *Gillespie et al. 9841* (CAN), *10053* (CAN, MT), *10160* (CAN), *10220* (CAN, O). **Prince Albert P.**: *Oldenburg 54-690* (GH). **Prince Albert S. (head)**: *Porsild 17444* (CAN). **Ulukhaktok**: *Edlund 876* (CAN), *Oldenburg 45-1653* (CAN, GH). *Porsild 17308* (CAN). **NUNAVUT. “30-Mile Cr.**”: *Bennett et al. 14-0338* (BABY). **Cambridge Bay**: *Bennett et al. 13-0217* (BABY, chars, UBC, UVIC), *Edlund & Argus 12645*, *12665* (CAN), *Gillespie et al. 8450* (CAN, O), *Oldenburg 44*-*934* (CAN, GH), *Polunin s.n.* (CAN, 2 sheets), *Porsild 21633* (CAN), *Stephens 1009*, *1260*, *1275* (CAN). **Greiner L.**: *Ponomarenko VI-030* (CAN). **Hadley B.**: *Edlund 135*, *63* (CAN). **Murray Pt.**: *Gillespie et al. 8176* (ALA, CAN, MT, O). **Natkusiak P.**: *Edlund 81* (CAN). **Oterkvik Pt.**: *Gillespie et al. 7626* (CAN, O), *7711* (ALA, CAN, MT, O). **Prince Albert S. (head)**: *Edlund 75* (CAN). **Read I.**: *Oldenburg 43-1074* (CAN, GH), *43-911* (CAN), *Porsild 17201* (CAN), *Ross 17A* (GH). **Sinclair Cr.**: *Gillespie et al. 8252* (ALA, CAN, O). **Storkerson P.**: *Edlund 160* (CAN).

***Saxifraga flagellaris*** subsp. ***platysepala*** (Trautv.) A.E.Porsild, Fig. 35J–Broad-sepal saxifrage | Circumpolar

Previously recorded from “Jackpot L.”, Natkusiak P. and Storkerson P (Porsild 1955, 1957, 1964, Aiken et al. 2007). Newly recorded from “Oldenburg L.” This is a high arctic taxon otherwise known from Banks, Prince of Wales, Southampton and the Queen Elizabeth islands (Aiken et al. 2007). The Victoria I. populations mark the species’ southern limit.

**NORTHWEST TERRITORIES. Jackpot L.**: *Porsild 17504* (CAN). **Natkusiak P.**: *Edlund 107* (CAN). “**Oldenburg L.**”: *Oldenburg 45-1347A* (CAN). **Storkerson P.**: *Edlund 220* (CAN), *P. Jenness 34* (CAN).

***Saxifraga hirculus*** L., Figs 35K, 36D–Yellow marsh saxifrage | Circumboreal-polar

Previously recorded from Cambridge Bay, Hadley B., Namaycush L., the head of Minto Inl. (Porsild obs.), the head of Prince Albert S., Richard Collinson Inl., Read I. (Porsild obs.), Storkerson P., Tahoe L. (Porsild obs.) and Ulukhaktok. Thannheiser et al. (2001) additionally recorded it from Johansen B. (conf.), Richardson I. and Surrey L. Newly recorded from Albert Edward B., Boot Inl., Greiner L., Kuujjua R., “Oldenburg L.”, Oterkvik Pt., Sinclair Cr., the south coast of Victoria I. north of Read I., Wollaston P.and two inland sites on Prince Albert P. Elsewhere in the Canadian Arctic recorded from Axel Heiberg, Baffin, Banks, Bathurst, Bylot, Coats, Cornwallis, Devon, Digges, Ellesmere, Igloolik, Jenny Lind, King William, Melville, Prince Charles, Prince of Wales, Prince Patrick, Somerset and Southampton islands, and across the mainland (Porsild and Cody 1980, Cody et al. 1989, Korol 1992, Cody et al. 2003, Aiken et al. 2007, Saarela et al. 2013, Blondeau 2015f, Saarela et al. 2017b).

**NORTHWEST TERRITORIES. Boot Inl.**: *Gillespie et al. 9515* (CAN, O). **Kuujjua R.**: *Gillespie et al. 9817* (ALA, ari, CAN, MT, O, WIN). **Minto Inl. (head)**: *Edlund 57*, *591* (CAN), *Gillespie et al. 10104* (CAN, O), *10180* (ALA, CAN, MT, O). “**Oldenburg L.**”: *Oldenburg 45-1349* (CAN, GH). **Prince Albert P.**: *Oldenburg Oldenburg 54-256* (GH), *54*-*689* (GH, UBC). **Prince Albert S. (head)**: *Porsild 17445* (CAN). **Richard Collinson Inl.**: *Edlund 698* (CAN), *P. Jenness 21* (CAN). **Ulukhaktok**: *Edlund 300* (CAN), *Oldenburg 42-75b*, *42-99a* (CAN), *42-98*, *45-1655* (CAN, GH), *Ross 20* (ALTA), *20A* (GH). **Walker B.**: *Oldenburg 45-1527A* (CAN, GH). **Wollaston P.**: *Oldenburg 54-513* (GH). **NUNAVUT. Albert Edward B.**: *Ponomarenko VI*-261 (CAN). **Cambridge Bay**: *Bennett et al. 13-0203* (chars, od), *Consaul & Gillespie 1122* (CAN), *Edlund & Argus 12623* (CAN), *Fortier 12* (CAN), *Gillespie et al. 8386* (ALA, CAN, MT, O, UBC), *Oldenburg 44-890* (CAN), *Polunin s.n.* (CAN), *Porsild 21634* (CAN), *Stephens 948*, *1194* (CAN). **Greiner L.**: *Ponomarenko VI-124*, *VI-132*, *VI-140C*, *VI-184a*, *VI-203G* (CAN). **Hadley B.**: *Edlund 46*, *125* (CAN). **Johansen B.**: *Gillespie et al. 7994* (CAN). **Ovayok TP**: *Gould s.n.* (ALA). **Namaycush L.**: *Edlund 125* (CAN). **Oterkvik Pt.**: *Gillespie et al. 7637*, *7678* (ALA, CAN, O). **Sinclair Cr.**: *Gillespie et al. 8234* (ALA, CAN, O, UBC). **S coast of Victoria I. N of Read I.**: *Ross 27* (ALTA), *27A* (GH). **Storkerson P.**: *Edlund 163*, *239*, *304* (CAN).

***Saxifraga hyperborea*** R.Br., Fig. 35L–Pygmy saxifrage | Circumpolar–alpine

Previously recorded from Cambridge Bay, “Jackpot L.”, Storkerson P. and Ulukhaktok (Porsild 1955, 1957, 1964, Porsild and Cody 1980, Aiken et al. 2007). Newly recorded from Kuujjua R., the head of Minto Inl., Murray Pt., Prince Albert P. and Washburn L. Elsewhere in the Canadian Arctic recorded from Baffin, Banks, Prince of Wales, Somerset and most of the Queen Elizabeth islands and scattered mainland sites (Aiken et al. 2007, Saarela et al. 2013, Blondeau 2015f, Saarela et al. 2017b).

**NORTHWEST TERRITORIES. Jackpot L.**: *Porsild 17505* (CAN). **Kuujjua R.**: *Gillespie et al. 10000*, *9894*, *9954* (CAN). **Minto Inl. (head)**: *Gillespie et al. 10308* (CAN), *Porsild 17403* (CAN). **Prince Albert P.**: *Oldenburg 54-254* (GH). **Ulukhaktok**: *Edlund 796* (CAN). **NUNAVUT. Cambridge Bay**: *Edlund & Argus 12882* (CAN), *Porsild 21635* (CAN), *Stephens 1182* (CAN). **Murray Pt.**: *Gillespie et al. 8175* (CAN). **Storkerson P.**: *Edlund 161*, *237* (CAN). **Washburn L.**: *Oldenburg 46-2176* (CAN, GH, MIN)

**Figure 37.**
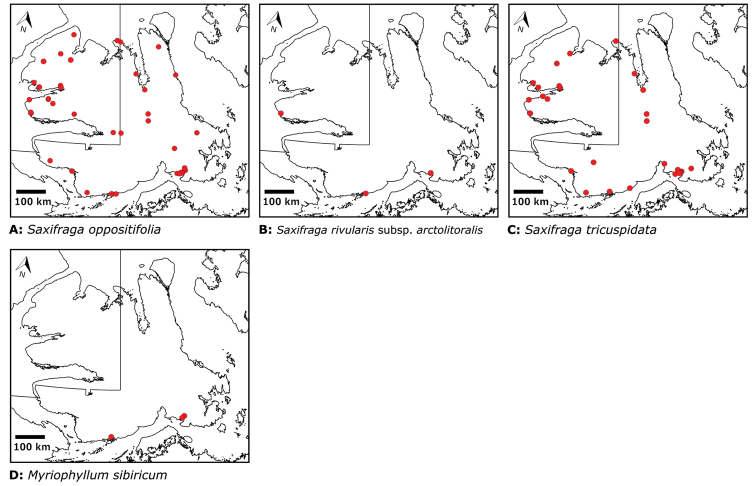
Species distribution maps. Saxifragaceae: **A***Saxifraga oppositifolia***B**Saxifraga rivularis subsp. arctolitoralis**C***Saxifraga tricuspidata*. Haloragaceae: **D***Myriophyllum sibiricum*.

***Saxifraga oppositifolia*** L., Figs 37A, 36E–Purple saxifrage | Circumpolar–alpine

Previously recorded from Cambridge Bay, Greely Haven, Natkusiak P., the head of Minto Inl. (Porsild obs., conf.), the head of Prince Albert S., Read I., Ulukhaktok and Wollaston P. (Porsild 1955, 1957, 1964, Porsild and Cody 1980, Aiken et al. 2007). Thannheiser et al. (2001) additionally recorded it from Johansen B. (conf.), Mt. Pelly, Surrey L. and Wellington B. Newly recorded from Boot Inl., C. Wollaston, Greiner L., Murray Pt., Namaycush L., “Oldenburg L.”, Oterkvik Pt., Prince Albert P., the north side of Prince Albert S., Richard Collinson Inl., Storkerson P., “Trunsky L.”, Walker B., Washburn L. and a site on eastern Victoria I. Widespread across the Canadian Arctic islands and mainland (Porsild and Cody 1980, Cody et al. 1989, Korol 1992, Aiken et al. 2007, Saarela et al. 2013, Blondeau 2015f, Saarela et al. 2017b).

**NORTHWEST TERRITORIES. Boot Inl.**: *Gillespie et al. 9584* (CAN), *Gillespie et al. 9682* (ALA, CAN, MT, WIN). **C. Wollaston**: *Edlund 13* (CAN). **Kuujjua R.**: *Gillespie et al. 9763* (ari, CAN, O), *9813* (ALA, CAN, O). **Minto Inl. (head)**: *Edlund 58* (CAN), *Gillespie et al. 10173* (CAN), *9482* (CAN, O). **Natkusiak P.**: *Edlund* 99, *100*, *101* (CAN). “**Oldenburg L.**”: *Oldenburg 45-1348* (CAN). **Prince Albert P.**: *Oldenburg 54-255, 54-692* (GH). **Prince Albert S. (head)**: *Stretton 1* (DAO), *Weerstra 31* (DAO). **Prince Albert S. (N)**: *Oldenburg 46-2282* (CAN). **Richard Collinson Inl.**: *Edlund 156*, *188* (CAN). **Ulukhaktok**: *Bandringa 315* (CAN), *Edlund 293* (CAN), *Gray & Gibbard 1*, *8* (DAO), *Oldenburg 45-1649* (CAN, GH), *Ross 10A* (GH), *Saarela & Bull 1491* (CAN). **Walker B.**: *Oldenburg 45-1526A* (CAN). **NUNAVUT. Cambridge Bay**: *Bennett et al. 13-0198* (BABY, chars), *13-0566* (UBC), *Calder et al. 24205* (DAO), *Edlund & Argus 12878* (CAN), *Fortier 16* (CAN), *Gillespie et al. 8490* (CAN, O), *Gould s.n.* (ALA), *Oldenburg 44*-*926* (CAN), *Smith 1* (CAN), *Stephens 833*, *1192* (CAN), *Washburn 40* (GH), *42* (CAN). **E. Victoria I.**: *Lee & Kittle s.n.* (CAN). **Greely Haven**: *Fortier 96* (CAN). **Greiner L.**: *Ponomarenko VI-221a*, *VI-224C* (CAN). **Hadley B.**: *Edlund 10*, *85* (CAN). **Johansen B.**: *Gillespie et al. 8025* (CAN, O, UBC). **Murray Pt.**: *Gillespie et al. 8213* (CAN, O). **Namaycush L.**: *Edlund 3*, *s.n.* (CAN). **Natkusiak P.**: *Edlund 83* (CAN). **Oterkvik Pt.**: *Gillespie et al. 7494* (ALA, CAN, O). **Read I.**: *Oldenburg 42-518B*, *43-1037*, *43-1065* (CAN), *Porsild 17202* (CAN), *Ross 22A* (GH). **Storkerson P.**: *Edlund 162* (CAN). “**Trunsky L.**”: *Bennett et al. 14-0387* (BABY). **Wollaston P.**: *D. Jenness 390* (CAN). **Washburn L.**: *Oldenburg 46-2174* (CAN).

***Saxifraga rivularis*** subsp. ***arctolitoralis*** (Jurtzev & V.V.Petrovsky) M.H.Jørg. & Elven, Fig. 37B–Alpine seashore saxifrage | Amphi-Beringian–North American

First reported for Victoria I. by Gillespie et al. (2015) based on our collection from Murray Pt. New records from Cambridge Bay and Ulukhaktok are reported here. This subspecies differs from the eastern Arctic subsp. rivularis by having longer glandular hairs on the hypanthium (3–6 mm versus 1–2 mm in the latter). The collection from Cambridge Bay appears somewhat intermediate between the two subspecies with hypanthium hairs mostly sparse and 1–2 mm long, but with some longer to 4 mm. Subspecies arctolitoralis approaches *S. hyperborea* and, apart from the presence of stolons in the former (which may not always be obvious on specimens), the two may be difficult to distinguish. Additional field collections with attention to presence of stolons are needed, as well as further study to better distinguish/resolve taxa in this species complex in the western Arctic islands. Subspecies were not recognized in previous Canadian Arctic floras relevant to the study area.

**NORTHWEST TERRITORIES. Ulukhaktok**: *Oldenburg 45-1650* (CAN), *Porsild 17310* (CAN). **NUNAVUT. Cambridge Bay**: *Oldenburg 44-939* (CAN). **Murray Pt.**: *Gillespie et al. 8174* (ALA, ALTA, ari, BABY, CAN, MT, O, UBC, V).

***Saxifraga tricuspidata*** Rottb., Figs 37C, 36F–Prickly saxifrage | North American (N)

Previously recorded from Byron B., Cambridge Bay, the head of Minto Inl., Mt. Bumpus, Namaycush L., Natkusiak P., Richard Collinson Inl. and Ulukhaktok (Porsild 1955, 1957, 1964, Porsild and Cody 1980, Aiken et al. 2007). Thannheiser et al. (2001) additionally recorded it from Johansen B. (conf.), Hadley B. (conf.), Mt. Pelly, Richardson I., Surrey L. and Wellington B. Newly recorded from Boot Inl., C. Wollaston, Ferguson L., Greiner L., Kuujjua R., Oterkvik Pt., an inland site on Prince Albert P., Read I., Sinclair Cr. and Walker B. Elsewhere in the Canadian Arctic recorded from Axel Heiberg, Banks, Baffin, Coats, Devon, Ellesmere, King William, Somerset and Southampton islands, and across the mainland (Porsild and Cody 1980, Cody et al. 1989, Korol 1992, Aiken et al. 2007, Saarela et al. 2013, Blondeau 2015f, Saarela et al. 2017b).

**NORTHWEST TERRITORIES. Boot Inl.**: *Gillespie et al. 9537* (ALA, ari, CAN, MT, O, UBC, WIN). **C. Wollaston**: *Edlund 11* (CAN). **Kuujjua R.**: *Dutilly 18773* (DAO), *Gillespie et al.* 9735 (ALA, CAN, MT, UBC). **Minto Inl. (head)**: *Edlund 45*, *592*, *593* (CANJ), *Gillespie et al. 10044* (ALA, ari, CAN, MT, O, WIN). **Natkusiak P.**: *Edlund 80* (CAN). **Prince Albert P.**: *Oldenburg 54*-691 (UBC). **Richard Collinson Inl.**: *P. Jenness 15* (CAN). **Ulukhaktok**: *Edlund 345* (CAN), *Ross 17* (ALTA), *17A* (GH), *Oldenburg 42-11* (CAN, GH), *45-1651* (CAN), *54-216* (GH), *Saarela & Bull 1436* (ALA, CAN, MT, O). **Walker B.**: *Oldenburg 45-1529A* (CAN, GH). **NUNAVUT. Cambridge Bay**: *Bennett et al. 13-0162* (chars, UBC), *Calder et al. 24162* (DAO), *Edlund & Argus 12664* (CAN), *Fortier 19* (CAN), *Gillespie et al. 8395* (ALA, ALTA, BABY, CAN, MT, O, UBC), *Oldenburg 44*-*946* (CAN), *Polunin s.n.* (CAN), *Ponomarenko VI-078A* (CAN), *Stephens 954*, *1193* (CAN), *Washburn 32* (GH). **Ferguson L. [Tahiryuaq**]: *Jones 15* (DAO). **Greiner L.**: *Ponomarenko VI-157*, *VI-274A* (CAN). **Hadley B.**: *Edlund 10*, *92* (CAN). **Johansen B.**: *Gillespie et al. 7868* (ALA, ALTA, BABY, CAN, MT, O, UBC, US). **Mt. Bumpus**: *Edlund 230* (CAN). **Namaycush L.**: *Edlund & Roncato-Spencer 114* (CAN), *Edlund 166* (CAN). **Oterkvik Pt.**: *Gillespie et al. 7529* (ALA, CAN, MT, O, UBC). **Read I.**: *Oldenburg 42-502*, *43-1080*, *43*-907, *43-950* (CAN). **Sinclair Cr.**: *Gillespie et al. 8330* (ALA, CAN, MT, O). **Washburn L.**: *Oldenburg 46-2177* (CAN).

### Haloragaceae [1/1]

***Myriophyllum* [1]**

***Myriophyllum sibiricum*** Kom. (*M. exalbescens* Fernald), Figs 37D, 36G–Northern water milfoil | Circumboreal–polar

Previously recorded from Cambridge Bay (Aiken et al. 2007) and newly recorded from Johansen B., where found floating along the edge of a small lake. The sites in the Cambridge Bay area are aquatic habitats (creek, lake edge) along the road to Mt. Pelly. Elsewhere in the Canadian Arctic recorded from Baffin, Belcher and Southampton islands and scattered sites across the mainland (Porsild and Cody 1980, Aiken et al. 2007, Saarela et al. 2013, Garneau 2015, Saarela et al. 2017b). The Victoria I. populations mark the northern edge of the species’ range.

**NORTHWEST TERRITORIES. Cambridge Bay**: *Bennett et al. 13-0291* (BABY, chars, UBC), *Gillespie et al. 8442* (CAN), *Stephens 1280* (CAN, KSTC). **Johansen B.**: *Gillespie et al. 7912* (ALA, CAN, MT, O).

### Core eudicots

**Rosids**

**Fabales**

**Fabaceae [4/10]**

**Key to Fabaceae [adapted from Porsild and Cody (1980) and Blondeau (2015b)]:**

**Table:** 

1	Leaves palmately compound; legume dehisced valves strongly twisted	*** Lupinus ***
–	Leaves pinnately compound; legumes indehiscent and segmented or with dehisced valves straight	**2**
2	Legumes flat, segmented, separating into distinct segments, indehiscent; keel petal much longer than wing petals	*** Hedysarum ***
–	Legumes sub-terete, not segmented, dehiscent; keel petal as long or only slightly longer than wing petals	**3**
3	Keel of the corolla blunt and without an appendage; legumes appearing 2-locular; plants cespitose, matted or trailing, with inflorescences borne on leafy stems	*** Astragalus ***
–	Keel of the corolla tipped with an erect point; legumes 1-locular; plants cespitose, usually densely so, with inflorescences borne on naked stems	*** Oxytropis ***

### *Astragalus* L. [2]

**Key to *Astragalus* [adapted from Porsild and Cody (1980) and Aiken et al. (2007)]**

**Table:** 

1	Legumes pubescent, not inflated, long-stipitate, yellow-green to brown at maturity; stems thin, prostrate to ascending	***A. alpinus***
1	Legumes glabrous, inflated, short-stipitate, red at maturity; stems stout, erect-ascending	***A. richardsonii***

**Figure 38.**
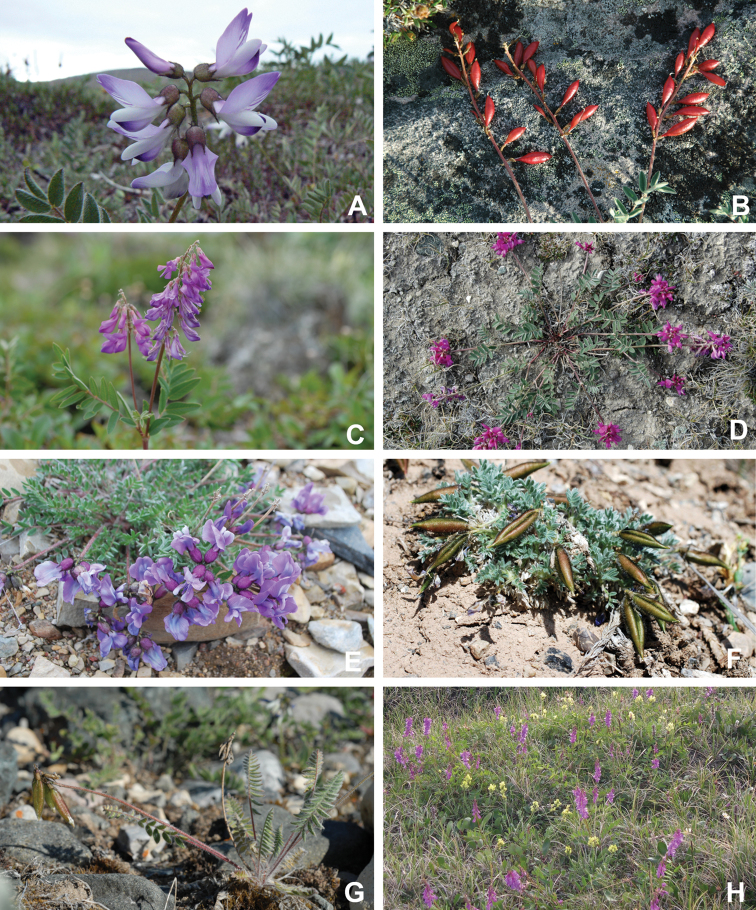
**A***Astragalus alpinus* inflorescence, Ulukhaktok, NT, 8 July 2010 **B***Astragalus richardsonii* infructescences, Johansen Bay, NU, 14 July 2008 **C***Hedysarum americanum* inflorescence, Johansen Bay, NU, 18 July 2008 **D**Hedysarum boreale subsp. mackenziei habit, Kuujjua River, NT, 16 July 2010 **E**Oxytropis arctica var. arctica habit **F***Oxytropis arctobia* habit, *Gillespie et al. 9593***G**Oxytropis deflexa var. foliolosa habit, *Gillespie et al. 10129***H***Oxytropis maydelliana* habitat, Johansen Bay, NU, 15 July 2008. Photos **A** by P.C. Sokoloff **B**, **D** by R.D. Bull **C**, **G**, **H** by L.J. Gillespie **E** by B.A. Bennett and **F** by J.M. Saarela.

**Figure 39.**
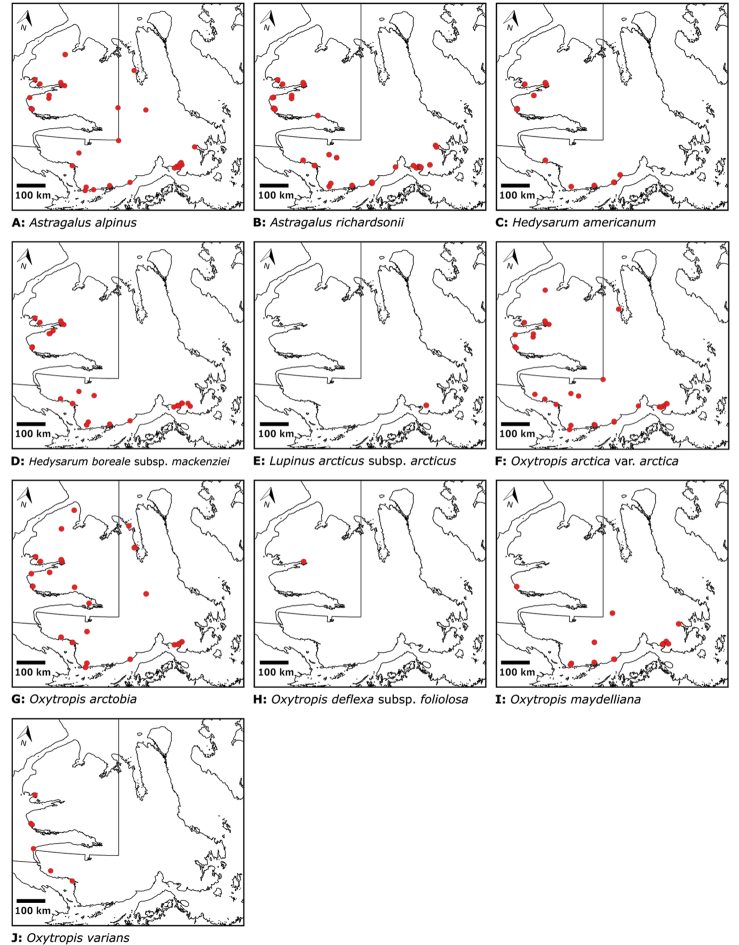
Species distribution maps. Fabaceae: **A***Astragalus alpinus***B***Astragalus richardsonii***C***Hedysarum americanum***D**Hedysarum boreale subsp. mackenziei**E**Lupinus arcticus subsp. arcticus**F**Oxytropis arctica var. arctica**G***Oxytropis arctobia***H**Oxytropis deflexa var. foliolosa**I***Oxytropis maydelliana***J***Oxytropis varians*.

***Astragalus alpinus*** L., Figs 39A, 38A–Alpine milk-vetch | Circumpolar–alpine

Previously recorded from Byron B., Cambridge Bay, Ferguson L., Kuujjua R., the head of Minto Inl., the head of Prince Albert S. (Porsild obs.), Read I. (Porsild obs., conf.), Ulukhaktok and Wollaston P. (Macoun and Holm 1921, Porsild 1955, 1957, 1964, Porsild and Cody 1980, Aiken et al. 2007). Newly recorded from Albert Edward B., Boot Inl., C. Wollaston, Greiner L., Oterkvik Pt., Prince Albert P., southeast of the head of Prince Albert S., east of Tahiryuaq at a site along the territorial border, Walker B. and Washburn L. Elsewhere in the Canadian Arctic recorded from Baffin, Banks, Coats, King William, Melville, Prince Patrick and Southampton islands, and numerous mainland sites (Porsild and Cody 1980, Cody et al. 1989, Korol 1992, Cody et al. 2003, Cody and Reading 2005, Aiken et al. 2007, Saarela et al. 2013, Blondeau 2015b).

**NORTHWEST TERRITORIES. Boot Inl.**: *Gillespie et al. 9510* (ALA, CAN, O). **C. Wollaston**: *Edlund 25*, *59* (CAN). **Kuujjua R.**: *Edlund 648* (CAN), *Gillespie et al. 9740* (ALA, CAN, MT, O). **Minto Inl. (head)**: *Edlund 596* (CAN), *Gillespie et al. 10017a* (ALA, ari, CAN, MT, O, UBC), *Porsild 17409* (CAN). **Tahiryuaq**: *Edlund 153* (CAN, KSTC). **Prince Albert P.**: *Oldenburg 54-676* (GH). **Ulukhaktok**: *Edlund 354*, *458*, *786* (CAN), *Oldenburg 42-105*, *45-1643* (CAN), *42-114*, *45-1645* (CAN, GH), *54-209* (GH), *Saarela & Bull 1431* (ALA, ari, CAN, MT, O, UBC), *Svoboda 745010* (UBC). **Walker B.**: *Oldenburg 45-1497*, *45-1498* (CAN). **NUNAVUT. Albert Edward B.**: *Ponomarenko VI-256* (CAN). **Byron B.**: *Dushenko 48* (UVIC). **Cambridge Bay**: *Bennett et al. 13-0208* (BABY, chars, DAO, UBC), *Edlund & Argus 12662* (CAN), *Gillespie 5848* (CAN), *Gillespie et al. 8398* (ALA, CAN), *Gould s.n.* (ALA), *Oldenburg 44*-*886* (CAN, GH), *Polunin s.n.* (CAN, 2 sheets), *Ponomarenko VI-310* (CAN), *Stephens 1028* (KSTC), *971*, *984*, *1136* (CAN, KSTC). **Greiner L.**: *Ponomarenko VI-083A*, *VI-280* (CAN). **Hadley B.**: *Edlund 28* (CAN). **Johansen B.**: *Gillespie et al. 7921* (ALA, ALTA, BABY, CAN, MT, O, UBC). **Oterkvik Pt.**: *Gillespie et al. 7550*, *7696* (CAN), *7712* (ALA, CAN, O). **Prince Albert S. (SE of head)**: *Edlund 77* (CAN). **Read I.**: *Oldenburg 42-527a*, *43-1057*, *43-972* (CAN), *Ross 37A* (GH). **Washburn L.**: *Oldenburg 46-2156* (CAN). **Wollaston P.**: *D. Jenness 388* (CAN).

***Astragalus richardsonii*** E.Sheld. (*A. australis* (L.) Lam., A. australis var. glabriusculus (Hook.) Isely), Figs 39B, Fig. 38B–Richardson’s milk-vetch | North American (NW)

Previously recorded from Cambridge Bay, Ferguson L., the north side of Prince Albert S., Read I. and Ulukhaktok (Porsild 1955, 1957, 1964, Porsild and Cody 1980, Aiken et al. 2007), though Aiken et al. (2007) mapped only a subset of these records. Thannheiser et al. (2001) additionally recorded it from Johansen B. (conf.), Surrey L. and Wellington B. Newly recorded from “30-Mile Cr.”, Albert Edward B., Boot Inl., C. Wollaston, Colville Mts., Falaise B., Greiner L., Kuujjua R., the head of Minto Inl., Mt. Bumpus, Oterkvik Pt., Read I., Sinclair Cr. and Walker B. Elsewhere in the Canadian Arctic recorded from Banks I. and western mainland sites as far east as Bathurst Inl. (Aiken et al. 2007, Saarela et al. 2013, Saarela et al. 2017b). Southeastern Victoria I. populations mark the eastern limit of its known range.

**NORTHWEST TERRITORIES. Boot Inl.**: *Gillespie et al. 9513* (ALA, CAN, MT, O). **C. Wollaston**: *Edlund 152*, *17*, *58* (CAN). **Kuujjua R.**: *Edlund 649* (CAN), *Gillespie et al. 9714* (ALA, CAN, MO, MT, O). **Minto Inl. (head)**: *Edlund 72* (CAN), *Gillespie et al. 10115* (CAN, O), *9473* (CAN, O). **Prince Albert S. (N)**: *Stretton 54* (DAO). **Ulukhaktok**: *Cooper Jr. s.n.* (CAN), *Edlund 324*, *364* (CAN), *Oldenburg 42-1024*, *45-1636* (CAN), *Ross 28*, *28A, 39A*, *39B* (ALTA, GH), *Saarela & Bull 1420* (ALA, ari, CAN, MT, O), *Salokangas 20* (CAN, UBC), *30* (CAN). **Walker B.**: *Oldenburg 45-1495* (CAN). **NUNAVUT. “30-Mile Cr.**”: *Bennett et al. 14-0333* (BABY, BRY). **Albert Edward B.**: O’Brian *s.n.* (CAN), *Ponomarenko VI-256A* (CAN). **Cambridge Bay**: *Bennett et al. 13-0209* (ALA, BABY, chars, UBC), *Edlund & Argus 12630*, *12682*, *12675* (CAN), *Fortier 21* (CAN), *Gillespie 5847* (CAN), *Gillespie et al. 8414* (CAN, O), *Oldenburg 44*-*881* (CAN, GH), *Polunin s.n.* (CAN, 2 sheets), *Ponomarenko VI-092* (CAN), *Porsild 21637*, *21638* (CAN), *Stephens 1077*, *936* (CAN, KANU, KSTC). **Colville Mts.**: *Gillespie et al. 7768* (CAN). **Falaise B.**: *Eriksen et al. 929* (ALA). **Greiner L.**: *Ponomarenko VI-198*, *VI-204* (CAN). **Johansen B.**: *Gillespie et al. 7870* (ALA, BABY, CAN, MT, O, UBC). **Mt. Bumpus**: *Edlund 187*, *265* (CAN). **Oterkvik Pt.**: *Gillespie et al. 7518* (CAN, O), *7671* (CAN). **Read I.**: *Oldenburg 42-533*, *43-1060* (CAN), *Porsild 17204* (CAN). **Sinclair Cr.**: *Gillespie et al. 8259*, *8321* (ALA, CAN, MT, O).

### *Hedysarum* L. [2]

**Key to *Hedysarum* [adapted from Porsild and Cody (1980) and Blondeau (2015b)]:**

**Table:** 

1	Calyx teeth linear to lanceolate; flowers 5–15 per inflorescence; adaxial surface of leaflets strigose to glabrous, nerves not apparent; legumes puberulent	**H. boreale subsp. mackenziei**
–	Calyx teeth deltoid; flowers 20–50 per inflorescence; adaxial surface of leaflets glabrous, nerves apparent; legumes glabrous	***H. americanum***

***Hedysarum americanum*** (Michx.) Britton (*H. alpinum* L., H. alpinum var. americanum Michx. ex Pursh), Figs 39C, 38C–Alpine sweet-vetch | Amphi-Beringian (E)–North American

Previously recorded from Byron B., the head of Minto Inl. and Ulukhaktok (Porsild 1955, 1957, 1964, Porsild and Cody 1980, Aiken et al. 2007). Thannheiser et al. (2001) additionally recorded it from Johansen B. (conf.), Richardson I. and Surrey L. Newly recorded from Boot Inl., Kuujjua R., Falaise B., Oterkvik Pt. and Sinclair Cr. Elsewhere in the Canadian Arctic recorded from southern Banks I. and the adjacent mainland east to the Hudson Bay coast (Porsild and Cody 1980, Korol 1992, Aiken et al. 2007, Saarela et al. 2013, Saarela et al. 2017b).

**NORTHWEST TERRITORIES. Boot Inl.**: *Gillespie et al. 9512* (ALA, CAN, MT, O). **Kuujjua R.**: *Gillespie et al. 9741* (ALA, ari, CAN, MT, O). **Minto Inl. (head)**: *Edlund 137* (CAN), *Gillespie et al. 9480* (CAN, O). **Ulukhaktok**: *Edlund 307*, *832* (CAN), *Oldenburg 42-110* (CAN, GH), *Pokiak 29* (CAN), *Porsild 17316* (CAN), *Ross 38* (ALTA), *Saarela & Bull 1471* (ALA, ALTA, ari, CAN, MT, O, UBC, US, WIN). **NUNAVUT. Byron B.**: *Dushenko 19* (UVIC), *Edlund & Argus 12850* (CAN). **Falaise B.**: *Eriksen et al. 970* (ALA). **Johansen B.**: *Gillespie et al. 7819* (ALA, CAN, MT, O, UBC). **Oterkvik Pt.**: *Gillespie et al. 7510* (CAN, O). **Sinclair Cr.**: *Gillespie et al. 8257*, *8331* (ALA, CAN, MT, O).

***Hedysarum boreale*** subsp. ***mackenziei*** (Richardson) S.L.Welsh (*H. mackenziei* Richardson), Figs 39D, 38D–Mackenzie’s sweet vetch | Amphi-Beringian (E)–North American

Previously recorded from Byron B., Cambridge Bay, Kuujjua R., Ulukhaktok (Porsild obs., conf.), the head of Minto Inl. (Porsild obs., conf.) and Wollaston P. (Simmons 1913, Macoun and Holm 1921, Porsild 1955, 1957, 1964, Porsild and Cody 1980, Aiken et al. 2007). Thannheiser et al. (2001) additionally recorded it from Johansen B. (conf.), Mt. Pelly, Richardson I. and Surrey L. Newly recorded from Boot Inl., Greiner L., Falaise B., Mt. Bumpus, Oterkvik Pt., Read I. and Walker B. Elsewhere in the Canadian Arctic recorded from Banks and Eglinton islands, the adjacent mainland east to Hudson Bay, and in northern Quebec at a few sites barely extending into the Arctic ecozone (Porsild and Cody 1980, Korol 1992, Cody and Reading 2005, Aiken et al. 2007, Saarela et al. 2013, Blondeau 2015b, Saarela et al. 2017b).

**NORTHWEST TERRITORIES. Boot Inl.**: *Gillespie et al. 9514* (ALA, CAN, MT, O). **Kuujjua R.**: *Gillespie et al. 9713* (ALA, ari, CAN, MT, O, UBC), *Stretton 70*, *76* (DAO). **Minto Inl. (head)**: *Edlund 73* (CAN), *Gillespie et al. 10016* (ALA, ari, CAN, MT, O), *10177* (CAN), *Gillespie et al. 10251* (CAN, O). **Ulukhaktok**: *Bandringa 308* (CAN), *Edlund 308* (CAN), *Oldenburg 42-112* (CAN), *Ross 39a* (ALTA, GH), *Saarela & Bull 1470* (ALA, CAN, O). **Walker B.**: *Oldenburg 45-1494* (CAN). **NUNAVUT. Byron B.**: *Dushenko 18* (UVIC). **Cambridge Bay**: *Bennett et al. 13-0321* (DAO), *Consaul & Gillespie 1112* (CAN), *Edlund & Argus 12631* (CAN), *Gillespie 5828* (CAN), *Gillespie et al. 8393* (ALA, CAN, MT, O, UBC), *8418* (ALA, CAN, MT, O), *Gould s.n.* (ALA), *Ponomarenko VI-064* (CAN), *Scotter s.n.* (ALTA), *Stephens 1004* (CAN), *1134* (CAN, KSTC), *Sweatman & Smith 32* (DAO). **Falaise B.**: *Eriksen et al. 942* (ALA). **Greiner L.**: *Ponomarenko VI-134*, *VI-194* (CAN). **Johansen B.**: *Gillespie et al. 7897* (ALA, ALTA, BABY, CAN, MT, O, UBC, US, V). **Mt. Bumpus**: *Edlund 218*, *261*, *277* (CAN). **Ovayok TP**: *Stephens 859* (KSTC). **Oterkvik Pt.**: *Gillespie et al. 7466*, *7519* (CAN, O), *7669* (ALA, BABY, CAN, MT, O, UBC). **Read I.**: *Ross 39* (ALTA). **Wollaston P.**: *D. Jenness 389* (CAN).

### *Lupinus* L. [1]

***Lupinus arcticus*** S.Watson subsp. ***arcticus***, Fig. 39E–Arctic lupine | North American (NW)

Previously recorded from “Long L.” and the “south coast of Victoria Land” (*Rae s.n.*) (Porsild 1955, 1957, 1964, Porsild and Cody 1980, Aiken et al. 2007). The latter collection is not mapped since the area of collection is ambiguous. Elsewhere in the Canadian Arctic recorded from Ballast Beach on northern Banks I. (K000829387!), where collected by Meirtsching in 1851 (Porsild 1955, 1957, 1964, Porsild and Cody 1980), and mainland sites, primarily from Bathurst Inl. westwards (Porsild and Cody 1980, Cody and Reading 2005, Aiken et al. 2007, Saarela et al. 2013, Saarela et al. 2017b). The northern Banks I. collection is far out of range and occurrence of the species in that area requires confirmation.

**NUNAVUT. “South coast**”: *Rae s.n.* (K). “**Long L.**”: *Lambert s.n.* (CAN, 3 sheets) (Suppl. material 6).

### *Oxytropis* DC. [5]

**Key to *Oxytropis* [adapted from Porsild and Cody (1980) and Aiken et al. (2007)]:**

**Table:** 

1	Flowers and legumes deflexed; stipules free from petiole	**O. deflexa var. foliolosa**
–	Flowers and legumes ascending; stipules adnate to petiole	**2**
2	Stipules chestnut brown; flowers yellow	***O. maydelliana***
–	Stipules white to papery-grey, sometimes slightly brown; flowers yellow or blue-purple	**3**
3	Flowers yellow, 3–12 per inflorescence	***O. varians***
–	Flowers blue-purple, 1–7 per inflorescence	**4**
4	Plants low and densely cespitose, cushion forming; leaves < 3 cm; flowers 1(2) per inflorescence	***O. arctobia***
–	Plants taller and loosely cespitose, not cushion forming; leaves ≥ 3 cm; flowers 2–6 per inflorescence	**O. arctica var. arctica**

***Oxytropis arctica*** R.Br. var. ***arctica***, Figs 39F, 38E–Arctic locoweed | Asian (N) & North American (NW)

Previously recorded from Albert Edward B., Cambridge Bay, C. Wollaston, Kuujjua R., the head of Minto Inl., Mt. Bumpus, Mt. Pelly, Read I., Richard Collinson Inl., Ulukhaktok and Wollaston P. (Simmons 1913, Macoun and Holm 1921, Porsild 1955, 1957, 1964, Porsild and Cody 1980, Aiken et al. 2007). Thannheiser et al. (2001) additionally recorded it from Johansen B. (conf.) and Surrey L. Newly recorded from “30-Mile Cr.”, Boot Inl., Colville Mts., Falaise B., Hadley B., Oterkvik Pt., a site southeast of the head of Prince Albert S. and Sinclair Cr. We have not seen a supporting voucher from the Albert Edward B. area, as mapped by Porsild and Cody (1980). Elsewhere in the Canadian Arctic recorded from Banks, Eglinton, Melville and Prince Patrick islands and on the mainland east to Adelaide P. (Porsild and Cody 1980, Cody et al. 2003, Aiken et al. 2007, Saarela et al. 2013, Saarela et al. 2017b). Records from Melville P. previously recorded as this species have been redetermined as *Oxytropis bellii* Palibine.

**NORTHWEST TERRITORIES. Boot Inl.**: *Dutilly 18745* (DAO), *Gillespie et al. 9511a*, *9511b* (ALA, CAN). **C. Wollaston**: *Edlund 16*, *60* (CAN). **Kuujjua R.**: *Gillespie et al. 9712* (ALA, CAN, MT, O). **Minto Inl. (head)**: *Edlund 71* (CAN), *Gillespie et al. 10017b* (CAN), *9474* (ALA, CAN), *Porsild 17410* (CAN). **Richard Collinson Inl.**: *Edlund 134* (CAN). **Ulukhaktok**: *Edlund 301* (CAN), *Gray & Gibbard 27* (DAO), *Pokiak 36* (CAN), *Ross 37* (ALTA), *Saarela & Bull 1423* (ALA, CAN). **NUNAVUT. “30-Mile Cr.**”: *Bennett et al. 14-0363* (BRY). **Cambridge Bay**: *Bennett et al. 13-0205* (ALA, BABY, chars, UBC, od), *Beschel 13492* (CAN), *Consaul & Gillespie 1100* (CAN), *Edlund & Argus 12674* (CAN), *Gillespie et al. 8477* (ALA, CAN, O), *Gould s.n.* (ALA), *Porsild 21639* (CAN), *Stephens 985* (CAN, KSTC). **Colville Mts.**: *Gillespie et al. 7776* (ALA, ALTA, BABY, CAN, MT, O, UBC, US). **Falaise B.**: *Eriksen et al. 939* (ALA), *Parker 9107* (ALA). **Hadley B.**: *Edlund 27*, *73* (CAN). **Johansen B.**: *Gillespie et al. 7820* (ALA, CAN, MT, O, UBC), *7979* (CAN), *7980* (ALA, CAN, O), *7981* (ALA, CAN, MT, O). **Mt. Bumpus**: *Edlund 229*, *258* (CAN). **Mt. Pelly**: *Stephens 1179* (CAN). **Oterkvik Pt.**: *Gillespie et al. 7462* (ALA, ALTA, BABY, CAN, MT, O, UBC, US), *7645b* (CAN). **Prince Albert S. (SE of head)**: *Edlund 86* (CAN). **Read I.**: *Porsild 17205* (CAN), *Ross 34* (ALTA). **Sinclair Cr.**: *Gillespie et al. 8294* (CAN, O), *8313* (CAN). **Wollaston P.**: *D. Jenness 387* (CAN).

***Oxytropis arctobia*** Bunge (O. nigrescens var. uniflora (Hook.) Barneby), Figs 39G, 38F–One-flowered locoweed | North American (N)

Previously recorded from Byron B., Cambridge Bay, C. Wollaston, Hadley B., Kuujjua R., the head of Minto Inl., Natkusiak P., the head and north side of Prince Albert S., Read I., Richard Collinson Inl. and Ulukhaktok (Porsild 1955, 1957, 1964, Porsild and Cody 1980, Aiken et al. 2007, Saarela et al. 2013). Thannheiser et al. (2001) additionally recorded it from Johansen B., Mt. Pelly, Surrey L. and Wellington B. Newly recorded from Colville Mts., Falaise B. and Walker B. Elsewhere in the Canadian Arctic recorded from Baffin, Banks, Eglinton, King William, Melville and Southampton islands and mainland Nunavut and Northwest Territories sites (Porsild and Cody 1980, Cody and Reading 2005, Aiken et al. 2007, Saarela et al. 2013, Saarela et al. 2017b).

**NORTHWEST TERRITORIES. Boot Inl.**: *Gillespie et al. 9593* (ALA, CAN). **C. Wollaston**: *Edlund 39* (CAN). **Kuujjua R.**: *Gillespie et al. 9711* (ALA, CAN, O). **Minto Inl. (head)**: *Edlund 75* (CAN), *Gillespie et al. 10026* (CAN). “**Oldenburg L.**”: *Oldenburg 45-1339* (CAN). **Prince Albert S. (N)**: *Oldenburg 46-2287* (CAN). **Prince Albert Sound (head)**: *Weerstra 13* (DAO). **Richard Collinson Inl.**: *Edlund 133* (CAN). **Ulukhaktok**: *Edlund 295* (CAN), *Gray & Gibbard 20* (DAO), *Ross 22* (ALTA), *Saarela & Bull 1429* (ALA, CAN, O), *Wolki 42-81A* (CAN). **Walker B.**: *Oldenburg 45-1500* (CAN). **NUNAVUT. Byron B.**: *Dushenko 14* (UVIC). **Cambridge Bay**: *Bennett 14-0308* (BABY), *Bennett et al. 13-0211* (ALA, BABY, chars), *Calder et al. 24198* (DAO), *Edlund & Argus 12629* (ALA, CAN), *Fortier 18* (CAN), *Gillespie* (CAN), *Gillespie et al. 8412* (CAN), *Gould s.n.* (ALA), *Oldenburg 44*-*898* (CAN), *Parker & Jonsdottir 9094* (ALA), *Polunin s.n.* (CAN), *Stephens 1091* (CAN, KANU, KSTC), *939* (CAN, KANU, KSTC), *Sweatman 16* (DAO), *Tasker 3767* (CAN), *Washburn 9* (CAN). **Colville Mts.**: *Gillespie et al. 7774* (ALA, CAN, MT, O). **Falaise B.**: *Eriksen et al. 927* (ALA), *Parker 9108* (ALA). **Hadley B.**: *Edlund s.n.*, *26*, *162*, *163* (CAN). **Natkusiak P.**: *Edlund 341* (CAN). **Oterkvik Pt.**: *Gillespie et al. 7463* (ALA, CAN, O), *7645a* (CAN), *Gillespie et al. 7717* (ALA, CAN). **Read I.**: *Porsild 17206* (CAN), *Wood 85* (CAN).

***Oxytropis deflexa*** var. ***foliolosa*** (Hook.) Barneby (O. deflexa subsp. foliolosa (Hook.) Cody), Figs 39H, 38G–Foliose locoweed | Amphi-Beringian–North American (W)

Known from a single collection at the head of Minto Inl., where gathered on cobble flats along a river in the vicinity of dense stands of *Salix alaxensis*; see additional details in Gillespie et al. (2015). Elsewhere in the Canadian Arctic recorded from Baffin I. and mainland sites (Porsild and Cody 1980, Cody and Reading 2005, Aiken et al. 2007, Saarela et al. 2013, Saarela et al. 2017b).

**NORTHWEST TERRITORIES. Minto Inl. (head)**: *Gillespie et al. 10129* (CAN).

***Oxytropis maydelliana*** Trautv. (O. maydelliana subsp. melanocephala (Hook.) A.E.Porsild), Figs 39I, 38H–Maydell’s locoweed | Amphi-Beringian–North American (N)

Previously recorded from Byron B., Cambridge Bay, “Long L.”, Minto Inl. (*Anderson*, K), Tahoe L. and Ulukhaktok (Porsild obs., conf.) (Porsild 1955, 1957, 1964, Porsild and Cody 1980, Aiken et al. 2007). Thannheiser et al. (2001) additionally recorded it from Johansen B. (conf.), Mt. Pelly, Richardson I., Surrey L. and Wellington B. Newly recorded from Albert Edward B., Oterkvik Pt. and Sinclair Pt. Elsewhere in the Canadian Arctic recorded from Baffin, Banks, Bylot, Igloolik, King William, Melville, Southampton and West Foxe islands and across the mainland (Porsild 1955, Porsild and Cody 1980, Cody et al. 1989, Korol 1992, Cody and Reading 2005, Saarela et al. 2013, Saarela et al. 2017b).

**NORTHWEST TERRITORIES. Ulukhaktok**: *Bliss s.n.* (ALTA). **NUNAVUT. Albert Edward B.**: *Ponomarenko VI-257* (CAN). **Byron B.**: *Dushenko 44* (UVIC). **Cambridge Bay**: *Bennett et al. 13-0320* (BABY, chars, O, UBC), *Calder et al. 24182* (DAO), *Edlund & Argus 12632* (CAN), *Fortier 22* (CAN), *Gillespie et al. 8396* (ALA, CAN), *Gould s.n.* (ALA), *Oldenburg 44*-*910*, *44*-*957* (CAN), *Polunin s.n.* (CAN, 2 sheets), *Stephens 1135* (CAN, KANU, KSTC), *940* (CAN, KANU, KSTC), *Sweatman & Smith 22* (DAO). **Johansen B.**: *Gillespie et al. 7822* (ALA, ALTA, BABY, CAN, MT, O, UBC, US). “**Long L.**”: *Lambert s.n.* (CAN). **Oterkvik Pt.**: *Gillespie et al. 7515* (ALA, BABY, CAN, MT, O, UBC), *Gillespie et al. 7572* (CAN). **Sinclair Cr.**: *Gillespie et al. 8345* (CAN). **South-central Victoria I.**: *Edlund 540* (CAN). **Tahoe L.**: *Porsild 17455* (CAN).

***Oxytropis varians*** (Rydb.) K.Schum (O. campestris var. varians (Rydb.) Barneby, *O. hyperborea* A.E.Porsild), Fig. 39J–Late yellow locoweed | North American

Previously recorded from Ulukhaktok and Wollaston P. (Macoun and Holm 1921, Porsild 1955, 1957, 1964, Porsild and Cody 1980, Aiken et al. 2007). Thannheiser et al. (2001) additionally recorded it from Johansen B., Wellington B. and Mt. Pelly. Newly recorded from Read I. and Walker B. Elsewhere in the Canadian Arctic recorded from Banks I. and a few mainland sites (Porsild and Cody 1980, Saarela et al. 2013, Saarela et al. 2017b) east to the Coppermine R. The Victoria I. populations mark the northeastern limit of its range.

**NORTHWEST TERRITORIES. C. Baring**: *Edlund & Nixon 312* (CAN). **Ulukhaktok**: *Bliss s.n.* (ALTA), *Edlund 355*, *459*, *780* (CAN), *Oldenburg 42-111* (CAN), *Porsild 17317* (CAN), *Salokangas 28* (CAN). **Walker B.**: *Oldenburg 45-1496* (CAN). **NUNAVUT. Read I.**: *Oldenburg 43-1055* (CAN). **Wollaston P.**: *D. Jenness 386* (CAN).

### Rosales

**Rosaceae [3/14/15]**

**Key to Rosaceae**

**Table:** 

1	Dwarf shrubs (woody); styles persistent and elongating after anthesis	***Dryas integrifolia***
–	Herbs (non-woody); styles deciduous, not elongating after anthesis	**2**
2	Leaves cauline, simple, blades broadly 3–7-lobed; inflorescences 1-flowered; flowers unisexual; petals white; fruits aggregated drupelets	***Rubus chamaemorus***
–	Leaves basal or cauline, compound, blades ternate, palmate, subpalmate or odd-pinnate, leaflets 3+; inflorescences 1–many-flowered; flowers bisexual; petals yellow; fruits aggregated achenes	*** Potentilla ***

### *Dryas* L. [1]

**Figure 40.**
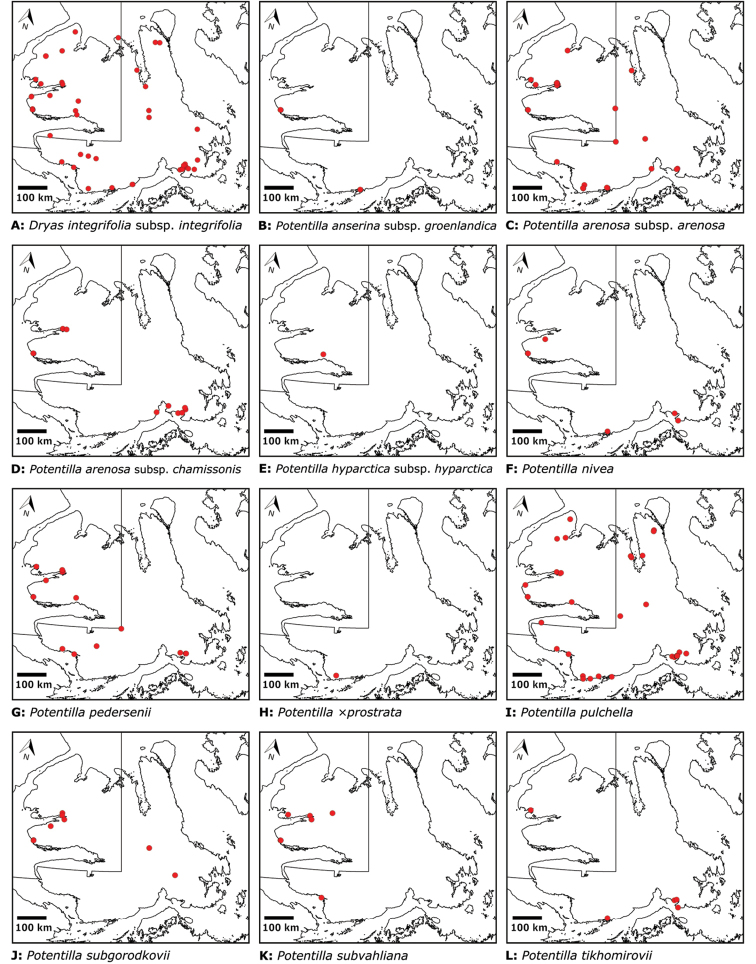
Species distribution maps. Rosaceae: **A**Dryas integrifolia subsp. integrifolia**B**Potentilla anserina subsp. groenlandica**C**Potentilla arenosa subsp. arenosa**D**Potentilla arenosa subsp. chamissonis**E**Potentilla hyparctica subsp. hyparctica**F***Potentilla nivea***G***Potentilla pedersenii***H***Potentilla × prostrata***I***Potentilla pulchella***J***Potentilla subgorodkovii***K***Potentilla subvahliana***L***Potentilla tikhomirovii*.

**Figure 41.**
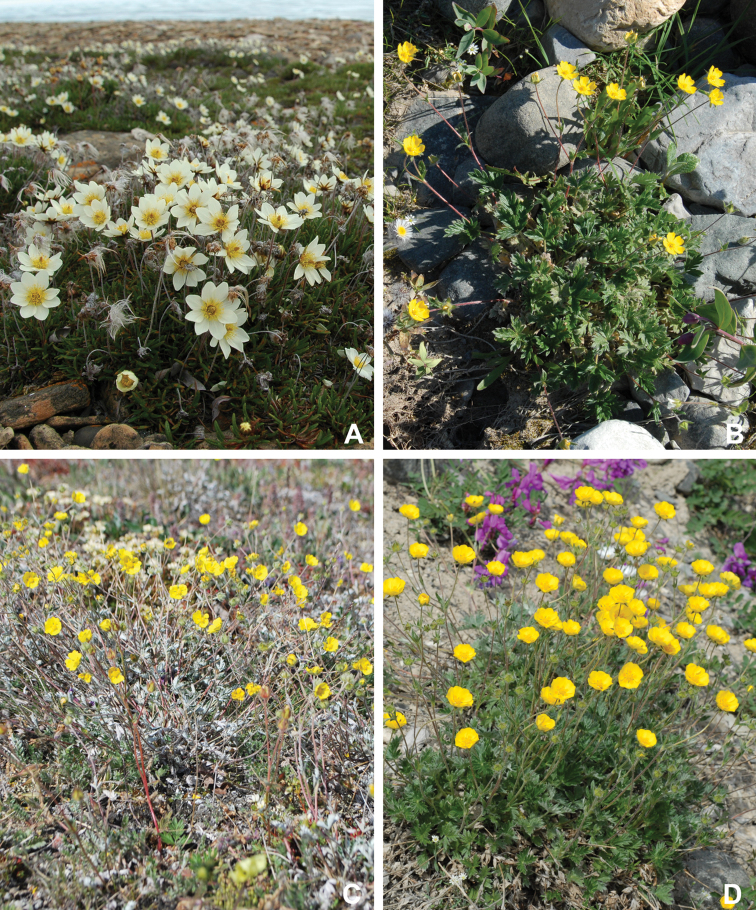
**A**Dryas integrifolia subsp. integrifolia habit, Oterkvik Point, NU, 7 July 2008 **B**Potentilla arenosa subsp. arenosa habit, *Gillespie et al. 10116a***C***Potentilla nivea* habit **D***Potentilla uschakovii* habit, *Gillespie et al. 9831*. Photos **A**, **B**, **D** by L.J. Gillespie and **C** by B.A. Bennett.

***Dryas integrifolia*** Vahl subsp. ***integrifolia***, Figs 40A, 41A–Mountain avens | Amphi-Beringian–North American (N)

Previously recorded from Byron B., Cambridge Bay, C. Wollaston, Hadley B., “Long L.”, the head of Minto Inl., Mt. Bumpus, Natkusiak P., Namaycush L., the north side of Prince Albert S., Richard Collinson Inl., Wollaston P. and Ulukhaktok (Porsild 1955, 1957, 1964, Porsild and Cody 1980, Aiken et al. 2007). Thannheiser et al. (2001) additionally recorded it from Johansen B. (conf.), Mt. Pelly, Richardson I., Surrey L. and Wellington B. Newly recorded from Boot Inl., Colville Mts., Falaise B., Greiner L., Kuujjua R., Namaycush L., “Oldenburg L.”, Oterkvik Pt., Prince Albert P., Read I., Sinclair Cr., Storkerson P., Walker B. and a site on eastern Victoria I. Widespread throughout the Canadian Arctic Archipelago and across the mainland (Porsild and Cody 1980, Aiken et al. 2007, Saarela et al. 2013, Bailleul 2015, Saarela et al. 2017b).

**NORTHWEST TERRITORIES. Boot Inl.**: *Gillespie et al. 9709* (ALA, CAN, MT, O, UBC). **C. Wollaston**: *Edlund 30*, *125* (CAN). **Kuujjua R.**: *Gillespie et al. 9767* (ALA, CAN, MT, O, UBC). **Minto Inl. (head)**: *Edlund 53* (CAN), *Gillespie et al. 9462* (CAN). **Natkusiak P.**: *Edlund 113*, *213* (CAN). “**Oldenburg L.**”: *Oldenburg 45-1393* (CAN, GH). **Prince Albert P.**: *Oldenburg 54-252* (GH). **Prince Albert S. (N)**: *Oldenburg 46-2288* (CAN), *Stretton 38* (DAO). **Richard Collinson Inl.**: *Edlund 93*, *155* (CAN), *Stretton 206* (DAO). **Ulukhaktok**: *Bliss s.n.* (ALTA), *Edlund 350*, *351*, *848*, *849* (CAN), *Gray & Gibbard 10*, *14*, *41* (DAO), *Oldenburg 42-70A*, *45-1646*, *44*-*947*, *45-1530A* (CAN), *42-23* (CAN, MIN), *54*-*214* (GH, MIN), *Porsild 17311* (CAN), *Ross 15* (ALTA), *15A* (GH), *Saarela & Bull 1433* (ALA, ari, CAN, O, UBC, WIN), *Stretton 75* (DAO). **Walker B.**: *Oldenburg 45-1530A* (CAN). **NUNAVUT. Byron B.**: *Dushenko* (UVIC). **Cambridge Bay**: *Bennett et al. 13-0618* (CAN), *13-0563* (BABY), *13-0246* (ALA, BABY, chars, UBC), *Edlund & Argus 12663* (CAN), *Fortier 15* (CAN), *Gibson 7076* (ALTA, DAO), *Gillespie et al. 8389* (ALA, CAN, MT, O), *Gould s.n.* (ALA), *Oldenburg 44*-*947* (CAN), *Polunin s.n.* (CAN, 2 sheets), *Stephens 941*, *1092* (CAN, KSTC), *Tasker s.n.* (CAN), *Washburn 33* (GH), *36* (CAN). **Colville Mts.**: *Gillespie et al. 7772* (ALA, CAN, O). **Eastern Victoria I.**: *Lee & Kittle s.n.* (CAN). **Falaise B.**: *Eriksen et al. 965* (ALA). **Greiner L.**: *Ponomarenko VI-027*, *VI-131*, *VI-220*, *VI-234* (CAN). **Hadley B.**: *Edlund 60*, *90* (CAN). **Johansen B.**: *Gillespie et al. 7867* (ALA, CAN, MT, O, UBC). “**Long L.**”: *Lambert s.n.* (CAN, 2 sheets). **Mt. Bumpus**: *Edlund 235* (CAN). **Namaycush L.**: *Edlund & Roncato-Spencer 12*, *30* (CAN). **Oterkvik Pt.**: *Gillespie et al. 7464* (ALA, ALTA, baby, CAN, MT, O), *7486* (CAN, O). **Read I.**: *Oldenburg 43-1058*, *43-1059* (CAN), *Ross 2A* (GH) . **Sinclair Cr.**: *Gillespie et al. 8315* (ALA, CAN, MT, O). **Storkerson P.**: *Edlund 218* (CAN), *P. Jenness 37* (CAN). **Washburn L.**: *Oldenburg 46-2160* (CAN, GH), *42-2161* (CAN). **Wollaston P.**: *Johansen & D. Jenness 573* (CAN), *Oldenburg 54*-*510* (GH, MIN).

### *Potentilla* L. [11/13]

**Key to *Potentilla* [adapted from Ertter et al. (2014), Elven and Ertter (2014) and Elven et al. (2014)]**

**Table:** 

1	Plants stoloniferous; stems flagelliform, becoming prostrate, rooting at some nodes; inflorescences solitary flowers at stolon nodes (P. sect. Pentaphylloides)	***P. anserina***
–	Plants not stoloniferous; stems not flagelliform, usually decumbent to erect, sometimes prostrate or pendent, not rooting at nodes; inflorescences usually cymose, sometimes racemiform or solitary flowers	**2**
2	Leaves pinnate to subpalmate (P. sect. Pensylvanicae)	***P. pulchella***
–	Leaves ternate or palmate	**3**
3	Leaflets pale to dark green abaxially, sometimes reddish, grayish or brownish, cottony or crisped hairs absent (P. sect. Aurea)	***P. hyparctica***
–	Leaflets gray to white abaxially, sometimes yellowish white or reddish, cottony and/or crisped (short, twisted) hairs abundant to dense	**4**
4	Leaflets 3, rarely more, on all basal leaves (P. sect. Niveae)	**5**
–	Leaflets 3–5(–7), usually more than 3 on at least some leaves (P. sect. Rubricaules)	**11**
5	Epicalyx bractlets usually 1/2 or less as wide as sepals, margins usually flat; petiole vestiture either primarily of cottony hairs or of ± stiff verrucose hairs; inflorescences usually more than 1-flowered; central leaflets with distal (1/2–)3/4 to nearly whole length incised less than 1/2 to midvein, teeth (2–)3–8(–12) per side	**6**
–	Epicalyx bractlets (1/2–)2/3 to ± as wide as sepals, margins often revolute; petiole vestiture primarily of soft to weak smooth hairs (or stiff verrucose hairs in *P. tikhomirovii*); inflorescences often only 1-flowered; central leaflets with distal (1/3–)1/2–2/3(–3/4) incised (1/3–)1/2–3/4 to midvein, teeth (1–)2–3(–4) per side	**8**
6	Petiole long hairs usually absent, sometimes sparse to common, usually soft, usually ± appressed, smooth, cottony hairs usually abundant to dense; central leaflets subsessile or short-petiolulate	***P. nivea***
–	Petiole long hairs sparse to abundant, usually stiff, spreading to ± ascending, verrucose, cottony hairs absent; central leaflets usually petiolulate, petiolules to 5 mm (***P. arenosa***)	**7**
7	Petioles with common to abundant short and/or stiff crisped hairs in addition to long verrucose hairs	***P. arenosa*** subsp. ***arenosa***
–	Petioles with sparse or no short and/or soft crisped hairs in addition to long verrucose hairs	***P. arenosa*** subsp. ***chamissonis***
8	Epicalyx bractlets usually with red glands; petiole long hairs verrucose	***P. tikhomirovii***
–	Epicalyx bractlets lacking red glands; petiole long hairs smooth	**9**
9	Carpels with apical hairs sparse to abundant (straight)	***P. vulcanicola***
–	Carpels with apical hairs usually absent, rarely present (cottony)	**10**
10	Petioles 0.3–1.5(–2) cm, crisped/short-cottony hairs usually absent, sometimes sparse, long hairs ± weak, rarely stiff; plants usually cushion-forming; basal leaves 0.5–2.5(–3) cm; sepals 3–5(–6) mm; petals 4–8(–9) × 4–9 m; caudex branches sheathed with marcescent whole leaves; abaxial leaf surfaces with long hairs 0.5–1 mm; pedicels 1–2(–3) cm in flower	***P. subvahliana***
–	Petioles (0.5–)1–5(–12) cm, crisped/short-cottony hairs usually sparse, sometimes absent or common, long hairs soft to weak; plants ± densely tufted to cushion-forming; basal leaves (1)2–10(–15) cm; sepals 4–6(–7) mm; petals (5–)6–9 × (5–)7–9 mm; caudex branches not or sometimes sheathed with marcescent whole leaves; abaxial leaf surfaces with long hairs 0.8–1.5 mm; pedicels (0.5–)2–4 cm in flower	***P. subgorodkovii***
11	Inflorescences (1–)3–7-flowered; petiole long hairs 1–2 mm, weak to ± stiff, verrucose; leaflet teeth with apical tufts ± 1 mm; adaxial leaflet surfaces with short (short-crisped) hairs absent or sparse, rarely common, cottony hairs absent; caudex branches not sheathed with marcescent whole leaves; petals usually not overlapping	***P. pedersenii***
–	Inflorescences 1–3(–4)-flowered; petiole long hairs (1–)1.5–2.5 mm, soft to ± weak, smooth; leaflet teeth with apical tufts 1–1.5 mm; adaxial leaflet surfaces with short/crisped/cottony hairs common to abundant; caudex branches often sheathed with marcescent whole leaves; petals often overlapping	***P. uschakovii***

***Potentilla anserina*** subsp. ***groenlandica*** Tratt. (*Argentina egedii* (Wormsk.) Rydb., *Potentilla egedii* Wormsk.), Fig. 40B–Greenland silverweed | Amphi-Beringian–North American (N)–amphi-Atlantic–European (N)

Previously recorded from Ulukhaktok (Porsild 1955, 1957, 1964, Porsild and Cody 1980, Aiken et al. 2007). Thannheiser et al. (2001) additionally recorded it from Johansen B. (conf.) and Richardson I. Elsewhere in the Canadian Arctic recorded from SE Baffin I. and along the mainland coast (Porsild and Cody 1980, Korol 1992, Aiken et al. 2007, Saarela et al. 2013, Bailleul 2015, Saarela et al. 2017b). The Ulukhaktok population marks the northern limit of its range in Canada.

**NORTHWEST TERRITORIES. Ulukhaktok**: *Edlund 291*, *823* (CAN), *Porsild 17312* (CAN). **NUNAVUT. Johansen B.**: *Gillespie et al. 8006* (CAN).

***Potentilla arenosa*** (Turcz.) Juz. subsp. ***arenosa*** (P. nivea var. arenosa Turcz.), Figs 40C, 41B–Bluff cinquefoil | Asian (N/C)–amphi-Beringian–North American (N)

Previously recorded from south of Burns L., Cambridge Bay, the head of Minto Inl., southeast of the head of Prince Albert S., Richard Collinson Inl., a site east of Tahiryuaq along the territorial border, Ulukhaktok, Walker B. and Washburn L. Thannheiser et al. (2001) additionally recorded it from Hadley B. (conf.) and Johansen B. (conf.). Newly recorded from “30-Mile Cr.”, Boot Inl., Falaise B., Greiner L. and Oterkvik Pt. Elsewhere in the Canadian Arctic recorded from Axel Heiberg, Banks, Devon, Ellesmere, Fitzwilliam Owen and Melville islands and western mainland sites as far east as the Coppermine R. area (Porsild and Cody 1980, Aiken et al. 2007, Saarela et al. 2013, Saarela et al. 2017b).

**NORTHWEST TERRITORIES. Boot Inl.**: *Gillespie et al. 9602a*, *9602b* (CAN, O). **Burns L. (S)**: *Edlund 50* (CAN). **Minto Inl. (head)**: *Edlund* 88 (CAN), *Gillespie et al. 10020b*, *10116a* (CAN), *10064aa* (ALA, CAN, MT, O), *10068a* (ari, CAN, MT, O, UBC), *10071a*, *10083a*, *10186a* (CAN, O), *10186b*, *10201* (ALA, CAN, O). **Prince Albert S. (head)**: *Edlund 156b* (CAN). **Richard Collinson Inl.**: *P. Jenness 17* (CAN). **Tahiryuaq**: *Edlund 154* (CAN). **Ulukhaktok**: *Edlund 334b*, *783*, *853* (CAN), *Oldenburg 45-1648* (CAN, GH). **Walker B.**: *Porsild 17496* (CAN). **NUNAVUT. “30-Mile Cr.**”: *Bennett et al. 14-0332* (DAO, UBC, SRP). **Cambridge Bay**: *Smith & Sweatman 39* (CAN). **Falaise B.**: *Eriksen et al. 982* (ALA). **Greiner L.**: *Ponomarenko VI-038* (CAN). **Hadley B.**: *Edlund 23* (CAN). **Johansen B.**: *Gillespie et al. 7874*, *7973a* (ALA, BABY, CAN, MT, O, UBC), **Oterkvik Pt.**: *Gillespie et al. 7587* (ALA, CAN, MT, O), *7667* (ALA, CAN, O), *7797* (ALA, CAN, MT, O, UBC). **Prince Albert S. (head)**: *Edlund 94* (CAN). **Washburn L.**: *Porsild 17459* (CAN).

***Potentilla arenosa*** subsp. ***chamissonis*** (Hultén) Elven & D.F.Murray (*P. chamissonis* Hultén, P. nivea subsp. chamissonis (Hultén) Hiitonen, P. hookeriana subsp. chamissonis (Hultén) Hultén), Fig. 40D–Chamisso’s cinquefoil | North American (NE)–amphi-Atlantic–European (N)–Asian (NW)

Previously recorded from Cambridge Bay (*Anderson*, K, conf. by Porsild), the head of Minto Inl. and Ulukhaktok (Simmons 1913, Porsild 1955, 1957, 1964, Porsild and Cody 1980, Aiken et al. 2007). Thannheiser et al. (2001) additionally recorded it from Surrey L. Newly recorded from “30-Mile Cr.”, Ferguson L., Greiner L. and Mt. Pelly. Elsewhere in the Canadian Arctic recorded from Baffin, Banks, Devon, Ellesmere, King William and Southampton islands and mainland sites (Porsild and Cody 1980, Cody et al. 1984a, Aiken et al. 2007, Bailleul 2015, Saarela et al. 2017b).

**NORTHWEST TERRITORIES. Minto Inl. (head)**: *Gillespie et al. 10218a*, *10218b* (CAN, O), *Porsild 17405* (CAN). **Ulukhaktok**: *Oldenburg 45-1647* (CAN), *Porsild 17313* (CAN), *Ross 36* (ALTA). **NUNAVUT. “30-Mile Cr.**”: *Bennett et al. 14-0349* (ALA, SRP). **NUNAVUT. Cambridge Bay**: *Bennett et al. 13-0210* (BABY, DAO, od, UBC), *Oldenburg 44*-*944* (CAN, GH). **Ferguson L. [Tahiryuaq**]: *Bennett et al. 14-0433b* (ALA, chars). **Greiner L.**: *Ponomarenko VI-278* (CAN). **Ovayok TP**: *Bennett & Sullivan 13-0287* (ALA, chars).

***Potentilla hyparctica*** Malte subsp. ***hyparctica***, Fig. 40E–Arctic cinquefoil | Circumpolar

Newly recorded for Victoria I., from a single collection from the north side of Prince Albert S. collected by M. Oldenburg in 1946. Elsewhere in the Canadian Arctic recorded from Banks, Baffin, Somerset and Southampton islands, nearly all of the Queen Elizabeth islands, northeastern mainland Nunavut, the Coppermine R. area, Nunavut, and northern Quebec (Aiken et al. 2007, Bailleul 2015, Saarela et al. 2017b).

**NORTHWEST TERRITORIES. Prince Albert S. (N)**: *Oldenburg 46-2290* (CAN, GH).

***Potentilla nivea*** L., Figs 40F, 41C–Snow cinquefoil | Circumpolar-alpine

Previously recorded from C. Colborne (Aiken et al. 2007). Thannheiser et al. (2001) additionally recorded it from Richardson I. and Ulukhaktok (conf.). Newly recorded from Cambridge Bay, Kuujjua R., and Johansen B. The single Cambridge Bay occurrence was gathered on a ridge between West Bay and Flagstaff Beach, growing in mesic *Dryas integrifolia*/*Salix arctica* tundra on limestone till of ancient beach ridges with *Taraxacum phymatocarpum*, *Oxytropis arctica*, *Androsace septentrionalis*, *Papaver hultenii*, *Stellaria longipes*, *Cerastium beeringianum* and *Saxifraga tricuspidata*. Elsewhere in the Canadian Arctic recorded from NE Banks I., central to southern Baffin I. and across the mainland (Porsild and Cody 1980, Cody and Reading 2005, Aiken et al. 2007, Bailleul 2015, Saarela et al. 2017b).

**NORTHWEST TERRITORIES. Kuujjua R.**: *Gillespie et al. 9715* (CAN, mixed with *P. uschakovii*). **Ulukhaktok**: *Edlund 334*, *335* (CAN), *Oldenburg 42-38* (CAN). **NUNAVUT. Cambridge Bay**: *Bennett et al. 13-0300* (ALA, BABY, SRP). **C. Colborne**: *Edlund & Argus 12738* (CAN). **Johansen B.**: *Gillespie et al. 7973a* (BABY, CAN), *7974a* (ALA, CAN, O).

***Potentilla pedersenii*** (Rydb.) Rydb. (*P. rubricaulis* Lehm. pro parte), Fig. 40G–Pedersen’s cinquefoil | European (N)–Asian (N)–amphi-Beringian–North American (N)

This species was, until recently, included in a more broadly circumscribed *P. rubricaulis* Lehm., which is now understood to have a much narrower circumscription and range (Elven and Ertter 2014). Following the current taxonomic treatment, *P. pedersenii* is known on Victoria I. from Cambridge Bay, Falaise B., Greiner L., Kuujjua R., the head of Minto Inl., Mt. Bumpus, Mt. Pelly, the north side and head of Prince Albert S. and Read I. Of these sites, *P. rubricaulis* s.l. was not previously recorded from Cambridge Bay, Kuujjua R., Greiner L. and the north side of Prince Albert S. Elsewhere in the Canadian Arctic, material at CAN revised by J.M. Saarela records the species from Axel Heiberg, Baffin, Devon, Ellesmere and Melville islands as well as mainland Nunavut and Northwest Territories sites. The general status rank of this species in Northwest Territories is Undetermined (Working Group on General Status of NWT Species 2016), owing to the recent change in taxonomic status.

**NORTHWEST TERRITORIES. Kuujjua R.**: *Gillespie et al. 9929* (ALA, CAN, O). **Minto Inl. (head)**: *Gillespie et al. 10060*, *10201b* (CAN), *10062a*, *10125* (CAN, O). **Ulukhaktok**: *Edlund 734* (CAN), *Saarela & Bull 1424* (ALA, CAN), *1432* (ALA, ari, CAN, MT, O). **Walker B.**: *Oldenburg 45-1532A* (CAN, GH). **NUNAVUT. Cambridge Bay**: *Bennett et al. 13-0273* (BABY, CAN, chars). **Falaise B.**: *Eriksen et al. 980*, *995* (ALA). **Greiner L.**: *Ponomarenko VI-115* (CAN). **Mt. Bumpus**: *Edlund 275* (CAN). **Ovayok TP**: *Gould s.n.* (ALA), *Stephens 1177* (CAN, KSTC), *1178* (CAN, KSTC, 2 sheets). **Prince Albert S. (head)**: *Edlund 83* (CAN). **Prince Albert S. (N)**: *Oldenburg 46-2290* (CAN). **Read I.**: *Oldenburg 43-1082* (CAN, GH).

***Potentilla*** ×***prostrata*** Rottb., Fig. 40H–Amphi-Atlantic

First record for Victoria I., where known from a single collection from Oterkvik Pt. This taxon is considered to be a hybrid between *P. arenosa* and *P. nivea*. See Elven et al. (2011) for information on taxonomy. Elsewhere in the Canadian Arctic recorded from Baffin I. (e.g., *Blouin* 1, CAN 10069756) and northern Quebec and Labrador (Bailleul 2015).

**NUNAVUT. Oterkvik Pt.**: *Gillespie et al. 7534* (ALA, CAN, MT, O)

***Potentilla pulchella*** R.Br., Fig. 40I–Pretty cinquefoil | Circumpolar

Previously recorded from Cambridge Bay, Hadley B., the head of Minto Inl., Namaycush L., the north side and a site east of the head of Prince Albert S., Richard Collinson Inl., Storkerson P. and Ulukhaktok (Porsild 1955, 1957, 1964, Porsild and Cody 1980, Aiken et al. 2007). Thannheiser et al. (2001) additionally recorded it from Johansen B. (conf.) and Surrey L. Newly recorded from C. Wollaston, “Oldenburg L.”, Falaise B., Greiner L., Murray Pt., Oterkvik Pt., Read I. and Wollaston P. Elsewhere in the Canadian Arctic recorded from Axel Heiberg, Baffin, Banks, Bathurst, Coast, Devon, Eglinton, Ellesmere, Igloolik, Jenny Lind, King William, Melville, Prince of Wales, Prince Patrick, Salisbury, Somerset and Southampton islands, and across the mainland (Porsild and Cody 1980, Aiken et al. 2007, Saarela et al. 2013, Bailleul 2015, Saarela et al. 2017b).

**NUNAVUT. C. Wollaston**: *Edlund 54* (CAN). **Minto Inl. (head)**: *Gillespie et al. 10196*, *10250* (CAN, O), *Porsild 17406* (CAN). “**Oldenburg L.**”: *Oldenburg 45-1394* (CAN). **Prince Albert S. (N)**: *Stretton* 36 (DAO). **Richard Collinson Inl.**: *Edlund 183*, *699* (CAN). **Ulukhaktok**: *Edlund 806* (CAN), *Porsild 17314* (CAN), *Saarela & Bull 1435* (ALA, CAN, O, V), *1452* (CAN, O). **Wollaston P.**: *Oldenburg 54-509* (GH). **NUNAVUT. Cambridge Bay**: *Bennett et al. 13-0280* (ALA, BABY, chars, od), *Bennett et al. 13-0568* (BABY), *Gillespie et al. 8385* (ALA, CAN, MT, O), *8483* (ALA, CAN, O), *Oldenburg 44*-*942* (CAN), *Polunin s.n.* (CAN, 2 sheets), *Porsild 21636* (CAN), *Stephens 1068* (CAN, KSTC). **Falaise B.**: *Eriksen et al. 940* (ALA). **Greiner L.**: *Ponomarenko VI-041*, *VI-195*, *VI-206*, *VI-326* (CAN). **Hadley B.**: *Edlund 33*, *59*, *324* (CAN). **Johansen B.**: *Gillespie et al. 8130* (ALA, CAN, O). **Murray Pt.**: *Gillespie et al. 8216* (CAN, O). **Namaycush L.**: *Edlund & Argus 12802* (CAN). **Oterkvik Pt.**: *Gillespie et al. 7551* (ALA, CAN, MT, O, UBC), *7620* (CAN, O), *7653* (CAN, O), *7695* (ALA, CAN, MT, O, UBC). **Prince Albert S. (head)**: *Edlund & Argus 12823* (CAN). **Read I.**: *Oldenburg 42-480*, *43-1081*, *43-945* (CAN). **Storkerson P.**: *Edlund 209*, *233*, *234* (CAN).

***Potentilla subgorodkovii*** Jurtzev (*P. uniflora* Ledeb. pro parte), Fig. 40J–Sheenjek River cinquefoil | Amphi-Beringian

Recorded from Kuujjua R., the head of Minto Inl., Namaycush L., “Trunsky L.”, Ulukhaktok and Washburn L. This is one of two taxa treated here (the other is *P. vulcanicola*), following Elven et al. (2014), that Aiken et al. (2007) included in *P. uniflora* andmapped from Namaycush L., Ulukhaktok and northwestern Wollaston P. The *P. uniflora* records from Ulukhaktok and Namaycush L. have been redetermined as this species, and the one from Wollaston Land (*Jenness 573*, CAN, det. “*P. uniflora* agg. (non *P. uniflora* Ledeb.)” by Elven in 2009) is not included here because it is in poor condition and a reliable identification is not possible. Revision of material at CAN records the taxon elsewhere in the Canadian Arctic on northern Axel Heiberg, northern Baffin, Banks, Devon, Ellesmere, King William and Melville islands and mainland sites.

**NORTHWEST TERRITORIES. Kuujjua R.**: *Gillespie et al. 9715b* (ALA, CAN, O). **Minto Inl. (head)**: *Gillespie et al. 10187*, *10261b* (CAN), *9471b* (CAN, O). **Ulukhaktok**: *Edlund 736* (CAN), *Oldenburg 42*-24, *42-38* (CAN). **NUNAVUT. Namaycush L.**: *Edlund & Roncato-Spencer 113* (CAN). “**Trunsky L.**”: *Bennett et al. 14-0401* (ALA, BABY). **Washburn L.**: *Oldenburg 46-2159* (cf.) (CAN, GH).

***Potentilla subvahliana*** Jurtzev (*P. vahliana* Lehm. pro parte), Fig. 40K–High arctic cinquefoil | Amphi-Beringian–North American (N)

The Sangraun Hills site mapped in Aiken et al. (2007) as *P. vahliana* (which has a more easterly distribution and is not known from Victoria I.) is this species; we have not seen the voucher for the other one, mapped from east-central Victoria I. Thannheiser et al. (2001) recorded *P. vahliana* from the head of Minto Inl. (conf.), Richardson I. and Cambridge Bay; vouchers require review. Newly recorded from Boot Inl., Read I. and Ulukhaktok. Based on revised material at CAN, following current taxonomy, elsewhere in the Canadian Arctic recorded from Axel Heiberg, northern Baffin, Banks, Melville and Southampton islands and scattered mainland sites.

**NORTHWEST TERRITORIES. Boot Inl.**: *Gillespie et al. 9688* (ALA, CAN, O). **Minto Inl. (head)**: *Gillespie et al. 10217*, *10261a* (CAN), *10259* (ALA, CAN, MT, O). **Sangraun Hills**: *Edlund 545* (CAN). **Ulukhaktok**: *Edlund 881* (CAN), *Gray & Gibbard 2* (DAO), *Oldenburg 42-18* (CAN). **NUNAVUT**. **Read I.**: *Oldenburg 43-918* (CAN).

***Potentilla tikhomirovii*** Jurtzev, Fig. 40L–Tikhomirov’s cinquefoil | Circumpolar

Newly recorded from Victoria I., where known from Cambridge Bay, C. Colborne, Johansen B. and Walker B. Thought to be a hybrid species of P. arenosa subsp. arenosa (P. sect. Nivea) and *P. hyparctica* (P. sect. Aurea), and may have multiple origins (Elven et al. 2014). Known from scattered sites across the mainland and the Arctic islands, based on revision of material at CAN. Some mainland records are recorded in Saarela et al. (2017b).

**NORTHWEST TERRITORIES. Walker B.**: *Oldenburg 45-1531A* (CAN, GH). **NUNAVUT. Cambridge Bay**: *Bennett et al. 13-0276* (ALA, BABY, chars), *Smith & Sweatman 39* (DAO). **C. Colborne**: *Edlund & Argus 12737* (CAN). **Johansen B.**: *Gillespie et al. 7973a2* (BABY).

**Figure 42.**
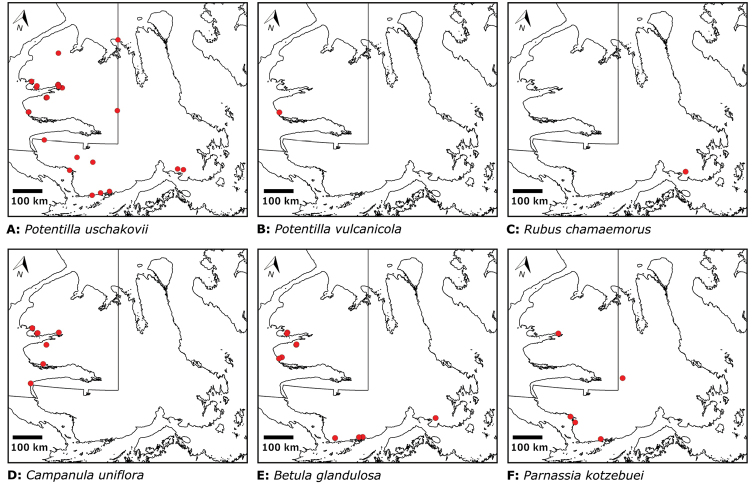
Species distribution maps. Rosaceae: **A***Potentilla uschakovii***B***Potentilla vulcanicola***C***Rubus chamaemorus*. Campanulaceae: **D***Campanula uniflora*. Betulaceae: **E***Betula glandulosa*. Celastraceae: **F***Parnassia kotzebuei*.

***Potentilla uschakovii*** Jurtzev (*P. rubricaulis* Lehm. pro parte), Figs 42A, 41D–Ushakov’s cinquefoil | Asian Beringian

This species was, until recently, included in a more broadly circumscribed *P. rubricaulis* Lehm., which is now understood to have a much narrower circumscription and range (Elven and Ertter 2014). Following the current taxonomic treatment, *P. uschakovii* is known from Boot Inl., Greiner L., Johansen B., Kuujjua R., the head of Minto Inl., Mt. Bumpus, Mt. Pelly, Natkusiak P., Oterkvik Pt., Read I., Richard Collinson Inl., a site east of Tahiryuaq along the territorial border, Ulukhaktok, Walker B. and Wollaston P. Based on the number of collections, it is apparently more common on the island than *P. pedersenii*. Elsewhere in the Canadian Arctic, material at CAN revised by J.M. Saarela records the species primarily from western Arctic islands (Banks, Eglinton, Melville) and some mainland sites in Northwest Territories and Nunavut. The general status rank of this species in Northwest Territories is Undetermined (Working Group on General Status of NWT Species 2016), owing to the recent change in taxonomic status.

**NORTHWEST TERRITORIES. Boot Inl.**: *Gillespie et al. 9527*, *9535b* (CAN, O), *9535a*, *9631* (CAN). **Kuujjua R.**: *Gillespie et al. 9715* (CAN, mixed with *P. nivea*), *9831* (ALA, ari, CAN, MT, O, UBC, WIN), *9836*, *9849* (ALA, CAN, O), *9987* (CAN, O). **Minto Inl. (head)**: *Edlund 89*, *175* (CAN), *Gillespie et al. 10020a*, *10061*, *10062b*, *10067* (CAN, O), *10072* (ALA, CAN, MT, O), *9472*, *10082*, *10117*, *10135* (CAN), *10089* (ALA, CAN), *10126*, *10140*, *10155* (CAN, O), *Porsild 17407*, *17408* (CAN). **Natkusiak P.**: *Edlund 124* (CAN). **Richard Collinson Inl.**: *Edlund 153*, *154* (CAN). **Tahiryuaq**: *Edlund 154* (CAN). **Ulukhaktok**: *Bandringa 316* (CAN, UBC), *Edlund 335b*, *506*, *734b*, *735* (CAN), *Porsild 17315* (ALTA, CAN), *Saarela & Bull 1424* (CAN). **Walker B.**: *Porsild 17497* (CAN). **Wollaston P. (NW)**: *Porsild 17227* (CAN). **NUNAVUT. Greiner L.**: *Ponomarenko VI-111* (CAN). **Johansen B.**: *Gillespie et al. 7973b*, *7974b* (CAN, O), *8123* (ALA, CAN, O). **Mt. Bumpus**: *Edlund 242* (CAN). **Ovayok TP**: *Gould s.n.* (ALA). **Oterkvik Pt.**: *Gillespie et al. 7687*, *7693* (ALA, CAN, O). **Read I.**: *Porsild 17203* (CAN). **Wollaston P.**: *D. Jenness 574* (CAN).

***Potentilla vulcanicola*** Juz. (*P. uniflora* Ledeb. pro parte), Fig. 42B–Kamchatka cinquefoil | Amphi-Beringian

We are aware of only a single record on Victoria I., from Ulukhaktok, which we were not able to confirm. Aiken et al. (2007) included this taxon in *P. uniflora*, which was mapped only from Ulukhaktok, Namaycush L. and Wollaston P. (see comments under *P. subgorodkovii*). Revision of material at CAN by J.M. Saarela records the taxon from Axel Heiberg, Banks and Melville islands and the Fosheim P., Ellesmere I., as well some western mainland sites. There are also records from the Milne Inl. area of northern Baffin I. (e.g., *Bennett 16-0507*, BABY, SRP). In the Canadian Arctic Archipelago, Elven and Ertter (2014) reported it as reaching only Banks and Victoria islands.

**NORTHWEST TERRITORIES. Ulukhaktok**: *Gray & Gibbard 47* (DAO, det. J. Cayouette, 2015).

### *Rubus* L. [1]

***Rubus chamaemorus*** L., Fig. 42C–Cloudberry | Circumboreal–polar

Known from a single collection gathered at “Long L.” (Aiken et al. 2007). Elsewhere in the Canadian Arctic recorded from southern Baffin, Southampton, Coats and King William islands, and across the mainland (Porsild and Cody 1980, Korol 1992, Aiken et al. 2007, Saarela et al. 2013, Bailleul 2015, Saarela et al. 2017b).

**NUNAVUT. “Long L.**”: *Lambert s.n.* (CAN) (Suppl. material 7).

### Campanulaceae [1/1]

***Campanula* L. [1]**

**Figure 43.**
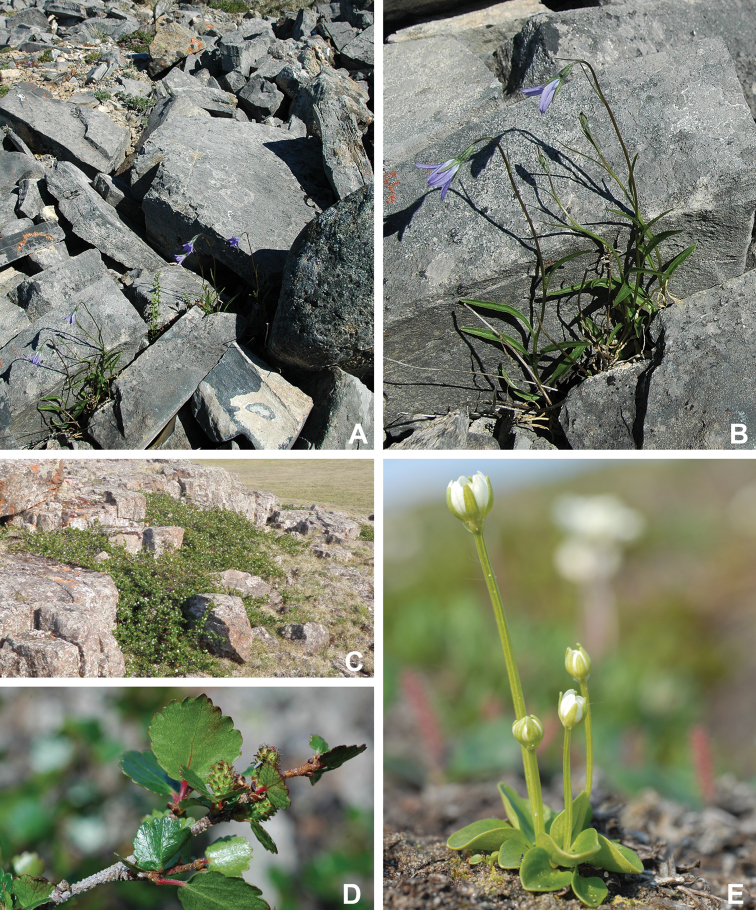
**A***Campanula uniflora* habitat, *Gillespie et al. 9680***B***Campanula uniflora* habit, *Gillespie et al. 9680***C***Betula glandulosa* habit, *Gillespie et al. 9623***D***Betula glandulosa* inflorescence and leaves, *Gillespie et al. 9623*. **E***Parnassia kotzebuei* habit, *Gillespie et al. 8127*. Photos **A**, **B**, **D**, **E** by R.D. Bull and **C** by L.J. Gillespie.

***Campanula uniflora*** L., Figs 42D, 43A, B–Arctic harebell | Amphi-Beringian–North American (N)–amphi-Atlantic

Previously known from Walker B. and the head of Minto Inl., the latter based on a specimen collected by Anderson housed at K and seen by Porsild (Porsild 1955, 1957, 1964, Porsild and Cody 1980, Aiken et al. 2007); we also collected the species at the head of Minto Inl. Newly recorded from Boot Inl., C. Baring, Kuujjua R. and Graveyard Bay on the north side of Prince Albert S. There are no records from the Nunavut part of Victoria I. Elsewhere in the Canadian Arctic known from Baffin, Banks, Coats, Devon, Ellesmere, Melville, Somerset and Southampton islands and mainland sites (Porsild and Cody 1980, Cody et al. 1989, Korol 1992, Cody and Reading 2005, Aiken et al. 2007, Saarela et al. 2013, Saarela et al. 2017b, Dignard 2018a).

**NORTHWEST TERRITORIES. Boot Inl.**: *Gillespie et al. 9594* (CAN), 9680 (ALA, ari, CAN, O). **C. Baring**: *Edlund 407* (CAN). **Kuujjua R.**: *Gillespie et al. 9818* (ALA, CAN, MT). **Minto Inl. (head)**: *Gillespie et al. 10106* (CAN, O). **Prince Albert S. (N)**: *Edlund 520* (CAN). **Walker B.**: *Oldenburg 45-1527B* (CAN), *Porsild 17498* (CAN).

### Fagales

**Betulaceae [1/1]**

***Betula* L. [1]**

***Betula glandulosa*** Michx., Figs 42E, 43C, D–Glandular birch | North American (N)

Previously recorded from Boot Inl., “Long L.”, Murray Pt. and Ulukhaktok (Porsild 1955, Aiken et al. 2007). Thannheiser et al. (2001) additionally recorded it from Richardson I. On Victoria I. the taxon grows in sheltered areas and on south-facing slopes. At Boot I., several small patches were found growing in the lee of rocks; plants grew to ca. 50 cm high. Elsewhere in the Canadian Arctic recorded from southern Banks, southern Baffin and Southampton islands as well as mainland sites (Porsild and Cody 1980, Cody et al. 1989, Korol 1992, Aiken et al. 2007, Saarela et al. 2013, Saarela et al. 2017b).

**NORTHWEST TERRITORIES. Boot Inl.**: *Edlund 577* (CAN), *Gillespie et al. 9623* (ari, CAN, MT, O, UBC). **Kuujjua R.**: *Gillespie et al. 9744* (ari, CAN, O, UBC, US, WIN), *9807* (ALA, CAN, O), *9819* (ALA, ari, CAN, O). **Ulukhaktok**: *Bliss s.n.* (ALTA), *Edlund 433*, *434*, *435*, *436*, *437*, *793* (CAN), *Porsild 17274* (ALTA, CAN). **NUNAVUT. Johansen B.**: *Gillespie et al. 7855* (CAN, O), *7919* (ALA, CAN, MT, O). “**Long L.**”: *Lambert s.n.* (CAN, 3 sheets). **Murray Pt.**: *Edlund 529* (CAN). **Oterkvik Pt.**: *Gillespie et al. 7599* (ALA, CAN, MT, O), *7805* (ALA, ALTA, BABY, CAN, MT, O, UBC).

### Celastrales

**Celastraceae [1/1]**

***Parnassia* L. [1]**

***Parnassia kotzebuei*** Cham. ex Spreng., Figs 42F, 43E–Kotzebue’s grass-of-Parnassus | Amphi-Beringian–North American (N)

Previously recorded from the Kagloryuak R. east of the head of Prince Albert S. (Aiken et al. 2007), but the location of the mapped point in that treatment is erroneous, being considerably south of the collecting site as described. Thannheiser et al. (2001) additionally recorded it from Hadley B. Newly recorded from the head of Minto Inl., Clouston B., Johansen B. and Read I. Elsewhere in the Canadian Arctic recorded from Banks I., southern Baffin I. and mainland sites (Porsild and Cody 1980, Korol 1992, Cody et al. 2003, Aiken et al. 2007, Saarela et al. 2013, Blondeau 2015c, Saarela et al. 2017b).

**NORTHWEST TERRITORIES. Minto Inl. (head)**: *Gillespie et al. 10221b* (ALA, CAN, O). **Prince Albert S. (head)**: *Edlund & Argus 12820* (CAN). **NUNAVUT. Clouston B.**: *Gillespie et al. 7722* (ALA, CAN, MT, O). **Johansen B.**: *Gillespie et al. 8127* (ALA, CAN, MT, O, UBC). **Read I.**: *Oldenburg 43-980* (CAN).

### Malpighiales

**Salicaceae [1/10/11]**

***Salix* L. [10/11]**

**Key to *Salix* [adapted from Argus (2010)]:**

Note: Medial leaf blades are the “normal” leaves along a branch.

**Table:** 

1	Low to tall shrubs, not dwarf, 0.08–6 m	**2**
–	Dwarf shrubs, 0.01–0.15 m	**6**
2	Flowering before leaves emerge, catkins not on distinct leafy branchlets	**3**
–	Flowering as or just before the leaves emerge, catkins on distinct leafy branchlets	**5**
3	Ovaries glabrous	***S. richardsonii***
–	Ovaries hairy	**4**
4	Largest medial leaf blades abaxially densely tomentose or villous-tomentose, hairs wavy, adaxially dull, sparsely or moderately densely villous (floccose) to glabrescent; ovaries tomentose, villous or woolly; juvenile leaf blades densely woolly-tomentose abaxially, hairs white	**S. alaxensis var. alaxensis**
–	Largest medial leaf blades abaxially glabrous or sparsely silky, hairs straight or wavy, adaxially highly glossy, glabrous or sparsely short-silky; ovaries long- or short-silky; juvenile leaf blades glabrous, puberulent, pubescent or densely long-silky abaxially, hairs white, sometimes also ferruginous	***S. planifolia***
5	Petioles 1–27 mm, much longer than subtending buds; ovary stipes 0.3–2.8 mm; largest medial leaf blades narrowly elliptic, elliptic, oblanceolate or obovate, apex acute, acuminate, convex or rounded	***S. glauca*** var. ***stipulata***
–	Petioles 2–5.5 mm, usually shorter than or barely exceeding subtended buds; ovary stipes 0–0.5 mm; largest medial leaf blades narrowly oblong, narrowly to broadly elliptic, lanceolate or obovate, apex acuminate or acute	***S. niphoclada***
6	Catkins from subterminal buds	**7**
–	Catkins from lateral buds	**8**
7	Largest medial leaf blade glaucous abaxially, (8–)12–66 × 8–50 mm, 1–1.5× as long as wide, venation deeply impressed; pistillate abaxial nectaries present, 0.3–0.5 mm, rarely absent; pistillate adaxial nectary narrowly oblong, 0.5–1 mm; styles 0.2–0.3 mm; capsules 4.5–5 mm	***S. reticulata***
–	Largest medial leaf blades not glaucous abaxially, 5–32 × 7–18 mm, 1.1–2.8× as long as wide; venation not deeply impressed; pistillate abaxial nectaries absent; pistillate adaxial nectary narrowly oblong, oblong or ovate, 0.8–1.8 mm; styles 0.7–1.2 mm; capsules 4.8–8.25 mm	***S. polaris***
8	Ovaries glabrous; juvenile leaves reddish; staminate abaxial nectary 0.6–1 mm	**S. ovalifolia var. ovalifolia**
–	Ovaries hairy; juvenile leaves yellow green; staminate abaxial nectary 0.3–0.8 mm or absent	**9**
9	Largest medial leaf blades not glaucous abaxially	***S. polaris***
–	Largest medial leaf blades glaucous abaxially	**10**
10	Largest medial leaf blades abaxial surface glabrous, margins closely and prominently serrulate or crenulate, sometimes entire; ovary hairs ribbonlike, usually crinkled (refractive); staminate abaxial nectaries absent	***S. arctophila***
–	Largest medial leaf blades abaxial surface pilose or midrib sparsely short-silky, or apex long-silky bearded, hairs usually straight or wavy, margins entire; ovary hairs flattened, not crinkled (white, not refractive); staminate abaxial nectaries present or absent	***S. arctica***

**Figure 44.**
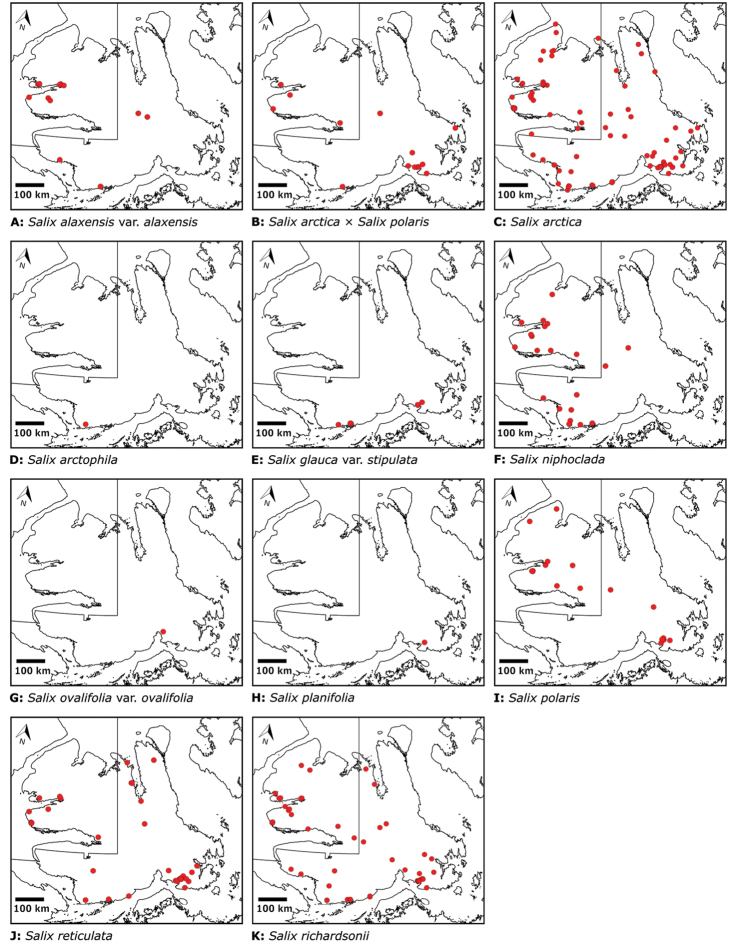
Species distribution maps. Salicaceae: **A**Salix alaxensis var. alaxensis**B***Salix arctica* × *Salix polaris***C***Salix arctica***D***Salix arctophila***E**Salix glauca var. stipulata**F***Salix niphoclada***G**Salix ovalifolia var. ovalifolia**H***Salix planifolia***I***Salix polaris***J***Salix reticulata***K***Salix richardsonii*.

**Figure 45.**
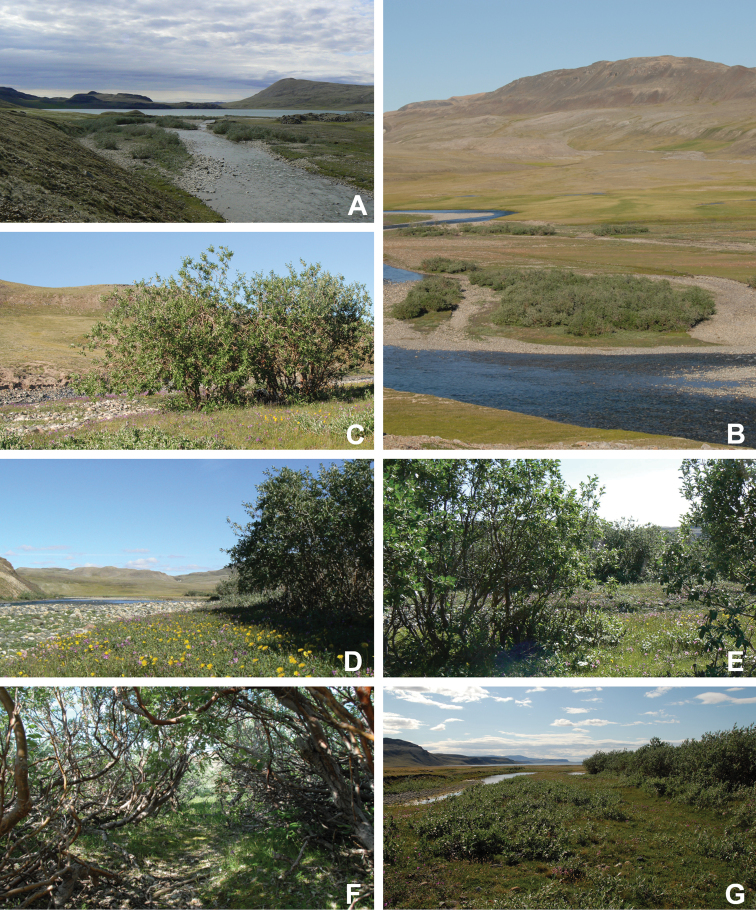
Salix alaxensis var. alaxensis**A** habitat, vicinity of Boot Inlet, *Gillespie et al 9577*, *9578***B** habitat at the head of Minto Inlet **C–G** habit at the head of Minto Inlet, *Gillespie et al 10191.* Photos **A**, **D** by P.C. Sokoloff and **B**, **C**, **E–G** by L.J. Gillespie.

***Salix alaxensis*** (Andersson ex DC.) Coville var. ***alaxensis***, Figs 44A, 45–Felt-leaf willow | Asian (N)–amphi-Beringian–North American (NW)

Previously recorded from Boot Inl., C. Wollaston, Kuujjua R., the head of Minto Inl., Namaycush L. Thannheiser et al. (2001) additionally recorded it from Johansen B. (conf.). Newly recorded from Falaise B. The habit of this species on Victoria I. varies dramatically, depending on habitat. For example, Edlund’s collection from Cape Wollaston, gathered from a gravel bar in a broad river channel, is recorded as having a prostrate growth form, whereas tree-sized thickets formed by this species occur along rivers in sheltered valleys around Minto Inl., at the edge of the species’ range (Aiken et al. 2007, Argus 2007). The latter are a remarkable feature of the otherwise low vegetation that characterizes the Minto Inl. area. Edlund (1983) regarded them as floristic oases. The Olokhaktomiut Community Conservation Plan 2016 (Olohaktomiut Hunters and Trappers Committee et al. 2016) identified “willow bushes” as unusual landscape features of the area, at Omingmakayok and Ungirut Bay (our Boot Inlet site) on the west and northeast sides of Boot Inlet, respectively, on the north side of Minto Inlet, Okpilik Lake, approximately 10 km north of Ulukhaktok, and along the rivers at the head of Minto Inlet (Kiyuktugak River (western river) and Kiyuktuluak River (eastern river)). The eastern river corresponds to the site visited by us and Edlund.

Edlund and Egginton (1984) reported a 1982 field study of the tree-sized willow thickets on floodplains near the head of Minto Inl. They recorded plants with heights up to 8 m and documented individuals as old as 81 years. Growth increments were found to be correlated with distance from the main river channels, with the greatest annual growth increments occurring within 30–50 metres of the channels and the lowest ones in the plants furthest from the channels. They suggested that the tree-sized growth form of these willows may be related to a warm microclimate in the sheltered valleys where the large thickets occur; snow cover in the winter, which protects at least the lower parts of plants from desiccation and abrasion; and moisture availability, which is enhanced along river channels. Zalatan and Gajewski (2009) developed a 74 year dendrochronology from populations of non-tree-sized *S. alaxensis* sampled in the Kuujjua R. area, and found winter precipitation to be correlated with growth ring width.

We studied the willow-thicket vegetation at a site in the valley of the Kiyuktuluak River, ca. 1 km north of the head of Minto Inl. on 24 July 2010. Based on comparison of site photographs published by Edlund (as well as previously unpublished photos, included here in Suppl. material 8) and our own photos (Fig. 45), we believe this is the same site studied by Edlund. The floodplain site was a mosaic of small thickets of willow (*S. alaxensis*) (no other species of willow was present) and small open grassy meadows with low scattered *S. alaxensis* reaching heights of up to 0.5 m. The trunk diameters of the five largest individuals in the largest thicket, measured near the base below the first branching split, were 40, 43.1, 43.3, 48.3 and 52 cm. These measurements are in approximately the middle of the range of variation for trunk diameter recorded at 10 m intervals along two transects at the same site, studied by Edlund.

Edlund (1983) suggested that the flora associated with the willow thickets, which is rich, is similar to that of communities near the tree line, some 400 km south. She did not, however, provide a list of species as validation (Edlund 1983, Edlund and Egginton 1984). With one exception, we found that none of the species growing in the understory or along the edges of the willow thickets, nor on the open rocky floodplain adjacent to the thickets, is unique to the site, as all are known elsewhere in the Minto Inl. area in non-willow thicket habitats. Rather than species diversity, Edlund may be been referring to the size and density of the associated vegetation, which, in the understory of the thicket and adjacent open meadow was lush, with plants being large and robust compared to the surrounding dwarf shrub tundra. Indeed, when inside the willow thicket, one gets the sense they have left the Arctic tundra.

Understory species present in the willow thicket included *Anemone parviflora*, Arnica angustifolia subsp. angustifolia, *Bistorta vivipara*, *Cardamine polemonioides*, *Cerastium arcticum*, *Chrysosplenium tetrandrum*, *Draba glabella*, Elymus alaskanus subsp. alaskanus, Erigeron eriocephalus subsp. eriocephalus, *Festuca baffinensis*, *Oxyria digyna*, Poa pratensis subsp. alpigena, *Potentilla uschakovii* and *Ranunculus arcticus*. In openings in the thickets we additionally recorded *Micranthes tenuis*, Silene involucrata subsp. involucrata and *Saxifraga caespitosa*. *Chamaenerion latifolium* was common around the edges of the thicket.

The lush grassy meadow along the river floodplain adjacent to the willow thickets comprised *Alopecurus borealis*, *Androsace septentrionalis*, *Bistorta vivipara*, Carex aquatilis subsp. stans (in wetter areas at the meadow edge), *Chamaenerion latifolium*, Elymus alaskanus subsp. alaskanus, *Festuca baffinensis*, Gentianella propinqua subsp. propinqua, Juncus arcticus subsp. alaskanus, Poa glauca subsp. glauca, P. pratensis subsp. alpigena, *Stellaria longipes*, low *S. alaxensis*, *Taraxacum ceratophorum* and *Trisetum spicatum*.

Species occurring on the drier river channel between the stable floodplain bars on which the thickets have developed included *Artemisia hyperborea*, *Astragalus richardsonii*, *Bistorta vivipara*, *Castilleja elegans*, *Cerastium arcticum*, *Chamaenerion latifolium*, *Dryas integrifolia* (dominant), *Papaver hultenii*, *Plantago canescens*, Poa glauca subsp. glauca, *Potentilla pedersenii*, *P. uschakovii*, *Ranunculus arcticus*, small, low plants of *S. alaxensis*, *Saxifraga cespitosa*, *S. hyperborea*, *Stellaria longipes* and *Taraxacum ceratophorum*. On the more mesic edges of the dry floodplain, sedges such as *Carex krausei* and *C. myosuroides* were present. Oxytropis deflexa var. foliolosa, which is the only species we recorded in this area not known from elsewhere on Victoria I., grew in the open rocky floodplain along the edges of the stable bars adjacent to the willow thickets. Additional plants seen in the vicinity of *O. deflexa* were *Astragalus alpinus*, *Draba* sp., *Festuca* sp. and *Saxifraga tricuspidata*.

The willow thickets at Boot Inl. were first recorded by Dutilly in 1940. His collection labels from this area record the site as “willows patch”, with imprecise coordinates that mark a site north of Boot Inlet. A report in The Polar Times (1940 no. 11) confirms the location of Dutilly’s “willows patch” as being in the Boot Inl. area, a site that subsequent collectors (including our team) have visited: “Willows in the Far North: [Inuit] told him of a valley in which a grove of willows was growing. Not far from Minto Inl., on Victoria Island, he found them in a sheltered valley about a quarter mile long. He said they had grown to a height of 7 or 8 feet. …”. The tallest individuals we observed in 2010, at a site in a river valley ca. 5 km inland from Boot Inl. on the northeast side of a small lake, reached ca. 3 m in height. At this site the willows formed dense thickets along the cobblestone floodplain of a small river, often growing with *Dryas integrifolia*. Edlund’s collections gathered in 1982 along the same river valley record the plants as reaching 3–4 m high. A map in Edlund and Egginton (1984) indicates the presence of this species along the same river as far east as the head of Minto Inl.; Edlund did not voucher the additional populations observed and it is unclear from available information how common are the tree-sized stands. Photographs of a large stand of this willow along Boot Inlet, downstream from the stand we explored, are included in Ecosystem Classification Group (2013).

At Johansen B., the species grew along Mackenzie Creek. Further inland, about 1 km from its mouth, it grew along the edges of the rocky, sheltered canyon of the creek, with most plants 1–1.5 m in height. Closer to the mouth of the creek it formed dense thickets along the partly flooded riparian zone along the creek; at this site most plants were about 1 m tall, with several in the grove reaching 1.5 m in height. Tree-sized willows also occur in the interior portion of Victoria Island. At a site 29 km east of Namaycush L., labels of Edlund’s collections (nos. 12838, 12839) record the species growing up to 3 m. Stefansson (1914) reported hearing of large, crooked willows in the valley of the Kagloryuak River that empties into Prince Albert Sound that “never stood quite as high as a mast head” (pp. 44). These willow stands, which likely are *S. alaxensis*, have not been documented by botanists.

**NORTHWEST TERRITORIES. Boot Inl.**: *Edlund 570*, *571*, *572A* (CAN), *Gillespie et al. 9577* (ALA, ari, CAN, MT, O), *9578* (CAN, O). **C. Wollaston**: *Edlund 26* (CAN). **Kuujjua R.**: *Edlund 531*, *666* (CAN), *Gillespie et al. 9958* (CAN, O). **Minto Inl. (head)**: *Edlund 52*, *131*, *144*, *153*, *590* (CAN), *Gillespie et al. 10141* (ALA, CAN, MT, O), *10183* (ALA, ari, CAN, MT, O, UBC, US, WIN), *Porsild 17379*, *17819* (CAN). **NUNAVUT. Falaise B.**: *Eriksen et al. 990* (ALA). **Johansen B.**: *Gillespie et al. 8134* (ALA, CAN, MT, O, UBC), *8145* (ALA, CAN, O). **Namaycush L.**: *Argus & Edlund 12834*, *12836*, *12837*, *12838*, *12839* (CAN).

**Figure 46.**
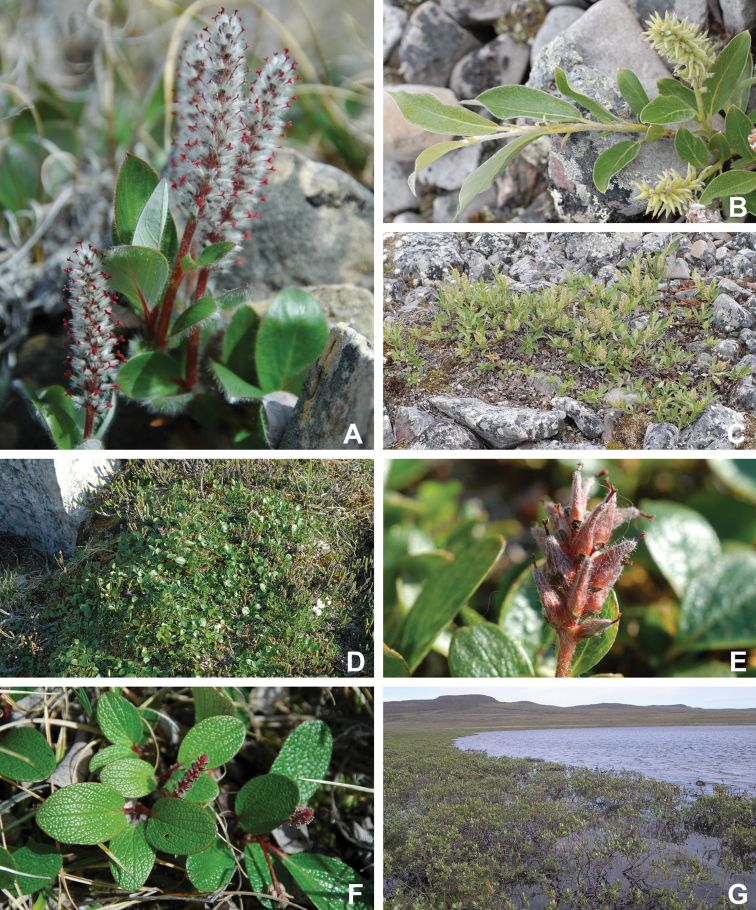
**A***Salix arctica* pistillate catkins, *Gillespie et al. 7484***B**Salix glauca var. stipulata pistillate catkins, *Gillespie et al. 8038***C**Salix glauca var. stipulata habit, *Gillespie et al. 8038***D***Salix polaris* habit, *Gillespie et al. 9991***E***Salix polaris* pistillate catkins, *Gillespie et al. 9991***F***Salix reticulata* habit, *Gillespie et al. 7475***G***Salix richardsonii* habitat, Johansen Bay, NU, 15 July 2008. Photos **A**, **F** by R.D. Bull and **B**, **C–E**, **G** by L.J. Gillespie.

***Salix arctica*** Pall., Figs 44C, 46A–Arctic willow | Circumpolar-alpine

There are more collections of this conspicuous and widespread species than of any other on Victoria I. Previously recorded from Albert Edward B., Anderson B., Byron B., Cambridge Bay, C. Wollaston, Collinson P., Ferguson L., Greely Haven, Kuujjua R., the head of Minto Inl., Mt. Bumpus, Mt. Pelly, Namaycush L., Natkusiak P., N of a large lake in the Ekalluk River system about 90 km NNE of Cambridge Bay, Peel Pt., north shore and head of Prince Albert S., Read I., Richard Collinson Inl., Storkerson P., Tahiryuaq, Ulukhaktok, Washburn L. and Wollaston P. (Porsild 1955, 1957, 1964, Porsild and Cody 1980, Aiken et al. 2007). Thannheiser et al. (2001) additionally recorded it from Johansen B. (conf.), Surrey L. and Wellington B. Newly recorded from Boot Inl., Cache Pt., Clouston B., Greiner L., Hadley B., “Oldenburg L.”, central Prince Albert P., Oterkvik Pt. and Sinclair Cr. Elsewhere in the Canadian Arctic recorded from most major Arctic islands except a few of the smaller Queen Elizabeth islands, and across the mainland (Porsild and Cody 1980, Aiken et al. 2007, Argus 2007, Saarela et al. 2017b).

**NORTHWEST TERRITORIES. Boot Inl.**: *Gillespie et al. 9580*, *9613* (CAN), *9581* (CAN, O, UBC), *9612* (CAN, O). **C. Wollaston**: *Edlund 4*, *27* (CAN). **Kuujjua R.**: *Edlund 620*, *668*, 669 (CAN), *Gillespie et al. 9871*, *9889*, *9959*, *9960* (CAN), *9872* (ALA, CAN, O). **Minto Inl. (head)**: *Edlund 77*, *87*, *139*, *141*, *142*, *268* (CAN), *Gillespie et al. 10203* (ALA, CAN, MT, O), *10204* (CAN, O), *9498* (CAN), *9499a* (ALA, ari, CAN), *Porsild 17380* (CAN). **Natkusiak P.**: *Edlund 74* (CAN). **Oldenburg L.**: *Oldenburg 45-1379* (MIN). **Peel Pt.**: *Edlund 423* (CAN). **Prince Albert P.**: *Oldenburg 54*-*683*, *54*-*685* (MIN). **Prince Albert S. (head)**: *Edlund 381* (CAN), *Shindman s.n.* (DAO). **Prince Albert S. (N)**: *Edlund 2* (CAN). **Richard Collinson Inl.**: *Edlund 198*, *672*, *695* (CAN). **Richard Collinson Inl.**: *P. Jenness 18* (CAN). **Tahiryuaq**: *Edlund 398*, *399* (CAN). **Ulukhaktok**: *Bandringa 307*, *343* (CAN, UBC), *Bliss s.n.* (ALTA, 2 sheets), *Edlund 329*, *330*, *331*, *471*, *495*, *739* (CAN), *Larsen s.n.* (CAN), *Pokiak 31* (CAN), *Oldenburg 42-72* (MIN), *Porsild 17272* (CAN), *17273* (ALTA, CAN), *Saarela & Bull 1411*, *1412* (CAN, O), *1439* (CAN), *Salokangas 22* (CAN, UBC). **NUNAVUT. Albert Edward B.**: *Argus & Edlund 12784* (CAN), *Ponomarenko VI*-*263* (CAN). **Anderson B.**: *Argus & Edlund 12702*, *12724* (CAN). **Byron B.**: *Dushenko 1* (UVIC). **Cache Pt.**: *Larsen s.n.* (CAN). **Cambridge Bay**: *Argus & Edlund 12611*, *12612*, *12621*, *12677*, *12894* (CAN), *Bennett et al. 13-0270* (BABY, CAN, chars), *Consaul & Gillespie 1137* (CAN), *Argus & Edlund 12866* (CAN), *Fortier 23* (CAN), *Gillespie et al. 8358* (ALA, CAN, O), *8505* (ALA, BABY, CAN, MT, O, UBC), *Polunin s.n.* (CAN, 3 sheets), *Porsild 17467*, *21610* (CAN), *Saarela & Teeter 5290*, *5300* (CAN), *Stephens 1060*, *1089* (CAN, KANU), *855*, *872*, *874*, *1133* (CAN, KANU, KSTC), *1271* (KANU, KSTC), *828* (CAN), *850* (CAN, KSTC), *873* (KSTC), *Washburn 15*, *18* (CAN). **Clouston B.**: *Gillespie et al. 7758* (CAN). **Collinson P.**: *Argus & Edlund 12747*, *12752* (CAN). **Eastern Victoria I.**: *Lee & Kittle s.n.* (CAN). **Falaise B.**: *Eriksen et al. 953* (ALA). **Ferguson L. [Tahiryuaq**]: *Argus & Edlund 12790* (CAN), *Hainault 1936* (CAN, DAO), *2021* (CAN, DAO), *2024*, *2025* (CAN). **Greiner L.**: *Ponomarenko VI-127*, *VI-138*, *VI-203B*, *VI-218B*, *VI-237A* (CAN). **Greely Haven**: *Fortier 98* (CAN). **Hadley B.**: *Edlund* 16, *105* (CAN). **Johansen B.**: *Gillespie et al. 7910*, *7927*, *7928*, *7982* (ALA, CAN, O). **Mt. Bumpus**: *Edlund 203*, *243*, *244* (CAN). **Ovayok TP**: *Stephens 860*, *861*, *989* (CAN, KANU), *863*, *867*, *990* (CAN, KANU, KSTC), *866* (KSTC). **Namaycush L.**: *Argus & Edlund 12841*, *12842* (CAN). **Oterkvik Pt.**: *Gillespie et al. 7484* (ALA, CAN, O), *7512*, *7521*, *7522*, *7651*, *7806* (CAN), *7565* (ALA, CAN, MT, O, UBC), *7566* (ALA, ALTA, BABY, CAN, MT, O, UBC), *7644* (ALA, CAN, MT, O). **Prince Albert S. (head)**: *Argus & Edlund 12827* (CAN). **Read I.**: *Oldenburg 42-517* (MIN), *Porsild 17192* (CAN), *Ross 20A*, *21A* (CAN). **Sinclair Cr.**: *Gillespie et al. 8266*, *8271* (CAN, O), *8267* (ALA, CAN, MT, O), *8268* (CAN), *8283* (ALA, CAN, O), *8349* (ALA, CAN, MT, O). **Ekalluk R.**: *Argus & Edlund 12742* (CAN). **Storkerson P.**: *Edlund 217* (CAN), *Schroder* 1 (DAO). **Tahoe L.**: *Porsild 17453* (CAN). **Tuktu R.**: *Gould s.n.* (ALA). **Washburn L.**: *Argus & Edlund 12797* (CAN), *Oldenburg 42-2168* (MIN). **Wollaston P.**: *D. Jenness 404*, *409*, *408a* (CAN), *Oldenburg 54-511* (MIN). **Wollaston P. (SW)**: *Edlund 524* (CAN).

***Salix arctophila*** Cockerell ex A.Heller, Fig. 44D–Northern willow | North American (N)

Known from a single collection on Victoria I. from Oterkvik Pt.; see Gillespie et al. (2015) for details. Elsewhere in the Canadian Arctic recorded from Baffin and Southampton islands and across the mainland (Porsild and Cody 1980, Korol 1992, Cody and Reading 2005, Aiken et al. 2007, Argus 2007, Saarela et al. 2013, Saarela et al. 2017b).

**NUNAVUT. Oterkvik Pt.**: *Gillespie et al. 7511* (ALA, CAN, MT, O).

***Salix glauca*** var. ***stipulata*** Flod. (S. glauca subsp. stipulifera (Flod. ex Hayren) Hiit.), Figs 44E, 46B, C–Northern grey-leaved willow | European (N)–Asian (N/C)–amphi-Beringian

Previously recorded only from Cambridge Bay (Aiken et al. 2007, Argus 2007). Newly recorded from Johansen B., where we made many collections. Elsewhere in the Canadian Arctic recorded from southern Banks I. and across the mainland (Porsild and Cody 1980, Aiken et al. 2007). The species is at the edge of its range on Victoria I. Most of our collections were made along the Nakoyoktok R. where it flows out of a small lake. In this area, the species grew as low shrub, ranging from prostrate (e.g., *Gillespie et al. 7856*, *7857*, *7858*, *7859*, *8038*, *8039*) to heights of 30 cm (*7860*, *786*). At one site in the area, it grew on a northwest facing boulder slope, reaching one metre in height (*7964*, *7965*), forming a thicket with *Salix richardsonii*. We also found the species on a pingo 23 km west of Johansen B. airstrip, where it reached 30 cm in height (*8154*, *8155*, *8156*). In the Cambridge Bay area, known from two sites discovered in 1987: 1.5 km NNW of the Dew Line station, where plants are recorded as reaching 20–25 cm tall (e.g., *Argus & Edlund 12892*, *12895*), and a site adjacent to the road on the NE side of the Dew Line Station, where a single plant reaching 25 cm tall was found (no. *12687*).

**NUNAVUT. Cambridge Bay**: *Argus & Edlund 12687*, *12890*, *12891*, *12892*, *12895*, *12896* (CAN). **Johansen B.**: *Gillespie et al. 7856* (CAN, O), *7857*, *8038*, *8156* (CAN), *7858*, *7859*, *7861*, *7964*, *7983*, *8039*, *8155* (ALA, CAN, O), *7860*, *7965*, *8154* (ALA, CAN, MT, O), *7926* (ALA, ALTA, BABY, CAN, MT, O, UBC), *7984* (ALA, CAN, MT, O, UBC).

***Salix niphoclada*** Rydb. (S. brachycarpa subsp. niphoclada (Rydb.) Argus), Fig. 44F–Barren-ground willow | Amphi-Beringian (E)

Previously recorded from Boot Inl., Kuujjua R., the head of Minto Inl., Mt. Bumpus, the north side and head of Prince Albert S., Richard Collinson Inl., Ulukhaktok and SW Wollaston P. (Porsild 1955, 1957, 1964, Porsild and Cody 1980, Aiken et al. 2007). Newly recorded from Clouston B., Falaise Bay, Johansen B., Oterkvik Pt. and Washburn L., which is the easternmost site on the island. Elsewhere in the Canadian Arctic recorded from southern Banks Island and across the mainland to Hudson Bay (Porsild and Cody 1980, Aiken et al. 2007, Argus 2007, Saarela et al. 2013, Saarela et al. 2017b).

**NORTHWEST TERRITORIES. Boot Inl.**: *Edlund 573* (CAN). **Kuujjua R.**: *Edlund 665*, *667* (CAN), *Gillespie et al. 9886* (CAN, O), *9887* (ari, CAN, O). **Minto Inl. (head)**: *Gillespie et al. 10014* (ALA, CAN, MT, O), *10290* (CAN), *10291* (ALA, CAN, O), *Porsild 17381* (CAN). **Prince Albert S. (head)**: *Edlund 373* (CAN). **Prince Albert S. (N)**: *Edlund 372*, *446* (CAN). **Richard Collinson Inl.**: *Edlund 686* (CAN). **Ulukhaktok**: *Edlund 742*, *846* (CAN), *Porsild 17270* (CAN). **NUNAVUT. Clouston B.**: *Gillespie et al. 7729* (CAN, O), *7730* (ALA, CAN, MT, O, UBC). **Falaise B.**: *Eriksen et al. 973* (ALA). **Johansen B.**: *Gillespie et al. 7924*, *8070* (ALA, CAN, MT, O), *7925*, *7930*, *8158* (ALA, CAN, O), *7929* (CAN, O), *8071*, *8157* (CAN), *8159* (ALA, CAN, MT, O, UBC). **Mt. Bumpus**: *Edlund 45*, *240*, *241* (CAN). **Oterkvik Pt.**: *Gillespie et al. 7482* (CAN, O), *7528*, *7541*, *7658*, *7794* (CAN), *7542*, *7656* (ALA, CAN, MT, O), *7795* (CAN, O). **Prince Albert S. (head)**: *Argus & Edlund 12809*, *12810*, *12811*, *12826*, *12828* (CAN). **Washburn L.**: *Oldenburg 46-2170* (MIN). **Wollaston P. (SW)**: *Edlund 527*, *528* (CAN).

***Salix ovalifolia*** Trautv. var. ***ovalifolia***, Fig. 44G–Oval-leaved willow | Amphi-Beringian

First record for Victoria I. and the Canadian Arctic Archipelago. Known from a single collection gathered at Ferguson L. (identification confirmed by G.W. Argus). Elsewhere in the Canadian Arctic recorded from northern Yukon (Argus 1969, 1973, Cody 2000) and a recently reported collection taken from Heart L. near Kugluktuk (Saarela et al. 2017b).

**NUNAVUT. Ferguson L. [Tahiryuaq**]: *Hainault 2022* (DAO) (Suppl. material 9).

***Salix planifolia*** Pursh, Fig. 44H–Tea-leaved willow | North American (N)

The single collection, gathered from a sedge meadow at “Long L.”, was originally determined as *Salix fuscescens* by G.W. Argus, in 1987, and accepted as that species in Aiken et al. (2007); it was the only record of *S. fuscescens* for the Canadian Arctic Archipelago. The specimen was re-determined as *Salix planifolia* by Argus in 2014, with the following text written on the sheet in his hand above his annotation label: “possibly: based on two ferruginous hairs ! [on the abaxial leaf surface]”. The collection is poor, comprising one shoot, ca. 10 leaves and no reproductive structures. We tentatively accept the taxon as *S. planifolia*, with the caveat that additional material from “Long L.” is needed to confirm its identity. Elsewhere in the Canadian Arctic *S. planifolia* is recorded from a few mainland Nunavut sites, southern Baffin Island (Soper River valley) and northern Quebec and Labrador (Porsild and Cody 1980, Cody et al. 1984a, Korol 1992, Cody and Reading 2005, Argus 2007, 2015, Saarela et al. 2017b).

**NUNAVUT. “Long L.**”: *Lambert s.n.* (CAN) (Suppl. material 10).

***Salix polaris*** Wahlenb., Figs 44I, 46D, E–Polar willow | Eurasian–amphi-Beringian–North American

Previously recorded from SE of Armstrong Pt., “Jackpot L.”, the head of Minto Inl., Tahiryuaq and an unnamed lake ca. 60 mi. N of Cambridge Bay (Aiken et al. 2007). Several specimens mapped as *S. polaris* in Aiken et al. (2007) are treated here under *S. arctica* × *S. polaris*, including *Argus & Edlund 12865* from Cambridge Bay, *Edlund 379* from the head of Prince Albert S., *Stephens 1167* from Mt. Pelly, *Edlund 723* from Ulukhaktok, *Argus & Edlund 12789* from Ferguson L., *Argus & Edlund 12676* from Cambridge Bay and *Argus & Edlund 12722* from Mt. Pelly. Thannheiser et al. (2001) additionally recorded it from Richardson I., Johansen B., Hadley B., Surrey L., Wellington B. and Mt. Pelly. Newly recorded from Cambridge Bay, Greiner L., “Oldenburg L.”, the north side of Prince Albert S. and Tuktu R. Elsewhere in the Canadian Arctic recorded from Banks, Eglinton and Melville islands and mainland sites eastwards to just beyond the Northwest Territories/Nunavut border (Porsild and Cody 1980, Argus 2007, Saarela et al. 2013).

**NORTHWEST TERRITORIES. SE of Armstrong Pt.**: *Edlund 589* (CAN). **Jackpot L.**: *Porsild 17502* (CAN). **Kuujjua R.**: *Gillespie et al. 10005* (CAN, O), *9759*, *9852*, *9852*, *9991*, *10006* (CAN). **Minto Inl. (head)**: *Gillespie et al. 10255*, *10256* (CAN), *Porsild 17382* (CAN). **Oldenburg L.**: *Oldenburg 45-1389* (MIN). **Prince Albert S. (N)**: *Oldenburg 46-2277* (MIN). **Tahiryuaq**: *Edlund 395* (CAN). **NUNAVUT. Cambridge Bay**: *Bennett & Sullivan 13-0295* (BABY, CAN, chars), *Polunin s.n.* (CAN). **Greiner L.**: *Ponomarenko VI-081*, *VI-213a*, *VI-218*, *VI-282* (CAN). **Tuktu R.**: *Gould s.n.* (ALA). **Unnamed lake ca. 60 mi. N of Cambridge Bay**: *Porsild 17473* (CAN).

***Salix reticulata*** L., Figs 44J, 46F–Net-vein willow | Circumpolar–alpine

Previously recorded from Anderson B., Byron B., Cambridge Bay, C. Wollaston, Ferguson L., Greiner L., Hadley B., the head of Minto Inl., Mt. Bumpus, Mt. Pelly, Natkusiak P., the head of Prince Albert S., Storkerson P. and Ulukhaktok. Thannheiser et al. (2001) additionally recorded it from Johansen B. (conf.), Richardson I., Surrey L. and Wellington B. Newly recorded from Albert Edward B., Boot Inl., Kuujjua R., Oterkvik Pt. and Washburn L. Elsewhere in the Canadian Arctic recorded from Air Force, Baffin, Banks, Bylot, Coats, Devon, Digges, Eglinton, Igloolik, King William, Nottingham, Prince Charles, Prince of Wales, Princess Royal, Resolution, Somerset and Southampton islands and across the mainland (Porsild and Cody 1980, Cody et al. 1989, Korol 1992, Cody and Reading 2005, Aiken et al. 2007, Saarela et al. 2013, Saarela et al. 2017b).

**NORTHWEST TERRITORIES. Boot Inl.**: *Gillespie et al. 9582* (CAN), *9583* (ALA, CAN, O). **C. Wollaston**: *Edlund 52*, *67* (CAN). **Kuujjua R.**: *Gillespie et al. 9791* (ari, CAN), *9792* (CAN, O). **Minto Inl. (head)**: *Edlund 138* (CAN), *Gillespie et al. 9499b* (CAN, O). **Prince Albert S. (head)**: *Edlund 380* (CAN). **Ulukhaktok**: *Bandringa 345* (CAN, UBC), *Bliss s.n.* (ALTA), *Edlund 456*, *502*, *749* (CAN), *Larsen s.n.* (CAN), *Oldenburg 45-1552* (MIN). **NUNAVUT. Albert Edward B.**: *Ponomarenko VI-264* (CAN). **Anderson B.**: *Argus & Edlund 12720* (CAN). **Byron B.**: *Dushenko 3* (UVIC) **. Cambridge Bay**: *Argus & Edlund 12620* (CAN), *Bennett et al. 13-0245* (ALA, chars, BABY, UBC), *Consaul & Gillespie 1106* (CAN), *Fortier 24* (CAN), *Gillespie et al. 8504* (ALA, CAN, O), *Gould s.n.* (ALA), *Polunin s.n.* (CAN), *Stephens 1087* (KANU). **Ferguson L. [Tahiryuaq**]: *Hainault 1975* (CAN, DAO). **Greiner L.**: *Ponomarenko VI-129*, *VI-203N*, *VI-237* (CAN). **Hadley B.**: *Edlund 31*, *49*, *142* (CAN). **Johansen B.**: *Gillespie et al. 7862* (CAN, O), *7863* (ALA, CAN, O). **Mt. Bumpus**: *Edlund 200* (CAN). **Ovayok TP**: *Argus & Edlund 12788* (CAN), *Stephens 1164* (CAN, KSTC), *869* (CAN), *991* (KANU, KSTC). **Natkusiak P.**: *Edlund 312* (CAN). **Oterkvik Pt.**: *Gillespie et al. 7475* (ALA, CAN, MT, O, UBC), *7476*, *7483* (CAN, O). **Storkerson P.**: *Edlund 345* (CAN). **Washburn L.**: *Oldenburg 46-2165* (MIN).

***Salix richardsonii*** Hook. (S. lanata subsp. richardsonii (Hook.) A.K.Skvortsov), Figs 44K, 46G–Richardson’s willow | Asian (N)–amphi-Beringian–North American (NW)

Previously recorded from Anderson B., Boot Inl., Cambridge Bay, Ferguson L., Jonnessee L., the head of Minto Inl., Mt. Bumpus, Mt. Lady Pelly, Mt. Pelly, N of a large lake in the Ekalluk River system about 90 km NNE of Cambridge Bay, the north side and east of the head of Prince Albert S., Richard Collinson Inl., Surrey L., Tahiryuaq, Ulukhaktok and Wollaston P. (Porsild 1955, 1957, 1964, Porsild and Cody 1980, Aiken et al. 2007). Thannheiser et al. (2001) additionally recorded it from Johansen B. (conf.) and Richardson I. Newly recorded from Falaise Bay, Greiner L., Hadley B., Kuujjua R., Oterkvik Pt., Sinclair Cr. and Walker Bay. Elsewhere in the Canadian Arctic recorded from Baffin (northern half), Banks, Prince of Wales and Southampton islands and across the mainland to Hudson Bay (Porsild and Cody 1980, Cody et al. 1984a, Korol 1992, Cody and Reading 2005, Aiken et al. 2007, Saarela et al. 2013, Saarela et al. 2017b).

**NORTHWEST TERRITORIES. Boot Inl.**: *Edlund 572B*, *582* (CAN), *Gillespie et al. 9603* (ALA, CAN, UBC). **Kuujjua R.**: *Dutilly 18864* (DAO), *Gillespie et al. 9800* (CAN), *9873*, *9888* (ALA, CAN, O), *9874* (ALA, CAN, MT, O), *9961* (CAN). **Minto Inl. (head)**: *Edlund 140*, *276* (CAN), *Gillespie et al. 10110* (CAN), *10184* (CAN, O). **Prince Albert S. (head)**: *Edlund 383* (CAN). **Prince Albert S. (N)**: *Edlund* 1 (CAN). **Richard Collinson Inl.**: *Edlund 128*, *199*, *670*, *673* (CAN). **Tahiryuaq**: *Edlund 396*, *397* (CAN). **Ulukhaktok**: *Bandringa 346* (CAN, UBC), *Edlund 474*, *737*, *738*, *763* (CAN), *Saarela & Bull 1503* (ari, CAN, O). **Walker B.**: *Oldenburg 45-1490* (MIN). **NUNAVUT. Anderson B.**: *Argus & Edlund 12725* (CAN). **Cambridge Bay**: *Argus & Edlund 12608*, *12609*, *12610*, *12613*, *12678*, *12679*, *12680*, *12681*, *12682*, *12683*, *12893* (CAN), *Bennett et al. 13-0192* (ALA, CAN, chars, od, UBC), *Calder et al. 24204* (DAO), *Consaul & Gillespie 1102*, *1103*, *1104*, *1105*, *1138*, *1139* (CAN), *Gillespie et al. 8500* (ALA, CAN, MT, O, UBC), *Gould s.n.* (ALA), *Porsild 21611* (CAN), *Polunin s.n.* (CAN, 2 sheets), *Stephens 1086* (CAN), *1285* (KANU, KSTC), *829*, *871* (CAN, KANU, KSTC), *849* (CAN, KSTC). **Falaise B.**: *Eriksen et al. 969* (ALA). **Ferguson L. [Tahiryuaq**]: *Hainault 2020* (CAN). **Greiner L.**: *Ponomarenko VI-237B* (CAN). **Ekalluk R.**: *Argus & Edlund 12739* (CAN). **Hadley B.**: *Edlund 54* (CAN), *Gould s.n.* (ALA). **Johansen B.**: *Gillespie et al. 7852* (ALA, CAN, MT, O), *7853*, *7854* (ALA, CAN, O), *7940* (ALA, CAN, MT, O, UBC), *7963* (ALA, CAN, MT, O), *8169* (CAN, O). **Jonnessee L.**: *Argus & Edlund 12778* (CAN). **Mt. Bumpus**: *Edlund 154*, *212* (CAN). **Mt. Lady Pelly [Amaaqtuq**]: *Jones & Hainault 1898* (CAN, DAO). **Ovayok TP**: *Stephens 862*, *868* (CAN, KANU, KSTC). **Namaycush L.**: *Argus & Edlund 12833* (CAN). **Oterkvik Pt.**: *Gillespie et al. 7481*, *7501* (CAN, O), *7500* (CAN), *7513* (ALA, BABY, CAN, MT, O, UBC), *7514* (ALA, CAN, MT, O). **Prince Albert S. (head)**: *Argus & Edlund 12825* (CAN). **Sinclair Cr.**: *Gillespie et al. 8295* (CAN), *8348* (CAN, O). **Surrey L.**: *Argus & Edlund 12803* (CAN). **Washburn L.**: *Oldenburg 46-2172* (MIN). **Wollaston P. (SW)**: *Edlund 525*, *526* (CAN). **Wollaston P.**: *D. Jenness 308E* (*279*) (CAN).

***Salix arctica* × *S. polaris***, Fig. 44B

Argus (2010) reported this hybrid from Victoria I., based on a subset of the collections reported here, none of which has been previously published. Aiken et al. (2007) did not mention it, and Argus (2007) did not map it. Hybrid plants are widespread across the island, with records from Anderson B., Boot Inl., Cambridge Bay, Collinson P., Ferguson L., Johansen B., Kuujjua R., Mt. Pelly, Namaycush L., Prince Albert Sound (head) and Ulukhaktok. Morphological characteristics of hybrid plants, which are not included in the key here, are described in Argus (2010).

**NORTHWEST TERRITORIES. Boot Inl.**: *Gillespie et al. 9614*, *8615* (CAN). **Kuujjua R.**: *Gillespie et al. 9758* (CAN). **Prince Albert S. (head)**: *Edlund 379* (CAN). **Ulukhaktok**: *Edlund 723* (CAN), *Porsild 17271* (CAN). **NUNAVUT. Anderson B.**: *Argus & Edlund 12722* (CAN). **Cambridge Bay**: *Argus & Edlund 12676*, *12865*, *12883*, *12885*, *12886*, *12887* (CAN), *Consaul & Gillespie 1130* (CAN), *Stephens 1088* (CAN). **Collinson P.**: *Argus & Edlund 12751* (CAN). **Ferguson L. [Tahiryuaq**]: *Argus & Edlund 12789* (CAN). **Johansen B.**: *Gillespie et al. 8094*, *8095b* (CAN, O). **Ovayok TP**: *Stephens 1167* (CAN, KSTC). **Namaycush L.**: *Argus & Edlund 12830*, *12831*, *12832*, *12835* (CAN).

### Linaceae [1/1]

***Linum* L. [1]**

**Figure 47.**
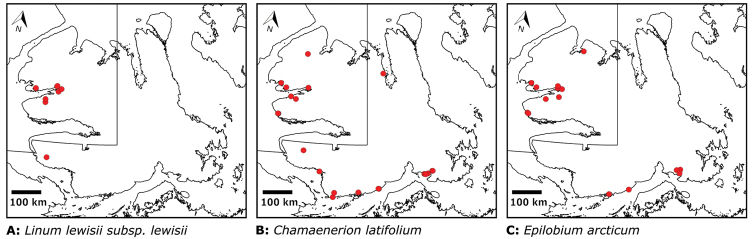
Species distribution maps. Linaceae: **A**Linum lewisii subsp. lewisii. Onagraceae: **B***Chamaenerion latifolium***C***Epilobium arcticum*.

**Figure 48.**
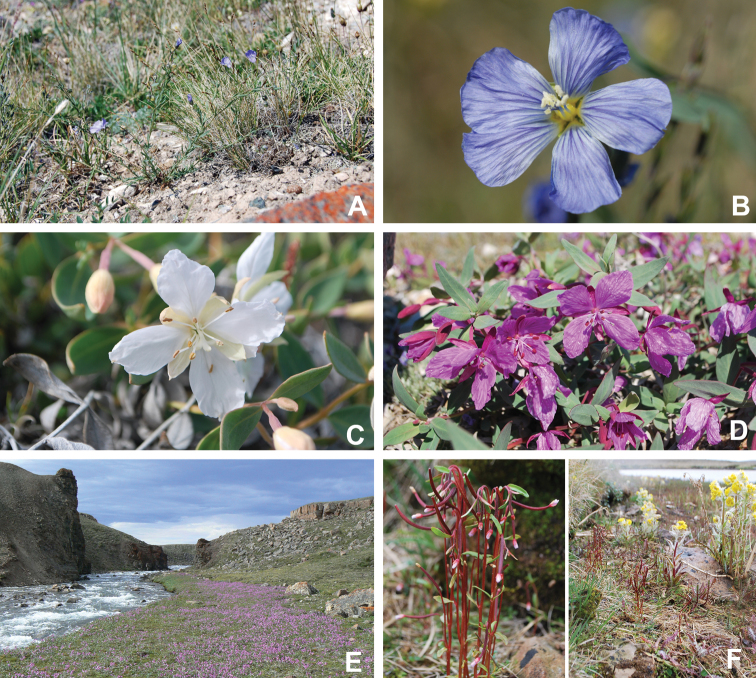
**A**Linum lewisii subsp. lewisii habit, *Gillespie et al. 9618***B**Linum lewisii subsp. lewisii flower, *Gillespie et al. 9618***C***Chamaenerion latifolium* inflorescence (white-flowered form), Minto Inlet, NT, 21 July 2010 **D***Chamaenerion latifolium* habit, Minto Inlet, NT, 21 July 2010 **E***Chamaenerion latifolium* habitat, Minto Inlet, NT, 21 July 2010 **F***Epilobium arcticum* habit (left) and habitat (right). Photos **A**, **B** by J.M. Saarela **C**, **D**, **F** by B.A. Bennett and **E** by P.C. Sokoloff.

***Linum lewisii*** Pursh subsp. ***lewisii***, Figs 47A, 48A, B–Lewis’s flax | North American (W)

Previously recorded from Kuujjua R. and the head of Minto Inl (Aiken et al. 2007). Newly recorded from the Hanerak area on southwestern Wollaston P. (see map in Jenness (1922)), where collected in 1955, and Boot Inl. Elsewhere in the Canadian Arctic recorded from western mainland sites east to Bathurst Inl. (Porsild and Cody 1980, Saarela et al. 2013, Saarela et al. 2017b). At Boot Inl. the taxon was growing on a south-facing slope with *Anemone parviflora*, *Arnica angustifolia*, *Dryas integrifolia* and Hedysarum boreale subsp. mackenziei (no. *9618*) and along the upper edge of a SE-facing cliff with *Carex myosuroides*, *Calamagrostis purpurascens*, *Oxytropis arctica*, *O. arctobia* and Poa glauca subsp. glauca (no. *9609*). In the Kuujjua R. area it grew on a stony dry hilltop and S-facing slope with *Artemisia hyperborea*, *C. purpurascens*, *O. arctica*, *O. arctobia*, Poa glauca subsp. glauca and *Saxifraga tricuspidata*. The three populations found at the head of Minto Inl. grew in similar habitats. At all sites, the taxon was infrequent. Elsewhere in the Canadian Arctic recorded from scattered mainland sites (Porsild and Cody 1980, Saarela et al. 2013, Saarela et al. 2017b).

**NORTHWEST TERRITORIES. Boot Inl.**: *Gillespie et al. 9609* (ALA, ari, CAN, MT, O, UBC, WIN), *Gillespie et al. 9618* (CAN). **Kuujjua R.**: *Edlund 630* (CAN), *Gillespie et al. 9730* (ALA, CAN, O). **Minto Inl. (head)**: *Gillespie et al. 10027* (CAN, O), *10098* (ALA, CAN, MT, O), *10265* (CAN, O), *Porsild 17417* (CAN). **NUNAVUT. Wollaston P.**: *Miller 201* (CAN).

### Myrtales

**Onagraceae [2/2]**

**Key to Onagraceae**

**Table:** 

1	Petals pink or pale purple, rarely white, 15–25 mm; sepals (11–)14–18 mm; capsules 45–70 mm; leaf blades (10–)25–45(–65) mm	***Chamaenerion latifolium***
–	Petals white or pale pink, 4–4.5 mm; sepals 3–3.5 mm; capsules 20–40 mm; leaf blades 4–10 mm	***Epilobium arcticum***

### *Chamaenerion* Ség. [1]

***Chamaenerion latifolium*** (L.) Sweet (*Chamerion latifolium* (L.) Holub, *Epilobium latifolium* L.), Figs 47B, 48C–E–River beauty | Circumpolar–alpine

Previously recorded from Byron B., Cambridge Bay, Hadley B., the head of Minto Inl. (Porsild obs., conf.), Mt. Pelly, Richard Collinson Inl., Ulukhaktok and Wollaston P. (Macoun and Holm 1921, Porsild 1955, 1957, 1964, Porsild and Cody 1980, Aiken et al. 2007). Thannheiser et al. (2001) additionally recorded it from Johansen B. (conf.). Newly recorded from Boot Inl., Kuujjua R., Oterkvik Pt., Sinclair Cr. and Walker B. Elsewhere in the Canadian Arctic recorded from Air Force, Axel Heiberg, Baffin, Banks, Coats, Devon, Digges, Eglinton, Ellesmere, King William, Melville and Southampton islands and across the mainland (Porsild and Cody 1980, Cody et al. 1989, Korol 1992, Cody and Reading 2005, Saarela et al. 2013, Saarela et al. 2017b, Blondeau 2018).

**NORTHWEST TERRITORIES. Boot Inl.**: *Gillespie et al. 9507*, *9523* (CAN, O). **Byron B.**: *Dushenko* (UVIC). **Kuujjua R.**: *Dutilly 18830* (QFA), *Gillespie et al. 9821* (CAN, O). **Minto Inl. (head)**: *Edlund 46*, *606* (CAN). **Richard Collinson Inl.**: *Edlund 201* (CAN). **Ulukhaktok**: *Dutilly 18654* (QFA), *Edlund 306* (CAN), *Oldenburg 42-49*, *45-1548* (CAN), *Porsild 17319* (CAN), *Ross 18A* (GH), *Saarela & Bull 1454* (ALA, ari, CAN, MT). **Walker B.**: *Oldenburg 45-1504* (CAN). **NUNAVUT. Cambridge Bay**: *Bennett et al. 13-0214* (UBC, V), *13-0310* (BABY, chars), *Edlund & Argus 12686* (CAN), *Stephens 1272* (CAN, KANU, KSTC). **Hadley B.**: *Edlund 71* (CAN). **Johansen B.**: *Gillespie et al. 7934* (ALA, CAN, MT, O, UBC). **Ovayok TP**: *Gillespie et al. 8427* (ALA, ALTA, BABY, CAN, MT, O, UBC), *Gould s.n.* (ALA), *Stephens 1173* (CAN, KSTC). **Oterkvik Pt.**: *Gillespie et al. 7598* (ALA, CAN, O), *7640* (ALA, ALTA, CAN, MT, O, UBC, UBC, US). **Read I.**: *Oldenburg 43-1064*, *43-908* (CAN), *Ross 29A* (GH). **Sinclair Cr.**: *Gillespie et al. 8355* (ALA, CAN, MT, O). **Wollaston P.**: *D. Jenness 575* (CAN).

### *Epilobium* L. [1]

***Epilobium arcticum*** Sam., Figs 47C, 48F–Arctic willowherb | Nearly circumpolar

Previously recorded from Cambridge Bay, Kuujjua R., the head of Minto Inl., Richard Collinson Inl. and Ulukhaktok (Porsild 1955, 1957, 1964, Porsild and Cody 1980, Aiken et al. 2007). Thannheiser et al. (2001) additionally recorded it from Johansen B. (conf.), Richardson I. and Surrey L. Newly recorded from Boot Inl., Sinclair Cr. and Walker B. Elsewhere in the Canadian Arctic recorded from Axel Heiberg, Baffin, Banks, Prince of Wales and Southampton islands and scattered mainland sites (Porsild and Cody 1980, Cody et al. 2003, Saarela et al. 2017b, Blondeau 2018).

**NORTHWEST TERRITORIES. Boot Inl.**: *Gillespie et al. 9672* (CAN, O). **Kuujjua R.**: *Edlund 680* (CAN), *Gillespie et al. 10004* (CAN). **Minto Inl. (head)**: *Edlund 105* (CAN), *Gillespie et al. 10040* (ari, CAN), *10200* (ALA, CAN, O), *Porsild 17318*, *17320*, *17412* (CAN). **Richard Collinson Inl.**: *Edlund 543* (CAN). **Ulukhaktok**: *Edlund 478* (CAN). **Walker B.**: *Oldenburg 45-1505* (CAN, GH). **NUNAVUT. Cambridge Bay**: *Stephens 1150* (CAN, KANU, KSTC). **Johansen B.**: *Gillespie et al. 8010* (ALA, CAN, MT, O). **Greiner L.**: *Ponomarenko VI-103*, *VI-314A* (CAN). **Sinclair Cr.**: *Gillespie et al. 8286* (ALA, CAN, O).

### Brassicales

**Brassicaceae [10/31/33]**

**Key to Brassicaceae [adapted from Porsild and Cody (1980), Aiken et al. (2007) and Al-Shehbaz (2010a)]:**

**Table:** 

1	Fruits silicles, less than 3 times long as wide	**2**
–	Fruits siliques, 3 or more times long as wide	**6**
2	Trichomes absent or present and simple	**3**
–	Trichomes present, branched	**4**
3	Basal leaves round, fleshy; silicles obovoid, ovoid or ellipsoid	*** Cochlearia ***
–	Basal leaves linear-oblanceolate to narrowly oblanceolate; silicles narrowly oblong to linear-lanceolate, often torulose	*** Parrya ***
4	Silicles globose, glabrous; petals yellow; plants silvery-canescent	*** Physaria ***
–	Silicles flattened to terete, hirsute (rarely glabrous); petals white, yellow or purple; plants glabrous to hirsute but not silvery-canescent	**5**
5	Petals white or yellow; siliques flattened	*** Draba ***
–	Petals white to purplish; siliques terete to ovoid	*** Braya ***
6	Trichomes absent or present and simple	**7**
–	Trichomes (at least some) branched	**9**
7	Siliques flattened, linear	*** Cardamine ***
–	Siliques terete or quadrangular in cross section, linear or torulose	**8**
8	Petals (8–)10–20 mm, purple to white; sepals (3–)4–8 mm; siliques 3–7 mm wide, sometimes torulose	*** Parrya ***
–	Petals 3–5 mm, white; sepals 1.5–3 mm; siliques 2–3 mm wide, not torulose	*** Eutrema ***
9	Basal and lower cauline leaves pinnate to bi-pinnately compound, blade margins deeply lobed	*** Descurainia ***
–	Basal and lower cauline leaves simple, blade margins entire to repand-dentate	**10**
10	Cauline leaf blade bases sagittate	*** Crucihimalaya ***
–	Cauline leaf blade bases cuneate, attenuate or absent	**11**
11	Trichomes sessile, malpighiaceous (with ends oriented along the long axis of the organ) and 3–5-rayed stellate, simple trichomes absent; petals (8–)10–20 mm	*** Erysimum ***
–	Trichomes stalked, cruciform, dendritic, stellate, submalpighiaceous or forked, simple trichomes sometimes present; petals 2–6(–8) mm	**12**
12	Branched trichomes submalpighiaceous or 2-forked; fruits terete to quadrangular in cross section; petals white, sometimes tinged pinkish or purplish	*** Braya ***
–	Branched trichomes mostly dendritic or stellate; fruits flattened in cross section; petals yellow or white	*** Draba ***

### *Braya* Sternberg & Hoppe [3/5]

**Key to *Braya* [adapted from Harris (2010)]:**

**Table:** 

1	Plants not scapose; cauline leaves (1–)2–4; fruits linear	***B. humilis***
–	Plants scapose; cauline leaves 0–1 (or with leaflike bract subtending proximalmost pedicel); fruits ovoid, globose, oval-elliptic, oblong-cylindrical or lanceoloid	**2**
2	Fruits ovoid or globose, (4–)5–8(–10) × (2.5–)3–5 mm (*B. thorild-wulffii*)	**3**
–	Fruits ovoid-elliptic, oblong-elliptic, oblong or narrowly oblong-lanceoloid, (3–)5–12(–15) × (0.8–)1.1–3(–3.6) mm (*B. glabella*)	**4**
3	Stems, pedicels, sepals, and fruits densely pubescent	**B. thorild-wulffii subsp. thorild-wulffii**
–	Stems, pedicels, sepals, and fruits glabrous or glabrescent	**B. thorild-wulffii subsp. glabrata**
4	Fruits oblong or narrowly oblong-lanceoloid, often curved, 3.5–8.3× as long as wide; racemes often loosely elongated in fruit	**B. glabella subsp. glabella**
–	Fruits ovoid-elliptic or oblong-elliptic, usually straight, 2.5–3.7× as long as wide; racemes not elongated in fruit, often compact	**B. glabella subsp. purpurascens**


**Figure 49.**
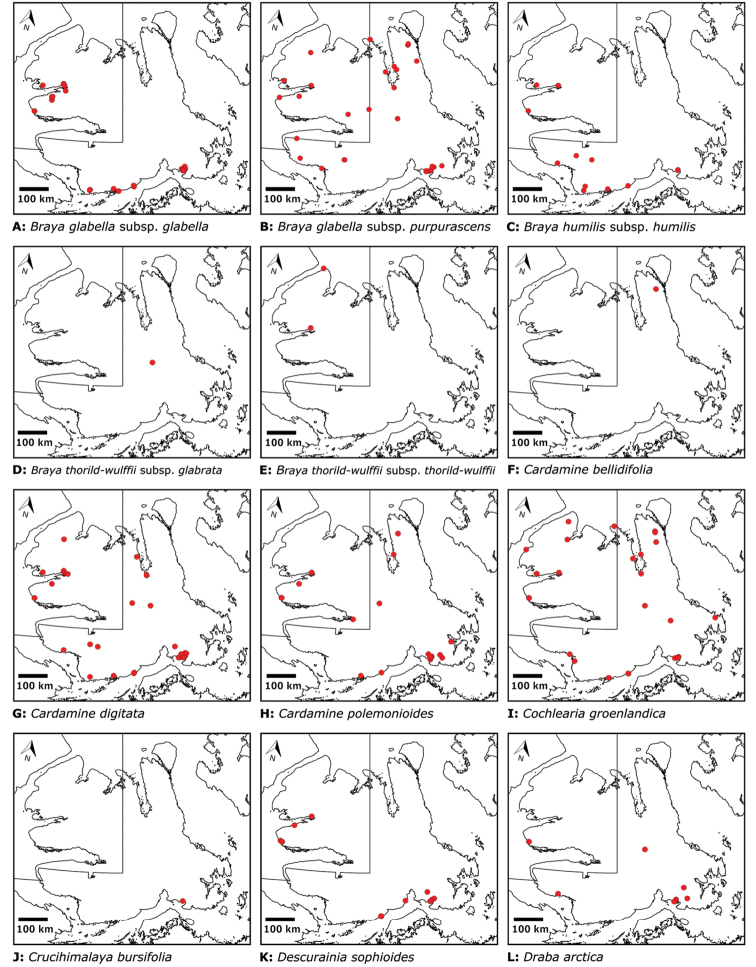
Species distribution maps. Brassicaceae: **A**Braya glabella subsp. glabella**B**Braya glabella subsp. purpurascens**C**Braya humilis subsp. humilis**D**Braya thorild-wulffii subsp. glabrata**E**Braya thorild-wulffii subsp. thorild-wulffii**F***Cardamine bellidifolia***G***Cardamine digitata***H***Cardamine polemonioides***I***Cochlearia groenlandica***J***Crucihimalaya bursifolia***K***Descurainia sophioides***L***Draba arctica*.

**Figure 50.**
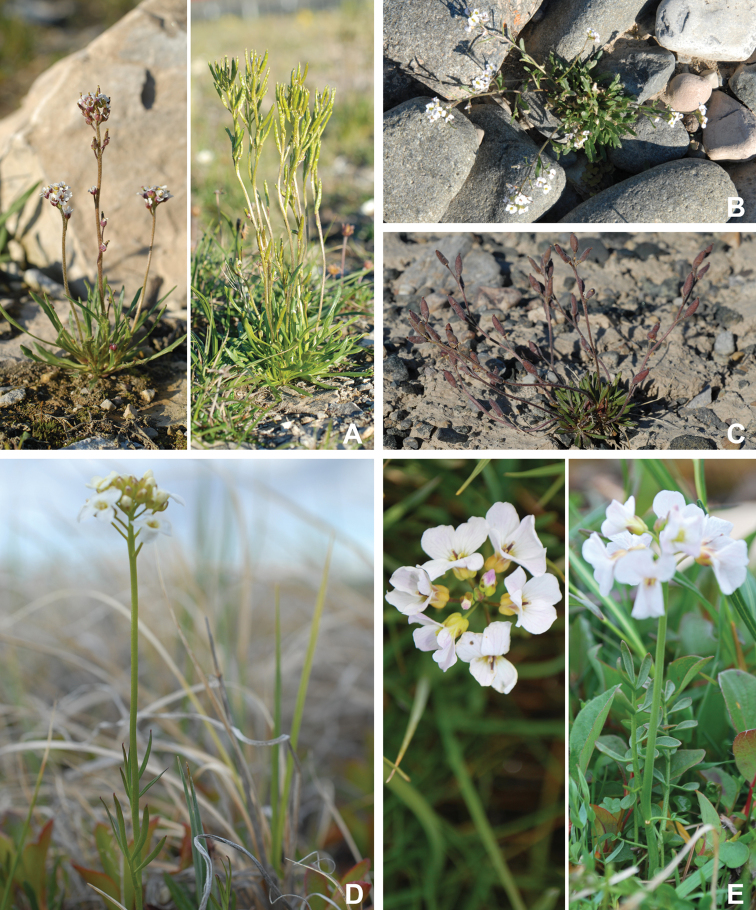
**A**Braya glabella subsp. glabella habit (left, *Gillespie et al. 8493*; right, *Gillespie et al. 8492*) **B**Braya humilis subsp. humilis habit, *Gillespie et al. 10170***C**Braya thorild-wulffii subsp. thorild-wulffii habit, *Gillespie et al. 10074***D***Cardamine digitata* habit, *Gillespie et al. 7498***E***Cardamine polemonioides* inflorescence (left) and habit (right). Photos **A–C** by L.J. Gillespie **D** by R.D. Bull and **E** by B.A. Bennett.

***Braya glabella*** Richardson subsp. ***glabella***, Figs 49A, 50A–Smooth northern rockcress | Amphi-Beringian–North American (N)

Previously recorded from Cambridge Bay, Kuujjua R., the head of Minto Inl. and Ulukhaktok (Porsild and Cody 1980, Harris 1985, Aiken et al. 2007). Newly recorded from Boot Inl., Greiner L., Johansen B., Murray Pt., Oterkvik Pt. and Sinclair Cr. Elsewhere in the Canadian Arctic recorded from Baffin, Banks and Southampton islands and mainland sites (Porsild and Cody 1980, Cody and Reading 2005, Aiken et al. 2007, Saarela et al. 2013, Saarela et al. 2017b, Garneau and Sabourin 2018). Three collections of the species at DAO were unavailable for determination to subspecies and are not mapped: Prince Albert S. (head): *Weerstra 1* (DAO); Cambridge Bay: *Calder et al.* (DAO); Ferguson L. [Tahiryuaq]: *Hainault 2090* (DAO).

**NORTHWEST TERRITORIES. Boot Inl.**: *Gillespie et al. 9586* (ALA, CAN, O). **Kuujjua R.**: *Edlund 622*, *657* (CAN). **Kuujjua R.**: *Gillespie et al. 9761* (CAN, O), *9844* (CAN). **Minto Inl. (head)**: *Edlund 67* (CAN), *Gillespie et al. 10075* (CAN, O), *10178* (ALA, ari, CAN, O), *10252* (CAN), *9475* (ALA, CAN, O). **Ulukhaktok**: *Edlund 298*, *495* (CAN), *Oldenburg 45-1690* (GH), *Porsild 17290* (CAN), *Saarela & Bull 1480* (CAN). **NUNAVUT. Cambridge Bay**: *Bennett et al. 13-0165* (chars, od), *13-0195* (ALA, BABY), *14-0341* (ALA, UBC), *14-0383* (DAO), *Dutilly & Duman 37162* (CAN), *Edlund & Argus 12655*, *12700* (CAN). *Gillespie et al. 8416* (CAN), *8492* (ALA, CAN, MO O), *8493* (CAN, O), *Oldenburg 44-900* (GH), *Porsild 21619*, *216200* (CAN), *Stephens 1038* (CAN, KSTC), *Stephens 1051*, *1069*, *1118*, *1137* (CAN, KSTC), *Washburn 24* (CAN, GH). **Greiner L.**: *Ponomarenko VI-289* (CAN). **Johansen B.**: *Gillespie et al. 7888* (CAN, MO). **Murray Pt.**: *Gillespie et al. 8182* (CAN). **Oterkvik Pt.**: *Gillespie et al. 7472*, *7560* (CAN, MO), *7489* (CAN, O). **Sinclair Cr.**: *Gillespie et al. 8263* (CAN), *8329* (CAN, O).

***Braya glabella*** subsp. ***purpurascens*** (R.Br.) Cody (*B. purpurascens* R.Br.), Fig. 49B–Purple rockcress | Circumpolar–Cordilleran

Previously recorded from Cambridge Bay, C. Wollaston, Hadley B., the head of Minto Inl., Natkusiak, Storkerson P., Read I., Ulukhaktok, Walker B. and Wollaston P. (Porsild 1955, 1957, 1964, Porsild and Cody 1980, Harris 1985, Aiken et al. 2007). Newly recorded from south of Burns L., Kuujjua R. (*Edlund 622*, mapped from here by Aiken et al. 2007, has been redetermined as subsp. glabella), Prince Albert P. and Richard Collinson Inl. Widespread across the Canadian Arctic Archipelago (Aiken et al. 2007). Some Northwest Territories mainland specimens that were published as this subspecies have been re-determined as subsp. glabella; there are few confirmed records of subsp. purpurascens from mainland Nunavut (Saarela et al. 2013). In their synopsis of *Braya*, Al-Shehbaz and German (2014) recognize this taxon at species level.

**NORTHWEST TERRITORIES. Burns L. (S)**: *Edlund 57* (CAN). **C. Wollaston**: *Edlund 53* (CAN). **Kuujjua R.**: *Gillespie et al. 9837* (CAN, O). **Minto Inl. (head)**: *Edlund 122* (CAN). **Natkusiak P.**: *Edlund 90*, *127* (CAN). **Prince Albert P.**: *Oldenburg 54-237* (GH), **Richard Collinson Inl.**: *Edlund 148* (CAN). **Tahiryuaq**: *Edlund 385* (CAN). **Walker B.**: *Porsild 17493A*, *17493B* (CAN). **Wollaston P. (NW)**: *Porsild 17218* (CAN). **NUNAVUT. Cambridge Bay**: *Bennett 13-0612* (CAN), *13-0318* (chars, BABY), *Beschel 13481* (CAN), *Gould s.n.* (ALA), *Polunin s.n.* (CAN), *Ponomarenko VI-056*, *VI-063*, *VI-094* (CAN), *Stephens 1014* (KSTC), *950*, *1034* (CAN, KSTC). **Greiner L.**: *Ponomarenko VI-167*, *VI-296B* (CAN). **Hadley B.**: *Edlund 13*, *101*, *337* (CAN). **Mt. Bumpus**: *Edlund 204*, *227* (CAN). **Namaycush L.**: *Edlund 37*, *40*, *43*, *148* (CAN), *Edlund & Roncato-Spencer 45* (CAN). **Read I.**: *Porsild 17195* (CAN). **Storkerson P.**: *Edlund 181*, *185*, *215*, *222*, *300*, *326* (CAN). **Wollaston P.**: *D. Jenness 413* (CAN).

***Braya humilis*** (C.A.Mey.) B.L.Rob. subsp. ***humilis*** (*B. richardsonii* (Rydb.) Fernald, B. humilis subsp. arctica (Böcher) Rollins), Figs 49C, 50B–Low rockcress | Asian (N/C)–amphi-Beringian–North American (N)

Previously recorded from Cambridge Bay, Mt. Bumpus, Ulukhaktok and Wollaston P. (Macoun and Holm 1921, Porsild 1955, Porsild and Cody 1980, Harris 1985, Aiken et al. 2007). Thannheiser et al. (2001) additionally recorded it from Johansen B. (conf.) and Richardson I. Elsewhere in the Canadian Arctic recorded from Ellesmere, Banks and Eglinton islands and mainland sites as far east as the Bathurst Inl. area (Harris 1985, Cody and Reading 2005, Aiken et al. 2007, Saarela et al. 2013, Saarela et al. 2017b).

**NORTHWEST TERRITORIES. Boot Inl.**: *Gillespie et al. 9571* (CAN, O). **Minto Inl. (head)**: *Edlund 122* (CAN), *Gillespie et al. 10170* (CAN). **Ulukhaktok**: *Edlund 862* (CAN), *Porsild 17291*, *17292* (ALTA, CAN), *Oldenburg 45-1688* (GH). **NUNAVUT**. **Cambridge Bay**: *Bennett et al. 13-0264* (BABY, chars, DAO, MO, UBC), *14-0304* (UAAH), *Gillespie et al. 8413* (CAN). **Falaise B.**: *Eriksen et al. 985* (ALA). **Johansen B.**: *Gillespie et al. 7887* (ALA, CAN, MO, O). **Mt. Bumpus**: *Edlund 220* (CAN). **Oterkvik Pt.**: *Gillespie et al. 7561*, *7562* (CAN), *7663* (CAN, MO). **Sinclair Cr.**: *Gillespie et al. 8224* (CAN, MO, O), *8319* (ALA, CAN, MO, O). **Wollaston P.**: *D. Jenness 411* (CAN).

***Braya thorild-wulffii*** subsp. ***glabrata*** J.G.Harris, Fig. 49D–Smooth Greenland rockcress | North American (N)

Known from a single collection from Namaycush L., which marks the known eastern and southern limit of the subspecies. Elsewhere in the Canadian Arctic recorded from Banks I. (Harris 2006). The taxon is endemic to the Canadian Arctic Archipelago. In their synopsis of *Braya*, Al-Shehbaz and German (2014) do not recognize infraspecic taxa in *B. thorild-wulffii*.

**NUNAVUT. Namaycush L.**: *Stretton 18* (DAO).

***Braya thorild-wulffii*** Ostenf. subsp. ***thorild-wulffii***, Figs 49E, 50C–Greenland rockcress | Amphi-Beringian–North American (N)

Previously recorded from Namaycush L., Peel Pt. and the north side of Prince Albert S. (Aiken et al. 2007). The single Namaycush L. record of the species has been redetermined as subsp. glabrata (Harris 2006). Newly recorded from the head of Minto Inl. Collections mapped on the Storkerson P. by Aiken et al. (2007) (*Edlund 215*, *222*) have been redetermined as Braya glabella subsp. purpurascens, and we have not seen a voucher for the record from the north shore of Prince Albert S. Elsewhere in the Canadian Arctic recorded from Banks I. and the Queen Elizabeth Islands (Aiken et al. 2007).

**NORTHWEST TERRITORIES. Minto Inl. (head)**: *Gillespie et al. 10074* (ALA, ari, CAN, O). **Peel Pt.**: *Edlund 424* (CAN).

### *Cardamine* L. [3]

**Key to *Cardamine* [adapted from Al-Shehbaz et al. (2010a)]:**

**Table:** 

1	Cauline leaves simple or absent; fruiting pedicels 3–6(–8) mm; petals 4–5.5(–7) mm, not clawed	***C. bellidifolia***
–	Cauline leaves pinnately compound, 3–25-foliolate, sometimes pinnatisect and appearing compound; fruiting pedicels 5–25(–8) mm; petals 5–12.3 mm, clawed	**2**
2	Cauline leaves (7–)9–21-foliolate or -pinnatisect; petals white-lilac, 9–12.3 mm; fruiting pedicels 5–15 mm; fruits 1–1.8 cm; rhizomes absent	***C. polemonioides***
–	Cauline leaves 3–7-foliolate; petals white, 5–9 mm; fruiting pedicels (7–)10–25 mm; fruits (1.5–)2–4 cm; rhizomes present, cylindrical, slender	***C. digitata***

***Cardamine bellidifolia*** L., Fig. 49F–Alpine bittercress | Circumpolar-alpine

Known from a single collection from the Storkerson P. (Aiken et al. 2007). Elsewhere in the Canadian Arctic recorded from across most of the Canadian Arctic Archipelago, but on adjacent Banks I. known only from the northern part of the island, a pattern similar to that seen on Victoria I. (Aiken et al. 2007). Also known from several Arctic mainland sites (Porsild and Cody 1980, Cody et al. 1989, Korol 1992, Cody and Reading 2005, Saarela et al. 2017b)(Garneau and Sabourin 2018).

**Nunavut: Storkerson P.**: *Edlund 238* (CAN).

***Cardamine digitata*** Richardson, Figs 49G, 50D–Richardson’s bittercress | Amphi-Beringian-North American (NW)

Previously recorded from Byron B., Cambridge Bay, Ferguson L., Hadley B., the head of Minto Inl., Mt. Bumpus, Namaycush L., Richard Collinson Inl. and Ulukhaktok (Porsild 1955, Porsild and Cody 1980, Aiken et al. 2007). Thannheiser et al. (2001) additionally recorded it from Richardson I. and Surrey L. Newly recorded from Boot Inl., Colville Mts., Falaise B., Greiner L., Johansen B., Kuujjua R., Oterkvik Pt., Sinclair Cr. and Tuktu R. Elsewhere in the Canadian Arctic recorded from Banks, Prince Charles and Southampton islands (Aiken et al. 2007) and mainland sites east to Hudson Bay (Porsild and Cody 1980, Korol 1992, Saarela et al. 2017b).

**NORTHWEST TERRITORIES. Boot Inl.**: *Gillespie et al. 9579* (ALA, CAN, O). **Kuujjua R.**: *Gillespie et al. 9810* (CAN). **Minto Inl. (head)**: *Edlund 152*, *614*, *62* (CAN), *Gillespie et al. 10046* (ALA, ari, CAN), *10307* (CAN), *Porsild 17393* (CAN). **Richard Collinson Inl.**: *Edlund 141* (CAN). **Ulukhaktok**: *Edlund 352*, *878* (CAN), *Oldenburg 45-1687* (GH), *Porsild 17293* (CAN), *17294* (ALTA, CAN). **NUNAVUT. Byron B.**: *Dushenko 30 (UVIC*). **Cambridge Bay**: *Bennett et al. 13-0160* (CAN, chars, MO, od), *Calder et al.* (DAO), *Edlund & Argus 12625*, *12689* (CAN), *Gillespie et al. 8367* (CAN), *Polunin s.n.* (CAN), *Ponomarenko VI-055*, *VI-080E* (CAN), *Porsild 21621* (CAN), *Stephens 1017*, *1018*, *11965* (KSTC), *1027*, *1067* (CAN, KSTC), *Sweatman & Smith 38* (DAO). **Colville Mts.**: *Gillespie et al. 7766* (CAN). **Falaise B.**: *Eriksen et al. 956* (ALA). **Ferguson L. [Tahiryuaq**]: *Hainault* (DAO). **Greiner L.**: *Ponomarenko VI-050*, *VI-052b*, *VI-105b*, *VI-300*, *VI-329b*, *VI-341* (CAN). **Hadley B.**: *Edlund 41*, *156* (CAN). **Johansen B.**: *Gillespie et al. 7817* (CAN, MO, O). **Mt. Bumpus**: *Edlund 246* (CAN). **Namaycush L.**: *Edlund & Roncato-Spencer 71* (CAN). **Oterkvik Pt.**: *Gillespie et al. 7498* (ALA, CAN, MT, O). **Sinclair Cr.**: *Gillespie et al. 8292* (CAN). **Tuktu R.**: *Gould s.n.* (ALA).

***Cardamine polemonioides*** Rouy (*C. nymanii* Gand., C. pratensis subsp. angustifolia (Hook.) O.E.Schultz), Figs 49H, 50E–Cuckoo flower, Meadow bittercress | Circumpolar

Previously recorded from Byron B., Cambridge Bay, Hadley B., the head of Minto Inl., the head of Prince Albert S., Storkerson P. and Ulukhaktok (Aiken et al. 2007). Thannheiser et al. (2001) additionally recorded it from Johansen B. (conf.). Newly recorded from Albert Edward B., Greiner L., Kuujjua R. and Tuktu R. Widespread throughout the Canadian Arctic (Porsild and Cody 1980, Korol 1992, Cody and Reading 2005, Aiken et al. 2007, Saarela et al. 2013, Saarela et al. 2017b, Garneau and Sabourin 2018).

**NORTHWEST TERRITORIES. Kuujjua R.**: *Gillespie et al. 9799* (CAN), **Minto Inl. (head)**: *Edlund 107*, *603*, *604* (CAN), *Gillespie et al. 10133* (CAN). **Prince Albert S. (head)**: *Porsild 17442* (CAN). **Ulukhaktok**: *Edlund 483*, *764* (CAN). **NUNAVUT. Albert Edward B.**: *Ponomarenko VI-258A* (CAN). **Byron B.**: *Dushenko 25* (UVIC). **Cambridge Bay**: *Bennett 13-0229* (BABY, chars, od), *Calder et al. 24181* (DAO), *Consaul & Gillespie 1123* (CAN), *Gillespie et al. 8366* (CAN, MO, O), *Oldenburg 44-950* (GH), *Polunin s.n.* (CAN), *Porsild 17468*, *21622* (CAN), *Stephens 1052*, *1093* (CAN), *1253* (KSTC). **Greiner L.**: *Ponomarenko VI-105*, *VI-137A*, *VI-203F* (CAN). **Hadley B.**: *Edlund 320* (CAN). **Johansen B.**: *Gillespie et al. 8045* (CAN, O). **Storkerson P.**: *Edlund 244* (CAN). **Tuktu R.**: *Gould s.n.* (ALA).

### *Cochlearia* L. [1]

**Figure 51.**
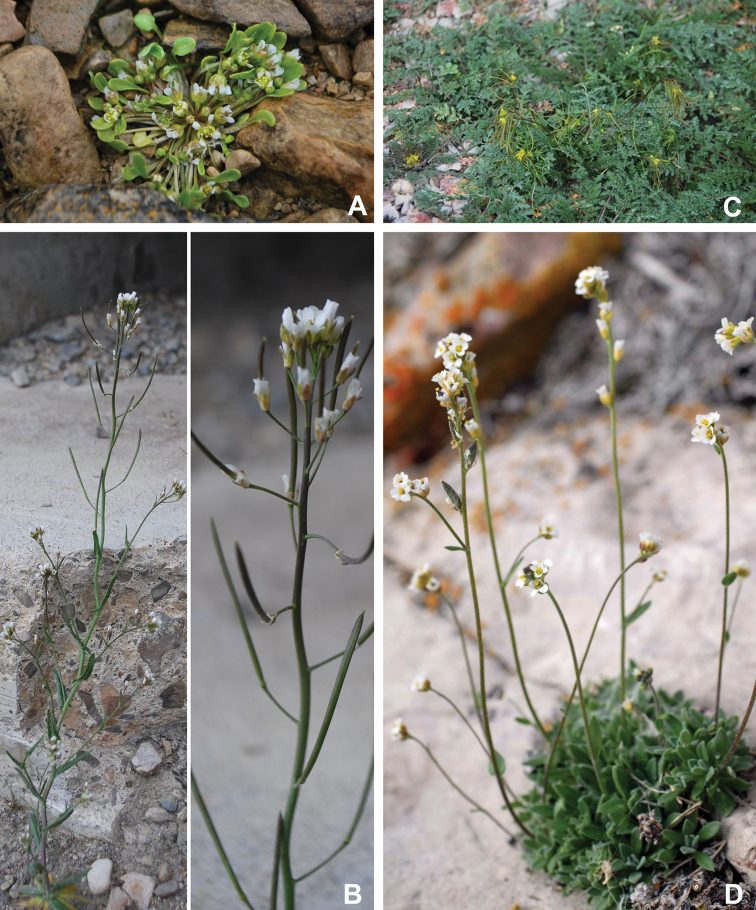
**A***Cochlearia groenlandica* habit **B***Crucihimalaya bursifolia* habit (left) and inflorescence and fruits (right) **C***Descurainia sophioides* habit, *Saarela & Teeter 5292***D***Draba cinerea* habit, *Gillespie et al 7531*. Photos **A**, **B** by B.A. Bennett, **C** by J.M. Saarela and **D** by R.D. Bull.

***Cochlearia groenlandica*** L. (C. officinalis subsp. arctica (Schltdl.) Hultén, C. officinalis subsp. groenlandica (L.) A.E.Porsild), Figs 49I, 51A–Greenland scurvygrass | Circumpolar

Previously recorded from Cambridge Bay, Collinson P., Gordon Pt., Hadley B., the head of Minto Inl., Namaycush L., Natkusiak P., the head of Prince Albert S. (Porsild obs.), Read I. (Porsild obs.; conf.), Richard Collinson Inl., Storkerson P., Ulukhaktok and an unnamed lake ca. 60 miles north of Cambridge Bay (Porsild 1955, 1957, 1964, Porsild and Cody 1980, Aiken et al. 2007). Thannheiser et al. (2001) additionally recorded it from Richardson I. Newly recorded from Clouston B., Johansen B., “Oldenburg L.” and Sinclair Cr., closing a conspicuous gap on southwestern Victoria I. Most sites on Victoria I. are near the coast. Elsewhere in the Canadian Arctic widespread across the Archipelago and mainland, primarily along the coast (Porsild and Cody 1980, Aiken et al. 2007, Saarela et al. 2013, Garneau and Sabourin 2018).

**NORTHWEST TERRITORIES. Gordon Pt.**: *Stretton 200* (DAO). **Minto Inl. (head)**: *Edlund 155* (CAN). **Natkusiak P.**: *Edlund 118* (CAN). “**Oldenburg L.**”: *Oldenburg 45-1352* (GH). **Richard Collinson Inl.**: *Edlund 694* (CAN). **Ulukhaktok**: *Edlund 346*, *795* (CAN), *Saarela & Bull 1451* (CAN, O). **NUNAVUT. Cambridge Bay**: *Bennett 14-0648* (DAO, UBC), *Bennett et al. 13-0248* (BABY, chars, UAAH), *13-0627* (od), *Calder et al. 24186* (DAO), *Gillespie et al. 8463* (ALA, CAN, O), *Oldenburg 44-902* (GH), *Polunin s.n.* (CAN), *Stephens 1155* (CAN, KSTC). **Clouston B.**: *Gillespie et al. 7738* (CAN). **Collinson P.**: *Edlund & Argus 12755* (CAN). **Hadley B.**: *Edlund 79*, *122*, *318* (CAN). **Johansen B.**: *Gillespie et al. 8009* (ALA, CAN, MT, O). **Namaycush L.**: *Edlund 121* (CAN). **Read I.**: *Oldenburg 42-529* (GH). **Sinclair Cr.**: *Gillespie et al. 8254*, *8285* (CAN). **Unnamed lake ca. 60 mi. N of Cambridge Bay**: *Porsild 17475* (CAN). **Storkerson P.**: *Edlund 166*, *210* (CAN), *Stretton 234* (DAO).

### *Crucihimalaya* Al-Shehbaz & O’Kane & R.A.Price [1]

***Crucihimalaya bursifolia*** (DC.) D.A.German & A.L.Ebel (*Halimolobos mollis* (Hook.) Rollins, *Transberingia bursifolia* (DC.) Al-Shehbaz *&* O’Kane), Figs 49J, 51B–Soft fissurewort | Asian (NE)–amphi-Beringian–North American (N)

Newly reported from Victoria I. based on a single collection from Cambridge Bay gathered in 2013 near the Defence Early Warning station. Taxonomy follows German (2005). Elsewhere in the Canadian Arctic recorded from Baffin, southern, Banks and Ellesmere islands (Aiken et al. 2007).

**NUNAVUT. Cambridge Bay**: *Bennett et al. 13-0325* (CAN).

### *Descurainia* Webb & Berthel. [1]

***Descurainia sophioides*** (Fisch. ex Hook.) O.E.Schulz, Figs 49K, 51C–Northern tansy mustard | Asian (N)–amphi-Beringian–North American (NW)

Previously recorded from Byron B., Cambridge Bay, Ferguson L., the head of Minto Inl. and Ulukhaktok (Porsild 1955, 1957, 1964, Porsild and Cody 1980, Aiken et al. 2007). Thannheiser et al. (2001) additionally recorded it from Johansen B. and Wellington B. Newly recorded from “30-Mile Cr.”, Kuujjua R. and Sinclair Cr. Elsewhere in the Canadian Arctic recorded from Banks I., a few sites on Baffin I. and mainland sites (Porsild and Cody 1980, Cody 1996a, Cody and Reading 2005, Aiken et al. 2007, Saarela et al. 2013, Saarela et al. 2017b). This species thrives in heavily disturbed areas and is common in Cambridge Bay.

**NORTHWEST TERRITORIES. Kuujjua R.**: *Gillespie et al. 9932* (ALA, CAN, O). **Minto Inl. (head)**: *Edlund 106* (CAN). **Ulukhaktok**: *Bliss s.n.* (ALTA), *Edlund 323* (CAN), *Gray & Gibbard 5*, *22*, *26* (DAO), *Oldenburg s.n.* (CAN), *Pokiak 30* (CAN, UBC), *Saarela & Bull 1413* (CAN). **NUNAVUT. “30-Mile Cr.**”: *Bennett et al. 14-0345* (UBC). **Byron B.**: *Dushenko 31* (UVIC). **Cambridge Bay**: *Bennett et al. 13-0173* (BABY, chars, od), *Calder et al. 24165* (DAO), *Edlund & Argus 12656* (CAN), *Fortier 27* (CAN), *Gould s.n.* (ALA), *Milne 42-804* (GH), *Oldenburg 44-943* (GH), *Parker & Jonsdottir 9090* (ALA), *Polunin s.n.* (CAN), *Porsild 17469*, *21623* (CAN), *Stephens 1202* (CAN, KSTC), *1286* (CAN, KANU, KSTC), *848* (KSTC), *933* (CAN, KANU, KSTC), *Sweatman & Smith 29* (CAN, DAO), *Saarela & Teeter 5287*, *5292*, *5293*, *5298* (CAN). **Ferguson L. [Tahiryuaq**]: *Jones & Hainault 38* (DAO). **Sinclair Cr.**: *Gillespie et al. 8310* (ALA, ALTA, BABY, CAN, MO, MT, O, UBC, US).

### *Draba* L. [16]

**Key to *Draba* [adapted from Al-Shehbaz et al. (2010b)]:**

Note: Species groups used in the *Draba* key in the Flora of North America (FNA) are referenced in square brackets. Several species key under more than one group.

**Table:** 

1	Cauline leaves of flowering stems 1+	**2**
–	Cauline leaves of flowering stems absent	**15**
2	Abaxial surface of leaf blades glabrous or with simple trichomes [FNA Group 2]	**3**
–	Abaxial surface of leaf blades with only branched trichomes	**5**
3	Abaxial surface of leaf blades glabrous	***D. lactea***
–	Abaxial surface of leaf blades pubescent	**4**
4	Leaf blade surfaces pubescent with simple and stalked, 2- or 3-rayed trichomes, and short-stalked, 8–12-rayed, stellate ones; fruit valves pubescent, trichomes (2–)5–12-rayed; style 0.2–0.8 mm; petals 3.5–5 × 1.5–2.5 mm; sepals 2–3 mm; stems proximally with branched trichomes 4–10-rayed	***D. oblongata***
–	Leaf blade surfaces abaxially pubescent or glabrous, trichomes simple, sometimes with fewer, short-stalked, 2-rayed ones, adaxially often glabrous; fruit valves glabrous; style 0.05–0.2(–0.3) mm; petals 2–3.5 × 0.8–1.5 mm; sepals 1.2–2.2 mm; stems glabrous or, rarely, sparsely pubescent, trichomes straight	***D. fladnizensis***
5	Fruit valves glabrous [FNA Group 3]	**6**
–	Fruit valves pubescent or puberulent (at least on margin)	**10**
6	Abaxial surface of leaf blades with some 7–15-rayed trichomes	**7**
–	Abaxial surface of leaf blades with 2–5(–6)-rayed trichomes	**9**
7	Margins of basal and cauline leaf blades entire	***D. nivalis***
–	Margins of basal and, sometimes, cauline leaf blades usually dentate or denticulate, if entire, racemes not flexuous in fruit	**8**
8	Cauline leaves usually 0, rarely 1 (as a bract); basal leaf blades each with prominent midvein; sepals glabrous or, rarely, with simple trichomes subapically; racemes 2–8(–12)-flowered	***D. lactea***
–	Cauline leaves usually 2–25; basal leaf blades each with obscure midvein; sepals with 2–5-rayed trichomes; racemes (5–)8–26(–34)-flowered	***D. glabella***
9	Abaxial surface of leaf blades with stalked, cruciform trichomes, adaxially with cruciform and/or simple and 2-rayed ones; sepals ovate, 2.2–3 mm, glabrous or pubescent subapically, trichomes simple, 2-rayed	***D. juvenilis***
–	Abaxial surface of leaf blades with stalked, (2–)4(–6)-rayed trichomes, adaxially glabrous or pubescent, with simple and stalked, 2-rayed trichomes; sepals 1.7–2.5 mm, pubescent, trichomes simple 2–4-rayed	***D. norvegica***
10	Abaxial surface of leaf blades with at least some 7–15-rayed trichomes [FNA Group 4]	**11**
–	Abaxial surface of leaf blades with 2–4(-6)-rayed trichomes [FNA Group 5]	**14**
11	Fruit trichomes simple and 2–5-rayed	***D. glabella***
–	Fruit trichomes 2–7-rayed (at least on replum)	**12**
12	Petals 2–3.5 × 0.8–1.4 mm; fruits 1.5–2.2 mm wide	***D. nivalis***
–	Petals 3.5–6 × 1.5–2 mm; fruits 2–3.5 mm wide	**13**
13	Basal leaf blades with simple trichomes apically, abaxial surfaces with distinct midveins; seeds 0.8–1.1 × (0.6–)0.7–0.8 mm	***D. arctica***
–	Basal leaf blades without simple trichomes apically, abaxial surfaces with obscure midveins; seeds 0.6–0.8 × 0.4–0.6 mm	***D. cinerea***
14	Fruit trichomes simple; adaxial surface of leaf blades with cruciform and/or simple and 2-rayed ones; sepals ovate, 2.2–3 mm, glabrous or pubescent subapically, trichomes simple and short-stalked, 2-rayed	***D. juvenilis***
–	Fruit trichomes simple and 2-rayed; adaxial surface of leaf blades glabrous or pubescent, with simple and stalked, 2-rayed trichomes; sepals 1.7–2.5 mm, pubescent, trichomes simple and short-stalked, 2–4-rayed	***D. norvegica***
15	Rachises glabrous	**16**
–	Rachises sparsely to densely pubescent	**20**
16	Abaxial surface of leaf blades glabrous (sometimes trichomes only on margins and apices) [FNA Group 6]	***D. lactea***
–	Abaxial surface of leaf blades pubescent [FNA Group 7]	**17**
17	Abaxial surface of leaf blades with some 7–16-rayed trichomes	***D. lactea***
–	Abaxial surface of leaf blades with simple and/or 2–5(–6)-rayed trichomes	**18**
18	Abaxial surface of leaf blades with cruciform trichomes; petals 3–5 × 1.5–2.5 mm; sepals 2.2–3 mm	***D. juvenilis***
–	Abaxial surface of leaf blades with mixture of simple and 2(–4)-rayed trichomes; petals 1.5–2.5 × 0.7–1.5 mm; sepals 1.2–2.2 mm	**19**
19	Fruits flattened, elliptic-lanceolate to oblong, 1.5–2 mm wide; rachises glabrous; petals 2–2.5 × 0.8–1.5 mm	***D. fladnizensis***
–	Fruits slightly inflated, ovoid to oblong, 2–3 mm wide; rachises usually pubescent, rarely glabrous; petals 1.5–2(–2.5) × 0.7–1 mm	***D. subcapitata***
20	Leaf blade margins not ciliate [FNA Group 8]	**21**
–	Leaf blade margins ciliate	**22**
21	Abaxial surface of leaf blades with pectinate trichomes; petals usually yellow, rarely creamy white; fruits 3–6(–7) mm, valves usually puberulent, rarely glabrous, trichomes simple and sessile, often unequally 2-rayed, ovules 6–12 per ovary	***D. oligosperma***
–	Abaxial surface of leaf blades with cruciform trichomes; petals pale yellow to creamy white; fruits 5–11(–14) mm, valves usually glabrous, rarely margins pubescent, trichomes simple, ovules 16–30 per ovary	***D. juvenilis***
22	Abaxial surface of leaf blades usually with simple or simple and branched trichomes, rarely glabrous [FNA Group 9]	**23**
–	Abaxial surface of leaf blades with branched trichomes [FNA Group 10]	**30**
23	Abaxial surface of leaf blades usually with simple and/or 2-rayed trichomes, rarely glabrous	***D. subcapitata***
–	Abaxial surface of leaf blades with simple and 2–6-rayed trichomes, sometimes subdendritic and up to 12-rayed	**24**
24	Racemes not or slightly elongated (sometimes subumbellate) in fruit	**25**
–	Racemes elongated in fruit	**27**
25	Basal leaves densely imbricate; petals white or creamy white; fruits 3–6 mm, inflated (at least basally)	***D. subcapitata***
–	Basal leaves not imbricate; petals pale yellow; fruits 5–10 mm, flattened	**26**
26	Fruits ovate-elliptic, 2–3.2 mm wide, valves often densely pubescent; ovules (16–)18–28 per ovary; leaf blades with apices obtuse to rounded, surfaces with subcruciform trichomes	***D. micropetala***
–	Fruits obovate, (3–)3.5–5 mm wide, valves often glabrate; ovules 8–16(–20) per ovary; leaf blades with apices acute or subacute, surfaces with simple and/or 2-branched trichomes	***D. pauciflora***
27	Petals white; fruit valves pubescent, trichomes (2–)5–12-rayed; stigmas distinctly wider than styles; abaxial surface of leaf blades with some 8–12-rayed trichomes	***D. oblongata***
–	Petals pale or bright yellow; fruit valves usually pubescent, sometimes glabrous, trichomes simple, spurred or 2-rayed; stigmas about as wide as styles; abaxial surface of leaf blades with some 2–5-rayed trichomes	**28**
28	Fruiting pedicels 1–3(–4) mm; petals 2–3 × (0.7–)1–1.5 mm; styles 0.05–0.3 mm; racemes slightly elongated in fruit	***D. micropetala***
–	Fruiting pedicels 2.5–10 mm; petals 3.5–6 × 2–3.8 mm; styles 0.1–0.9 mm; racemes often considerably elongated in fruit	**29**
29	Basal leaf blades linear to linear-oblanceolate, 1–2.5(–4) mm wide, midveins prominent; style 0.4–0.9 mm; petals obovate, 3.5–6 × 2–3.5 mm	***D. pilosa***
–	Basal leaf blades oblong, lanceolate, oblanceolate or obovate, 2–9 mm wide, midveins obscure; style 0.1–0.3 mm; petals narrowly obovate, (3.5–)3.8–5.5(–5.8) × (2.5–)2.8–4(–4.6) mm	***D. simmonsii***
30	Abaxial surface of leaf blades with pectinate, subdendritic or some 7–12-rayed, stellate trichomes	**31**
–	Abaxial surface of leaf blades with 2–6-rayed trichomes	**34**
31	Abaxial surface of leaf blades with pectinate trichomes	***D. oligosperma***
–	Abaxial surface with stellate or subdendritic trichomes	**32**
32	Fruit valves glabrous	***D. lactea***
–	Fruit valves pubescent, trichomes 2–6-rayed	**33**
33	Basal leaf blades apically with simple trichomes, midveins distinct abaxially; seeds 0.8–1.1 × (0.6–)0.7–0.8 mm	***D. arctica***
–	Basal leaf blades apically without simple trichomes, midveins obscure abaxially; seeds 0.6–0.8 × 0.4–0.6 mm	***D. cinerea***
34	Petals 4–6 × 3–5 mm; fruits 3.5–5.5 mm wide (stigmas distinctly wider than styles); racemes not elongated (corymbose) in fruit	***D. corymbosa***
–	Petals 2–3 × (0.7–)1–1.5 mm; fruits 2–3.2 mm wide (stigmas narrower than styles); racemes mostly elongated in fruit	***D. micropetala***

***Draba arctica*** J.Vahl, Fig. 49L–Arctic draba | probably amphi-Atlantic

Aiken et al. (2007) recorded a single collection, *Stephens 877*, from Cambridge Bay; we reidentified this collection as *D. cinerea*. Newly recorded from Cambridge Bay, Falaise B., Ferguson L., Greiner L. watershed, Namaycush L. and Ulukhaktok. Elsewhere in the Canadian Arctic recorded from Axel Heiberg, Baffin, Banks, Devon, King William, Melville and Southampton islands and Melville Peninsula (Aiken et al. 2007). The general status rank of this species in Northwest Territories is Sensitive (Working Group on General Status of NWT Species 2016); the taxon was not recognized in earlier status reports.

**NORTHWEST TERRITORIES. Ulukhaktok**: *Edlund 364* (CAN), *Porsild 17295*, *17296* (CAN). **NUNAVUT. Cambridge Bay**: *Bennett et al. 14-0647b* (BABY, chars, od), *Edlund & Argus 12633*. **Ferguson L. [Tahiryuaq**]: *Edlund & Argus 12777* (CAN). **Falaise B.**: *Eriksen et al. 987* (ALA). **Greiner L.**: *Ponomarenko VI-308A*, *VI-208A* (cf.) (CAN). **Namaycush L.**: *Edlund & Roncato-Spencer 52* (CAN).

**Figure 52.**
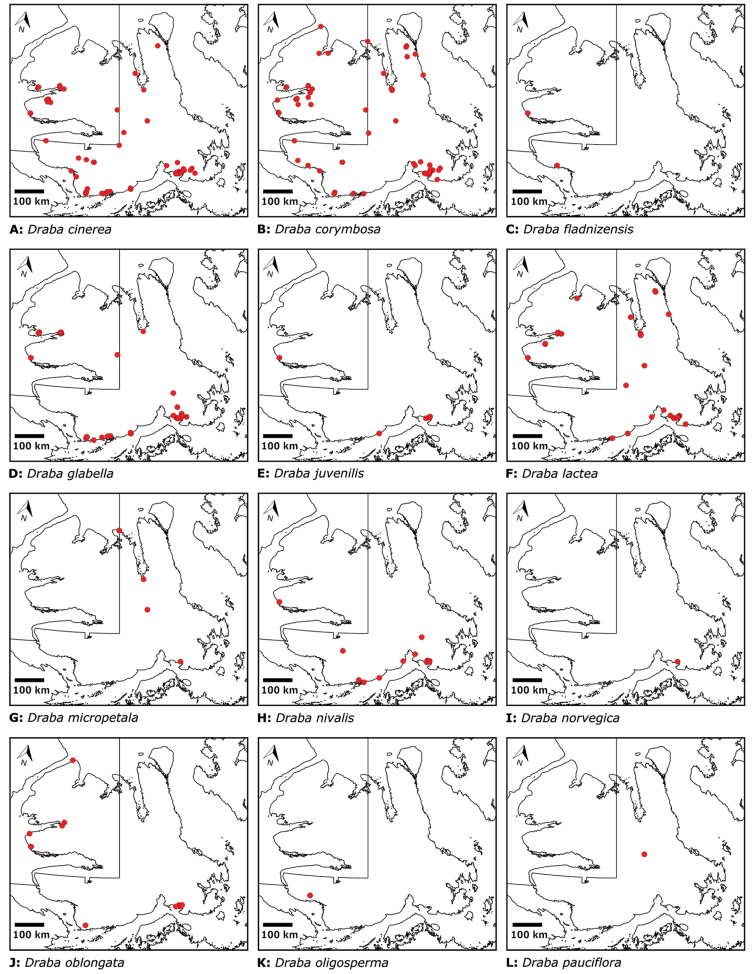
Species distribution maps. Brassicaceae: **A***Draba cinerea***B***Draba corymbosa***C***Draba fladnizensis***D***Draba glabella***E***Draba juvenilis***F***Draba lactea***G***Draba micropetala***H***Draba nivalis***I***Draba norvegica***J***Draba oblongata***K***Draba oligosperma***L***Draba pauciflora*.

***Draba cinerea*** Adams, Figs 52A, 51D–Greyleaf draba | Circumboreal-polar

Previously recorded from Boot Inl., Cambridge Bay, Ferguson L., Hadley B., Kuujjua R., the head of Prince Albert S., Read I., Storkerson P., Ulukhaktok and northwestern Wollaston P. (Porsild 1955, 1957, 1964, Porsild and Cody 1980, Aiken et al. 2007). Thannheiser et al. (2001) additionally recorded it from Johansen B. (conf.), Mt. Pelly (conf.), Surrey L. and Wellington B. Newly recorded from south of Burns L., Clouston B., Colville Mts., Diamond Jenness P., Ferguson L., Greiner L., Mt. Bumpus, Namaycush L. and Oterkvik Pt. This is the second most-collected species on Victoria I., known from over 100 collections. Elsewhere in the Canadian Arctic widespread on both the islands and mainland (Porsild and Cody 1980, Aiken et al. 2007, Saarela et al. 2013, Saarela et al. 2017b).

**NORTHWEST TERRITORIES. Boot Inl.**: *Dutilly 18727* (DAO), *Gillespie et al. 9532*, *9569* (CAN, O), *9686* (CAN). **Burns L. (S)**: *Edlund 549* (CAN). **Kuujjua R.**: *Edlund 664* (CAN), *Gillespie et al. 9870c*, *9898* (CAN, O), *9890*, *9996*, *9997*, *9970* (CAN). **Minto Inl. (head)**: *Gillespie et al. 10019* (ALA, CAN, O), *9469*, *10081*, *10171* (CAN), *10122b* (CAN, O), *Porsild 17395* (ALA, CAN), *17396* (CAN). **Ulukhaktok**: *Edlund 361*, *713*, *779* (CAN), *Gray & Gibbard 12 & 19*, *44* (DAO) *Porsild 17302* (CAN), *Saarela & Bull 1415* (ALA, CAN, O), *1425*, *1479* (CAN, O), *1487* (CAN). **Wollaston P. (NW)**: *Porsild 17221*, *17223* (CAN). **NUNAVUT. Cambridge Bay**: *Bennett et al. 13-0212* (BABY, DAO), *13-0254* (chars, DAO), *13-0567*, *14-0662* (DAO), *13-0625*, *14-0312* (MO), *13-0638*, *14-0647a* (MO, DAO), *13-0639* (MO, UAAH, UBC), *14-0546* (UAAH), *Calder et al. 24211A* (DAO), *Edlund & Argus 12648* (CAN), *Gillespie et al. 8448*, *8465* (CAN), *Polunin s.n.* (CAN), *Ponomarenko VI-061*, *VI-065*, *VI-081b* (CAN), *Porsild 21624*, *21627* (CAN), *Smith & Sweatman 36* (DAO), *Stephens 1011*, *1115* (CAN), *877*, *1013*, *1070*, *1116*, *1117* (CAN, KSTC), *Tasker 2989* (CAN). **Clouston B.**: *Gillespie et al. 7732* (CAN, MO, O). **Colville Mts.**: *Gillespie et al. 7771* (CAN). **Diamond Jenness P.**: *Edlund & Argus 7503*, *7504* (CAN). **Ferguson L. [Tahiryuaq**]: *Bennett et al. 14-0667* (MO), *Hainault 2119* (DAO). **Greiner L.**: *Ponomarenko VI-200*, *VI-156*, *VI*-327 (CAN). **Hadley B.**: *Edlund 21*, *107* (CAN). **Johansen B.**: *Gillespie et al. 7827* (ALA, CAN, MO, MT, O), *7889* (ALA, CAN, MO, O), *7890*, 7894, *8078b*, *8165* (CAN), *7893*, *8054*, *8129*, *8163* (CAN, mixed with *D. glabella*, O), *8106* (CAN, MO, O). **Mt. Bumpus**: *Edlund 149*, *205*, *226* (CAN). **Ovayok TP**: *Gillespie et al. 8428* (CAN, MO). **Namaycush L.**: *Edlund 5*, *6* (CAN), *Roncato-Spencer 3* (CAN). **Oterkvik Pt.**: *Gillespie et al. 7531*, *7657* (ALA, CAN, MO, MT, O, UBC), *7536* (CAN, O), *7576b*, *7585*, *7588*, *7593*, *7659*, *7660*, *7664*, *7801b* (CAN), *7592* (ALA, CAN, MO, O). **Prince Albert S. (head)**: *Edlund & Argus 12818* (CAN), *Edlund 21*, *89*, *99* (CAN). **Read I.**: *Porsild 17196* (CAN). **Sinclair Cr.**: *Gillespie et al. 8225a*, *8264* (CAN, MO, O), *8281* (CAN, O), *8316* (BABY, CAN, O), *8328* (CAN). **Storkerson P.**: *Edlund 208* (CAN). **Wollaston P.**: *D. Jenness 652* (CAN).

**Figure 53.**
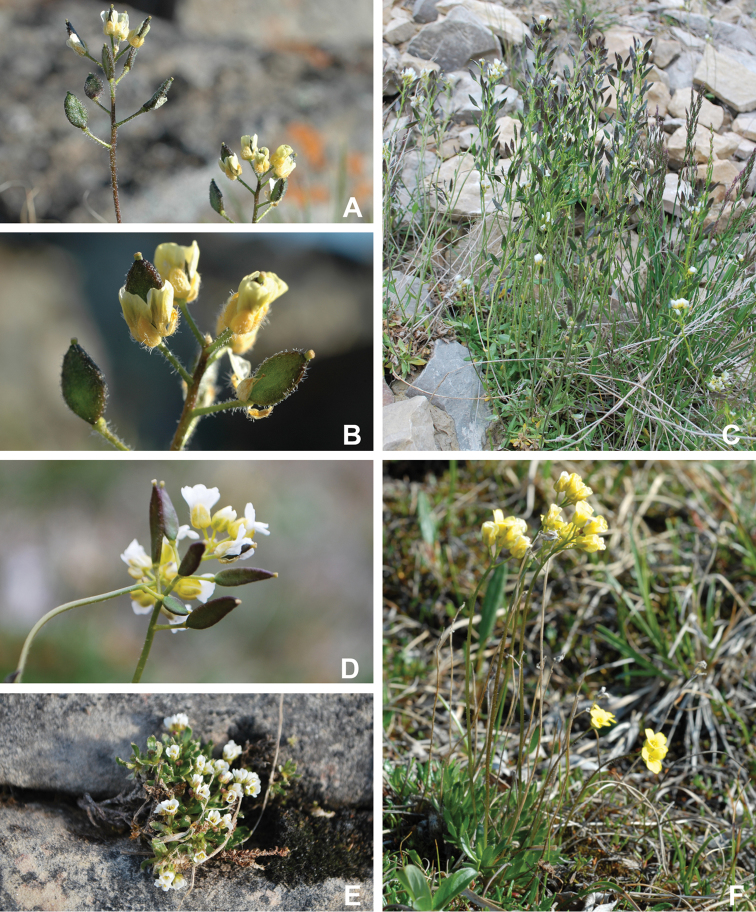
**A***Draba corymbosa* habit, *Gillespie et al. 9994***B***Draba corymbosa* developing fruits, *Gillespie et al. 9994***C***Draba glabella* habit **D***Draba glabella* inflorescence and fruits **E***Draba lactea* habit **F***Draba pilosa* habit, *Gillespie et al. 9679.* Photos **A**, **B** by R.D. Bull **C–E** by B.A. Bennett and **F** by L.J. Gillespie.

***Draba corymbosa*** R.Br. ex DC. (*D. bellii* Holm), Figs 52B, 53A, B–Flat-top draba | Circumpolar

Previously recorded from Anderson B., south of Burns L., Cambridge Bay, C. Wollaston, Greely Haven, Hadley B., Kuujjua R., the head of Minto Inl., Mt. Bumpus, Namaycush L., Peel Pt., east of the head of Prince Albert S., Read I., Richard Collinson Inl., Ulukhaktok and Wollaston P. (Porsild 1955, 1957, 1964, Porsild and Cody 1980, Aiken et al. 2007). Thannheiser et al. (2001) additionally recorded it from Johansen B. (conf.) and Surrey L. Newly recorded from Boot Inl., Falaise B., Ferguson L., Greiner L., Mt. Lady Pelly, Mt. Pelly, Murray Pt., Natkusiak P. and Oterkvik Pt. Widespread across the Canadian Arctic, and particularly common in the western and northern Arctic islands (Porsild and Cody 1980, Aiken et al. 2007, Garneau and Sabourin 2018).

**NORTHWEST TERRITORIES. Boot Inl.**: *Gillespie et al. 9685*, *9701* (cf.), *9702*, *9703*, *9704*, *9705* (cf.), *9706*, *9707* (CAN), *9687* (CAN, O). **Burns L. (S)**: *Edlund 551* (CAN). **C. Wollaston**: *Edlund 37a* (CAN). **Kuujjua R.**: *Edlund 679* (CAN), *Gillespie et al. 9834*, *9870a*, *9994* (CAN), *9843* (ALA, CAN, MT, O), *9949* (ALA, CAN, O), *Stretton 56* (DAO). **Minto Inl. (head)**: *Gillespie et al. 9470*, *10012*, *10261C* (CAN), *10058* (ALA, CAN, O), *10253* (ALA, CAN, MT, O), *Porsild 17394* (CAN). **Natkusiak P.**: *Edlund 94* (CAN). **Peel Pt.**: *Edlund 430* (CAN, mixed with *D. oblongata*). **Prince Albert S. (head)**: *Stretton 32* (DAO). **Richard Collinson Inl.**: *P. Jenness 19* (CAN), *Stretton 217* (DAO). **Ulukhaktok**: *Edlund 465* (CAN), *Gray & Gibbard 18* (DAO), *Ross 196* (ALTA). **Wollaston P. (NW)**: *Porsild 17219*, *17220* (CAN). **NUNAVUT. Anderson B.**: *Edlund & Argus 12703* (CAN). **Cambridge Bay**: *Dutilly 28068* (DAO), *Bennett et al. 13-0610* (BABY, DAO), *13-0650*, *13-0651* (DAO), *13-0659* (CAN), *Calder et al. 24211B* (DAO), *Consaul & Gillespie 1119* (CAN), *Dutilly 28068*, *28069* (DAO), *Edlund & Argus 12647* (CAN), *Gillespie et al. 8459* (CAN, MO, O), *Polunin s.n.* (CAN), *Ponomarenko VI-057B* (CAN), *Porsild 21624* (CAN, KSTC), *Saarela & Teeter 5291* (CAN), *Scotter s.n.* (ALTA), *Stephens 852*, *875*, *978*, *1016*, *1071*, *1269* (CAN, KSTC), *853*, *1267* (KSTC), *Sweatman & Smith 8* (DAO), *Tasker 2989* (CAN). **Falaise B.**: *Eriksen et al. 944* (ALA). **Ferguson L. [Tahiryuaq**]: *Bennett et al. 14-0405* (MO), *Hainault 2072* (DAO), *Jones 9* (DAO). **Greely Haven**: *Fortier 95* (CAN). **Greiner L.**: *Ponomarenko VI-031A*, *VI-093*, *VI-271*, *VI-283*, *VI-309A*, *VI-311* (CAN). **Hadley B.**: *Edlund 12*, *508*, *96*, *128*, *150* (CAN). **Johansen B.**: *Gillespie et al. 8078a* (CAN, MO). **Mt. Bumpus**: *Edlund 147*, *266* (CAN). **Mt. Lady Pelly [Amaaqtuq**]: *Hainault 1831* (DAO). **Ovayok TP**: *Gillespie et al. 8422* (ALA, CAN, MOO), *Bennett & Sullivan 13-0652* (DAO). **Murray Pt.**: *Gillespie et al. 8179* (ALA, CAN, MOO), *8185* (CAN, O). **Namaycush L.**: *Edlund 20*, *25* (CAN), *Edlund & Roncato-Spencer 24*, *47* (CAN). **Oterkvik Pt.**: *Gillespie et al. 7471* (ALA, CAN, MO, O), *7497a*, *7573* (CAN, MO, O). **Read I.**: *Porsild 17197* (CAN). **Storkerson P.**: *Edlund 182*, *200*, *216*, *221*, *282* (CAN), *Stretton* 225 (DAO). **Wollaston P.**: *D. Jenness 651* (CAN).

***Draba fladnizensis*** Wulfen, Fig. 52C–Austrian draba | Circumpolar-alpine

Newly recorded for Victoria I., where known from Falaise B. and Ulukhaktok. The collection from the latter site was taken by Porsild in 1949, mixed with *D. lactea*. Elsewhere in the Canadian Arctic recorded from Baffin, Banks, Cornwallis and Southampton islands and scattered mainland sites (Mulligan 1974, Porsild and Cody 1980, Korol 1992, Cody and Reading 2005, Saarela et al. 2013, Saarela et al. 2017b). This species was assessed as Sensitive by the Working Group on General Status of NWT Species (2011) but the status was revised to Secure by Working Group on General Status of NWT Species (2016) in light of new information on the species in the territory.

**NORTHWEST TERRITORIES. Ulukhaktok**: *Porsild 17298* (CAN, mixed with *D. lactea*). **NUNAVUT. Falaise B.**: *Parker 91 10* (ALA).

***Draba glabella*** Pursh, Figs 52D, 53C, D–Smooth draba | Circumboreal-polar

Previously recorded from south of Burns L., Cambridge Bay, “Long L.”, the head of Minto Inl. and Ulukhaktok (Porsild 1955, 1957, 1964, Porsild and Cody 1980, Aiken et al. 2007). Thannheiser et al. (2001) additionally recorded it from Johansen B. (conf.), Richardson I. and Surrey L. Newly recorded from Boot Inl., Ferguson L., Greiner L., Hadley B., Oterkvik Pt., Sinclair Cr. and “Trunsky L.” Widespread throughout the Canadian Arctic, but with a conspicuous gap in the Central Canadian Arctic islands (Porsild and Cody 1980, Cody et al. 1989, Cody et al. 2003, Aiken et al. 2007, Saarela et al. 2013, Sokoloff 2015, Saarela et al. 2017b). Elsewhere in the Canadian Arctic recorded from Axel Heiberg, Baffin, Banks, Bylot, Coats, Digges, Eglinton, Ellesmere, Igloolik, Melville and Southampton islands and across the mainland (Porsild and Cody 1980, Cody et al. 1989, Cody et al. 2003, Saarela et al. 2013, Saarela et al. 2017b, Garneau and Sabourin 2018).

**NORTHWEST TERRITORIES. Boot Inl.**: *Gillespie et al. 9570*, *9621* (CAN, O). **Burns L. (S)**: *Edlund 550* (CAN). **Minto Inl. (head)**: *Edlund 79*, *80A*, *616*, *618* (CAN), *Gillespie et al. 10161*, *10122a*, *10167* (cf.) (CAN, O), *10166* (ALA, ari, CAN, MT, O), *10172*, *10182*, *10214* (CAN). **Ulukhaktok**: *Porsild 17300* (CAN). **NUNAVUT. Cambridge Bay**: *Bennett 14-0306* (UAAH), *13-0175* (BABY, chars, DAO), *13-0654*, *14-0376* (MO), *13-0224a* (cf.) (MO, UBC), *13-0648* (cf.) (chars, DAO, UBC), *Calder et al. s.n.* (DAO), *Edlund & Argus 12649* (CAN, mixed with *D. juvenilis* and *D. lactea*), *2861* (CAN), *Fortier 26* (CAN), *Gillespie et al. 8447*, *8452a* (CAN, MO, O), *8471* (CAN), *Parker & Jonsdottir 9092* (ALA), *Polunin s.n.* (CAN), *Porsild 21626* (CAN), *Stephens 979*, *1011* (CAN, KSTC), *1013*, *1120* (CAN), *1076*, *1261* (KSTC), *Washburn* 25 (CAN, GH). **Ferguson L. [Tahiryuaq**]: *Hainault 2116* (DAO). **Greiner L.**: *Ponomarenko VI-039*, *VI-044* (CAN). **Hadley B.**: *Edlund 100* (CAN). **Johansen B.**: *Gillespie et al. 7826*, *7829*, *8144*, *8163* (CAN, mixed with *D. cinerea*), *7892* (CAN, O), *8041* (ALA, CAN, MO, MT, O), *8042* (CAN, MO). “**Long L.**”: *Lambert s.n.* (CAN). **Oterkvik Pt.**: *Gillespie et al. 7537*, *7538*, *7704*, *7801a*, *7810* (CAN), *7576a* (CAN, MO, O). **Sinclair Cr.**: *Gillespie et al. 8255* (CAN), *8314* (CAN, O). “**Trunsky L.**”: *Bennett et al. 14-0671* (MO).

***Draba juvenilis*** Kom. (*D. longipes* Raup), Fig. 52E–Long stalked draba | Asian (NE)–amphi-Beringian–Cordilleran

Newly recorded for Victoria I., from Cambridge Bay, Sinclair Cr. and Ulukhaktok. We have also confirmed the first record from adjacent Banks I. (Sachs Harbour, *Lambert s.n.*, CAN 529469); this specimen was previously determined as D. ? oblongata and *D. glabella*. These are the first records for the Canadian Arctic Archipelago. In the Cambridge Bay area, the species has been collected at the following four sites: 4 km northeast of Cambridge Bay along the road to Mt. Pelly (*Bennett 13-0224b*), where it grew around edge of nutrient enriched tundra pond with *Cardamine polemonioides*, *Epilobium arcticum*, *Chrysosplenium rosendahlii*, *Hippuris lanceolata* and *Tephroseris palustris*; ca. 2 km northeast of Cambridge Bay along the road to Mt. Pelly, just over the bridge, growing in small rocky outcrops above the river (*Gillespie et al. 3499*, *8400*); east of the of DEW Line Station along the road to the village (*Edlund & Argus 12649*, a mixed collection of three species); and on Long Point beach, between Long Point and Flagstaff Point ca. 10 km west of Cambridge Bay (*Gillespie et al. 8945b*).

**NORTHWEST TERRITORIES. Ulukhaktok**: *Saarela & Bull 1463* (ALA, CAN, MT, O). **NUNAVUT. Cambridge Bay**: *Bennett 13-0224b* (DAO, MO), *Edlund & Argus 12649* (CAN, mixed with *D. lactea* and *D. glabella*), *Gillespie et al. 8399*, *8452b* (CAN), *8400* (CAN, MO, O). **Sinclair Cr.**: *Gillespie et al. 8274* (CAN).

***Draba lactea*** Adams, Figs 52F, 53E–Milky draba | Circumpolar

Previously recorded from Anderson B., Cambridge Bay, Hadley B., the head of Minto Inl., Richard Collinson Inl., Storkerson P., Tahoe L. and Ulukhaktok (Porsild 1955, 1957, 1964, Porsild and Cody 1980, Aiken et al. 2007). Thannheiser et al. (2001) additionally recorded it from Richardson I. Newly recorded from “30-Mile Cr.”, Ferguson L., Greiner L., Kuujjua R., Namaycush L. and Sinclair Cr. Widespread across the Arctic (Mulligan 1974, Porsild and Cody 1980, Aiken et al. 2007, Garneau and Sabourin 2018).

**NORTHWEST TERRITORIES. Kuujjua R.**: *Gillespie et al. 9755*, *9756b*, *9870b* (CAN). **Minto Inl. (head)**: *Edlund 617*, *80B*, *81*, 96 (CAN), *Gillespie et al. 10213* (ALA, ari, CAN, MT, O), *Porsild 17398*, *17399* (CAN). **Richard Collinson Inl.**: *Stretton 196*, *215* (DAO). **Ulukhaktok**: *Edlund 797* (CAN), *Porsild 17297*, *17298*, mixed with *D. fladnizensis*, *17299* (CAN). **NUNAVUT. “30-Mile Cr.**”: *Bennett et al. 14-0665* (MO). **Anderson B.**: *Edlund & Argus 12714* (CAN). **Cambridge Bay**: *Bennett et al. 13-0216*, *13-0647* (BABY, DAO), *13-0658* (DAO), *13-0643* (chars), *Calder et al. s.n.* (DAO, 2 sheets), *Edlund & Argus 12649* (CAN, mixed with *D. juvenilis* and *D. glabella*), *12859* (CAN), *Gillespie 5840* (CAN), *Polunin s.n.* (CAN), *Stephens 1012* (KSTC), *876*, *1012B*, *1015*, *1075*, *1268* (CAN, KSTC), *Sweatman & Smith* (DAO). **Ferguson L. [Tahiryuaq**]: *Bennett et al. 14-0668* (MO). **Greiner L.**: *Ponomarenko VI-308* (CAN). **Hadley B.**: *Edlund 118*, *151*, *42*, *97* (CAN). **Murray Pt.**: *Gillespie et al. 8178* (CAN, mixed with *D. nivalis*, MO, O), *8180* (CAN, O). **Namaycush L.**: *Roncato-Spencer 3b* (CAN). **NE Victoria I.**: *Edlund 231* (CAN). **Sinclair Cr.**: *Gillespie et al. 8273*, *8275*, *8278*, *8279* (CAN). **Storkerson P.**: *Edlund 196*, *308*, *309* (CAN). **Tahoe L.**: *Porsild 17455A* (CAN).

***Draba micropetala*** Hook., Fig. 52G–Small-flowered draba | Circumpolar

Previously recorded from Cambridge Bay and Hadley B. (Aiken et al. 2007) and newly reported from Natkusiak P. and Namaycush L. This is primarily a high Arctic taxon, known in Canada from Axel Heiberg, Baffin, Banks, Bathurst, Cornwallis, Devon, Ellef Ringnes, Ellesmere, Igloolik, King Christian, King William, Lougheed, Meighen, Melville, Prince of Wales, Prince Patrick, Somerset and Ward Hunt islands, and a few northern mainland sites (Aiken et al. 2007, Garneau and Sabourin 2018).

**NORTHWEST TERRITORIES. Natkusiak P.**: *Edlund 93*, *103*, *114* (CAN). **NUNAVUT. Cambridge Bay**: *Calder et al. 24209* (aff.) (CAN). **Hadley B.**: *Edlund 149* (CAN). **Namaycush L.**: *Edlund 12* (CAN).

***Draba nivalis*** Lilj., Fig. 52H–Snow draba | Circumpolar-alpine

Previously recorded from Cambridge Bay and Ulukhaktok (Porsild 1955, 1957, 1964, Porsild and Cody 1980, Aiken et al. 2007). Thannheiser et al. (2001) additionally recorded it from Johansen B. (conf.), Richardson I., Surrey L. and Wellington B. Newly recorded from “30 Mile-Cr.”, Ferguson L., Mt. Bumpus, Murray Pt., Sinclair Cr. and “Trunsky L.” Elsewhere in the Canadian Arctic recorded from Axel Heiberg, Baffin, Banks, Bylot, Coats, Devon, Digges, Ellesmere, Gilmore, King William, Nottingham, Somerset and Southampton islands and across the mainland (Mulligan 1974, Porsild and Cody 1980, Cody et al. 1989, Korol 1992, Cody and Reading 2005, Aiken et al. 2007, Saarela et al. 2013, Saarela et al. 2017b, Garneau and Sabourin 2018).

**NORTHWEST TERRITORIES. Ulukhaktok**: *Edlund 895* (CAN), *Porsild 17301* (CAN). **NUNAVUT. “30-Mile Cr.**”: *Bennett et al. 14-0666* (MO). **Cambridge Bay**: *Bennett et al. 13-0642* (DAO), *14-0313*, *14-0317* (MO), *Polunin s.n.* (CAN, 2 sheets), *Porsild 21628* (CAN), *Stephens 1012A* (CAN), *1119* (CAN, KSTC). **Ferguson L. [Tahiryuaq**]: *Bennett et al. 14-0669* (MO). **Johansen B.**: *Gillespie et al. 7825*, *8043* (CAN). **Mt. Bumpus**: *Edlund 151* (CAN). **Murray Pt.**: *Gillespie et al. 8178* (CAN, mixed with *D. glabella*). **Sinclair Cr.**: *Gillespie et al. 8225b* (CAN). “**Trunsky L.**”: *Bennett et al. 14-0391* (MO).

***Draba norvegica*** Gunn. (*D. rupestris* W.T.Aiton), Fig. 52I–Norway draba | Amphi-Atlantic

Newly recorded from Victoria I., based on a single collection from Cambridge Bay (Hudson Bay Post) determined by G.A. Mulligan in 1997 and conf. by R. Elven in 2003. In Aiken et al. (2007), only a single unconfirmed record of this species from Southampton I., based on the map in Porsild and Cody (1980), was mapped for the Canadian Arctic Archipelago. The species was not recorded as occurring in Nunavut in Al-Shehbaz et al. (2010b). Elsewhere in the Canadian Arctic recorded from the Belcher Islands (e.g., *Consaul et al. 3506* [CAN 10055172], *3512* [CAN 10055171]) and northern Quebec and Labrador (Garneau and Sabourin 2018). Elsewhere in western Canada known only from a disjunct site north of the east arm of Great Slave L. (Mulligan and Cody 1968, Porsild and Cody 1980). There are taxonomic problems with this species (Elven et al. 2011). The general status rank of this species in Northwest Territories is Undetermined (Working Group on General Status of NWT Species 2016).

**NUNAVUT. Cambridge Bay**: *Calder et al. s.n.* (DAO).

***Draba oblongata*** R.Br. ex DC. (*D. groenlandica* Ekman), Fig. 52J–Canada arctic draba | Circumpolar?

Previously recorded from Cambridge Bay, C. Wollaston, the head of Minto Inl. and Ulukhaktok (Porsild and Cody 1980, Aiken et al. 2007), as well as from the head of Prince Albert S. (Mulligan 1971, Porsild and Cody 1980) and the Burns L. area (Aiken et al. 2007), sites for which we have not seen vouchers. The record *Jenness 652* from Wollaston P., mapped as this species in Aiken et al. (2007) and originally published as *D. nivalis* (Macoun and Holm 1921), has been re-determined as *D. cinerea*. Thannheiser et al. (2001) additionally recorded it from Mt. Pelly and Read I. Newly recorded from Colville Mts., Greiner L. and Peel Pt. Elsewhere in the Canadian Arctic recorded from Axel Heiberg, Baffin, Banks, Bathurst, Bylot, Coats, Cornwallis, Devon, Ellef Ringnes, Ellesmere, King William, Melville, Prince Charles, Prince Patrick, Prince of Wales, Somerset and Southampton islands (Mulligan 1971, Porsild and Cody 1980, Aiken et al. 2007). On the mainland Canadian Arctic known only from Boothia and Melville peninsulas (Aiken et al. 2007) and Bernard Harbour [*Johansen 312a*, CAN 10055408, det. R. Elven, 2003, published as *D. corymbosa* (Macoun and Holm 1921)].

**NORTHWEST TERRITORIES. C. Wollaston**: *Edlund 37b* (CAN). **Minto Inl. (head)**: *Gillespie et al. 10276* (CAN, O), *Porsild 17397* (CAN). **Peel Pt.**: *Edlund 430* (CAN, mixed with *D. corymbosa*). **Ulukhaktok**: *Gray & Gibbard 25* (DAO), *Edlund 798* (CAN). **NUNAVUT. Cambridge Bay**: *Bennett et al. 14-0318* (MO), *Beschel 13491* (CAN), *Calder et al. 24210* (DAO), *Ponomarenko VI-058* (CAN). **Colville Mts.**: *Gillespie et al. 7783* (CAN, MO). **Greiner L.**: *Ponomarenko VI-024* (CAN).

***Draba oligosperma*** Hook., Fig. 52K–Few-seed draba | Cordilleran

Known from two collections in the vicinity of Falaise B. on the south side of Wollaston P. (Elven et al. 2011), as mapped in Aiken et al. (2007). These are the only records of the species for the Canadian Arctic Archipelago. Elsewhere in the Canadian Arctic recorded from mainland Northwest Territories (Saarela et al. 2013).

**NUNAVUT. Falaise B.**: *Eriksen et al. 983* (ALA, CAN), *988* (O).

**Figure 54.**
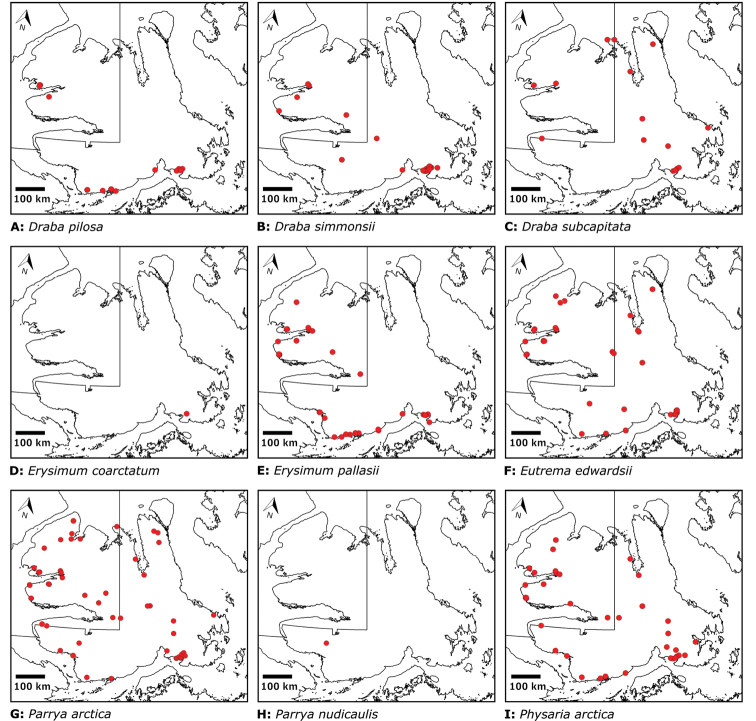
Species distribution maps. Brassicaceae: **A***Draba pilosa***B***Draba simmonsii***C***Draba subcapitata***D***Erysimum coarctatum***E***Erysimum pallasii***F***Eutrema edwardsii***G***Parrya arctica***H***Parrya nudicaulis***I***Physaria arctica*.

***Draba pauciflora*** R.Br., Fig. 54L–Few-flowered draba | Circumpolar

Known on Victoria I. from a single collection from the vicinity of Namaycush L., as mapped in Aiken et al. (2007). This collection marks the known southern limit of this high Arctic taxon, also known from Axel Heiberg, Banks, Bathurst, Devon, Ellef Ringnes, Ellesmere, Meighen, Melville, Prince Patrick and Somerset islands (Aiken et al. 2007). The general status rank of this species in Northwest Territories is Sensitive (Working Group on General Status of NWT Species 2016); there are no records from the Northwest Territories portion of Victoria I.

**NUNAVUT. Namaycush L.**: *Roncato-Spencer 8* (CAN).

***Draba pilosa*** Adams ex DC., Figs 54A, 53F–Pilose draba | Asian (N)–amphi-Beringian

Newly recorded for Victoria I., where known from “30-Mile Cr.”, Boot Inl., Cambridge Bay, Johansen B., Kuujjua R., Murray Pt. and Oterkvik Pt. Not known from elsewhere in the Canadian Arctic Archipelago. These collections represent a considerable range extension northwards. Elsewhere in the Canadian Arctic recorded from a few mainland sites (Porsild and Cody 1980, Saarela et al. 2013, Saarela et al. 2017b, Garneau and Sabourin 2018).

**NORTHWEST TERRITORIES. Boot Inl.**: *Gillespie et al. 9575*, *9683* (CAN), *9637* (cf.) (CAN, O), *9679* (ALA, CAN, O). **Kuujjua R.**: *Gillespie et al. 9754* (cf.) (ALA, CAN, O). **NUNAVUT. “30-Mile Cr.**”: *Bennett et al. 14-0664* (MO). **Cambridge Bay**: *Bennett et al. 13-0239* (chars, MO), *13-0259* (BABY, DAO, MO), *13-0649* (DAO), *14-0319* (DAO, UAAH), *14-0384* (UAAH), *Stephens 1010* (CAN). **Johansen B.**: *Gillespie et al. 7937* (CAN, MO), *7960* (CAN, MO, O), *8135* (CAN). **Murray Pt.**: *Gillespie et al. 8183* (CAN). **Oterkvik Pt.**: *Gillespie et al. 7497b*, *7523* (CAN).

**Figure 55.**
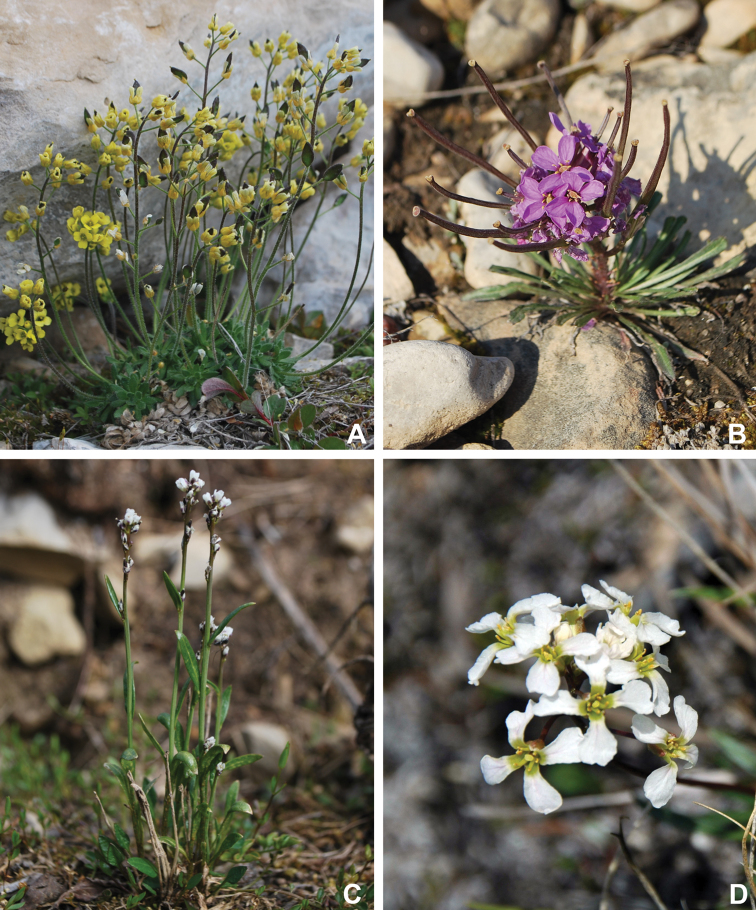
**A***Draba simmonsii*, habit **B***Erysimum pallasii*, habit **C***Eutrema edwardsii* habit **D***Parrya arctica* inflorescence, *Gillespie et al. 7488*. Photos **A–C** by B.A. Bennett and **D** by R.D. Bull.

***Draba simmonsii*** Elven *&* Al-Shehbaz (D. alpina var. gracilescens Simmons), Figs 54B, 55A–Simmons’ draba | North American (N)

Newly recorded for Victoria I., from “30-Mile Cr.”, Cambridge Bay, Greiner L., Kuujjua R., the head of Minto Inl., Mt. Bumpus, Mt. Pelly, Tahiryuaq, Tahoe L. and Ulukhaktok. The older records included here were previously treated under *D. alpina* L. (Porsild and Cody 1980, Aiken et al. 2007). Distributed across the Arctic islands, with few records from mainland Nunavut and Northwest Territories (Elven and Al-Shehbaz 2008, Saarela et al. 2017b). The general status rank of this species in Northwest Territories is Sensitive (Working Group on General Status of NWT Species 2016).

**NORTHWEST TERRITORIES. Kuujjua R.**: *Gillespie et al. 9998* (CAN). **Minto Inl. (head)**: *Edlund 95* (CAN), *Gillespie et al. 10059* (CAN). **Tahiryuaq**: *Edlund 386* (CAN). **Ulukhaktok**: *Edlund 718* (CAN). **NUNAVUT. “30-Mile Cr.**”: *Bennett et al. 14-0342* (MO). **Cambridge Bay**: *Bennett 14-0375*, *14-0307* (MO), *13-0159* (BABY, chars, DAO), *14-0381* (MO, BABY), *Calder et al. 24211B* (DAO), *Gillespie et al. 8449* (CAN, MO, O), *8451b* (CAN), *Ponomarenko VI-057A*, *VI-080a* (cf.) (CAN), *Sweatman & Smith 8* (DAO). **Greiner L.**: *Ponomarenko VI-031*, *VI-051*, *VI-083C*, *VI-193*, *VI-296C*, *VI-322* (CAN). **Mt. Bumpus**: *Edlund 186*, *223* (CAN). **Ovayok TP**: *Bennett & Sullivan 13-0655* (DAO). **Tahoe L.**: *Porsild 17454* (CAN).

***Draba subcapitata*** Simmons, Fig. 54C–Ellesmere Island draba | Circumpolar

Previously recorded from Cambridge Bay, Collinson P., the head of Minto Inl. (Porsild obs., conf.), Hadley B., Storkerson P., Washburn L. and northwestern Wollaston P. (Porsild 1955, 1957, 1964, Porsild and Cody 1980, Aiken et al. 2007). Thannheiser et al. (2001) additionally recorded it from Wellington B. Newly recorded from Boot Inl., Mt. Pelly, Natkusiak P. and “Trunsky L.” Widespread across the Canadian Arctic Archipelago and on the mainland recorded from Melville and Boothia peninsulas, Naujaat (formerly Repulse Bay) and northern Quebec (Mulligan 1974, Porsild and Cody 1980, Aiken et al. 2007, Garneau and Sabourin 2018). The southern-most Victoria I. populations are near the southwestern limit of this high arctic species, with few records known from the adjacent mainland: one from the Croker R. delta (Cody et al. 1992, Saarela et al. 2013) and one from the south coast of Coronation Gulf (*Cox & O’Neill 641*, CAN 10057076, det. R. Elven, 2003, published by Macoun and Holm (1921) as *D. alpina*).

**NORTHWEST TERRITORIES. Boot Inl.**: *Gillespie et al. 9700* (CAN). **Minto Inl. (head)**: *Gillespie et al. 10300* (CAN). **Natkusiak P.**: *Edlund 116*, *123* (CAN). **Wollaston P. (NW)**: *Porsild 17224* (ALTA, CAN). **NUNAVUT. Cambridge Bay**: *Bennett et al. 13-0177* (BABY, chars, DAO, od), *14-0311* (DAO, UAAH), *Porsild 21629* (CAN), *Stephens 1114* (CAN), *Washburn 43* (CAN, GH), *43A* (CAN). **Collinson P.**: *Edlund & Argus 12761* (CAN). **Hadley B.**: *Edlund 22* (CAN). **Namaycush L.**: *Edlund 21* (CAN), *Roncato-Spencer 9* (CAN). **Ovayok TP**: *Bennett & Sullivan 13-0644* (BABY, DAO). **Storkerson P.**: *Edlund 201* (CAN). “**Trunsky L.**”: *Bennett et al. 14-0398* (BABY, DAO, MO). **Washburn L.**: *Porsild 17458* (CAN).

### *Erysimum* L. [2]

**Key to *Erysimum* [adapted from Al-Shehbaz (2010b)]:**

**Table:** 

1	Petals yellow; basal leaf blades oblanceolate to linear-oblanceolate, 2–7 mm wide; fruits 2.5–5.8(–6.4) cm × 1.8–2.5 mm	***E. coarctatum***
–	Petals usually purple, rarely lilac; basal leaf blades linear to narrowly linear-oblanceolate, 1–2 mm wide; fruits (3–)5–11(–13) cm × 2–4 mm	***E. pallasii***

***Erysimum coarctatum*** Fernald, Fig. 54D–Wallflower | North American (N)

Newly reported from Victoria I., known only from “Long L.” where collected by Lambert in 1964. This is the only record of the taxon for the Canadian Arctic Archipelago. The collection was overlooked in previous treatments (Porsild and Cody 1980, Aiken et al. 2007). Elsewhere in the Canadian Arctic recorded from scattered mainland sites (Porsild and Cody 1980, Gould and Walker 1997, Saarela et al. 2013, Saarela et al. 2017b).

**NUNAVUT. “Long L.**”: *Lambert s.n.* (CAN) (Suppl. material 11).

***Erysimum pallasii*** (Pursh) Fernald, Figs 54E, 55B–Pallas’ wallflower | Asian (N)–amphi-Beringian–North American (N)

Previously recorded from Cambridge Bay, C. Colborne, C. Wollaston, the head of Minto Inl., the head of Prince Albert S., Read I. and Ulukhaktok (Porsild 1955, 1957, 1964, Porsild and Cody 1980, Aiken et al. 2007). Thannheiser et al. (2001) additionally recorded it from Johansen B. (conf.). Newly recorded from “30-Mile Cr.”, Boot Inl., Clouston B., Oterkvik Pt., Prince Albert P. and Sinclair Cr. Elsewhere in the Canadian Arctic known from Axel Heiberg, northern Baffin, Banks, Bylot, Ellesmere and Melville islands and western mainland sites (Porsild and Cody 1980, Cody and Reading 2005, Aiken et al. 2007, Saarela et al. 2013, Saarela et al. 2017b).

**NORTHWEST TERRITORIES. Boot Inl.**: *Gillespie et al. 9533* (ari, CAN), *9697* (CAN). **C. Wollaston**: *Edlund 21* (CAN). **Kuujjua R.**: *Gillespie et al. 9731*, *9747* (CAN). **Minto Inl. (head)**: *Edlund 64* (CAN), *Gillespie et al. 10029* (ALA, CAN, O), *Porsild 17400*, *17401* (CAN). **Prince Albert P.**: *Oldenburg 54-236* (GH). **Prince Albert S. (head)**: *Stretton 55* (DAO), *Weerstra 5*, *22* (DAO). **Ulukhaktok**: *Bandringa 317* (CAN), *Bliss s.n.* (ALTA), *Edlund 362*, *748* (CAN), *Gray & Gibbard 6*, *40* (DAO), *Oldenburg 42-96*, *45-1694* (GH), *Porsild 17303* (ALTA, CAN), *Ross 5* (ALTA), *5B* (GH), *Saarela & Bull 1501* (CAN, O). **NUNAVUT. “30-Mile Cr.**”: *Bennett et al. 14-0378*, *14-0477* (CAN). **Cambridge Bay**: *Bennett 14-0303* (UBC), *13-0312* (BABY, chars), *Consaul & Gillespie 1132* (CAN), *8455* (CAN, O), *Stephens 1264* (KSTC), *1270* (CAN, KSTC). **C. Colborne**: *Edlund & Argus 12732* (CAN). **Clouston B.**: *Gillespie et al. 7739* (CAN, MO). **Johansen B.**: *Gillespie et al. 7970* (ALA, CAN, MO, MT, O), *8124* (CAN, O), *8162* (ALA, CAN, MO, MT, O, UBC), *8164* (CAN). **Oterkvik Pt.**: *Gillespie et al. 7625* (ALTA, BABY, CAN), *7691* (ALA, CAN, MO, O). **Read I.**: *Porsild 17198* (ALTA, CAN), *Ross 15* (ALTA), *15A* (GH), *Wood s.n.* (CAN). **Sinclair Cr.**: *Gillespie et al. 8250* (CAN, MO, O), *8325* (CAN, MO).

### *Eutrema* R.Br. [1]

***Eutrema edwardsii*** R.Br., Figs 54F, 55C–Edward’s eutrema | Circumpolar–alpine

Previously recorded from C. Wollaston, Hadley B., Minto Inl. (Porsild obs., conf.), the head of Prince Albert S. (Porsild obs.), Richard Collinson Inl. and Ulukhaktok. Thannheiser et al. (2001) additionally recorded it from Johansen B. (conf.), Richardson I., Surrey L. and Wellington B. Newly recorded from Boot Inl., south of Burns L., Greiner L., Mt. Bumpus, Namaycush L., Oterkvik Pt., Prince Albert P., Sinclair Cr., Storkerson P. and an inland site on southeastern Victoria I. Elsewhere in the Canadian Arctic recorded from Air Force, Axel Heiberg, Baffin, Banks, Coats, Cornwallis, Devon, Digges, Ellesmere, Melville, Prince Charles, Prince of Wales, Southampton and West Foxe islands and across the mainland (Porsild and Cody 1980, Cody et al. 1989, Korol 1992, Cody and Reading 2005, Aiken et al. 2007, Saarela et al. 2013, Saarela et al. 2017b, Garneau and Sabourin 2018).

**NORTHWEST TERRITORIES. Boot Inl.**: *Gillespie et al. 9563* (ALA, CAN, O), *9638* (CAN). **Burns L. (S)**: *Edlund 554*, *59* (CAN). **C. Wollaston**: *Edlund 41*, *66* (CAN). **Kuujjua R.**: *Gillespie et al. 9756a*, *9869*, *9989* (CAN). **Minto Inl. (head)**: *Edlund 66* (CAN), *Gillespie et al. 10056* (CAN). **Richard Collinson Inl.**: *Edlund 142*, *615*, *677* (CAN). **Ulukhaktok**: *Edlund 326*, *714*, *838*, *863* (CAN), *Oldenburg 45-1692* (GH), *Porsild 17304*, *17305*, *17306* (CAN). **NUNAVUT. Cambridge Bay**: *Bennett et al. 13-0171* (BABY, chars, od), *Calder et al. 24179* (DAO), *Edlund & Argus 12641* (CAN), *Polunin s.n.* (CAN, 3 sheets), *Ponomarenko VI-062* (CAN), *Stephens 934*, *1019*, *1033*, (KSTC), *937*, *1080* (CAN, KSTC), *Washburn 21* (CAN). **Greiner L.**: *Ponomarenko VI-052*, *VI-305*, *VI*-324 (CAN). **Hadley B.**: *Edlund 77*, *86*, *126* (CAN). **Johansen B.**: *Gillespie et al. 8033* (CAN, MO). **Mt. Bumpus**: *Edlund 221* (CAN). **Namaycush L.**: *Edlund & Roncato-Spencer 20*, *50* (CAN). **Oterkvik Pt.**: *Gillespie et al. 7499* (CAN, MO). **Prince Albert P.**: *Oldenburg 54-649* (GH). **Sinclair Cr.**: *Gillespie et al. 8256*, *8272* (CAN). **South-central Victoria I.**: *Edlund & Argus 12873* (CAN). **Storkerson P.**: *Edlund 167*, *173* (CAN).

### *Parrya* R.Br. [2]

**Key to *Parrya***

**Table:** 

1	Leaf blades entire or rarely obscurely dentate; petals 10–13 mm; anthers ovate, 0.8–1.5 mm; fruit not or scarcely torulose	***P. arctica***
–	Leaf blades entire to dentate or lobed; petals 12–22 mm; anthers linear-oblong, 1.5–2.5 mm; fruit usually torulose (constricted between seeds)	***P. nudicaulis***

***Parrya arctica*** R.Br., Figs 54G, 55D–Arctic false wallflower | North American (N)

Previously recorded from south of Burns L., Cambridge Bay, an unnamed lake ca. 60 mi. N of Cambridge Bay, C. Wollaston, Collinson P., Hadley B., the head of Minto Inl., Mt. Pelly, Namaycush L., Natkusiak P., the head of Prince Albert S., the head of Minto Inl., Richard Collinson Inl., Storkerson P., Tahiryuaq, Ulukhaktok, Walker B. and Wollaston P. (Macoun and Holm 1921, Porsild 1955, 1957, 1964, Porsild and Cody 1980, Aiken et al. 2007). Thannheiser et al. (2001) additionally recorded it from Johansen B. (conf.), Wellington B. and Surrey L. Newly recorded from “30-Mile Cr.”, Boot Inl., Falaise B., Ferguson L., Greiner L., Kuujjua R., “Oldenburg L.”, Oterkvik Pt., Read I. and “Trunsky L.” Elsewhere in the Canadian Arctic recorded from primarily the western Arctic islands (Banks, Bathurst, Cameron, Cornwallis, Eglinton, Jenny Lind, King William, Melville and Somerset), as well as Southampton and Ellesmere islands and mainland sites (Porsild and Cody 1980, Cody and Reading 2005, Aiken et al. 2007, Saarela et al. 2013).

**NORTHWEST TERRITORIES. Boot Inl.**: *Dutilly 18731* (DAO), *Gillespie et al. 9536*, *9695* (CAN, O), *9678* (CAN). **Burns L. (S)**: *Stretton 12*, *13* (DAO). **C. Wollaston**: *Edlund 3A*, *8* (CAN), *61* (CAN, ID). **Kuujjua R.**: *Gillespie et al. 10002* (CAN), *9760* (ari, CAN). **Minto Inl. (head)**: *Edlund 129* (CAN), *Gillespie et al. 10275* (CAN), *9476* (CAN, O). **Natkusiak P.**: *Edlund 119* (CAN). “**Oldenburg L.**”: *Oldenburg 45-1338* (GH). **Prince Albert S. (head)**: *Stretton 34*, *48* (DAO), *Weerstra 19* (DAO). **Prince Albert P.**: *Oldenburg 54-234* (GH, UBC). **Richard Collinson In.**: *Stretton 195*, *211* (DAO), *Edlund 179*, *419* (CAN), *P. Jenness 20* (CAN). **Storkerson P.**: *Stretton 224*, *231* (DAO). **Tahiryuaq**: *Edlund 389* (CAN). **Ulukhaktok**: *Gray & Gibbard 9* (DAO), *Ross 2* (ALTA), *2B* (GH), *Saarela & Bull 1483* (CAN), *Svoboda 745017* (DAO). **Walker B.**: *Porsild 17495* (CAN). **Wollaston P.**: *Oldenburg 54-492* (GH). **NUNAVUT. “30-Mile Cr.**”: *Bennett et al. 14-0353* (ALA). **Cambridge Bay**: *Bennett 13-0218*, *14-0371* (UBC), *13-0322* (BABY, chars, MO, od), *14-0322* (UAAH), *Calder et al. 24199* (DAO), *Consaul & Gillespie 1128* (CAN), *Gould s.n.* (ALA), *Polunin s.n.* (CAN, 3 sheets), *Ponomarenko VI-096* (CAN), *Porsild 21630*, *21631* (CAN), *Scotter s.n.* (ALTA), *Stephens 1006*, *1090* (KSTC), *974* (CAN), *Sweatman & Smith 6* (DAO), *10* (CAN, DAO), *Washburn 26* (CAN). **Collinson P.**: *Edlund & Argus 12764* (CAN). **Falaise B.**: *Eriksen et al. 935* (ALA). **Ferguson L. [Tahiryuaq**]: *Bennett et al. 14-0419* (DAO, UBC). **Greiner L.**: *Ponomarenko VI-040*, *VI-217*, *VI-223A* (CAN). **Hadley B.**: *Edlund 14*, *15*, *88*, *89* (CAN). **Johansen B.**: *Gillespie et al. 8026* (CAN, MO). **Ovayok TP**: *Gillespie et al. 8437* (CAN, MO), *Gould s.n.* (ALA), *Stephens 865* (CAN, KSTC). **Namaycush L.**: *Edlund & Argus 12800A*, *12800B* (CAN), *Edlund & Roncato-Spencer 26* (CAN), *Edlund 7*, *8* (CAN). **Oterkvik Pt.**: *Gillespie et al. 7488* (CAN, MO), *7804* (CAN). **Unnamed lake ca. 60 mi. N of Cambridge Bay**: *Porsild 17477* (CAN). **Read I.**: *Ross 1a* (GH). **Storkerson P.**: *Edlund 169* (CAN), *P. Jenness 35* (CAN). “**Trunsky L.**”: *Bennett et al. 14-0390* (BABY, CAN). **Wollaston P.**: *D. Jenness 412* (CAN), *Porsild 17225*, *17226* (CAN).

***Parrya nudicaulis*** (L.) Regel, Fig. 54H–Naked-stemmed false wallflower | European (NE)–Asian (N/C)–amphi-Beringian–Cordillera

One specimen (*Jenness 650*) is assigned to this species based on its distinctly dentate-lobed leaves, linear-oblong anthers and torulose young fruit. Porsild and Cody (1980) show one dot on Wollaston Peninsula, probably based on this specimen. Several collections previously identified as this species are here re-identified as *P. arctica*, including one from Richard Collinson Inlet (*Edlund 419*) mapped as *P. nudicaulis* by Aiken et al. (2007). Preliminary molecular and morphological analyses (S. Godfrey and Gillespie, unpublished data) suggest that all *Parrya* specimens analyzed so far from Nunavut and Northwest Territories (including several previously identified as *P. nudicaulis*) belong to *P. arctica* and that this species can be more robust on the southern edge of its range (such as southern Victoria Island) than is typical and that current keys indicate. Further study is needed to verify the presence and range of *P. nudicaulis* on Victoria Island. Elsewhere in the Canadian Arctic recorded from Arctic Yukon and adjacent Northwest Territories (Porsild and Cody 1980, Cody 1996b), and from Banks I. based on a single record (*Edlund s.n.*, CAN 10027184). Collections from Bernard Harbour, mainland Nunavut, mapped in Porsild and Cody (1980) have been redetermined to *P. arctica*.

**NUNAVUT. Wollaston P.**: *D. Jenness 650* (CAN).

### *Physaria* Rchb. [1]

***Physaria arctica*** (Wormsk. ex Hornem.) O’Kane *&* Al-Shehbaz, Fig. 54I–Arctic bladderpod | Asian (N)–amphi-Beringian–North American (N)

Previously recorded from Byron B., Cambridge Bay, an unnamed lake ca. 60 mi. N of Cambridge Bay, Hadley B., the head of Minto Inl., Mt. Lady Pelly, Namaycush L., the north side and head of Prince Albert S., Read I., Richard Collinson Inl., Ulukhaktok and Walker B. (Porsild 1955, 1957, 1964, Porsild and Cody 1980, Aiken et al. 2007). Thannheiser et al. (2001) additionally recorded it from Johansen B. (conf.), Mt. Pelly (conf.), Surrey L. and Wellington B. Newly recorded from Albert Edward B., Boot Inl., C. Wollaston, Falaise B., Greiner L., Kuujjua R., an inland site on Prince Albert P., Sinclair Cr., “Trunsky L.” and Wollaston P. Elsewhere in the Canadian Arctic recorded from Baffin, Banks, Coats, Devon, Ellesmere and Southampton islands and scattered mainland sites (Rollins and Shaw 1973, Porsild and Cody 1980, Aiken et al. 2007, Garneau and Sabourin 2018).

**NORTHWEST TERRITORIES. Boot Inl.**: *Gillespie et al. 9526b* (CAN, O). **C. Wollaston**: *Edlund 6*, *49* (CAN). **Kuujjua R.**: *Gillespie et al. 9762* (CAN, O). **Minto Inl. (head)**: *Edlund* 59 (CAN), *Gillespie et al. 9481* (CAN), *Porsild 17402* (CAN). **Prince Albert P.**: *Oldenburg 54*-*655* (GH, UBC), *54-235* (GH). **Prince Albert S. (head)**: *Edlund & Argus 12814* (CAN), *Weerstra* (DAO). **Prince Albert S. (N)**: *Stretton 40* (DAO). **Richard Collinson Inl.**: *Edlund 140*, *182* (CAN). **Ulukhaktok**: *Edlund 292*, *448* (CAN), *Ross 24* (ALTA), *24A* (GH), *Saarela & Bull 1506* (CAN, O). **Walker B.**: *Porsild 17494* (CAN). **Wollaston P.**: *Oldenburg 54-493* (GH). **NUNAVUT. Albert Edward B.**: *Ponomarenko VI-270* (CAN). **Cambridge Bay**: *Bennett et al. 13-0176* (BABY, chars), *13-0207* (od), *Calder et al. 24195* (DAO), *Hainault 2143* (DAO), *Ponomarenko VI-073A* (CAN), *Stephens 981* (CAN), *1110* (KSTC). **Falaise B.**: *Eriksen et al. 931* (ALA). **Ferguson L. [Tahiryuaq**]: *Hainault 2034* (DAO). **Greiner L.**: *Ponomarenko VI-201* (CAN). **Hadley B.**: *Edlund 29*, *110* (CAN). **Johansen B.**: *Gillespie et al. 7886* (ALA, CAN, MO, O), *Oswald 149* (DAO). **Mt. Lady Pelly [Amaaqtuq**]: *Hainault 1888* (DAO). **Ovayok TP**: *Gillespie et al. 8430* (CAN, O), *Gould s.n.* (ALA). **Namaycush L.**: *Edlund 15*, *22*, *36* (CAN). **Oterkvik Pt.**: *Gillespie et al. 7465* (ALA, CAN, MO, MT, O). **Read I.**: *Porsild 17199* (CAN). **Sinclair Cr.**: *Gillespie et al. 8332* (ALA, CAN, MO, MT, O). “**Trunsky L.**”: *Bennett et al. 14-0393* (CAN). **Unnamed lake ca. 60 mi. N of Cambridge Bay**: *Porsild 17476* (CAN).

### Superasterids

**Caryophyllales**

**Plumbaginaceae [1/1]**

***Armeria* Willd. [1]**

**Figure 56.**
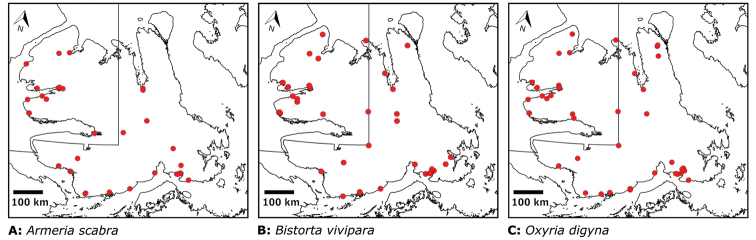
Species distribution maps. Plumbaginaceae: **A***Armeria scabra*. Polygonaceae: **B***Bistorta vivipara***C***Oxyria digyna*.

**Figure 57.**
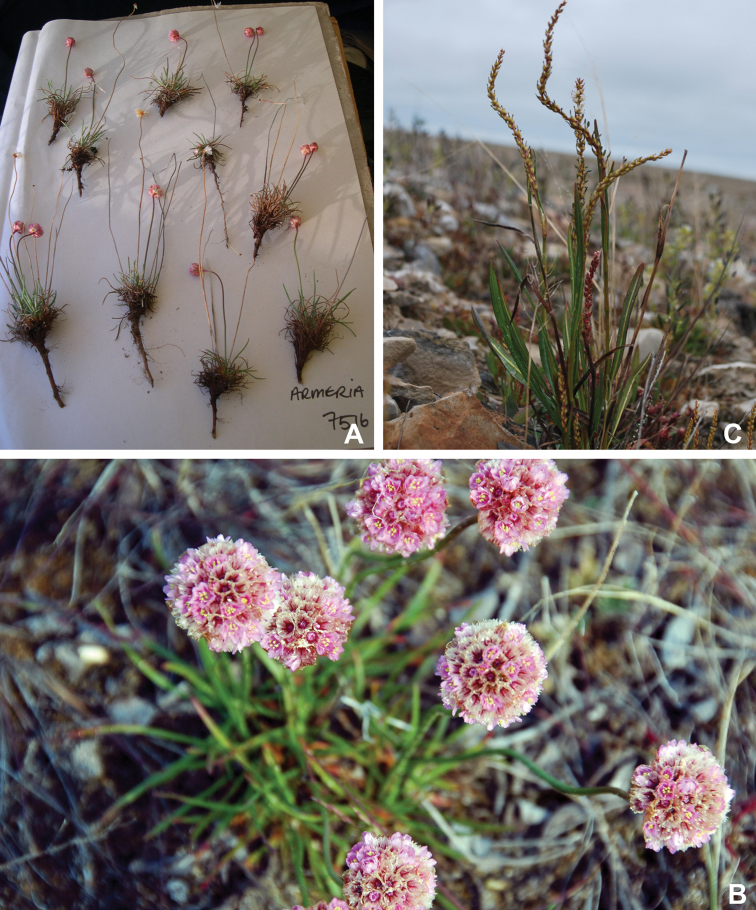
**A***Armeria scabra* plants ready to be pressed, *Gillespie et al. 7516***B***Armeria scabra* habit **C***Bistorta vivipara* habit. Photos **A**, **C** by R.D. Bull and **B** by B.A. Bennett.

***Armeria scabra*** Pall. ex Roem. *&* Schult. (A. maritima subsp. sibirica (Turcz. ex Boiss.) Nyman), Figs 56A, Fig. 57A, B–Sea thrift | Circumpolar

Previously recorded from C. Wollaston, Gordon Pt., the head of Minto Inl., Mt. Lady Pelly, Richard Collinson Inl. and Wollaston P. (Macoun and Holm 1921, Porsild 1955, 1957, 1964, Porsild and Cody 1980, Aiken et al. 2007). The site mapped on the north shore of Prince Albert S. in Aiken et al. (2007) is a mapping error. This collection (*Shindman s.n.*) was gathered on George Island at the head of the sound during “Operation Magnetic, 1949”, a magnetic survey of the Canadian Arctic, of which B. Shindman was an expedition member; this was the only site visited on Victoria Island (Hutchison 1949). Thannheiser et al. (2001) additionally recorded it from Hadley B. (conf.), Johansen B. (conf.) and Surrey L. Newly recorded from “30-Mile Cr.”, Anderson B., Cambridge Bay, Boot Inl., Falaise B., Hadley B., Kuujjua R., Namaycush L., Oterkvik Pt., the head of Prince Albert S., Read I., Sinclair Cr., “Trunsky L.” and Ulukhaktok. Elsewhere in the Canadian Arctic recorded from Axel Heiberg, Baffin, Banks, Bylot, Devon, Ellesmere, King William, Somerset, Southampton and West Foxe islands and across the mainland (Porsild and Cody 1980, Aiken et al. 2007, Saarela et al. 2013, Saarela et al. 2017b).

**NORTHWEST TERRITORIES. Boot Inl.**: *Gillespie et al. 9632* (ALA, ari, CAN, MT, O). **C. Wollaston**: *Edlund 5* (CAN). **Diamond Jenness P. (W end)**: *Stretton 84* (DAO). **Gordon Pt.**: *Stretton 198* (DAO). **Kuujjua R.**: *Dutilly 18776*, *18777*, *18777a* (QFA), *Gillespie et al. 9805* (CAN, O, UBC, WIN). **Minto Inl. (head)**: *Edlund 126* (CAN), *Gillespie et al. 10197* (ALA, CAN, MT, O), *Porsild 17404* (CAN). **Prince Albert S. (head)**: *Edlund & Argus 12815* (CAN), *Shindman s.n.* (DAO). **Richard Collinson Inl.**: *P. Jenness 12* (CAN), *Edlund 176* (CAN). **Ulukhaktok**: *Bliss s.n.* (ALTA, CAN), *Edlund 294* (CAN), *Oldenburg 45-1544* (CAN), *Ross 29* (ALTA, CAN), *29A* (GH), *Saarela & Bull 1414* (ALA, CAN, O). **NUNAVUT. “30-Mile Cr.**”: *Bennett et al. 14-0359* (ALA, CAN). **Anderson B.**: *Edlund & Argus 12705* (CAN). **Cambridge Bay**: *Bennett et al. 13-0232* (chars), *13-0309* (BABY, CAN, chars), *Gillespie et al. 8464* (ALTA, CAN), *8507* (CAN), *Stephens 1005* (KSTC), *1085* (CAN, KSTC), *Sutton 1005* (CAN). **Falaise B.**: *Eriksen et al. 971* (ALA), *Parker 9109* (ALA, BABY). **Hadley B.**: *Edlund 114*, *143* (CAN). **Johansen B.**: *Gillespie et al. 7898* (CAN). **Mt. Lady Pelly [Amaaqtuq**]: *Jones & Hainault 28* (DAO). **Namaycush L.**: *Edlund & Roncato-Spencer 46* (CAN), *Edlund 27*, *130* (CAN). **Oterkvik Pt.**: *Gillespie et al. 7516* (ALA, BABY, CAN, MT, O, UBC), *7563* (CAN). **Read I.**: *Oldenburg 42-501*, *43-1072*, *43-954*, *44*-*1036* (CAN), *Ross 25* (ALTA), *25A* (GH). **Sinclair Cr.**: *Gillespie et al. 8333* (CAN). “**Trunsky L.**”: *Bennett et al. 14-0389* (BABY, UBC). **Wollaston P.**: *D. Jenness 578* (CAN).

### Polygonaceae [2/2]

**Key to Polygonaceae [adapted from Freeman and Reveal (2005), Freeman and Hinds (2005) and Freeman and Packer (2005)]:**

**Table:** 

1	Leaf blades reniform; inflorescences bearing flowers, flowers 2–6 per ocreate fascicle; perianth greenish to reddish brown; achenes lenticular, winged	***Oxyria digyna***
–	Leaf blades linear to lanceolate or oblong-ovate; inflorescences usually bearing pink to brown or purple pyriform bulblets proximally and sterile flowers distally, sterile flowers 1–2 per ocreate fascicle; perianth greenish proximally, usually white or pink distally, rarely red; achenes rarely produced, when present trigonous, unwinged	***Bistorta vivipara***

### *Bistorta* (L.) Scop. [1]

***Bistorta vivipara*** (L.) Delarbre (*Persicaria vivipara* (L.) Ronse Decr., *Polygonum viviparum* L.), Figs 56B, 57C–Alpine bistort | Circumboreal–polar

Previously recorded from Byron B., Cambridge Bay, the head of Minto Inl., Mt. Bumpus, Namaycush L., Natkusiak P., east of the head of Prince Albert S., Richard Collinson Inl., Storkerson P. and Ulukhaktok (Porsild 1955, 1957, 1964, Porsild and Cody 1980, Aiken et al. 2007). Thannheiser et al. (2001) additionally recorded it from Johansen B. (conf.), Mt. Pelly, Richardson I., Surrey L. and Wellington B. Newly recorded from Albert Edward B., Boot Inl., south of Burns L., Ferguson L., Kuujjua R., “Oldenburg L.”, Oterkvik Pt., the north side of Prince Albert S., Richardson I. and Washburn L. Widespread across the Canadian Arctic islands and mainland (Porsild and Cody 1980, Cody et al. 1989, Korol 1992, Cody and Reading 2005, Aiken et al. 2007, Saarela et al. 2013, Saarela et al. 2017b).

**NORTHWEST TERRITORIES. Boot Inl.**: *Dutilly 18689* (QFA), *Gillespie et al. 9517* (CAN). **Burns L. (S)**: *Edlund 17* (CAN). **Byron B.**: *Dushenko* (UVIC). **Kuujjua R.**: *Dutilly 18783*, *18804* (QFA), *Edlund 640* (CAN), *Gillespie et al. 9801* (CAN, O). **Minto Inl. (head)**: *Edlund 60* (CAN), *Gillespie et al. 9467* (CAN). **Natkusiak P.**: *Edlund 73* (CAN). “**Oldenburg L.**”: *Oldenburg 45-1355* (CAN). **Prince Albert S. (N)**: *Oldenburg 46-2292* (CAN). **Richard Collinson Inl.**: *Edlund 191*, *607* (CAN). **Ulukhaktok**: *Dutilly 18645* (QFA), *Edlund 365*, *451* (CAN), *Gray & Gibbard 24*, *48*, *s.n.* (DAO), *Oldenburg 42-55*, *42-60*, *45-1558*, *45-1559* (CAN), *Porsild 17276*, *17277* (CAN), *Saarela & Bull 1455* (ALA, ari, CAN), *1508* (CAN, O), *Stretton 60* (DAO). **Walker B.**: *Oldenburg 45-1503* (CAN). **NUNAVUT. Albert Edward B.**: *Ponomarenko VI-337C* (CAN). **Cambridge Bay**: *Dutilly 28060* (QFA), *Bennett et al. 13-0190* (chars, V), *Consaul & Gillespie 1131*, *1136* (CAN), *Edlund & Argus 12668* (CAN), *Fortier 14* (CAN), *Gillespie et al. 8384* (ALA, CAN, MT, O), *Gould s.n.* (ALA), *Oldenburg 44*-*882* (CAN), *Polunin s.n.* (CAN), *Smith & Sweatman 45* (DAO), *Stephens 1040*, *1081* (CAN, KANU, KSTC), *1041*, *1082* (KANU, KSTC), *Thomson s.n.* (WIS), *Washburn 29* (CAN). **Ferguson L. [Tahiryuaq**]: *Hainault 2005* (DAO), *Ponomarenko VI-232A*, *VI-305A* (CAN). **Hadley B.**: *Edlund 72*, *137* (CAN). **Johansen B.**: *Gillespie et al. 7900*, *7942* (CAN). **Mt. Bumpus**: *Edlund 268* (CAN). **Namaycush L.**: *Edlund & Roncato-Spencer 44* (CAN). **Oterkvik Pt.**: *Gillespie et al. 7703* (CAN, O). **Prince Albert S. (head)**: *Edlund 96* (CAN). **Read I.**: *Oldenburg 43-1035* (CAN). **Storkerson P.**: *Edlund* 186 (CAN). **Washburn L.**: *Oldenburg 46-2183* (CAN).

### *Oxyria* Hill [1]

***Oxyria digyna*** Hill, Fig. 56C–Mountain sorrel | Circumpolar–alpine

Previously recorded from Byron B., Cambridge Bay, Hadley B., the head of Minto Inl., Natkusiak P., the north side of Prince Albert S., Richard Collinson Inl., Tahiryuaq, Walker B. (Porsild obs., conf.) and Wollaston P. (Macoun and Holm 1921, Porsild 1955, 1957, 1964, Porsild and Cody 1980, Aiken et al. 2007). Thannheiser et al. (2001) additionally recorded it from Johansen B. (conf.), Mt. Pelly (conf.), Richardson I. and Surrey L. Newly recorded from Boot Inl., C. Wollaston, Falaise B., Ferguson L., Greiner L., “Oldenburg L.”, Oterkvik Pt., southeast of the head of Prince Albert S., Sinclair Cr. and Washburn L. Widespread across the Canadian Arctic Archipelago and mainland (Porsild and Cody 1980, Cody et al. 1989, Korol 1992, Aiken et al. 2007, Saarela et al. 2013, Blondeau 2015d, Saarela et al. 2017b).

**NORTHWEST TERRITORIES. Boot Inl.**: *Gillespie et al. 9599* (ari, CAN). **C. Wollaston**: *Edlund 47* (CAN). **Kuujjua R.**: *Dutilly 18836* (QFA), *Gillespie et al. 9739* (ALA, CAN, MT, O), *Stretton 73* (DAO). **Minto Inl. (head)**: *Edlund 108* (CAN), *Gillespie et al. 10215* (CAN, MT, UBC, WIN), *9463* (ALA, CAN, O). **Natkusiak P.**: *Edlund 72* (CAN). “**Oldenburg L.**”: *Oldenburg 45-1354* (CAN). **Prince Albert S. (head)**: *Edlund 56* (CAN). **Prince Albert S. (N)**: *Oldenburg 46-2293* (CAN), *Stretton 37* (DAO). **Richard Collinson Inl.**: *Edlund 192* (CAN), *P. Jenness 14* (CAN). **Tahiryuaq**: *Edlund 157* (CAN). **Ulukhaktok**: *Dutilly 18652* (QFA), *Edlund* 366 (CAN), *Gray & Gibbard 31* (DAO), *Oldenburg 45-1560* (CAN), *Porsild 17275* (CAN), *Ross 4* (ALTA, GH), *Saarela & Bull 1486* (CAN, O). **Walker B.**: *Oldenburg 45-1502* (CAN). **NUNAVUT. “30-Mile Cr.**”: *Bennett et al. 14-0355* (BABY, UBC). **Anderson B.**: *Edlund & Argus 12715* (CAN). **Burns L. (S)**: *Edlund 155* (CAN). **Cambridge Bay**: *Bennett & Sullivan 13-0293* (chars, od, V), *Consaul & Gillespie 1129* (CAN), *Gillespie et al. 8458* (ALA, CAN, O, V), *Polunin s.n.* (CAN), *Ponomarenko VI-072* (CAN). **Falaise B.**: *Eriksen et al. 968* (ALA). **Ferguson L. [Tahiryuaq**]: *Hainault 1956* (DAO). **Greiner L.**: *Ponomarenko VI-214*, *VI-224A*, *VI-290* (CAN). **Hadley B.**: *Edlund 17*, *84* (CAN). **Johansen B.**: *Gillespie et al. 7932* (ALA, CAN, MT, O), *8101* (ALA, CAN, O). **Ovayok TP**: *Gillespie et al. 8424* (CAN, O), *Stephens 986* (CAN, KSTC). **Oterkvik Pt.**: *Gillespie et al. 7473* (CAN, O, V). **Prince Albert S. (head)**: *Edlund 90* (CAN). **Sinclair Cr.**: *Gillespie et al. 8253* (ALA, CAN, MT, O, UBC, V), *8322* (CAN, V, Z). **Storkerson P.**: *Edlund 164*, *192* (CAN), *Stretton 222* (DAO). **Washburn L.**: *Oldenburg 46-2185* (CAN). **Wollaston P.**: *D. Jenness 655* (CAN).

### Caryophyllaceae [7/19/21]

**Key to Caryophyllaceae [adapted from Rabeler and Hartman (2005)]**

**Table:** 

1	Sepals united into a tube; petals pink or white	*** Silene ***
–	Sepals free; petals white	**2**
2	Petal apices 2-lobed or 2-fid, often divided nearly to base, or 4-fid	**3**
–	Petal apices entire, emarginate, jagged or notched	**4**
3	Capsules cylindric, often ± curved, opening by 10 teeth; styles 5	*** Cerastium ***
–	Capsules ovoid to globose, opening by 6 valves; styles 3(–5)	*** Stellaria ***
4	Leaf blades conspicuously fleshy	*** Honckenya ***
–	Leaf blades herbaceous to slightly succulent	**5**
5	Capsule valves or teeth 6, two times number of styles	*** Arenaria ***
–	Capsule valves 4 or 5, equal in number to styles	**6**
6	Sepals 4 or 5; styles 4 or 5; capsule valves 4 or 5	*** Sagina ***
–	Sepals 5; styles 3, occasionally 4; capsule valves or teeth 3	*** Sabulina ***

### *Arenaria* L. [2]

**Key to *Arenaria* [adapted from Hartman et al. (2005)]**

**Table:** 

1	Flowering pedicels (5–)9–20 mm, flowers long-exserted above leaves; sepals glandular villous basally; leaf blade margins often ciliate proximally; capsules ellipsoid	***A. longipedunculata***
–	Flowering pedicels 1–5 mm, flowers not or little exserted above leaves; sepals glabrous; leaf blade margins smooth; capsules broadly ellipsoid	***A. humifusa***

**Figure 58.**
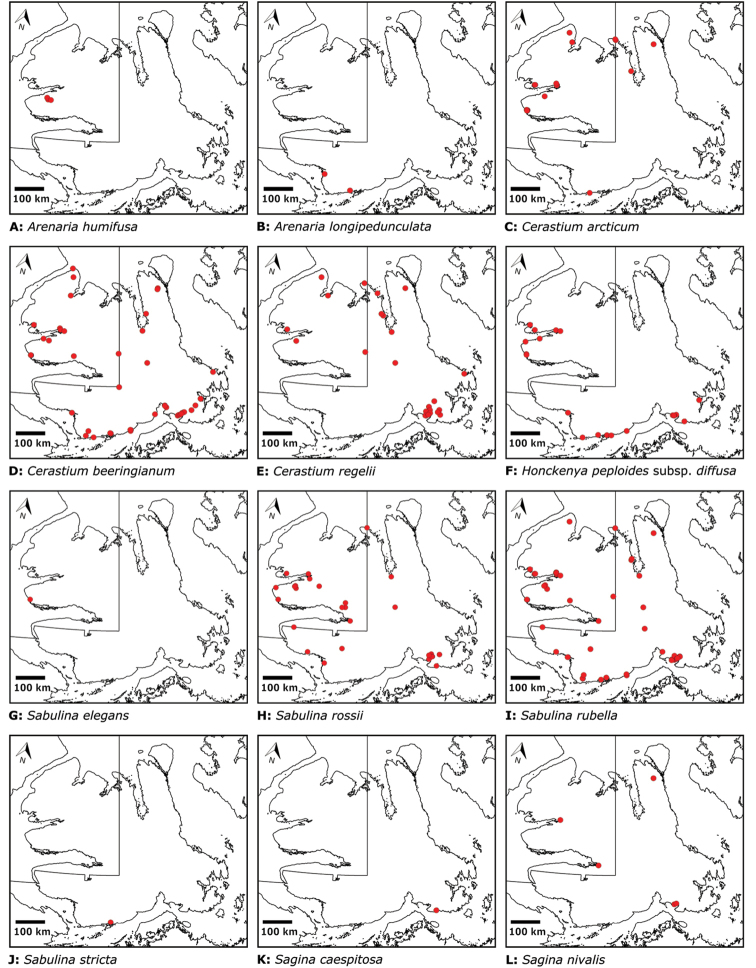
Species distribution maps. Caryophyllaceae: **A***Arenaria humifusa***B***Arenaria longipedunculata***C***Cerastium arcticum***D***Cerastium beeringianum***E***Cerastium regelii***F**Honckenya peploides subsp. diffusa**G***Sabulina elegans***H***Sabulina rossii***I***Sabulina rubella***J***Sabulina stricta***K***Sagina caespitosa***L***Sagina nivalis*.

***Arenaria humifusa*** Wahlenb., Fig. 58A–Creeping sandwort | North American (N)–amphi-Atlantic

Known from three sites in the Kuujjua R. area, where we collected it in 2010 growing on inland sand dunes (Gillespie et al. 2015). Edlund made a collection of this species in 1982 in the same general area, with the habitat recorded as dunes, but previous workers were unaware of this collection as it was part of unprocessed herbarium backlog; it is newly recorded here. Elsewhere in the Canadian Arctic recorded from Baffin, Coats, Cornwallis, Ellesmere, Igloolik and Southampton islands and mainland sites in Northwest Territories, Nunavut, and northern Quebec and Labrador (Porsild and Cody 1980, Korol 1992, Aiken et al. 2007, Saarela et al. 2013, Blondeau 2015a, Saarela et al. 2017b).

**NORTHWEST TERRITORIES. Kuujjua R.**: *Edlund 662* (CAN), *Gillespie et al. 9882* (ALA, ari, CAN, O), *9893* (ALA, ari, CAN, O), *9971* (CAN).

***Arenaria longipedunculata*** Hultén, Fig. 58B–Long-stemmed sandwort | Arctic-alpine amphi-Beringian–North America

Known from Clouston B. and Johansen B., where collected in 2008; details are provided in Gillespie et al. (2015). The general status rank of this species in Northwest Territories is Sensitive (Working Group on General Status of NWT Species 2016); there are no records from the Northwest Territories portion of Victoria I.

**NUNAVUT. Clouston B.**: *Gillespie et al.* 7721 (CAN). **Johansen B.**: *Gillespie et al. 8136* (CAN).

### *Cerastium* L. [3]

**Key to *Cerastium* [adapted from Morton (2005a)]**

**Table:** 

1	Leaf blades succulent, subglabrous or ciliate; plants often not flowering	***C. regelii***
–	Leaf blades not succulent, pubescent; plants normally flowering	**2**
2	Inflorescences (1–)3–10-flowered cymes; sepals 3–7 mm, petals ± equalling sepals in length	***C. beeringianum***
–	Inflorescences 1–3-flowered cymes; sepals 8–11 mm; petals 1–2× length of sepals	***C. arcticum***

**Figure 59.**
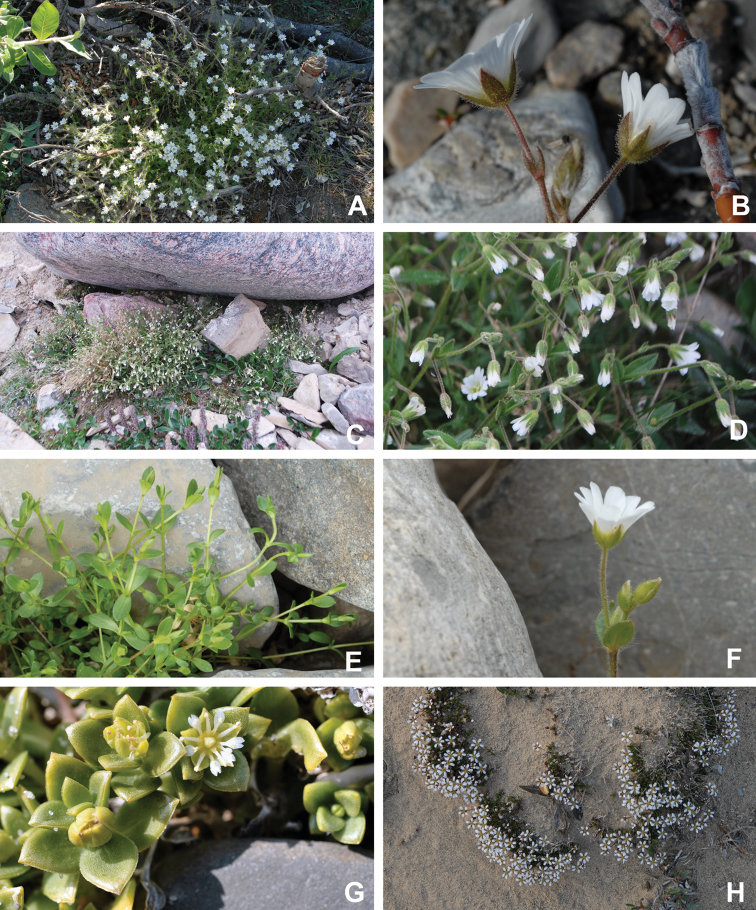
**A***Cerastium arcticum* habit, *Gillespie et al. 10164***B***Cerastium arcticum* inflorescence, *Gillespie et al. 9823***C***Cerastium beeringianum* habit **D***Cerastium beeringianum* inflorescence **E***Cerastium regelii* habit, *Gillespie et al. 9710***F***Cerastium regelii* inflorescence, *Gillespie et al. 9710***G**Honckenya peploides subsp. diffusa habit and inflorescence, *Gillespie et al. 9385***H***Sabulina rossii* habit, *Gillespie et al. 9385.* Photos **A**, **B**, **E**, **F**, **H** by L.J. Gillespie **C**, **D** by B.A. Bennett and **G** by R.D. Bull.

***Cerastium arcticum*** Lange, Figs 58C, 59A, B–Arctic chickweed | North American (N)–amphi-Atlantic–European (N)

The *Cerastium alpinum* L. aggregate, of which *C. arcticum* is a part, is a taxonomically complicated polyploid group (Elven et al. 2011), and earlier treatments did not distinguish *C. alpinum* as separate from *C. arcticum*. No collections of *C. alpinums.l.* were recorded on Victoria I. by Porsild (Porsild 1955, 1957, 1964, Porsild and Cody 1980). Aiken et al. (2007) recognized *C. arcticum* and recorded it on Victoria I. from the head of Minto Inl., Richard Collinson Inl. and Ulukhaktok (Aiken et al. 2007). It is newly recorded from Hadley B., Kuujjua R., Natkusiak P., “Oldenburg L.” and Oterkvik Pt. Elsewhere in the Canadian Arctic recorded from across the archipelago, a few mainland Nunavut and Northwest Territories sites and northern Quebec and Labrador (Porsild and Cody 1980, Aiken et al. 2007, Saarela et al. 2013, Blondeau 2015a). *Cerastium alpinum* s.s. is an amphi-Atlantic species recorded from the eastern Canadian Arctic islands (Axel Heiberg, Baffin, Cornwallis, Devon, Ellesmere and Somerset), mainland Nunavut sites and northern Quebec and Labrador (Aiken et al. 2007, Blondeau 2015a, Saarela et al. 2017b).

The morphology of one collection (*Oldenburg 42-33B*), from Ulukhaktok, is somewhat intermediate between *C. arcticum* and *C. alpinum*. *Cerastium alpinum* differs from *C. arcticum* by having hairs on leaf blades and/or stems flexuous, often tangled, translucent and silvery (vs. hairs on leaf blades and/or stems straight, not tangled, and yellowish or straw-coloured) (Morton 2005a). Hairs on the basal-most leaves of the Oldenburg collection are ±flexuous and tangled, approaching the morphology typical of *C. alpinum*, whereas those on non-basal leaves are straighter, as in *C. arcticum*; all hairs on the leaves are yellowish, as in *C. arcticum*. We have included this collection in *C. arcticum*. Additional collections from the Ulukhaktok area should be gathered to determine if *C. alpinum* occurs there.

**NORTHWEST TERRITORIES. Boot Inl.**: *Gillespie et al. 9526* (ALA, CAN, MT, O), *9572*, *9691* (CAN, O). **Kuujjua R.**: *Gillespie et al. 9823* (CAN). **Minto Inl. (head)**: *Edlund 54*, *612* (CAN), *Gillespie et al. 10127*, *10128a* (CAN, O), *10030* (CAN), *10164* (ALA, ALTA, ari, CAN, MO, MT, O, UBC, US, WIN). **Natkusiak P.**: *Edlund 87*, *89* (CAN). “**Oldenburg L.**”: *Oldenburg 45-1345* (CAN, GH). **Richard Collinson Inl.**: *Edlund* 417 (CAN). **Ulukhaktok**: *Edlund 840*, *847* (CAN), *Oldenburg 42-33B*, *45-1697* (CAN), *Porsild 17280* (CAN). **NUNAVUT. Hadley B.**: *Edlund 57* (CAN). **Oterkvik Pt.**: *Gillespie et al. 7694* (CAN).

***Cerastium beeringianum*** Cham. *&* Schltdl., Figs 58D, 59C, D–Bering Sea chickweed | Asian (N)–amphi-Beringian–North American (N)

Previously recorded from Byron B., Cambridge Bay, Collinson P., Hadley B., the head of Minto Inl., Namaycush L., Peel Pt., Read I., Richard Collinson Inl., Storkerson P. and Ulukhaktok (Porsild 1955, 1957, 1964, Porsild and Cody 1980). Newly recorded from “30-Mile Cr.”, Albert Edward B., south of Burns L., the Greiner L. watershed, Johansen B., Kuujjua R., “Oldenburg L.”, Oterkvik Pt., southeast of the head and north side of Prince Albert S., Sinclair Cr. and Walker B. The collection *Porsild 17280* from Ulukhaktok reported as this species (Porsild 1955) has been redetermined as *C. arcticum*. Elsewhere in the Canadian Arctic recorded across the western and northern islands, southern sites on the eastern islands and numerous mainland sites (Porsild and Cody 1980, Cody and Reading 2005, Saarela et al. 2013, Blondeau 2015a, Saarela et al. 2017b).

**NORTHWEST TERRITORIES. Burns L. (S)**: *Edlund 47* (CAN). **Kuujjua R.**: *Gillespie et al. 9842* (CAN, O), *9920* (CAN). **Minto Inl. (head)**: *Gillespie et al. 10128b*, *10174* (CAN), *10080* (ari, CAN), *10127* (CAN, O), *Porsild 17386* (CAN). “**Oldenburg L.**”: *Oldenburg 45-1341* (CAN). **Peel Pt.**: *Edlund 420* (CAN), **Prince Albert S. (N)**: *Oldenburg 46-2285* (CAN). **Richard Collinson Inl.**: *P. Jenness 16* (CAN). **Ulukhaktok**: *Edlund 344* (CAN), *Gray & Gibbard* 28 (DAO), *Ross 7B* (GH), *Saarela & Bull 1437* (ALA, CAN, MT, O, UBC), *1461* (ALA, CAN, O). **Walker B.**: *Oldenburg 45-1539A* (CAN, GH). **NUNAVUT. “30-Mile Cr.**”: *Bennett et al. 14-0361* (CAN). **Albert Edward B.**: *Ponomarenko VI-258*, *VI-335* (CAN). **Byron B.**: *Dushenko* (UVIC). **Cambridge Bay**: *Bennett et al. 13-0196* (CAN, chars, od, UBC), *Gillespie et al. 8470* (ALA, CAN, MT, O, UBC), *Oldenburg 44*-*936* (CAN), *Polunin s.n.* (CAN), *Porsild 21612* (CAN), *Stephens 996*, *1048*, *1145* (CAN, KANU, KSTC), 955 (CAN, KSTC), *Tasker 2430* (CAN). **Collinson P.**: *Edlund & Argus* 12763 (CAN). **Ferguson L. [Tahiryuaq**]: *Bennett et al. 14-0414* (ALA, CAN, UBC), *Hainault 1949*, *2007* (DAO). **Greiner L.**: *Ponomarenko VI-160*, *VI-244* (CAN). **Hadley B.**: *Edlund 134*, *327* (CAN). **Johansen B.**: *Gillespie et al. 7824* (CAN, O), *7971* (ALA, CAN, MT, O, UBC). **Ovayok TP**: *Gillespie et al. 8436* (ALA, CAN, MT, O, UBC), *Stephens 1166* (CAN, KANU, KSTC). **Namaycush L.**: *Edlund 27* (CAN). **Oterkvik Pt.**: *Gillespie et al. 7577*, *7670*, *7672* (CAN). **Prince Albert S. (head)**: *Edlund 78* (CAN). **Read I.**: *Oldenburg 42-520*, *43-927* (CAN). **Sinclair Cr.**: *Gillespie et al. 8226* (CAN, O), *8312* (BABY, CAN, O). **Storkerson P.**: *Edlund 171*, *202*, *224* (CAN).

***Cerastium regelii*** Ostenf. (*C. gorodkovianum* Schischk.), Figs 58E, 59E, F–Regel’s chickweed | Circumpolar

Previously recorded from Cambridge Bay, Collinson P., Hadley B., Namaycush L., Natkusiak P., Richard Collinson Inl. and Storkerson P. (Aiken et al. 2007). Thannheiser et al. (2001) additionally recorded it from the head of Minto Inl. Newly recorded from Kuujjua R. and “Oldenburg L.”. Elsewhere in the Canadian Arctic recorded primarily from the western and northern islands and scattered mainland sites (Porsild and Cody 1980, Aiken et al. 2007, Saarela et al. 2013, Blondeau 2015a). Plants from the High Arctic rarely flower and most collections are sterile, while those from the southern part of its range (e.g., Cambridge Bay) frequently flower and most collections include flowers.

**NORTHWEST TERRITORIES. Boot Inl.**: *Gillespie et al. 9567* (ALA, ari, CAN, O), *9710* (CAN). **Burns L. (S)**: *Edlund 568* (CAN). **Kuujjua R.**: *Gillespie et al. 10007* (CAN). “**Oldenburg L.**”: *Oldenburg 45-1342* (CAN). **Natkusiak P.**: *Edlund 86* (CAN). **Richard Collinson Inl.**: *Stretton 220* (DAO). **NUNAVUT. Cambridge Bay**: *Bennett et al. 13-0252* (ALA, BABY, chars, od, UBC), *Edlund & Argus 12669* (CAN), *Gillespie et al. 8480* (CAN, O), *Oldenburg 44-954* (CAN), *Polunin s.n.* (CAN, 3 sheets), *Stephens 1049*, *1265* (CAN, KSTC), *1063*, *1123*, *1125* (CAN). **Collinson P.**: *Edlund & Argus 12769* (ALA, CAN). **Ferguson L. [Tahiryuaq**]: *Edlund & Argus 12775* (ALA, CAN). **Greiner L.**: *Ponomarenko VI-104*, *VI-123*, *VI-174*, *VI-203M* (CAN). **Hadley B.**: *Edlund 44*, *70*, *106* (CAN). **Mt. Lady Pelly [Amaaqtuq**]: *Hainault 1841* (DAO), *Jones 31* (DAO). **Namaycush L.**: *Edlund 11* (CAN). **Natkusiak P.**: *Edlund 335* (CAN). **Storkerson P.**: *Edlund 219* (CAN).

### *Honckenya* Ehrh. [1]

***Honckenya peploides*** subsp. ***diffusa*** (Hornem.) Hultén (Arenaria peploides var. diffusa Hornem.), Figs 58F, 59G–Seabeach sandwort | Circumpolar

Previously recorded from Albert Edward B., Anderson B., Cambridge Bay, C. Wollaston, the head of Minto Inl., Read I. (Porsild obs., conf.) and Ulukhaktok (Simmons 1913, Porsild 1957, 1964, Aiken et al. 2007). Thannheiser et al. (2001) additionally recorded it from Johansen B. (conf.). Newly recorded from Boot Inl., Kuujjua R., Murray Pt., Oterkvik Pt., Sinclair Cr. and Walker B. Elsewhere in the Canadian Arctic recorded from Baffin, Banks, Coats, King William, Prince Charles, Salisbury and Southampton islands and numerous mainland sites along the coast (Porsild and Cody 1980, Korol 1992, Aiken et al. 2007, Saarela et al. 2013, Blondeau 2015a, Saarela et al. 2017b). This species is restricted to seashores.

**NORTHWEST TERRITORIES. Boot Inl.**: *Gillespie et al. 9645* (CAN, O). **C. Wollaston**: *Edlund 23* (CAN). **Kuujjua R.**: *Gillespie et al. 9916* (ALA, ALTA, ari, CAN, MO, O, UBC, US, WIN), *Oldenburg 54-221* (GH). **Minto Inl. (head)**: *Gillespie et al. 10192* (ALA, CAN, O), *Porsild 17383* (CAN). **Ulukhaktok**: *Edlund 500*, *785* (CAN). **Walker B.**: *Oldenburg 45-1501* (CAN). **NUNAVUT. Albert Edward B.**: *Edlund & Argus 12785* (CAN). **Anderson B.**: *Edlund & Argus 12708* (CAN). **Cambridge Bay**: *Bennett 14-0327* (CAN, UBC), *13-0243* (ALA, BABY, chars), *Gillespie et al. 8476* (CAN), *Stephens 1273* (CAN). **Johansen B.**: *Gillespie et al. 8008* (ALA, ALTA, BABY, CAN, MT, O, UBC, US), *8097* (ALA, CAN, MT, O, UBC). **Murray Pt.**: *Gillespie et al. 8199* (CAN, O). **Oterkvik Pt.**: *Gillespie et al. 7629* (CAN). **Read I.***Oldenburg 42-497* (CAN), *43-1084* (CAN, GH), *Ross 9A* (GH). **Sinclair Cr.**: *Gillespie et al. 8241* (ALA, CAN, MT, O).

### *Sabulina* Rchb. [4]

**Key to *Sabulina* [adapted from Wolf et al. (1979) and Rabeler et al. (2005)]:**

**Table:** 

1	Leaves stiff, 3-nerved (visible in marcescent leaves); stems and pedicels stipitate-glandular	***S. rubella***
–	Leaves 1-nerved or nerves not apparent; stems and pedicels glabrous	**2**
2	Inflorescences 2–3(–5)-flowered cymes or occasionally flowers solitary	***S. stricta***
–	Inflorescences only with flowers solitary	**3**
3	Leaves imbricate, 2–4 mm; sepals oblong-ovate, 1.5–2.5 mm, 1-nerved; petals obovate to spatulate, 1.5–2× as long as sepals; dense cushion plants with flowers absent or few to abundant	***S. rossii***
–	Leaves spreading to ascending, 3–10 mm; sepals ovate to lanceolate, 2–4 mm, 3-nerved; petals oblong to obovate, 0.8–1× as long as sepals; loosely caespitose plants with flowers mostly abundant	***S. elegans***

***Sabulina elegans*** (Cham. & Schltdl.) Dillenb. & Kadereit (*Arenaria elegans* Cham. & Schltdl., *Minuartia elegans* (Cham. & Schltdl.) Schischk.), Fig. 58G–Elegant stitchwort | Amphi-Beringian–Cordilleran

Previously reported on Victoria I. only from Ulukhaktok (Aiken et al. 2007). We tentatively accept the single collection from that locality (*Edlund 880*) as this species, with the caveat that some characters appear intermediate towards *S. rossii*. Elsewhere in the Canadian Arctic recorded from western mainland sites as far east as the Coppermine R., Nunavut (Wolf et al. 1979, Aiken et al. 2007, Saarela et al. 2013, Saarela et al. 2017b).

**NORTHWEST TERRITORIES. Ulukhaktok**: *Edlund 880* (CAN).

***Sabulina rossii*** (R.Br. ex Richardson) Dillenb. *&* Kadereit (*Minuartia rossii* R.Br. ex Richardson) Graebn.), Figs 58H, 59H–Ross’s stitchwort | Amphi-Beringian (E)–North American (N)–amphi-Atlantic (W)

Previously recorded from Cambridge Bay, the head of Prince Albert S., Ulukhaktok and northwestern Wollaston P. (Porsild 1955, Wolf et al. 1979, Porsild and Cody 1980, Aiken et al. 2007). Thannheiser et al. (2001) additionally recorded it from Hadley B. (conf.), Johansen B., the head of Minto Inl. (conf.) and Surrey L. Newly recorded from Anderson B., Boot Inl., C. Wollaston, Clouston B., Falaise B., Greiner L., Kuujjua R., Mt. Bumpus, Mt. Pelly, Namaycush L., Natkusiak P. and Tahiryuaq. Many of these records close the conspicuous distribution gap on northwestern Victoria I. in published maps (Wolf et al. 1979, Aiken et al. 2007). Elsewhere in the Canadian Arctic widespread across the western and northern islands and known from scattered mainland sites (Wolf et al. 1979, Porsild and Cody 1980, Cody and Reading 2005, Aiken et al. 2007, Saarela et al. 2013, Blondeau 2015a, Saarela et al. 2017b).

**NORTHWEST TERRITORIES. Boot Inl.**: *Gillespie et al. 9564* (ari, CAN). **C. Wollaston**: *Edlund 38* (CAN). **Kuujjua R.**: *Edlund 547*, *661* (CAN), *Gillespie et al. 9806*, *9879* (CAN), *9892* (ALA, ari, CAN), *9881* (CAN, O). **Minto Inl. (head)**: *Edlund 94* (CAN), *Gillespie et al. 10258* (CAN, O). **Natkusiak P.**: *Edlund 108* (CAN). **Prince Albert S. (head)**: *Edlund 377*, *394* (CAN), *Porsild 17384*CAN). **Tahiryuaq**: *Edlund 393* (CAN). **Ulukhaktok**: *Edlund 467*, *539*, *912* (CAN), *Porsild 17278* (CAN). **Wollaston P. (NW)**: *Porsild 17216* (CAN). **NUNAVUT. Anderson B.**: *Edlund & Argus 12721* (CAN). **Cambridge Bay**: *Bennett et al. 13-0202* (BABY, chars), *Stephens 1157* (CAN, KSTC), *Tasker s.n.* (CAN). **Clouston B.**: *Gillespie et al. 7723* (CAN). **Falaise B.**: *Eriksen et al. 992* (ALA). **Greiner L.**: *Ponomarenko VI-175*, *VI-183*, *VI-220A*, *VI-304* (CAN). **Hadley B.**: *Edlund 155* (CAN). **Mt. Bumpus**: *Edlund 252* (CAN). **Ovayok TP**: *Gillespie et al. 8434* (CAN, O), *Gould s.n.* (ALA), *Stephens 1064* (CAN, KSTC). **Namaycush L.**: *Edlund & Roncato-Spencer 119* (CAN).

**Figure 60.**
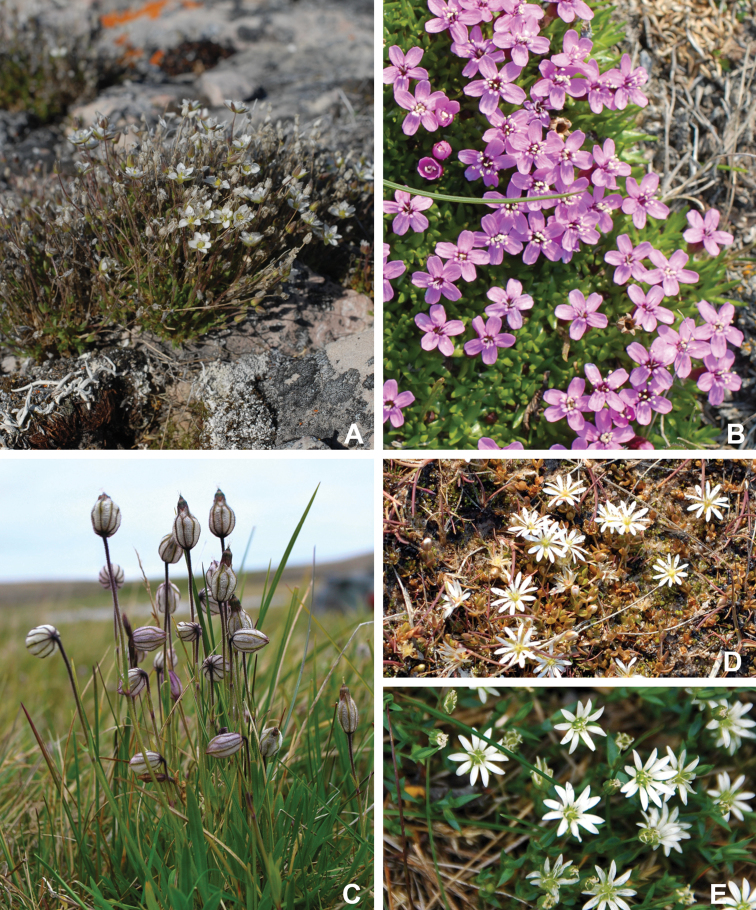
**A***Sabulina rubella* habit, *Gillespie et al. 7591***B***Silene acaulis* habit, Johansen Bay, NU, 20 July 2008 **C**Silene uralensis subsp. arctica habit **D***Stellaria humifusa* habit, *Gillespie et al. 8200***E***Stellaria longipes* habit, Johansen Bay, NU, 18 July 2008. Photos **A**, **B**, **D**, **E** by R.D. Bull and **C** by B.A. Bennett.

***Sabulina rubella*** (Wahlenb.) Dillenb. *&* Kadereit (*Minuartia rubella* (Wahl.) Hiern), Figs 58I, 60A–Reddish stitchwort | Circumpolar–alpine

Previously recorded from south of Burns L., Cambridge Bay, Hadley B., the head of Minto Inl., Namaycush L., the head of Prince Albert S., Read I., Ulukhaktok, Washburn L. and Wollaston P. (Porsild 1955, 1957, 1964, Porsild and Cody 1980, Aiken et al. 2007). Newly recorded from Boot Inl., Ferguson L., Greiner L., Johansen B., Kuujjua R., Mt. Bumpus, Mt. Pelly, “Oldenburg L.”, Oterkvik Pt., Natkusiak P., the north side of Prince Albert S., Sinclair Cr. and Walker B. Widespread across the Canadian Arctic Archipelago and mainland (Porsild and Cody 1980, Aiken et al. 2007, Saarela et al. 2013, Blondeau 2015a, Saarela et al. 2017b).

**NORTHWEST TERRITORIES. Boot Inl.**: *Gillespie et al. 9568*, *9699* (CAN). **Burns L. (S)**: *Edlund 569*, *569B* (CAN). **Kuujjua R.**: *Gillespie et al. 9848*, *9897*, *9972* (CAN). **Minto Inl. (head)**: *Gillespie et al. 10018* (ALA, ari, CAN), *10075b*, *10085b*, *10175* (CAN), *10085* (CAN, O), *Porsild 17385* (CAN). **Natkusiak P.**: *Edlund 91*, *92*, *144*, *145* (CAN). “**Oldenburg L.**”: *Oldenburg 45-1344* (CAN). **Prince Albert S. (N)**: *Oldenburg 46-2284* (CAN). **Prince Albert S. (head)**: *Porsild 17438* (CAN). **Ulukhaktok**: *Edlund 328*, *466*, *538*, *787*, *889*, *890*, *891* (CAN), *Oldenburg 42-97A*, *45-1700*, *45-1701* (CAN), *Porsild 17279* (CAN). **Walker B.**: *Oldenburg 45-1536A* (CAN). **NUNAVUT. Cambridge Bay**: *Bennett & Sullivan 13-0292* (DAO), *Bennett 13-0613* (UBC), *13-0166* (ALA), *13-0250* (BABY, UBC, WTU), *13-0533* (chars, od), *13-0624* (UBC), *14-0663* (DAO), *Edlund & Argus 12889B* (CAN), *Polunin s.n.* (CAN), *Ponomarenko VI-075* (CAN), *Stephens 993*, *1113*, *1239* (CAN, KSTC), *Tasker 2443* (CAN). **Falaise B.**: *Eriksen et al. 984* (ALA). **Ferguson L. [Tahiryuaq**]: *Bennett et al. 14-0407* (CAN). **Greiner L.**: *Ponomarenko VI-036*, *VI-117* (CAN). **Hadley B.**: *Edlund 75*, *140*, *s.n.* (CAN). **Johansen B.**: *Gillespie et al. 7895*, *7923* (CAN), *8076* (CAN, O). **Mt. Bumpus**: *Edlund 267* (CAN, US). **Ovayok TP**: *Gillespie et al. 8439* (CAN, O). **Namaycush L.**: *Edlund & Roncato-Spencer 85* (CAN). **Oterkvik Pt.**: *Gillespie et al. 7525*, *7666* (CAN), *7591* (ALA, CAN, MT, O). **Read I.**: *Oldenburg 43-928* (CAN), *Porsild 17193* (CAN). **Sinclair Cr.**: *Gillespie et al. 8265* (ALA, CAN, O), *8323* (CAN). **Storkerson P.**: *Edlund 225* (CAN). **Washburn L.**: *Porsild 17457* (CAN). **Wollaston P. (NW)**: *Porsild 17217* (CAN).

***Sabulina stricta*** (Sw.) Rchb. (*Minuartia stricta* (Sw.) Hiern), Fig. 58J–Bog stitchwort | Circumpolar-alpine

Known from a single collection from the Johansen B. area; see details in Gillespie et al. (2015). Elsewhere in the Canadian Arctic recorded from northern Quebec and Labrador, scattered Nunavut and Northwest Territories mainland sites, and Baffin and Southampton islands (Porsild and Cody 1980, Aiken et al. 2007, Saarela et al. 2013, Saarela et al. 2017b). The general status rank of this species in Northwest Territories is Sensitive (Working Group on General Status of NWT Species 2016); there are no records from the Northwest Territories portion of Victoria I.

**NUNAVUT. Johansen B.**: *Gillespie et al. 7966* (ALA, CAN, MT, O).

### *Sagina* L. [2]

**Key to *Sagina* [adapted from Crow (2005)]:**

**Table:** 

1	Flowers 5-merous, sometimes accompanied by 4-merous flowers; petals 2.5–3 mm, longer than or rarely equaling sepals,; primary basal rosette of leaves absent, secondary rosettes of linear leaves often present	***S. caespitosa***
–	Flowers 4-merous, sometimes accompanied by 5-merous flowers; petals 1.5–2 mm, shorter than or equaling sepals; primary basal rosette of fleshy, subulate leaves present, secondary rosettes absent	***S. nivalis***


***Sagina caespitosa*** Lange, Fig. 58K–Tufted pearlwort | Amphi-Atlantic

Previously recorded from Anderson B., the only record for the island and the western limit for the species (Aiken et al. 2007). Elsewhere in the Canadian Arctic recorded from Baffin and Southampton islands, northern Quebec and Labrador, and mainland Nunavut (Crow 1978, Porsild and Cody 1980, Aiken et al. 2007).

**NUNAVUT. Anderson B.**: *Edlund & Argus 12710* (CAN).

***Sagina nivalis*** (Lindblom) Fr. (*S. intermedia* Fenzl), Fig. 58L–Snow pearlwort | Circumpolar

Previously recorded from the head of Minto Inl., the head of Prince Albert S., Storkerson P. and Ulukhaktok (Porsild obs.) (Porsild 1955, 1957, 1964, Crow 1978, Porsild and Cody 1980, Aiken et al. 2007). Thannheiser et al. (2001) additionally recorded it from Hadley B. Newly recorded from Cambridge Bay. Elsewhere in the Canadian Arctic recorded from scattered sites on Axel Heiberg, Baffin, Banks, Cornwallis, Devon, Ellesmere, Melville and Smith islands and the mainland (Crow 1978, Porsild and Cody 1980, Aiken et al. 2007).

**NORTHWEST TERRITORIES. Minto Inl. (head)**: *Porsild 17388* (CAN). **Prince Albert S. (head)**: *Porsild 17439* (CAN). **NUNAVUT. Cambridge Bay**: *Bennett et al. 13-0319* (BABY, CAN, chars), *14-0315* (CAN), *Polunin s.n.* (cf.) (CAN). **Storkerson P.**: *Edlund 180* (CAN).

### *Silene* L. [4/6]

**Key to *Silene* [adapted from Morton (2005b), Aiken et al. (2007) and Elven et al. (2011)]**

**Table:** 

1	Plants mat- or cushion-forming, 2–5 cm high, petals bright pink, rarely white, styles 3	***S. acaulis***
–	Plants tufted, 4–30 cm high; petals white to pink or dusky purple-red; styles 5	**2**
2	Calyces inflated; petals dusky purple-red; flowers nodding (*S. uralensis*)	**3**
–	Calyces not inflated; petals white, pink or purple-tinged; flowers erect	**4**
3	Calyces strongly inflated, in fruit becoming globose or broader than long; petals much emerging from calyx	**S. uralensis subsp. arctica**
–	Calyces weakly inflated, in fruit usually longer than broad; petals slightly emerging from the calyx	**S. uralensis subsp. uralensis**
4	Calyces elliptic to campanulate; seeds not winged, 0.6–1 mm wide; capsules slightly longer than calyx	***S. ostenfeldii***
–	Calyces campanulate or ovate; seeds winged, 1–1.5 mm wide, wing to ½ seed diam.; capsules equalling calyx (*S. involucrata*)	**5**
5	Calyces 10–20 mm in fruit; flowering stems sturdy, usually < 20 cm, internodes equaling or shorter than leaves	**S. involucrata subsp. involucrata**
–	Calyces 8–10(–12) mm in fruit; flowering stems slender, usually > 30 cm, internodes longer than leaves	**S. involucrata subsp. tenella**

**Figure 61.**
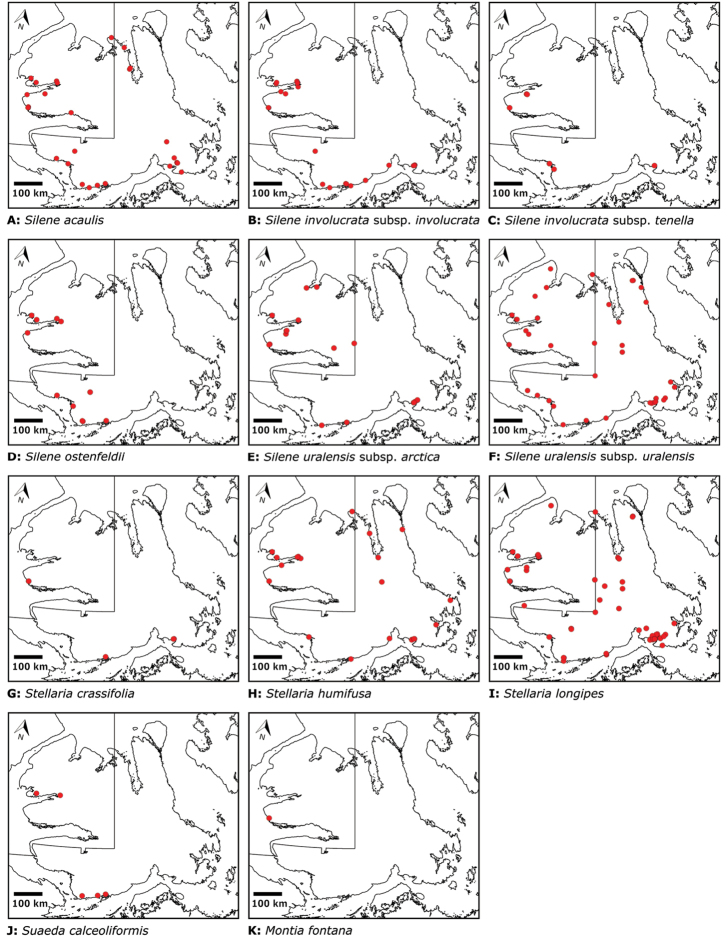
Species distribution maps. Caryophyllaceae: **A***Silene acaulis***B**Silene involucrata subsp. involucrata**C**Silene involucrata subsp. tenella**D***Silene ostenfeldii***E**Silene uralensis subsp. arctica**F**Silene uralensis subsp. uralensis**G***Stellaria crassifolia***H***Stellaria humifusa***I***Stellaria longipes*. Amaranthaceae: **J***Suaeda calceoliformis*. Montiaceae: **K***Montia fontana*.

***Silene acaulis*** (L.) Jacq., Figs 61A, 60B–Moss campion | Amphi-Beringian–North American–amphi-Atlantic–European (N/C)–Asian (NW)

Previously recorded from Cambridge Bay, C. Wollaston, the head of Minto Inl., Mt. Pelly, Natkusiak P., the north side of Prince Albert S., Read I. (Porsild obs., conf.), Ulukhaktok and Wollaston P. (Simmons 1913, Macoun and Holm 1921, Porsild and Cody 1980, Aiken et al. 2007). Newly recorded from Anderson B., Boot Inl., Falaise B., Hadley B., Johansen B., Mt. Lady Pelly, Oterkvik Pt., Sinclair Cr. and “Trunsky L.” Widespread throughout the Canadian Arctic (Porsild and Cody 1980, Cody et al. 1989, Korol 1992, Saarela et al. 2013, Blondeau 2015a, Saarela et al. 2017b).

**NORTHWEST TERRITORIES. Boot Inl.**: *Gillespie et al. 9528* (ALA, CAN, MT). **C. Wollaston**: *Edlund 50* (CAN). **Kuujjua R.**: *Gillespie et al. 9766* (ALA, ari, CAN, UBC). **Minto Inl. (head)**: *Edlund 128* (CAN), *Gillespie et al. 9464* (CAN, O). **Natkusiak P.**: *Edlund 85* (CAN). **Prince Albert S. (N)**: *Stretton 42* (DAO). **Ulukhaktok**: *Bandringa 313* (CAN), *Edlund 320* (CAN), *Oldenburg 42-8* (CAN, GH), *45-1698* (CAN), *Porsild 17282* (CAN), *Ross 34* (ALTA), *34A* (GH), *Saarela & Bull 1434* (CAN, O). **Walker B.**: *Oldenburg 45-1541* (CAN). **NUNAVUT. Anderson B.**: *Edlund & Argus 12713* (CAN). **Cambridge Bay**: *Bennett & Sullivan 13-0294* (chars, V), *Bennett et al. 13-0215* (od). **Falaise B.**: *Eriksen et al. 961* (ALA). **Hadley B.**: *Edlund 47*, *s.n.* (CAN). **Johansen B.**: *Gillespie et al. 7821* (CAN, V, Z), *8092* (CAN, O). **Mt. Lady Pelly [Amaaqtuq**]: *Hainault 1807* (DAO). **Ovayok TP**: *Gould s.n.* (ALA), *Stephens 1053*, *1168* (CAN). **Natkusiak P.**: *Edlund 333* (CAN). **Oterkvik Pt.**: *Gillespie et al. 7496* (CAN, O, Z, V), *7701* (CAN). **Read I.**: *Oldenburg 42-522*, *43-1066*, *43-915* (CAN), *Ross 12A* (GH). “**Trunsky L.**”: *Bennett et al. 14-0396* (BABY, CAN, chars, UBC). **Wollaston P.**: *D. Jenness 656* (CAN).

***Silene involucrata*** (Cham. *&* Schltdl.) Bocquet subsp. ***involucrata*** (*Melandrium affine* (J.Vahl ex Fr.) J.Vahl), Fig. 61B–Arctic catchfly | Circumpolar

The species was previously recorded from Cambridge Bay, the head of Minto Inl., Read I., Wollaston P., Ulukhaktok (Simmons 1913, Porsild 1955, 1957, 1964, Porsild and Cody 1980, Aiken et al. 2007); none of these authors recognized infraspecific taxa. Newly recorded from “30-Mile Cr.”, Boot Inl., Kuujjua R., Johansen B., Murray Pt., Oterkvik Pt. and Sinclair Cr. Elsewhere in the Canadian Arctic widespread across the islands (Aiken et al. 2007). There are taxonomic problems in the complex (Elven et al. 2011); we follow Morton (2005b).

**NORTHWEST TERRITORIES. Boot Inl.**: *Edlund 583* (CAN), *Gillespie et al.* 9624 (CAN). **Kuujjua R.**: *Gillespie et al. 9738* (CAN), *9922* (ALA, CAN, MT, WIN). **Minto Inl. (head)**: *Edlund 119*, *158*, *601*, *68* (CAN), *Gillespie et al. 10057a*, *10069* (CAN), *10118* (ari, CAN), *10162*, *10263* (CAN, O). **Ulukhaktok**: *Bandringa* 341 (CAN, UBC), *Edlund 325*, *336* (CAN), *Oldenburg 45-1704* (CAN, GH), *Porsild 17281* (ALTA, CAN), *Ross 23* (ALTA), *23A* (GH), *Saarela & Bull 1427* (CAN, O). **NUNAVUT. “30-Mile Cr.**”: *Bennett et al. 14-0348* (CAN). **Cambridge Bay**: *Edlund & Argus 12897* (CAN), *Oldenburg 44*-*904* (CAN), *Polunin s.n.* (CAN), *Porsild 21613* (CAN), *Stephens 983*, *1032*, *1112* (KSTC), *1056*, *1111*, *1240* (CAN, KSTC), *Stephens 994* (CAN). **Johansen B.**: *Gillespie et al. 7823* (ALA, CAN, MT, O), *7978* (CAN). **Murray Pt.**: *Gillespie et al. 8181* (CAN). **Oterkvik Pt.**: *Gillespie et al. 7594* (ALA, CAN, MT, O), *7700* (CAN). **Sinclair Cr.**: *Gillespie et al.* 8326, *8334* (ALA, CAN, O), *8327* (CAN). **Wollaston P.**: *D. Jenness 658*, *658a* (CAN).

***Silene involucrata*** subsp. ***tenella*** (Tolm.) Bocquet, Fig. 61C–Taylor’s arctic catchfly | European (NE)–Asian (N)–amphi-Beringian

This is a boreal-low Arctic taxon, known from Cambridge Bay, Clouston B., Kuujjua R., Read I. and Ulukhaktok. Elsewhere in the Canadian Arctic recorded from Banks I. and mainland sites, based on recently-revised specimens at CAN following taxonomy of Morton (2005b), including those reported in Saarela et al. (2017b).

**NORTHWEST TERRITORIES. Kuujjua R.**: *Gillespie et al. 9993* (CAN), *9737* (ALA, CAN, O). **Ulukhaktok**: *Ross 23* (ALTA). **NUNAVUT. Cambridge Bay**: *Oldenburg 44*-*905* (CAN). **Clouston B.**: *Gillespie et al. 7731* (CAN, O). **Read I.**: *Oldenburg 42-526*, *43-910*, *43-953* (CAN).

***Silene ostenfeldii*** (A.E.Porsild) J.K.Morton (*Melandrium ostenfeldii* A.E.Porsild), Fig. 61D–Ostenfeld’s catchfly | Asian (NE)–amphi-Beringian

Previously recorded from Walker B. (Porsild 1955, 1957, 1964, Porsild and Cody 1980, Aiken et al. 2007). Of the two “additional records from Porsild and Cody (1980)” mapped in Aiken et al. (2007), the one from the Ulukhaktok area is likely a mis-interpretation of the nearby Walker B. site, as we are not aware of any collections from Ulukhaktok, and we have not seen a supporting voucher for the other one, from southwestern Victoria I. Thannheiser et al. (2001) additionally recorded it from the head of Minto Inl. (conf.), Johansen B. (conf.) and Richardson I. Newly recorded from Boot Inl., C. Wollaston, Falaise B., Mt. Bumpus and Oterkvik Pt. The Victoria I. occurrences mark the eastern limit of the species, which is otherwise known in the Canadian Arctic from Banks I. and western mainland sites (Porsild and Cody 1980, Aiken et al. 2007, Saarela et al. 2013).

**NORTHWEST TERRITORIES. Boot Inl.**: *Gillespie et al. 9530* (ALA, CAN, O). **C. Wollaston**: *Edlund 159* (CAN). **Minto Inl. (head)**: *Gillespie et al. 10063* (CAN, O), *Porsild 17387* (CAN). **Walker B.**: *Porsild 17492* (CAN). **NUNAVUT. Clouston B.**: *Gillespie et al. 7756* (ALA, BABY, CAN, MT, O, UBC). **Falaise B.**: *Eriksen et al. 979* (ALA). **Johansen B.**: *Gillespie et al. 7976* (ALA, CAN, O). **Mt. Bumpus**: *Edlund 237*, *262* (CAN). **Oterkvik Pt.**: *Gillespie et al. 7527* (CAN), *7586* (ALA, CAN, MT, O).

***Silene uralensis*** subsp. ***arctica*** (Th.Fr.) Bocquet (Melandrium apetalum subsp. arcticum (Fr.) Hultén), Figs 61E, 60C–Arctic nodding catchfly | Circumpolar

Aiken et al. (2007) recognized all plants from the Canadian Arctic Archipelago as S. uralensis subsp. arctica, whereas we find the majority of collections from Victoria I. to be S. uralensis subsp. uralensis. Subspecies arctica is known from south of Burns L., Cambridge Bay, Kuujjua R., the head of Minto Inl., Richard Collinson Inl., Tahiryuaq, Ulukhaktok and Walker B.. Its full range in the Canadian Arctic is unclear as all relevant herbarium material has not yet been determined following the taxonomy proposed by Elven et al. (2011); those authors state that subsp. arctica is the common taxon in the Canadian Arctic Archipelago. It is, however, known from mainland sites adjacent to Victoria I. (Saarela et al. 2013, Saarela et al. 2017b).

**NORTHWEST TERRITORIES. Burns L. (S)**: *Edlund 66* (CAN). **Kuujjua R.**: *Edlund 655* (CAN), *Gillespie et al. 9825* (CAN), *9839* (ari, CAN). **Minto Inl. (head)**: *Edlund 55* (CAN). **Richard Collinson Inl.**: *Edlund 703* (CAN), *Stretton 216* (DAO). **Tahiryuaq**: *Edlund 384* (CAN). **Ulukhaktok**: *Edlund 887*, *888* (CAN). **Walker B.**: *Porsild 17491* (CAN). **NUNAVUT. Cambridge Bay**: *Bennett 13-0226* (ALA, CAN, chars, UBC), *Calder et al. 24196* (DAO), *Edlund & Argus 12642* (CAN), *Gould s.n.* (ALA), *Porsild 21614* (CAN). **Johansen B.**: *Gillespie et al. 8011* (CAN, O). **Ovayok TP**: *Gillespie et al. 8431* (ALA, CAN, O). **Oterkvik Pt.**: *Gillespie et al. 7716* (ALA, CAN, MT, O).

***Silene uralensis*** (Rupr.) Bocquet subsp. ***uralensis*** (*Melandrium apetalum* (L.) Fenzl, M. apetalum subsp. arcticum (Fr.) Hultén), Fig. 61F–Nodding catchfly | European (NE)–Asian (N)–amphi-Beringian–North American (N)

Taxonomy of *Silene uralensis* follows Elven et al. (2011), not Morton (2005b); the latter included the two subspecies recognized here, as well as a third, in a more broadly circumscribed subsp. uralensis. Aiken et al. (2007) recognized all plants from the Canadian Arctic Archipelago as S. uralensis subsp. arctica, whereas our identifications indicate that both this and subsp. uralensis occur on Victoria I., as Elven et al. (2011) also found. The species was previously recorded from Cambridge Bay, Hadley B., Kuujjua R., the head of Minto Inl., the head of Prince Albert S., Read I. (Porsild obs., confirmed), Richard Collinson Inl., Storkerson P., Ulukhaktok and Walker B. (Simmons 1913, Porsild 1955, 1957, 1964, Porsild and Cody 1980, Aiken et al. 2007). Thannheiser et al. (2001) additionally recorded it from Johansen B. (conf.) and Surrey L. Newly recorded from Albert Edward B., Boot Inl., Clouston B., Falaise B., Greiner L., Mt. Pelly, Namaycush L., Natkusiak P., Oterkvik Pt., the north side of Prince Albert S., an inland site on Prince Albert P., Sinclair Cr., Tahiryuaq and Washburn L. Subspecies uralensis is known from all but two (south of Burns L., Mt. Pelly) of these areas. Elsewhere in the Canadian Arctic subsp. uralensis is known from Banks and Baffin islands as well as mainland sites (Elven et al. 2011, Saarela et al. 2017b). Its distribution will be clarified pending revision of herbarium material.

**NORTHWEST TERRITORIES. Boot Inl.**: *Gillespie et al. 9516* (CAN, O), *9566* (ari, CAN), *9689* (ALA, CAN, O). **Kuujjua R.**: *Gillespie et al. 9969*, *9802* (CAN, O). **Minto Inl. (head)**: *Gillespie et al. 9496*, *10057b* (CAN). **Natkusiak P.**: *Edlund 109* (CAN). “**Oldenburg L.**”: *Oldenburg 45-1351* (CAN). **Prince Albert S. (N)**: *Oldenburg 46-2286* (CAN). **Prince Albert P.**: *Oldenburg 54*-*642* (UBC). **Richard Collinson Inl.**: *Edlund 600* (CAN). **Tahiryuaq**: *Edlund 159* (CAN). **Ulukhaktok**: *Edlund 353* (CAN), *Oldenburg 42-73C*, *45-1703*, *45-1705*, *45-1706* (CAN). **Walker B.**: *Oldenburg 45-1534A* (CAN). **NUNAVUT. Albert Edward B.**: *Ponomarenko VI-249*, *VI*-*259* (CAN). **Cambridge Bay**: *Edlund & Argus 12889A* (CAN), *Gillespie et al. 8383* (ALA, CAN, O), *Gillespie et al. 8453* (CAN), *Oldenburg 44*-*903*, *44*-*904a* (CAN), *Polunin s.n.* (CAN, 2 sheets), *Ponomarenko VI-053*, *VI-313* (CAN), *Stephens 942*, *1030*, *1214*, *1215*, *1216* (CAN, KSTC), *1031*, *1078*, *1165* (KSTC). **Clouston B.**: *Gillespie et al. 7720* (CAN). **Falaise B.**: *Eriksen et al. 959* (ALA). **Greiner L.**: *Ponomarenko VI-042*, *VI-184*, *VI*-286, *VI-294B* (CAN). **Hadley B.**: *Edlund 43*, *159* (CAN). **Johansen B.**: *Gillespie et al. 7871* (CAN), *8046* (ALA, ALTA, BABY, CAN, MT, O, UBC). **Namaycush L.**: *Edlund 144*, *145*, *146* (CAN). **Oterkvik Pt.**: *Gillespie et al. 7638* (CAN). **Prince Albert S. (head)**: *Edlund 79* (CAN). **Read I.***Oldenburg 43-1068*, *43-1069*, *44*-*1037* (CAN). **Sinclair Cr.**: *Gillespie et al. 8289* (CAN). **Storkerson P.**: *Edlund 170*, *241*, *283*, *307* (CAN). **Washburn L.**: *Oldenburg 46-2179* (CAN). **Wollaston P.**: *D. Jenness 657* (CAN).

### *Stellaria* L. [3]

**Key to *Stellaria* [adapted from Porsild and Cody (1980) and Morton (2005c)]**

**Table:** 

1	Leaf blades firm, thin to coriaceous, not succulent, often glaucous, keeled, with midrib prominent	***S. longipes***
–	Leaf blades soft, succulent or ± succulent, not glaucous, not keeled, with midrib obscure	**2**
2	Sepals narrowly lanceolate-triangular, prominently 3-veined, 3–3.5(–4) mm; petals 2.5–5 mm; pedicels 3–40 mm, sharply angled below the capsule; capsules longer than sepals; seeds rugose; plants delicate, slender, straggling or loose tangled mats, fresh green, of wet meadows	***S. crassifolia***
–	Sepals lanceolate, 1–3-veined, 4–5 mm; petals 4–6 mm; pedicels 5–10 mm, not sharply angled below the capsule; capsules equal to sepals; seeds smooth to slightly rugose; plants low mats, often pinkish, of seashores	***S. humifusa***

***Stellaria crassifolia*** Ehrh., Fig. 61G–Thick-leaved starwort | Circumboreal-polar

Previously recorded from Cambridge Bay and Ulukhaktok (Porsild 1955, 1957, 1964, Porsild and Cody 1980, Aiken et al. 2007). Newly recorded from Johansen B. Elsewhere in the Canadian Arctic recorded from scattered sites on Baffin, Ellesmere, Melville, Prince Charles and Southampton islands and across the mainland (Porsild and Cody 1980, Cody et al. 2003, Aiken et al. 2007, Blondeau 2015a, Saarela et al. 2017b).

**NORTHWEST TERRITORIES. Ulukhaktok**: *Edlund 327*, *507*, *819*, *911* (CAN), *Porsild 17283* (CAN). **NUNAVUT. Cambridge Bay**: *Stephens 1154* (CAN), *1156*, *1204* (CAN, KSTC). **Johansen B.**: *Gillespie et al. 7920* (CAN, O).

***Stellaria humifusa*** Rottb., Figs 61H, 60D–Salt-marsh starwort | Circumpolar–amphi-Pacific

Previously recorded from Albert Edward B., Cambridge Bay (Porsild obs., conf.), Collinson P., Hadley B., the head of Minto Inl., Natkusiak P., head of Prince Albert Sound (Porsild obs.), Read I. (Porsild obs., conf.), Storkerson P. and Ulukhaktok (Porsild 1955, 1957, Porsild and Cody 1980, Aiken et al. 2007). Newly recorded from “30-Mile Cr.”, Boot Inl., Kuujjua R., Murray Pt., Walker B. and Washburn L. Widespread throughout the Canadian Arctic (Porsild and Cody 1980, Korol 1992, Aiken et al. 2007, Saarela et al. 2013, Blondeau 2015a).

**NORTHWEST TERRITORIES. Boot Inl.**: *Gillespie et al. 9634* (ari, CAN, O). **Kuujjua R.**: *Gillespie et al. 9919* (CAN, O). **Minto Inl. (head)**: *Edlund 152* (CAN), *Gillespie et al. 10194* (CAN), *10246* (ALA, CAN, MT, O). **Natkusiak P.**: *Edlund 71* (CAN). **Ulukhaktok**: *Edlund 317* (CAN). **Walker B.**: *Oldenburg 45-1540A* (CAN). **NUNAVUT. “30-Mile Cr.**”: *Bennett et al. 14-0358* (CAN, DAO, UBC). **Albert Edward B.**: *Edlund & Argus 12786* (CAN). **Cambridge Bay**: *Bennett et al. 13-0277* (BABY, chars, od, UBC), *Gillespie et al. 8475* (ALA, CAN, O), *Polunin s.n.* (CAN, 3 sheets). **Collinson P.**: *Edlund & Argus 12756* (CAN). **Hadley B.**: *Edlund 121* (CAN). **Murray Pt.**: *Gillespie et al. 8200* (ALA, CAN, O). **Natkusiak P.**: *Edlund 336* (CAN). **Read I.**: *Oldenburg 42-496*, *43-1079*, *43-929* (CAN). **Storkerson P.**: *Edlund 297* (CAN). **Washburn L.**: *Oldenburg 46-2182* (CAN).

***Stellaria longipes*** Goldie (*S. arenicola* Raup, *S. stricta* Richardson, *S. subvestita* Greene, *S. crassipes* Hultén, *S. monantha* Hultén, *S. edwardsii* R.Br., S. laeta *Richardson*), Figs 61I, 60E–Long-stalked starwort | Circumboreal-polar

Previously recorded from south of Burns L., Cambridge Bay, Hadley B., “Long L.”, Namaycush L., the north side of Prince Albert Sound, Read I., Storkerson P., Ulukhaktok, Washburn L. and Wollaston P. (Porsild obs.) (Simmons 1913, Porsild 1955, 1957, 1964, Porsild and Cody 1980, Aiken et al. 2007). Thannheiser et al. (2001) additionally recorded it from Johansen B., Surrey L., Wellington B. and Mt. Pelly (conf.). Newly recorded from Anderson B., Boot Inl., Greiner L., Kuujjua R., Natkusiak P., “Oldenburg L.”, Oterkvik Pt., Sinclair Cr., Tahiryuaq and Wollaston P. Widespread across the Canadian Arctic (Porsild 1955, 1957, 1964, Porsild and Cody 1980, Aiken et al. 2007, Saarela et al. 2013, Saarela et al. 2017b).

**NORTHWEST TERRITORIES. Boot Inl.**: *Gillespie et al. 9521*, *9574* (CAN), *Gillespie et al. 9585* (ALA, CAN, MT, O). **Burns L. (S)**: *Edlund 68* (CAN). **C. Wollaston**: *Edlund 20*, *22*, *s.n.* (CAN). **Kuujjua R.**: *Edlund 642* (CAN), *Gillespie et al. 9736* (CAN). **Minto Inl. (head)**: *Edlund 620*, *621*, *63* (CAN), *Gillespie et al. 9468*, *10048*, *10049* (CAN), *10050* (ALA, CAN, O), *10055* (ALA, ari, CAN, MT, O), *10070* (ALA, CAN, MT, O), *10124* (ALA, ALTA, ari, CAN, MT, O, UBC, US, WIN), *10132* (CAN, O). **Natkusiak P.**: *Edlund 88* (CAN). “**Oldenburg L.**”: *Oldenburg 45-1343* (CAN). **Prince Albert S. (head)**: *Edlund & Argus 12824* (CAN). **Tahiryuaq**: *Edlund 160* (CAN). **Ulukhaktok**: *Edlund 321*, *322*, *482*, *774*, *803*, *892*, *893* (CAN), *Oldenburg 42-26*, *42-4*, *42-70B*, *45-1699*, *45-1702* (CAN), *Porsild 17284*, *17285* (CAN), *Saarela & Bull 1418* (ALA, ari, CAN, O, UBC), *1462* (ALA, CAN, O). **Walker B.**: *Oldenburg 45-1537A* (CAN). **Wollaston P.**: *Oldenburg 54-486*, *54-487*, *54-488* (GH). **NUNAVUT. Albert Edward B.**: *Ponomarenko VI-337D* (CAN). **Anderson B.**: *Edlund & Argus 12709* (CAN). **Cambridge Bay**: *Bennett et al. 13-0253* (BABY, chars, od, UBC), *13-0260* (BABY, chars), *13-0315* (ALA, CAN, chars), *13-0559* (od), *14-0370* (UBC), *Calder et al. 24206* (DAO), *24207A* (DAO), *Edlund & Argus 12650*, *12651*, *12657*, *12658* (CAN), *Gillespie et al. 8397* (ALA, CAN, O), *Oldenburg 44-952* (CAN, GH), *44-953* (CAN), *Parker & Jonsdottir 9089* (ALA), *Polunin s.n.* (CAN, 2 sheets), *Ponomarenko VI-079*, *VI-309B* (CAN), *Stephens 1007*, *1050* (KSTC), *1054*, *1122*, *1146* (CAN, KSTC), *963*, *1121* (KSTC), *Sweatman & Smith 17* (CAN, KSTC). **Ferguson L. [Tahiryuaq**]: *Hainault 1981*, *2051*, *2083* (DAO). **Greiner L.**: *Ponomarenko VI-032*, *VI-112*, *VI-162*, *VI-211*, *VI-221*, *VI-319* (CAN). **Hadley B.**: *Edlund 95*, *131*, *157* (CAN). “**Long L.**”: *Lambert s.n.* (CAN, 2 sheets). **Mt. Bumpus**: *Edlund 143*, *196*, *215*, *260*, *275* (CAN). **Ovayok TP**: *Gillespie et al. 8438* (ALA, ALTA, BABY, CAN, MT, O, UBC), *Gould s.n.* (ALA). **Namaycush L.**: *Edlund & Roncato-Spencer 58* (CAN). **Oterkvik Pt.**: *Gillespie et al. 7526* (ALA, CAN, MT, O), *7628* (CAN, O), *Gillespie et al. 7803* (CAN, O). **Prince Albert S. (head)**: *Edlund 80* (CAN). **Read I.**: *Oldenburg 42-524* (CAN, GH), *43-1078*, *43-930* (CAN), *Porsild 17194* (CAN). **Sinclair Cr.**: *Gillespie et al. 8251* (ALA, CAN, MT, O), *8269* (CAN), *8317* (ALA, CAN, O), *8318* (ALA, ALTA, BABY, CAN, MT, O, UBC). **Storkerson P.**: *Edlund 193*, *232* (CAN). **Tuktu R.**: *Gould s.n.* (ALA). **Washburn L.**: *Edlund & Argus 12798* (CAN), *Oldenburg 46-2181* (CAN).

### Amaranthaceae [1/1]

***Suaeda* Forssk. ex J.F. Gmel. [1]**

***Suaeda calceoliformis*** (Hook.) Moq., Fig. 61J–Horned sea-blite | North American

Known from Boot Inl., Johansen B., the head of Minto Inl. and Oterkvik Pt., with these populations representing the species’ northern range limit. Additional information is provided in Gillespie et al. (2015), including photographs.

**NORTHWEST TERRITORIES. Boot Inl.**: *Gillespie et al. 9662* (CAN, O). **Minto Inl. (head)**: *Gillespie et al. 10243* (CAN). **NUNAVUT. Johansen B.**: *Gillespie et al. 8068* (ALA, CAN, MT, O, UBC), *8137* (ALTA, BABY, CAN). **Oterkvik Pt.**: *Gillespie et al. 7570* (ALA, CAN, O).

### Montiaceae [1/1]

***Montia* L. [1]**

***Montia fontana*** L., Fig. 61K–Water blinks | North American (NE)–amphi-Atlantic–European & amphi-Pacific/Beringian

Known from a single collection gathered in the Ulukhaktok area in 1949 (Porsild 1955, 1957, 1964, Porsild and Cody 1980, Aiken et al. 2007). Elsewhere in the Canadian Arctic recorded from southern Baffin and Coats islands, and scattered mainland sites (Porsild and Cody 1980, Aiken et al. 2007).

**NORTHWEST TERRITORIES. Ulukhaktok**: *Porsild 17286* (CAN).

### Asterids

**Ericales**

**Primulaceae [2/3]**

**Key to Primulaceae**

**Table:** 

1	Corolla campanulate, lavender; leaf blades glabrous	*** Primula ***
–	Corolla rotate, white; leaf blades pubescent	*** Androsace ***

### *Androsace* L. [2]

**Key to *Androsace* [adapted from Aiken et al. (2007) and Kelso (2009)]**

**Table:** 

1	Plants perennial, mat-forming, 2–15 cm high; leaves in multiple rosettes; blades obovate; flowers (1–)2–5 per inflorescence; petals 6–9 mm	**A. chamaejasme subsp. andersonii**
–	Plants annual or biennial, not mat-forming, (2–)5–30 cm high; leaves in single rosette; blades linear or lanceolate; flowers 3–16 per inflorescence; petals 4–4.5 mm	***A. septentrionalis***

**Figure 62.**
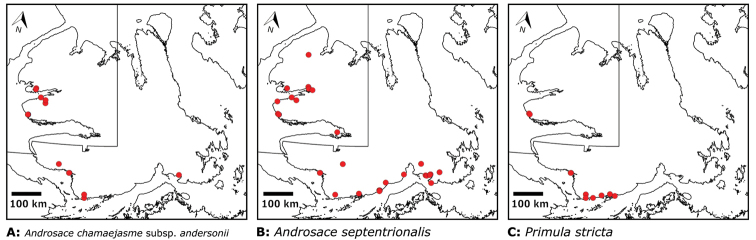
Species distribution maps. Primulaceae: **A**Androsace chamaejasme subsp. andersonii**B***Androsace septentrionalis***C***Primula stricta*.

**Figure 63.**
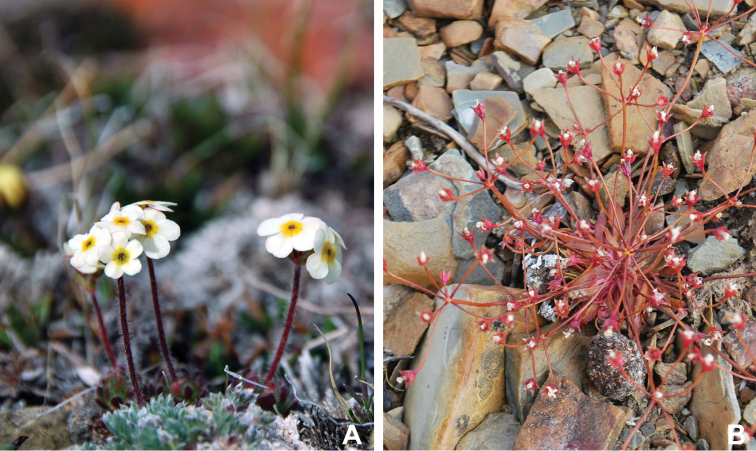
**A**Androsace chamaejasme subsp. andersonii habit, *Gillespie et al. 7495***B***Androsace septentrionalis* habit. Photo **A** by R.D. Bull and **B** by B.A. Bennett.

***Androsace chamaejasme*** subsp. ***andersonii*** (Hultén) Hultén, Figs 62A, 63A–Rock jasmine | Asian (N/C)–amphi-Beringian–North American (NW)

Previously recorded from Cambridge Bay (Porsild obs.), Cambridge Bay/Mt. Pelly, Kuujjua R., the head of Minto Inl. (Porsild obs.), Read I., Ulukhaktok and the “south coast” (*Rae*, K) (Simmons 1913, Porsild 1955, 1957, 1964, Porsild and Cody 1980, Aiken et al. 2007). Although there are at least three reports of this taxon from Cambridge Bay–an observation by Porsild, a Dutilly collection made in 1940 from “Cambridge Bay & Mt. Pelly” and a report by Thannheiser et al. (2001)–its area of occurrence in the area is uncertain, owing to imprecise locality information. This is the eastern most limit of the species in Canada, and vouchers are needed to more accurately document existence in the area. Newly recorded from Boot Inl., Falaise B. and Oterkvik Pt. Elsewhere in the Canadian Arctic recorded from the western mainland eastwards to the Coppermine R. (Porsild and Cody 1980, Aiken et al. 2007, Saarela et al. 2013, Saarela et al. 2017b).

**NORTHWEST TERRITORIES. Boot Inl.**: *Dutilly 18691* (QFA), *Gillespie et al. 9617* (CAN). **Kuujjua R.**: *Dutilly 18834*, *18835* (QFA), *Edlund 636* (CAN), *Gillespie et al. 9716* (ari, CAN, O). **Ulukhaktok**: *Dutilly 18265*, *18639* (QFA), *18625* (QFA, 2 sheets, QUE), *Edlund 288*, *709* (CAN), *Oldenburg 42-941* (CAN), *45-1652* (CAN, GH), *54-213* (GH), *Ross 6* (ALTA), *Saarela & Bull 1438* (CAN, O), *Svoboda 745021* (UBC). **NUNAVUT. Cambridge Bay & Ovayok TP**: *Dutilly 18168* (QFA). **Falaise B.**: *Levesque s.n.* (O). **Oterkvik Pt.**: *Gillespie et al. 7495* (CAN), *7622* (ALA, ALTA, BABY, CAN, MT, O, UBC, US). **Read I.**: *Oldenburg 43-1062*, *43-906*, *43-958*, *43-975* (CAN), *Porsild 17207* (CAN), *Ross 14A* (GH), *Wood s.n.* (CAN).

***Androsace septentrionalis*** L., Figs 62B, 63B–Northern fairy-candelabra | Circumboreal-polar

Previously recorded from Byron B., Cambridge Bay, C. Colborne, C. Wollaston, Ferguson L., the head of Minto Inl., Mt. Bumpus, the head of Prince Albert S., Richard Collinson Inl., Read I. and Ulukhaktok (Simmons 1913, Porsild 1955, 1957, 1964, Porsild and Cody 1980, Aiken et al. 2007). Thannheiser et al. (2001) additionally recorded it from Johansen B. (conf.) and Surrey L. Newly recorded from “30-Mile Cr.”, Boot Inl., Greiner L., Oterkvik Pt. and Sinclair Cr. Elsewhere in the Canadian Arctic recorded from Axel Heiberg, northern Baffin, Banks, Bylot, Ellesmere, King William, Melville and Southampton islands and scattered mainland sites (Porsild and Cody 1980, Korol 1992, Aiken et al. 2007, Saarela et al. 2013, Saarela et al. 2017b, Cuerrier 2018). There is a large distribution gap in the central Canadian Arctic Archipelago, bordered by the southern/western Victoria I., northern Baffin and northern Queen Elizabeth Islands populations.

**NORTHWEST TERRITORIES. Boot Inl.**: *Dutilly 18754* (QFA), *Gillespie et al. 9531* (CAN, O). **C. Wollaston**: *Edlund 46* (CAN). **Kuujjua R.**: *Dutilly 18853* (CAN), *18828*, *18865* (QFA), *Gillespie et al. 9986* (CAN, WIN). **Minto Inl. (head)**: *Edlund 51* (CAN), *Gillespie et al. 10022*, *10024*, *10119* (CAN), *Porsild 17414* (CAN). **Prince Albert S. (head)**: *Weerstra 14* (DAO). **Richard Collinson Inl.**: *Edlund 131* (CAN). **Ulukhaktok**: *Edlund 447* (CAN), *Oldenburg 42-33*, *45-1545* (CAN, GH), *Saarela & Bull 1473* (CAN). **NUNAVUT. “30-Mile Cr.**”: *Bennett et al. 14-0339* (UBC). **Byron B.**: *Dushenko 41* (UVIC), *Edlund & Argus 12848* (CAN). **Cambridge Bay**: *Bennett et al. 13-0206* (BABY, UBC, chars), *Dutilly & Duman 37123* (QFA), *Gillespie et al. 8460* (CAN, O), *Porsild 21641* (CAN), *Stephens 1029* (CAN, KANU), *1238* (CAN), *Washburn 20* (CAN, GH). **C. Colborne**: *Edlund & Argus 12731* (CAN). **Ferguson L.**: *Hainault 1962* (DAO). **Greiner L.**: *Ponomarenko VI-038A*, *VI-207* (CAN). **Johansen B.**: *Gillespie et al. 8066* (CAN). **Mt. Bumpus**: *Edlund 217* (CAN). **Oterkvik Pt.**: *Gillespie et al. 7524* (ALA, CAN, O). **Read I.**: *Oldenburg 43-1067 (CAN)*, *Porsild 17208* (CAN). **Sinclair Cr.**: *Gillespie et al. 8227* (CAN), *8280* (ALA, CAN, O, UBC), *8311* (ALA, CAN, MT, O).

### *Primula* L. [1]

***Primula stricta*** Hornem., Fig. 62C–Coastal primrose | North American (N)–amphi-Atlantic–European (N)

Previously recorded from Read I. and Ulukhaktok (Porsild 1955, 1957, 1964, Porsild and Cody 1980, Aiken et al. 2007). Thannheiser et al. (2001) additionally recorded it from Johansen B. (conf.). Newly recorded from Murray Pt. and Oterkvik Pt. The Murray Pt. population is the easternmost one known for the Canadian Arctic Archipelago. Elsewhere in the Canadian Arctic known from Banks I. and scattered mainland sites (Porsild and Cody 1980, Korol 1992, Aiken et al. 2007, Saarela et al. 2013, Saarela et al. 2017b, Cuerrier 2018).

**NORTHWEST TERRITORIES. Ulukhaktok**: *Edlund 519*, *827* (CAN). **NUNAVUT. Johansen B.**: *Gillespie et al. 7997* (CAN, O, UBC), *8125* (CAN, O). **Murray Pt.**: *Gillespie et al. 8187* (ALA, CAN, MT, O). **Oterkvik Pt.**: *Gillespie et al. 7597* (CAN), *7623* (ALA, CAN, O), *7709* (CAN). **Read I.**: *Oldenburg 43-962* (CAN, GH), *Porsild 17209* (CAN), *Ross 26A* (GH).

### Ericaceae [8/11]

**Key to Ericaceae [adapted from Murray et al. (2008), Fabijan (2009), Judd and Kron (2009), Tucker (2009a), Tucker (2009b) and Wallace (2009)]**

**Table:** 

1	Herbs; leaves basal (or appearing so); petals distinct	**2**
–	Shrubs or subshrubs; leaves cauline; petals connate (distinct in *Empetrum*)	**3**
2	Inflorescences symmetric racemes, usually erect; calyx lobes (2.2–)2.8–6 mm; corolla crateriform to broadly campanulate; petals obovate to round, 6–10(–11) mm, without basal tubercles	***Pyrola grandiflora***
–	Inflorescences secund racemes, often lax in bud or flower, becoming ± erect in fruit; calyx lobes 0.5–1.5 mm; corolla suburceolate; petals broadly ovate, 4.5–6 mm, with 2 inconspicuous basal tubercles	**Orthilia secunda subsp. obtusata**
3	Ovaries inferior; fruits baccate	*** Vaccinium ***
–	Ovaries superior; fruits drupaceous or capsules	**4**
4	Fruits drupaceous	**5**
–	Fruits capsular	**6**
5	Leaves whorled or spirally arranged, blades linear, oblong or elliptic; inflorescences solitary flowers; drupes black	*** Empetrum ***
–	Leaves alternate, blades ovate, obovate or oblanceolate; inflorescences racemes, 2–7-flowered; drupes black-purple, brick red or scarlet	*** Arctous ***
6	Corollas broadly funnelform or ± rotate; anthers without awns; fruit dehiscence septicidal	*** Rhododendron ***
–	Corollas cylindric or globose-urceolate; anthers with awns; fruit dehiscence loculicidal	**7**
7	Stems decumbent to erect, forming dense mats; leaves closely imbricate, blades narrowly triangular, 3–6 mm; inflorescences axillary, solitary flowers; corollas white to yellowish	***Cassiope tetragona***
–	Stems ascending or spreading; leaves not closely imbricate, blades linear to narrowly elliptic or oblong, (10–)20–50 mm; inflorescences terminal, umbelliform corymbs, 2–8-flowered, sometimes flowers solitary; corollas pink	***Andromeda polifolia***


### *Andromeda* L. [1]

**Figure 64.**
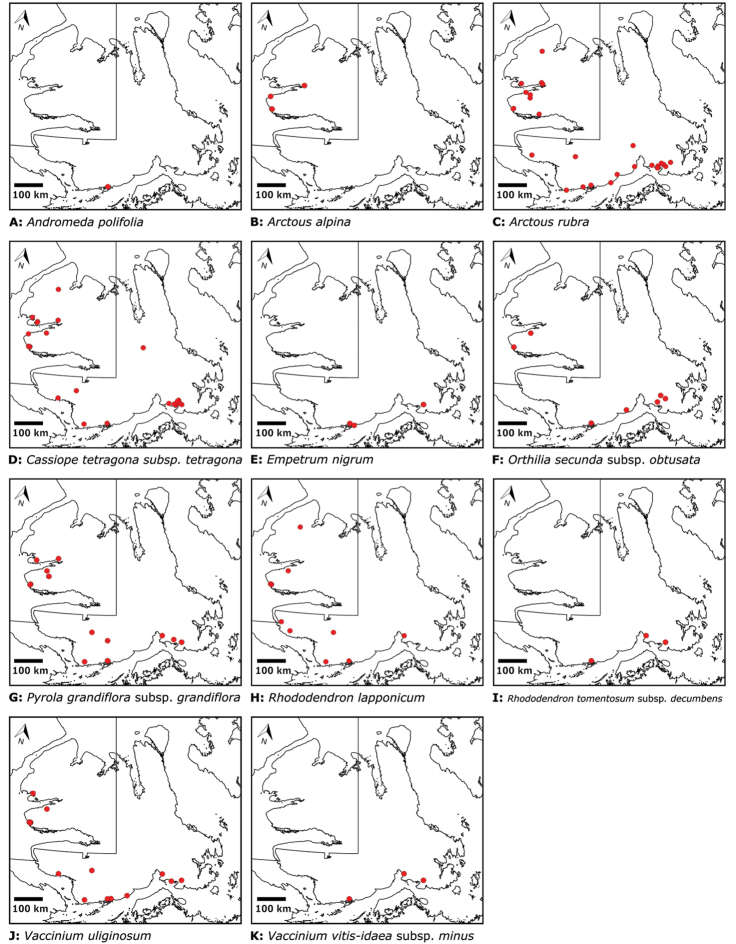
Species distribution maps. Ericaceae: **A***Andromeda polifolia***B***Arctous alpina***C***Arctous rubra***D**Cassiope tetragona subsp. tetragona**E***Empetrum nigrum***F**Orthilia secunda subsp. obtusata**G***Pyrola grandiflora***H***Rhododendron lapponicum***I**Rhododendron tomentosum subsp. decumbens**J***Vaccinium uliginosum***K**Vaccinium vitis-idaea subsp. minus.

***Andromeda polifolia*** L., Fig. 64A | Northern bog rosemary | Circumboreal-polar

Known from a single collection from Johansen B., marking the northern edge of the species’ range in the central Arctic; see additional details in Gillespie et al. (2015), including photographs.

**NUNAVUT. Johansen B.**: *Gillespie et al. 8002* (ALA, BABY, CAN, MT, O, UBC).

### *Arctous* (A.Gray) Nied. [2]

**Key to *Arctous* [adapted from Tucker (2009a)]**

**Table:** 

1	Leaf blades 4–15 mm, surfaces rugose, hairy toward base and on petiole (hairs 1–2 mm); twigs clothed with persistent old leaves or petioles; corolla lobes 0.5 mm; fruits black-purple; stones 2.7–4.6 × 2–3.6 mm	***A. alpina***
–	Leaf blades (10–)15–30(–60) mm, surfaces not or only slightly rugose, glabrous; twigs bare of old leaves; corolla lobes 1 mm; fruits brick red or scarlet; stones 2.5–3 × 1.6–2.2 mm	***A. rubra***

***Arctous alpina*** (L.) Nied. (*Arctostaphylos alpina* (L.) Spreng.), Fig. 64B–Alpine bearberry | Circumpolar-alpine

Previously recorded from the head of Minto Inl. and Ulukhaktok (Porsild obs., conf.) (Simmons 1913, Porsild 1955, 1957, 1964, Porsild and Cody 1980, Aiken et al. 2007). Four records mapped from “Long L.” as this species (Aiken et al. 2007), collected by Lambert in 1964, have been redetermined as *A. rubra*. Newly recorded from C. Wollaston; this collection was previously determined as *A. rubra*. The Northwest Territories Ecosystem Classification Group (2013) included a photo of this taxon reported as occurring within a stand of *Salix alaxensis* along Boot Inlet; we are not aware of a voucher specimen. Elsewhere in the Canadian Arctic recorded from southern Baffin, Banks and Southampton islands and numerous mainland sites (Porsild and Cody 1980, Cody et al. 1989, Korol 1992, Cody and Reading 2005, Aiken et al. 2007, Saarela et al. 2017b, Gauthier 2018). *Arctous alpina* grows in more acidic habitats than does *A. rubra*, which is more common on Victoria I.

**NORTHWEST TERRITORIES. C. Wollaston**: *Edlund 40* (CAN). **Minto Inl. (head)**: *Porsild 17403*, *17413* (CAN). **Ulukhaktok**: *Dutilly 18682* (DAO, QFA), *18686* (QFA), *Oldenburg 45-1543* (CAN).

**Figure 65.**
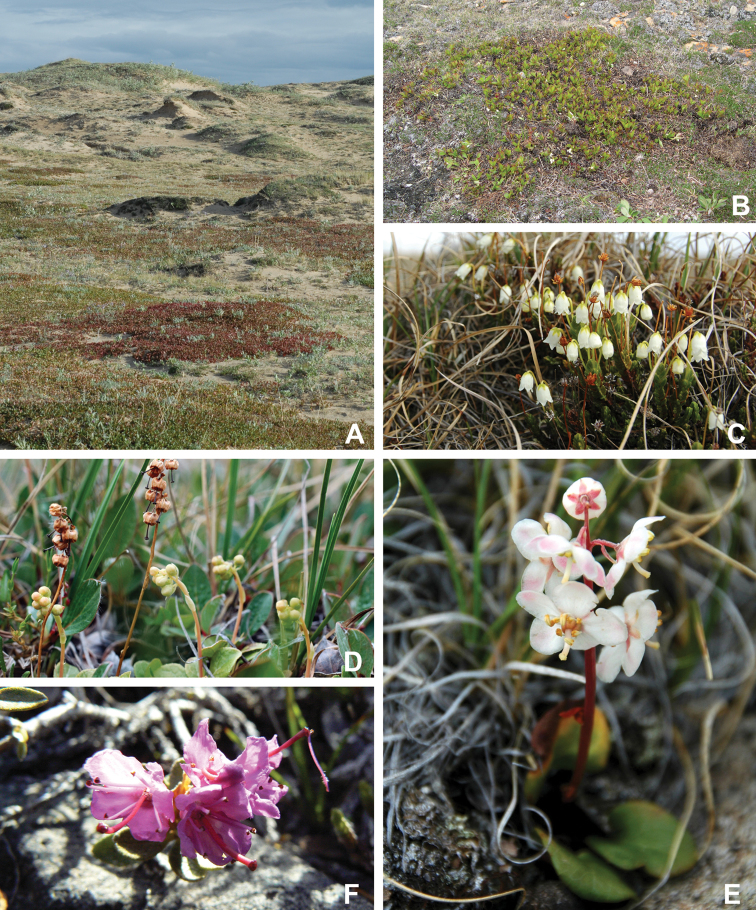
**A***Arctous rubra* habitat, *Gillespie et al. 9884***B***Arctous rubra* habit, *Gillespie et al. 7714***C**Cassiope tetragona subsp. tetragona habit, Oterkvik Point, NU, 3 July 2008 **D**Orthilia secunda subsp. obtusata habit, *Gillespie et al. 8712***E**Pyrola grandiflora subsp. grandiflora habit, *Gillespie et al. 7967***F***Rhododendron lapponicum* Kuujjua River, NT, 16 July 2010. Photos **A**, **B** by L.J. Gillespie **C**, **D**, **F** by R.D. Bull and **E** by P.C. Sokoloff.

***Arctous rubra*** (Rehder & E.H.Wilson) Nakai (*Arctostaphylos rubra* Rehder & E.H.Wilson), Figs 64C, 65A, B–Red bearberry | Asian (NE)–amphi-Beringian–North American (N)

Previously recorded from Kuujjua R., the head of Minto Inl., Mt. Bumpus, the north side of Prince Albert S., Richard Collinson Inl., Ulukhaktok and Wollaston P. (Macoun and Holm 1921, Porsild 1955, Porsild and Cody 1980, Aiken et al. 2007). A record previously mapped from C. Wollaston has been re-determined as *A. alpina*. Thannheiser et al. (2001) additionally recorded it from Johansen B. (conf.), Mt. Pelly (conf.), Richardson I. and Surrey L. Newly recorded from “30-Mile Cr.”, Byron B., Greiner L., Johansen B., Oterkvik Pt. and Sinclair Cr. Elsewhere in the Canadian Arctic recorded from Banks, Baffin (?) and Southampton islands and a few mainland sites (Porsild and Cody 1980, Aiken et al. 2007, Saarela et al. 2013, Saarela et al. 2017b, Gauthier 2018). This species is a calciphile (Porsild 1955), unlike its congener.

**NORTHWEST TERRITORIES. Boot Inl.**: *Edlund 584*, *585* (CAN), *Gillespie et al. 9587* (ALA, CAN, MT, O, UBC). **Kuujjua R.**: *Dutilly 18858* (DAO, QFA), *Edlund 637*, *638* (CAN), *Gillespie et al. 9794* (ALA, ari, CAN, O). **Minto Inl. (head)**: *Edlund 61*, *127* (CAN), *Gillespie et al. 10100* (ALA, CAN, MT, O). **Prince Albert S. (N)**: *Edlund 441* (CAN). **Richard Collinson Inl.**: *Edlund 129* (CAN). **Ulukhaktok**: *Bliss s.n.* (ALTA), *Edlund 460*, *750* (CAN), *Ross 12A* (GH), *Saarela & Bull 1482* (CAN, O), *Svoboda 745034* (UBC). **NUNAVUT. “30-Mile Cr.**”: *Bennett et al. 14-0352* (DAO). **Byron B.**: *Edlund & Argus 12851* (CAN). **Cambridge Bay**: *Bennett et al. 13-0269* (BABY, chars), *14-0323* (od), *Gillespie et al. 8479* (ALA, CAN, MT, O), *Gould s.n.* (ALA), *Stephens 831* (CAN, KSTC), *870* (KSTC). **Greiner L.**: *Ponomarenko VI-029*, *VI-181* (CAN). **Johansen B.**: *Gillespie et al. 7851* (ALA, ALTA, BABY, CAN, MT, O, UBC), *8104* (ALA, CAN, O). “**Long L.**”: *Lambert s.n.* (CAN, 4 sheets). **Mt. Bumpus**: *Edlund 211*, *285* (CAN). **Ovayok TP**: *Stephens 1176* (CAN), *988*, *1282* (CAN, KSTC). **Oterkvik Pt.**: *Gillespie et al. 7714* (ALA, BABY, CAN, MT, O, UBC). **Sinclair Cr.**: *Gillespie et al. 8293* (CAN). **Surrey L.**: *Edlund & Argus 12804* (CAN). **Wollaston P.**: *D. Jenness 576* (CAN).

### *Cassiope* D.Don [1]

***Cassiope tetragona*** (L.) D.Don subsp. ***tetragona***, Figs 64D, 65C–Arctic heather | Circumpolar–alpine

Previously recorded from Cambridge Bay, “Long L.”, the head of Minto Inl. (Porsild obs., conf.), Richard Collinson Inl., Ulukhaktok, Washburn L. (Porsild obs., conf.) and Wollaston P. (Macoun and Holm 1921, Porsild 1955, 1957, 1964, Porsild and Cody 1980, Aiken et al. 2007). Thannheiser et al. (2001) additionally recorded it from Johansen B. (conf.), Mt. Pelly (conf.), Richardson I. and Surrey L. Newly recorded from Boot Inl., C. Wollaston, Falaise B., Greiner L. and Oterkvik Pt. Widespread across the Canadian Arctic Archipelago and Arctic mainland (Porsild and Cody 1980, Aiken et al. 2007, Gauthier 2018), growing in areas where snow accumulates.

**NORTHWEST TERRITORIES. Boot Inl.**: *Dutilly 18742* (QFA), *Gillespie et al. 9604* (ALA, CAN, MT, O). **C. Wollaston**: *Edlund 43* (CAN). **Kuujjua R.**: *Gillespie et al. 9764* (ari, CAN, MT, O). **Minto Inl. (head)**: *Gillespie et al. 9497* (CAN, O). **Richard Collinson Inl.**: *Edlund 180* (CAN). **Ulukhaktok**: *Bliss s.n.* (ALTA, DAO), *Dutilly 18679* (QFA), *Edlund 461*, *751* (CAN), *Oldenburg 42-81*, *45-1547* (CAN), *Ross 3* (ALTA), *31A* (GH), *Saarela & Bull 1472* (CAN). **Walker B.**: *Oldenburg 45-1508* (CAN). **NUNAVUT. Cambridge Bay**: *Bennett et al. 13-0189* (chars, od, V), *14-0324* (CAN), *Calder et al. 24150* (DAO), *Edlund & Argus 12852* (ALA), *Gould s.n.* (ALA, CAN), *Porsild 21640* (CAN), *Stephens 973*, *1252* (CAN). **Falaise B.**: *Eriksen et al. 963* (ALA). **Greiner L.**: *Ponomarenko VI-028*, *VI-224* (CAN). **Johansen B.**: *Gillespie et al. 7831* (ALTA, BABY, CAN, MT, UBC, US). “**Long L.**”: *Lambert s.n.* (CAN). **Ovayok TP**: *Gillespie et al. 8426* (ALA, CAN, O). **Oterkvik Pt.**: *Gillespie et al. 7467* (CAN, MT, O, UBC), *7485* (ALTA, CAN, US). **Washburn L.**: *Oldenburg 46-2149* (CAN, GH). **Wollaston P.**: *D. Jenness 577* (CAN).

### *Empetrum* L. [1]

***Empetrum nigrum*** L., Fig. 64E–Crowberry | Circumboreal–polar

Previously known only from Ulukhaktok, based on an observation by Porsild (Porsild 1955), which was subsequently mapped (Porsild 1957, 1964, Porsild and Cody 1980, Aiken et al. 2007). Porsild (1955) noted the occurrence as “in one place only” in the vicinity of Old Holman, where the species should be looked for and a voucher obtained. Newly recorded from Johansen B., “Long L.” and Murray Pt. This species is restricted to acidic substrates, hence its sparseness on Victoria I. Elsewhere in the Canadian Arctic recorded from Axel Heiberg, Baffin, Somerset and Southampton islands and across the mainland (Porsild and Cody 1980, Cody et al. 1989, Korol 1992, Aiken et al. 2007, Saarela et al. 2013, Saarela et al. 2017b, Gauthier 2018).

**NUNAVUT. Johansen B.**: *Gillespie et al. 7968* (ALA, CAN, MT, O). “**Long L.**”: *Lambert s.n.* (CAN, 4 sheets). **Murray Pt.**: *Gillespie et al. 8184* (CAN, O).

### *Orthilia* Raf. [1]

***Orthilia secunda*** subsp. ***obtusata*** (Turcz.) Böcher (Pyrola secunda var. obtusata Turcz.), Figs 64F, 65D–One-sided wintergreen | Asian (N/C)–amphi-Beringian–North American

Previously recorded from C. Peel and Ulukhaktok (Aiken et al. 2007). Thannheiser et al. (2001) additionally recorded it from Cambridge Bay, Johansen B. (conf.), the head of Minto Inl. and Richardson I. At our Johansen B. site, we recorded it growing with Rhododendron tomentosum subsp. decumbens on dark grey igneous rock. Newly recorded from three sites in the vicinity of Greiner L. Elsewhere in the Canadian Arctic recorded from southern Baffin I. and across the mainland (Porsild and Cody 1980, Aiken et al. 2007, Saarela et al. 2013, Gillespie et al. 2015, Saarela et al. 2017b, Gauthier 2018).

**NORTHWEST TERRITORIES. Kuujjua R.**: *Gillespie et al. 9793* (CAN). **Ulukhaktok**: *Porsild 17323*, *17324*, *17325* (CAN). **NUNAVUT. C. Peel**: *Edlund 1* (CAN). **Greiner L.**: *Ponomarenko VI-102, VI-400*, *VI-451* (CAN). **Johansen B.**: *Gillespie et al. 8036* (ALA, CAN, O).

### *Pyrola* L. [1]

***Pyrola grandiflora*** Radius subsp. ***grandiflora***, Figs 64G, 65E–Large-flowered wintergreen | Circumpolar

Previously recorded from Boot Inl., Cambridge Bay, “Long L.”, Mt. Bumpus, the head of Prince Albert S. (Porsild obs.), south-central Victoria I., Washburn L. (Porsild obs.) and Ulukhaktok. Thannheiser et al. (2001) additionally recorded it from Cambridge Bay, Johansen B. (conf.), Richardson I. and Surrey L. Newly recorded from Kuujjua R., the head of Minto Inl., Greiner L. (possibly the area around Cambridge Bay where found by Thannheiser) and Oterkvik Pt. Elsewhere in the Canadian Arctic recorded from Baffin, Banks, Bylot, Coats, Devon, Ellesmere, Melville and Southampton islands and across the mainland (Porsild and Cody 1980, Cody et al. 1989, Korol 1992, Aiken et al. 2007, Saarela et al. 2013, Saarela et al. 2017b, Gauthier 2018).

**NORTHWEST TERRITORIES. Boot Inl.**: *Edlund 579* (CAN). **Kuujjua R.**: *Gillespie et al. 9808* (CAN, MT, O), *9953* (CAN), *9956* (ALA, ari, CAN, O, UBC, WIN). **Minto Inl. (head)**: *Gillespie et al. 10101* (ALA, ari, CAN, O). **Ulukhaktok**: *Edlund 835* (CAN), *Porsild 17322* (ALTA, CAN), *Ross 11* (ALTA). **NUNAVUT. Ferguson L. [Tahiryuaq**]: *Bennett et al. 14-0406* (UBC). **Greiner L.**: *Ponomarenko VI-118* (CAN). **Johansen B.**: *Gillespie et al. 7967* (CAN, O), *8050* (CAN). “**Long L.**”: *Lambert s.n.* (CAN, 3 sheets). **Mt. Bumpus**: *Edlund 222* (CAN). **Oterkvik Pt.**: *Gillespie et al. 7540* (CAN, O). **South-central Victoria I.**: *Edlund 541* (CAN).

### *Rhododendron* L. [2]

**Key to *Rhododendron* [adapted from Judd and Kron (2009)]**

**Table:** 

1	Inflorescences 3–6-flowered; corollas rose or purple (rarely white), broadly funnelform, (6.5–)7.5–14(–15) mm, petals connate 3/4+ their lengths; capsules basipetally dehiscent; leaf blades oblong-elliptic, elliptic, ovate or obovate, 2–7(–9) mm wide; flowers fragrant	***R. lapponicum***
–	Inflorescences 10–35-flowered; corollas white to cream, ± rotate, 2–8 mm, petals appearing distinct or slightly connate basally; capsules acropetally dehiscent; leaf blades linear, 1–2 mm wide; flowers not fragrant	**R. tomentosum subsp. decumbens**

***Rhododendron lapponicum*** (L.) Wahlenb. (R. lapponicum subsp. alpinum (Glehn.) A.P.Khokhr.), Figs 64H, 65F–Lapland rosebay | Asian (NE)–amphi-Beringian–North American (N)–amphi-Atlantic (W)

Previously recorded from Richard Collinson Inl., Ulukhaktok and Wollaston P. (Macoun and Holm 1921, Porsild 1955, 1957, 1964, Porsild and Cody 1980, Aiken et al. 2007). Thannheiser et al. (2001) additionally recorded it from Johansen B. (conf.) and Richardson I. Newly recorded from Kuujjua R., Ferguson L., Mt. Bumpus and Oterkvik Pt. Elsewhere in the Canadian Arctic recorded from the eastern archipelago (Baffin, Devon and Southampton islands), southern Banks I. and across the mainland (Porsild and Cody 1980, Cody et al. 1989, Korol 1992, Aiken et al. 2007, Saarela et al. 2013, Saarela et al. 2017b, Gauthier 2018). There is a conspicuous distribution gap in the central Canadian Arctic Archipelago (Aiken et al. 2007).

**NORTHWEST TERRITORIES. Kuujjua R.**: *Gillespie et al. 9803* (CAN, O). **Richard Collinson Inl.**: *Edlund 197* (CAN). **Ulukhaktok**: *Bliss s.n.* (ALTA, BABY, CAN, UBC), *Dutilly 18678* (QFA), *Edlund 454*, *741* (CAN), *Porsild 17326* (CAN), *Ross 25* (ALTA). **NUNAVUT. Ferguson L. [Tahiryuaq**]: *Bennett et al. 14-0421* (BABY). **Johansen B.**: *Gillespie et al. 7850* (ALA, CAN, MT, O, UBC). **Kugaluk R.**: *Edlund & Nixon 207* (CAN). **Mt. Bumpus**: *Edlund 209* (CAN). **Oterkvik Pt.**: *Gillespie et al. 7460* (ALTA, BABY, CAN, MT, UBC). **Wollaston P.**: *D. Jenness 579* (CAN).

**Figure 66.**
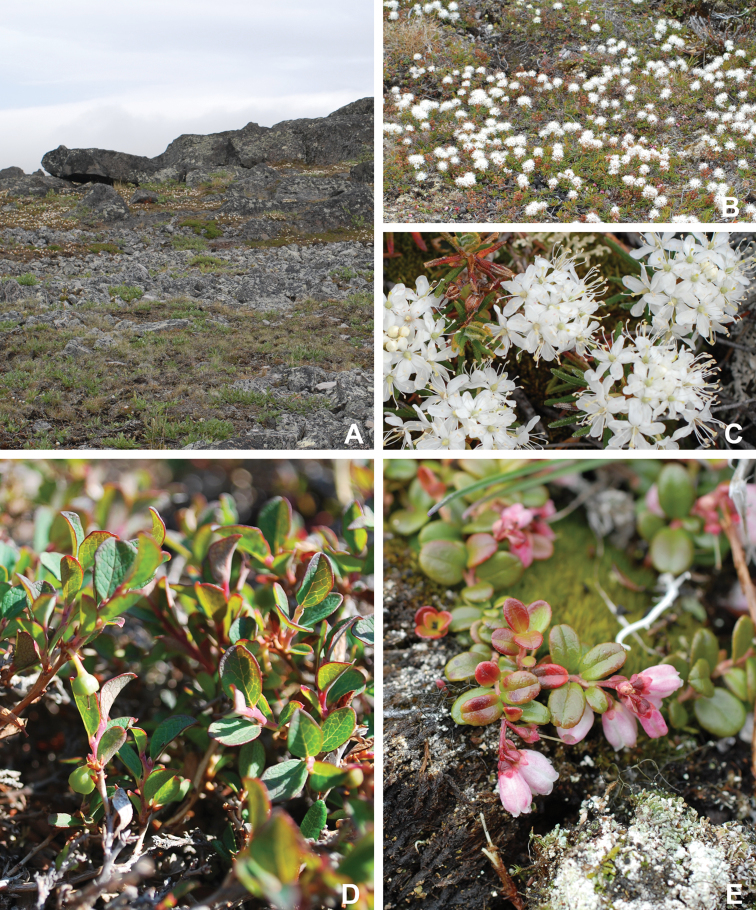
**A**Rhododendron tomentosum subsp. decumbens habitat, *Gillespie et al. 7849***B**Rhododendron tomentosum subsp. decumbens habit, *Gillespie et al. 7849***C**Rhododendron tomentosum subsp. decumbens inflorescence, *Gillespie et al. 7849***D***Vaccinium uliginosum* habit **E**Vaccinium vitis-idaea subsp. minus habit, *Gillespie et al. 8037.* Photos **A**, **B**, **C** by L.J. Gillespie **D** by B.A. Bennett and **E** by R.D. Bull.

***Rhododendron tomentosum*** subsp. ***decumbens*** (Aiton) Elven & D.F.Murray (*Ledum decumbens* (Aiton) Lodd. ex Steud., L. palustre var. decumbens Aiton, L. palustre subsp. decumbens (Aiton) Hultén, Figs 64I, 66A–C–Dwarf Labrador tea | Asian (N/C)–amphi-Beringian–North American (N)

Previously recorded from Ulukhaktok (Porsild obs.) (Porsild 1955), which was subsequently mapped, and “Long L.” (Porsild 1957, 1964, Porsild and Cody 1980, Aiken et al. 2007). At Ulukhaktok the species should be looked for in the vicinity of Old Holman, where it likely grows in the same area as the unvouchered *Empetrum nigrum*; a voucher should be obtained. Thannheiser et al. (2001) additionally recorded it from Johansen B. (conf.) and Richardson I. Newly recorded from Ferguson L. Elsewhere in the Canadian Arctic recorded from the eastern archipelago (Baffin and Southampton islands) and across the mainland (Porsild and Cody 1980, Cody et al. 1989, Korol 1992, Aiken et al. 2007, Saarela et al. 2013, Saarela et al. 2017b, Gauthier 2018). Like, *R. lapponicum*, there is a conspicuous gap in distribution in the central Canadian Arctic Archipelago (Aiken et al. 2007)

**NUNAVUT. Ferguson L. [Tahiryuaq**]: *Bennett et al. 14-0418* (BABY, chars). **Johansen B.**: *Gillespie et al. 7849* (ALA, ALTA, BABY, CAN, MT, O, UBC, US). “**Long L.**”: *Lambert s.n.* (CAN, 3 sheets).

### *Vaccinium* L. [2]

**Key to *Vaccinium* [adapted from Vander Kloet (2009)]**

**Table:** 

1	Leaves deciduous, blades usually glaucous abaxially, green to glaucous adaxially; inflorescences axillary; berries blue, 6–8 mm diam	***V. uliginosum***
–	Leaves persistent, blades pale and glandular abaxially, bright green adaxially; inflorescences terminal; berries red, 8–10 mm diam	**V. vitis-idaea subsp. minus**


***Vaccinium uliginosum*** L. (V. uliginosum subsp. microphyllum (Lange) Tolm.), Figs 64J, 66D–Bilberry | Circumboreal–polar

Previously recorded from “Long L.”, the head of Minto Inl. (Porsild obs.), Mt. Bumpus, Murray Pt. and Ulukhaktok (Porsild 1955, 1957, 1964, Porsild and Cody 1980, Aiken et al. 2007). Thannheiser et al. (2001) additionally recorded it from Johansen B. (conf.) and Richardson I. Newly recorded from Cambridge Bay, Falaise B., Ferguson L., Kuujjua R., Sinclair Cr. and Walker B. Observed in Ovayok TP by B. Bennett in 2013; a voucher is needed. Elsewhere in the Canadian Arctic recorded from Baffin, Banks, Coats, Cornwallis, Salisbury and Southampton islands and across the mainland (Porsild and Cody 1980, Cody et al. 1989, Korol 1992, Aiken et al. 2007, Saarela et al. 2013, Saarela et al. 2017b, Gauthier 2018).

**NORTHWEST TERRITORIES. Kuujjua R.**: *Gillespie et al. 9796* (ALA, CAN, MT, O). **Ulukhaktok**: *Dutilly 18677* (QFA), *Edlund 453*, *740*, *839* (CAN), *Oldenburg 42-11* (CAN), *Porsild 17327* (CAN), *Svoboda 745033* (UBC). **NUNAVUT. Cambridge Bay**: *Bennett et al. 13-0302* (BABY, CAN, chars). **Falaise B.**: *Eriksen et al. 964* (ALA). **Ferguson L. [Tahiryuaq**]: *Bennett et al. 14-0428* (CAN). **Johansen B.**: *Gillespie et al. 7848* (ALA, ALTA, BABY, CAN, MT, O, UBC, US). “**Long L.**”: *Lambert s.n.* (CAN). **Mt. Bumpus**: *Edlund 210* (CAN). **Murray Pt.**: *Edlund 530* (CAN). **Oterkvik Pt.**: *Gillespie et al. 7520* (ALA, ALTA, BABY, CAN, MT, O, UBC). **Sinclair Cr.**: *Gillespie et al. 8344* (CAN, O). **Walker B.**: *Oldenburg 45-1542* (CAN).

***Vaccinium vitis-idaea*** subsp. ***minus*** (Lodd., G.Lodd. & W.Lodd.) Hultén, Figs 64K, 66E–Mountain cranberry | Circumboreal–polar

Previously recorded from Walker B., based on an observation by H. Larsen reported in Porsild (1955), which was subsequently mapped (Porsild 1957, 1964, Porsild and Cody 1980). Aiken et al. (2007) mapped occurrences from the Johansen B. area, “Long L.”, the head of Minto Inl., Mt. Bumpus and Ulukhaktok. Of these records we are only aware of vouchers from “Long L.”, and occurrence in the Johansen B. area is confirmed by our collection made there in 2008. Thannheiser et al. (2001) additionally recorded it from Richardson I. Newly recorded from Ferguson L. Elsewhere in the Canadian Arctic recorded from Baffin, Banks, Coats, Salisbury and Southampton islands and across the mainland (Porsild and Cody 1980, Korol 1992, Cody and Reading 2005, Aiken et al. 2007, Saarela et al. 2013, Saarela et al. 2017b, Gauthier 2018).

**NUNAVUT. Ferguson L. [Tahiryuaq**]: *Bennett et al. 14-0420* (CAN). **Johansen B.**: *Gillespie et al. 7830* (CAN), *8037* (ALA, ALTA, BABY, CAN, MT, O, UBC). “**Long L.**”: *Lambert s.n.* (CAN, 2 sheets).

### Gentianales

**Rubiaceae [1/1]**

***Galium* L. [1]**

**Figure 67.**
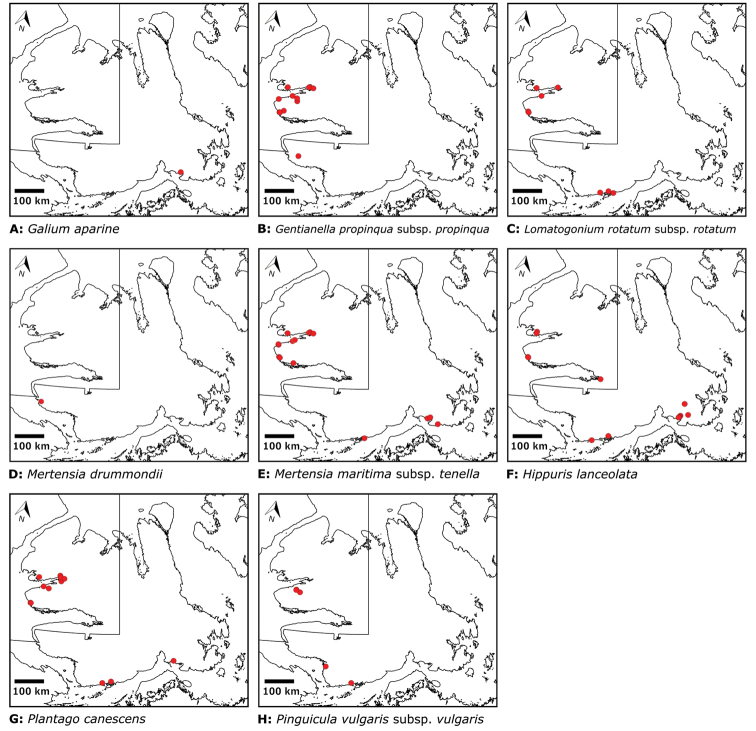
Species distribution maps. Rubiaceae: **A***Galium aparine*. Gentianaceae: **B**Gentianella propinqua subsp. propinqua**C**Lomatogonium rotatum subsp. rotatum. Boraginaceae: **D***Mertensia drummondii***E**Mertensia maritima subsp. tenella. Plantaginaceae: **F***Hippuris lanceolata***G***Plantago canescens*. Lentibulariaceae: **H**Pinguicula vulgaris subsp. vulgaris.

**Figure 68.**
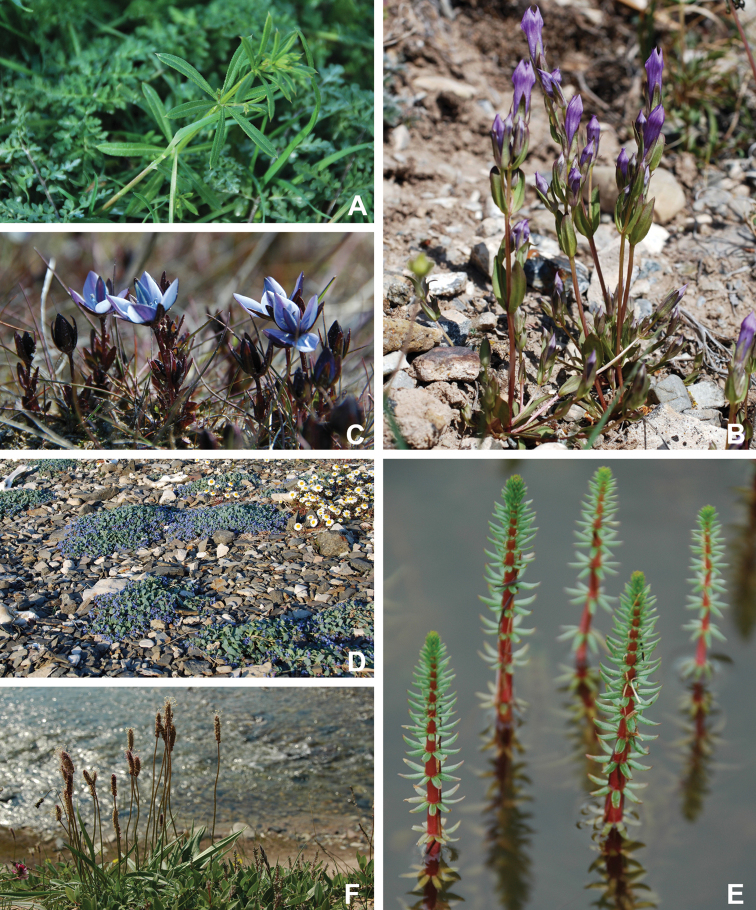
**A***Galium aparine* habit, *Saarela and Teeter 5295***B**Gentianella propinqua subsp. propinqua habit, *Gillespie et al. 9619***C**Lomatogonium rotatum subsp. rotatum habit, *Gillespie et al. 8109***D**Mertensia maritima subsp. tenella habitat, *Saarela & Bull 1469***E***Hippuris lanceolata* habit, Johansen Bay, NU, 18 July 2008 **F***Plantago canescens* habit, *Gillespie et al. 8103.* Photos **A**, **B**, **D** by J.M. Saarela and **C**, **E**, **F** by R.D. Bull.

***Galium aparine*** L., Figs 67A, 68A–Common bedstraw

A single small vegetative plant was found growing in Cambridge Bay, in 2017, in heavily disturbed ground with several introduced grasses (*Festuca rubra*, Poa pratensis subsp. pratensis and *Lolium perenne*) and the native *Descurainia sophioides*. We suspect the bedstraw was a contaminant of the mixed seed from which we assume the grasses originated, as there are no other records of this non-native, annual taxon for the community. Whatever its origin, this is the first record for Nunavut. It is unknown, however, if the species persists; our collection may have extirpated it from the territory.

**NUNAVUT. Cambridge Bay**: *Saarela & Teeter* 5295 (CAN).

### Gentianaceae [2/2]

**Key to Gentianaceae [adapted from Porsild and Cody (1980) and Aiken et al. (2007)]**

**Table:** 

1	Corollas tubular, petals connate 2/3+ their length; plants of dry sunny slopes and herbmats	**Gentianella propinqua subsp. propinqua**
–	Corollas rotate, petals connate only near base; plants of imperfectly drained moist areas, lake shores, and saline areas	**Lomatogonium rotatum subsp. rotatum**

### *Gentianella* Moench [1]

***Gentianella propinqua*** (Richardson) J.M.Gillett subsp. ***propinqua*** (*Gentiana arctophila* Griseb.), Figs 67B, 68B–Four-parted gentian | Amphi-Beringian (E)–North American (N)

Previously recorded from Boot Inl., C. Wollaston, Kuujjua R., the head of Minto Inl. and Ulukhaktok (Porsild 1955, 1957, 1964, Porsild and Cody 1980, Aiken et al. 2007). Thannheiser et al. (2001) additionally recorded it from Johansen B., which, if confirmed, would be the easternmost record on Victoria I. Newly recorded from Wollaston P., where collected in the Hanerak area of SW Wollaston P. (see map in Jenness (1922)) in 1955. At one Boot Inl. site plants grew on a dry S-facing slope with Oxytropis arctica var. arctica and *Dryas integrifolia*. Elsewhere in the Canadian Arctic recorded from Banks I. and scattered mainland sites (Porsild and Cody 1980, Cody et al. 2003, Aiken et al. 2007, Saarela et al. 2013, Saarela et al. 2017b, Dignard 2018b).

**NORTHWEST TERRITORIES. Boot Inl.**: *Gillespie et al. 9605* (CAN), *9619* (CAN). **C. Wollaston**: *Edlund* 10 (CAN). **Kuujjua R.**: *Edlund 629* (CAN), *Gillespie et al. 9907* (CAN, O), *9921* (CAN, O). **Minto Inl. (head)**: *Edlund 159* (CAN), *Gillespie et al. 10168* (ALA, ari, CAN), *10191* (CAN), *Porsild 17415* (CAN). **Ulukhaktok**: *Bliss s.n.* (ALTA), *Edlund 282*, *431*, *776* (CAN), *Porsild 17328* (CAN), *Ross 35* (ALTA), *35A* (GH), *Svoboda 745011* (UBC). **NUNAVUT. Wollaston P.**: *Miller 201* (CAN).

### *Lomatogonium* A.Braun [1]

***Lomatogonium rotatum*** (L.) Fr. subsp. ***rotatum***, Figs 67C, 68C–Marsh felwort | European (NE) & Asian (C-NE) & North American–amphi-Atlantic (W)

Previously recorded from Ulukhaktok (Porsild 1955, 1957, 1964, Porsild and Cody 1980, Aiken et al. 2007). Thannheiser et al. (2001) additionally recorded it from Johansen B. (conf.). Newly recorded from Boot Inl., Kuujjua R., the head of Minto Inl. and Murray Pt. Elsewhere in the Canadian Arctic recorded from scattered sites across the mainland (Porsild and Cody 1980, Cody et al. 2003, Aiken et al. 2007, Saarela et al. 2013, Saarela et al. 2017b, Gauthier 2018).

**NORTHWEST TERRITORIES. Boot Inl.**: *Gillespie et al. 9629* (CAN, O). **Kuujjua R.**: *Gillespie et al. 9930* (CAN, O). **Minto Inl. (head)**: *Gillespie et al. 10195* (ALA, CAN, O). **Ulukhaktok**: *Edlund 516* (CAN), **Ulukhaktok**: *Porsild* 17329 (ALA, ALTA, CAN), *Ross 40* (ALTA). **Johansen B.**: *Gillespie et al. 8000* (ALA, CAN, MT, O), *Gillespie et al. 8109* (ALTA, BABY, CAN, UBC). **Murray Pt.**: *Gillespie et al. 8218* (CAN).

### Boraginales

**Boraginaceae [1/2]**

***Mertensia* Roth [2]**

**Key to *Mertensia* [adapted from Porsild and Cody (1980) and Aiken et al. (2007)]**

**Table:** 

1	Petals purple and blue, 6–7.5 mm; sepals 1–2.5 mm; stems decumbent or spreading; leaf blades ± fleshy, glaucous, glabrous, apices obtuse or rounded; a seashore species	**M. maritima subsp. tenella**
–	Petals blue, 10–15 mm; sepals 0.9–1.1 mm; stems erect or ascending; leaf blades not fleshy, not glaucous, with short, stiff hairs on the margins and adaxially, apices acute; not a seashore species	***M. drummondii***

***Mertensia drummondii*** (Lehmann) G.Don., Fig. 67D–Drummond bluebell | American Beringian

Previously recorded from Wollaston P., where gathered in 1915 (Macoun and Holm 1921, Porsild 1955, Aiken et al. 2007) and not seen on the island since. This is one of few collections made by Diamond Jenness on Wollaston P. in 1915 for which a precise date (11 August 1915) of collection is recorded on the specimen label; for most collections made during this expedition, only the year and month are recorded. A hand drawn map in Jenness (1922) shows the general locations, by date, of his travels on the island. Between 10 and 12 August 1915, Jenness was inland of “Pt. William’s” (Williams Point) and Cape Hamilton, travelling in a northwesterly direction. Based on this information, we have georeferenced the 11 August *Mertensia* collecting site at 69°31'23"N, 116°23'38"W ± 10 km, the approximate midpoint between his locations on 10 and 12 August. This general location is about 100 km northwest of the occurrence as mapped in earlier treatments. Porsild mapped the site further east, at the base of Wollaston P. in the vicinity of Read Island (Porsild 1957, Porsild and Cody 1980), and Aiken et al. (2007) mapped the site in the same area, but slightly further inland, presumably basing the record on the map in Porsild and Cody (1980).

Elsewhere in the Canadian Arctic this globally rare species is known from only the Croker R. delta and Clifton Point, Nunavut and C. Young, Northwest Territories (Williams 1940, Cody et al. 1992, Saarela et al. 2013). The general status rank of this species in Northwest Territories is May Be At Risk (Working Group on General Status of NWT Species 2016). Known from fewer than 20 sites on the North Slope of Alaska, where the habitat is recorded as sparsely vegetated, active sand dunes and blowouts near rivers (Cortés-Burns et al. 2009).

**NUNAVUT**. **Wollaston P.**: *D. Jenness 410* (CAN).

***Mertensia maritima*** subsp. ***tenella*** (Th.Fr.) Elven & Skarpaas, Figs 67E, 68D–Seaside bluebells | Amphi-Beringian–North American (N)–amphi-Atlantic (W)

Previously recorded from Anderson B., C. Wollaston, the head of Minto Inl., the north side of Prince Albert S. and Ulukhaktok (Porsild 1955, 1957, 1964, Porsild and Cody 1980, Aiken et al. 2007). An inland site on Wollaston P., mapped by Aiken et al. (2007), is likely an error as this is a sea shore species. Newly recorded from Boot Inl., Cambridge Bay, Kuujjua R. and Murray Pt. Elsewhere in the Canadian Arctic recorded from Baffin, Banks, Coats, Devon and Southampton islands and several mainland sites (Porsild and Cody 1980, Cody et al. 1989, Korol 1992, Aiken et al. 2007, Saarela et al. 2013, Saarela et al. 2017b, Labrecque 2018).

**NORTHWEST TERRITORIES. Boot Inl.**: *Gillespie et al. 9627* (ALA, ari, CAN, MT, O, UBC). **C. Wollaston**: *Edlund 24* (CAN), *62* (CAN, GH, ID). **Kuujjua R.**: *Gillespie et al. 9917* (ALA, ari, CAN, MT, O), *Oldenburg 54-220* (GH). **Minto Inl. (head)**: *Edlund 170* (CAN), *Gillespie et al. 10193* (ALA, CAN, O, UBC, US, WIN), *Porsild 17416* (CAN). **Prince Albert S. (N)**: *Edlund 438* (CAN). **Ulukhaktok**: *Edlund 497*, *851* (CAN), *Ross 42* (ALTA), *42A* (GH), *Saarela & Bull 1469* (ALA, ari, CAN, MT, O). **NUNAVUT. Anderson B.**: *Edlund & Argus 12704* (CAN). **Cambridge Bay**: *Bennett et al. 13-0313* (BABY, chars), *Gillespie et al. 8474* (ALA, CAN, O), *Polunin s.n.* (CAN). **Murray Pt.**: *Gillespie et al. 8214* (CAN).

### Lamiales

**Plantaginaceae [2/2]**

**Key to Plantaginaceae [adapted from Aiken et al. (2007)]:**

**Table:** 

1	Plants aquatic; leaves distributed along the stems, whorled; flowers solitary, axillary; petals absent; stamens 1; fruit an achene	***Hippuris lanceolata***
–	Plants terrestrial; leaves basal; inflorescences oblong-ovate spikes on leaf-less scapes petals present; stamens 4; fruit a capsule	***Plantago canescens***

### *Hippuris* L. [1]

***Hippuris lanceolata*** Retz., Figs 67F, 68E–Lance-leaved mare’s-tail | Circumpolar

Previously recorded from Cambridge Bay, the head of Prince Albert S. and Ulukhaktok (Porsild observation, as *H. vulgaris* L.; conf.) (Porsild 1955, 1957, 1964, Porsild and Cody 1980, Aiken et al. 2007). Thannheiser et al. (2001) additionally recorded it (as *H. vulgaris*) from Johansen B. (conf.), Mt. Pelly, Richardson I., Surrey L. and Wellington L. Newly recorded from Boot Inl., Ferguson L., Greiner L. and Oterkvik Pt. Taxonomy follows Elven et al. (2011) and Elven et al. (2012). Records of *H. vulgaris* mapped for Victoria I. in Aiken et al. (2007), from Cambridge Bay, Ferguson L. and Ulukhaktok, have been redetermined as this species. The Boot Inl. records represent a range extension on the island with respect to the previously-known Ulukhaktok occurrence. Elsewhere in the Canadian Arctic recorded from Coats, Baffin, Banks, Ellesmere and Southampton islands as well as mainland sites (Aiken et al. 2007, Saarela et al. 2013, Saarela et al. 2017b, Labrecque 2018).

**NORTHWEST TERRITORIES. Boot Inl.**: *Dutilly 18720* (QFA), *Gillespie et al. 9663* (CAN, O). **Prince Albert S. (head)**: *Porsild 17446* (CAN). **Ulukhaktok**: *Edlund 480*, *761* (cf.) (CAN). **NUNAVUT. Cambridge Bay**: *Bennett 13-0221* (ALA, BABY, chars, UBC), *13-0193* (od), *Gillespie et al. 8378* (ALA, BABY, CAN, MT, O, UBC), *8420* (ALA, CAN, MT, O), *Oldenburg 44*-897 (CAN), *Polunin s.n.* (CAN), *Porsild 17471* (CAN), *Stephens 1130* (CAN). **Ferguson L. [Tahiryuaq**]: *Edlund & Argus 12773*, *12774* (cf.) (CAN). **Greiner L.**: *Ponomarenko VI-202* (CAN). **Johansen B.**: *Gillespie et al. 7907* (ALA, ALTA, BABY, CAN, MT, O, UBC, US). **Oterkvik Pt.**: *Gillespie et al. 7677* (CAN, O).

### *Plantago* L. [1]

***Plantago canescens*** Adams, Figs 67G, 68F–Hairy plantain | American Beringian

Previously recorded from Cambridge Bay (Augustus Hills area), the head of Minto Inl. and Ulukhaktok (Porsild 1955, 1957, 1964, Porsild and Cody 1980, Aiken et al. 2007). Thannheiser et al. (2001) additionally recorded it from Richardson I. Newly recorded from Boot Inl., Kuujjua R. and Johansen B. Observed and photographed by J. Wagner in 2015 growing close to the seashore in the Augustus Hills area (69.16628, -105.62496), the same general area where the taxon was collected by Edlund & Argus in 1987. Elsewhere in the Canadian Arctic recorded from Banks I., mainland Northwest Territories and a few sites on mainland Nunavut as far east as Bathurst Inl. (Porsild and Cody 1980, Aiken et al. 2007, Saarela et al. 2013). The Victoria I. populations mark the northeastern limit of the species’ range.

**NORTHWEST TERRITORIES. Boot Inl.**: *Gillespie et al. 9534* (CAN, O). **Kuujjua R.**: *Gillespie et al. 9829*, *9927* (CAN, O). **Minto Inl. (head)**: *Edlund 146* (CAN), *Gillespie et al. 10028* (ari, CAN, O), *10120*, *10134* (CAN), *10264* (ALA, CAN, O), *Porsild 17418* (CAN). **Ulukhaktok**: *Edlund 286*, *781* (CAN). **NUNAVUT. Cambridge Bay**: *Edlund & Argus 12853* (CAN). **Johansen B.**: *Gillespie et al. 7991* (CAN, O), *8103* (ALA, ALTA, BABY, CAN, MT, O, UBC).

### Lentibulariaceae [1/1]

***Pinguicula* L. [1]**

***Pinguicula vulgaris*** L. subsp. ***vulgaris***, Fig. 67H–Common butterwort | Amphi-Pacific–North American–amphi-Atlantic–European

Known from multiple collections gathered in 2008 and 2010 at Clouston B., Kuujjua R. and Johansen B., and an unvouchered report; see Gillespie et al. (2015) for details. Elsewhere in the Canadian Arctic recorded from southern Baffin Island and across the mainland (Porsild and Cody 1980, Aiken et al. 2007, Saarela et al. 2013, Saarela et al. 2017b, Garneau 2018a).

**NORTHWEST TERRITORIES. Kuujjua R.**: *Gillespie et al. 9878*, *9880* (CAN), *9967* (CAN, O). **NUNAVUT. Clouston B.**: *Gillespie et al. 7718* (ALA, CAN, MT, O, UBC). **Johansen B.**: *Gillespie et al. 8132* (CAN).

### Orobanchaceae [2/8]

**Key to Orobanchaceae [adapted from Porsild and Cody (1980) and Egger et al. (2019)]**

**Table:** 

1	Leaves pinnately lobed; bracts of inflorescence greenish	*** Pedicularis ***
–	Leaves entire or with up to 5 linear to lanceolate lobes; bracts of inflorescence pink, red, purple, yellow, yellow-green or pale-whitish	*** Castilleja ***

### *Castilleja* Mutis ex L.f. [2]

**Key to *Castilleja* [adapted from Egger et al. (2019)]**

**Table:** 

1	Inflorescences mostly pink or purple, bracts sometimes whitish distally, rarely mostly white	***C. elegans***
–	Inflorescences mostly to entirely yellow, yellow-green or cream, sometimes with a dull purplish wash on some bracts and/or on lower lip of corollas	***C. caudata***

**Figure 69.**
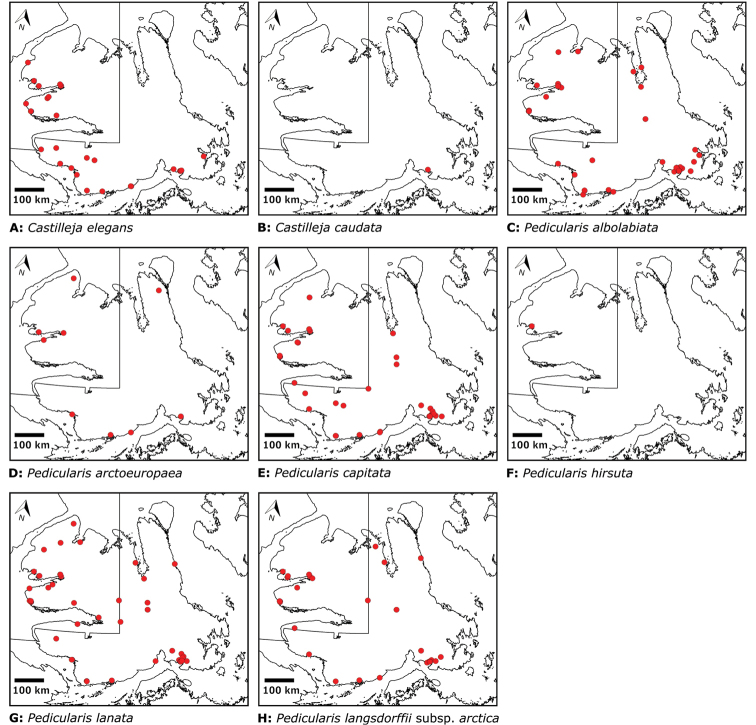
Species distribution maps. Orobanchaceae: **A***Castilleja elegans***B***Castilleja caudata***C***Pedicularis albolabiata***D***Pedicularis arctoeuropaea***E***Pedicularis capitata***F***Pedicularis hirsuta***G***Pedicularis lanata***H**Pedicularis langsdorffii subsp. arctica.

**Figure 70.**
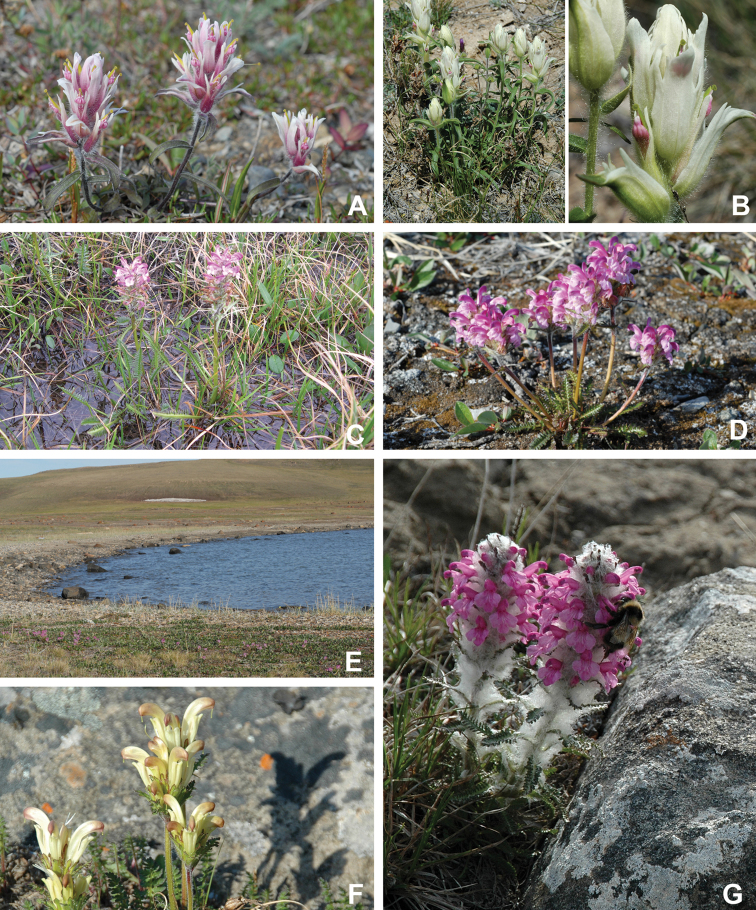
**A***Castilleja elegans* habit, *Gillespie et al. 9822***B***Castilleja elegans* habit (left) and inflorescence (right), *Gillespie et al. 9606***C***Pedicularis albolabiata* habit, *Gillespie et al. 7792***D***Pedicularis arctoeuropaea* habit, *Gillespie et al. 9628***E***Pedicularis arctoeuropaea* habitat, *Gillespie et al. 9628***F***Pedicularis capitata* habit, *Gillespie et al. 9992***G***Pedicularis lanata* habit, Kuujjua River, NT, 15 July 2010. Photos **A**, **C–E** by L.J. Gillespie and **B**, **F**, **G** by R.D. Bull.

***Castilleja elegans*** Malte (C. pallida subsp. elegans (Malte) Pennell, Figs 69A, 70A, B–Elegant paintbrush | Amphi-Beringian–North American (NW)

Previously recorded from Albert Edward B., Byron B., Cambridge Bay, the west end of Diamond Jenness P., the head of Minto Inl., Mt. Bumpus, Ulukhaktok and Wollaston P. (Macoun and Holm 1921, Porsild 1955, 1957, 1964, Porsild and Cody 1980, Aiken et al. 2007). Thannheiser et al. (2001) additionally recorded it from Johansen B. (conf.). Newly recorded from Clouston B., Colville Mts., Falaise B., Gordon Pt., Kuujjua R., Oterkvik Pt., the north side of Prince Albert S., Read I., Sinclair Cr. and Walker B. Elsewhere in the Canadian Arctic recorded from Banks I. and across the mainland to Hudson Bay (Porsild and Cody 1980, Aiken et al. 2007, Saarela et al. 2017b).

**NORTHWEST TERRITORIES. Boot Inl.**: *Gillespie et al. 9606* (CAN). **Byron B.**: *Dushenko s.n.* (UVIC). **Diamond Jenness P. (W end)**: *Stretton 83* (DAO). **Gordon Pt.**: *Stretton 197* (DAO). **Kuujjua R.**: *Gillespie et al. 9822* (ari, CAN, O), *9884* (CAN, O). **Minto Inl. (head)**: *Edlund 113*, *595*, *69* (CAN). **Minto Inl. (head)**: *Gillespie et al. 10086* (ALA, CAN, MT, O, UBC), *10130* (ALA, CAN, O). **Prince Albert S. (N)**: *Edlund 439* (CAN). **Ulukhaktok**: *Edlund 309*, *654* (CAN), *Oldenburg 45-1635* (CAN), *Ross 27A* (GH), *Saarela & Bull 1422* (ALA, CAN, O), *Svoboda* 745036 (UBC). **Walker B.**: *Oldenburg 45-1523A* (CAN, GH), *45-1525A* (CAN), *45-1524A* (CAN, GH). **Albert Edward B.**: *Edlund & Argus 12787* (ALA, CAN). **Cambridge Bay**: *Bennett et al. 13-0234* (chars, UBC, WTU), *13-0324* (BABY, WTU), *Edlund & Argus 12660*, *12858* (ALA, CAN), *Gillespie et al. 8388* (ALA, CAN, MT, O), *Smith & Sweatman 44* (DAO), *Stephens 1102* (CAN). **Clouston B.**: *Gillespie et al. 7727* (CAN, O). **Colville Mts.**: *Gillespie et al. 7769* (CAN). **Falaise B.**: *Eriksen et al. 930* (ALA), *Parker 9106* (ALA). **Johansen B.**: *Gillespie et al. 8085* (ALA, BABY, CAN, MT, O, UBC). **Kugaluk R.**: *Edlund & Nixon 208* (CAN). **Mt. Bumpus**: *Edlund 254* (CAN). **Oterkvik Pt.**: *Gillespie et al. 7543* (CAN). **Read I.**: *Ross 31A* (GH). **Sinclair Cr.**: *Gillespie et al. 8352* (CAN). **Wollaston P.**: *D. Jenness 324b* (CAN).

***Castilleja caudata*** (Pennell) Rebrist. (C. pallida var. caudata (Pennell) B.Boivin), Fig. 69B–Pale paintbrush | Asian (NE)–amphi-Beringian

Not recorded for Victoria I. by Porsild and Cody (1980) or Aiken et al. (2007), whereas Thannheiser et al. (2001) recorded it from Cambridge Bay, Johansen B., Minto Inl. and Richardson I. The only confirmed collection from Victoria I. is a recent one from Cambridge Bay, where the taxon grew along the roadside N of the DEW line site, on limestone bedrock and associated with *Equisetum arvense*, *Puccinellia nuttalliana*, *Poa glauca*, *Festuca brachyphylla*, *Salix arctica* and *Draba glabella*. This is the only confirmed record for the Canadian Arctic Archipelago. Elsewhere in the Canadian Arctic recorded from western mainland sites as far east as the Bathurst Inl. area (Porsild and Cody 1980, Gould and Walker 1997, Saarela et al. 2013, Saarela et al. 2017b).

**NUNAVUT. Cambridge Bay**: *Bennett et al. 14-0382* (WTU, det. M. Egger, 2015).

### *Pedicularis* L. [6]

**Key to *Pedicularis* [adapted from Porsild and Cody (1980), Molau and Murray (1996) and Sokoloff et al. (2016)]**

**Table:** 

1	Corollas yellow, 3–4 cm; inflorescences of 2–4 flowers; stems emerging from a thin, spindly rhizome	***P. capitata***
–	Corollas pink to purple, 1–3 cm; inflorescences of 10–30 flowers; stems emerging from a stout rootstock or a taproot	**2**
2	Stems scapose, from a branching rootstock	**3**
–	Stems leafy, from a central taproot	**4**
3	Inflorescences glabrate to white lanate; corolla lips white to pale pink; galeas dark purple recurved, and appressed to lip; petioles of basal leaves long (approx. 2–3× length of blade)	***P. albolabiata***
–	Inflorescences usually yellowish-white lanate; corolla lips pink; galeas only slightly darker pink, not recurved or appressed to lip; petioles of basal leaves shorter (approx. 0.5–1.2× length of blade)	***P. arctoeuropaea***
4	Taproot deep lemon-yellow; inflorescences densely white-woolly, hairs often obscuring the calyces; flowers deep-pink to purple	***P. lanata***
–	Taproot pale yellow; inflorescences glabrous to densely hairy but calyces still visible; flowers pale pink to purple	**5**
5	Style hidden inside galea; anthers 0.8–2.0 mm; galea teeth absent or short (0–0.5 mm); corolla pale pink; inflorescence moderately to densely hairy	***P. hirsuta***
–	Style protruding 0.5–3.0 mm from tip of galea; anthers 2.0–3.2 mm; galea teeth longer (0.2–0.7 mm); corolla deep pink to purple; inflorescence glabrate to moderately hairy (rarely densely hairy)	**P. langsdorffii subsp. arctica**


***Pedicularis albolabiata*** (Hultén) Kozhevn (P. sudetica subsp. albolabiata Hultén), Figs 69C, 70C–White-lipped lousewort | Asian (N)–amphi-Beringian–North American (N)

Previously recorded from Cambridge Bay, Hadley B., the head of Minto Inl., Mt. Bumpus, Namaycush L. and Richard Collinson Inl. (Aiken et al. 2007). Newly recorded from Albert Edward B., Boot Inl., Clouston B., Falaise B., Ferguson L., Greiner L., Kuujjua R., Johansen B., Murray Pt. and Oterkvik Pt. Earlier authors (Porsild 1955, 1957, 1964, Porsild and Cody 1980), Thannheiser et al. (2001)included this species and the next one in *Pedicularis sudetica* Willd. and did not recognize infraspecific taxa. Elsewhere in the Canadian Arctic recorded from Axel Heiberg, Baffin, Banks, Coats, Devon, Eglinton, Ellesmere, King William, Melville, Prince Patrick and Southampton islands and across the mainland (Aiken et al. 2007, Saarela et al. 2013, Saarela et al. 2017b).

**NORTHWEST TERRITORIES. Boot Inl.**: *Gillespie et al. 9520* (ALA, ari, CAN, O). **Kuujjua R.**: *Gillespie et al. 9790* (ALA, CAN, MT, O). **Minto Inl. (head)**: *Edlund 48* (CAN), *Gillespie et al. 10199* (CAN), *10248* (ALA, CAN, MT, O), *9461* (ALA, CAN, MT, O). **Richard Collinson Inl.**: *Edlund 131* (CAN), *Stretton 218* (DAO). **Ulukhaktok**: *Bandringa 309* (CAN), *Edlund 296*, *639* (CAN), *Gray & Gibbard 33* (DAO), *Oldenburg 45-1631* (CAN). **NUNAVUT. Albert Edward B.**: *Ponomarenko VI-248*, *VI*-*262* (CAN). **Cambridge Bay**: *Bennett 13-0338* (BABY, CAN), *13-0178* (ALA, BABY, chars, od, UBC), *Calder et al. 24200* (DAO), *Edlund & Argus 12624* (CAN), *Fortier 20* (CAN), *Gillespie et al. 8382* (ALA, CAN, MT, O, UBC, Z), *Gould s.n.* (ALA), *Polunin s.n.* (CAN, 2 sheets), *Porsild 21644* (CAN), *Stephens 938* (CAN, KANU), *946* (CAN, KANU). **Clouston B.**: *Gillespie et al. 7724* (CAN, O, Z). **Falaise B.**: *Eriksen et al. 954* (ALA). **Ferguson L. [Tahiryuaq**]: *Hainault 1970* (DAO). **Greiner L.**: *Ponomarenko VI-033*, *VI-099*, *VI-108*, *VI-128*, *VI-232* (CAN). **Hadley B.**: *Edlund 124*, *340* (CAN), *78* (CAN, mixed with P. langsdorffii subsp. arctica). **Johansen B.**: *Gillespie et al. 7936* (CAN). **Mt. Bumpus**: *Edlund 245* (CAN). **Murray Pt.**: *Gillespie et al. 8173* (CAN, O, Z). **Namaycush L.**: *Edlund & Roncato-Spencer 59* (CAN), *Edlund 139* (CAN). **Oterkvik Pt.**: *Gillespie et al. 7506* (ALA, ALTA, BABY, CAN, MT, O, UBC, Z), *7715* (CAN), *7792* (CAN, Z).

***Pedicularis arctoeuropaea*** (Hultén) Molau *&* D. F. Murray (P. sudetica subsp. arctoeuropaea Hultén), Figs 69D, 70D, E–Arctoeuropean lousewort | European (N)–Asian (N)–amphi-Beringian

Previously recorded from Richard Collinson Inl. and Storkerson P. (Aiken et al. 2007), and newly recorded from Boot Inl., Cambridge Bay, Johansen B., Kuujjua R., the head of Minto Inl., “Oldenburg L.”, Richardson I. and Sinclair Cr. Elsewhere in the Canadian Arctic recorded from Banks, Coats and Melville islands and a few mainland sites (Aiken et al. 2007, Saarela et al. 2013, Saarela et al. 2017b). Earlier treatments (Porsild 1955, 1957, 1964, Porsild and Cody 1980), Thannheiser et al. (2001) included this species and the previous one in *Pedicularis sudetica*.

**NORTHWEST TERRITORIES. Boot Inl.**: *Gillespie et al. 9625* (CAN), *9628* (ALA, ari, CAN, O). **Kuujjua R.**: *Gillespie et al. 9923* (CAN). **Minto Inl. (head)**: *Gillespie et al. 10249* (ALA, CAN, MT, O). “**Oldenburg L.**”: *Oldenburg 45-1358* (CAN, GH). **NUNAVUT. Cambridge Bay**: *Oldenburg 44*-*923B*, *44*-*928* (CAN). **Read I.**: *Oldenburg 42-528* (CAN), *Ross 3A* (GH). **Sinclair Cr.**: *Gillespie et al. 8288* (CAN, O, Z). **Storkerson P.**: *Edlund 240* (CAN).

***Pedicularis capitata*** Adams, Figs 69E, 70F–Capitate lousewort | Asian (N)–amphi-Beringian–North American (N)

Previously recorded from Byron B., Cambridge Bay, Hadley B., “Long L.”, the head of Minto Inl. (Porsild obs., conf.), Mt. Bumpus, Mt. Pelly, Prince Albert S. (Porsild obs.), inland from the head of Prince Albert S., Tahoe L. (Porsild obs.), Ulukhaktok and Wollaston P. (Simmons 1913, Porsild 1955, 1957, 1964, Porsild and Cody 1980, Aiken et al. 2007). Thannheiser et al. (2001) additionally recorded it from Johansen B. (conf.), Richardson I., Surrey L. and Wellington B. Newly recorded from Boot Inl., Colville Mts., Falaise B., Ferguson L., Greiner L., Kuujjua R., Mt. Lady Pelly, Namaycush L., Oterkvik Pt., Richard Collinson Inl., Sinclair Cr., Walker B., Washburn L. and Wollaston P. Elsewhere in the Canadian Arctic recorded from Akpatok, Axel Heiberg, Baffin, Banks, Bylot, Coats, Devon, Ellesmere, Igloolik, King William, Prince of Wales, Somerset and Southampton islands and across the western mainland (Porsild and Cody 1980, Korol 1992, Cody and Reading 2005, Aiken et al. 2007, Saarela et al. 2013, Saarela et al. 2017b, Garneau 2018b).

**NORTHWEST TERRITORIES. Boot Inl.**: *Gillespie et al. 9509* (ALA, CAN, MT, O). **Byron B.**: *Dushenko 5* (UVIC). **Kuujjua R.**: *Gillespie et al. 9742* (ari, CAN), *9992* (CAN, O). **Minto Inl. (head)**: *Edlund 47*, *602* (CAN), *Gillespie et al. 9465* (CAN). **Richard Collinson Inl.**: *Edlund 130*, *485* (CAN). **Ulukhaktok**: *Edlund 287* (CAN), *Gray & Gibbard 37* (DAO), *Oldenburg 42-16*, *45-1632* (CAN), *45-1633* (CAN, GH), *54-217, 54-217a* (GH), *Porsild 17331* (CAN), *Ross 8A* (ALTA), *8B* (GH) *Saarela & Bull 1489* (CAN), *Stretton 53* (DAO). **Walker B.**: *Oldenburg 45-1539* (CAN, GH). **Wollaston P.**: *Oldenburg 54-514* (GH). **NUNAVUT. Cambridge Bay**: *Bennett et al. 13-0174* (chars, od), *Calder et al. 24159* (DAO), *Edlund & Argus 12667* (CAN), *Fortier 11* (CAN), *Gillespie et al. 8387* (ALA, CAN, O, Z), *Oldenburg 44-889* (CAN, GH), *Polunin s.n.* (CAN), *Stephens 962* (KANU), *Sweatman & Smith 18* (DAO). **Colville Mts.**: *Gillespie et al. 7773* (CAN). **Falaise B.**: *Eriksen et al. 932* (ALA). **Ferguson L. [Tahiryuaq**]: *Hainault 2060* (DAO). **Greiner L.**: *Ponomarenko VI-121A*, *VI-215* (CAN). **Hadley B.**: *Edlund 83*, *158* (CAN). **Johansen B.**: *Gillespie et al. 7818* (ALA, ALTA, BABY, CAN, MT, O, UBC, US, Z). “**Long L.**”: *Lambert s.n.* (CAN, 2 sheets). **Mt. Bumpus**: *Edlund 257* (CAN). **Mt. Lady Pelly [Amaaqtuq**]: *Jones & Hainault 1886* (DAO). **Ovayok TP**: *Stephens 987* (CAN). **Namaycush L.**: *Edlund & Roncato-Spencer 61* (CAN). **Oterkvik Pt.**: *Gillespie et al. 7517* (CAN, Z), *7800* (CAN). **Prince Albert S. (head)**: *Edlund 92* (CAN). **Sinclair Cr.**: *Gillespie et al. 8291* (CAN). **Washburn L.**: *Oldenburg 46-2146* (CAN). **Wollaston P.**: *D. Jenness 307b* (CAN).

***Pedicularis hirsuta* L**., Fig. 69F–Hairy lousewort | Circumpolar

Thannheiser et al. (2001) reported this species from Mt. Pelly. The only confirmed record is one newly reported here, gathered at Walker B. in 1945. Elsewhere in the Canadian Arctic recorded from Axel Heiberg, Baffin, northwestern Banks, Bathurst, Bylot, Coats, Cornwallis, Devon, Digges, Ellesmere, Melville, Nottingham, Prince Charles, Somerset and Southampton islands and mainland sites from southwest of Bathurst Inl. eastwards (Aiken et al. 2007, Sokoloff et al. 2016, Garneau 2018c). The general status rank of this species in Northwest Territories is Undetermined (Working Group on General Status of NWT Species 2016).

**NORTHWEST TERRITORIES. Walker B.**: *Oldenburg 45-1522A* (CAN).

***Pedicularis lanata*** Willd. ex Cham. *&* Schltdl., Figs 69G, 70G–Woolly lousewort | Amphi-Beringian–North American (N)

Previously recorded from Byron B., Cambridge Bay, Greely Haven, Hadley B., the head of Minto Inl. (Porsild obs., conf.), Namaycush L., the head of Prince Albert S. (Porsild obs., conf.), Read I., Richard Collinson Inl., Ulukhaktok and Wollaston P. (Macoun and Holm 1921, Porsild 1955, 1957, 1964, Porsild and Cody 1980, Aiken et al. 2007). Thannheiser et al. (2001) additionally recorded it from Johansen B. (conf.), Mt. Pelly, Surrey L. and Wellington B. Newly recorded from “30-Mile Cr.”, Boot Inl., south of Burns L., C. Wollaston, Ferguson L., Greiner L., Kuujjua R., “Long L.”, Mt. Lady Pelly, Murray Pt., Prince Albert P., the north side of Prince Albert S., Walker B. and Washburn L. Elsewhere in the Canadian Arctic recorded from Axel Heiberg, Baffin, Banks, Bathurst, Bylot, Devon, Digges, Eglinton, Ellesmere, King William, Melville, Nottingham, Prince of Wales, Somerset and Southampton islands and across the mainland (Porsild and Cody 1980, Korol 1992, Cody et al. 2003, Aiken et al. 2007, Saarela et al. 2013, Saarela et al. 2017b, Garneau 2018c).

**NORTHWEST TERRITORIES. Boot Inl.**: *Gillespie et al. 9508* (CAN), *9677* (ALA, CAN, O). **Burns L. (S)**: *Edlund 51* (CAN). **C. Wollaston**: *Edlund 3* (CAN). **Kuujjua R.**: *Gillespie et al. 9765* (ALA, CAN, O), *Stretton 68* (DAO). **Minto Inl. (head)**: *Edlund 145* (CAN), *Gillespie et al. 10306* (CAN, O), *9478* (CAN). “**Oldenburg L.**”: *Oldenburg 45-1360* (CAN, GH). **Prince Albert P.**: *Oldenburg 54-258* (GH). **Prince Albert S. (head)**: *Stretton 11*, *19* (DAO) **. Prince Albert S. (N)**: *Oldenburg 46-2280* (CAN). **Prince Albert S. (S)**: *Edlund 533* (CAN). **Richard Collinson Inl.**: *Edlund 184* (CAN), *Stretton 219* (DAO). **Ulukhaktok**: *Edlund 297* (CAN), *Gray & Gibbard 7* (DAO), *Porsild 17327*, *17332* (CAN), *Ross 1A* (ALTA), *1B* (GH), *Saarela & Bull 1504* (ari, CAN, O), *Salokangas 21* (CAN), *Stretton 61* (DAO). **Walker B.**: *Oldenburg 45-1540* (CAN). **NUNAVUT. “30-Mile Cr.**”: *Bennett et al. 14-0362* (UBC). **Cambridge Bay**: *Bennett et al. 13-0257* (BABY, chars, UBC), *14-0380* (UBC), *Calder et al. 24160* (DAO), *Edlund & Argus 12628*, *12672* (CAN), *Gillespie et al. 8415* (CAN, Z), *Oldenburg 44*-*923* (CAN), *44-930* (CAN, GH), *Polunin s.n.* (CAN, 2 sheets), *Stephens 1209* (CAN, KANU), *Stephens 943* (KANU), *Sweatman & Smith 4* (DAO), *Tasker 7648* (CAN). **Ferguson L. [Tahiryuaq**]: *Hainault 1927* (DAO). **Greiner L.**: *Ponomarenko VI-026*, *VI-222* (CAN). **Greely Haven**: *Fortier 92* (CAN). **Hadley B.**: *Edlund 11*, *109* (CAN). “**Long L.**”: *Lambert s.n.* (CAN). **Mt. Lady Pelly [Amaaqtuq**]: *Hainault 1800* (DAO). **Murray Pt.**: *Stretton 30* (DAO). **Namaycush L.**: *Edlund & Roncato-Spencer 7*, *19*, *110* (CAN). **Oterkvik Pt.**: *Gillespie et al. 7461* (ALA, CAN, O, Z), *7492*, *7508* (CAN, Z). **Read I.**: *Oldenburg 43-912*, *43-936*, *43-969* (CAN), *Porsild 17210* (CAN), *Ross 30A* (GH), *30B* (ALTA). **Washburn L.**: *Oldenburg 46-2147* (CAN). **Wollaston P.**: *D. Jenness 283b* (CAN).

***Pedicularis langsdorffii*** subsp. ***arctica*** (R.Br.) Pennell ex Hultén (*P. arctica* R.Br.), Fig. 69H–Arctic lousewort | Asian (NE)–Amphi-Beringian–North American (N)

Previously recorded from Cambridge Bay, the head of Minto Inl. (Porsild obs., conf.), the head of Prince Albert S. (Porsild obs., conf.), Read I. (Porsild obs.) and Ulukhaktok (Simmons 1913, Porsild 1955, 1957, 1964, Porsild and Cody 1980, Aiken et al. 2007). Thannheiser et al. (2001) additionally recorded it from Hadley B. (conf.), Mt. Pelly, Surrey L. and Wellington B. Newly recorded from Boot Inl., Kuujjua R., Falaise B., Ferguson L., Greiner L., Johansen B., “Long L.”, Namaycush L., Oterkvik Pt., Sinclair Cr., Storkerson P. and Walker B. Elsewhere in the Canadian Arctic recorded from Axel Heiberg, Banks, Bylot, Devon, Ellesmere, Melville, Prince of Wales and Somerset islands and mainland sites from Boothia P. westwards (Porsild and Cody 1980, Aiken et al. 2007, Saarela et al. 2013, Sokoloff et al. 2016, Saarela et al. 2017b).

**NORTHWEST TERRITORIES. Boot Inl.**: *Gillespie et al. 9519* (CAN, O), *9625* (CAN, mixed with *P. arctoeuropaea*). **Burns L. (S)**: *Edlund 65* (CAN). **Kuujjua R.**: *Gillespie et al. 9798* (ALA, CAN, O). **Minto Inl. (head)**: *Edlund 49* (CAN), *Gillespie et al. 9479*, *10247* (CAN). **Ulukhaktok**: *Edlund 343*, *747* (CAN), *Oldenburg 42-16A*, *45-1634* (CAN), *Ross 1B* (GH), *Saarela & Bull 1496* (ari, CAN, O). **Walker B.**: *Oldenburg 45-1521A* (CAN). **Wollaston P.**: *Oldenburg 54-516* (GH). **NUNAVUT. Cambridge Bay**: *Bennett et al. 13-0168* (chars, V), *Calder et al. 24201* (DAO), *Edlund & Argus 12671* (CAN), *Gillespie et al. 8472* (CAN), *Oldenburg 44*-*930B* (CAN), *Polunin s.n.* (CAN, 2 sheets), *Porsild 21642* (CAN), *Stephens 1208* (KANU), *944* (CAN, KANU), *947* (CAN), *Sutton 1046* (CAN). **Falaise B.**: *Eriksen et al. 955* (ALA). **Ferguson L. [Tahiryuaq**]: *Hainault 1963* (DAO). **Greiner L.**: *Ponomarenko VI-163* (CAN). **Hadley B.**: *Edlund 78* (CAN, mixed with *P. albolabiata*), *Gould s.n.* (ALA). **Johansen B.**: *Gillespie et al. 7896* (CAN, O). “**Long L.**”: *Lambert s.n.* (CAN). **Namaycush L.**: *Edlund & Roncato-Spencer 60*, *111* (CAN). **Oterkvik Pt.**: *Gillespie et al. 7507* (ALA, CAN, MT, O, UBC, Z). **Sinclair Cr.**: *Gillespie et al. 8287* (CAN, Z). **Storkerson P.**: *Edlund 305* (CAN).

### Asterales

**Asteraceae [13/25/26]**

**Key to Asteraceae [adapted from Porsild and Cody (1980), Barkley (2006) and Barkley et al. (2006)]**

**Table:** 

1	Heads liguliflorous (all florets ligulate [bisexual with zygomorphic corollas])	**2**
–	Heads not liguliflorous (not all florets ligulate, ray flowers when present pistillate or neuter)	**3**
2	Heads 5–80+, born in cymiform arrays; florets 9–12 per head	***Askellia pygmaea***
–	Heads born singly; florets (15–)30–85+ per head	*** Taraxacum ***
3	Heads composed of central disc florets and marginal ray florets	**4**
–	Heads composed entirely of disc florets	**12**
4	Rays yellow	**5**
–	Rays white, pink or purple	**7**
5	Leaves mostly opposite, distal-most ones sometimes alternate	***Arnica angustifolia***
–	Leaves alternate	**6**
6	Phyllaries glabrous or sparsely puberulent adaxially, tips black	***Senecio lugens***
–	Phyllaries villous adaxially, tips green or yellowish green, sometimes pinkish	*** Tephroseris ***
7	Pappi of bristles (at least in part)	**8**
–	Pappi of scales, or lacking	**11**
8	Basal leaf blades sagittate, deltate or reniform to cordate; heads appearing before the leaves	**Petasites frigidus subsp. frigidus**
–	Basal leaf blades lanceolate, oblanceolate, spatulate or linear (finely divided in *Tripleurospermum*); heads appearing after the leaves	**9**
9	Phyllaries in (1–)2(–3) series; corollas of disc florets 2.4–5 mm; style-branch appendages deltate	*** Erigeron ***
–	Phyllaries in 3–4(–5) series; corollas of disc florets 5–8.1 mm; style-branch appendages lanceolate	**10**
10	Leaf blade apices pointed, margins often dentate, sometimes entire, abaxial surfaces glabrescent to scabridulous, sparsely villous along veins, adaxial surfaces sparsely to ± densely villous or villoso-strigose; corollas of ray florets 0.8–1.8 mm wide, pappus dark cinnamon or reddish tan; involucres 6–9 mm	***Eurybia sibirica***
–	Leaf blade apices obtuse to blunt, margins entire, abaxial and adaxial surfaces sparsely woolly; corollas of ray florets 2–3.2 mm wide, pappus whitish or yellowish; involucres 9–12.5 mm	***Symphyotrichum pygmaeum***
11	Plants (0.6–)1–12 cm; leaves all or mostly basal, basal ones marcescent, blades linear, not lobed; involucres 4–6.5 mm diam.	***Hulteniella integrifolia***
–	Plants 10–50(–80) cm; leaves basal and cauline, none marcescent, blades oblong, 1–3-pinnately lobed; involucres 8–12+ mm diam.	**Tripleurospermum maritimum subsp. phaeocephalum**
12	Pappus of scales or lacking	*** Artemisia ***
–	Pappus of bristles	**13**
13	Leaf blades (1–)2–3(–4)-ternately lobed or dissected	***Erigeron compositus***
–	Leaf blade entire, subentire, coarsely dentate or subpinnatifid	**14**
14	Phyllaries in (1–)2 series, equal	*** Tephroseris ***
–	Phyllaries in 3–6+ series, unequal	*** Antennaria ***

### *Antennaria* Gaertn. [3]

**Key to *Antennaria* [adapted from Chmielewski (1994) and Chmielewski (1997)]**

**Table:** 

1	Heads usually borne singly, rarely in 2s or 3s	**A. monocephala subsp. angustata**
–	Heads usually 2–6, rarely borne singly	**2**
2	Basal leaf blades spathulate-oblanceolate, densely and loosely strigose-tomentose on both surfaces; stems not stipitate-glandular	**A. media subsp. compacta**
–	Basal leaf blades linear-lanceolate to spathulate-lanceolate, abaxial surfaces tomentose, adaxial green-glabrescent to gray-pubescent; stems stipitate-glandular, hairs purple	**A. friesiana subsp. friesiana**

**Figure 71.**
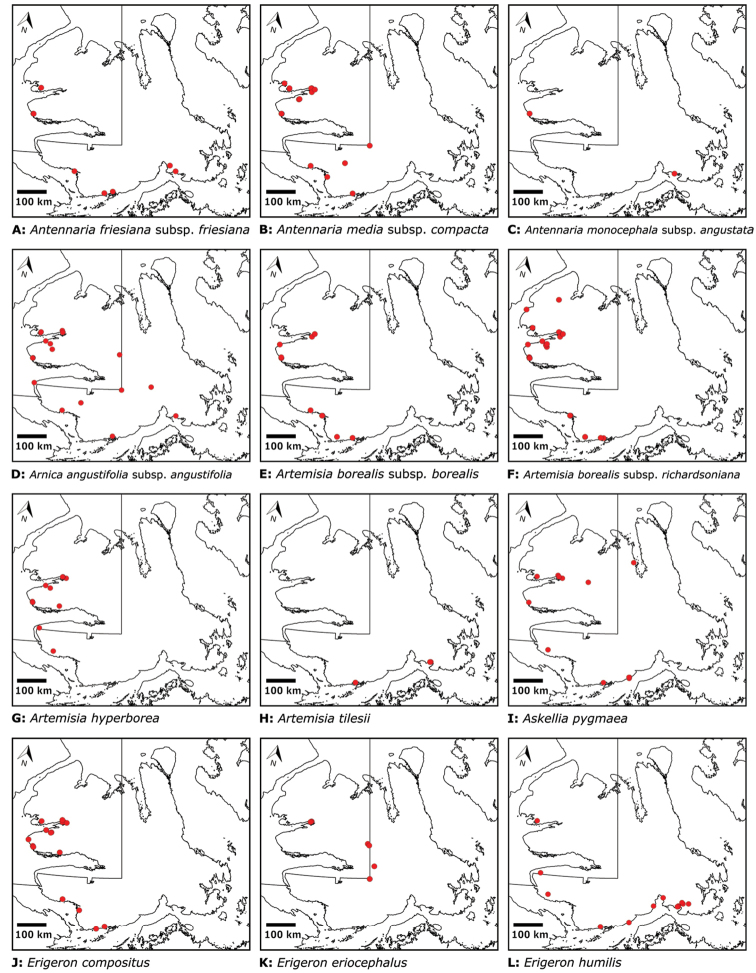
Species distribution maps. Asteraceae: **A**Antennaria friesiana subsp. friesiana**B**Antennaria media subsp. compacta**C**Antennaria monocephala subsp. angustata**D**Arnica angustifolia subsp. angustifolia**E**Artemisia borealis subsp. borealis**F**Artemisia borealis subsp. richardsoniana**G***Artemisia hyperborea***H***Artemisia tilesii***I***Askellia pygmaea***J***Erigeron compositus***K***Erigeron eriocephalus***L***Erigeron humilis*.

***Antennaria friesiana*** (Trautv.) Ekman subsp. ***friesiana*** (*A. ekmaniana* A.E.Porsild), Fig. 71A–Fries’ pussy-toes | Asian (NE)–amphi-Beringian–North American (N)

Previously recorded from Cambridge Bay (Augustus Hills area) and Ferguson L. (Aiken et al. 2007). Thannheiser et al. (2001) additionally recorded it from Johansen B. (conf.), Minto Inl., Richardson I. and Ulukhaktok (conf.). Newly recorded from Boot Inl. and Read I. Elsewhere in the Canadian Arctic recorded from Axel Heiberg, Baffin, Banks, Devon, Ellesmere, Melville and Southampton islands as well as numerous mainland sites (Porsild and Cody 1980, Aiken et al. 2007).

**NORTHWEST TERRITORIES. Boot Inl.**: *Gillespie et al. 9684* (CAN, O). **Ulukhaktok**: *Oldenburg 45-1715* (CAN), *Edlund* 756 (CAN). **NUNAVUT. Cambridge Bay**: *Edlund & Argus 12855* (CAN). **Ferguson L.**: *Hainault 2102A* (DAO) **Johansen B.**: *Gillespie et al.* 7891 (ALA, CAN, MT), *8143* (CAN, MT). **Read I.**: *Oldenburg 43-1085* (CAN).

**Figure 72.**
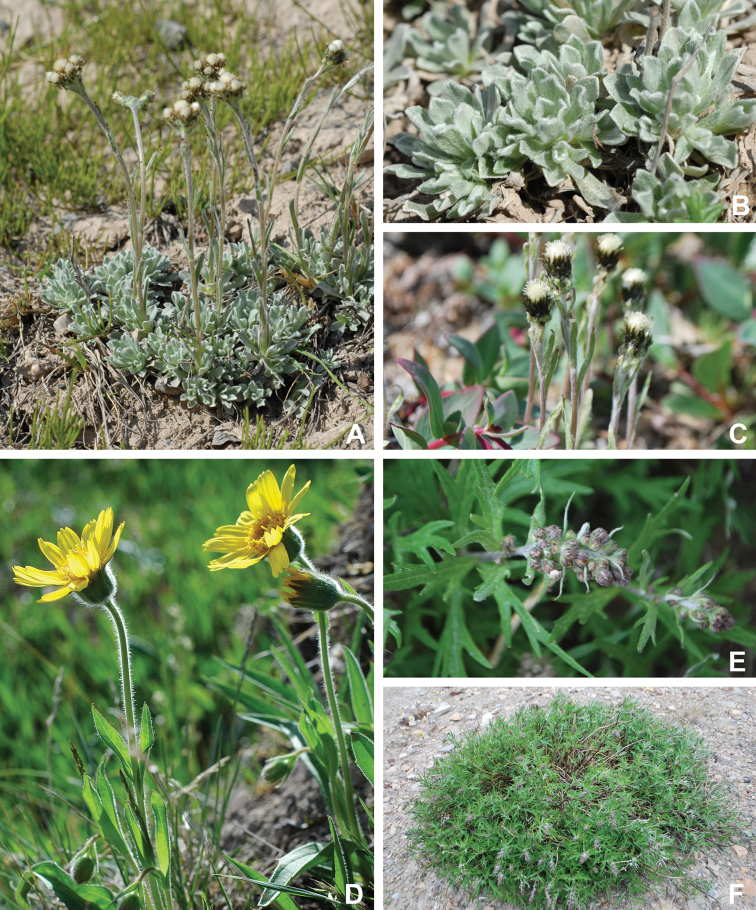
**A**Antennaria media subsp. compacta habit, *Gillespie et al. 9622***B**Antennaria media subsp. compacta basal rosettes, *Gillespie et al. 9622***C**Antennaria monocephala subsp. angustata inflorescences **D**Arnica angustifolia subsp. angustifolia habit, *Gillespie et al. 9607***E***Artemisia tilesii* inflorescence **F***Artemisia tilesii* habit. Photos **A**, **B** by L.J. Gillespie **C**, **E**, **F** by B.A. Bennett and **D** by R.D. Bull.

***Antennaria media*** subsp. ***compacta*** (Malte) Chmiel. (*A. compacta* Malte), Figs 71B, 72A, B–Pussytoes | Amphi-Beringian (E)–North American (N)

Previously recorded from the head of Minto Inl., the head of Prince Albert S., Ulukhaktok and Walker B. (Porsild 1955, 1957, 1964, Porsild and Cody 1980, Aiken et al. 2007). Newly recorded from Clouston B., Johansen B., Kuujjua R. and Mt. Bumpus. Elsewhere in the Canadian Arctic recorded from Axel Heiberg, Baffin, Banks, Eglinton and Ellesmere islands and mainland sites from Nunavut to Yukon (Chmielewski 1997, Aiken et al. 2007). Taxonomy follows Chmielewski (1997) and Elven et al. (2011), not Bayer (2006), who included this taxon in a broadly circumscribed *A. alpina*.

**NORTHWEST TERRITORIES. Boot Inl.**: *Gillespie et al. 9592b* (CAN), *9622* (CAN, MT, O). **Kuujjua R.**: *Gillespie et al. 9732* (ALA, CAN, O), *9838* (CAN). **Minto Inl. (head)**: *Edlund 83*, *608* (CAN), *Gillespie et al. 10176* (ALA, CAN, MT), *10266* (CAN), *Porsild 17419* (CAN). **Ulukhaktok**: *Edlund 841* (CAN), *Oldenburg 42-36*, *42-97e* (CAN), *Porsild 17333*, *17335* (CAN). **Walker B.**: *Porsild 17499* (CAN). **Clouston B.**: *Gillespie et al. 7736* (CAN, MT, O). **NUNAVUT. Falaise B.**: *Eriksen et al. 994* (ALA). **Johansen B.**: *Gillespie et al. 8121* (CAN, MT). **Mt. Bumpus**: *Edlund 264* (CAN). **Prince Albert S. (head)**: *Edlund 98* (CAN).

***Antennaria monocephala*** subsp. ***angustata*** (Greene) Hultén, Figs 71C, 72C–Pygmy pussy-toes | Amphi-Beringian–North American (N)

Newly recorded for Victoria I. based on collections from Cambridge Bay (Long Point area) and Ulukhaktok. Elsewhere in the Canadian Arctic recorded from Banks, Coats, Devon, Melville and Nottingham islands and mainland sites (Porsild and Cody 1980, Chmielewski and Chinnappa 1991, Aiken et al. 2007, Saarela et al. 2013, Saarela et al. 2017b).

**NORTHWEST TERRITORIES. Ulukhaktok**: *Edlund 733*, *842* (CAN). **NUNAVUT. Cambridge Bay**: *Bennett et al. 13-0303* (BABY, CAN, WTU).

### *Arnica* L. [1]

***Arnica angustifolia*** Vahl subsp. ***angustifolia*** (Arnica alpina subsp. angustifolia (J.Vahl) Maguire), Figs 71D, 72D–Alpine arnica | North American (N)–amphi-Atlantic (W)

Previously recorded from Cambridge Bay (Augustus Hills), Kuujjua R., the head of Minto Inl., Ulukhaktok, Washburn L. and Wollaston P. (Macoun and Holm 1921, Porsild 1955, 1957, 1964, Porsild and Cody 1980, Aiken et al. 2007). Thannheiser et al. (2001) additionally recorded it from Johansen B. (conf.). Newly recorded from Boot Inl., south of Burns L., C. Baring, Falaise B. and southeast of the head of Prince Albert S. Elsewhere in the Canadian Arctic recorded from Baffin, Banks, Ellesmere, Emerald, Melville and Somerset islands and mainland sites (Porsild and Cody 1980, Cody and Reading 2005, Aiken et al. 2007, Saarela et al. 2013, Saarela et al. 2017b).

**NORTHWEST TERRITORIES. Boot Inl.**: *Edlund* 578 (CAN), *Gillespie et al. 9545*, *9598* (CAN), *9607* (ALA, CAN, O). **Burns L. (S)**: *Edlund 553* (CAN). **C. Baring**: *Edlund 410* (CAN). **Kuujjua R.**: *Dutilly 18842* (QFA), *Gillespie et al. 9955* (ALA, CAN, MT, O, UBC, US, WIN), *9985* (ALA, CAN, O). **Minto Inl. (head)**: *Edlund 150* (CAN), *Gillespie et al. 10031* (ALA, CAN, MT, O, UBC), *10138* (ari, CAN, MT, O, UBC, WIN). **Ulukhaktok**: *Bliss s.n.* (ALTA, DAO), *Dutilly 18656* (DAO, QFA), *Edlund 710* (CAN), *Oldenburg 42-32* (CAN), *54*-*207* (UBC), *Porsild 17336* (CAN), *Ross 32a* (ALTA). **NUNAVUT. Cambridge Bay**: *Edlund & Argus 12854* (CAN). **Falaise B.**: *Eriksen et al. 933* (ALA). **Johansen B.**: *Gillespie et al. 7869* (CAN, MT). **Prince Albert S. (head)**: *Edlund 150* (CAN). **Washburn L.**: *Porsild 17460* (CAN). **Wollaston P.**: *D. Jenness 335b* (CAN).

### *Artemisia* L. [3/4]

**Key to *Artemisia* [adapted from Shultz (2006)]**

**Table:** 

1	Plants rhizomatous; leaf blades coarsely pinnately lobed, 2–5(–6) cm wide	***A. tilesii***
–	Plants cespitose; leaf blades finely and deeply 1–3-pinnately or -palmately lobed, 0.4–1 cm wide	**2**
2	Involucres broadly campanulate, 4.5–8 mm wide; corollas funnelform; leaves densely covered in silvery or whitish hairs	***A. hyperborea***
–	Involucres hemispheric, 3.5–4 mm wide; corollas subglobose; leaves sparsely to densely covered in whitish or pale yellowish hairs, glabrate or glabrous (*A. borealis*)	**3**
3	Plants glabrous, glabrate or sparsely hairy; corollas 2.2–3 mm, usually yellow-orange (at least lobes)	**A. borealis subsp. borealis**
–	Plants densely hairy; corollas 3–3.5 mm, deep red (at least lobes)	**A. borealis subsp. richardsonii**

***Artemisia borealis*** Pall. subsp. ***borealis***, Fig. 71E–Boreal wormwood | European (NE)–Asian(N/C)–amphi-Beringian–Cordilleran–North American (N)

Previously recorded from C. Wollaston, the head of Minto Inl., the north side of Prince Albert S. (voucher not located), Read I. and northwestern Wollaston P. (voucher not located) (Porsild 1955, 1957, 1964, Porsild and Cody 1980, Aiken et al. 2007). Thannheiser et al. (2001) additionally recorded it from Johansen B. (conf.). Newly recorded from Falaise B., Oterkvik Pt. and Ulukhaktok. Elsewhere in the Canadian Arctic recorded from southern Baffin I., Banks I. and a few mainland sites (Porsild and Cody 1980, Gould and Walker 1997, Aiken et al. 2007, Saarela et al. 2013, Saarela et al. 2017b).

**NORTHWEST TERRITORIES. C. Wollaston**: *Edlund 31* (CAN). **Minto Inl. (head)**: *Gillespie et al. 10292* (ALA, CAN, O, UTC), *Porsild 17423* (CAN). **Ulukhaktok**: *Edlund 464*, *732*, *865* (CAN), *Saarela & Bull 1507* (ALA, CAN, O, UTC). **NUNAVUT. Falaise B.**: *Eriksen et al. 946* (ALA). **Johansen B.**: *Gillespie et al. 8120* (CAN, UTC). **Oterkvik Pt.**: *Gillespie et al. 7602* (CAN, O, UTC). **Read I.**: *Oldenburg 43-1077* (CAN), *Porsild 17211* (CAN).

***Artemisia borealis*** Pall. subsp. ***richardsoniana*** (Besser) Korobkov (*Artemisia richardsoniana* Besser), Fig. 71F–Richardson’s wormwood | Asian (NE)–amphi-Beringian–North American (NW)

Previously recorded from C. Wollaston, the head of Minto Inl., the north side of Prince Albert S., Read I., Ulukhaktok and Walker B. (Porsild 1955, 1957, 1964, Porsild and Cody 1980, Aiken et al. 2007). Thannheiser et al. (2001) additionally recorded it from Johansen B. (conf.). Newly recorded from Kuujjua R., Oterkvik Pt. and Richard Collinson Inl. The Johansen B. occurrences mark the known eastern limit of this amphi-Beringian taxon. Elsewhere in the Canadian Arctic recorded from Banks I., mainland Nunavut (Bernard Harbour) and mainland Northwest Territories (Macoun and Holm 1921, Porsild and Cody 1980, Aiken et al. 2007, Saarela et al. 2013).

**NORTHWEST TERRITORIES. C. Wollaston**: *Edlund 19B* (CAN). **Kuujjua R.**: *Dutilly 18840* (QFA), *Edlund 645* (CAN), *Gillespie et al. 9748*, *9885* (ari, CAN, O, UTC). **Minto Inl. (head)**: *Edlund 82*, *168* (CAN), *Gillespie et al. 10107* (CAN, MT, O, UTC), *10293* (CAN), *Porsild 17420*, *17422* (CAN). **Prince Albert S. (N)**: *Stretton 201* (DAO). **Richard Collinson Inl.**: *Edlund 138* (CAN). **Ulukhaktok**: *Dutilly 18635* (QFA), *Edlund 319*, *773*, *866* (CAN), *Oldenburg 45-1716* (CAN), *Saarela & Bull 1442* (CAN, MT, O, UTC). **Walker B.**: *Oldenburg 45-1510* (CAN), *Porsild 17500* (CAN). **NUNAVUT. Johansen B.**: *Gillespie et al. 8080* (CAN, UTC), *8119* (CAN, MT, UTC). **Oterkvik Pt.**: *Gillespie et al. 7468* (CAN, O, UTC), *7603* (CAN, UTC). **Read I.**: *Oldenburg 43-1075*, *43-1076*, *43-919*, *43-934* (CAN), *Porsild 17212*, *17213* (CAN), *Ross 32B* (ALTA).

***Artemisia hyperborea*** Rydb., Fig. 71G–Northern wormwood | American Beringian

Previously recorded from the head of Minto Inl., Ulukhaktok and Wollaston P. (Porsild 1955, Porsild and Cody 1980). Despite these previous collections–all confirmed–Aiken et al. (2007) did not map the taxon for Victoria I. Thannheiser et al. (2001) additionally recorded it from Johansen B. Newly recorded from the north side of Prince Albert S. Elsewhere in the Canadian Arctic known from Banks I. and western mainland sites as far east as the Coppermine R. valley, Nunavut (Porsild and Cody 1980, Aiken et al. 2007, Saarela et al. 2013, Saarela et al. 2017b).

**NORTHWEST TERRITORIES. C. Baring**: *Edlund 414* (CAN). **Kuujjua R.**: *Dutilly 18841* (QFA), *18845* (CAN), *Gillespie et al. 9733* (ALA, CAN, MT, O, UTC). **Minto Inl. (head)**: *Edlund 169*, *609* (CAN), *Gillespie et al. 10131* (ALA, CAN, O, UTC), *Porsild 17421* (CAN). **Prince Albert S. (N)**: *Edlund 445* (CAN). **Ulukhaktok**: *Bliss s.n.* (ALTA), *Edlund 318*, *777* (CAN), *Porsild 17338* (CAN), *Saarela & Bull 1419* (ALA, CAN, O, UTC). **Wollaston P.**: *D. Jenness 337a* (*304b*) (CAN).

***Artemisia tilesii*** Ledeb., Figs 71H, 72E, F–Tilesius’s wormwood | European (NE)–Asian (N)–amphi-Beringian–North American (N)

Newly recorded from Victoria I., where known from one collection from Johansen B. and two from Cambridge Bay. The Cambridge Bay collections, taken in 2013, were discovered growing in a disturbed lot near Elk’s Lodge with *Descurainia sophioides*, *Saxifraga cernua* and *Puccinellia* sp. (likely *P. nuttalliana*) and at the Defence Early Warning station in disturbed gravel at top of gray water outfall. Our 2008 collection was made on the Johansen B. airstrip, growing on sandy gravel substrate. Elsewhere in the Canadian Arctic recorded from Banks I. and mainland sites (Porsild and Cody 1980, Cody et al. 2003, Cody and Reading 2005, Aiken et al. 2007, Saarela et al. 2013, Saarela et al. 2017b).

**NUNAVUT. Cambridge Bay**: *Bennett 13-0299* (UBC), *13-0323* (BABY, CAN, chars). **Johansen B.**: *Gillespie et al. 8079* (CAN, UTC).

### *Askellia* W.A.Weber [1]

**Figure 73.**
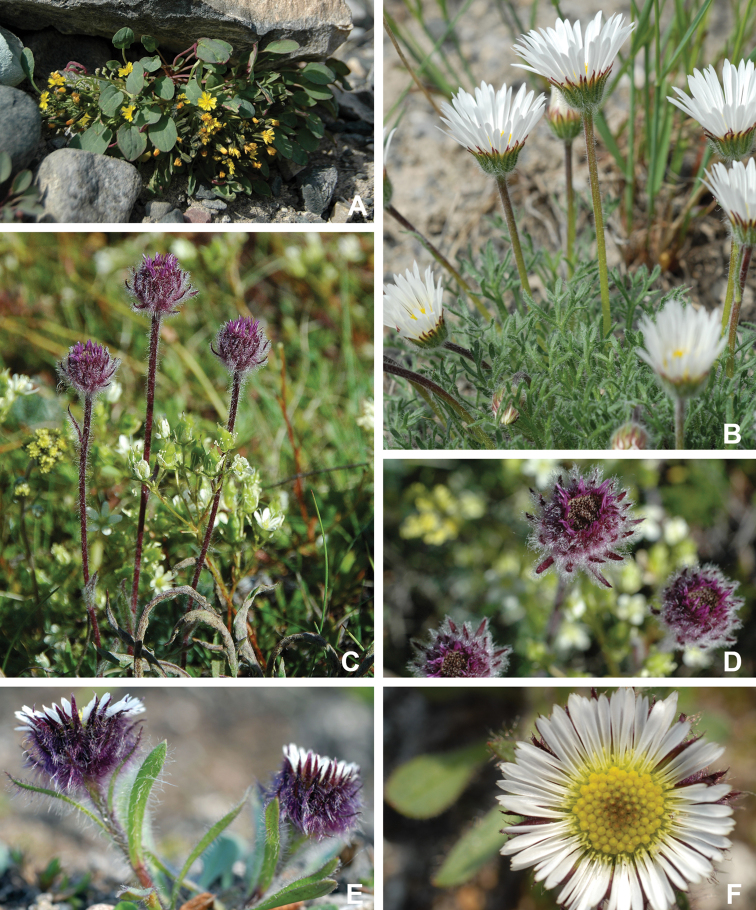
**A***Askellia pygmaea* habit, *Gillespie et al. 10087***B***Erigeron compositus* habit, *Gillespie et al. 9832***C***Erigeron eriocephalus* habit, *Gillespie et al. 10189***D***Erigeron eriocephalus* inflorescences, *Gillespie et al. 10189***E***Erigeron humilis* habit, *Gillespie et al. 8087***F***Erigeron humilis* inflorescence, *Gillespie et al. 8087.* Photos **A**, **C–F** by R.D. Bull and **B** by L.J. Gillespie.

***Askellia pygmaea*** (Ledeb.) Sennikov (*Crepis nana* Richardson, *Askellia nana* (Richardson) W.A.Weber), Figs 71I, 73A–Dwarf alpine hawks-beard | Asian (C-NE)–amphi-Beringian–North American (NW)

Previously recorded from Byron B., “Jackpot L.”, the head of Minto Inl. and Wollaston P. (Macoun and Holm 1921, Porsild 1955, 1957, 1964, Porsild and Cody 1980, Aiken et al. 2007). Thannheiser et al. (2001) additionally recorded it from Hadley B. (conf.), Johansen B. (conf.) and Ulukhaktok (conf.). Newly recorded from Boot Inl. and Sinclair Cr. The taxon was observed and photographed on 30 July 2015 by J. Wagner in the Cambridge Bay area on the shore at the first cape to the east of Long Point (image! BAB and JMS); a voucher is needed from this locality. Elsewhere in the Canadian Arctic recorded from Baffin, Banks, Melville, Prince Patrick and Southampton islands and scattered mainland sites (Porsild and Cody 1980, Gould and Walker 1997, Cody and Reading 2005, Aiken et al. 2007, Saarela et al. 2013, Saarela et al. 2017b).

**NORTHWEST TERRITORIES. Boot Inl.**: *Gillespie et al. 9525* (CAN, O). “**Jackpot L.**”: *Porsild 17506* (CAN). **Minto Inl. (head)**: *Edlund 597*, *70* (CAN), *Gillespie et al. 10087* (ALA, ari, CAN), *10169* (CAN), *Washburn 17424* (CAN). **Ulukhaktok**: *Svoboda 745023* (UBC). **NUNAVUT. Byron B.**: *Dushenko 42* (UVIC). **Hadley B.**: *Edlund 76* (CAN). **Johansen B.**: *Gillespie et al. 8074* (ALA, ALTA, BABY, CAN, MT, O, UBC). **Sinclair Cr.**: *Gillespie et al. 8242* (CAN, MT), *8320* (CAN, MT). **Wollaston P.**: *D. Jenness 403* (CAN).

### *Erigeron* L. [4]

**Key to *Erigeron* [adapted from Nesom (2006)]**

**Table:** 

1	Leaf blades (1–)2–3(–4)-ternately deeply lobed or dissected	***E. compositus***
–	Leaf blades not lobed or dissected	**2**
2	Ray laminae erect, filiform, 3–6(–8) × 0.3–1 mm	***E. porsildii***
–	Ray laminae spreading, strap-shaped, 13–17 × 1.2–1.7 mm	3
3	Hairs of phyllaries and distal stems with dark reddish to blackish-purple cross walls; phyllaries strigoso-hirsute, usually dark purple; involucres 6–9 × 10–15(–20) mm; cypselae 2.2–2.5 mm	***E. humilis***
–	Hairs of phyllaries with clear or sometimes bright reddish cross walls; phyllaries densely lanate (hairs tangled, soft), reddish purple; involucres 8–10(–11) × (10–)15–20(–30) mm; cypselae 1.8–2.2 mm	***E. eriocephalus***

***Erigeron compositus*** Pursh, Figs 71J, 73B–Cut-leaved fleabane | Amphi-Beringian (E)–North American (N)–Cordilleran

Previously recorded from the head of Minto Inl., the west end of Diamond Jenness P., the north side of Prince Albert S. and Ulukhaktok (Porsild 1955, 1957, 1964, Porsild and Cody 1980, Aiken et al. 2007). Thannheiser et al. (2001) additionally recorded it from Johansen B. (conf.), the known eastern limit of the taxon on Victoria I. Newly recorded from Boot Inl., Clouston B., Falaise B., Kuujjua R. and Oterkvik Pt. Elsewhere in the Canadian Arctic recorded on mainland Northwest Territories and Nunavut as far east as Bathurst Inl. (Cody et al. 1984a, Saarela et al. 2013, Saarela et al. 2017b), and on Axel Heiberg, Baffin, Banks, Ellesmere, Melville and adjacent islands, with a conspicuous gap in the central Arctic islands (Aiken et al. 2007).

**NORTHWEST TERRITORIES. Boot Inl.**: *Gillespie et al. 9529* (CAN, O). **Diamond Jenness P.**: *Stretton 86* (DAO). **Kuujjua R.**: *Gillespie et al. 9734* (ari, CAN, MT, O), *9832* (ALA, ari, CAN, O). **Minto Inl. (head)**: *Edlund 166* (CAN), *Gillespie et al. 10065* (ALA, ari, CAN, MT, UBC), *10190* (CAN, O), *Porsild 17425* (CAN). **Prince Albert S. (N)**: *Edlund 444* (CAN). **Ulukhaktok**: *Edlund 499*, *788* (CAN), *Porsild 17340* (ALTA, CAN), *Ross 30* (ALTA). **Kuujjua R.**: *Dutilly 18778*, *18779*, *18780*, *18781*, *18782* (QFA). **NUNAVUT. Clouston B.**: *Gillespie et al. 7726* (ALA, ALTA, BABY, CAN, MT, O, UBC). **Falaise B.**: *Eriksen et al. 974* (ALA). **Johansen B.**: *Gillespie et al. 8122* (ALA, BABY, CAN, MT, O, UBC). **Oterkvik Pt.**: *Gillespie et al. 7692* (CAN, MT).

***Erigeron eriocephalus*** J.Vahl (Erigeron uniflorus L. subsp. eriocephalus (J.Vahl) Cronquist), Figs 71K, 73C, D–Woolly-headed fleabane | Circumpolar

Previously reported from south of Burns L., east of the head of Prince Albert S., the head of Minto Inl. (Porsild obs., conf.) and Ulukhaktok (Porsild obs.) (Porsild 1955, 1957, 1964, Porsild and Cody 1980, Aiken et al. 2007). Records mapped from Cambridge Bay by Aiken et al. (2007) (*Edlund & Argus 12673*, *12883*) have been re-determined as *E. humilis*. Thannheiser et al. (2001) additionally recorded it from Richardson I. Elsewhere in the Canadian Arctic recorded from Axel Heiberg, Baffin, Banks, Coats, Melville, Prince Patrick and Southampton islands (with a conspicuous gap in distribution in the central Arctic islands) and scattered mainland sites (Porsild and Cody 1980, Korol 1992, Cody and Reading 2005, Aiken et al. 2007, Saarela et al. 2013, Saarela et al. 2017b).

**NORTHWEST TERRITORIES. Burns L. (S)**: *Edlund 557*, *s.n.* (CAN). **Minto Inl. (head)**: *Edlund 599*, *89* (CAN), *Gillespie et al. 10137* (ALA, ALTA, ari, CAN, MT, UBC, US, WIN), *10165* (CAN, O), *10189* (CAN, O), *10114* (ALA, CAN, MT, O). **NUNAVUT. Prince Albert S. (head)**: *Edlund 87*, *151* (CAN), *Edlund & Argus 12816* (CAN).

***Erigeron humilis*** Graham, Figs 71L, 73E, F–Low fleabane | Amphi-Beringian–North American (N)–amphi-Atlantic (W)

Previously recorded from Cambridge Bay and Wollaston P. (Macoun and Holm 1921, Aiken et al. 2007). Thannheiser et al. (2001) additionally recorded it from Hadley B., Johansen B. (conf.), the head of Minto Inl., Surrey L. and Ulukhaktok. Newly recorded from “30-Mile Cr.”, Boot Inl., Ferguson L., Greiner L., Mt. Pelly, Sinclair Cr. and NW Wollaston P. Elsewhere in the Canadian Arctic recorded from Baffin, Banks, Coats, Ellesmere and Southampton islands and scattered mainland sites (Porsild and Cody 1980, Cody et al. 1989, Korol 1992, Aiken et al. 2007, Saarela et al. 2013, Saarela et al. 2017b).

**NORTHWEST TERRITORIES. Boot Inl.**: *Edlund 574* (CAN). **Wollaston P. (NW)**: *Edlund 371* (CAN). **NUNAVUT. “30-Mile Cr.**”: *Bennett et al. 14-0330* (UBC). **Cambridge Bay**: *Edlund & Argus 12673* (CAN), *12883* (ALA, CAN), *Stephens 1143* (CAN, KANU, KSTC), *1251* (CAN, KSTC). **Ferguson L. [Tahiryuaq**]: *Hainault 1999* (DAO). **Greiner L.**: *Ponomarenko VI-287*, *VI-325* (CAN) **Johansen B.**: *Gillespie et al. 8087* (ALA, ALTA, BABY, CAN, MT, O, UBC), *8102* (CAN, MT). **Ovayok TP**: *Stephens 1180* (KANU, KSTC). **Sinclair Cr.**: *Gillespie et al. 8276* (CAN). **Wollaston P.**: *D. Jenness 375a* (CAN).

**Figure 74.**
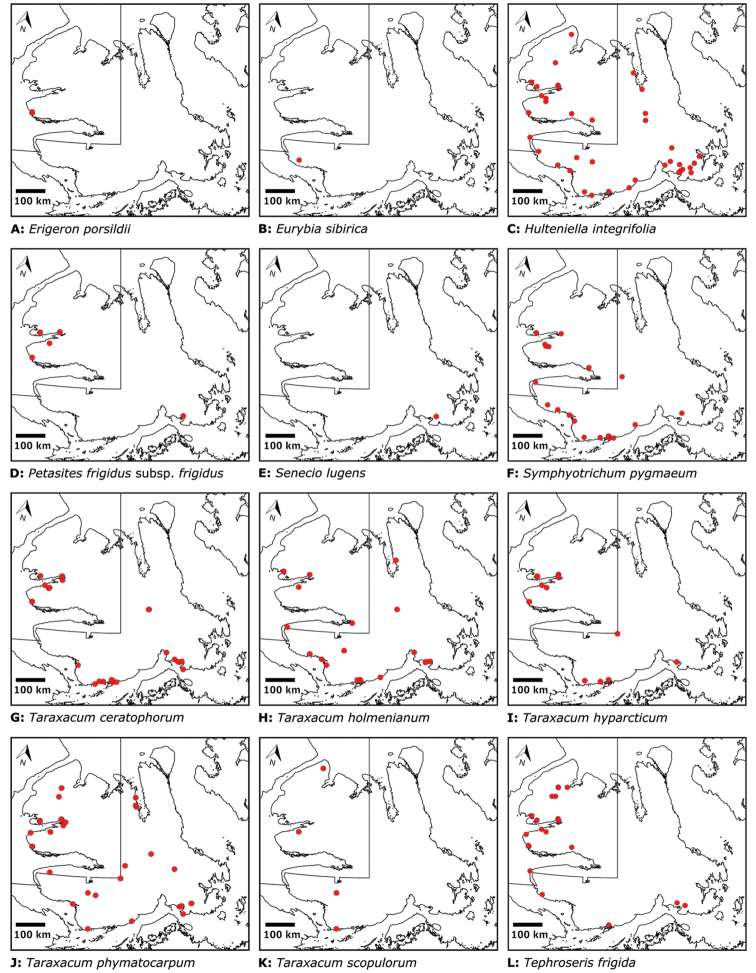
Species distribution maps. Asteraceae: **A***Erigeron porsildii***B***Eurybia sibirica***C***Hulteniella integrifolia***D**Petasites frigidus subsp. frigidus**E***Senecio lugens***F***Symphyotrichum pygmaeum***G***Taraxacum ceratophorum***H***Taraxacum holmenianum***I***Taraxacum hyparcticum***J***Taraxacum phymatocarpum***K***Taraxacum scopulorum***L***Tephroseris frigida*.

***Erigeron porsildii*** G.L.Nesom & D.F.Murray (Erigeron grandiflorus subsp. arcticus A.E.Porsild), Fig. 74A–Porsild’s fleabane | American Beringian

Known only from Ulukhaktok, the type locality (Porsild 1955, Aiken et al. 2007). The most recent collection of the species is from 1959, gathered by Stretton. Elsewhere in the Canadian Arctic recorded from a few mainland Northwest Territories and Yukon sites (Porsild and Cody 1980).

**NORTHWEST TERRITORIES. Ulukhaktok**: *Porsild 17341* (CAN), *17342* (holotype CAN; isotypes ALTA, V, WTU), *Stretton 54* (DAO).

### *Eurybia* (Cass.) Cass. [1]

***Eurybia sibirica*** (L.) G.L.Nesom (*Aster sibiricus* L.), Fig. 74B–Arctic aster | European (N)–Asian (N/C)–amphi-Beringian–Cordilleran

Collected on the Wollaston P. by D. Jenness in 1915, during the Canadian Arctic Expedition (Macoun and Holm 1921). Subsequent authors did not record the species for Victoria I. (Porsild 1957, 1964, Porsild and Cody 1980, Aiken et al. 2007). It remains known on the island and the Canadian Arctic Archipelago from this single collection. Elsewhere in the Canadian Arctic recorded from western mainland sites as far east as Bathurst Inl. (Porsild and Cody 1980, Saarela et al. 2013, Saarela et al. 2017b). Both this sheet (CAN 10089223) and one of *Symphyotrichum pygmaeum* (CAN 10088665) bear the collection number 349a, suggesting they were taken as a single gathering and later split upon realization the collection was mixed.

**NUNAVUT. Wollaston P.**: *Johansen 349a* (CAN).

### *Hulteniella* Tzvelev [1]

**Figure 75.**
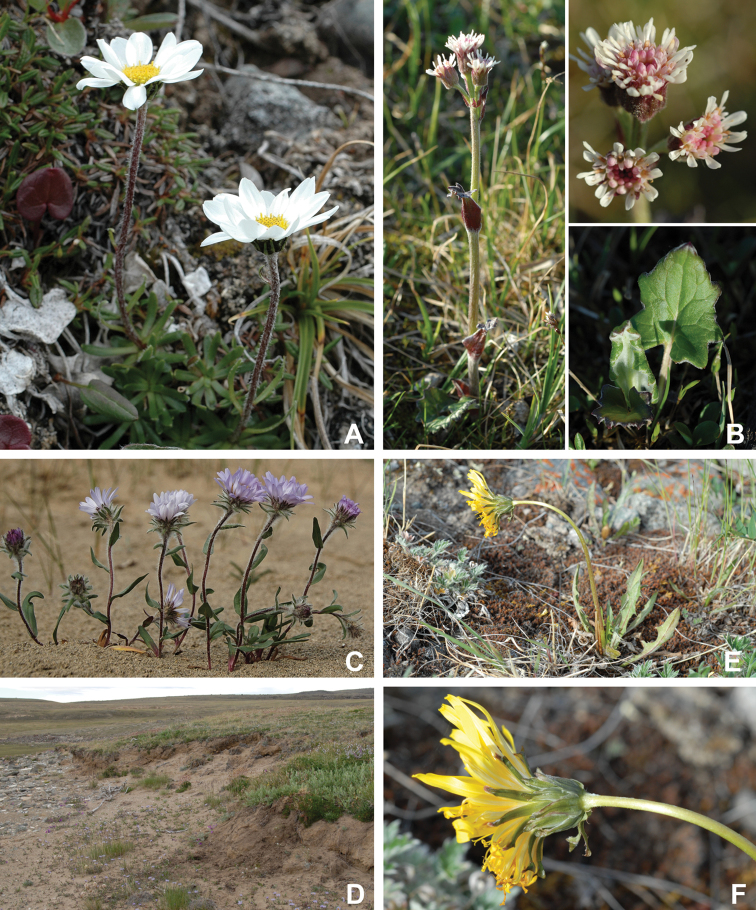
**A***Hulteniella integrifolia* habit, Minto Inlet, NT, 2 July 2010 **B**Petasites frigidus subsp. frigidus habit (left), inflorescence, (upper right) and basal leaf (lower right), *Gillespie et al. 9752***C***Symphyotrichum pygmaeum* habit, *Gillespie et al. 9883***D***Symphyotrichum pygmaeum* habitat, *Gillespie et al. 9883***E***Taraxacum ceratophorum* habit, *Gillespie et al. 9745***F***Taraxacum ceratophorum* inflorescence, *Gillespie et al. 9745.* Photos **A**, **C** by R.D. Bull and **B**, **D–F** by L.J. Gillespie.

***Hulteniella integrifolia*** (Richardson) Tzvelev (*Arctanthemum integrifolium* (Richardson) Tzvelev, *Chrysanthemum integrifolium* Richardson, *Dendrathema integrifolium* (Richardson) Tzvelev, *Leucanthemum integrifolium* (Richardson) DC.), Figs 74C, 75A–Small arctic daisy | Amphi-Beringian–North American (N)

Previously recorded from Byron B., Cambridge Bay, Hadley B., Kuujjua R., Ulukhaktok and Wollaston P. (Macoun and Holm 1921, Porsild 1955, 1957, 1964, Porsild and Cody 1980, Aiken et al. 2007). Thannheiser et al. (2001) additionally recorded it recorded it from Richardson I. Newly recorded from Albert Edward B., Boot Inl., Cape Baring, Diamond Jenness P., Falaise B., Ferguson L., the Greiner L. watershed, the head of Minto Inl., Mt. Bumpus, Mt. Lady Pelly, Namaycush L., “Oldenburg L.”, Oterkvik Pt., an inland site on Prince Albert P., the north side and head of Prince Albert S., Read I., “Trunsky L.”, Walker B. and Washburn L. Elsewhere in the Canadian Arctic recorded throughout the archipelago except for the western Queen Elizabeth Islands and across the mainland (Porsild and Cody 1980, Korol 1992, Cody 1996a, Cody et al. 2003, Aiken et al. 2007, Saarela et al. 2013, Saarela et al. 2017b).

**NORTHWEST TERRITORIES. Boot Inl.**: *Dutilly 18696* (QFA), *Gillespie et al. 9589* (CAN, MT, O). **C. Baring**: *Edlund 408* (CAN). **Kuujjua R.**: *Dutilly 18775* (QFA), *Edlund 631* (CAN), *Gillespie et al. 9743* (CAN, WIN). **Minto Inl. (head)**: *Edlund 167* (CAN), *Gillespie et al. 9483* (CAN). “**Oldenburg L.**”: *Oldenburg 45-1361* (CAN). **Prince Albert P.**: *Oldenburg 54-643* (UBC). **Prince Albert S. (head)**: *Edlund 375* (CAN). **Prince Albert S. (N)**: *Oldenburg 46-2260* (CAN). **Ulukhaktok**: *Edlund 299* (CAN), *Oldenburg 42-21*, *42-23a*, *45-1720* (CAN), *Porsild 17339* (CAN), *Saarela & Bull 1477* (CAN). **Walker B.**: *Oldenburg 45-1512* (CAN). **NUNAVUT. Albert Edward B.**: *Ponomarenko VI*-*269* (CAN). **Byron B.**: *Edlund & Argus 12847* (CAN), *Dushenko 21* (UVIC). **Cambridge Bay**: *Bennett et al. 13-0180* (BABY, od), *Edlund & Argus 12627* (ALA, CAN), *Gillespie et al. 8417* (ALA, CAN, MT, O), *Gould s.n.* (ALA), *Oldenburg 44*-*888* (CAN), *Polunin s.n.* (CAN), *Porsild 21645* (CAN), *Sutton 1047* (CAN, KSTC). **Falaise B.**: *Eriksen et al. 945* (ALA). **Ferguson L. [Tahiryuaq**]: *Bennett et al. 14-0412* (ALA, UBC), *Hainault 2033* (DAO). **Greiner L.**: *Ponomarenko VI-144*, *VI-168*, *VI-228* (CAN). **Hadley B.**: *Edlund 24*, *108* (CAN). **Johansen B.**: *Gillespie et al. 7933* (CAN). **Kugaluk R.**: *Edlund 202* (CAN). **Mt. Bumpus**: *Edlund 228*, *259* (CAN). **Mt. Lady Pelly [Amaaqtuq**]: *Hainault 1825*, *1828* (DAO). **Namaycush L.**: *Edlund & Roncato-Spencer 115* (CAN), *Edlund 30* (CAN). **Oterkvik Pt.**: *Gillespie et al. 7702*, *7814* (CAN). **Read I.**: *Oldenburg 42-503*, *43-960* (CAN). “**Trunsky L.**”: *Bennett et al. 14-0394* (BABY, UBC). **Washburn L.**: *Oldenburg 46-2151* (CAN). **Wollaston P.**: *D. Jenness 317b* (CAN).

### *Petasites* Mill. [1]

***Petasites frigidus*** (L.) Fr. subsp. ***frigidus***, Figs 74D, 75B–Arctic sweet coltsfoot | European (N)–Asian (N)–amphi-Beringian

Previously recorded from Cambridge Bay, the head of Minto Inl. and Ulukhaktok (Aiken et al. 2007). Newly recorded from Boot Inl. and Kuujjua R. Elsewhere in the Canadian Arctic recorded from Banks, Eglinton, Melville and Prince Patrick islands and scattered western mainland sites as far east as the Adelaide Peninsula, Nunavut (Porsild and Cody 1980, Cherniawsky and Bayer 1998, Aiken et al. 2007, Saarela et al. 2013).

**NORTHWEST TERRITORIES. Boot Inl.**: *Gillespie et al. 9600* (CAN, O), *9610* (ALA, CAN, MT, O, UBC, WIN). **Kuujjua R.**: *Gillespie et al. 9752* (ALA, CAN, MT, O). **Minto Inl. (head)**: *Edlund 177*, *179* (CAN). **Ulukhaktok**: *Edlund 791* (CAN). **NUNAVUT. Cambridge Bay**: *Sutton 1278* (CAN, KSTC).

### *Senecio* L. [1]

***Senecio lugens*** Richardson, Fig. 74E–Black-tipped groundsel | American Beringian–Cordilleran

Newly reported from Victoria I. Collected at “Long L.” in 1964. This is the only record of the taxon in the Canadian Arctic Archipelago. Elsewhere in the Canadian Arctic recorded from numerous western mainland sites as far east as the Coppermine R. valley, Nunavut (Porsild and Cody 1980, Saarela et al. 2013, Saarela et al. 2017b).

**NUNAVUT. “Long L.**”: *Lambert s.n.* (CAN) (Suppl. material 12).

### *Symphyotrichum* Nees [1]

***Symphyotrichum pygmaeum*** (Lindl.) Brouillet & Selliah (*Aster pygmaeus* Lindl., A. sibiricus subsp. pygmaeus (Lindl.) Á.Löve & D.Löve, A. sibiricus var. pygmaeus (Lindl.) Cody, *Eurybia pygmaeus* (Lindl.) G.L.Nesom), Figs 74F, 75C, D–Pygmy aster | American Beringian

Previously recorded from Read I. and Wollaston P. (Macoun and Holm 1921, Porsild 1955, Porsild and Cody 1980, Aiken et al. 2007). Thannheiser et al. (2001) additionally recorded it from Johansen B. (conf.). Newly recorded from Boot Inl., Byron B., C. Baring, Clouston B., Falaise B., Greiner L., Kuujjua R., the head of Minto Inl., Murray Pt., Oterkvik Pt., the head of Prince Albert S. and east of the head of Prince Albert S. along the Kagloryuak R. The Greiner L. collections marks the known eastern limit of the taxon in Canada. This species was assessed as May Be at Risk by the Working Group on General Status of NWT Species (2011) but the status was revised to Secure by Working Group on General Status of NWT Species (2016) in light of new information on the species, including reports of newly discovered populations in Tuktut Nogait National Park and vicinity (Saarela et al. 2013) and knowledge of the 2010 records from the Northwest Territories portion of Victoria I. published here. Elsewhere in the Canadian Arctic recorded from Banks I. and western mainland sites as far east as the Hope Bay area northeast of Bathurst Inl. (Porsild and Cody 1980, Cody and Reading 2005, Saarela et al. 2017b).

**NORTHWEST TERRITORIES. Boot Inl.**: *Gillespie et al. 9630* (ALA, CAN, MT). **C. Baring**: *Edlund 415* (CAN). **Kuujjua R.**: *Edlund 632* (CAN), *Gillespie et al. 9883* (ALA, ari, CAN, MT, O), *9968* (ari, CAN, MT, O, UBC, US, WIN). **Minto Inl. (head)**: *Gillespie et al. 10242* (ALA, CAN, O). **Prince Albert S. (head)**: *Edlund 370* (CAN). **NUNAVUT. Byron B.**: *Edlund & Argus 12846* (ALA, CAN). **Falaise B.**: *Eriksen et al. 926* (ALA, CAN). **Greiner L.**: *Ponomarenko VI-340* (CAN). **Wollaston P.**: *D. Jenness 349a* (CAN), *Johansen & D. Jenness 349b* (CAN). **Clouston B.**: *Gillespie et al. 7737* (CAN, MT). **Johansen B.**: *Gillespie et al. 7992* (ALA, ALTA, BABY, CAN, MT, O, UBC, US), *7999* (CAN, MT), *8007* (CAN, MT), *8069* (CAN, MT), *8099* (CAN, MT, O). **Murray Pt.**: *Gillespie et al. 8186* (CAN). **Oterkvik Pt.**: *Gillespie et al. 7548* (CAN, MT, O). **Prince Albert S. (east of head)**: *Edlund & Argus 12813* (ALA, CAN). **Read I.**: *Oldenburg 44*-*1039* (CAN), *Porsild 17214* (CAN), *Ross 23* (ALTA).

### *Taraxacum* L. [5]

**Key to *Taraxacum* [adapted from Brouillet (2006)]:**

**Table:** 

1	All or some phyllary apices notably horned; calyculus bractlets notably horned	***T. ceratophorum***
–	Phyllary apices usually hornless (sometimes callous or horns relatively small); calyculus bractlets usually hornless or horns relatively small	**2**
2	Corollas pink (sometimes ± bronze when fresh) or cream-coloured to white or pink distally, abaxially pinkish-striped; involucres 15–30 mm; calyculi horned	***T. hyparcticum***
–	Corollas pale to dark yellow; involucres (6–)9–14 mm (–15(–20) mm in *T. holmenianum*); calyculi hornless	**3**
3	Leaf blade margins usually entire or toothed to denticulate (sometimes somewhat runcinate and then mostly irregularly and shallowly triangular-lobed); corollas pale yellow, sometimes lemon-coloured; cypselae dark brown, grayish or blackish, muricate 1/2–3/4+	***T. phymatocarpum***
–	Leaf blade margins runcinate, regularly and usually deep triangularly-lobed; corollas yellow to dark yellow; cypselae usually yellowish to brown or reddish brown, sometimes grayish, muricate in distal 1/2 or less	**4**
4	Plants 1–5 cm; leaves fewer than 10, blades (1–)1.5–4 cm; phyllaries 8–12 in two series, corollas yellow, 7.5–8.8 mm; cypsela bodies 2.8–3.5 mm, distal conical part 0.5–0.6 mm; pappi 4.8–5.5 mm	***T. scopulorum***
–	Plants 5–15(–20) cm; leaves 10+, blades (1.5–)2–6(–9) cm; phyllaries ca. 14 in two series; corollas dark yellow, 15–20 mm; cypsela bodies 3.5–4.2 mm, distal conical part 0.9–1.1 mm; pappi 5.5–6.5 mm	***T. holmenianum***


***Taraxacum ceratophorum*** (Ledeb.) DC. (*T. lacerum* Greene), Figs 74G, 75E, F–Horned dandelion | Circumboreal-polar

Previously recorded from Cambridge Bay and Ulukhaktok (Porsild 1955, 1957, 1964, Porsild and Cody 1980, Aiken et al. 2007). Newly recorded from Boot Inl., Clouston B., Ferguson L., Johansen B., Kuujjua R., the head of Minto Inl., Murray Pt., Namaycush L., Oterkvik Pt. and Walker B. Elsewhere in the Canadian Arctic recorded from Axel Heiberg, Baffin, Banks, Eglinton, Ellesmere, King William, Melville and Southampton islands and mainland sites (Porsild and Cody 1980, Aiken et al. 2007, Saarela et al. 2013, Saarela et al. 2017b).

**NORTHWEST TERRITORIES. Boot Inl.**: *Gillespie et al. 9546* (ALA, ari, CAN, MT, O, US, WIN). **Kuujjua R.**: *Gillespie et al. 9745* (ari, CAN, MT), *9877* (ari, CAN, MT), *9928* (ari, CAN, MT). **Minto Inl. (head)**: *Edlund 117*, *147*, *598* (CAN), *Gillespie et al. 10113* (ALA, ari, CAN, MO, MT, US, WIN), *10278* (CAN). **Ulukhaktok**: *Edlund 363*, *757*, *782* (CAN), *Porsild 17344*, *17345* (CAN), *Saarela & Bull 1467* (ALA, ari, CAN, MT, O, UBC). **Walker B.**: *Oldenburg 45-1511* (CAN). **NUNAVUT. Cambridge Bay**: *Edlund & Argus 12860* (CAN), *Gillespie et al. 8488* (ALA, CAN, MT, O), *Ponomarenko VI-312* (CAN), *Stephens 1257* (KSTC). **C. Colborne**: *Edlund & Argus 12728*, *12729* (CAN). **Clouston B.**: *Gillespie et al. 7755* (CAN). **Ferguson L. [Tahiryuaq**]: Myers *& Hainault 2125* (DAO). **Johansen B.**: *Gillespie et al. 7977* (CAN, MT), *8012* (CAN, MT), *8040* (CAN), *8089* (ALA, CAN), *8153* (ALA, ALTA, BABY, CAN, MT, O, UBC). **Murray Pt.**: *Gillespie et al. 8217* (CAN). **Namaycush L.**: *Edlund & Roncato-Spencer 51* (CAN, mixed with *T. holmenianum*), *Edlund 31* (CAN). **Oterkvik Pt.**: *Gillespie et al. 7698* (CAN, MT), *7699* (CAN).

***Taraxacum holmenianum*** Sahlin (*T. pumilum* Dahlst.), Fig. 74H–Holmen’s dandelion | North American (N)

Previously recorded from Cambridge Bay, C. Baring, Mt. Bumpus, Namaycush L., the head of Prince Albert S. and Ulukhaktok (Porsild 1955, 1957, 1964, Porsild and Cody 1980, Aiken et al. 2007). A specimen mapped from C. Colborne in Aiken et al. (2007) has been redetermined as *T. ceratophorum*. Newly recorded from Clouston B., Falaise B., Ferguson L., Hadley B., Johansen B., Kuujjua R., the head of Minto Inl., Read I. and Sinclair Cr. Elsewhere in the Canadian Arctic recorded from Axel Heiberg, Baffin, Banks, Emerald, Ellesmere, King William, Melville, Prince of Wales, Prince Patrick and Somerset islands and a few northern mainland sites (Porsild and Cody 1980, Saarela et al. 2013, Saarela et al. 2017b).

**NORTHWEST TERRITORIES. C. Baring**: *Edlund & Nixon 412* (CAN). **Kuujjua R.**: *Gillespie et al. 9833* (CAN). **Minto Inl. (head)**: *Gillespie et al. 10088* (CAN). **Prince Albert S. (head)**: *Porsild 17447* (CAN). **NUNAVUT. Cambridge Bay**: *Bennett et al. 13-0275* (BABY, CAN, chars, od, UBC), *13-0236* (BABY), *Calder et al. 24208* (DAO), *Ponomarenko VI-074* (CAN). **Clouston B.**: *Gillespie et al. 7753* (ALA, CAN, MT, O). **Falaise B.**: *Eriksen et al. 978* (ALA, O). **Ferguson L. [Tahiryuaq**]: *Hainault & Hunter 1964* (DAO). **Hadley B.**: *Edlund 331* (CAN). **Johansen B.**: *Gillespie et al. 7873a*, *8034*, *8168* (CAN), *7998* (CAN, MT). **Mt. Bumpus**: *Edlund 263*, *278* (CAN). **Namaycush L.**: *Edlund & Roncato-Spencer 51* (CAN, mixed with *T. ceratophorum*). **Read I.**: *Oldenburg 43-1073* (CAN). **Sinclair Cr.**: *Gillespie et al. 8282* (CAN, MT).

**Figure 76.**
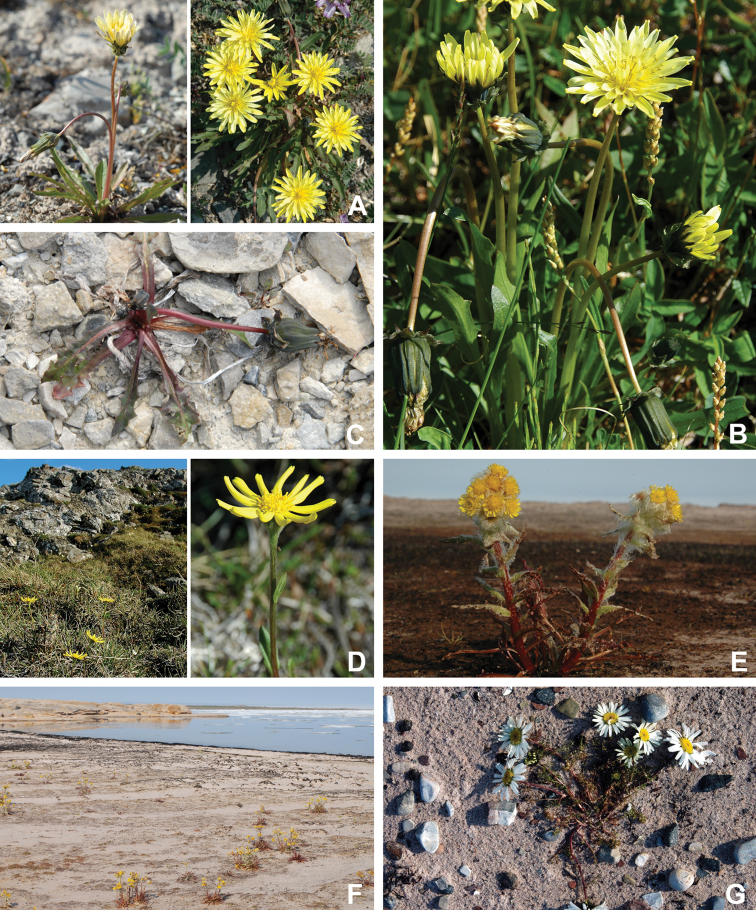
**A***Taraxacum hyparcticum* habit (left), *Gillespie et al. 9746*, inflorescences (right), *Saarela & Bull 1441***B***Taraxacum phymatocarpum* habit, *Gillespie et al. 9698***C***Taraxacum scopulorum* habit, *Gillespie et al. 9835***D***Tephroseris frigida* habitat (left) and habit (right), Minto Inlet, NT, 25 July 2010 **E**Tephroseris palustris subsp. congesta habit **F**Tephroseris palustris subsp. congesta habitat, *Gillespie et al. 8195***G**Tripleurospermum maritimum subsp. phaeocephalum habit, *Gillespie et al. 8215.* Photos **A** left, **C**, **F** by L.J. Gillespie, and **A** right, **B**, **D**, **E**, **G** by R.D. Bull.

***Taraxacum hyparcticum*** Dahlst., Figs 74I, 76A–High Arctic dandelion

Specimens mapped from Cambridge Bay and Ulukhaktok by Aiken et al. (2007) have been re-determined to *T. phymatocarpum*, based primarily on having involucres <15 mm (Brouillet 2006). Similarly, Porsild’s collection nos. 17428 and 17346, treated by him as *T. hyparcticum* (Porsild 1955), are placed under *T. phymatocarpum*. The two species are difficult to distinguish in the herbarium using the key in Brouillet (2006) because the split (lead 10) that divides the species requires knowledge of corolla color (much lighter in *T. hyparcticum* than in *T. phymatocarpum*), which is rarely preserved in dried specimens. Our identifications of these two species are based primarily on horned calyculi and involucre length, described as being 15–30 mm in *T. hyparcticum* vs. 9–14 mm in *T. phymatocarpum* by Brouillet (2006). Newly recorded from Boot Inl., Cambridge Bay, Johansen B., Kuujjua R., the head of Minto Inl., Oterkvik Pt., southeast of the head of Prince Albert S. and Ulukhaktok. Based on the maps in Aiken et al. (2007), both species are fairly widespread across the Canadian Arctic Archipelago. This species is known from fewer collections and sites on Victoria I. than *T. phymatocarpum*.

**NORTHWEST TERRITORIES. Boot Inl.**: *Gillespie et al. 9595* (CAN), *9681* (ALA, ALTA, CAN, MT). **Kuujjua R.**: *Gillespie et al. 9746* (ari, CAN, MT), *9925* (CAN). **Minto Inl. (head)**: *Gillespie et al. 10025* (ALTA, CAN, MT, O), *10185* (CAN, MT), *10188* (ari, CAN, MT), *10219* (CAN, MT). **Ulukhaktok**: *Oldenburg 45-1718*, *45-1719* (CAN), *Saarela & Bull 1440*, *1441* (CAN). **NUNAVUT. Cambridge Bay**: *Bennett et al. 13-0317* (UBC). **Johansen B.**: *Gillespie et al. 7872* (CAN), *7873b* (CAN, MT), *8088* (ALA, CAN, MT, O, UBC). **Oterkvik Pt.**: *Gillespie et al. 7812* (ALA, CAN, MT, O). **Prince Albert S. (head)**: *Edlund 82* (CAN).

***Taraxacum phymatocarpum*** J.Vahl, Figs 74J, 76B–Northern dandelion | Circumpolar

See comments re: taxonomy under previous species. Previously recorded from Cambridge Bay, C. Colborne, an unnamed lake ca. 60 mi. N of Cambridge Bay, the head of Minto Inl., Namaycush L., Read I., Richard Collinson Inl. and Ulukhaktok (Porsild 1955, 1957, 1964, Porsild and Cody 1980, Aiken et al. 2007). Thannheiser et al. (2001) additionally recorded it from Johansen B. Newly recorded from Boot Inl., C. Wollaston, Colville Mts., Greiner L., Kuujjua R., Mt. Bumpus, Oterkvik Pt., an inland site on Prince Albert P., east of the head of Prince Albert S., Sinclair Cr. and Wollaston P. Elsewhere in the Canadian Arctic recorded from also recorded from Baffin, Banks, Devon, Ellesmere, Emerald, Melville and Prince Patrick islands and a few northern mainland sites (Porsild and Cody 1980, Aiken et al. 2007, Saarela et al. 2017b).

**NORTHWEST TERRITORIES. Boot Inl.**: *Gillespie et al. 9592a*, *9698* (CAN). **C. Wollaston**: *Edlund 18* (CAN). **Kuujjua R.**: *Gillespie et al. 9535*, *9847* (CAN). **Minto Inl. (head)**: *Edlund 85*, *86* (CAN), *Gillespie et al. 10021* (CAN, MT), *10066* (CAN), *10076* (ALA, ari, CAN, MT, O, UBC), *10260* (ari, CAN, MT), *Porsild 17428*, *17429* (CAN). **Prince Albert P.**: *Oldenburg 54*-*644* (UBC). **Richard Collinson Inl.**: *Edlund 135* (CAN), *136* (CAN). **Ulukhaktok**: *Edlund 428*, *429*, *722* (CAN), *Gray & Gibbard 35*, *45* (DAO), *Porsild 17346* (CAN). **Wollaston P.**: *Oldenburg 54*-*489* (UBC). **NUNAVUT. Cambridge Bay**: *Gillespie et al. 8489* (CAN, MT), *Porsild 1104* (KSTC), *Stephens 982* (KSTC), *992* (CAN), *Zoltai s.n.* (DAO). **C. Colborne**: *Edlund & Argus 12735* (CAN). **Colville Mts.**: *Gillespie et al. 7770* (CAN). **Greiner L.**: *Ponomarenko VI-196*, *VI-208B* (CAN). **Hadley B.**: *Edlund* 39, *61*, *365* (CAN). **Mt. Bumpus**: *Edlund 214* (CAN). **Namaycush L.**: *Edlund & Argus 12801* (CAN). **Oterkvik Pt.**: *Gillespie et al. 7654* (CAN). **Prince Albert S. (head)**: *Edlund 81*, *152* (CAN), *Edlund & Argus 12817* (CAN). **Read I.**: *Porsild 17215* (CAN). **Sinclair Cr.**: *Gillespie et al. 8340* (CAN). **Unnamed lake ca. 60 mi. N of Cambridge Bay**: *Porsild 17478* (CAN). **Wollaston P.**: *D. Jenness 415* (CAN).

***Taraxacum scopulorum*** (A.Gray) Rydb., Figs 74K, 76C–Alpine dandelion | North American

Newly recorded for Victoria I., where known from Colville Mts., Kuujjua R., “Oldenburg L.” and Oterkvik Pt. Taxonomy follows Brouillet (2006), who reported its range as the “western Canadian Arctic Archipelago and from high-alpine summits in the western Cordilleras”. Taxonomic study of this species, at least with respect to Arctic material, is needed. The general status of this species in the Northwest Territories is Undetermined (Working Group on General Status of NWT Species 2016).

**NORTHWEST TERRITORIES. Kuujjua R.**: *Gillespie et al. 9835* (CAN, MT). “**Oldenburg L.**”: *Oldenburg 45-1353* (CAN). **NUNAVUT. Colville Mts.**: *Gillespie et al. 7767* (CAN). **Oterkvik Pt.**: *Gillespie et al. 7630* (CAN, MT).

### *Tephroseris* (Rchb.) Rchb. [2]

**Key to *Tephroseris* [adapted from Barkley and Murray (2006)):**

**Table:** 

1	Annuals or biennials, rhizomes lacking, stems single, 20–100 cm; leaf blades oblanceolate to linear-oblanceolate or spatulate, 5–15 × 0.5–3(–5) cm; mid-stem leaves prominent, not bractlike; heads (4–)6–20(–40+)	**T. palustris subsp. congesta**
–	Perennials, rhizomatous, stems loosely clustered, (5–)10–20(–30) cm; leaf blades ovate, 1.5–3 × 1–2 cm; midstem leaves bractlike; heads 1(–2)	***T. frigida***

***Tephroseris frigida*** (Richardson) Holub (*Senecio frigidus* (Richardson) Less., *S. atropurpureus* (Ledeb.) B.Fedtsch.), Figs 74L, 76D–Arctic groundsel | Amphi-Beringian–North American (NW)

Previously recorded from C. Baring, the head of Minto Inl., Prince Albert P., Ulukhaktok and Wollaston P. (Macoun and Holm 1921, Porsild 1955, 1957, 1964, Porsild and Cody 1980). Thannheiser et al. (2001) additionally recorded it from Richardson I. We have not seen a voucher corresponding to the Cambridge Bay area mapped in Porsild and Cody (1980), but records from “Long L.” and the Greiner L. area, marking the eastern limit of the taxon in Canada, confirm its presence in the area. Newly recorded from Boot Inl., C. Wollaston, Greiner L., “Long L.”, and the north side of Prince Albert S. Elsewhere in the Canadian Arctic known from Banks I. and mainland sites as far east as Bathurst Inl. (Porsild and Cody 1980, Aiken et al. 2007, Saarela et al. 2013, Saarela et al. 2017b).

**NORTHWEST TERRITORIES. Boot Inl.**: *Dutilly 18693* (CAN, QFA), *Gillespie et al. 9518* (CAN). **C. Baring**: *Edlund 409* (CAN). **C. Wollaston**: *Edlund 29* (CAN). **Kuujjua R.**: *Dutilly 18849* (QFA), *Gillespie et al. 9795* (ALA, CAN). **Minto Inl. (head)**: *Edlund 76* (CAN), *Gillespie et al. 10047* (CAN, MT, O), *9460* (CAN). **Prince Albert S. (N)**: *Oldenburg 46-2263* (CAN). **Prince Albert P.**: *Edlund 416* (CAN), *Oldenburg 54*-*646*, *54*-*647* (UBC). **Richard Collinson Inl.**: *Edlund 137*, *146*, *702* (CAN). **Ulukhaktok**: *Bandringa 314* (CAN), *Dutilly 18619* (CAN, QFA), *Edlund 711* (CAN), *Oldenburg 42-70C*, *45-1709*, *45-1712* (CAN), *Porsild 17343* (CAN), *Ross 326* (ALTA), *Saarela & Bull 1502* (CAN). **Walker B.**: *Oldenburg 45-1514*, *45-1515* (CAN). **NUNAVUT. Greiner L.**: *Ponomarenko VI-098* (CAN). **Johansen B.**: *Gillespie et al. 7938* (CAN, MT, O), *7939* (ALA, CAN, MT, O). “**Long L.**”: *Lambert s.n.* (CAN, 2 sheets). **Wollaston P.**: *D. Jenness 329a* (CAN).

**Figure 77.**
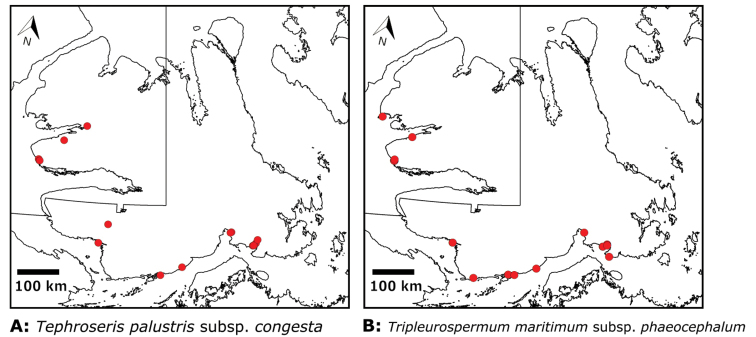
Species distribution maps. Asteraceae: **A**Tephroseris palustris subsp. congesta**B**Tripleurospermum maritimum subsp. phaeocephalum.

***Tephroseris palustris*** subsp. ***congesta*** (R.Br.) Holub (*Senecio congestus* R.Br.), Figs 77A, 76E, F–Marsh groundsel | European (C-S) & European (NE)–Asian (N/C)–amphi-Beringian–North American

Previously recorded from Cambridge Bay, the head of Minto Inl., Read I. (Porsild obs., conf.), Ulukhaktok and Wollaston P. (Porsild 1955, 1957, 1964, Porsild and Cody 1980, Aiken et al. 2007). Thannheiser et al. (2001) additionally recorded it from Surrey L. and Wellington B. Newly recorded from Kuujjua R., Ferguson L., Murray Pt. and Sinclair Cr. Elsewhere in the Canadian Arctic recorded from across the Canadian Arctic mainland and throughout the Canadian Arctic Archipelago except for the northern and eastern Queen Elizabeth Islands (Porsild and Cody 1980, Korol 1992, Cody and Reading 2005, Aiken et al. 2007, Saarela et al. 2017b).

**NORTHWEST TERRITORIES. Kuujjua R.**: *Gillespie et al. 9904* (CAN). **Minto Inl. (head)**: *Porsild 17426* (CAN). **Ulukhaktok**: *Edlund 496* (CAN), *809* (CAN), *Oldenburg 45-1708* (CAN), *Saarela & Bull 1448* (CAN, MT, O). **NUNAVUT. Cambridge Bay**: *Dutilly 28175* (QFA), *Bennett et al. 13-0247* (ALA, chars, UBC), *Ducruc s.n.* (QFA), *Edlund & Argus 12643* (CAN), *Gillespie et al. 8401* (CAN, MT, O), *Parker & Jonsdottir 9091* (ALA), *Polunin s.n.* (CAN), *Porsild 972* (KSTC), *Saarela & Teeter 5288*, *5296* (CAN), *Sweatman & Smith 26* (DAO). **Ferguson L. [Tahiryuaq**]: *Hainault* 1967 (DAO), *Jones 29* (DAO). **Greiner L.**: *Ponomarenko VI-330* (CAN). **Murray Pt.**: *Gillespie et al. 8195* (ALA, ALTA, BABY, CAN, MT, O, UBC, US). **Read I.**: *Oldenburg 43-1070*, *43-955* (CAN). **Sinclair Cr.**: *Gillespie et al. 8335* (CAN, MT). **Wollaston P.**: *D. Jenness 414* (CAN).

### *Tripleurospermum* Sch. Bip. [1]

***Tripleurospermum maritimum*** subsp. ***phaeocephalum*** (Rupr.) Hämet-Ahti (*Matricaria ambigua* (Ledeb.) Krylov, M. maritima subsp. phaeocephala (Rupr.) Rauschert), Figs 77B, 76G–Arctic chamomile | Circumpolar

Previously recorded from Berkeley Pt., Cambridge Bay, the head of Minto Inl. (Porsild obs.), the head of Prince Albert S. (Porsild obs.), Read I. (Porsild obs., conf.) and Ulukhaktok (Porsild 1955, 1957, 1964, Porsild and Cody 1980, Aiken et al. 2007). Thannheiser et al. (2001) additionally recorded it from Johansen B. (conf.) and Wellington B. We have not seen a supporting specimen for the inland site north of Cambridge Bay mapped in Aiken et al. (2007), which is likely an error given this is a seashore species. Elsewhere in the Canadian Arctic recorded from Baffin, Banks, King William and Southampton islands and mainland sites (Porsild and Cody 1980, Cody et al. 1989, Cody and Reading 2005, Aiken et al. 2007, Saarela et al. 2017b).

**NORTHWEST TERRITORIES. Berkeley Pt.**: *Stretton 91* (DAO). **Kuujjua R.**: *Gillespie et al. 9918* (ALA, ari, CAN, MO, MT). **Ulukhaktok**: *Edlund 314* (CAN), *Gray & Gibbard 21* (DAO), *Oldenburg 45-1717* (CAN), *Saarela & Bull 1426* (CAN, O), *Salokangas 24* (CAN, UBC). **NUNAVUT. Cambridge Bay**: *Bennett 13-0267* (BABY, chars), *13-0262* (CAN, od), *Gillespie et al. 8462* (CAN), *Saarela & Teeter 5283* (CAN), *Stephens* (KSTC), *Stephens 1263* (CAN). **C. Colborne**: *Edlund & Argus 12736* (CAN). **Ferguson L. [Tahiryuaq**]: *Hainault 1968* (DAO). **Johansen B.**: *Gillespie et al. 8004* (CAN, MT, O). **Murray Pt.**: *Gillespie et al. 8215* (ALA, CAN, MT, O). **Oterkvik Pt.**: *Gillespie et al. 7627* (CAN, MT, O, UBC). **Read I.**: *Oldenburg 42-478*, *43-1063*, *43-959* (CAN). **Sinclair Cr.**: *Gillespie et al. 8258* (CAN, MT, O).

### Excluded taxa

***Eremogone capillaris*** (Poir.) Fenzl var. ***capillaris*** (*Arenaria capillaris* Poir.)–Aiken et al. (2007) noted occurrence of this taxon on Victoria I. based on “literature record”, but did not cite the literature source or map the record on the island. We are not aware of any material of this taxon from the study area.

***Carex holostoma*** Drejer–Aiken et al. (2007) mapped this species on Victoria I. based on a collection from Surrey L. The specimen, *Edlund & Argus 12805* (CAN), was previously misidentified as *Carex norvegica* Retz. and *C. holostoma*, and has been re-identified as Carex bigelowii subsp. lugens.

***Carex lachenalii*** Schkuhr–A record mapped by Aiken et al. (2007) from Ulukhaktok (*Edlund 338*) has been redetermined as C. simpliciuscula subsp. subholarctica, and a collection from Cambridge Bay (*Porsild 17464*) reported by Porsild (1955) has been redetermined as *C. glareosa*. Given these new determinations, this species is now known in the Canadian Arctic Archipelago only from Baffin, Coats and Southampton islands (Aiken et al. 2007). It is, however, known from the mainland adjacent to Victoria I. (Saarela et al. 2013, Saarela et al. 2017b) and may be expected on the island.

***Draba borealis*** DC.–Mapped in Aiken et al. (2007) from Ferguson Lake, based on specimens (CAN 561159, CAN 561455) annotated by G.A. Mulligan. We were unable to locate the two specimens at CAN, and exclude the species from the flora pending verification.

***Hippuris vulgaris*** L.–Previous records of this species from Victoria I. have been redetermined as *H. lanceloata*; see comments under that taxon.

***Poa alpina*** L.–Mapped from southeastern Victoria I. in Soreng (2007). We have not seen a supporting voucher.

***Salix cordifolia*** var. ***callicarpaea*** (Trautv.) Fernald (=Salix glauca var. cordifolia (Pursh) Dorn)–Reported from the head of Minto Inl. by Thannheiser et al. (2001); we have not seen a supporting voucher.

***Salix fuscescens*** Andersson–See comments under *S. planifolia*.

***Solidago multiradiata*** Aiton–Mapped in Aiken et al. (2007) from the Walker Bay area, based on the map in Porsild and Cody (1980). We have not seen a supporting voucher.

## Supplementary materials

Supplementary material 1 List of 19th Century collections gathered on Victoria Island by Andersson (Cambridge Bay, Minto Inlet, Cambridge Bay & Minto Inlet, and Prince Albert Sound), Miertsching (Prince Albert Sound) and Rae (“south coast”), summarized from Simmons (1913) Data type: species list https://binary.pensoft.net/file/387097This dataset is made available under the Open Database License (http://opendatacommons.org/licenses/odbl/1.0/). The Open Database License (ODbL) is a license agreement intended to allow users to freely share, modify, and use this Dataset while maintaining this same freedom for others, provided that the original source and author(s) are credited.Jeffery M. Saarela, Paul C. Sokoloff, Lynn J. Gillespie, Roger D. Bull, Bruce A. Bennett, Serguei Ponomarenko

Supplementary material 2 Dataset of voucher specimens documenting vascular plant diversity on Victoria Island, Nunavut and Northwest Territories, Canada Data type: species list https://binary.pensoft.net/file/387098This dataset is made available under the Open Database License (http://opendatacommons.org/licenses/odbl/1.0/). The Open Database License (ODbL) is a license agreement intended to allow users to freely share, modify, and use this Dataset while maintaining this same freedom for others, provided that the original source and author(s) are credited.Jeffery M. Saarela, Paul C. Sokoloff, Lynn J. Gillespie, Roger D. Bull, Bruce A. Bennett, Serguei Ponomarenko

Supplementary material 3 Vascular plants recorded from Victoria Island Data type: species list Explanation note: Taxa are listed by major clade and then alphabetically by family. The dataset records whether or not each taxon is recorded in Northwest Territories and Nunavut, eight sites on the island that have explored comprehensively (NWT: Ulukhaktok, Boot Inlet, Kuujjua River, the head of Minto Inlet; NU: Oterkvik Point, Johansen Bay, Sinclair Creek, Cambridge Bay) and Ovayok Territorial Park, Nunavut. The table also records present or absence of each taxon in Circumpolar Arctic Vegetation Map bioclimate subzones C and D, northern and southern areas of subzone C, areas of subzone D east and west of Wellington Bay, ecological regions of the Northwest Territories portion of Victoria Island, taxon rank, and the total number of collections for each taxon included in the dataset. https://binary.pensoft.net/file/387099This dataset is made available under the Open Database License (http://opendatacommons.org/licenses/odbl/1.0/). The Open Database License (ODbL) is a license agreement intended to allow users to freely share, modify, and use this Dataset while maintaining this same freedom for others, provided that the original source and author(s) are credited.Jeffery M. Saarela, Paul C. Sokoloff, Lynn J. Gillespie, Roger D. Bull, Bruce A. Bennett, Serguei Ponomarenko

Supplementary material 4 Image of collection of *Bromus pumpellianus* from “Long Lake”, Victoria Island, Nunavut (*Lambert s.n.*, CAN-529365/ CAN 10009718) Data type: image Explanation note: Photo credit: Jasmine Lai, Canadian Museum of Nature. https://binary.pensoft.net/file/387100This dataset is made available under the Open Database License (http://opendatacommons.org/licenses/odbl/1.0/). The Open Database License (ODbL) is a license agreement intended to allow users to freely share, modify, and use this Dataset while maintaining this same freedom for others, provided that the original source and author(s) are credited.Jeffery M. Saarela, Paul C. Sokoloff, Lynn J. Gillespie, Roger D. Bull, Bruce A. Bennett, Serguei Ponomarenko

Supplementary material 5 Image of collection of *Pulsatilla nuttalliana* from “Long Lake”, Victoria Island, Nunavut (*Lambert s.n.*, CAN- 529362/ CAN 10048735) Data type: image Explanation note: Photo credit: Wilson Wang, Canadian Museum of Nature. https://binary.pensoft.net/file/387101This dataset is made available under the Open Database License (http://opendatacommons.org/licenses/odbl/1.0/). The Open Database License (ODbL) is a license agreement intended to allow users to freely share, modify, and use this Dataset while maintaining this same freedom for others, provided that the original source and author(s) are credited.Jeffery M. Saarela, Paul C. Sokoloff, Lynn J. Gillespie, Roger D. Bull, Bruce A. Bennett, Serguei Ponomarenko

Supplementary material 6 Images of three collections of *Lupinus arcticus* from “Long Lake”, Victoria Island, Nunavut (*Lambert s.n.*, CAN-529350/ CAN 10071815, CAN-529326/ CAN 10071817, CAN-529355/ CAN 10071816) Data type: image Explanation note: Photo credit: Annie Dicaire, Canadian Museum of Nature. https://binary.pensoft.net/file/387102This dataset is made available under the Open Database License (http://opendatacommons.org/licenses/odbl/1.0/). The Open Database License (ODbL) is a license agreement intended to allow users to freely share, modify, and use this Dataset while maintaining this same freedom for others, provided that the original source and author(s) are credited.Jeffery M. Saarela, Paul C. Sokoloff, Lynn J. Gillespie, Roger D. Bull, Bruce A. Bennett, Serguei Ponomarenko

Supplementary material 7 Image of collection of *Rubus chamaemorus* from “Long Lake”, Victoria Island, Nunavut (*Lambert s.n.*, CAN-529339/ CAN 10070345) Data type: image Explanation note: Photo credit: Wilson Wang, Canadian Museum of Nature. https://binary.pensoft.net/file/387103This dataset is made available under the Open Database License (http://opendatacommons.org/licenses/odbl/1.0/). The Open Database License (ODbL) is a license agreement intended to allow users to freely share, modify, and use this Dataset while maintaining this same freedom for others, provided that the original source and author(s) are credited.Jeffery M. Saarela, Paul C. Sokoloff, Lynn J. Gillespie, Roger D. Bull, Bruce A. Bennett, Serguei Ponomarenko

Supplementary material 8 Photographs of tree-sized stand of *Salix alaxensis* at the head of Minto Inlet in 1982 taken by Sylvia A. Edlund Data type: image Explanation note: Source: Canadian Museum of Nature Archival Fond CMNAC/2015-006. https://binary.pensoft.net/file/387104This dataset is made available under the Open Database License (http://opendatacommons.org/licenses/odbl/1.0/). The Open Database License (ODbL) is a license agreement intended to allow users to freely share, modify, and use this Dataset while maintaining this same freedom for others, provided that the original source and author(s) are credited.Jeffery M. Saarela, Paul C. Sokoloff, Lynn J. Gillespie, Roger D. Bull, Bruce A. Bennett, Serguei Ponomarenko

Supplementary material 9 Image of collection of Salix ovalifolia var. ovalifolia from the vicinity of Ferguson Lake [Tahiryuak], Victoria Island, Nunavut (Hainault 2022, DAO-840911/ 01-01000684637) Data type: image Explanation note: Photo: National Collection of Vascular Plants, Agriculture and Agri-Food Canada. https://binary.pensoft.net/file/387105This dataset is made available under the Open Database License (http://opendatacommons.org/licenses/odbl/1.0/). The Open Database License (ODbL) is a license agreement intended to allow users to freely share, modify, and use this Dataset while maintaining this same freedom for others, provided that the original source and author(s) are credited.Jeffery M. Saarela, Paul C. Sokoloff, Lynn J. Gillespie, Roger D. Bull, Bruce A. Bennett, Serguei Ponomarenko

Supplementary material 10 Image of collection of *Salix planifolia* from “Long Lake”, Victoria Island, Nunavut (*Lambert s.n.*, CAN-529349/ CAN 10024243) Data type: image Explanation note: Photo credit: Galyna Vakulenko, Canadian Museum of Nature. https://binary.pensoft.net/file/387106This dataset is made available under the Open Database License (http://opendatacommons.org/licenses/odbl/1.0/). The Open Database License (ODbL) is a license agreement intended to allow users to freely share, modify, and use this Dataset while maintaining this same freedom for others, provided that the original source and author(s) are credited.Jeffery M. Saarela, Paul C. Sokoloff, Lynn J. Gillespie, Roger D. Bull, Bruce A. Bennett, Serguei Ponomarenko

Supplementary material 11 Image of collection of *Erysimum coarctatum* from “Long Lake”, Victoria Island, Nunavut (*Lambert s.n.*, CAN-529364/ CAN 10057248) Data type: image Explanation note: Photo credit: Wilson Wang, Canadian Museum of Nature. https://binary.pensoft.net/file/387107This dataset is made available under the Open Database License (http://opendatacommons.org/licenses/odbl/1.0/). The Open Database License (ODbL) is a license agreement intended to allow users to freely share, modify, and use this Dataset while maintaining this same freedom for others, provided that the original source and author(s) are credited.Jeffery M. Saarela, Paul C. Sokoloff, Lynn J. Gillespie, Roger D. Bull, Bruce A. Bennett, Serguei Ponomarenko

Supplementary material 12 Image of collection of *Senecio lugens* from “Long Lake”, Victoria Island, Nunavut (*Lambert s.n.*, CAN-529359/ CAN 10089218) Data type: image Explanation note: Photo credit: Erin Howard, Canadian Museum of Nature. https://binary.pensoft.net/file/387108This dataset is made available under the Open Database License (http://opendatacommons.org/licenses/odbl/1.0/). The Open Database License (ODbL) is a license agreement intended to allow users to freely share, modify, and use this Dataset while maintaining this same freedom for others, provided that the original source and author(s) are credited.Jeffery M. Saarela, Paul C. Sokoloff, Lynn J. Gillespie, Roger D. Bull, Bruce A. Bennett, Serguei Ponomarenko
