# Supplementary material for: Vascular plants of Victoria Island (Northwest Territories and Nunavut, Canada): a specimen-based study of an Arctic flora
Source: PhytoKeys. 2020 Mar 6;141:1–330. doi: 10.3897/phytokeys.141.48810 (PMC7070024; doi:10.3897/phytokeys.141.48810)
Supplement: Supplementary material 1 [file phytokeys-141-001-s001.pdf]

Supplementary File 1.

List of 19th Century collections gathered on Victoria Island by Andersson (Cambridge Bay, Minto Inlet, Cambridge Bay & Minto Inlet, and Prince Albert Sounds), Miertsching (Prince Albert Sound) and along the south coast by Rae, summarized from Simmons (1913).

Currently accepted names, where applicable, are listed in square brackets. We have not reviewed any of the specimens to confirm identifications. In cases where the

Minto Inlet | collected by Andersson | housed at K

Cystopteris arvense [Cystopteris fragilis
Equisetum arvense
Hierochloe pauciflora [*Anthoxanthum arcticum*]
Arctagrostis latifolia
Calamagrostis purpurascens
Aira cespitosa [Deschampsia cespitosa; collections are likely *Deschampsia brevifolia*]
Dupontia fisheri
Atropa angustata [*Puccinellia angustata*]
Festuca ovina [*Festuca brachyphylla* complex]
Agropyron violaceum [*Elymus violaceus*, probably *E. alaskanus*]
Elymus mollis [*Leymus mollis*]
Eriophorum vaginatum
Eriophorum scheuchzeri
Cobresia bellardi [*Carex myosuroides*]
Carex scirpoidea
Carex incurve [*C. maritima*]
Carex aquatilis var. *stans*
Carex vaginata
Carex membranopacta [*Carex membranacea*]
Salix richardsonii
Salix glauca
Anemone richardsonii [*Anemonastrum richardsonii*]
Lesquerella arctica [*Physaria arctica*]
Braya purpurascens
Hesperis pallasii [*Erysimum pallasii*]
Chrysosplenium alternifolium [*Chrysosplenium tetrandrum*]
Saxifraga tricuspidata

Saxifraga aizoides
Potentilla rubricaulis
Hedysarum alpinum
Linum perenne
Epilobium anagallidifolium
Cassiope tetragona
Arctostaphylos alpina [*Arctous alpina*]
Vaccinium uliginosum
Androsace chamaejasme
Statice armeria L. [*Armeria scabra*]
Pedicularis lanata
Campanula uniflora
Erigeron uniflorus
Antennaria alpina
Artemisia borealis
Petasites frigida
Arnica alpina
Senecio frigidus [*Tephroseris frigida*]

Cambridge Bay and Minto Inlet | collected by Andersson | housed at K

Alopecurus alpinus [*Alopecurus borealis*]
Carex misandra [*Carex fuliginosa* subsp. *misandra*]
Salix arctica
Salix reticulata
Polygonum vivipara [*Bistorta vivipara*]
Stellaria longipes
*Cerastium alpinum [*probably *C. arcticum* or *C. beeringianum*]
Cerastium fischerianum
Honckenya peploides
Melandrium apetalum [*Silene uralensis*]
Caltha palustris
Ranunculus affinis [*Ranunculus arcticus*]
Papaver radicatum
Cochlearia officinalis
Eutrema edwardsii
Draba hirta [Draba glabella]
Parrya arctica
Saxifraga oppositifolia
Saxifraga hirculus
Saxifraga cernua
Dryas integrifolia
Potentilla nivea
Astragalus alpinus [*A. australis*]
Astragalus aboriginorum [*A. richardsonii*]]
Oxytropis campestris

Oxytropis arctica
Oxytropis arctobia
Hedysarum mackenzii
Chamaenerion latifolium
Androsace septentrionalis
Statice maritima [*Armeria scabra*]
Castilleja pallida
Pedicularis sudetica [*Pedicularis arctoeuropaea or Pedicularis albolabiata*]
Pedicularis arctica [*Pedicularis langsdorfii* subsp. *arctica*]
Pedicularis capitata
Chrysanthemum integrifolium [*Hulteniella integrifolium*]
Senecio palustris [*Tephroseris palustris*]
Taraxacum hyparcticum

Prince Albert Sound | collected by Andersson | housed at K

Salix myrsinites
Silene acaulis

Cambridge Bay | collected by Andersson | housed at K

Carex ustulata Wahlenb. [*Carex atrofusca*]
Oxyria digyna
Melandrium affine [*Silene uralensis*]
Cardamine hyperborea O.E.Schulz. [*Cardamine digitata* Richardson]
Draba fladnizensis
Saxifraga nivalis [*Micranthes nivalis*]
Saxifraga groenlandica L. [*Saxifraga cespitosa*]
Hippuris vulgaris

Collinson: Walker Bay:

Oxyria digyna

Mount Adventure -nw passage p. 125 | McClure | housed at K

Salix arctica

Prince Albert Sound | collected by Miertsching | housed at K

Draba hirta [*Draba glabella*]
Saxifraga oppositifolia
Potentilla rubricaulis
Astragalus aboriginorum [*Astragalus richardsonii*]

Arctostaphylos alpina [*Arctous alpina*]

"South coast" Victoria Island | Collected by John Rae | housed at K

Hierochloe alpina [*Anthoxanthum arcticum*]
Arctagrostis latifolia
Poa cenisia [*P. arctica*]
Dupontia fisheri
Festuca ovina [*F. brachyphylla complex*]
Carex aquatilis var. *stans*
Carex scirpoidea
Salix richardsonii
Salix glauca
Oxyria digyna
Caltha palustris
Anemone richardsonii [Anemonastrum richardsonii]
Ranunculus pygmaeus
Papaver radicatum
Cochlearia officinalis [*Cochlearia groenlandica*]
Cardamine hyperborea [*Cardamine digitata*]
Draba hirta [*Draba glabella*]
Draba fladnizensis
Arabis arenicola
Hesperis pallasii [*Erysimum pallasii*]
Saxifraga hirculus
Dryas integrifolia
Oxytropis campestris
Oxytropis arctica
Chamaenerion latifolium
Arctostaphylos alpina [*Arctous alpina*]
Vaccinium uliginosum
Androsace chamaejasme
Mertensia maritima
Pedicularis arctica [*Pedicularis langsdorfii* subsp. *arctica*]
Pedicularis capitata
Chrysanthemum integrifolium [*Hulteniella integrifolia*]
Taraxacum hyparcticum

Reference:
Simmons HG. 1913. A survey of the phytogeography of the Arctic American Archipelago. Lunds Universitets Arsskrift, Nyfoljd, Afdelning 1 9:1–183.
