# Supplementary material for: Vascular plants of Victoria Island (Northwest Territories and Nunavut, Canada): a specimen-based study of an Arctic flora
Source: PhytoKeys. 2020 Mar 6;141:1–330. doi: 10.3897/phytokeys.141.48810 (PMC7070024; doi:10.3897/phytokeys.141.48810)
Supplement: Supplementary material 4 [file phytokeys-141-001-s004.pdf]

canadian museum of nature
nature
musée canadien de la nature

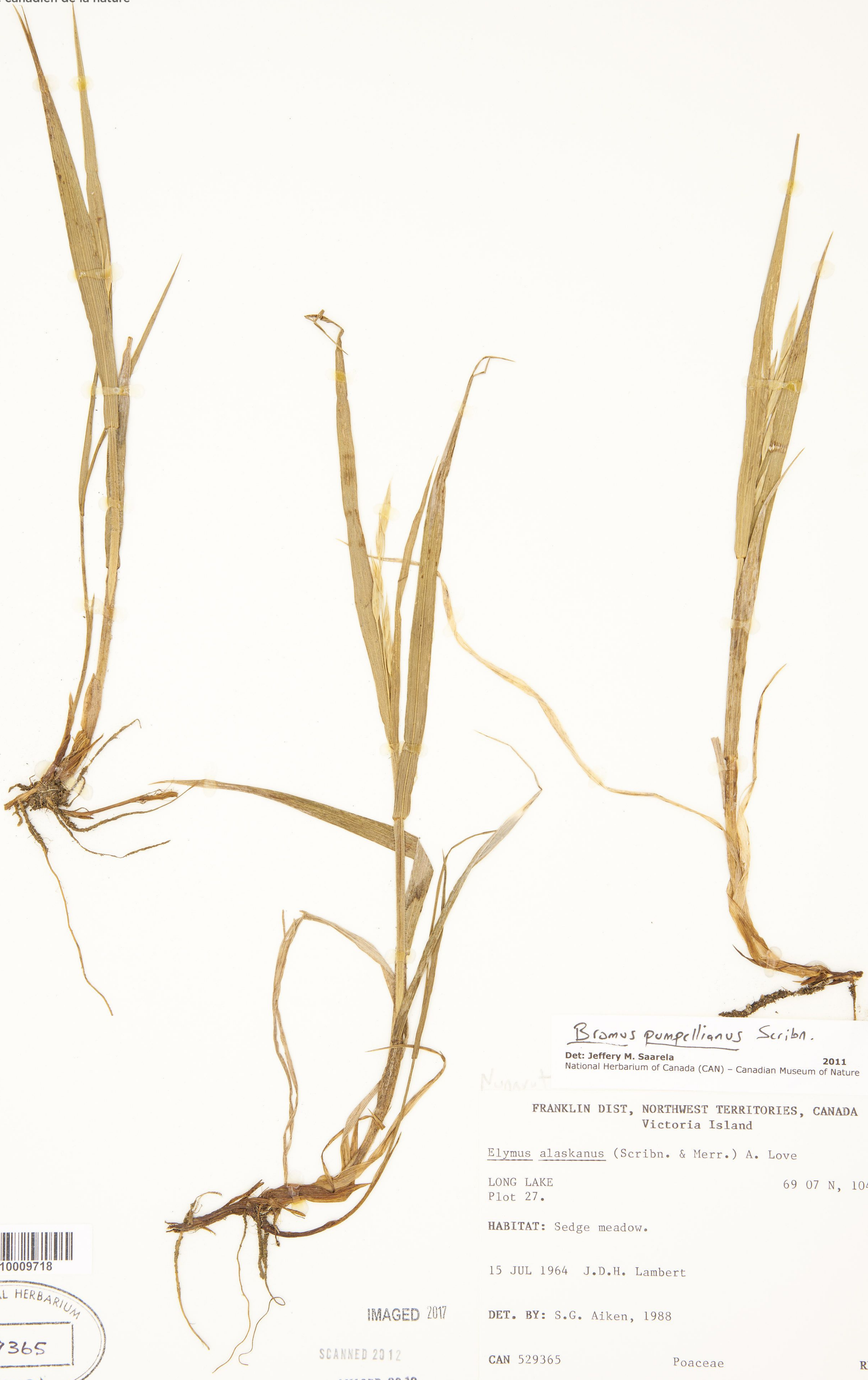

Bromus pumpellianus Scribn.

Det: Jeffery M. Saarela 2011
National Herbarium of Canada (CAN) – Canadian Museum of Nature

Nunavut

FRANKLIN DIST, NORTHWEST TERRITORIES, CANADA
Victoria Island

Elymus alaskanus (Scribn. & Merr.) A. Love

LONG LAKE
Plot 27.
69 07 N, 104 34 W

HABITAT: Sedge meadow.

15 JUL 1964 J.D.H. Lambert

DET. BY: S.G. Aiken, 1988

CAN 529365 Poaceae REPS: 1

National Herbarium of Canada

CAN 10009718

NATIONAL HERBARIUM
529365
CANADA

IMAGED 2017

SCANNED 2012

IMAGED 2018 MM

canadian museum of nature
nature
musée canadien de la nature
