# Supplementary material for: Vascular plants of Victoria Island (Northwest Territories and Nunavut, Canada): a specimen-based study of an Arctic flora
Source: PhytoKeys. 2020 Mar 6;141:1–330. doi: 10.3897/phytokeys.141.48810 (PMC7070024; doi:10.3897/phytokeys.141.48810)
Supplement: Supplementary material 5 [file phytokeys-141-001-s005.pdf]

canadian museum of nature
nature
musée canadien de la nature

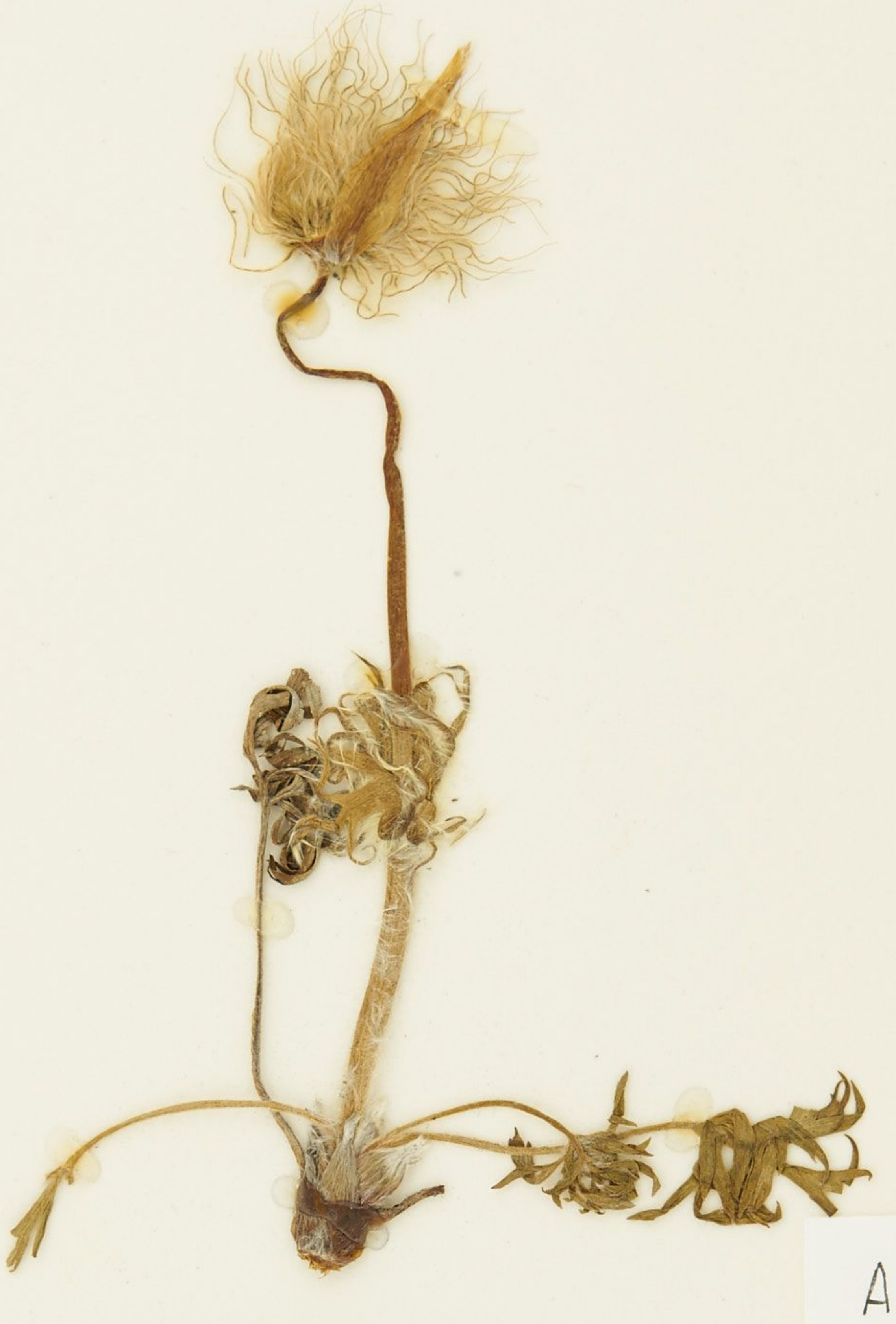

Anemone patens subsp. multifida (Pritz.) L.

Paul C. Sokoloff
National Herbarium of Canada (CAN) 2017

Pulsatilla patens (L.) Mill. subsp. multifida (Pritz.) Zamelis
Laurie L. Consaul
National Herbarium of Canada (CAN) 2011

CAN 529362

Recorded CAA2004

Annot.
Anemone patens L. var. multifida Printzel
Peter J. Scott Sept. 1998
Agnes Marion Ayre Herbarium, Memorial Univ. of Nfld.

NU

FRANKLIN DIST, NORTHWEST TERRITORIES, CANADA
Victoria Island

Pulsatilla patens (L.) P. Mill. ssp. patens

LONG LAKE 69 07 N, 104 34 W
Plot 26.

HABITAT: Sedge meadow.

NOTES: (= Anemone patens).

15 JUL 1964 J.D.H. Lambert

DET. BY: A.W. Dugal, 1988

CAN 529362 Ranunculaceae REPS: 1

National Herbarium of Canada

SCANNED 2011

CAN 10048735

NATIONAL HERBARIUM
529362
CANADA

CAN
IMAGED
2018

DNA Barcode Voucher Specimen
Barcode of Life Data Systems (BOLD) Sample ID
Lambert_sn_CAN529362
Sequenced from tissue removed from sheet (see arrow)
www.boldsystems.org 2010
Canadian Museum of Nature

canadian museum of nature
nature
musée canadien de la nature
