# Supplementary material for: Vascular plants of Victoria Island (Northwest Territories and Nunavut, Canada): a specimen-based study of an Arctic flora
Source: PhytoKeys. 2020 Mar 6;141:1–330. doi: 10.3897/phytokeys.141.48810 (PMC7070024; doi:10.3897/phytokeys.141.48810)
Supplement: Supplementary material 6 [file phytokeys-141-001-s006.pdf]

canadian museum of nature
nature
musée canadien de la nature

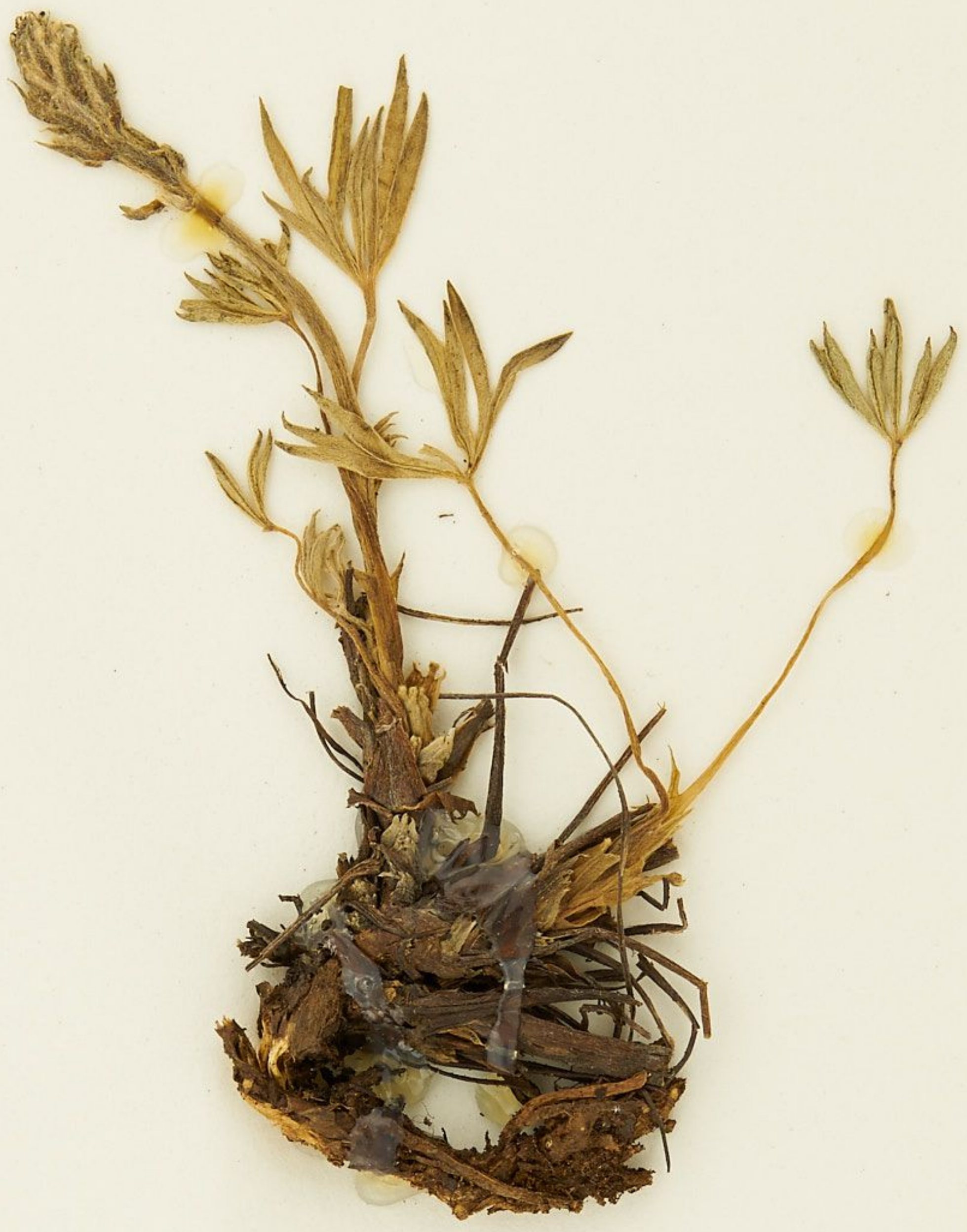

NU

DATA RECORDED
CAN 1999

RARE AND ENDANGERED PLANTS PROJECT

Lupinus arcticus S.Wats.

Det. J.M.Gillett, 1987

National Herbarium of Canada

FRANKLIN DIST, NORTHWEST TERRITORIES, CANADA
Victoria Island

Lupinus arcticus S. Wats.

LONG LAKE 69 07 N, 104 34 W
Plot 25.

HABITAT: Sedge meadow.

15 JUL 1964 J.D.H. Lambert

DET. BY: J.M. Gillett, 1988

CAN 529350 Fabaceae **REPS:** 1

National Herbarium of Canada

NATIONAL HERBARIUM
529350
CANADA

CAN
IMAGED
2018

SCANNED 2013

CAN 10071815

canadian museum of nature
nature
musée canadien de la nature

canadian museum of nature
nature
musée canadien de la nature

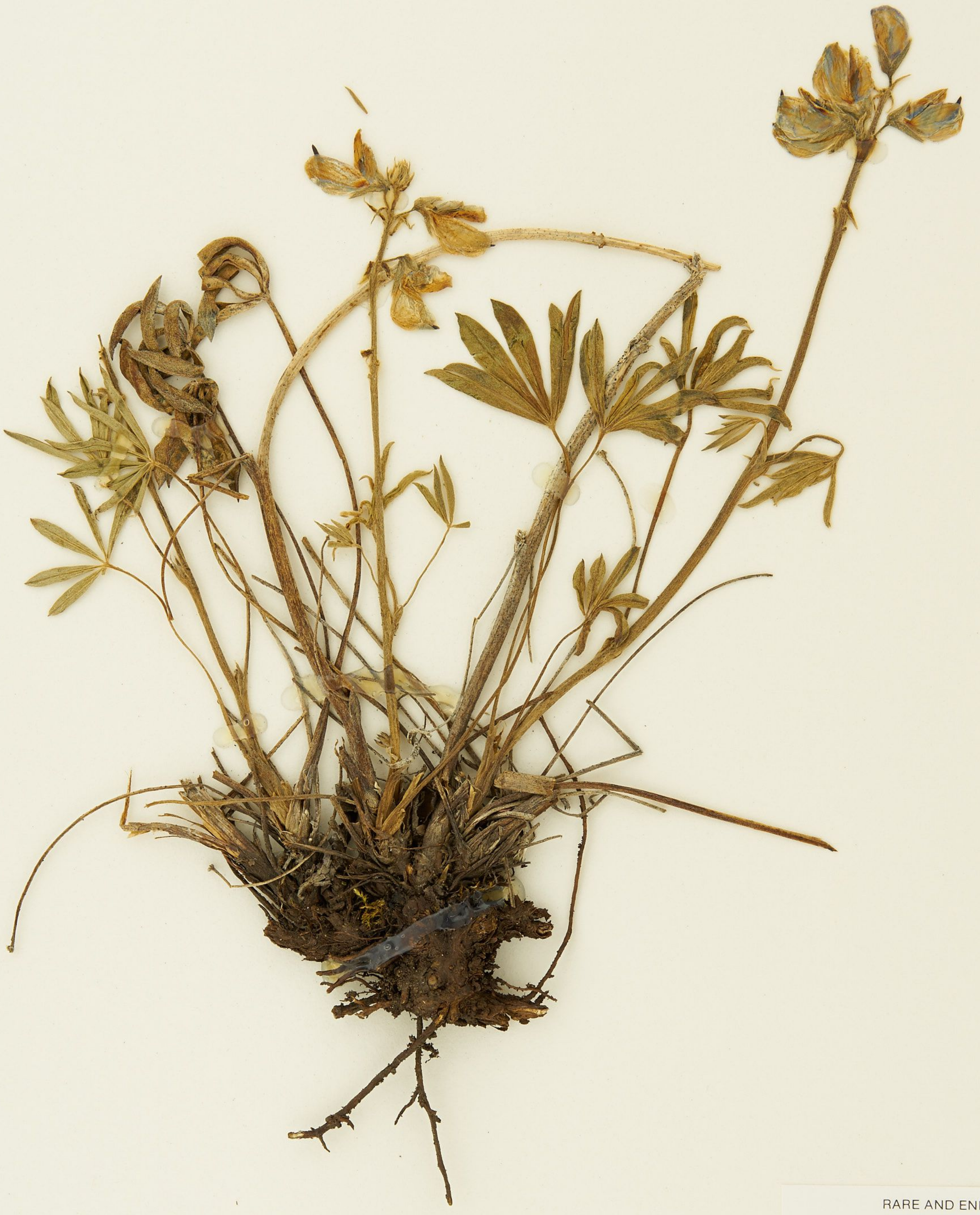

DATA RECORDED
CAN 1999

RARE AND ENDANGERED PLANTS PROJECT

Lupinus arcticus S. Wats.

Det. J.M.Gillett, 1987

National Herbarium of Canada

NU

FRANKLIN DIST, NORTHWEST TERRITORIES, CANADA
Victoria Island

Lupinus arcticus S. Wats.

LONG LAKE
Plot 21.

69 07 N, 104 34 W

13 JUL 1964 J.D.H. Lambert

DET. BY: J.M. Gillett, 1988

CAN 529326 Fabaceae **REPS:** 1

National Herbarium of Canada

NATIONAL HERBARIUM
529326
CANADA

CAN 10071817

CAN
IMAGED
2018

SCANNED 2013

canadian museum of nature
nature
musée canadien de la nature

canadian museum of nature
nature
musée canadien de la nature

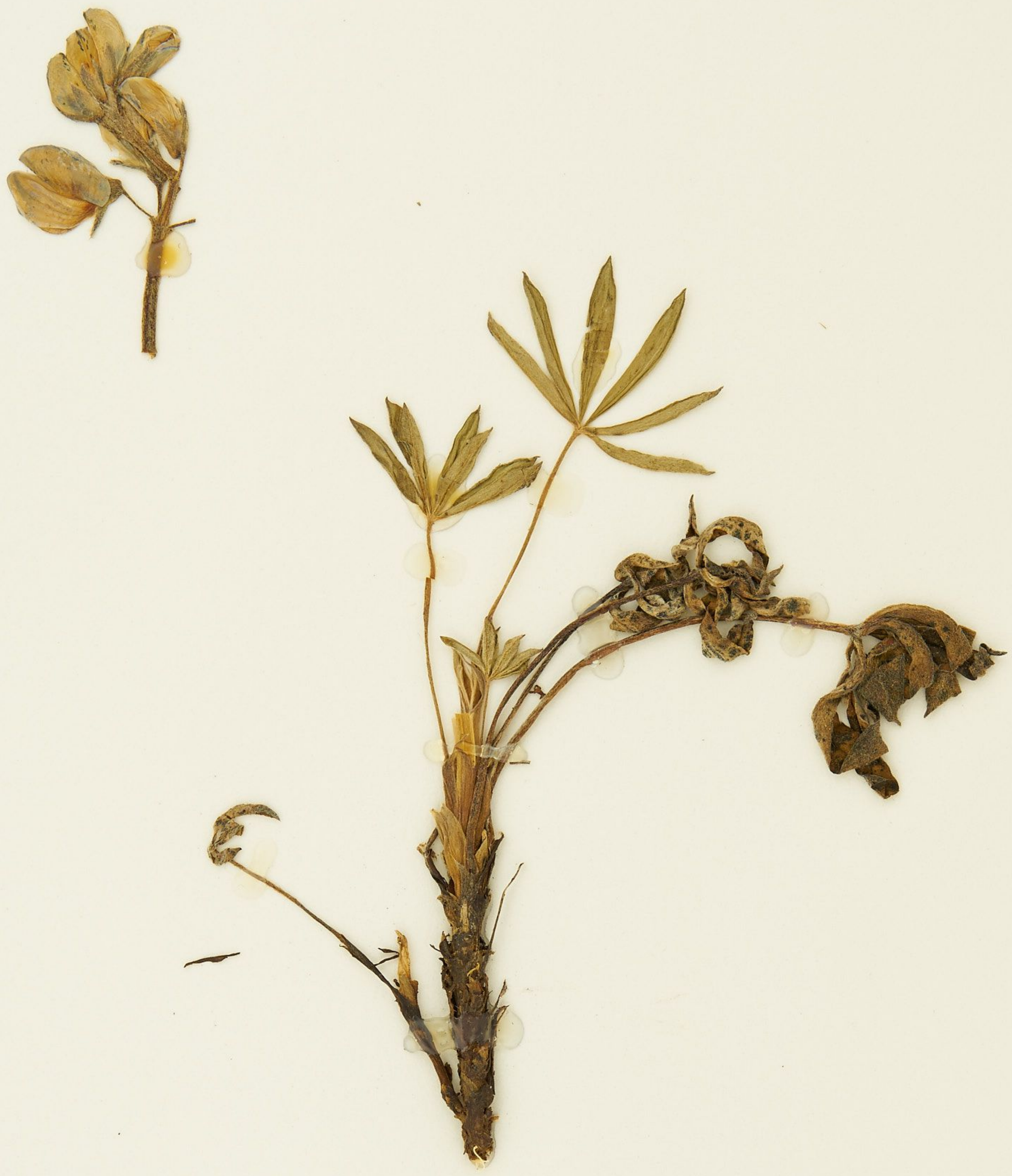

NU

DATA RECORDED
CAN 1999

RARE AND ENDANGERED PLANTS PROJECT

Lupinus arcticus S.Wats.

Det. J.M.Gillett, 1987

National Herbarium of Canada

FRANKLIN DIST, NORTHWEST TERRITORIES, CANADA
Victoria Island

Lupinus arcticus S. Wats.

LONG LAKE — 69 07 N, 104 34 W
Plot 26.

HABITAT: Sedge meadow.

15 JUL 1964 J.D.H. Lambert

DET. BY: J.M. Gillett, 1988

CAN 529355 Fabaceae **REPS:** 1

National Herbarium of Canada

NATIONAL HERBARIUM
529355
CANADA

CAN 10071816

SCANNED 2013

CAN
IMAGED
2018

0 1 2 3 4 5 6 7 8 9 10 11 12 13 14 15

canadian museum of nature
nature
musée canadien de la nature
