# Supplementary material for: Vascular plants of Victoria Island (Northwest Territories and Nunavut, Canada): a specimen-based study of an Arctic flora
Source: PhytoKeys. 2020 Mar 6;141:1–330. doi: 10.3897/phytokeys.141.48810 (PMC7070024; doi:10.3897/phytokeys.141.48810)
Supplement: Supplementary material 7 [file phytokeys-141-001-s007.pdf]

canadian museum of nature
nature
musée canadien de la nature

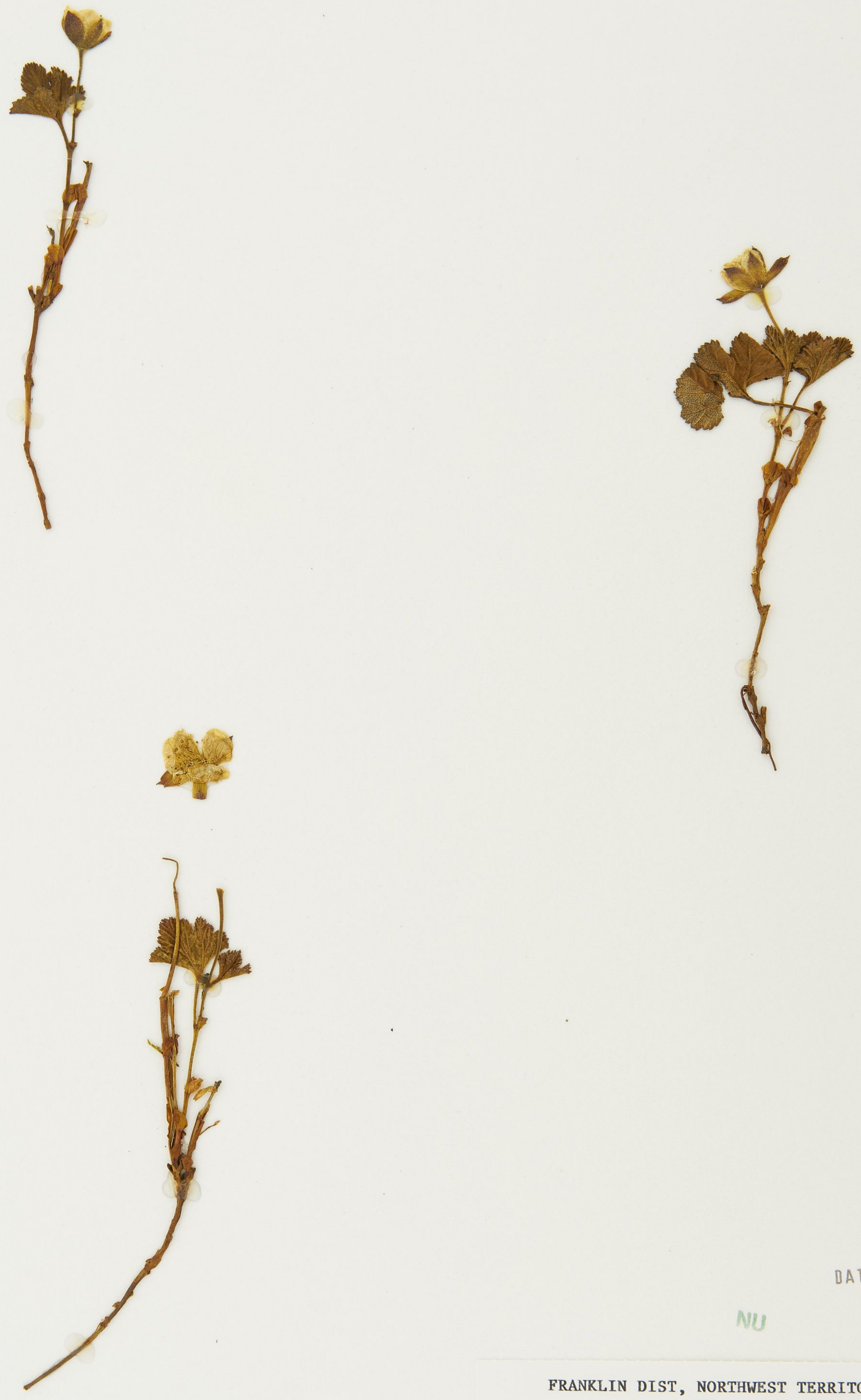

DATA RECORDED
CAN 2000

NU

FRANKLIN DIST, NORTHWEST TERRITORIES, CANADA
Victoria Island

Rubus chamaemorus L.

LONG LAKE 69 07 N, 104 34 W
Plot 22.

14 JUL 1964 J.D.H. Lambert

DET. BY: A.W. Dugal, 1988

CAN 529339 Rosaceae REPS: 1

National Herbarium of Canada

NATIONAL HERBARIUM
529339
CANADA

CAN 10070345

CAN
IMAGED
2018
