# Supplementary figures and images for: Vascular plants of Victoria Island (Northwest Territories and Nunavut, Canada): a specimen-based study of an Arctic flora
Source: PhytoKeys. 2020 Mar 6;141:1–330. doi: 10.3897/phytokeys.141.48810 (PMC7070024; doi:10.3897/phytokeys.141.48810)

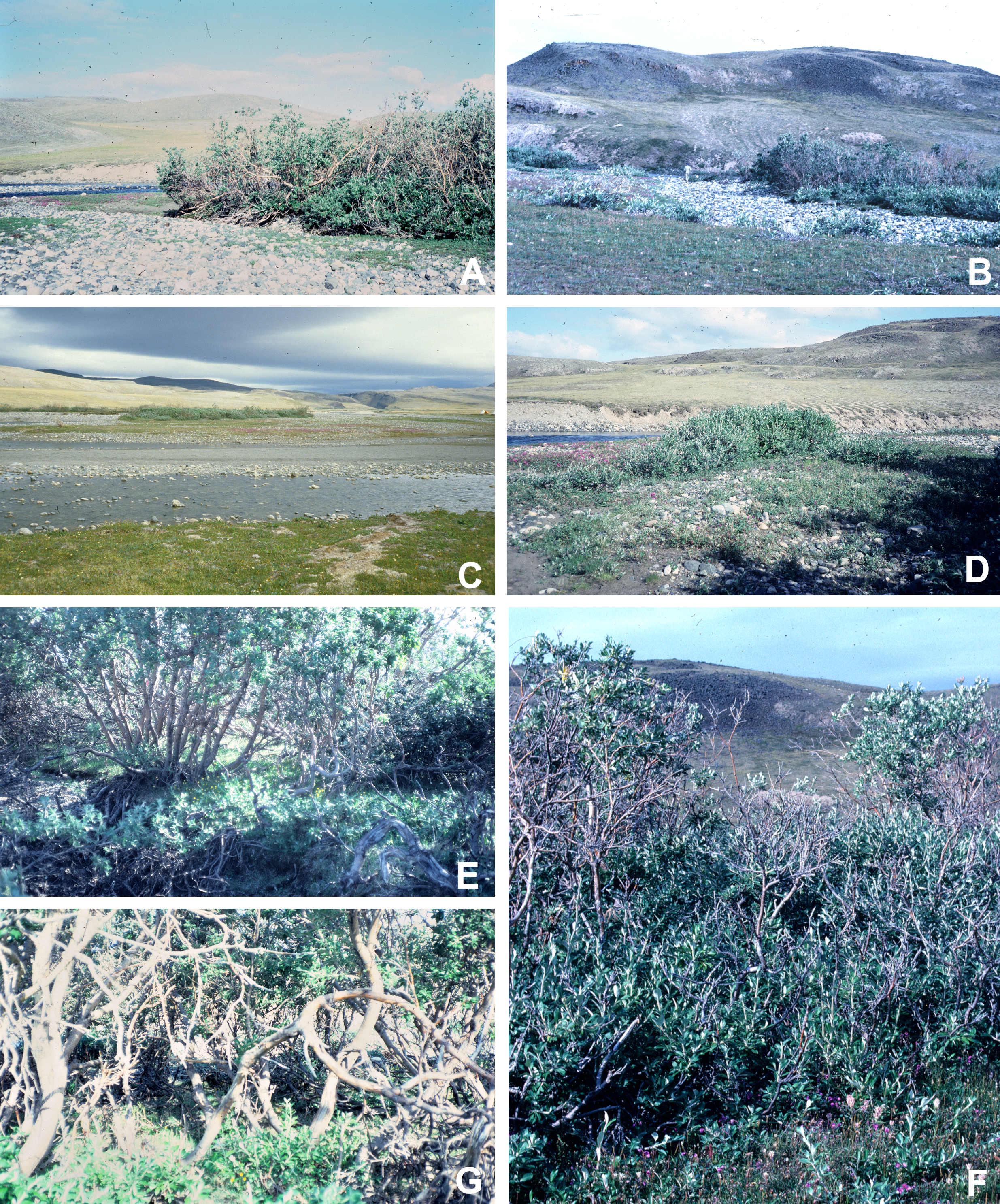

Supplement: Supplementary material 8 [file phytokeys-141-001-s008.png]
