# Supplementary material for: Vascular plants of Victoria Island (Northwest Territories and Nunavut, Canada): a specimen-based study of an Arctic flora
Source: PhytoKeys. 2020 Mar 6;141:1–330. doi: 10.3897/phytokeys.141.48810 (PMC7070024; doi:10.3897/phytokeys.141.48810)
Supplement: Supplementary material 9 [file phytokeys-141-001-s009.pdf]

0 1 2 3 4 5 6 7 8 9 10
cm
copyright reserved
DAO
Canadä

DAO 01-01000684837

AGRICULTURE AND AGRI-FOOD CANADA
DAO
840911
OTTAWA, CANADA
2008

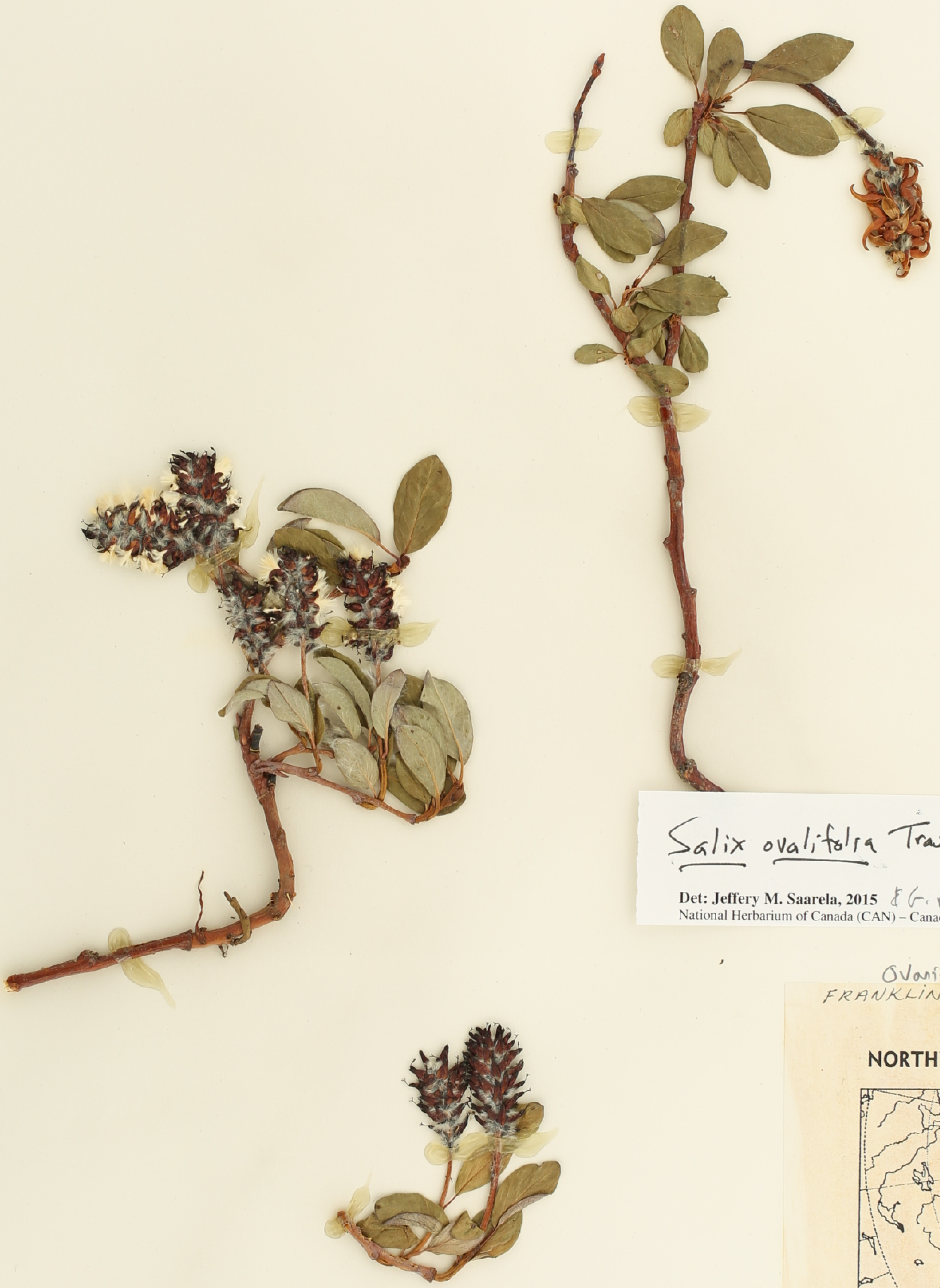

gonoles/carpel

Nunavut Territory

Ania Wojciechowski – May 2016
DAO Herbarium
Agriculture and Agri-Food Canada / Agriculture et Agroalimentaire Canada

Salix ovalifolia Trautv. var. *ovalifolia*

Det: Jeffery M. Saarela, 2015 & G. Argus
National Herbarium of Canada (CAN) – Canadian Museum of Nature

Ovaries glabrous

FRANKLIN DISTRICT

FLORA OF NORTHWEST TERRITORIES

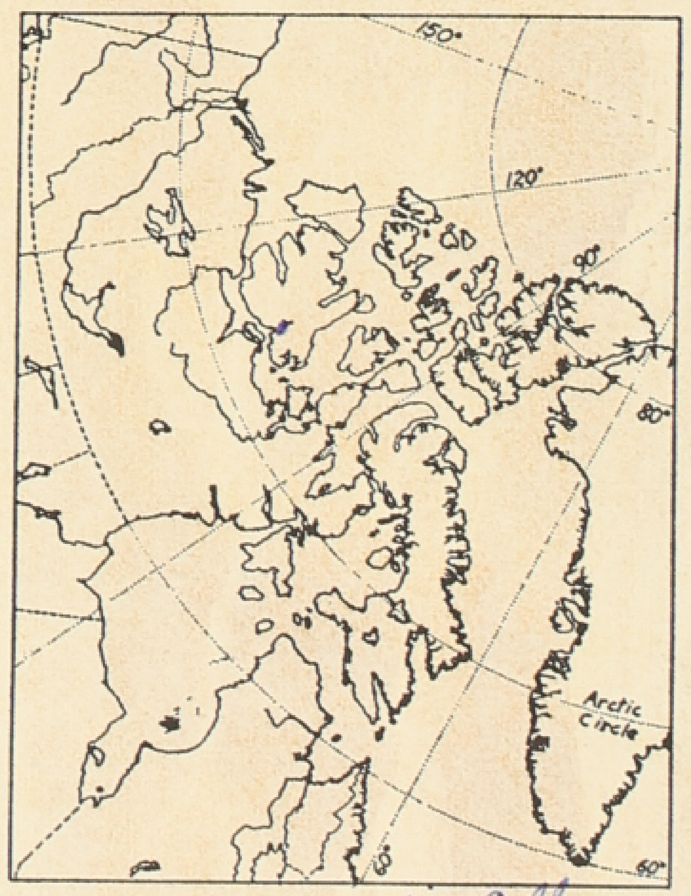

Name: ~~Salix [illegible] Cockerell f. [illegible] (Andersson) [illegible]~~ arctica Pall.

Coll. No.: 2022 Date: 22/7/1962

Locality: ~~[illegible]~~
FRANKLIN DISTRICT, VICTORIA ISLAND
71° 00', 110° 00'

Habitat: On hummocks in

Notes: willow thicket
fruits glabrous

Collected by: Robert Hainault

Determined by: "

Salix arctica Pall.
GWArgus 1997

Victoria Is.
Nunavut

DAO
Imaged

56-2
