# Supplementary material for: Vascular plants of Victoria Island (Northwest Territories and Nunavut, Canada): a specimen-based study of an Arctic flora
Source: PhytoKeys. 2020 Mar 6;141:1–330. doi: 10.3897/phytokeys.141.48810 (PMC7070024; doi:10.3897/phytokeys.141.48810)
Supplement: Supplementary material 10 [file phytokeys-141-001-s010.pdf]

canadian museum of nature
nature
musée canadien de la nature

× one ferruginous hair on adaxial surface

[illegible]

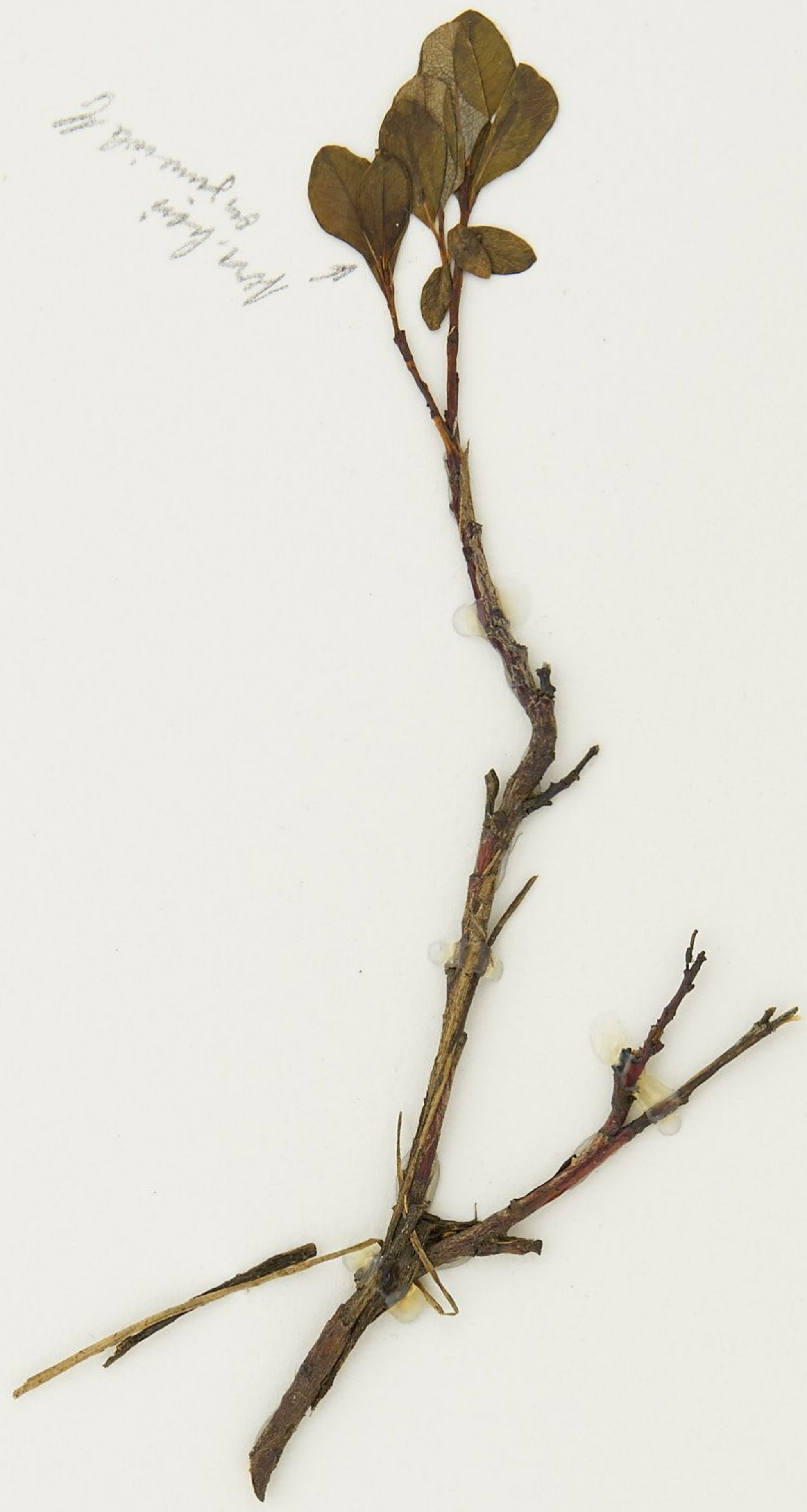

DATA RECORDED
CAN 2000

Possibly: based on two ferruginous hairs!

Salix planifolia Pursh

George W. Argus
National Herbarium of Canada 2014

Salix fuscescens Andersson

George W. Argus
National Herbarium of Canada 1987

lvs l 13-15 mm
w 1.5-2 mm
petioles 7-9 mm
lvs glabrous, one ferruginous hair on adaxial surface
mature lvs hypostomatous
smallest lvs amphistomatous

CAN10024243

NU

FRANKLIN DIST, NORTHWEST TERRITORIES, CANADA
Victoria Island

Salix fuscescens Anderss.

LONG LAKE 69 07 N, 104 34 W
Plot 24.

HABITAT: Sedge meadow.

14 JUL 1964 J.D.H. Lambert

DET. BY: George W. Argus, 1988

CAN 529349 Salicaceae **REPS:** 1

National Herbarium of Canada

NATIONAL HERBARIUM
529349
CANADA

IMAGED 2018

CAN 10024243

MM

0 1 2 3 4 5 6 7 8 9 10 11 12 13 14 15

canadian museum of nature
nature
musée canadien de la nature
