# Supplementary material for: Vascular plants of Victoria Island (Northwest Territories and Nunavut, Canada): a specimen-based study of an Arctic flora
Source: PhytoKeys. 2020 Mar 6;141:1–330. doi: 10.3897/phytokeys.141.48810 (PMC7070024; doi:10.3897/phytokeys.141.48810)
Supplement: Supplementary material 11 [file phytokeys-141-001-s011.pdf]

canadian museum of nature
nature
musée canadien de la nature

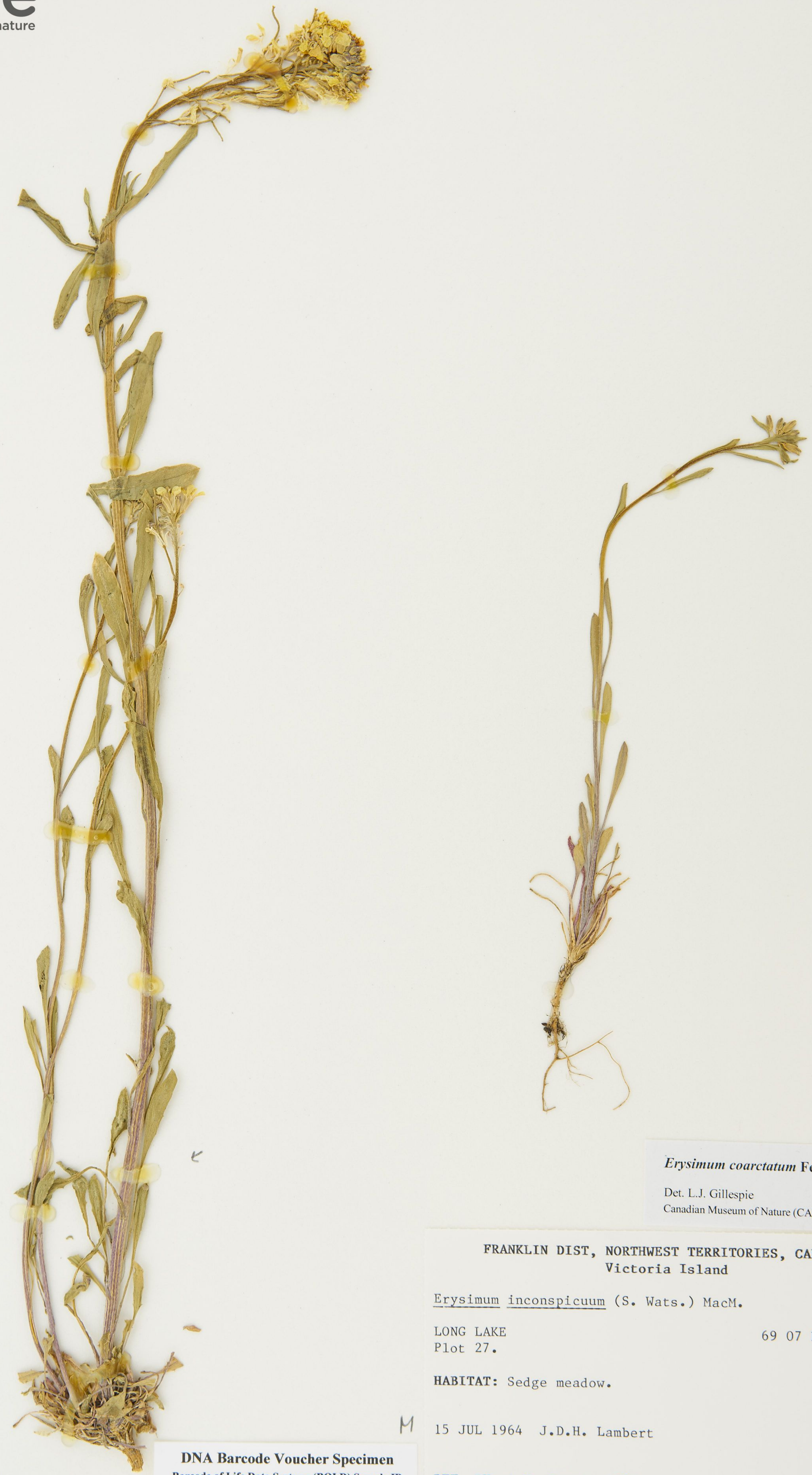

Erysimum coarctatum Fernald

Det. L.J. Gillespie 2011
Canadian Museum of Nature (CAN)

FRANKLIN DIST, NORTHWEST TERRITORIES, CANADA
Victoria Island

Erysimum inconspicuum (S. Wats.) MacM.

LONG LAKE
Plot 27.
69 07 N, 104 34 W

HABITAT: Sedge meadow.

15 JUL 1964 J.D.H. Lambert

DET. BY: A.W. Dugal, 1988

CAN 529364 Brassicaceae REPS: 2

National Herbarium of Canada

CAN 10057248

NATIONAL HERBARIUM
529364
CANADA

CAN
IMAGED
2018

SCANNED 2011

M

DNA Barcode Voucher Specimen
Barcode of Life Data Systems (BOLD) Sample ID
Lambert_sn_CAN529364
Sequenced from tissue removed from sheet (see arrow)
www.boldsystems.org 2010
Canadian Museum of Nature

0 1 2 3 4 5 6 7 8 9 10 11 12 13 14 15

canadian museum of nature
nature
musée canadien de la nature
