# Supplementary material for: Vascular plants of Victoria Island (Northwest Territories and Nunavut, Canada): a specimen-based study of an Arctic flora
Source: PhytoKeys. 2020 Mar 6;141:1–330. doi: 10.3897/phytokeys.141.48810 (PMC7070024; doi:10.3897/phytokeys.141.48810)
Supplement: Supplementary material 12 [file phytokeys-141-001-s012.pdf]

canadian museum of nature
nature
musée canadien de la nature

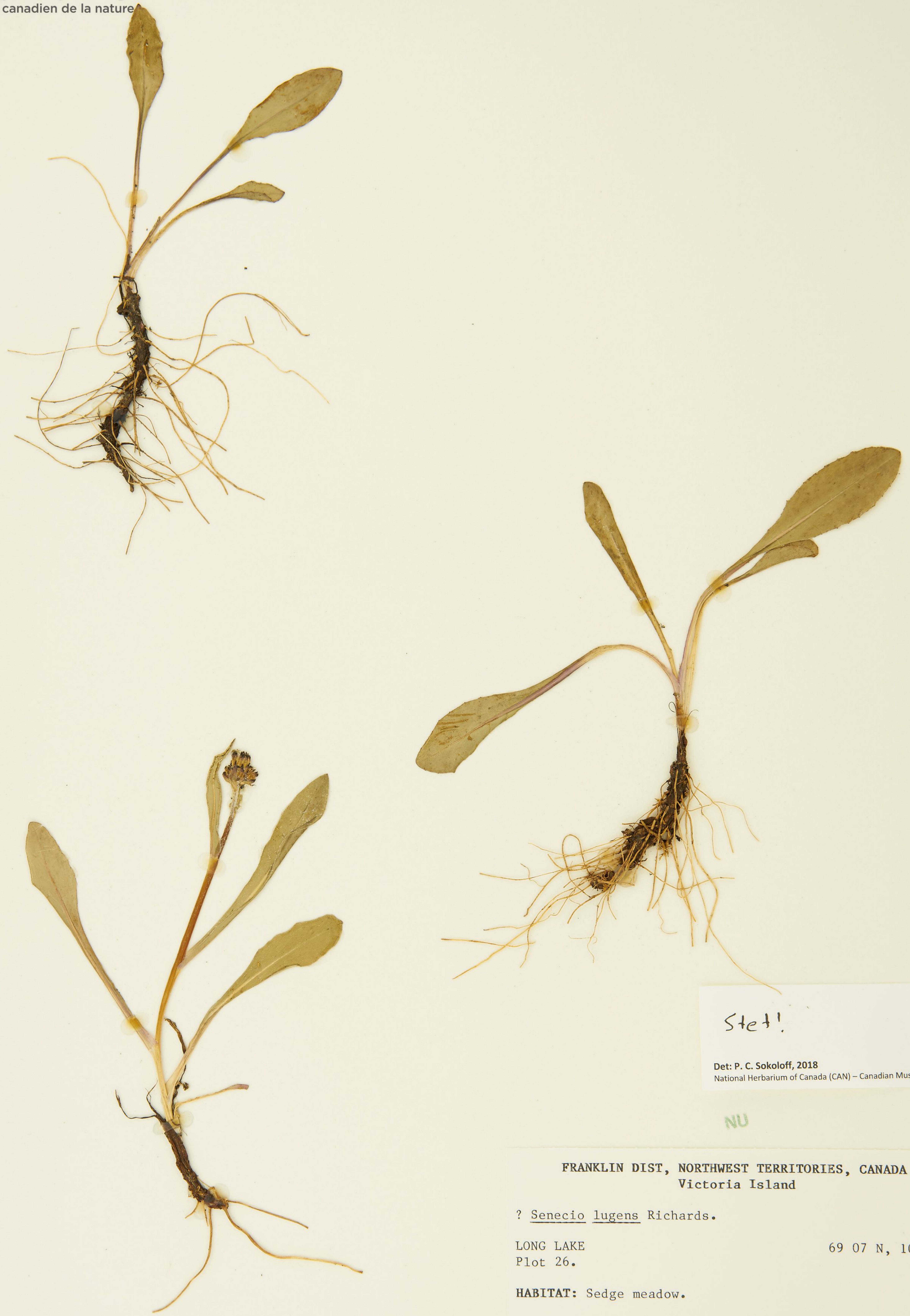

Stet!

Det: P. C. Sokoloff, 2018
National Herbarium of Canada (CAN) – Canadian Museum of Nature

NU

FRANKLIN DIST, NORTHWEST TERRITORIES, CANADA
Victoria Island

? Senecio lugens Richards.

LONG LAKE
Plot 26.

69 07 N, 104 34 W

HABITAT: Sedge meadow.

15 JUL 1964 J.D.H. Lambert

DET. BY: A.W. Dugal, 1988

M

CAN 529359 Asteraceae **REPS:** 1

National Herbarium of Canada

NATIONAL HERBARIUM
529359
CANADA

CAN 10089218

SCANNED 2014

CAN
IMAGED
2018

canadian museum of nature
nature
musée canadien de la nature
